# Importance of RpoD- and Non-RpoD-Dependent Expression of Horizontally Acquired Genes in Cupriavidus metallidurans

**DOI:** 10.1128/spectrum.00121-22

**Published:** 2022-03-21

**Authors:** Cornelia Große, Jan Grau, Ivo Große, Dietrich H. Nies

**Affiliations:** a Institute for Biology/Microbiology, Molecular Microbiology, Martin-Luther-University Halle-Wittenberg, Halle-Wittenberg, Germany; b Institute for Computer Science, Bioinformatics, Martin-Luther-University Halle-Wittenberg, Halle-Wittenberg, Germany; Università Roma Tre

**Keywords:** *Cupriavidus metallidurans*, RpoD, metal resistance

## Abstract

The genome of the metal-resistant, hydrogen-oxidizing bacterium Cupriavidus metallidurans contains a large number of horizontally acquired plasmids and genomic islands that were integrated into its chromosome or chromid. For the *C. metallidurans* CH34 wild-type strain growing under nonchallenging conditions, 5,763 transcriptional starting sequences (TSSs) were determined. Using a custom-built motif discovery software based on hidden Markov models, patterns upstream of the TSSs were identified. The pattern TTGACA, −35.6 ± 1.6 bp upstream of the TSSs, in combination with a TATAAT sequence 15.8 ± 1.4 bp upstream occurred frequently, especially upstream of the TSSs for 48 housekeeping genes, and these were assigned to promoters used by RNA polymerase containing the main housekeeping sigma factor RpoD. From patterns upstream of the housekeeping genes, a score for RpoD-dependent promoters in *C. metallidurans* was derived and applied to all 5,763 TSSs. Among these, 2,572 TSSs could be associated with RpoD with high probability, 373 with low probability, and 2,818 with no probability. In a detailed analysis of horizontally acquired genes involved in metal resistance and not involved in this process, the TSSs responsible for the expression of these genes under nonchallenging conditions were assigned to RpoD- or non-RpoD-dependent promoters. RpoD-dependent promoters occurred frequently in horizontally acquired metal resistance and other determinants, which should allow their initial expression in a new host. However, other sigma factors and sense/antisense effects also contribute—maybe to mold in subsequent adaptation steps the assimilated gene into the regulatory network of the cell.

**IMPORTANCE** In their natural environment, bacteria are constantly acquiring genes by horizontal gene transfer. To be of any benefit, these genes should be expressed. We show here that the main housekeeping sigma factor RpoD plays an important role in the expression of horizontally acquired genes in the metal-resistant hydrogen-oxidizing bacterium *C. metallidurans*. By conservation of the RpoD recognition consensus sequence, a newly arriving gene has a high probability to be expressed in the new host cell. In addition to integrons and genes travelling together with that for their sigma factor, conservation of the RpoD consensus sequence may be an important contributor to the overall evolutionary success of horizontal gene transfer in bacteria. Using *C. metallidurans* as an example, this publication sheds some light on the fate and function of horizontally acquired genes in bacteria.

## INTRODUCTION

Survival of populations requires constant adaptation to an ever-changing environment. An important contributor to resilience of populations is diversity, which allows at least one part of the population to strive or survive when conditions have changed (1). Access to a metagenome through horizontal gene transfer is central to the creation of strain diversity within a bacterial species (2–4). Horizontally acquired genes may reside permanently in a host only if they mediate an additional adaptive function (5), such as the ability to degrade unusual organic substances, to grow as a chemolithoautotroph using molecular hydrogen, or to respond to stress conditions. In contrast to the general stress response system in bacteria, which is deeply interwoven with its regulatory network and metabolic activity, specific stress response systems contain genes for avoidance, alleviation, or damage repair and can be easily used as add-ons after horizontal gene transfer of resistance determinants (6). Indeed, the genes involved in defense mechanisms were those with the highest transfer propensity when the verticality of the overall bacterial genome evolution was determined (7).

To be of any benefit, a horizontally acquired gene must be expressed. It could integrate as gene cassette into an existing integron by double site-specific recombination, which would bring this gene under the control of the respective integron promoter (8). When not expressed as part of an integron, another promoter is needed for the newly acquired gene. Promoter recognition in bacteria is mediated by the sigma factor subunit of the RNA polymerase (RNAP) holoenzyme (9). To guarantee expression of a new gene in a broad spectrum of bacterial hosts, a traveling gene may be accompanied by the gene for a respective sigma factor or use a host sigma factor. The higher the frequency of occurrence of a given host sigma factor, the broader is the host range for a horizontally transmitted gene. The gene with the highest distribution in bacteria is the housekeeping sigma factor RpoD (alternative names, sigma-70 or sigma-55/SigA in some Gram-positive bacteria), which is sometimes the only sigma factor in a bacterial strain (10). RpoD-dependent promoters seem to be universal in bacteria.

As a concluding hypothesis, horizontally transmitted genes should either contain the attachment site for recombination into an integron, travel in horizontally transmittable units together with genes for sigma factors, or contain with high probability a promoter recognized by the RpoD-containing holoenzyme of the RNAP. The latter fact would also exert selection pressure on bacteria to maintain the consensus sequence for RpoD-initialized promoters, because otherwise access to the metagenome would be barred. This would explain the conserved RpoD recognition motifs even between Escherichia coli as a proteobacterium (11) and Bacillus subtilis as a firmicute (12), different phyla of the superkingdom *Bacteria*, which are separated by about 3.2 billion years of evolution (13).

Cupriavidus metallidurans is a metal-resistant, facultative chemolithoautrophic hydrogen-oxidizing bacterium (14–16). Its high-level resistance to the cations of cobalt, zinc, and cadmium (*czc*), copper and silver (*cop*, *sil*), lead (*pbr*), cobalt and nickel (*cnr*), and chromate (*chr*) are located on the horizontally transmittable plasmids pMOL30 and pMOL28 and a chromid, while additional genes involved in metal resistance reside on the chromosome (14, 16–20). The chromosome and the chromid carry several genomic islands also obtained by horizontal gene transfer (21, 22). Two of these genomic islands, CMGI2 and CMGI3, harbor the genes for a membrane-bound hydrogenase, a soluble NAD-reducing hydrogenase, and the enzymes for the Calvin cycle (23), allowing facultative chemolithoautotrophic growth as aerobic hydrogen-oxidizing bacterium.

Horizontally acquired genomic elements are thus central to the metal resistance of *C. metallidurans* and its ability to grow as a hydrogen-oxidizing chemolithoautotrophic bacterium. These elements can be easily lost in *C. metallidurans* mutant strains kept under laboratory conditions (24). *C. metallidurans* contains several sigma factors that might be involved in expression of the genes on the horizontally acquired elements RpoD1 and a paralog, RpoD2, RpoN, RpoS, RpoH, and FliA, as well as 11 extracytoplasmic function (ECF) sigma factors (10, 25, 26). In addition to the metal transportome (16) and cellular metal repository (27), the ECF sigma factors form a third pillar of metal homeostasis (25). Despite a large amount of gene expression and other data for the *C. metallidurans* wild-type strain and many mutants (24), it is not clear how the ECF sigma factors are involved in metal homeostasis, how they interact with other sigma factors, especially RpoD, and how the interaction between horizontally acquired and chromosomal genes works.

We describe here the “ground state” for a deeper understanding of metal resistance and other processes in *C. metallidurans*: the role of RpoD- and non-RpoD-dependent promoters in expression of genes in *C. metallidurans* wild-type cells growing heterotrophically under nonchallenging conditions, especially genes located in horizontally acquired metal resistance determinants. First, all transcriptional start sites (TSSs) were determined, ranked by their strength, and assigned to the previously published expression level (25) of downstream genes. Second, a hidden Markov model (HMM) for discovery of frequently occurring sequence motifs was used. This identified consensus sequences upstream of a large portion of the TSSs that could be assigned to the typical RpoD-dependent promoter motifs. Third, an algorithm was derived to differentiate between strong, medium, weak, or no RpoD-dependent promoters. Next, this algorithm was used to assign all experimentally identified TSSs to RpoD or not RpoD, meaning other sigma factors, including the ECF factors. Last, the resulting data were used to discuss the influence of RpoD and non-RpoD sigma factors on the expression of horizontally acquired genes involved in metal resistance and in chemolithoautotrophic growth as a hydrogen-oxidizing bacterium. We learned from this that an RpoD-dependent promoter may allow an initial expression of a newly acquired gene, but an additional fine-tuning seems to be required to fit its expression into the regulatory network of the new host cell. Other sigma factors and antisense transcription were the tools for such a fine-tuning in *C. metallidurans*. This publication is the first step to unravel the roles of RpoD and other sigma factors in its transition metal resistance, which is in large part mediated by horizontally acquired genetic elements.

## RESULTS

### Transcription start sites in *C. metallidurans*.

The transcriptome of the *C. metallidurans* CH34 wild type growing heterotrophically on gluconate in Tris-buffered mineral salts medium (TMM) had already been determined by transcriptome sequencing (RNA-Seq) and used to predict the operon structure of the genome of *C. metallidurans* in a first approximation (25). Using RNA isolated from *C. metallidurans* CH34 cells cultivated under the same conditions, the transcriptional start sites (TSSs) were additionally determined using the Cappable-seq enrichment strategy (28) as a method to detect all start sites in the wild-type strain under nonchallenging conditions. A nonenriched control library was performed together with the Cappable-seq library. In both libraries, relative read scores (RRS*_io_*s) were obtained, defined as the number of TSS reads (*n_io_*) at position *i* and orientation *o* divided by the total number of reads (*N*) multiplied by 10^6^. The ratio of RRS*_io_*_TSS to RRS*_io_*_control was used as a TSS quality score, with a cutoff of 5 for the TSS quality score of each biological experiment.

The TSS score defines the enrichment ratio of 5′-triphosphorylated RNA characteristic for TSS divided by depleted positions corresponding to processed or degraded 5′ ends (28). The 5′ ends of RNA stemming from degradation should occur with similar probability at each position *N_io_*. Cleavage of RNA by endonucleases cannot happen at the 5′ end resulting from a fresh transcription event because the endonuclease should need at least 1 base upstream and downstream of the cleavage site. Accordingly, RRS*_io_*_control values should be similar for all transcribed positions, with exception of cleavage sites, which should have a very low probability of occurrence close to the 5′ end of a mRNA. Consequently, the denominators of the ratio TSS score = RRS*_io_*_TSS/RRS*_io_*_control should be similar for all transcription initiation sites. Thus, the TSS score is a normalized and corrected value for the abundance of 5′ ends of synthesized transcripts and can be used as a measure of transcription initiation activity at the particular position and orientation.

Since the determined TSS may have an imprecise start (28), all adjacent TSSs in the same orientation were clustered into a single position corresponding to the position with the highest RRS*_io_*. A cluster size cutoff of 5 was used so that all RRS*_io_* values 5 bp upstream and downstream of the position with the highest RRS*_io_* were clustered into this position, meaning all 11 RRS*_io_* values were summarized here. The “real” TSS was therefore the TSS with the highest probability at the position with the highest RRS*_io_* score—maybe also located a few base pairs upstream or downstream of this position with declining probability, but no more than 5 bp away from the indicated TSS position.

With three biological repeats and a cutoff of 10 for the mean value of the TSS quality score from these three repeats, 5,765 TSS signals were found with quality scores between 29,914 ± 8,216 and 13.7 ± 1.2 for TSS_769713-2 upstream of *pilA* (Rmet_0697) and for TSS_2033304-2, respectively, both on the chromosome. These TSSs were ranked according to their mean TSS score (Table 1; see Table S1 in the supplemental material). The abundance of the TSS signals represented by the score indicated a strong (>1,000), medium (100 to 1,000), low (50 to 100), or very low (<50) transcription initiation activity at these positions.

**TABLE 1:** Distribution of the scores of the identified transcriptional start sites^*a*^

TSS score	No. of TSSs	% of TSSs identified
>10,000	19	0.33
3,000–9,999	76	1.3
1,000–2,999	292	5.1
300–999	481	8.3
100–299	1,125	20
30–99.9	1,900	33
<30	1,870	32

a A total of 5,765 transcriptional start sites (TSSs) were identified in RNA isolated from *C. metallidurans* CH34 cells cultivated under nonchallenging conditions in Tris-buffered mineral salts medium in three biological repeats. Two TSSs could not be assigned to the *C. metallidurans* genome. The scores and positions of the remaining 5,763 TSSs are listed in Table S1. A cutoff for the TSS score of 10 was used. The score was defined as the ratio of RRS*_io_*_TSS to RRS*_io_*_control, with RRS*_io_* defined as the number of reads *n_io_* at a position *i* and orientation *o* divided by the total number of reads *N*, multiplied by 10^6^.

Two TSSs were not further considered, leaving 5,763 associated TSSs. Only TSSs were considered that appeared in all three biological repeats. TSS names follow the schema TSS_<position><direction><replicon>, where the replicon is identified by its last digit (CP000352 for the chromosome, CP000353 for the chromid, CP000354 for plasmid pMOL30, and CP000355 for plasmid pMOL28) and the direction is specified as “−” or “+.” For instance, TSS_769713-2 refers to the TSS at position 769713 on the negative DNA strand of replicon CP000352. The newly identified TSSs were combined with the information from the RNA-Seq analysis (25), the transcript frequency measured as the mean NPKM value of a gene (i.e., nucleotide activities per kilobase of exon model per million mapped reads), the 5′ and 3′ untranslated regions (5′ UTR and 3′ UTR, respectively), and the operon model, yielding a transcription landscape of *C. metallidurans* CH34 cells growing under nonchallenging conditions (see Data Set S1 in the supplemental material).

The activity of a promoter could influence the amounts of transcripts of a gene downstream of this promoter. The NPKM value as a measure of the amount of transcript of a given gene, which was also determined in *C. metallidurans* strain CH34 cultivated in TMM (25), was plotted against the TSS score (see Fig. S1 in the supplemental material). In this double log_10_ plot, the relationship of both data was very weak, with a general increase of the NPKM value with the TSS score. On the other hand, the ratio log_10_ (NPKM)/log_10_ (TSS_score) of all TSSs and associated NPKM values for downstream genes was 0.94 ± 0.48, indicating overall similar values of the NPKM value of a given gene with the score of a transcriptional start site upstream of the gene. About one-third of the TSSs had a score above 100, or log_10_ = 2 (Table 1; Fig. S1), and the overall number of TSSs was in the same range as the number of open reading frames in the genome of *C. metallidurans*. Since most genes in this genome were organized in multicistronic operons (25), a TSS with a low score may be product of an intermediary start site and therefore not responsible for the transcript content of a downstream gene, because it is actually transcribed from a promoter further upstream. Figure S1 thus shows the effect of multiple promoters within and upstream of operons.

The mean ratio log_10_ (NPKM)/log_10_ (TSS_score) = 0.94 ± 0.48 indicates that the transcript abundance represented by the NPKM value should correspond with a deviation of 10^0.48^ = 3 to the transcription initiation activity given by the TSS score. This information can be used to identify the TSS responsible for or contributing to the transcript level of a gene. A contributing or responsible promoter should have a TSS score between NPKM/3 and 3 times the NPKM value. Moreover, low NPKM values downstream of TSSs with a high score may indicate mRNA instability. A high NPKM value of a gene without a high-scoring TSS upstream may be the result of increased mRNA stability, or upstream transcription events may continue into the respective gene. In the latter case, upstream genes on the same DNA strand should exhibit NPKM values similar to that of the gene in question.

### RpoD promoter motifs in *C. metallidurans*.

**FIG 1:**
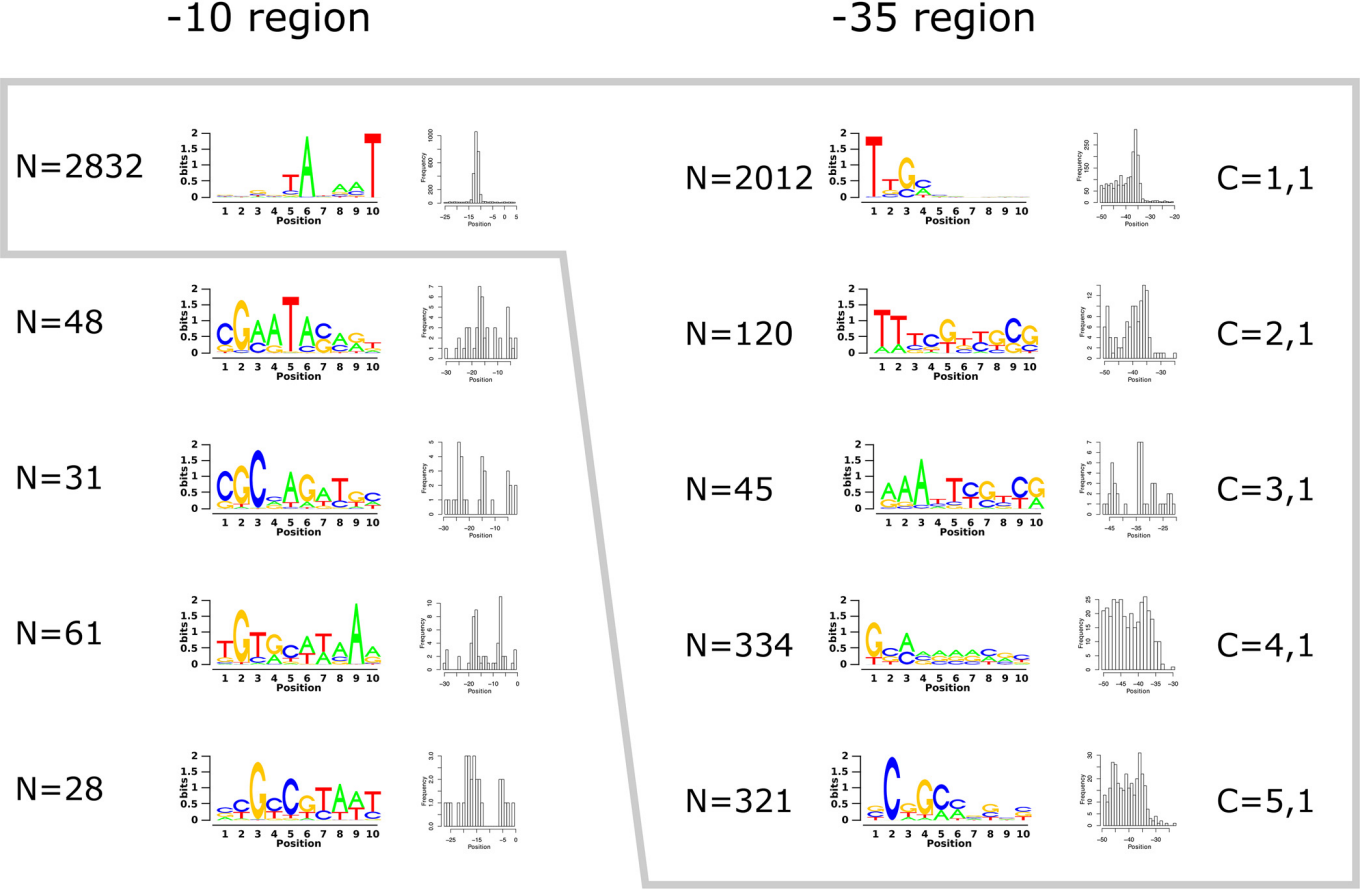
Overview of the motifs discovered in the −10 and −35 regions. For each motif, a sequence logo and a histogram of the positions of motif matches are shown. In the −10 region, 5 motifs were discovered (from the top to the bottom, components 0,1 to 0,5), of which one (component 0,1 at the top) covered the majority of TSSs and showed strict positioning. Only promoters of TSSs containing this motif were considered when searching for a second, upstream motif. Of the 5 motifs discovered, only two components (C = 1,1 and C = 2,1) were clearly positioned and showed similarities to known −35 consensus sequences.

The 3,000 TSSs with the highest scores from TSS_769713-2 mentioned above to TSS_1343494+2 (score, 56 ± 7) were searched for patterns in the −90 to +10 region of the respective TSS with a custom-built motif discovery software based on hidden Markov models (see Fig. S2 in the supplemental material). The software was first set up to discover up to five motifs in the −50 to +10 region of the 3,000 TSSs (Fig. 1), aiming for a putative motif around the −10 position upstream of a TSS. Of the 3,000 TSSs, 2,832 shared the same pattern clearly positioned around the −10 position upstream of the TSS site (−10 model, component 0,1). The remaining 168 TSSs were rather evenly distributed across the remaining 4 patterns (components 0,2, 0,3, 0,4, and 0,5), which were not clearly positioned (Fig. 1), indicating that the patterns of the DNA sequences upstream of these 168 TSSs may have been artifacts rather than −10 regions for sigma factors different from RpoD.

The remaining 2,832 TSSs were the input to the software to again search for up to 5 motifs upstream of the previous one (Fig. 1). Five combinations of patterns upstream of the component 0,1 motif were identified. Among these, 2,232 TSSs could be assigned with a high score of the motif model to consensus motifs for the −35 model number 1 or 2 in combination with the previously selected −10 model number 1 (component 1,1 or 2,1) (Fig. 1). The majority of 2,012 TSSs belong to component 1,1, while 120 TSSs belong to component 2,1. Not all of these promoter models displayed correctly positioned −10 and −35 motifs.

Because of their frequent occurrence, the patterns of components 1,1 and 2,1 identified by the motif discovery software within a region −90 to +10 bp of the TSS may represent the −35 and −10 regions of RpoD-specific promoters in *C. metallidurans*. If this is the case, TSSs upstream of housekeeping genes should display a 1,1 or 2,1 pattern in the DNA sequences at the −35 and −10 positions, respectively. A total of 48 genes involved in ribosome biosynthesis or in general for translation, integration host factor (IHF), DNA replication, cell division, ATP biosynthesis, including respiratory chain components and F_1_F_0_ ATPase, and tricarboxylic acid cycle indeed carried pattern 1,1 or 1,2 motifs upstream (Table 2), eight motifs 1,2, and 40 motifs 1,1.

**TABLE 2:** DNA sequences upstream of transcriptional start sites corresponding to −35 model number 1 or number 2 combined with −10 model number 1 upstream of housekeeping genes^*a*^

TSS rank	Start at −35	Sequence for −35 model	Start at −10	Sequence for −10 model	Locus tag	Gene	RpoD score
2,376	−34	TTGACAAGGA	−13	TCTATAGTTG	Rmet_R0003	*rplY*	13.6
2	−35	TTGCAACGGC	−15	TGTATAATTC	Rmet_6415	*rplM*	12.6
53	−34	TTGATTGATA	−13	GCTAATATCG	Rmet_0722	*rpsA*	10.6
**18**	−36	TTTGTTTGGG	−14	GCTATACTCT	Rmet_0749	*rimM*	10.9
1,211	−37	TTGCCGGTCC	−15	CTTAACATCG	Rmet_0920	*rpsO*	11.4
886	−36	TTGCACGCGA	−14	GTTATACTTG	Rmet_R0015	*thrS*	12.4
714	−37	TTGCGTAACG	−16	TATAAAATTC	Rmet_1161	*infC*	10.8
2,673	−36	TTGATAACGA	−13	GTCAAATTCA	Rmet_1162	*rpmI*	11.4
**68**	−35	TTGCGTTCCG	−14	AAGATACTGG	Rmet_1166	*ihfA*	10.6
**245**	−39	TTTGTTTGCG	−15	GCTATAATTG	Rmet_1975	*dnaB*	6.9
49	−35	TTGCGGGAAG	−13	GCTATACTCG	Rmet_1979	*rpsF*	12.2
1,413	−36	TGGAAAGCCT	−15	GTTACAATGC	Rmet_2134	*trxA*	12.3
14	−35	TGGCGCACCG	−15	GCTATGATGC	Rmet_2135	*rho*	9.1
31	−35	TTGCACCTGC	−13	GCCATAATCC	Rmet_2137	*rpmE*	12.2
7	−35	TTGATTCGTC	−13	TCTATAATGT	Rmet_2870	*rpmB*	14.2
38	−36	TTGCCTGTCC	−14	TGTATAATCG	Rmet_2904	*rpsT*	14.4
33	−35	TTGCAGTTTT	−14	TTTATAATCA	Rmet_3106	*rplU*	12.6
1,622	−39	TGGCGCCGTG	−15	GATAAAGTGG	Rmet_3291	*rpoA*	4.9
60	−34	TTGCAAGTCC	−14	GCTATAATCC	Rmet_3307	*rplN*	11.6
526	−34	TTGCCCTTTC	−13	GTTATAGTGT	Rmet_3317	*rplC*	11.6
219	−34	TTGACCATTG	−13	GCTAGAGTGC	Rmet_3327	*rpsL*	11.6
2,084	−37	TTCAGTTCCG	−14	GGTATCATCC	Rmet_3336	*rplJ*	9.9
11	−35	TTGACAGCCA	−14	ACCATAATCA	Rmet_R0059	*tRNA*	14.6
2,648	−36	TGGAAGTGGT	−13	TCTAAGCTTC	Rmet_R0060	*tRNA*	8.9
2,789	−36	TTGACGGGGA	−13	TGGATGATGT	Rmet_R0063	*tRNA*	12.4
2,182	−35	TTGTTGGGGA	−14	AGTAACGTAG	Rmet_R0053	*tRNA*	9.6
1,312	−35	TTGAACTGAA	−11	CTCAGATTGA	Rmet_R0063	*tRNA*	9.2
235	−34	TTGACGAAAC	−13	TGCATAATCT	Rmet_R0064	*tRNA*	12.6
19	−35	TTGACTGATG	−13	AATAGAATCG	Rmet_3501	*atpI*	14.2
1,108	−39	TGGCGCGGCT	−13	GATACGCTGG	Rmet_3501	*atpI*	1.9
15	−35	TTGTTCAGGT	−14	CATATAATGC	Rmet_0260	*coxB*	12.6
**2,505**	−33	TTTCTCCCCC	−13	GTGAAATTGG	Rmet_0927	*nuoA*	5.1
1,708	−33	TTCATTGTTC	−13	GTGATGCTGA	Rmet_2039	*cco*	6.1
389	−36	TTGCCCTGAA	−14	GGCACTATCA	Rmet_2188	*ftsH*	11.4
2,159	−34	TTGTTATCTC	−14	CCCAACTTGT	Rmet_2188	*ftsH*	7.6
1,336	−34	TTGCAAAGAC	−12	CGTACAATGC	Rmet_2621	*zupT*	12.2
2,410	−34	TTGTCAGGGG	−13	AGCACACTGG	Rmet_2623	*ndh*	10.6
**577**	−36	TTGCTTTTCG	−14	GACAGAATGT	Rmet_3227	*sspA*	11.4
**317**	−37	TATCGTCGCC	−15	GGTATAATTT	Rmet_3230	*petA*	9.4
1,092	−36	TTCCCACGAT	−13	GGCATGATCA	Rmet_3230	*petA*	10.9
**977**	−39	TATCGGCGCG	−14	GTTATCCTGC	Rmet_2031	*infB*	2.4
391	−35	TTGATACCGG	−13	CCTACACTAC	Rmet_2192	*greA*	13.2
861	−36	TTGACATCAA	−15	CCTACACTCG	Rmet_2489	*mdh*	13.8
**165**	−38	TATTGGTGCG	−13	GCTAAAATCA	Rmet_2486	*sdhC*	4.4
2,042	−33	TGGCGTGCCC	−13	TGCATAATAT	Rmet_2895	*icd*	7.1
2,941	−38	TTGCACCTCC	−14	GGCAAAATTC	Rmet_3729	*icdA*	7.4
1,295	−36	TTGCATCGAT	−15	TCTAGTATAT	Rmet_4268	*citA*	10.8
723	−35	TTGATCTGGC	−14	TCTACAATCA	Rmet_5296	*acnB*	12.6

a The 3,000 transcriptional start points identified in *C. metallidurans* CH34 with the highest TSS scores (Table S1), as indicated by the position in the score ranking, were searched with a pattern-finding software: 2,832 were assigned to −35 and −10 models, and 2,132 TSSs with DNA sequences corresponding to −35 model number 1 (rank in standard black letters) or 2 (rank in boldface letters) in combination with −10 model number 1 were further characterized (Table S1). Among these, 48 putative promoter sequences upstream of housekeeping genes were used to develop a score for RpoD consensus motifs in *C. metallidurans.* The results are indicated in the last row on the right hand. Strong RpoD-dependent promoters according to the RpoD score are on a white field, medium strong one are shaded in light gray, and weak are shaded in medium gray.

Within these 48 patterns upstream of housekeeping genes 100 bp around their TSS, the main distance between the −35 model number 1 or 2 and the TSS was −35.6 ± 1.6 bp, with a minimum distance of −33 and a maximum distance of −39. Although a motif discovery software without strict constraints on motif positioning was used, this corresponded perfectly to the position of the −35 region of all RpoD-dependent promoters. Among the 48 patterns, 33 motifs began with a “TTG”; among these were 4 motifs with a distance of −34 bp. This TTG corresponds to the general consensus motif of the −35 region of RpoD-dependent promoters, “TTGACA” (11, 12), which was also identified in bacteria by other methods (29, 30). The lack of the “TTG” triad in the −35 element found in the betaproteobacterium Burkholderia cenocepacia (31) could not be confirmed in *C. metallidurans*, although both species belong to the same family, *Burkholderiaceae*.

The pattern recognition software identified model 1 patterns at a mean position of −13.7 ± 0.9 bp among the DNA sequences upstream of TSSs for housekeeping genes (Table 2). In all of these patterns, motifs corresponding to the consensus −10 region of RpoD-dependent promoters “TATAAT” (11, 12) were indeed identified, but they started at the third position of the −10 model number 1 sequences (Table 2). The first 2 bases in the −10 model number 1 sequences may correspond to the last 2 bases of the extended −10 motif “TGx” (32), which shifts the beginning of the −10 region to 11.7 ± 0.9 bp and the distance between the last bp of the −35 motif and the first bp of the −10 motif of 15.8 ± 1.4 bp. All 48 patterns upstream of the housekeeping genes contained an “A” at the second position of the −10 region and a “T” at position number 6. From the positions and conservation of the DNA sequences within the −35 and −10 region, these 48 TSS patterns corresponded most likely to RpoD-dependent promoter motifs in *C. metallidurans*.

### A scoring algorithm for RpoD-dependent promoter motifs in *C. metallidurans*.

A scoring schema for RpoD-dependent promoter motifs in *C. metallidurans* was derived to discriminate between strong, medium, and weak RpoD-dependent promoters on the one hand and non-RpoD-dependent promoters on the other hand. This algorithm included information not considered by the motif discovery software, namely, the correspondence to −10 and −35 regions of RpoD-dependent promoters upstream of housekeeping genes and the position and distance of the two motif matches. When present in a given DNA sequence upstream of a TSS, the first T of the TTGACA −35 motif counted as 3, the subsequent TG as 1.5 each, and the following ACA as just 1 per conserved nucleotide. This agreed also with the component 1,1 and 1,2 patterns (Fig. 1). In the −10 T**A**TAA**T** motif, all counted as 1, with exception of the highly conserved second A and last T (boldface) (see also Fig. 1, component 0,1 on the left upper corner), which counted as 2. From the resulting sum with a maximum value of 17, the distance to the mean −35 position at −35.6 bp and the absolute difference to a distance of 15.8 between the −35 and −10 sites was subtracted, giving a maximum score of 16.4. The mean score of the 48 housekeeping promoters (Table 2) was 11.2 ± 2.7, with a maximum of 14.6 and a minimum of 1.9. Two motifs were below the mean value − 2 standard deviations (SD) due to a distance of −39 bp of from the −35 motifs and a low degree of conservation of the −35 sequence, corresponding to a TSS upstream of *atpI* and of *infB* (Table 2). These promoters should be very weak RpoD-dependent promoters, and in the case of the *atp* operon, transcription should start from another, strong RpoD-dependent promoter with a score of 14.2 (Table 2).

### Assignment of the TSSs in nonchallenged *C. metallidurans* cells to RpoD- and non-RpoD-dependent promoters.

The motif discovery and RpoD scoring algorithm was used for all 5,763 TSSs (Table S1) that were integrated into the *C. metallidurans* transcriptional landscape (Data Set S1). Moreover, the distance of the −35 and −10 motifs from their associated and experimentally determined TSSs was also included in this analysis to understand the biological meaning of these results: RNA polymerase (RNAP) holoenzyme (RNAP core enzyme plus a sigma factor) binds to DNA containing a promoter motif in one or two steps: first to the closed binary complex. Subsequently, the enzyme-DNA complex is reorganized into the open binary complex by melting the DNA double strand, which brings the DNA template strand into the active site (33, 34). In all cases (meaning when a TSS was experimentally determined), some RNAP holoenzyme must have been able to recognize the DNA region upstream of the TSS as promoter motif. Among the sigma factors present in *C. metallidurans*, RpoN is the only one that uses a −24 and −12 promoter element and needs an ATP-hydrolyzing activator to mediate formation of the open complex (35). RpoN promoter motifs were not identified by the algorithm used here (Fig. 1). Binding of all other RNAP holoenzymes requires binding of the conserved sigma factor regions 4.2 and 2.4 to the promoter regions −35 and −10, which have to be correctly positioned and spaced with respect to each other and the transcriptional start site (34, 36–39). Binding should be strongest when both regions interact with correctly spaced −35 and −10 sites (40). With a larger or smaller distance, the sites are no longer on the same face of the DNA, unless activators such as MerR-type proteins facilitate binding of the holoenzyme by twisting the DNA (41). As these MerR-type regulators demonstrate, RNAP holoenzyme may also bind to DNA just by interaction with the −35 site (42) in a closed binary complex that is unable to move into the open binary complex. This may result in the observed half of the RNAP molecules bound to the DNA without transcribing it (43, 44): among these, 23% are promoter-bound holoenzymes. Nevertheless, during sigma factor competition (45), these promoters are blocked for the access of other RNAP holoenzymes—for instance, those that use other sigma factors.

A TSS upstream sequence with a high RpoD score and correctly positioned −35 and −10 sites should be a strong indicator for an RpoD RNAP holoenzyme as being responsible for the observed transcription initiation event. With decreasing RpoD score but still correctly positioned −35 and −10 sites, the probability increases that RNAP holoenzymes with other sigma factors might use a DNA sequence upstream of a determined TSS as promoter. In these cases, the −35 and −10 sites could be hybrids of the consensus sequences of more than one sigma factor. As another possibility, a promoter may be influenced by activators or the upstream element (46, 47). The presence of no RpoD score at all should indicate a non-RpoD RNAP as responsible holoenzyme for the observed transcription initiation event.

TSSs were counted as “RpoD dependent” when the −35 motif was between positions −31 and −39 upstream of the TSS and the −10 motif between positions −10 and −18. The mean RpoD score of the TSS upstream of housekeeping genes (Table 2) was 11.2 ± 2.7. This was used to judge candidates for an RpoD-dependent promoter as “strong” (where an RpoD score >6.35 = mean value − 1.8-fold deviation [1.8-fold since another scoring algorithm was initially used that was based on the 3,000 TSSs with the highest score]), “medium” (between 3.28 and 6.35), “weak” (between 0.21 and 3.28), and “none” (below 0.21). Using this score, 2,094 TSSs (36.3%) within the screening window for the positions of the −35 and −10 sites were predicted to be strong RpoD-dependent promoters, most with model components 1,1 and 2,1: 454 were medium, 184 were weak, and 52 were probably not RpoD dependent (Table 3). The percentage of the components 3,1, 4,1, or 5,1 in comparison to the components 1,1 or 2,1 increased from the TSSs with a strong RpoD score from just 8.6% via 51.1% for weakly scoring to 94.2% among the TSSs that were not RpoD dependent. This may indicate an increasing probability that the other sigma factors may be involved in the transcription initiation event and may use recognition sequences associated with component 3,1, 4,1, or 5,1. In total (Table 3), and also considering the TSSs upstream of housekeeping genes (Table 2), there were 2,094 TSSs with a strong RpoD score and 454 TSSs with a medium RpoD score and correctly positioned −35 and −10 motifs (2,548 TSSs) with high-probability RpoD-dependent promoters, 184 TSSs with a weak RpoD score with low probability, and 52 TSSs with an RpoD score beyond weak with a high-probability of no RpoD-dependent promoters.

**TABLE 3:**
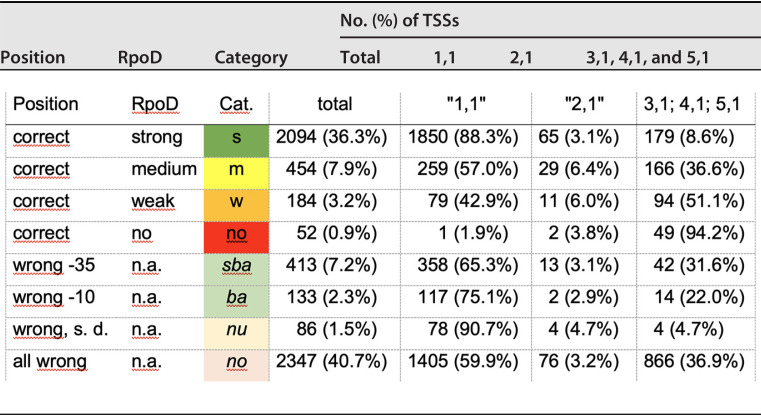
Distribution of the scores of the identified transcriptional start sites^*a*^

a A total of 5,763 of the 5,765 TSSs were analyzed for RpoD promoter consensus motifs. “Position” indicates if the position of the −35 motif was between −31 and −39 and the −10 motif between −10 and −18 or not. The result of the RpoD scoring is shown in the next row, followed by the total number of TSSs in this category and the distribution of these TSSs to the components 1,1 or 2,1 or the remaining components, 3,1, 4,1, or 5,1 (Fig. S3). “Category” shows the category of the TSS as indicated in Fig. S1 with the corresponding cell color in case of categories s, m, w, no, and nu; for sba and ba, the cell colors of the (s)-sba and (s)-ba sites in Table S1 are indicated. The percentage of the total number of TTSs is that of 5,763 TSSs, and that of the components is the portion of TSSs of this category. “Wrong, s.d.” means both positions are wrong, but positions −35 and −10 are either both too far upstream or downstream of the TSS, and “all wrong” indicates both are too close together. n.a., analysis of the RpoD score not shown.

Evaluation of the importance of the sequence motifs with incorrectly positioned −35 and −10 sites needs to consider again the uncertainty of the TSS scoring method used. All identified TSSs 5 bp up- or downstream of the position with the highest TSS score were pooled into the position of the TSS with the highest TSS score, and this position was indicated (Table S1). The “real” TSS may be up to 5 bp up- or downstream of the indicated TSS position, with declining probability, which depends on the steepness of the score-position curve around the position with highest TSS score. This indicates that a −10 site with a high RpoD score at a position just adjacent to the upper or lower limit of the search window may be nevertheless an RpoD promoter site. A −35 site with a few base pairs too close to or too far from the −10 site with a high RpoD score may bind the RpoD-dependent RNAP holoenzyme into the closed binary complex but block the promoter due to a slow transition into the open binary complex. Alternatively, the −35 and −10 motifs for RNAP holoenzymes using alternative RpoD-related sigma factors may be correctly positioned and responsible for the transcription initiation event instead.

The TSSs with a high-scoring model for the −35 and −10 motifs, albeit at positions that did not agree with the current models for transcription initiation in bacteria (33, 34), are also listed in Table 3 and in Table S1. They were sorted into 413 “sba” (“sliding-blocking-another sigma factor”) TSSs with not correctly positioned −35 motifs but correctly positioned −10 motifs, 133 “-ba” (“blocking or used by another sigma factor”) TSSs with correctly positioned −35 sites but incorrectly positioned −10 sites, 86 “nu” (possibly “not used by RpoD”) TSSs with −35 and −10 motifs just outside the screening window but with a correct −35/−10 distance, and 2347 TSSs with RpoD motifs not at all correctly positioned (Table 3). All of them were sorted into subgroups with (s) = strong, (m) = medium strong, and (w) = weak RpoD scores. Depending on the RpoD score and the position of the −35 and −10 sites, these TSSs were assigned to RpoD and non-RpoD promoter motifs.

In summary, 5,765 transcriptional start sites were experimentally determined in the transcriptome of *C. metallidurans* cells, and 5,763 could be assigned to the genome of this bacterium. A TSS quality score was obtained for each TSS as a measure of the transcription initiation activity at this position. Among the 5,763 positions, patterns were obtained with a pattern recognition program obtained in the region between −90 and +10 around the start position patterns in the −35 and −10 regions. In 2,094 TSSs, a DNA sequence close to the consensus sequence TTGACA was found at position −35.6 and a sequence similar to TATAAT at a distance of 15.8 bp from the −35 site, indicating a high probability that these TSSs were strong candidates for an RpoD-dependent transcription initiation event. An additional 454 TSSs with medium RpoD score and correctly positioned −35 and −10 sites and 11 (s)-sba sites and 13 (s)-ba sites should be RpoD-dependent promoters with high probability, summing up to 2,572 of 5,763 (44.6%) TSSs. Start sites with a low probability of being RpoD associated include the 184 with correctly positioned −35 and −10 sites but a weak RpoD score, plus 107 (m)-sba, 65 (m)-ba, and 17 (m)-nu sites, summing up to 373 TSSs (6.5%) that may be RpoD dependent with a low probability. The remaining 2,818 TSSs (48.9%) had a high probability of having no RpoD-dependent promoters. About half of the TSSs in *C. metallidurans* cells grown under nonchallenging conditions and in the exponential phase of growth were RpoD dependent, and the other half were non-RpoD-dependent. These promoters might be under the control of RpoD2, RpoN, FliA, RpoS, RpoH, or the 11 ECF sigma factors. At this point, all experimentally determined TSSs in *C. metallidurans* CH34 wild-type cells grown under nonchallenging conditions were sorted into the categories “RpoD-dependent,” meaning housekeeping functions, or “non-RpoD-dependent.”

To understand their biological meaning, these data can now be combined with expression data to obtain insights into the generation of the transcriptome in *C. metallidurans*. A TSS with a score between NPKM/3 and 3-fold NPKM should be a candidate for the transcription initiation events responsible or contributing to the transcript level of a gene downstream of the TSS. The RpoD score revealed whether RpoD RNAP may have been responsible for the transcription initiation event. When a TSS possesses a low TSS score but a high RpoD score, this would mean that RpoD RNAP was not able to bind to this site, indicating occupation of the promoter, for instance, by regulation of proteins or RNAs, or transcription and subsequent translation events coming from upstream TSSs.

### Importance of predicted RpoD-dependent promoters in *C. metallidurans*.

Since the hypothesis was tested that horizontally acquired genes may especially use RpoD-dependent promoters, 794 TSSs associated with the genomic islands on the chromosome (21) or the plasmids pMOL30 and pMOL28 (Rmet number on a blue field in Table S1) were analyzed for their association with RpoD. Of these 794 TSSs, 293 had strong RpoD motifs (36.9%), 68 medium motifs (8.6%), and 20 weak motifs (2.5%). Within the subgroup of 468 TSSs associated with the two plasmids, 177 had strong RpoD motifs (37.8%), 33 medium motifs (7.1%), and 14 weak motifs (3%) (Table S1). These numbers were not different from those obtained for the overall TSS library (Table 3). An enrichment of RpoD promoters among horizontally acquired genes could not be seen on the overall TSS level. Testing the hypothesis needed closer examination. The relationship between RpoD- and not-RpoD-dependent promoters as well as between horizontally acquired and other genes may be more complicated than expected.

The RpoD scores for correctly and incorrectly positioned −35 and −10 motifs were not indicators for a strong transcription initiation event (see Fig. S3 in the supplemental material), indicating an influence of the upstream regulatory region, activators, and repressors on transcription initiation (46). The strong TSS score of TSSs not associated with RpoD (blue points in Fig. S4 in the supplemental material) were candidates for transcription initiation events using sigma factors other than RpoD in *C. metallidurans*. A plot of the NPKM value and TSS score, sorted into RpoD-dependent and non-RpoD-dependent promoters (shown for chromosomal genes in the “+” direction in Fig. S4, with all other genes not shown) also did not yield any insights. This analysis had to be done at a level with higher resolution. First, the interaction of RpoD- and non-RpoD-dependent promoters was investigated for the horizontally acquired metal resistance determinant *czc* on plasmid pMOL30. Second, all other metal resistance determinants in *C. metallidurans* were also examined. Last, as an example for a horizontally acquired genes not involved in metal resistance, genes required for chemolithoautotrophic growth of *C. metallidurans* were analyzed.

### Role of RpoD in expression of horizontally acquired genes.

For resistance determinants in *C. metallidurans*, TSSs were associated with the respective operons and sorted into those containing a RpoD promoter motif or not (see Fig. 1 for the first part of *czc*, and for all other determinants, see Fig. S5 in the supplemental material). TSSs with a weak score (<50) were only indicated if no other, stronger TSS was in the vicinity. Gene expression under nonchallenged conditions in TMM-grown cells was added as NPKM values (25) and supplemented with the response value for the respective genes (24). An NPKM value below 10 indicates a very low value or no expression, and an overall response of <5 indicates no up- or downregulation under conditions of metal stress or metal starvation in strain CH34 or its mutant strains.

**FIG 2:**
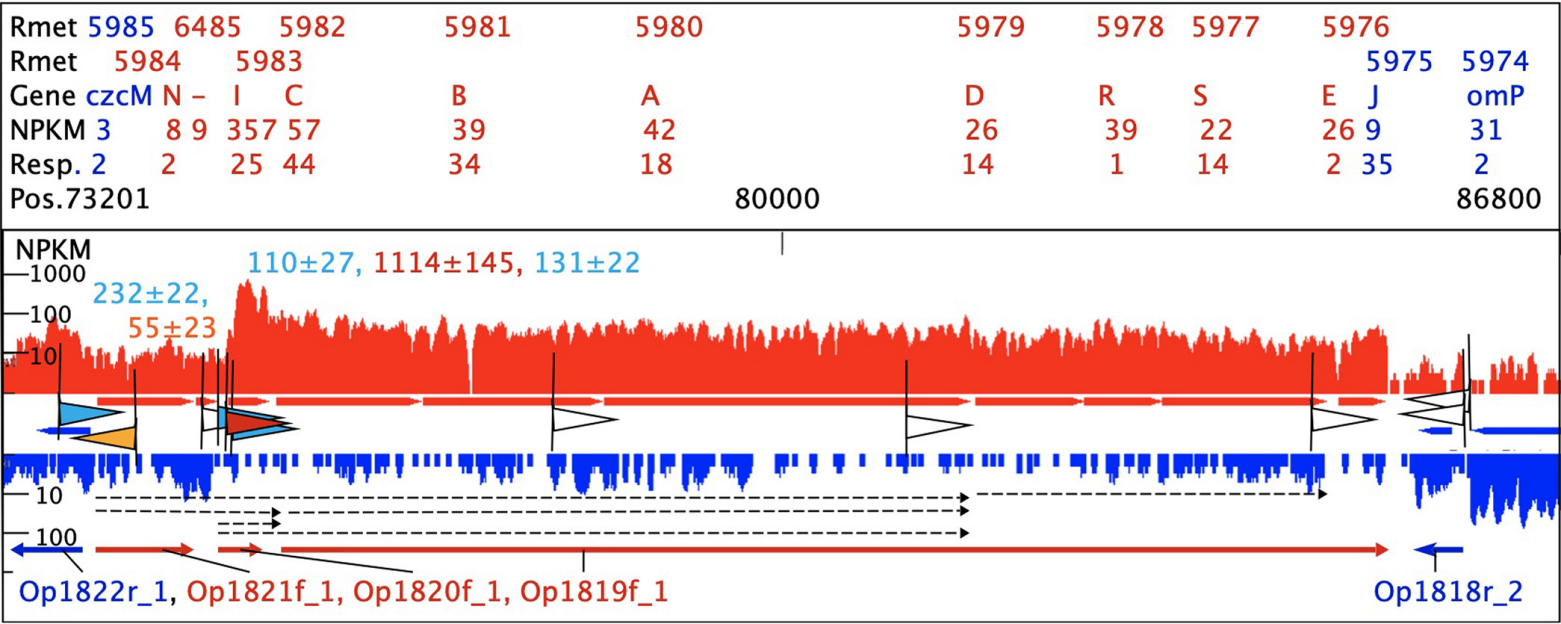
Map of the cobalt-zinc-cadmium resistance determinant *czc* on plasmid pMOL30. The map shows the first part of the *czc* determinants in the indicated regions with NPKM values on one DNA strand (red) or the other direction of transcription (blue). Above are the Rmet locus and gene names, the mean NPKM, and response values (24). TSSs (flags) are indicated with the corresponding TSS score as white with a score of <50, shades of red for strong (>1,000) or orange for medium (50 to 1000) transcription initiation from RpoD promoters (medium or strong RpoD score), and shades of blue if not associated or only weakly associated with the RpoD model. Transcripts (48) and operons (25) are also indicated in the transcript map. TSSs with scores of <50 (white arrows) are shown without TSS scores.

A weak TSS upstream of *czcM*, the first gene of the *czc* cobalt-zinc-cadmium resistance determinant on plasmid pMOL30 of *C. metallidurans*, could be associated with RpoD but not a medium-strong TSS 643 bp upstream of *czcN* (Fig. 2). Three more possible promoters 834, 844, and 1,395 bp upstream of *czcN* exhibited TSS scores of <50 (outside the window of Fig. 2): two of these were not RpoD dependent, but the promoter 1,395 bp upstream had a strong RpoD score. Due to the low TSS score, however, these promoters had no relevance for growth of *C. metallidurans* and *czc* expression under nonchallenging conditions.

Expression of *czc* increased within *czcI*, which was strongly expressed even in TMM-grown cells and contained two medium-strong promoters (TSS score of 100 to 1,000) and a strong promoter (TSS score of >1,000) upstream, all three of which sufficiently explained the transcript abundance of *czcI* and the subsequent genes. The strong TSS was RpoD dependent but displayed only a medium RpoD score (Data Set S1). It was probably responsible for the majority of the transcripts of *czcI* and *czcICBA* since only weak TSSs were found upstream of *czcA*, *czcD*, or *czcE* (Fig. 2). This strong RpoD-dependent TSS was located 53 bp upstream of *czcI* and had been identified previously in primer extension experiments in the region 52 ± 1 bp upstream of *czcI* (48), indicating that the accuracy of the determination of the TSS position was about ±1 bp, which was well within the *n_io_* clustering range of 5 bp. Since the binding site for the response regulator CzcR was directly upstream of *czcN* (48) but the medium-strong TSS was 643 bp upstream of the *czcN* open reading frame, a promoter adjacent to this binding site may not have been identified as TSS under nonchallenging conditions. Because the region between the binding site of CzcR and *czcI* contains a possible recognition motif of the integration host factor (IHF) (49) and *czcN* was not responding to metal stress and was expressed at a low level, CzcR may rather activate transcription initiation of the promoters upstream of *czcI*, relying on DNA bending by IHF.

The other two TSSs upstream of *czcI* (Fig. 2) could not be associated with RpoD, while a TSS with a score of only 22 ± 5 (white flag upstream of *czcI* in Fig. 2) displayed a strong RpoD score (Data Set S1). The *czcJ* gene (Fig. 1) and the *flgB-czcP* (Fig. S5A) operon further downstream from the main *czc* determinant were only weakly expressed in TMM-grown cells. The *czcJ* gene was under both RpoD control and non-RpoD control. The TSS of the *czcP* gene for the P_IB4_-type export system for “loosely” bound cytoplasmic zinc ions (50) could not be assigned to RpoD (Fig. S5A). Since the two-component response regulator CzcR controls gene expression from the promoters upstream of *czcN* and *czcP* under conditions of metal stress (48, 50–52) and the respective genes *czcN* and *czcP* were only weakly expressed in TMM-grown cells, the TSS responsible for CzcR-regulated expression of these genes may not have been identified.

An overview of the TSSs associated with the *czc* determinant on plasmid pMOL30 summarized 5 RpoD-dependent promoters in the sense direction and 4 in the antisense direction (Table 4). Additionally, nine non-RpoD-dependent promoters initiated *czc* transcription in the sense direction and 4 in the antisense direction. This indicated a complicated network controlling expression of *czc* and fine-tuning of its components. A detailed analysis of the TSSs involved in expression of all the other metal resistance determinants in *C. metallidurans* (Table 4; Fig. S5) also identified possible sense-antisense and RpoD/non-RpoD interactions for these other determinants, which with exception of *cup*, were horizontally acquired determinants.

**TABLE 4:** RpoD- and non-RpoD-dependent promoters and possible antisense transcripts involved in expression of metal resistance determinants in *C. metallidurans*^*a*^

RpoD-dependent promoters					Non-RpoD-dependent promoters				
TSS	TSS score	S	Putative transcript	Position	TSS	TSS score	S	Putative transcript	Position
*czc*									
**TSS_72644+4**	26 ± 10	+	*czcN*-anti.*czcM*	1,395 bp upstr. Rmet_5984	**TSS_73195+4**	17 ± 5	+	*czcN*-anti.*czcM*	844 bp upstr. Rmet_5984
*TSS_73647-4*	55 ± 23	−	*czcM*	482 bp upstr. Rmet_5985	**TSS_73205+4**	17 ± 4	+	*czcN*-anti.*czcM*	834 bp upstr. Rmet_5984
**TSS_74891+4**	22 ± 5	+	*czcICBADRSE*	284 bp upstr. Rmet_5983	**TSS_73396+4**	232 ± 22	+	*czcN*-anti.*czcM*	643 bp upstr. Rmet_5984
*TSS_75063-4*	29 ± 5	−	*czcM*-anti.*czcN*	1,103 bp upstr. Rmet_5985	**TSS_75078+4**	110 ± 27	+	*czcICBADRSE*	97 bp upstr. Rmet_5983
**TSS_75122+4**	1,114 ± 145	+	*czcICBADRSE*	53 bp upstr. Rmet_5983	**TSS_75144+4**	131 ± 22	+	*czcICBADRSE*	31 bp upstr. Rmet_5983
* TSS_75311-4*	50 ± 12	−	*czcM*-anti.*czcIN*	1,351 bp upstr. Rmet_5985	**TSS_78143+4**	32 ± 12	+	*czcADRSE*	308 bp upstr. Rmet_5980
* TSS_78567-4*	19 ± 2	−	*czcM*-anti.*czcAIN*	4,607 bp upstr. Rmet_5985	*TSS_79420-4*	17 ± 5	−	*czcM*-anti.*czcAIN*	5,460 bp upstr. Rmet_5985
* TSS_85892-4*	17 ± 5	−	*czcJ*	47 bp upstr. Rmet_5975	**TSS_80690+4**	36 ± 4	+	*czcDRSE*	1,003 bp upstr. Rmet_5979
**TSS_88154+4**	15 ± 2	+	*flgB*	528 bp upstr. Rmet_5971	**TSS_84829+4**	26 ± 5	+	*czcE*	37 bp upstr. Rmet_5976
					*TSS_85937-4*	23 ± 4	−	*czcJ*	92 bp upstr. Rmet_5975
					*TSS_87294-4*	89 ± 26	−	*ompP*-*czcJ*	199 bp upstr. Rmet_5974
					*TSS_88410-4*	160 ± 35	−	*tnpAB-ompP-czcJ*	12 bp upstr. Rmet_5972
					**TSS_89563+4**	24 ± 6	+	*czcP*	41 bp upstr. Rmet_5970

*cnr*									
** TSS_53286+5**	204 ± 31	+	*cnrYXHCABT*	23 bp upstr. Rmet_6205	**TSS_52343+5**	80 ± 14	+	*cnrYXHCABT*	966 bp upstr. Rmet_6205
** TSS_56132+5**	47 ± 8	+	*cnrAT*	989 bp upstr. Rmet_6210	**TSS_54660+5**	87 ± 17	+	*cnrCBAT*	24 bp upstr. Rmet_6208
** TSS_57815+5**	32 ± 9	+	*cnrT*	2,582 bp upstr. Rmet_6211	**TSS_59634+5**	164 ± 16	+	*cnrT*	763 bp upstr. Rmet_6211

*czc2*									
*TSS_1215464-3*	743 ± 13	−	*zntA*	12 bp upstr. Rmet_4594					
**TSS_1215587+3**	155 ± 52	+	*czcC2B2*′	474 bp upstr. Rmet_4596					
* TSS_1221261-3*	317 ± 65	−	*tnpRA*-anti.*czcB2*′	25 bp upstr. Rmet_4599					

*czc2* part 2									
** TSS_1061603+3**	27 ± 5	+	*czcR2S2*-*ubiG*-anti.*czcA2B2*′′-*uspA11*-*sulP*	55 bp upstr. Rmet_4465	**TSS_1061540+3**	41 ± 9	+	*czcR2S2-ubiG*-anti-*czcA2B2*′′-*uspA11*-*sulP*	118 bp upstr. Rmet_4465
*TSS_1071434-3*	12,866 ± 1,637	−	*sulP*-*uspA11*-*czcB2*′′*A2*-anti.*ubiG*-*czcS2R2*	231 bp upstr. Rmet_4472	**TSS_1063787+3**	159 ± 30	+	*ubiG*-anti-*czcA2B2*-*uspA11-sulP*	8 bp upstr. Rmet_4467

*zni*/*zne*									
*TSS_2029923-3*	2,807 ± 1,154	−	*zniBA*	28 bp upstr. Rmet_5320	*TSS_2033447-3*	22 ± 3	−	*zniS*-anti-*zniR*	247 bp upstr. Rmet_5322
**TSS_2030010+3**	530 ± 145	+	*zniC*-anti.*zniS*	54 bp upstr. Rmet_5321	*TSS_2034321-3*	66 ± 35	−	*zniS*-anti-*zniR*	72 bp upstr. Rmet_5324
*TSS_2033229-3*	30 ± 6	−	*zniS*-anti.*zniC*	29 bp upstr. Rmet_5322	**TSS_2035339+3**	45 ± 9	+	*zneRS*-anti-*zneCAB*	27 bp upstr. Rmet_5326
**TSS_2033306+3**	278 ± 19	+	*zniR*	3 bp upstr. Rmet_5323	*TSS_2040956-3*	19 ± 2	−	*zneC*-anti-*zneSR*	1,986 bp upstr. Rmet_5328
**TSS_2033338+3**	16 ± 3	+	*zneP*	1,215 bp upstr. Rmet_5325	**TSS_2043398+3**	122 ± 52	+	*zneR2S2*	18 bp upstr. Rmet_5331
* TSS_2033649-3*	142 ± 16	−	*zniS*-anti.*zniR*	449 bp upstr. Rmet_5322	*TSS_2043482-3*	28 ± 11	−	*zneBAC*-anti-*zneR2*	243 bp upstr. Rmet_5330
* TSS_2043295-3*	189 ± 76	−	*zneBAC*-anti.*zneRS*	56 bp upstr. Rmet_5330					
**TSS_2043372+3**	36 ± 4	+	*zneR2S2*	44 bp upstr. Rmet_5331					

*ncc*					**TSS_154117+4**	29 ± 8	+	*nreB*	850 bp upstr. Rmet_6144

*hmv*									
** TSS_377775+3**	495 ± 50	+	*hmvCBA*′	181 bp upstr. Rmet_3836					
** TSS_377885+3**	60 ± 32	+	*hmvCBA*′	71 bp upstr. Rmet_3836					

*hmy*					**TSS_689178+3**	375 ± 63	+	*hmyFCB*	111 bp upstr. Rmet_4119
					**TSS_689199+3**	74 ± 6	+	*hmyFCB*	90 bp upstr. Rmet_4119

*nim*									
**TSS_2431185+3**	25 ± 6	+	*copR2S2*-anti.*nimC*	147 bp upstr. Rmet_5672	**TSS_2431257+3**	35 ± 6	+	*copR2S2*-anti.*nimC*	75 bp upstr. Rmet_5672
					**TSS_2431286+3**	58 ± 11	+	*copR2S2*-anti.*nimC*	46 bp upstr. Rmet_5672
					*TSS_2437189-3*	94 ± 22	−	*nimA1*′*C*-anti.*copS2R2*	94 bp upstr. Rmet_5678
					*TSS_2438305-3*	168 ± 42	−	*tnpAB*-*nimA1*′*C*-anti.*copS2R2*	12 bp upstr. Rmet_5680
					*TSS_2442033-3*	1,025 ± 113	−	*tnpAB-nimA1*′*C*-anti.*copS2R2*	0 bp upstr. Rmet_5683

*hmz*									
** TSS_3271982+2**	81 ± 16	+	*yodB*-anti.*hmzSR*	74 bp upstr. Rmet_3013	**TSS_3271933+2**	70 ± 15	+	*yodB*-anti.*hmzSR*	123 bp upstr. Rmet_3013
** TSS_3272142+2**	16 ± 4	+	anti.*hmzSR*	507 bp upstr. Rmet_3014	*TSS_3276923-2*	50 ± 9	−	*hmzRS*-anti.*yodB*	113 bp upstr. Rmet_3017
* TSS_3275756-2*	55 ± 5	−	*hmzRS*-anti-*yodB*	304 bp upstr. Rmet_3016					
* TSS_3276729-2*	22 ± 3	−	*hmzRS*-anti-*yodB*	1,277 bp upstr. Rmet_3016					

*cus*									
**TSS_1715990+3**	2,235 ± 160	+	*cusDCBAF*	20 bp upstr. Rmet_5030	*TSS_1723457-3*	1,233 ± 204	−	anti.*cusDCBAF*	24 bp upstr. Rmet_5036
*TSS_1724117-3*	284 ± 48	−	anti.*cusDCBAF*	30 bp upstr. Rmet_5037					

*sil*									
*TSS_170434-4*	133 ± 45	−	*silDCBA*	16 bp upstr. Rmet_6133	*TSS_170449-4*	51 ± 14	−	*silDCBA*	31 bp upstr. Rmet_6133

*cad*									
*TSS_2523175-2*	1,256 ± 163	−	*cadR*	43 bp upstr. Rmet_2302	*TSS_2526632-2*	29 ± 2	−	anti.*cadCA*	3,500 bp upstr. Rmet_2302
**TSS_2523190+2**	58 ± 21	+	*cadAC*	2,949 bp upstr. Rmet_2304					

*pbr*									
**TSS_111789+4**	21 ± 2	+	*pbrR*	2,793 bp upstr. Rmet_5946	*TSS_111006-4*	36 ± 10	−	*pbrD*	150 bp upstr. Rmet_5949
* TSS_112599-4*	21 ± 3	−	*pbrB*/*C-D*	499 bp upstr. Rmet_5948	*TSS_116694-4*	33 ± 10	−	*pbrA*-anti.TR	2,198 bp upstr. Rmet_5947
* TSS_114524-4*	27 ± 6	−	*pbrA-B*/*C-D*	28 bp upstr. Rmet_5947	*TSS_117004-4*	47 ± 10	−	*pbrA*-anti.TR	2,508 bp upstr. Rmet_5947
** TSS_114561+4**	215 ± 13	+	*pbrRTUa*	21 bp upstr. Rmet_5946	*TSS_117403-4*	25 ± 2	−	*pbrA*-anti.*pbrUaTE*	2,907 bp upstr. Rmet_5947
** TSS_115102+4**	273 ± 29	+	*pbrTUa*	39 bp upstr. Rmet_5945					
** TSS_117222+4**	39 ± 6	+	*pbrUa*	35 bp upstr. Rmet_5944					

*cop*									
** TSS_180770+4**	25 ± 6	+	*copO*-anti.*copL*	1,441 bp upstr. Rmet_6382	*TSS_181983-4*	45 ± 3	−	*copL*-anti.*copQH*	14 bp upstr. Rmet_6120
** TSS_182294+4**	21 ± 6	+	*copFGJ*	101 bp upstr. Rmet_6119	*TSS_191327-4*	26 ± 8	−	*copA1B1C1D1I*-anti.*copJGFO*	128 bp upstr. Rmet_6112
* TSS_184844-4*	148 ± 32	−	*copL*-anti-*copFO*	2,875 bp upstr. Rmet_6120	**TSS_191346+4**	28 ± 9	+	*copR1S1N*	56 bp upstr. Rmet_6111
* TSS_185929-4*	190 ± 22	−	*copL*-anti.*copJGFO*	3,960 bp upstr. Rmet_6120	TSS_191954-4	51 ± 12	−	*copA1B1C1D1I*-anti.*copJGFO*	755 bp upstr. Rmet_6112
* TSS_191580-4*	21 ± 3	−	*copA1B1C1D1I*-anti.*copJGFO*	381 bp upstr. Rmet_6112	**TSS_192980+4**	19 ± 2	+	*copN*	547 bp upstr. Rmet_6109
** TSS_193249+4**	35 ± 12	+	*copN*	278 bp upstr. Rmet_6109	**TSS_195510+4**	20 ± 4	+	*copT*	127 bp upstr. Rmet_6106
** TSS_195615+4**	69 ± 12	+	*copT*	22 bp upstr. Rmet_6106					
*TSS_197125-4*	185 ± 14	−	*copM*-anti.*copVT*	2,354 bp upstr. Rmet_6107					

*cop2*									
**TSS_2431185+3**	25 ± 6	+	*copR2S2*	147 bp upstr. Rmet_5672	**TSS_2426368+3**	32 ± 8	+	anti.*copD2C2B2A2*	118 bp upstr. Rmet_5667
					**TSS_2431257+3**	35 ± 6	+	*copR2S2*	75 bp upstr. Rmet_5672
					**TSS_2431286+3**	58 ± 11	+	*copR2S2*	46 bp upstr. Rmet_5672

*cup*					*TSS_3818027-2*	82 ± 13	−	*cupAR*	39 bp upstr. Rmet_3524
					*TSS_3819200-2*	1,312 ± 146	−	*ompW1*-anti.*cupC*	39 bp upstr. Rmet_3526
					*TSS_3819255-2*	26 ± 7	−	*ompW1*-anti.*cupC*	94 bp upstr. Rmet_3526

*chr*									
*TSS_52146-5*	149 ± 61	−	*chrB1*	0 bp upstr. Rmet_6203	**TSS_46003+5**	15 ± 2	+	*chrI*-anti.*chrYPNOF1ECA1B1*	6,297 bp upstr. Rmet_6204
					**TSS_48568+5**	300 ± 44	+	*chrI*-anti.*chrF1ECA1B1*	3,732 bp upstr. Rmet_6204
					**TSS_49695+5**	16 ± 1	+	*chrI*-anti.*chrCA1B1*	2,605 bp upstr. Rmet_6204
					**TSS_50747+5**	61 ± 16	+	*chrI*-anti.*chrA1B1*	1,553 bp upstr. Rmet_6204

*chr2*									
*TSS_410991-3*	60 ± 3	−	*chrB2A2F2*	0 bp upstr. Rmet_3867	*TSS_408671-3*	36 ± 11	−	*chrF2*	931 bp upstr. Rmet_3864

*Ars*									
*TSS_351119-2*	164 ± 57	−	*arsR*	0 bp upstr. Rmet_0333					

a Transcriptional start sites (TSSs) are listed in the vicinity of metal resistance genes with their TSS score and DNA strand (S). TSSs in boldface are on the “+” strand, and those in italic are on the “−” strand. The putative transcript is given with fields shaded gray representing antisense transcripts. The position of the TSS with respect to a gene (Rmet sequence tag) is also provided. All TSSs were sorted into the group of RpoD- and non-RpoD-dependent promoters as outlined in the text. upstr., upstream; anti., antisense.

A large operon encoding a membrane-bound hydrogenase was transcribed from a non-RpoD-dependent promoter, and a possible non-RpoD-dependent promoter may also initiate transcription in the antisense direction (see Fig. S6 in the supplemental material). In contrast, a large horizontally acquired region encoding a soluble, NAD-reducing hydrogenase and the enzymes of the Calvin cycle was mainly under RpoD control. These regions were not connected to metal resistance but sense/antisense and RpoD/non-RpoD interactions were also involved in control of expression of these genes.

In summary, RpoD-dependent promoters were indeed important for expression of the horizontally acquired genes involved and not involved in metal resistance in *C. metallidurans*. On the other hand, sense/antisense transcriptional events plus non-RpoD sigma factors were also important—probably to mold the regulation of expression of these genes into the regulatory network of the new host *C. metallidurans*.

## DISCUSSION

### RpoD is central to control of the expression of active metal resistance determinants.

Resistance to the divalent cations of transition metal cations, chromate, or arsenate as specific stress systems can be mediated by determinants that are often found on transmittable replicons such as plasmids, plasmid-derived chromids, or transposons (20, 53). Resistance mechanisms include covalent modification, reduction to a less harmful oxidation state, or efflux, which can be across the cytoplasmic membrane into the periplasm of Gram-negative bacteria (e.g., by P-type ATPases) or from the periplasm to the outside by transenvelope efflux systems (54–56).

*C. metallidurans* contains 12 resistance determinants harboring genes for transenvelope efflux systems, 1 on plasmid pMOL28 (*cnr*), 3 on plasmid pMOL30 (*czc*, *ncc*, *sil*), 7 on the chromid (*czc2* or *hmu*, *zni*, *zne*, *hmv*, *hmy*, *nim*, *cus*), and 1 on the chromosome (*hmz*). Two of these systems mediate resistance to monovalent transition metal cations such as Cu(I) and Ag(I), and the other 10 mediate resistance to divalent transition metal cations such as Zn(II) and Ni(II) (15–18, 57–63). Among these 10, the plasmid-bound *czc* and *cnr* determinants are dominant over the eight remaining determinants (24).

Transenvelope efflux complexes are composed of an outer membrane factor (OMF) of the OMF protein family (64–67), a membrane fusion protein (MFP) or periplasmic adapter protein of the MFP family (68, 69), and a heavy metal-exporting (HME) protein of the resistance nodulation-cell division (RND) protein superfamily (70–72). In addition to the transenvelope systems, *C. metallidurans* contains 8 genes for transition metal cation-exporting P_IB_-type ATPases, three P_IB2_-type ATPases (*zntA*, *cadA*, *pbrA*) and one P_IB4_-type ATPases (*czcP*) for divalent metal cations, and two P_IB1_-type ATPases for export (*cupA*, *copG*) of surplus cytoplasmic Cu(I) plus two (*ctpA1*, *rdxI*) for delivery of this ion to copper-containing enzymes with active sites in the periplasm (15, 16, 50, 73). These genes are part of large metal resistance determinants on plasmid pMOL30 (*czcP*/*czc*, *pbrA*/*pbr*, *copG*/*cop*), plasmid pMOL28 (*cnr*), on the chromid (*zntA*/*czc2*), on the chromosome (*cadA*/*cad*, *cupA*/*cup*), or on the chromosome as part of large regions encoding proteins of the respiratory chain (*ctpA1*, *rdxI*). Chromate resistance determinants are located on plasmid pMOL28 (*chr*) and the chromide (*chr2*), an arsenate resistance determinant (*ars*) on the bacterial chromosome (74), mercury resistance determinants (*mer*) on the chromosome, and both plasmids (75, 76).

The *czc* determinant on plasmid pMOL30 contains several operons predicted from RNA-Seq experiments (Fig. 2): Op1822r_1 with *czcM* (alternative name *mgtC*), Op1821f_1 with *czcN*, Op1820f_1 with *czcI*, Op1819f_1 with *czcCBADRSE*, Op1818r_2 with *czcJ*, and Op1817f_1 with *flgB-czcP* (Fig. S5A) (25). CzcCBA is the RND-driven transenvelope efflux system for cobalt, zinc, and cadmium ions, CzcRS, a two-component regulatory system, CzcD, an inner membrane efflux pump and one of the founders of the CDF protein family, CzcE, a periplasmic protein involved in regulation of *czc*, CzcP, a P_IB4_-type exporter of loosely bound cytoplasmic zinc ions, CzcJ and CzcI, additional periplasmic proteins, and CzcN and FlgB, which are of unknown functions (16, 50). CzcI functions as a “quencher” of Czc activity to avoid overpumping of essential transition metal cations, mainly cobalt (77). MgtC-like proteins such as CzcM inhibit phosphate uptake under conditions of magnesium starvation (78).

Transcripts after metal induction had been identified by Northern RNA-DNA hybridization and reverse transcription-PCR (RT-PCR) experiments for the regions *czcNICBA*, *czcNI*, *czcI*, *czcICBA*, *czcCBA*, and *czcDRS* (48), indicating some read-through within the operons in forward orientation Op1821f_1 to Op1819f_1 (Fig. 2). The *czcM* and *czcN* genes, transcribed into the opposite direction from each other from a binding site of the response regulator CzcR (48), were nearly not expressed in TMM-grown cells (NPKM < 10) (Fig. 1) and also did not respond to metal stress.

There was considerable expression of *czcICBADRSE* even in cells not challenged by metals (Fig. 2). This indicated that the Czc system was also needed for metal homeostasis at low zinc concentrations. Since only very weak promoter motifs were found within this region, transcription from the three *czcIp* promoters may yield different transcripts by cleavage or termination events downstream of *czcI* and of *czcA*, where a terminator stem-loop exists (52), and downstream of *czcS*. While two of these TSSs could not be assigned to RpoD, the strongest TSS upstream of *czcI* was clearly an RpoD-dependent promoter. Under metal stress, additional CzcR-dependent transcription should activate the *czcNp* and *czcPp* promoters, which were no RpoD promoters or had not been identified yet. Thus, the dominant *czc* determinant was under RpoD control but other sigma factors and CzcR may contribute to its expression, especially under conditions of metal stress.

A second, *czc*-related *czc2* or *hmu* determinant resides on the chromid and is also related to *zntA↔czcICBA←czcRS* determinants in other bacteria (57). The region in *C. metallidurans* was divided into halves by transposon insertion into *czcB2*, and both halves were also translocated to different regions of the chromid (Fig. S5B and C). Expression of *zntA* for the main zinc-exporting P_IB2_-type ATPase of *C. metallidurans* (50, 79) was under the control of RpoD, the ZntR regulatory protein (77). RpoD was clearly responsible for expression of most genes of the *czc2* determinant, including *czcI2C2B2*′. The two-component regulatory system CzcR2S2 may be still active, partly under RpoD control, and interfere with the RpoD-dependent expression of *czcI2C2*, which may contribute to metal homeostasis in *C. metallidurans* (24). CzcC2 may have interacted with the CzcCBA complex to modify the substrate range of the transenvelope efflux system (24, 80). The possible contribution of CzcC2 and CzcI2 to fine-tuning of the plasmid pMOL30-encoded Czc system plus the vicinity to *zntA* may explain why this damaged *czc2* determinant had not been deleted during evolution of *C. metallidurans* CH34.

Transcription of the *cnrYXHCBAT* determinant on plasmid pMOL28 encodes the CnrCBA transenvelope efflux system, the CnrT inner membrane exporter, the sigma factor CnrH and the corresponding membrane-bound anti-sigma factor complex CnrYX. The position of the *cnrYp* and *cnrCp* had been previously determined by primer extension at positions 24 and 25 upstream of the respective gene (81), again clearly indicating that the accuracy of the TSS determination was ±1 bp. The strong RpoD-dependent promoter upstream of *cnrY* (Fig. S5D) explains expression of *cnr* in the absence of CnrH (25). The non-RpoD-dependent *cnrYp* and *cnrCp* promoters agreed to the fact that nickel-induced *cnr* expression was under the control of the sigma factor CnrH (81–86). The expression level of *cnrYXHCBAT* in *C. metallidurans* cells grown in TMM decreased from NPKM = 50 to NPKM = 17 (Fig. S5D; deviations in Data Set S1). Since half-maximal activation of *cnr* occurs at a nickel concentration of 50 μM (81) and the nickel concentration in TMM is below 1 μM due to the added trace element solution (14), *cnr* should not be transcribed under responsibility of the sigma factor CnrH in nonamended TMM. Consequently, the observed expression level of *cnr* (Fig. S5D) derives from the activity of the RpoD-dependent *cnrYp* promoter, which exhibited a sufficient TSS score. This guarantees that the CnrH/CnrYX regulatory proteins and the CnrCBA transenvelope efflux complex are ready to fight off a sudden increase of the nickel concentration and able to produce a system with more efflux by expression of *cnrCBAT* from the CnrH-dependent *cnrYp* and *cnrCp* promoters. Nevertheless, the baseline of expression of *cnr* was under RpoD control, so that RpoD was also responsible for the second dominant determinant for a transenvelope efflux system. It is interesting to note that *cnr* was not regulated by a two-component regulatory system but rather by a ECF sigma factor. This situation may be required to avoid cross talk of regulators involved in nickel, copper, and zinc homeostasis, respectively.

The *zni-zne* double resistance determinant on the chromid is on a low expression level and might be associated with zinc resistance (59) but is among the recessive metal resistance determinants in *C. metallidurans* (24). It encodes two putative transenvelope efflux systems, ZniCBA and ZneCBA, three two-component regulatory systems, ZniRS, ZneRS, and ZneR2S2, and a putative periplasmic protein, ZneP (Fig. S5E). The expression levels in nonchallenged cells in combination with the responsiveness to metal stress conditions identified the Zni system as a minor transenvelope efflux system, which may contribute to metal homeostasis and is under RpoD and ZniRS control. The operon *zneBAC* (Op1577r_1) was also expressed and under RpoD control (Fig. S5E and F). As with *czc* and *czc2*, which were not associated with an own sigma factor such as CnrH, *zni* and *zne* were under RpoD control, but other sigma factors may be involved in expression of *zneRS* and *zneR2S2*, which may cross talk to other two-component regulatory system for fine control of metal homeostasis.

Among the inactivated determinants encoding transenvelope efflux systems, the *ncc* determinant on plasmid pMOL30, which is related to *cnr* and in other bacteria is under the control of the sigma factor NccH (87), was nearly not expressed in nonchallenged cells and the *nccB* gene is inactivated by a frameshift mutation (Fig. S5G). No TSS could be identified upstream of this inactivated metal resistance determinant, which agreed with the absence of the *nccH* gene for an *ncc*-specific sigma factor in *C. metallidurans* (Fig. S5G). Operon Op1066f_2 on the chromid with *hmvCBA*′/*A*′′ has the *hmvA* gene interrupted by a frameshift mutation (Fig. S5H) and was under RpoD control. The genes *hmvCB* were responding to metal stress and may also interact with CzcCBA (24, 80). The *hmyFCB* genes were responding to metal stress, under non-RpoD control, and may interfere with CzcCBA or other transenvelope efflux systems (24, 80). The *nim* genes, interrupted by a transposon insertion into the *nimA* gene, were expressed but could not be associated with RpoD (Fig. S5J). The *hmz* determinant was located on the chromosomal island, again with a transposon adjacent (Fig. S5K). While *hmzBA* were nearly not expressed and no TSS could be identified, the regulatory genes *hmzRS* were expressed from an RpoD-dependent TSS. Together, *hmvCB* and *hmzRS* were expressed, responding to, and under RpoD control, and *hmyFCB* and *nimC* were expressed, responding to, and under non-RpoD control.

RpoD was strongly involved in expression of the dominant metal resistance determinants *czc* and *cnr*, despite the fact that *cnr* possesses its own sigma factor, and were involved to some degree in expression of *zni*/*zne*; however, other sigma factors may be required to express parts of the recessive determinants. The various two-component regulatory systems from *czc* and the recessive determinants may be involved in a fine-tuning of metal homeostasis (24) and other sigma factors could be involved in this process, especially to produce NimCB and HmyFCB as additional interaction partners of CzcA, CnrA, CusA, or SilA. The close proximity of *cop2* and *nim* on the chromid indicates a possible interaction of NimBC with CusA or SilA.

Taken together, transenvelope efflux systems allow *C. metallidurans* its outstanding metal resistance by adjusting the periplasmic zinc, cobalt, cadmium, and nickel concentrations as the first line of metal defense. The Czc and the Cnr systems are the dominant determinants for cobalt, zinc, and cadmium and for cobalt and nickel resistance, respectively (24). Fine-tuning of expression of the multitude of *czc* genes is done by interaction of RpoD and non-RpoD-dependent promoters and sense and antisense transcription (Table 4), and that of *cnr* genes is done by interaction of sigma factors CnrH and RpoD. A similar network of RpoD/non-RpoD sense/antisense also seems to control the minor *zni*/*zne* determinant. Other determinants have an interrupted gene for the central RND efflux pump but may nevertheless contribute outer membrane factors, membrane fusion proteins, periplasmic proteins, and two-component regulatory systems to the cell (24). These components may enhance the ability of *C. metallidurans* to handle divalent transition metals in a broad range of concentrations and mixtures. Again, RpoD/non-RpoD and sense/antisense interaction may regulate expression of the genes for these additional components (Table 4).

### Copper resistance determinants.

Two transenvelope efflux systems are involved in copper resistance: *cus* and *sil*. The *cusDCBA* determinant operon Op1480f_2 on the chromid was not expressed in nonchallenged cells, with the exception of the RpoD-controlled *cusD* gene at the 5′ end, which encodes an uncharacterized protein (Fig. S5L). Transcription from two non-RpoD-dependent promoters downstream of the *cus* operon on the other DNA strand may yield an antisense transcript that destabilizes the *cusDCBAF* transcript. There are no genes for a two-component regulatory system part of *cus*, in contrast to E. coli (88, 89), one of the CopRS systems may be required for the clear response of the *cus* determinant to metal stress or the antisense transcripts are downregulated under these conditions. The *silDCBA* genes on plasmid pMOL30 (Fig. S5M) were not responding to metal stress, but silver was not part of the challenging metals (24). The *cus* and *sil* determinants for copper-exporting transenvelope efflux systems were RpoD dependent, with additional non-RpoD sigma factors possibly contributing to *sil* expression and to *cus* antisense repression.

The *cop*, *cop2*, and *cup* determinants are involved in copper resistance in addition to *cus* and *sil*. The huge multigene copper resistance region *cop* on plasmid pMOL30, organized in nine operons (Fig. S6P and Q), was under the control of RpoD and at least one other sigma factor, probably of the two-component regulatory system CopR1S1. Control of *copR1S1* was by a non-RpoD sigma factor. A possible antisense action to expression of *copF* for a copper-exporting P_IB1_-type ATPAse in *C. metallidurans* in addition to CupA was under RpoD control.

The *cup* determinant for the P_IB1_-type ATPase CupA is located on the chromosome outside any chromosomal island (Fig. S5R). Expression of *cupAR* is probably regulated by CupR (90) and under non-RpoD control. The *cop2* determinant around the CopA2 periplasmic Cu(I) oxidase is on the chromid (Fig. S5S) and organized in the two divergently oriented operons. While Op1695r_1/2 with *copA2B2C2D2* was nearly not expressed, the genes *copR2S2* may be expressed by RpoD and at least one other sigma factor. While expression of *cop2* was at a very low level, expression of *cup* was between NPKM values of 26 for *cupA* and 84 for the gene *cupR* for a MerR-type regulator. In TMM under nonchallenging conditions, copper resistance in *C. metallidurans* was based on export of Cu(I) from the cytoplasm by CupA, while the other systems were only needed at high copper concentrations.

Overall, regulation of copper homeostasis by *cus*, *sil*, *cop2*, *cop*, and *cup* (and maybe also *nim*) seems to involve RpoD, at least one other sigma factor, two two-component regulatory systems, and possibly also some antisense effects (Table 4).

### Control of other metal resistance determinants by RpoD.

The *cad* cadmium, *pbr* lead, *chr* and *chr2* chromate, *ars* arsenate, and *mer* mercury resistance determinants were also investigated with respect to involvement of RpoD-dependent promoters (Fig. S5N to Z). The chromosomal *cad* determinant, composed of the *cadR* gene for the MerR-type regulator (77) in one direction of transcription and *cadAC* for the CadA P_IB2_-type ATPase and protein CadC, started from two RpoD-dependent TSSs in a common promoter region (Fig. S5N). Lead resistance, encoded by *pbr* on plasmid pMOL30, was under RpoD control (Fig. S4O). The MerR-type regulator PbrR and the possible lead uptake protein PbrT may be present in the cells to watch out for lead ions so that the *pbr* determinant can be activated when needed (19, 91–93). The *chr* chromate resistance determinant on plasmid pMOL28 was organized as a decacistronic operon around the *chrA1* gene for a chromate efflux pump (74, 94, 95) and is clearly under RpoD control (Fig. S5T). Expression of a second *chr2* resistance determinant Op1075r_2 on the chromid was also from an TSS with a RpoD motif upstream (Fig. S5U). Expression of the *arsRIC2BC1HP* arsenate resistance determinant Op0094r_2/3 on the chromosomal island CMGI-7 was RpoD dependent, but not that of the *arsM* gene for an arsenate methyltransferase upstream of *arsR* (Fig. S5V).

Again, while most genes were under RpoD control, genes that may enlarge the capability of arsenate resistance in *C. metallidurans* (for instance, by allowing arsenate methylation) were under non-RpoD control. Among the *mer* determinants for mercury resistance, the *merP* gene was under RpoD control (Fig. S5Y and Z).

### Control of horizontally acquired determinants not involved in metal resistance: expression of hydrogenase and Calvin cycle genes.

*C. metallidurans* contains two large clusters of genes, which enable the bacterium to grow facultatively as a chemolithoautotroph with molecular hydrogen and oxygen as energy sources, used to assimilate carbon dioxide via the Calvin cycle. These two clusters are part of two different genomic islands on the bacterial chromosome (21–23) and can be lost easily in *C. metallidurans* mutants (24). The genes for synthesis of a membrane-bound hydrogenase are all in one large multicistronic operon, Op0370r_1, and transcribed from a non-RpoD-dependent promoter (Fig. S6A and B). The genes for the Calvin cycle enzymes and a soluble, NADH-reducing hydrogenase were arranged in six operon regions, Op0422r to Op0427f (Fig. S6C to H), and heavily affected by transposon insertions and rearrangements. In contrast to the large operon that encodes the membrane-bound hydrogenase, these six operons were under RpoD control. In the related bacterium *C. eutrophus*, the hydrogenase determinants are under RpoN control (96), but the ATP-hydrolyzing activator HoxA, which is required for the formation of the open complex of the RpoN-RNAP, is truncated in *C. metallidurans* (23). An RpoN-dependent transcription initiation of the hydrogenase genes should not be possible in this bacterium. In case of the soluble hydrogenase and the Calvin cycle enzymes, RpoD-dependent promoters seem to have evolved after transfer of this genomic island into *C. metallidurans*, and also after transposon insertion into the *pntAA* gene, while another sigma factor took over expression of the operon for the membrane-bound hydrogenase. This indicated that horizontally transmitted genes may not obligately require an active promoter when arriving in a new host but that new promoters may originate by adaptive evolution.

### Conclusion.

The outstanding features of *C. metallidurans* CH34 are its high metal resistance and its facultative ability to grow as an aerobic hydrogen-oxidizing chemolithoautotrophic bacterium. These central traits were predominantly acquired by horizontal gene transfer, as indicated by the localization of the respective determinants on CMGIs: either of the two plasmids or the chromid. Most of the determinants were indeed transcribed from TSSs with RpoD consensus motifs upstream, with the exceptions of the non-island-located chromosomal *cup* determinant and operon Op0370r_1 for the membrane-bound hydrogenase. On the one hand, this indicated the importance of RpoD-dependent promoters, which allow a rapid initial expression and subsequent benefit of a determinant just acquired by horizontal gene transfer. On the other hand, that most of the active determinants involved as well as those not involved in metal resistance also contained non-RpoD-dependent promoters and displayed antisense transcription (Table 4) hints at differential RNA stability and leaderless mRNA (lmRNA) initiation (97). These regulatory mechanisms may be required to mold regulation of expression of an assimilated gene or determinant into the regulatory network of the new host cell, which should increase the benefit of the new gene even more in a second adaptation step. Concerning the central hypothesis of this publication: Yes, RpoD-dependent promoters are indeed widespread in horizontally acquired genetic elements. But this is only one side to the coin. Non-RpoD-dependent promoters are of similar importance and may be needed to assimilate a gene into the genome by molding its expression into the regulatory network of the host cell.

## MATERIALS AND METHODS

### Bacterial strains and growth conditions.

Only wild-type strain *C. metallidurans* CH34 (14) was used, as well as Tris-buffered mineral salts medium (14) containing 2 g sodium gluconate/L (TMM). The bacterium was cultivated aerobically with shaking at 30°C. Solid Tris-buffered medium contained 20 g agar/L.

### RNA isolation.

At a cell turbidity of 120 Klett units, the cells were rapidly harvested at room temperature and stored at −80°C. Total RNA was isolated with the RNeasy Plus minikit (Qiagen, Hilden, Germany) according to the manufacturer’s instruction. One DNase treatment was performed. To exclude experimental artifacts resulting from DNA contaminations, only RNA was used that did not generate products in several PCRs with chromosomal and plasmid primers. The RNA concentration was determined photometrically, and RNA quality was checked on formamide gels (98) and measured as an RNA integrity number (RIN) on an Agilent 2100 Bioanalyzer (Agilent Technologies, Waldbronn, Germany).

### TSS determination.

RNA was prepared from *C. metallidurans* CH34 cells cultivated in TMM (Tris-buffered mineral salts medium with 2 g/L gluconate as the carbon source) for three independent biological repeats. RNA-Seq was performed by Vertis Biotechnology AG (Freising, Germany) using a Cappable-seq protocol for TSS determination (28). The TSSs were trimmed and mapped to the reference genomes CP000352 (chromosome), CP000353 (chromid, also named “megaplasmid”), CP000354 (plasmid pMOL30), and CP000355 (pMOL28), and potential TSSs were annotated as peaks using program tools made available by Laurence Ettwiller (New England Biolabs; https://github.com/Ettwiller/TSS). Since the number of control reads was small compared to the TSS reads, TSSs were calculated without using the control reads. The *n_io_* value was the number of reads at position *i* in orientation *o*, and *N* was the total number of mapped reads. The RRS*_io_* value for each position and orientation was the reads per million and was defined as RRS*_io_* = PRM = (*n_io_*/*N*) × 10^6^ for TSS determination and control. For each TSS, the score was RRS*_io_*_TSS/RRS*_io_*_control. For the TSS determination, a cutoff value of RRS*_io_* = 5 and a cluster value of 5 were used, the latter defining the size in bp of the upstream and downstream region used for clustering conditions. Only TSSs were further considered that appeared in all three biological repeats and had a score of >10.

### Extraction of promoter sequences.

Promoter sequences per TSS were extracted as sequence regions −90 to +10 bp around the TSS position on the respective replicon (GenBank accession no. CP000352.1, CP000353/NC_007974.2, CP000354/NC_007971.2, CP000355/NC_007972.2) and according to the strand orientation of the TSS. Two TSSs/promoters (TSS_3928014-2, TSS_171430-5) were excluded from further analyses because their −90 to +10 region would have extended beyond the end of the replicon.

### Hidden Markov model for motif discovery.

A custom hidden Markov model (HMM) architecture was designed for modeling combinations of putative motifs at −35 and −10 positions of promoters, referred to as “components” as follows. In this model, the two motifs are represented by contiguous paths of states with inhomogeneous (i.e., position-specific) emission probabilities for the four nucleotides. States located before, after, and between the two motifs all share the same, homogeneous emission probabilities. Alternative motifs are represented by alternative paths, where switches between these alternative paths are not allowed within motifs, but between the first set and the second set of alternative motifs. For each motif, the model allows for skipping the motif path and using the homogeneous background probabilities, instead. A schematic representation of the HMM architecture is given in Fig. S2. Parameters of the HMM (emission probabilities and transition probabilities between states) were learned by Viterbi training only considering the most likely path along the states for each input sequence. As Viterbi training gets easily stuck in local optima, training was repeated from multiple random initializations. HMM and training were implemented using the Jstacs library (99), and corresponding source code is available from the GitHub repository at https://github.com/Jstacs/Jstacs in the package projects.sigma. This model was trained from the promoters around the top 3,000 TSSs in a two-step procedure. First, the numbers of motifs in the first and second components were set to 0 and 5, respectively, and training was started from 100 random initializations. This resulted in 5 alternative motifs, one of which (see Results) was substantially more prevalent among the promoters than the other 4 and resembled the known −10 motif. Hence, promoters containing this motif were the input of a second training run. Here, the numbers of motifs in the first and second components were set to 5 and 1, respectively, and training was started from 500 random initializations to accommodate the increased combinatorial configurations when learning actual motifs in two components. In both training steps, the length of each motif was set to 10 bp, the offset of the first motif from the 5′ end of the promoter sequence was set to 40 bp, and the minimum distance between motifs was set to 10 bp. Evaluation of the model on input sequences reports the start position and sequence of the two motif matches, the log probability of the most likely (Viterbi) path (score), the components used in the Viterbi path, and the log-likelihood ratio (LLR) of the two motif models versus the homogeneous background probabilities.

### Assignment of TSSs with not correctly positioned −35 and −10 motifs to RpoD- and not-RpoD-dependent promoters.

A total of 413 of TSSs displayed not correctly positioned −35 motifs but correctly positioned −10 motifs, indicating possible blocking by RpoD-dependent RNAP holoenzyme, slow transition into the open binary complex, or transcription initiation mediated by another sigma factor. These 413 predicted promoters were sorted into the group “sba” (“sliding-blocking-another sigma factor”) (Table S1). According to the RpoD score, 11 (s)-sba sites exhibited a strong score despite the incorrect position of the −35 site (Table S1), starting with a TSS 39 bp upstream of the *ompW1* gene for an outer membrane factor. The −35 positions of these 11 sba sites were at −40 or −41 just outside the screening window, so that the uncertainty of the TSS determination of a RpoD-dependent promoter seems to be the best explanation for these 11 (s)-sba sites. These TSSs were added to the group of RpoD promoters with high probability.

A total of 107 (m)-sba sites displayed an RpoD score in the medium range and also −35 positions between −40 and −42, and in one example, at position −30, all positions were just one, two, or three positions outside the screening window. The remaining 295 (w)-sba sites had a weak RpoD score due to a −35 position between −40 and −43, with the exception of two (w)-sba sites with a −35 position again at −30. Again, these positions were up to 4 bp outside the screening window. These sites had a probability of use by RpoD that decreases with the RpoD score, while the probability of use by another sigma factor increases in a reciprocal manner. The (m)-sba sites may be RpoD promoters with low probability and the (w)-sba sites non-RpoD promoters with high probability.

A correctly positioned −35 motif but not correctly positioned −10 motif may lead to an RpoD-containing RNA polymerase binding here to form a closed binary complex; however, this polymerase should be unable to form an open complex since the −10 region is out of reach for DNA melting. Nevertheless, the −10 region could be responsible for the transcription initiation event since the position of the real TSS was obscured by the uncertainty of the TSS determination. In this case, the −10 regions should be positioned just outside the borders of the screening window. A total of 133 predicted promoters with correctly positioned −35 sites but incorrectly positioned −10 sites were sorted into the group “-ba” (“blocking or used by another sigma factor”) (Fig. S1). The 13 (s)-ba sites with a strong RpoD score had −10 sites positioned between −6 and −9, the 65 medium-strong (m)-ba sites at −19 or between −9 and −4, and the remaining weak (w)-ba sites at −19 or between −9 and −1. Again, with a declining probability following the RpoD score, these ba sites may also represent active RpoD-dependent promoters, albeit with a somewhat slow transition into the open complex, in need of an activator, or may be used by other sigma factors. As in case of the sba sites, the (s)-ba sites may be RpoD promoters with high probability, (m)-ba sites may be RpoD promoters with low probability, and (w)-ba sites may be non-RpoD promoters with high probability.

Third, −35 and −10 motifs both upstream or both downstream of the usually positions may be nevertheless responsible for the measured transcription initiation event if the −10 motif was within the limit of the uncertainty of the TSS determination used, ±5 bp outside the screening window, and the distance between the −35 and the −10 site was not outside the limits. Such −35 and −10 motifs could be outside the screening window but not more than 5 bp. Both positions were either too far upstream or too far downstream of the TSS position, but in the same direction. There were only 86 TSSs in this group, “nu” (possibly “not used by RpoD”). There were no nu sites with a strong RpoD score, as expected due to subtraction of the penalty for “wrong” positions from the portion of the RpoD score coming from the conserved −35 and −10 sequence. A number of 17 nu sites displayed a medium RpoD score with both the −35 and −10 positions just outside the screening window and had a low remaining probability to be RpoD sites. Three of these nu sites with a −35 position at −40 or −41 had a −10 position at −19, and 14 nu sites with −35 positions between −27 and −30 had −10 positions between −4 and −8. A similar geometry between −35 and −10 positions outside the search window was also true for the 69 nu sites with a weak RpoD score. These TSSs were probably not used by RpoD.

Finally, a −35 site far upstream in combination of a −10 far downstream of the usual positions, or a −35 far downstream in combination with a −10 far upstream should not associated with a RpoD-dependent transcription initiation event. All 2,347 TSSs with not correctly positioned RpoD motifs (Table 3) were also indicated in the TSS overview (Table S1), the position of the TSSs in combination with the genes and their NPKM values (Data Set S1). These 2,347 TSSs were the prominent candidates for non-RpoD promoters, together with correctly or incorrectly positioned −35 and −10 sites with a weak or even lower RpoD score.

### Accession number(s).

RNA-Seq data were deposited as BioProject no. PRJNA753702.
