# Supplementary material for: Importance of RpoD- and Non-RpoD-Dependent Expression of Horizontally Acquired Genes in Cupriavidus metallidurans
Source: Microbiol Spectr. 2022 Mar 21;10(2):e00121-22. doi: 10.1128/spectrum.00121-22 (PMC9045368; doi:10.1128/spectrum.00121-22)
Supplement: SUPPLEMENTAL FILE 2 — Supplemental material. Download SPECTRUM00121-22_Supp_2_seq8.pdf, PDF file, 3.8 MB [file spectrum00121-22_supp_2_seq8.pdf]

Supplementary Figures

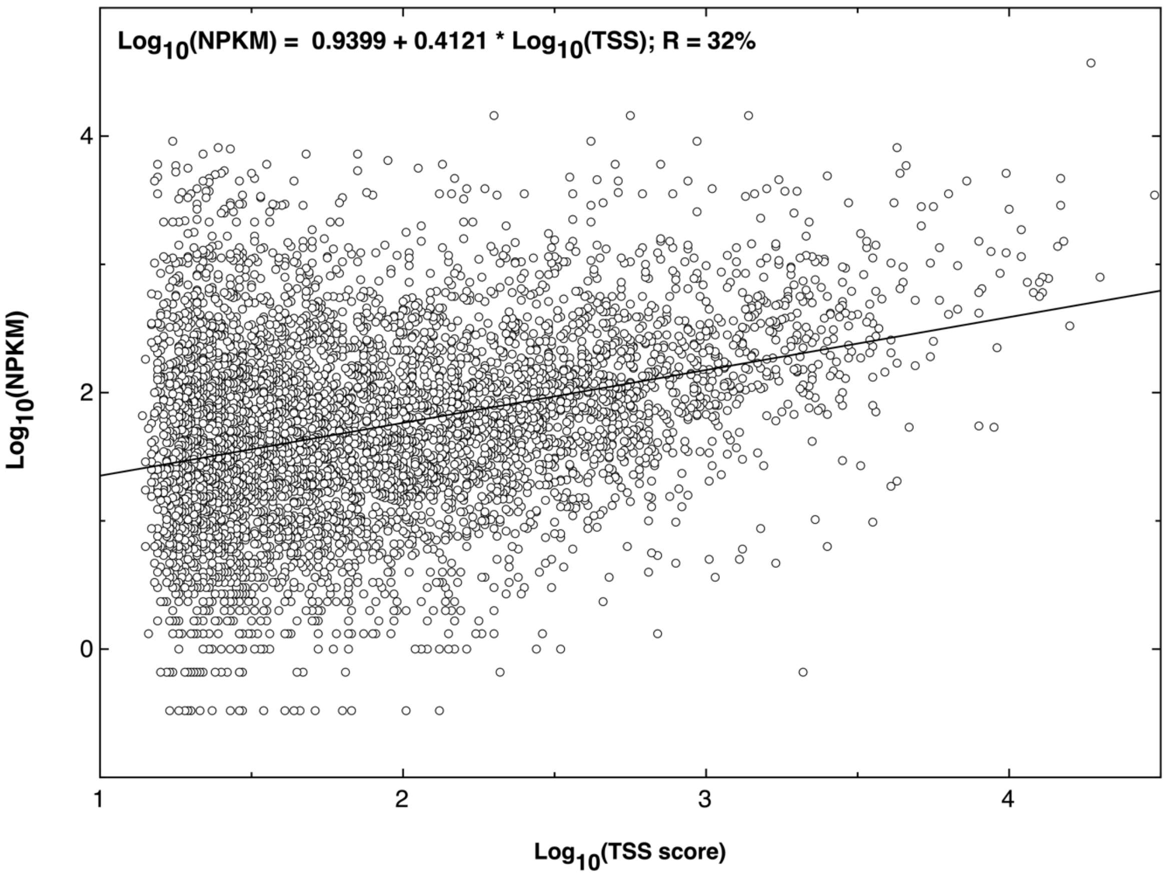

Supplementary Figure S1. Relationship of NPKM value of a gene with the TSS score of a promoter upstream of this gene. The $\log_{10}$ of both values is plotted. The line gives a linear regression with a regression coefficient of 32%.

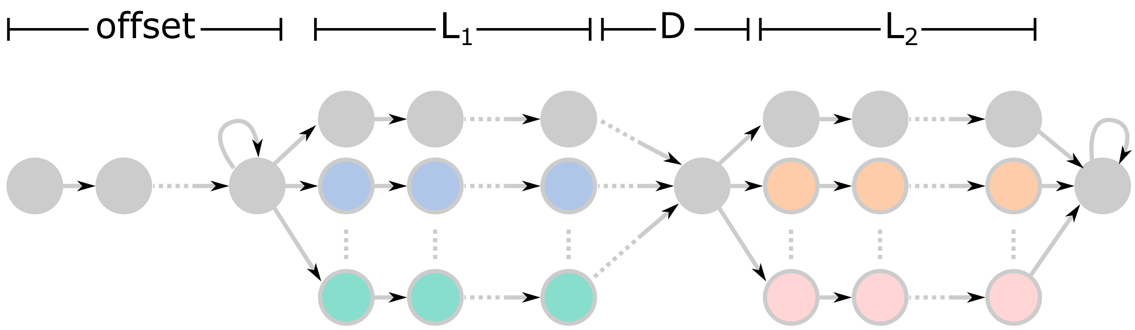

Supplementary Figure S2. Schema of the hidden Markov-model for motif discovery. Nodes filled in grey use the same, homogeneous probabilities for the nucleotides, whereas colored nodes correspond to nodes representing motifs with position-dependent probabilities. The offset from the 5’ end of the promoter, the lengths of the motifs L_1 and L_2, the minimum distance D between the two motifs, and the number of motifs in each component are adjustable by user parameters.

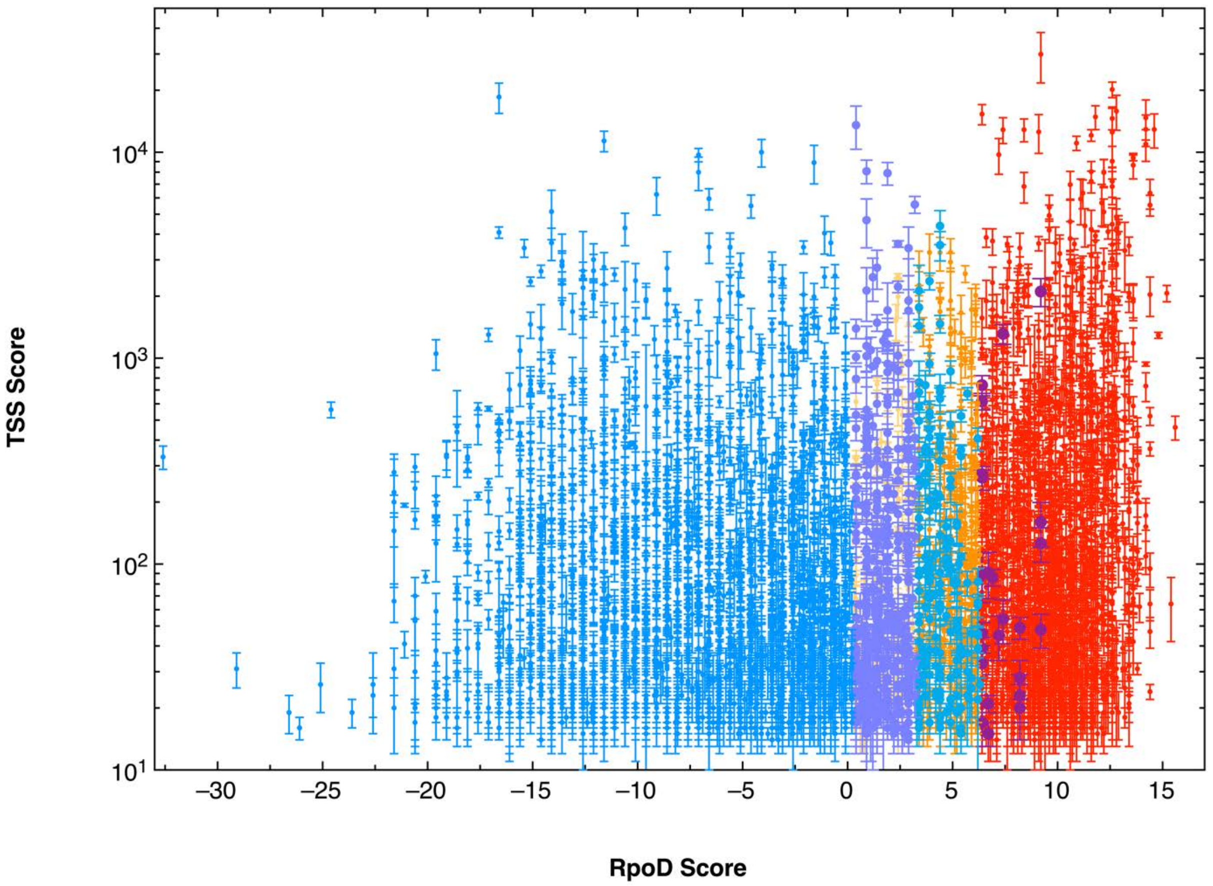

Supplementary Figure S3. Relationship of the TSS and RpoD score. The score of a given TSS was plotted against the score of the RpoD promoter motif upstream of it. The TSS scores with deviations were group into strong (red), medium (orange), weak (yellow) and no RpoD-dependent promoters (blue) and plotted against the RpoD score. Moreover, TSSs with RpoD promoter motifs that were not correctly positioned were also included for upstream sequences containing otherwise strong (purple), medium (light blue) or weak (medium blue) -35 and -10 motifs. The TSS score of three independent biological experiments is shown. The correlation between the values were R^2 = 0.15 for the strong (red) RpoD-dependent promoters and R^2 = -0.035 for not (blue) RpoD-dependent promoters.

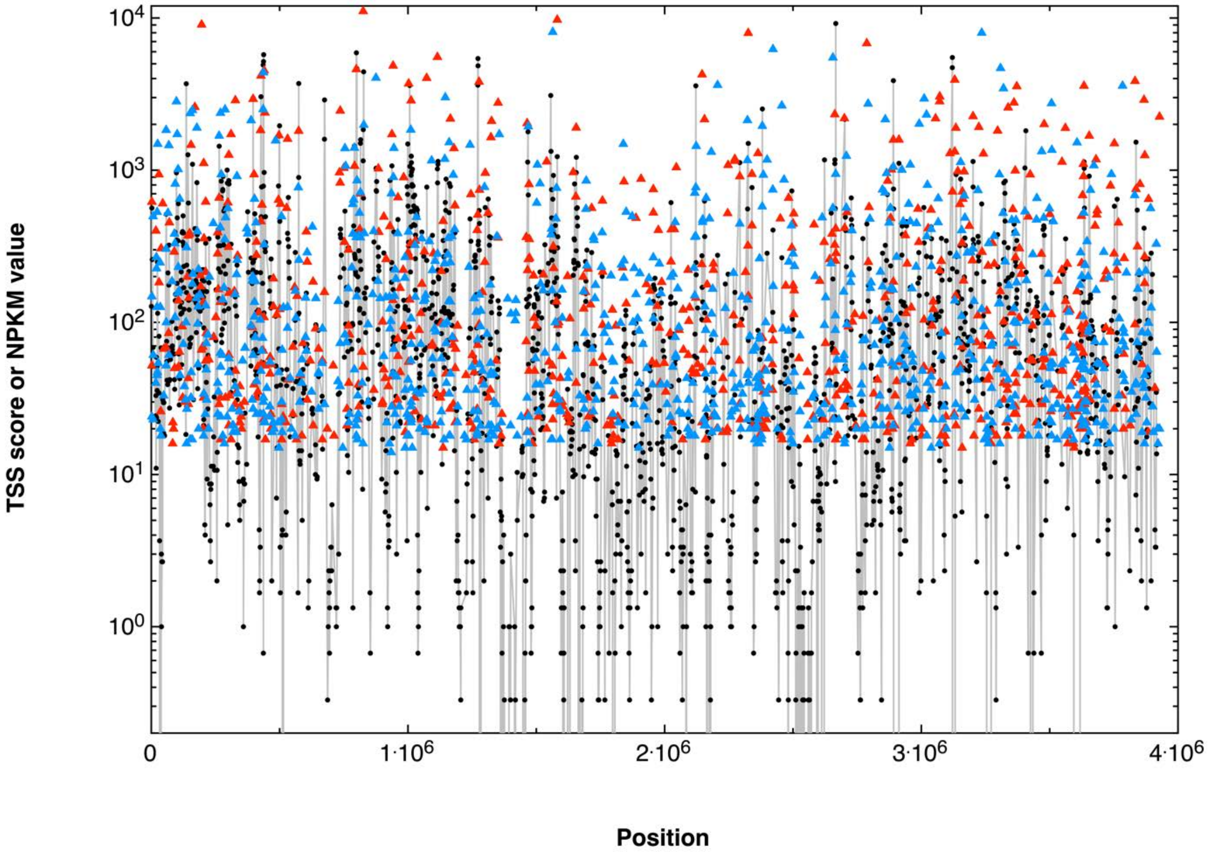

Supplementary Figure S4. Relationship of TSS score, RpoD-dependent promoters and expression level of the subsequent genes. For the chromosomal genes in “+” orientation, the NPKM values of the genes in this orientation (black dots) and the TSS score of all TSSs in this orientation (triangles) were plotted against the position. Red triangles indicate strong or medium-strong RpoD-dependent promoters, blue triangles all others.

Literature cited in the Supplement S1-S4

1. Große C, Poehlein A, Blank K, Schwarzenberger C, Schleuder G, Herzberg M, Nies DH. 2019. The third pillar of metal homeostasis in *Cupriavidus metallidurans* CH34: Preferences are controlled by extracytoplasmic functions sigma factors. Metallomics 11:291-316.
2. Große C, Grass G, Anton A, Franke S, Navarrete Santos A, Lawley B, Brown NL, Nies DH. 1999. Transcriptional organization of the *czc* heavy metal homoeostasis determinant from *Alcaligenes eutrophus*. J Bacteriol 181:2385-2393.
3. Große C, Kohl T, Herzberg M, Nies DH. submitted. Loss of mobile genetic elements in *Cupriavidus metallidurans*.

Supplementary Figure S5. Maps of genetic determinants encoding metal resistances. Maps are shown of determinants in the indicated regions with NPKM values on one DNA strand (red) or the other direction of transcription (blue). Values in orange and light blue are transcripts resulting from multiple homologous DNA regions, which could not be associated to a single locus. Above are the Rmet locus and gene names, the mean NPKM and response values (1). TSSs (flags) are indicated with the corresponding TSS score (not: RpoD score), white for scores < 50 with no promoter consensus motifs indicated, red shades for strong (>1000, red), medium (100-1000, orange) or weak (50-100, light orange) RpoD promoters, blue shades (strong, medium, light) according if not associated to the RpoD model.

Active determinants encoding transenvelope efflux systems for divalent metal cations.

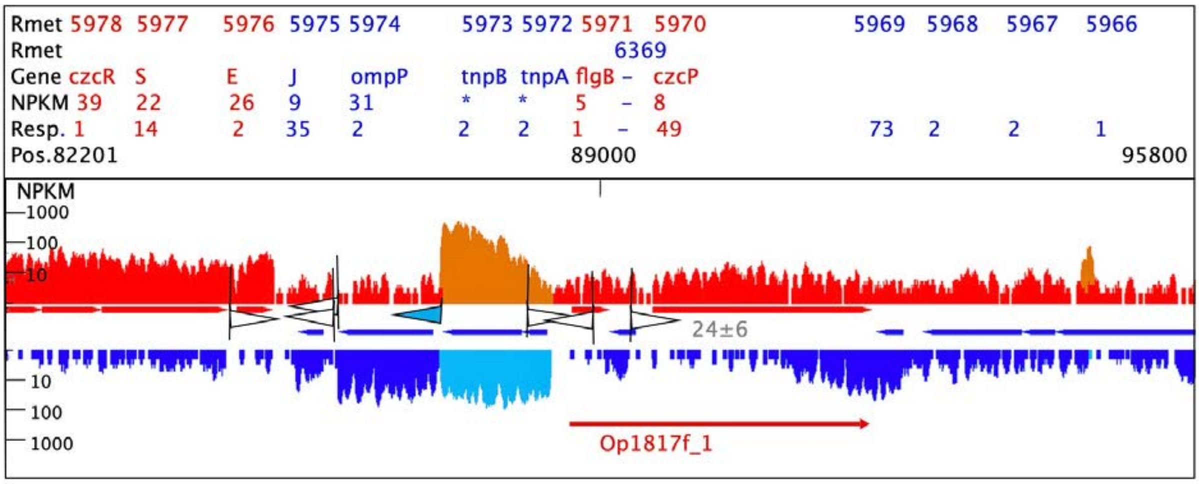

Panel A. The *czc* determinant on plasmid pMOL30, part 2, the *czcP* region with Op1817f_1. The *czcJ* gene and the *flgB-czcP* operon further downstream from the main *czc* determinant were again only weakly expressed in TMM-grown cells. The *czcJ* gene had two weak TSSs 47 and 92 bp upstream, one with a strong RpoD score, the other was no RpoD promoter. The *czcP* gene for a P_{IB4}-type export system had a weak TSS 41 bp upstream not assigned to RpoD. The genes *czcP* and *flgB* may be separated by an open reading frame Rmet_6369 on the other DNA strand, which was, however, not expressed.

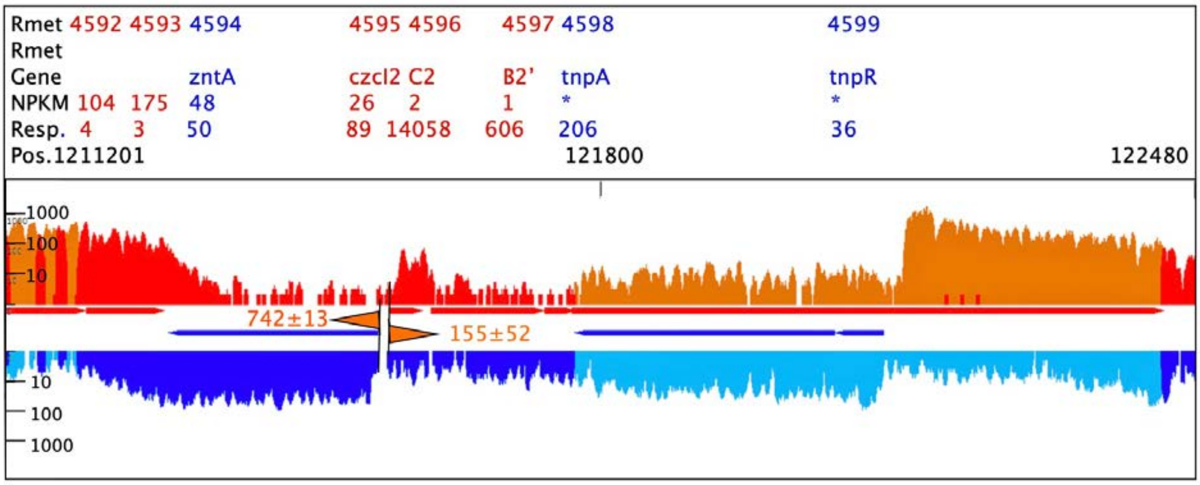

Panel B. The ancestral *czc2* determinant on the chromid; part 1, *zntA* region. Expression of *zntA* for the main zinc-exporting P_{IB2}-type was expressed with an NPKM value of 48 from a RpoD-dependent medium-strong promoter 12 bp upstream of *zntA* with sufficient TSS score. The TSS for *czcI2C2B2'* also started from a RpoD-dependent promoter. This TSS was annotated at position +2 of the *czcI2* gene, the second base pair of the AUG start codon. The score for a RpoD promoter responsible for this TSS was only 3.4, however, the search algorithm identified as -35 motif a sequence AATGTTTG at position -39. If instead the

sequence TGTTTG at position -37 was scored, which moved the -35 region also more closely to the ideal TATAAT sequence at position -10, the score increased to 8.40, a strong RpoD score. Consequently, this TSS at +2 of the *czcI2* gene started from a RpoD-dependent promoter that was strong enough for the transcript abundance of *czcI2*. There was no evidence from alignments of *czcI* genes that *czcI2*-translation started at a triplet further downstream. Transcription of *czcI2* began at a low NPKM value of the 5' end of the gene, increasing to a mean NPKM value of 26, and dropped sharply downstream of *czcI2*, so that *czcC2* and the truncated *czcB2'* were nearly not expressed in non-amended TMM. Since the clustering window of the n_{io} counts was ±5 bp, the real transcriptional starting point of *czcI2* may be close to the +1 position, so that the mRNA may start with the AUG at the 5' end. This would indicate an alternative translation initiation of *czcI2* by lmRNA initiation as in case of *rpoN.* While *czcI2* was expressed in TMM-grown cells but not *czcC2*, both genes were strongly responding to metal stress.

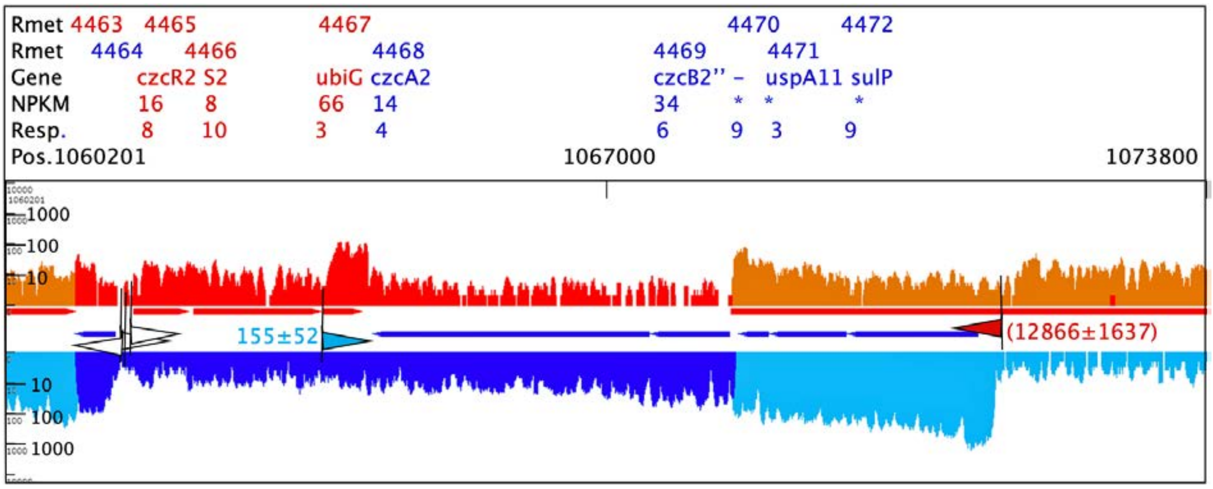

Panel C. The ancestral *czc2* determinant on the chromid; part 2, *czcR2S2* region. Stars indicate no NPKM value due to multiple homologous regions in the genome. In the other half of *czc2*, two TSSs mediated a low-level transcription of *czcR2S2* with NPKM values of 16 and 8, respectively, which are around the usual cut-off value for NPKM or 10. While the TSS 118 bp upstream of *czcR2* was a (w)-sba promoter with weak but not correctly spaced RpoD consensus motifs, the TSS 55 bp upstream of *czcR2* displayed a strong RpoD score albeit a low TSS score. Both promoters may be activated under metal stress conditions, for instance by CzcR2. The *czcB2''A* genes were fused to upstream genes with a strong RpoD-dependent TSS upstream of *sulP* for a sulfate transporter. The genes were transcribed but should not be translated due to a lacking 5'-end of *czcB2''.*

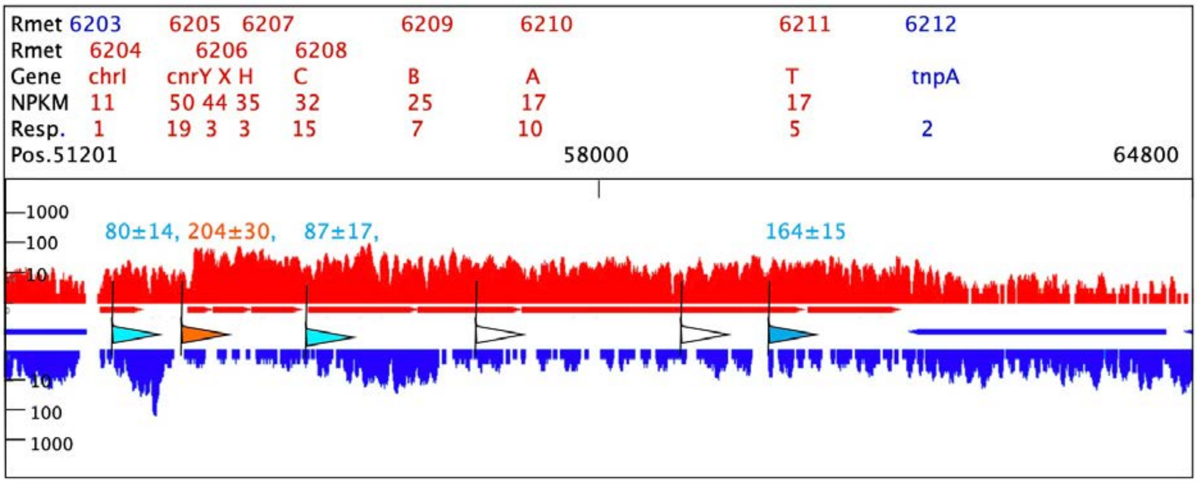

Panel D. The *cnr* determinant on plasmid pMOL28. Transcription of the determinant was initiated from two weak TSSs 966 bp upstream of *cnrY* and 24 bp upstream of *cnrC*, and two

medium-strong TSSs 23 bp upstream of *cnrY* and 763 bp upstream of *cnrT*, respectively. The medium-strong *cnrYp* promoter 23 bp upstream of *cnrY* was a RpoD-dependent promoter with a strong RpoD score while *cnrYp* 966 bp upstream of *cnrY* and *cnrCp* 24 bp upstream of *cnrC* were no RpoD promoters, which agrees to the fact that *cnr* expression is under control of the sigma factor CnrH.

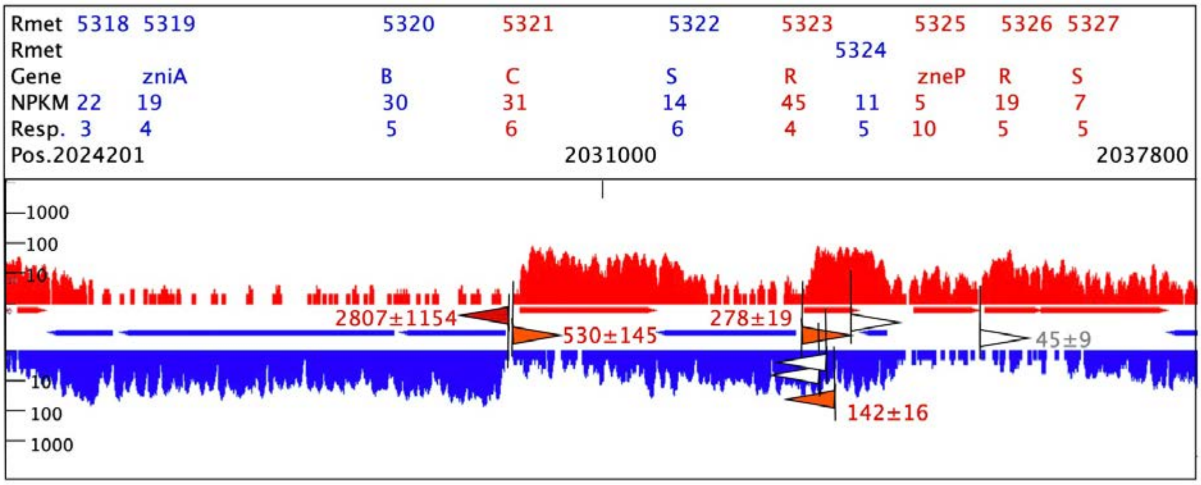

Panel E. The *zni/zne* determinant on the chromid; part 1. The operons *zniBA-Rmet_5318* (Op1571r_1), *zniC* (Op1572f_2), *zniS* (Op1573r_1) and *zniR* (Op1574f_1) were expressed in unchallenged *C. metallidurans* cells and were clearly under control of RpoD-dependent TSSs. The TSS scores were much higher than the NPKM values, indicating the possibility of rapid mRNA degradation. The expression levels in non-challenged cells in combination with the responsiveness to metal stress conditions identified the Zni system as a minor transenvelope efflux system, which may contribute to metal homeostasis and is under RpoD- and ZniRS control.

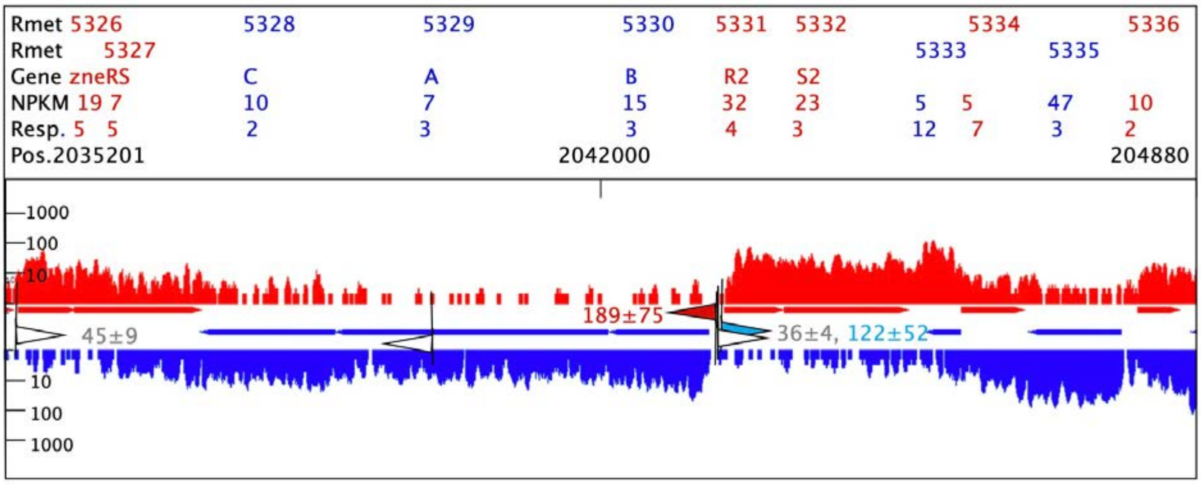

Panel F. The *zni/zne* determinant on the chromid; part 2. The operon *zneBAC* (Op1577r_1) was also expressed and under RpoD control while only weak, not-RpoD-associated TSSs were upstream of the operons *zneRS* (Op1576f_1) and *zneR2S2* (Op1578f_1). Expression of *zneP* (Op1576f_1) was very low but the gene was responding to metal stress. It could be transcribed from the RpoD-dependent promoter upstream of *zniR* and under metal stress also from the weak (white flag in Panel E) but clearly RpoD-dependent promoter within *zniR*. The weak TSS upstream of *zneRS* was not RpoD-dependent. Upstream of *zneR2S2* were a RpoD and a non-RpoD promoter.

Inactivated determinants

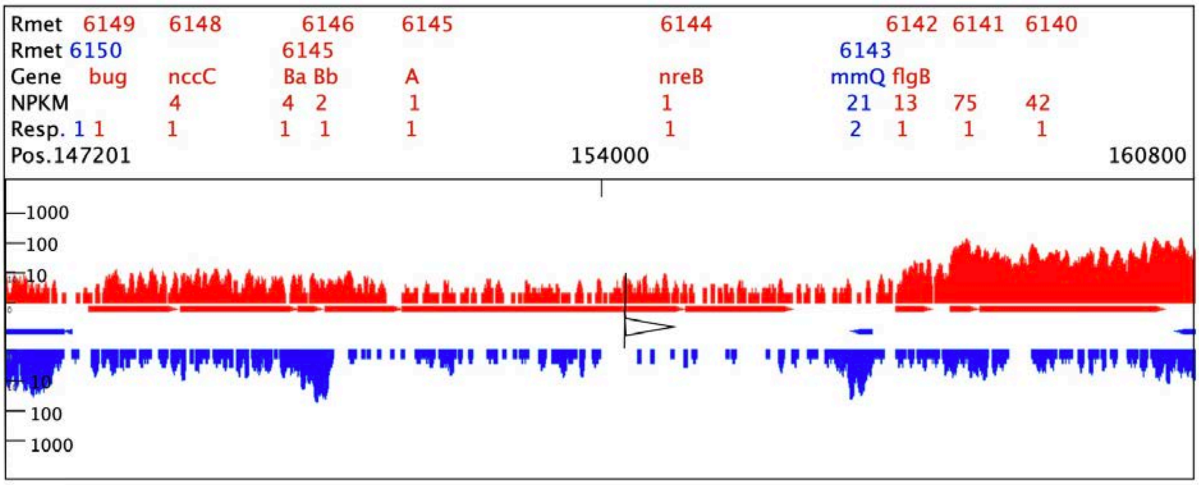

Panel G. The *ncc* determinant on plasmid pMOL30. No TSS could be identified upstream of this inactivated metal resistance determinant, which agreed to the absence of *nccH* gene for a *ncc*-specific sigma factor in *C. metallidurans*. In contrast to *cnr*, no RpoD-dependent promoter mediated expression of *ncc* under non-challenging conditions. A weak TSS 850 bp upstream of *nreB* could not be associated to RpoD and *nreB* was not expressed in TMM-grown cell (NPKM = 1) and not responding to metal stress. The determinant was not expressed, contained no TSS and the frame-shift mutation in *nccB* should result in a premature transcription termination event so that *nccA* would not be expressed anyhow.

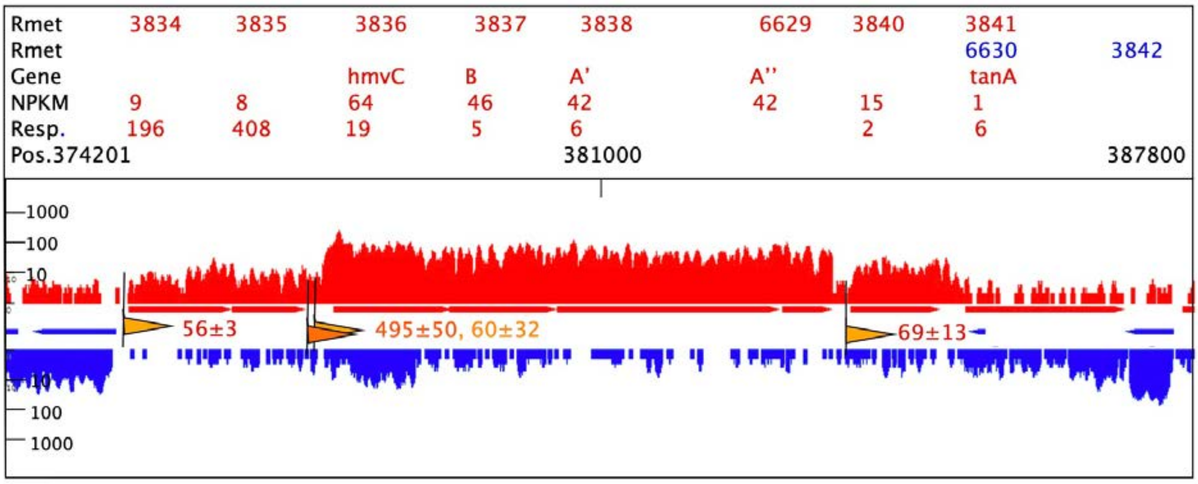

Panel H. The *hmv* determinant on the chromid. Operon Op1066f_2 on the chromid with *hmvCBA'/A''* has the *hmvA* gene interrupted by a frame-shift mutation. It started from a strong TSS 181 bp upstream and a medium TSS 71 bp upstream of *hmvC* and both TSSs could be associated to RpoD. Two flanking operons on the same DNA strand started from TSSs, which also could be assigned to the RpoD model.

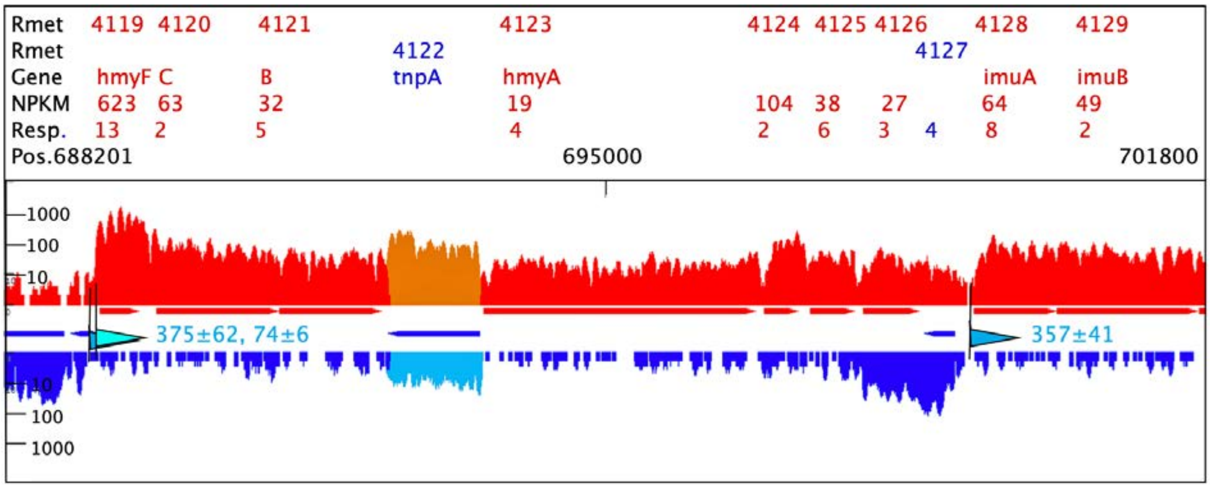

Panel I. The *hmy* determinant on the chromid. The *hmy* determinant on the chromid was split into two halves by a transposon insertion between *hmyB* and *hmyA*, which inactivates the genes. The first half is Op1160f_1 with *hmyFCB* and a strong expression of *hmyF* from a strong and a medium strong TSS, which could both not be associated to RpoD. The *hmyA* gene showed some expression (NPKM = 19) but no TSS. Expression could be the result of a read-through from *hmyB*.

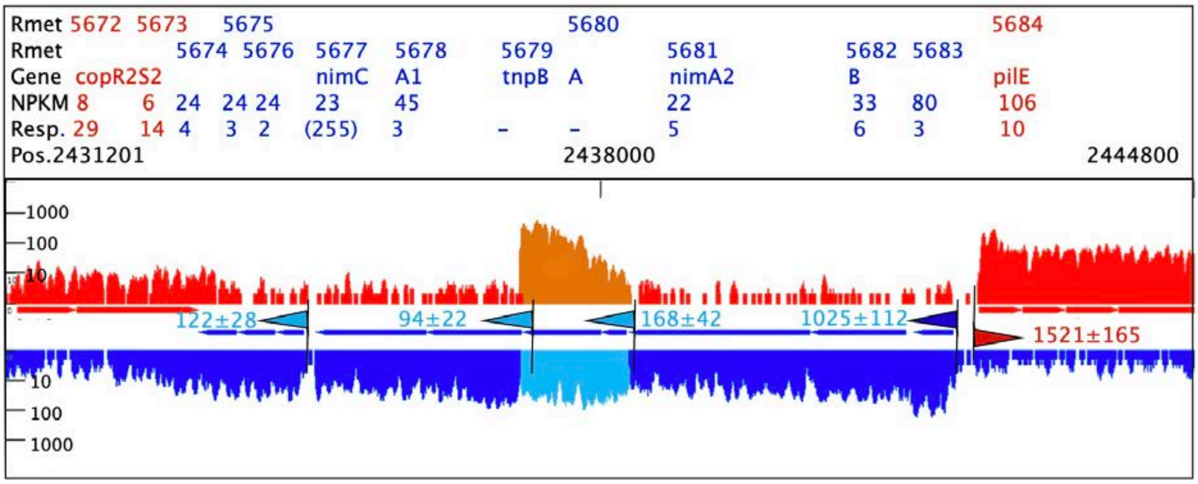

Panel J. The *nim* determinant on the chromid. The *nim* determinant on the chromid is adjacent to a copper resistance cluster and also interrupted by a transposon insertion into the *nimA* gene. The *nim* genes were expressed, TSS were identified but could not be associated to RpoD. A strong RpoD-associated TSS started transcription of a pilus synthesis operon on the other DNA strand.

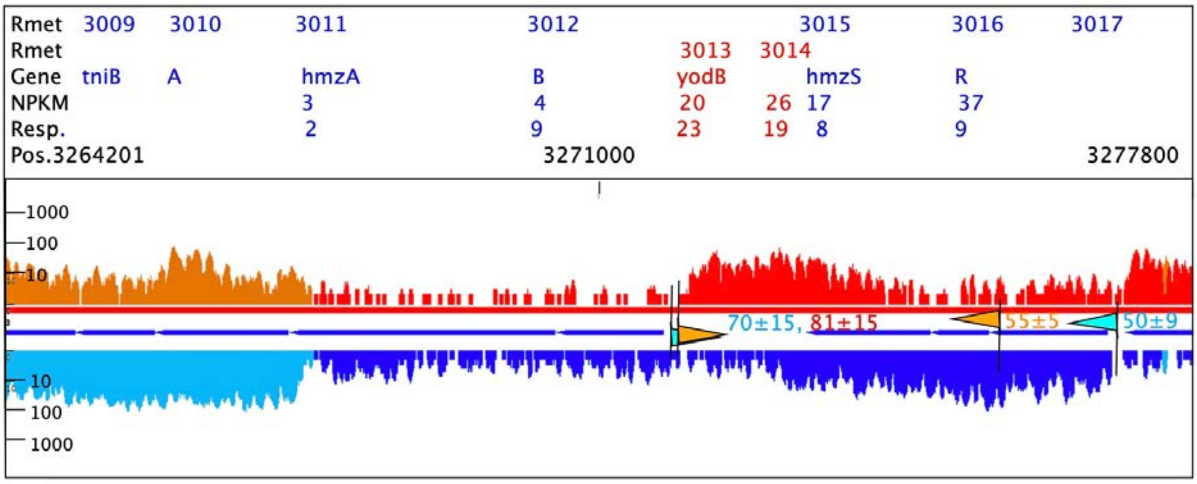

Panel K. The *hmz* determinant on the chromosomal island CMGI-4. A last determinant was *hmz* and located on the chromosome, again with a transposon adjacent. While *hmzBA* were nearly not expressed and no TSS could be identified, the regulatory genes *hmzRS* was expressed from a RpoD-dependent TSS*.*

Transenvelope efflux systems involved in copper resistance.

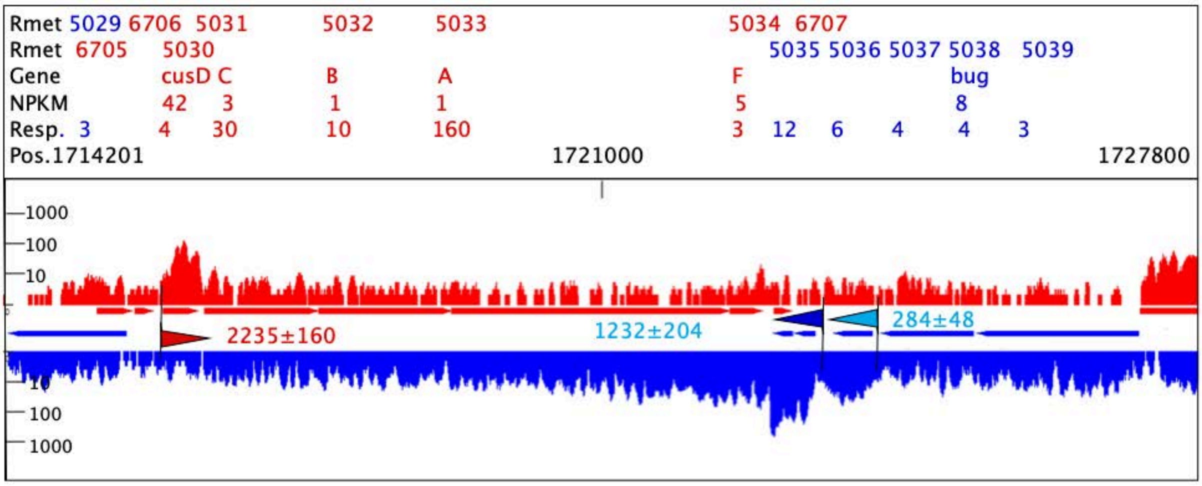

Panel L. The *cus* determinant on the chromid. The *cusDCBA* determinant operon Op1480f_2 on the chromid was not expressed in non-challenged cells with the exception of the *cusD* gene at the 5' end, which encodes an uncharacterized protein. This operon was under control of a strong TSS upstream of *cusD* and this TSS could be clearly associated to the RpoD model. The expression level of *cusCBAF* was very low with NPKM values of 3, 1, 1, 5 respectively, although the TSS representing the *cusDp* promoter had a strong TSS score of 2235 and yielded a *cusD* transcript with NPKM = 42. A terminator may be located downstream of *cusD* but this would not explain the small NPKM value of *cusD* with respect to the strong TSS score of its promoter. Alternatively, transcription from two promoters downstream of the *cus* operon on the other DNA strand may yield an antisense transcript that de-stabilizes the *cusDCBAF* transcript. This would be the first example of an antisense regulation in *C. metallidurans.* The scores of the TSSs in the antisense direction would be strong enough to explain such an event. Both were non-RpoD-promoters, so that another sigma factor should be involved here.

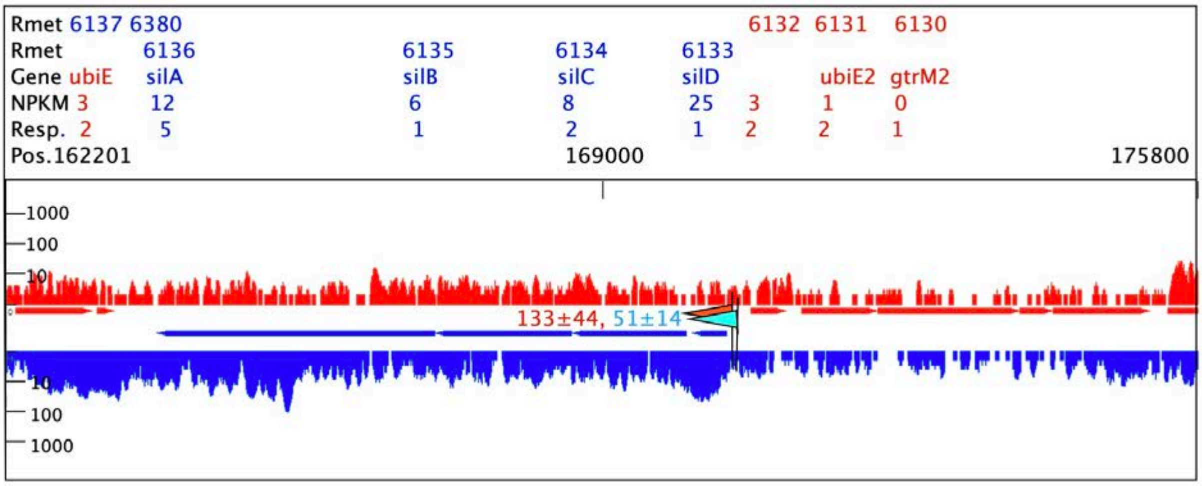

Panel M. The *sil* determinant on plasmid pMOL30. The second determinant for a copper-exporting transenvelope system is *silDCBA* in operon Op1874r_1 on plasmid pMOL30, which was also weakly expressed in unchallenged cells, again with the exception of the *cusD* paralog *silD*. The *sil* genes were not responding to metal stress but silver was not part of the challenging metals. Two TSSs were upstream of *silD* and strong enough for *sil* expression, one of these could be associated to the RpoD model.

<u>Other transition metal resistance determinants.</u>

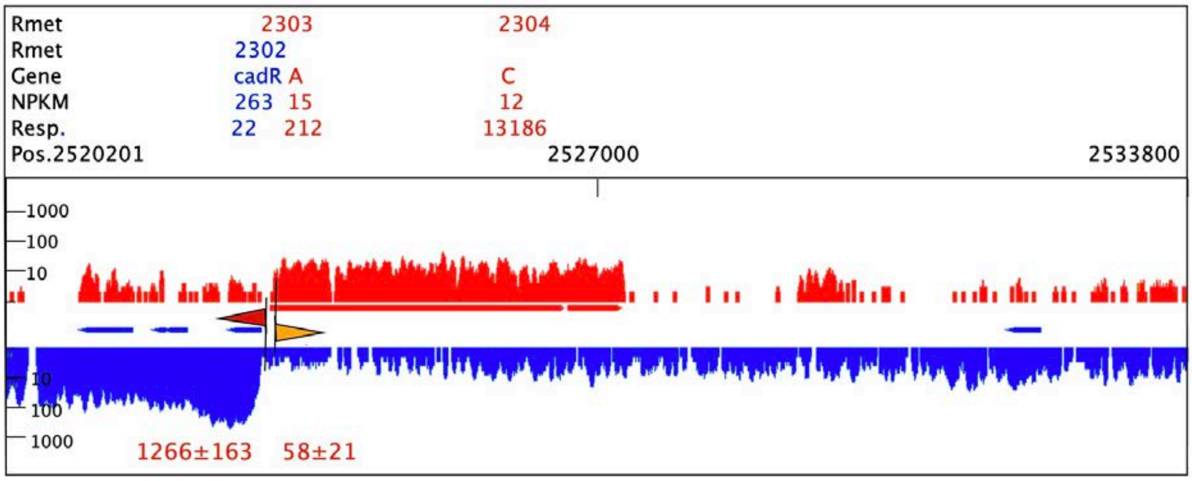

Panel N. The *cadA* determinant on the chromosomal island CMGI-1. The chromosomal *cad* determinant, composed of the *cadR* gene for the MerR-type regulator (2) in one direction of transcription and *cadAC* for the CadA P_{IB2}-type ATPase and protein CadC, started from two RpoD-dependent TSSs in a common promoter region, a strong TSS for *cadR* and a weak TSS for *cadA*, which was interestingly 9 base pairs downstream of the annotated *cadA* ATG. This indicated a later translation initiation start site, e.g. at the ATG 14 triplets further downstream than the annotated ATG, although a respectively truncated CadA protein ($CadA_3$) mediated no cadmium resistance in *Escherichia coli* compared to $CadA_2$ as annotated (3). An explanation could be that *cadA3* expression in *C. metallidurans* occurs as lmRNA initiation but *E. coli* was not able to translate such a message. The *cad* determinant was strongly responding to metal stress and under control of RpoD and CadR.

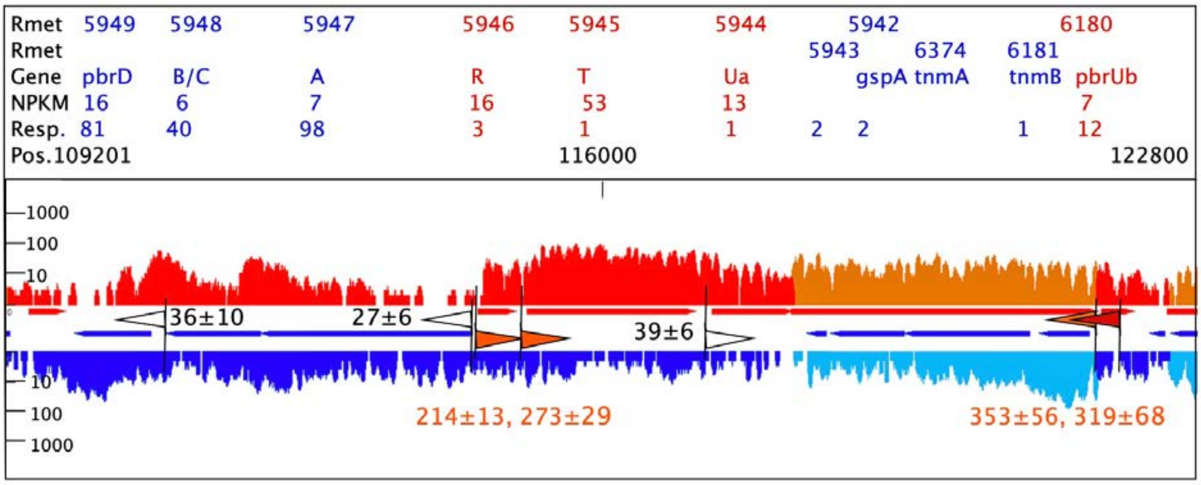

Panel O. The *pbr* determinant on plasmid pMOL30. The *pbr* lead resistance region on pMOL30 is organized as divergonic operons *pbrAB/CD* (Op1808r_1) and *pbrRTU* (Op1807f_1) with a transposon insertion interrupting *pbrU*. The *pbrAB/CD* genes were expressed in TMM-grown cells and responding to metal stress, *pbrRT* expressed but not responding. Only weak TSSs could be associated to *pbrAB/CD* for the P_{IB2}-type ATPase PbrA, a putative periplasmic protein PbrC/D, which is organized as two polypeptides in other bacteria, and a lead-binding protein PbrD (4), indicating a repressed operon in the non-challenged cells. The *pbrAp, pbrRp, pbrTp* and *pbrB/Cp* promoters displayed a strong RpoD consensus score. While transcriptional initiation activity from *pbrAp* and *pbrB/Cp* was below a TSS score of 50, activity of the TSSs 21 bp upstream of the gene for the MerR-type regulator *pbrR* and 39 bp upstream of *pbrT*, annotated as lead uptake protein, respectively, were above a TSS score of 200 (4-6). Lead resistance was under RpoD control, PbrR and PbrT may be present in the cells to watch out for lead ions so that the *pbr* determinant can be activated when needed.

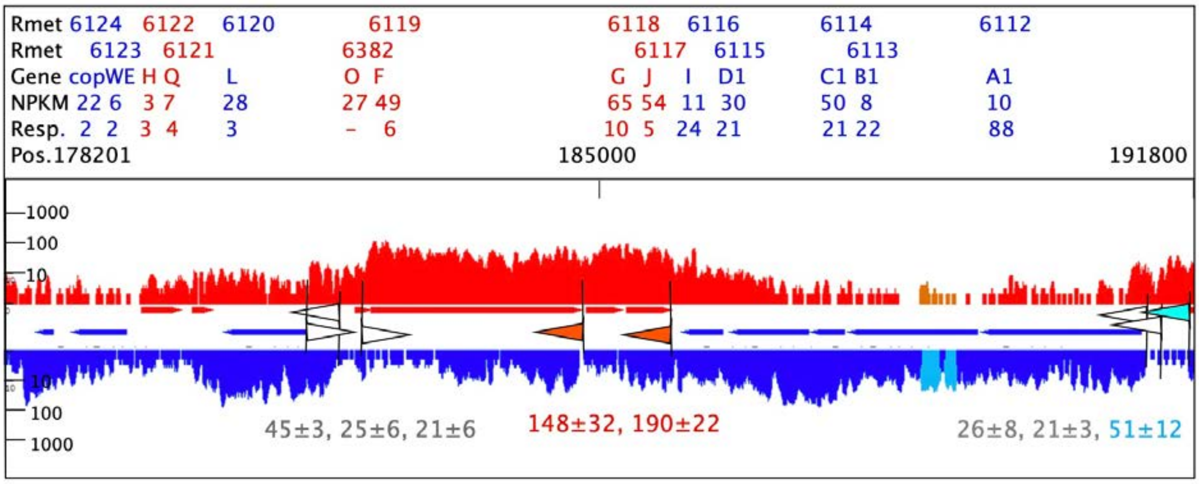

Panel P. The *cop* determinant on plasmid pMOL30, part 1. The huge multi-gene copper resistance region *cop* on plasmid pMOL30 is organized in nine operons (also Suppl. Fig. 6Q): (i) Op1871r_1 with *copW* but no TSS; (ii) Op1870r_1 with *copE* and also no TSS found; (iii) Op1869f_1 with *copHQ* without TSS; (iv) Op1868r_1 with *copL* and a weak TSS upstream; (v) Op1867f_1 with *copOFGJ* with weak TSSs 661 bp upstream of *copO* and 101 bp upstream of *copF*; (vi) Op1866r_1 with *copA1B1C1D1J* in a clear pentacistronic operon with three weak TSSs upstream of *copA1*; (vii) Op1865f_1 with *copR1S1N* tricistronic with several weak TSS far upstream of this region; (viii) Op1864r_1 with *copMK* with a strong RpoD-promoter 2.3 kb upstream of *copM*; and (ix) Op1863r_1 with *copTV* and a RpoD-dependent promoter 22 bp upstream of the *copT* gene. Main products of this large region at the copper-exporting P_{IB1}-type ATPase CopF and the periplasmic Cu(I)-oxidase CopA1 (7). One of the three TSSs upstream of *copA1* displayed a strong RpoD motif but low TSS score, indicating a repressed state. The other two TSSs were none RpoD-dependent and scoring as “(m)-sba”, indicating a

possible influence of other sigma factors on *copA1* expression. The *copFp* promoter was clearly RpoD-dependent but on a low level of activity. Expression level of all these genes was very low in unchallenged cells, between NPKM 3 for *copH* to 65 for *copG*, corresponding to the fact that this region mediates a high level of copper resistance but only in copper-induced *C. metallidurans* cells (8), as was also demonstrated by the response values of the *cop* genes. Reminiscent to *cus*, two strong RpoD-associated TSSs started transcription in an anti-sense direction of *copOFGJ*, which may help to repress the determinant when not needed. Otherwise, the two-component regulatory system CopR1S1 may be responsible for *cop* activation.

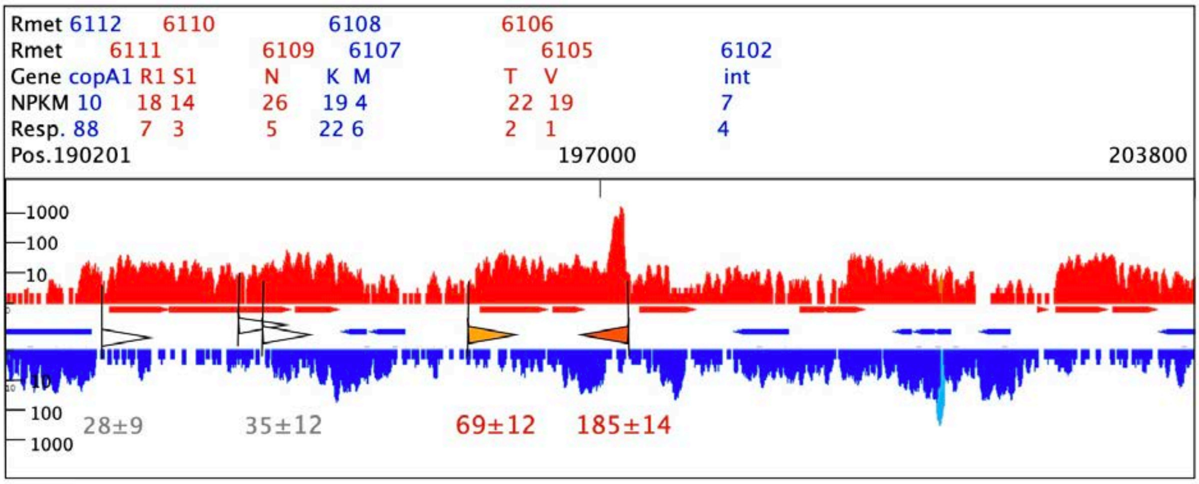

Panel Q. The *cop* determinant on plasmid pMOL30, part 2. The *copR1p* upstream promoter with a TSS score of 28 is clearly a non-RpoD-promoter so that another sigma factor may be responsible for expression of the *cop* activator. This *cop* determinant on plasmid pMOL30 was under control of RpoD, at least one other sigma factor, probably of CopR1S1, control of *copR1S1* by a non-RpoD sigma factor, and a possible antisense action on *copF* expression under RpoD control.

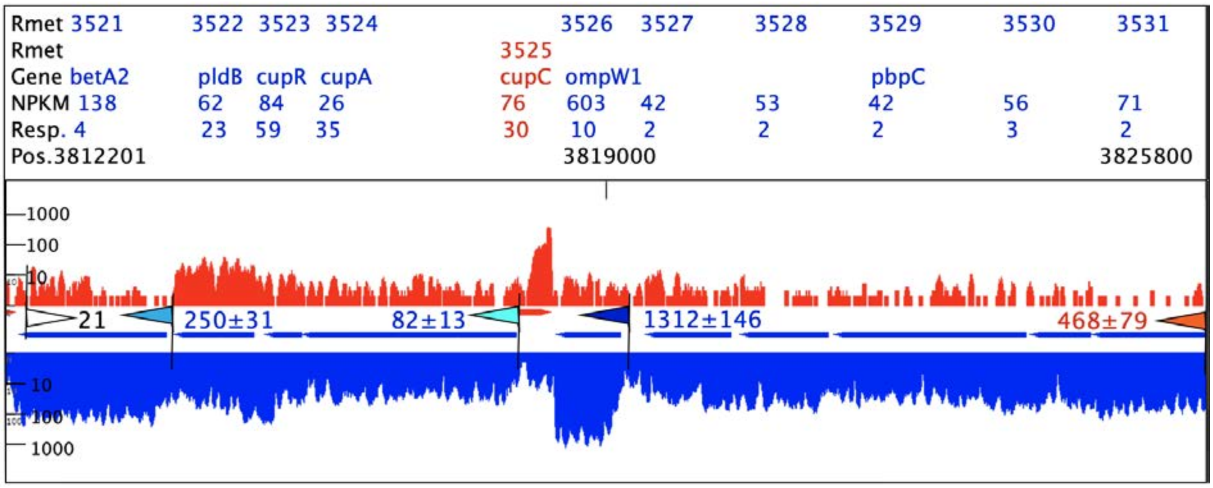

Panel R. The *cup* determinant on the chromosome. Two other determinants involved in copper resistance in *C. metallidurans* are the *cup* determinant for the P_{IB1}-type ATPase CupA on the chromosome outside of any chromosomal island and the *cop2* determinant around the CopA2 periplasmic Cu(I) oxidase on the chromid shown in Panel S. Operon Op0974r_1 with *cupAR* was transcribed from a non-RpoD-associated, sufficiently strong TSS 38 bp upstream of *cupA*, while no TSS was found upstream of Op0975f_1 *cupC*, despite an expression level of NPKM = 76. Expression of *cupAR* is probably regulated by CupR (9) but again, a non-RpoD sigma factor seems to be required for *cup* expression.

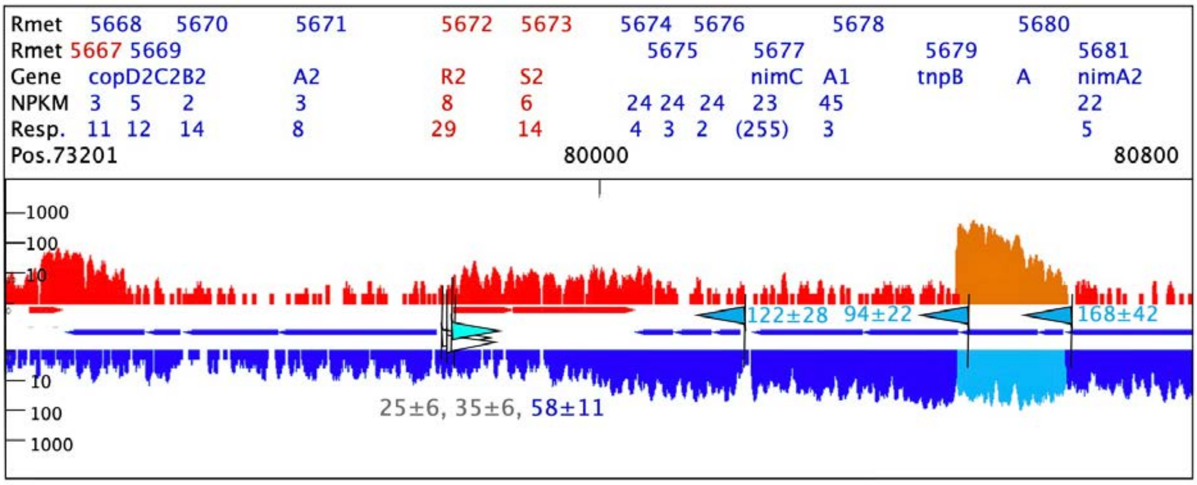

Panel S. The *cop2* determinant on the chromid. The *cop2* determinant on the chromid is organized in the two divergently oriented operons. Op1695r_1/2 with *copA2B2C2D2* was nearly not expressed and no TSS was found. The operon is clearly responding to metal stress so that the lack of an identified TSS was probably due to a lack of transcription. Op1696f_1 with *copR2S2* for a two-component regulatory system started from three weakly scoring TSSs. A promoter 147 bp upstream of *copR2* possessed a strong RpoD consensus, the promoters 75 bp and 46 bp upstream were non-RpoD promoters. The genes *copR2S2* may be expressed by RpoD and at least one other sigma factor.

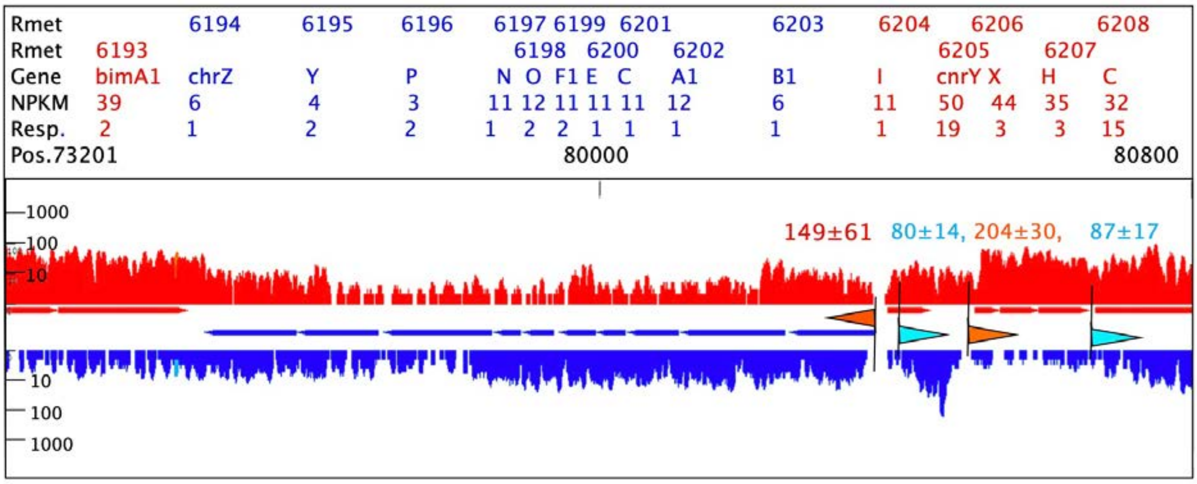

Panel T. The *chr* determinant on plasmid pMOL28. The *chr* chromate resistance determinant on plasmid pMOL28 was organized as deca-cistronic operon around the *chrA1* gene for a chromate efflux pump (10-12) and clearly under RpoD control. Since chromate was not part of the metal cocktail challenging *C. metallidurans* strains (1), *chr* was not responding and served as negative control here. No TSSs for the divergently transcribed *chrI* gene for a possible regulator was in the direct vicinity of *chrI*, the closest TSS was 1553 bp upstream within *chrA1*, not associated to RpoD and had a TSS score of 61±16 (Suppl. Data Set 1; TSS not shown in the figure above). A possible CnrH-dependent promoter for the adjacent *cnrYXH* genes was within the *chrI* genes and 43 bp downstream of the *chrI* start codon. Should *chrI* start later than predicted, the gene may be expressed together with *cnr* under CnrH and nickel control. The *chr* genes were nearly not expressed in non-challenged cells with NPKM values between 3 and 12.

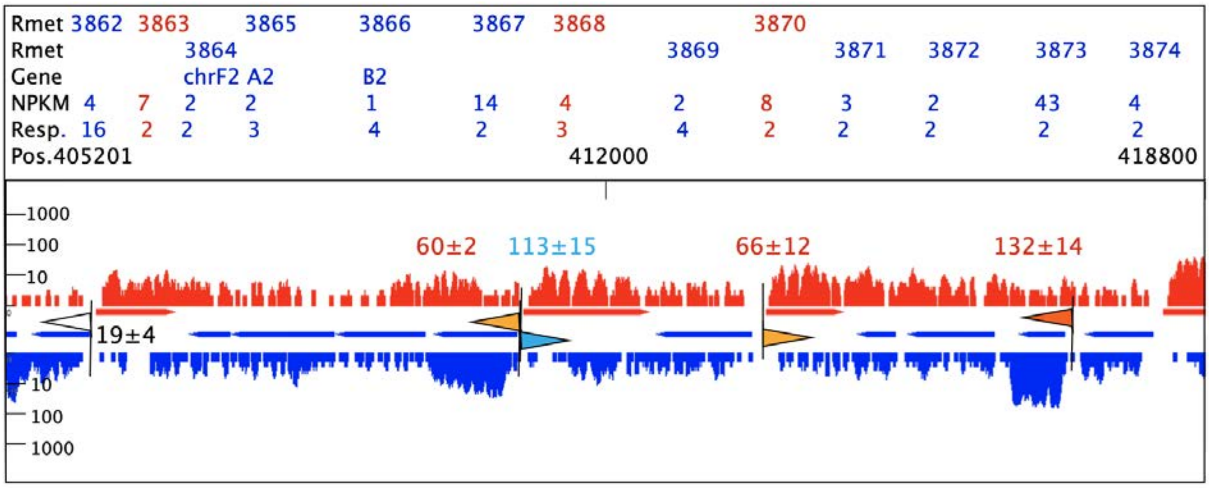

Panel U. The *chr2* determinant on the chromid. Expression of a second *chr2* resistance determinant Op1075r_2 on the chromid was even lower than that of *chr*, between NPKM 1 and 2 but also from an TSS with a RpoD motif upstream. In case of *chrB1* on pMOL28 (Panel T) and the open reading frame Rmet_3867 upstream of *chrB2* on the chromid, the distance between the TSS and the start codon was 0, again indicating alternative translation initiation by lmRNA initiation (13). Chromate resistance was under RpoD control with a remote possibility of a contribution of CnrH via ChrI.

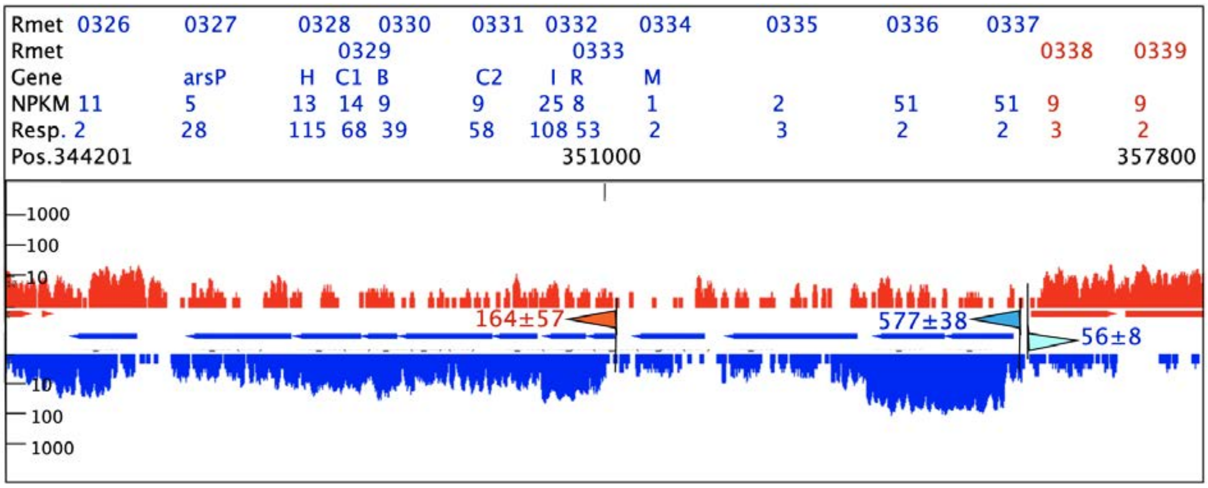

Panel V. The *ars* determinant on the chromosomal island CMGI-7. The *arsRIC2BC1HP* determinant Op0094r_2/3 on the chromosomal island CMGI-7 was on an expression level between NPKM 5 and 25, also from a TSS immediately upstream of *arsR*, which was RpoD-dependent. The respective transcript should start with the start codon of *arsR*, indicating again lmRNA-specific translation initiation (13). The *arsM* gene for an arsenate methyl-transferase upstream of *arsR* was the last gene of the tetracistronic operon Op0094r_1 upstream of the *arsRIC2BC1HP* operon. Transcription started from a sufficiently strong but not RpoD-associated TSS 20 bp upstream of Rmet_0337 but only this gene and Rmet_0336 downstream exhibited some level of expression. Again, while most genes were under RpoD control, genes that may enlarge the capability of arsenate resistance in *C. metallidurans,* for instance by allowing arsenate methylation, were under non-RpoD control.

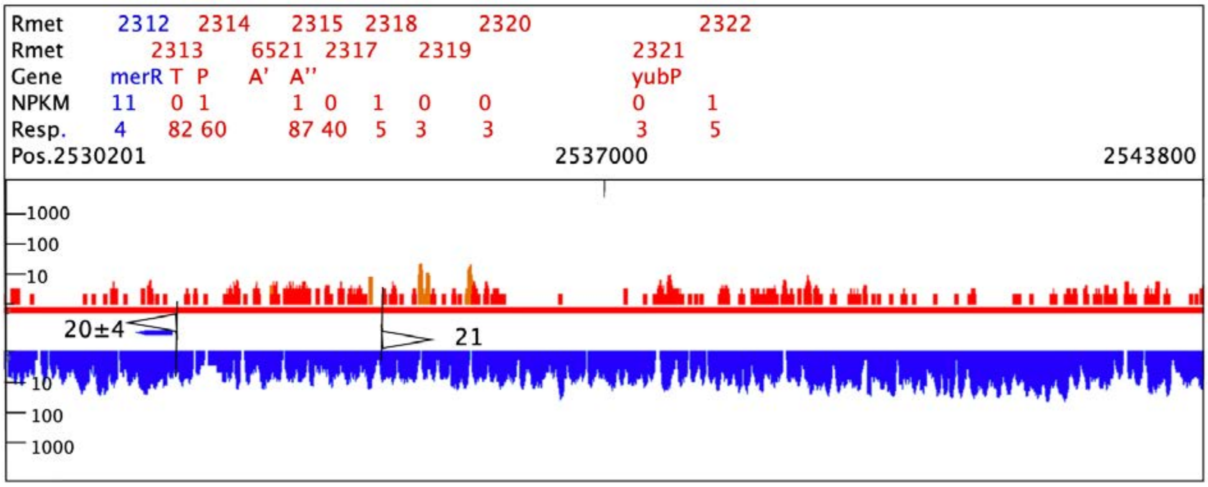

Panel W. The *mer* determinant on the chromosome. Expression of the *mer* determinant on the chromosome was so low that no open reading frames could be assigned to the RNASeq sequences, and only one weak TSS was found upstream of the *merR* gene.

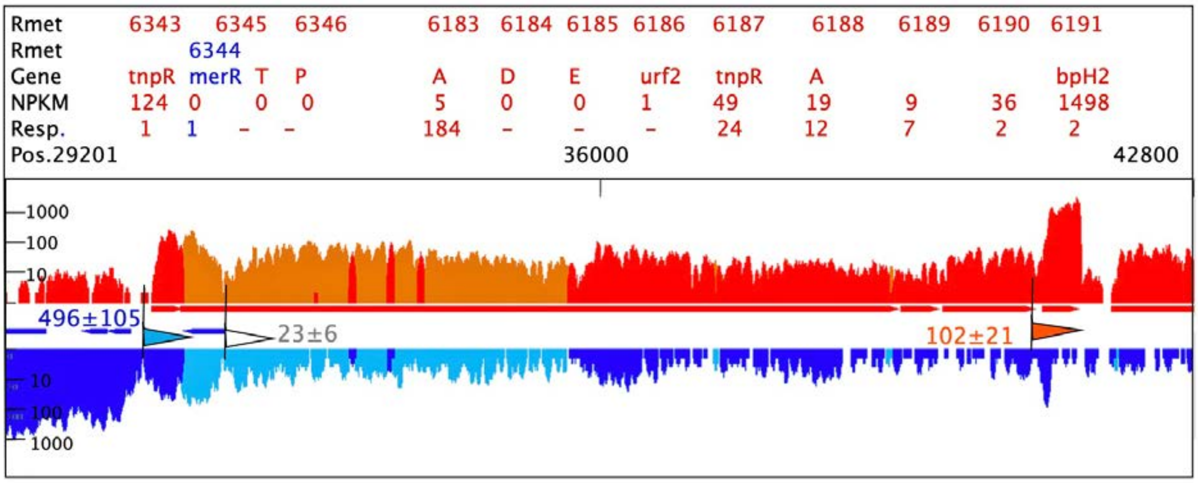

Panel X. The *mer* determinant on plasmid pMOL28. Transcripts of the *mer* determinant on plasmid pMOL28 could not be assigned to this region due to the high degree of homology between this and the *mer* determinant on pMOL30.

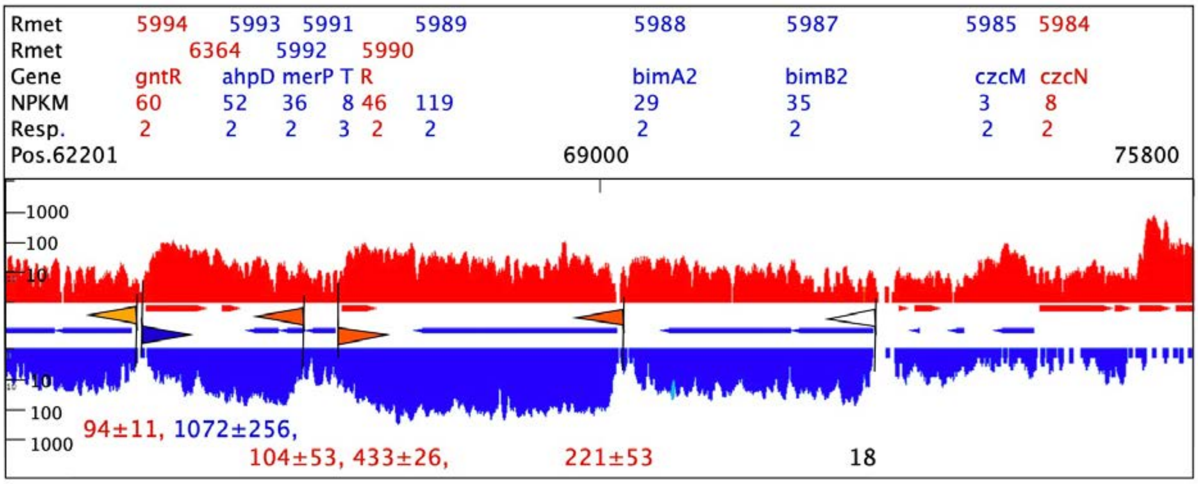

Panel Y. The *mer* determinant on plasmid pMOL30, part 1. A *merP* gene apart from the *merTPADE* region on plasmid pMOL30 (but nevertheless on plasmid pMOL30) was under RpoD control

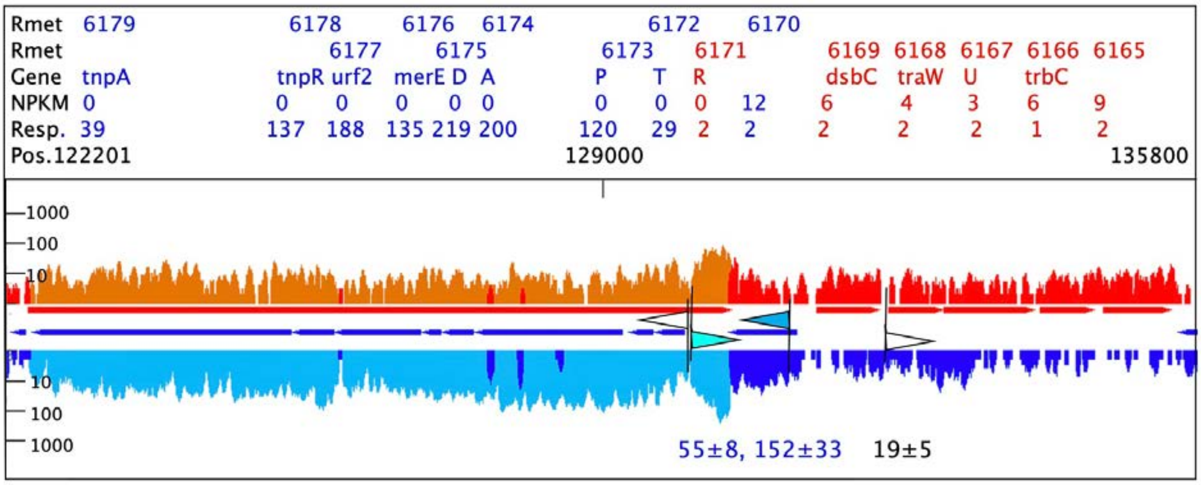

Panel Z. The *mer* determinant on plasmid pMOL30, part 2. Transcripts of this part of the *mer* determinant on plasmid pMOL30 could not be assigned to this region due to the high degree of homology between this and the *mer* determinant on pMOL28.

Literature for this figure.

1. Große C, Kohl T, Herzberg M, Nies DH. 2022. Loss of mobile genomic islands in metal resistant, hydrogen-oxidizing *Cupriavidus metallidurans*. Appl Environ Microbiol 88:e02048-21.
2. Schulz V, Schmidt-Vogler C, Strohmeyer P, Weber S, Kleemann D, Nies DH, Herzberg M. 2021. Behind the shield of Czc: ZntR controls expression of the gene for the zinc-exporting P-type ATPase ZntA in *Cupriavidus metallidurans*. J Bacteriol 203:e00052-21.
3. Scherer J, Nies DH. 2009. CzcP is a novel efflux system contributing to transition metal resistance in *Cupriavidus metallidurans* CH34. Mol Microbiol 73:601-621.
4. Borremans B, Hobman JL, Provoost A, Brown NL, Van der Lelie D. 2001. Cloning and functional analysis of the *pbr* lead resistance determinant of *Ralstonia metallidurans* CH34. J Bacteriol 183:5651-5658.
5. Taghavi S, Lesaulnier C, Monchy S, Wattiez R, Mergeay M, van der Lelie D. 2009. Lead(II) resistance in *Cupriavidus metallidurans* CH34: interplay between plasmid and chromosomally-located functions. Antonie Van Leeuwenhoek 96:171-182.
6. Debut AJ, Dumay QC, Barabote RD, Saier MH. 2006. The iron/lead transporter superfamily of Fe^{3+}/Pb^{2+} uptake systems. J Mol Microbiol Biotechnol 11:1-9.
7. Monchy S, Benotmane MA, Wattiez R, van Aelst S, Auquier V, Borremans B, Mergeay M, Taghavi S, van der Lelie D, Vallaeys T. 2006. Transcriptomics and proteomic analysis of the pMOL30-encoded copper resistance in *Cupriavidus metallidurans* strain CH34. Microbiology 152:1765-1776.
8. Wiesemann N, Bütof L, Herzberg M, Hause G, Berthold L, Etschmann B, Brugger J, Martinéz-Criado G, Dobritzsch D, Baginski S, Reith F, Nies DH. 2017. Synergistic toxicity of copper and gold compounds in *Cupriavidus metallidurans* Appl Environ Microbiol 83:e01679-17.
9. Jian X, Wasinger EC, Lockard JV, Chen LX, He C. 2009. Highly sensitive and selective gold(I) recognition by a metalloregulator in *Ralstonia metallidurans*. J Am Chem Soc 131:10869-10871.
10. Juhnke S, Peitzsch N, Hübener N, Große C, Nies DH. 2002. New genes involved in chromate resistance in *Ralstonia metallidurans* strain CH34. Arch Microbiol 179:15-25.
11. Nies DH, Koch S, Wachi S, Peitzsch N, Saier MHJ. 1998. CHR, a novel family of prokaryotic proton motive force-driven transporters probably containing chromate/sulfate transporters. J Bacteriol 180:5799-5802.
12. Nies A, Nies DH, Silver S. 1990. Nucleotide sequence and expression of a plasmid-encoded chromate resistance determinant from *Alcaligenes eutrophus*. J Biol Chem 265:5648-5653.
13. Yamamoto H, Wittek D, Gupta R, Qin B, Ueda T, Krause R, Yamamoto K, Albrecht R, Pech M, Nierhaus KH. 2016. 70S-scanning initiation is a novel and frequent initiation mode of ribosomal translation in bacteria. Proc Natl Acad Sci U S A 113:E1180-E1189.

Supplementary Figure S6. Maps of chromosomal genetic determinants encoding genes required for autotrophic growths. Maps are shown of determinants in the indicated regions with NPKM values on one DNA strand (red) or the other direction of transcription (blue). Values in orange and light blue are transcripts resulting from multiple homologous DNA regions, which could not be associated to a single locus. Above are the Rmet locus and gene names, the mean NPKM and response values (3). TSSs (flags) are indicated with the corresponding TSS score, white for scores < 50 that were not assigned to promoter consensus motifs, red shades for strong (>1000, red), medium (100-1000, orange) or weak (50-100, light orange) RpoD promoters, blue shades (strong, medium, light) according if not associated to the RpoD model. The region for the soluble hydrogenase was annotated by TraV-Mac as integrative element as one long red bar, so that the positions of the open reading frames were added manually for one direction of transcription (green arrows).

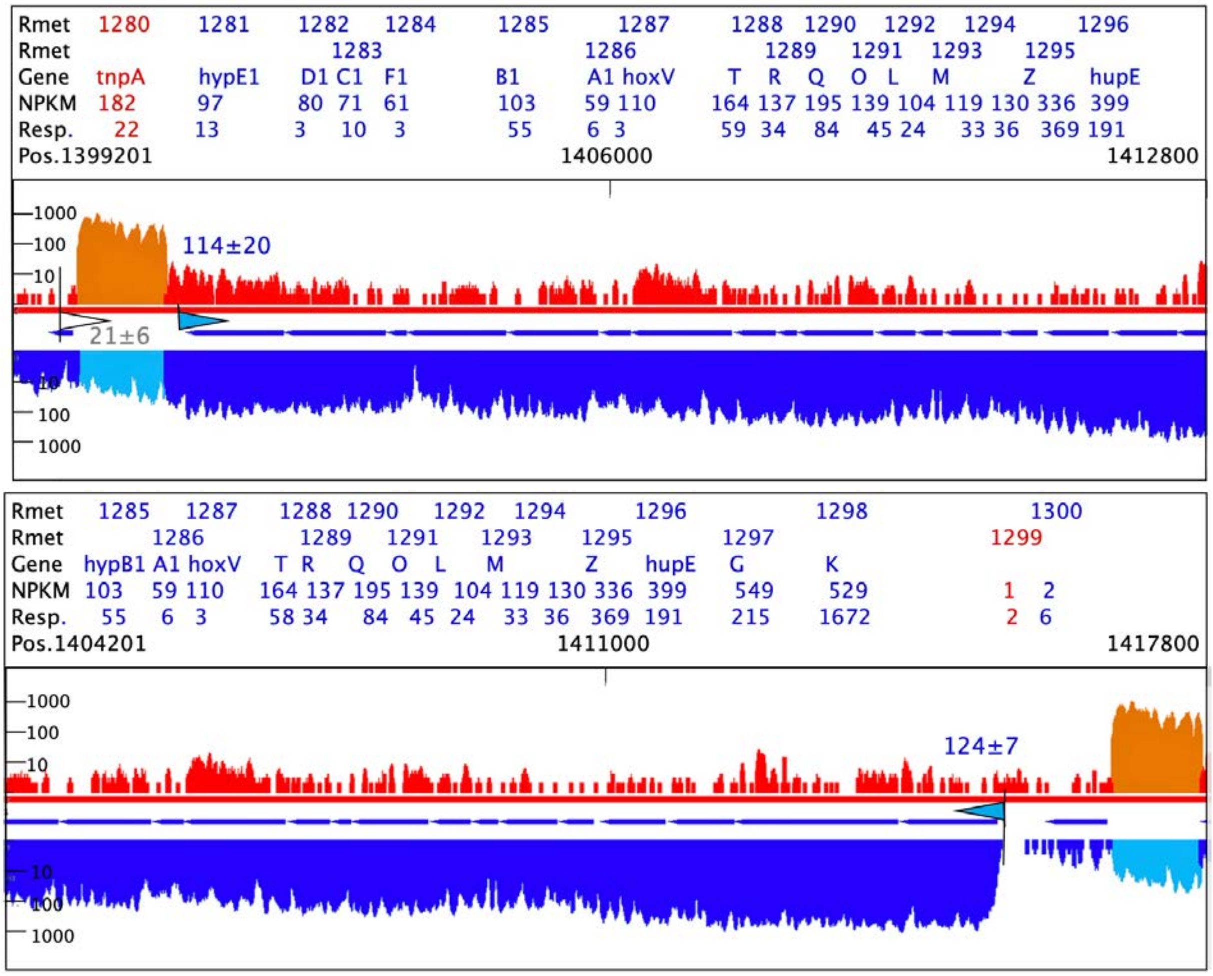

Panels A (top) and B (bottom). The predicted operon Op0370r_1 on chromosomal island CMGI-3 encoding the membrane-bound hydrogenase. The genes for synthesis of a membrane-bound hydrogenase are all in one large multicistronic operon Op0370r_1 and transcribed from one sufficiently strong TSS that could not be associated to RpoD (Panel B). After 28 genes were transcribed in this operon, transcription may even continue across an inserted transposon (Panel A), indicating the ability of the *C. metallidurans* RNA polymerase to transcribe more than 18.5 kb of DNA. The responsible TSS was 56 bp upstream of *hoxK*, the first gene of this large operon. The pattern recognition software identified a 4,1 model upstream of this TSS with a GCCCGACGC at position -41 and a CGTACATTGT at -13 with the highly conserved second “A” and last “T” of the typical TATAAT -35 site conserved (Suppl. Table S1). The RpoD score was -4.6 so that a sigma factor different from RpoD, which uses nevertheless a -10 site similar to that of RpoD, may be responsible for expression of this large operon. A non-RpoD-dependent promoter may initiate antisense-transcription of the operon.

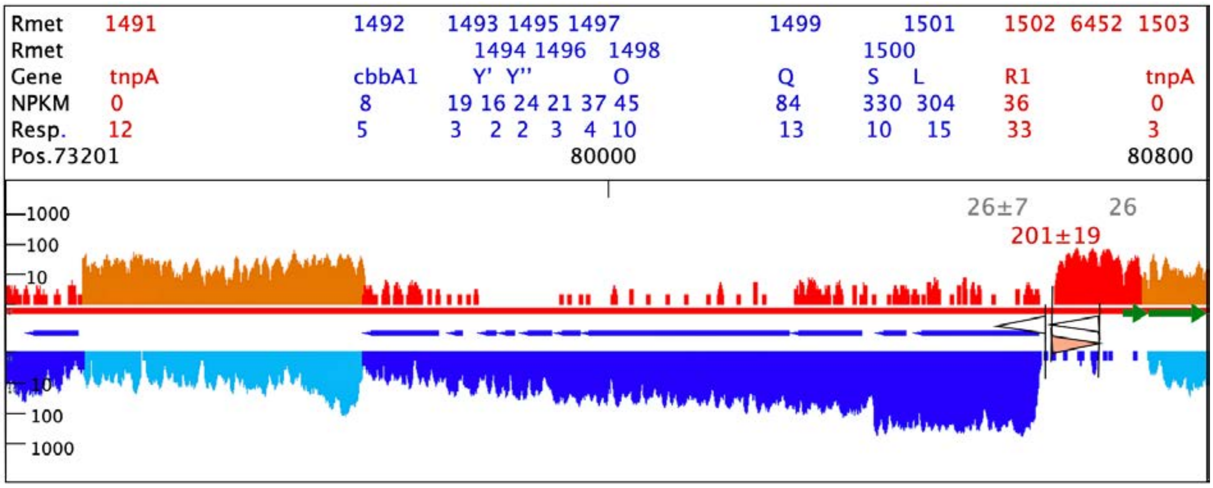

Panel C. The region on chromosomal island CMGI-2 encoding the soluble hydrogenase, part 1. Arrangement of the genes for the Calvin cycle enzymes and a soluble, NADH-reducing hydrogenase was far more complicated than that of the genes for the membrane-bound hydrogenase. These genes were arranged in six operon regions Op0422r to Op0427f (Panels C to H). Transposon insertions flank this gene cluster, which seem to have been integrated into a *ccbA* gene for a fructose-1,6-bisphosphate aldolase, with the *cbbA1* and a *cbbA*Δ gene fragment still located at the flanks of the cluster. Additional transposon insertions re-arranged Op0423f and Op0424r, and interrupted the *pntAA* gene to separate the regions Op0425f and Op0427f from each other. The operon Op0422r_1 *cbbLSQOY* for the ribulose-bis-phosphate carboxylase and other enzymes was expressed and the weakly scoring TSSs (26±7) was under control of a medium-strong RpoD promoter, which was not strong enough to explain the NPKM value of the first gene, *cbbL*. A RpoD-associated TSS initiated transcription of *cbbR1* for a regulatory protein in operon Op0423f_1, 1 bp downstream of the start codon of this gene, indicating again lmRNA-type translation initiation (1).

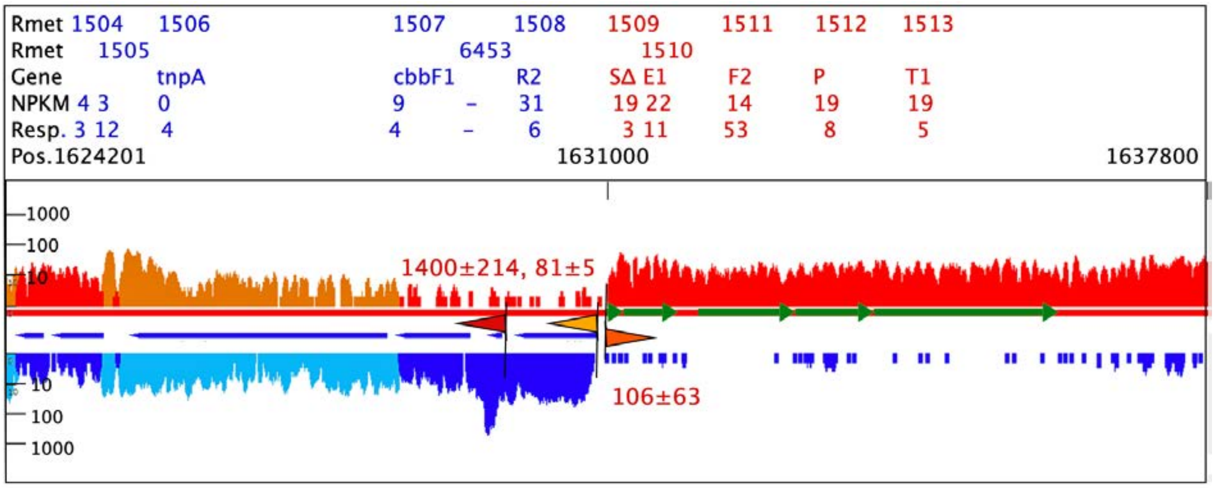

Panel D. The region encoding the soluble hydrogenase, part 2. A strong, RpoD-dependent promoters upstream of *cbbF1* may be responsible for transcription events than continue into the *cbbLSQOY* region (Panel C) and may have been followed by differential mRNA stability concerning the middle part of the transcript (2). A weakly scoring TSS associated to RpoD initiate transcription of Op0424r_1 with *cbbR2* for a regulatory protein and Op0424r_2/3 with *cbbF1* and the transposase. The gene expression levels downstream of the strong RpoD-dependent promoter did not match the TSS score of this promoter. This indicated that these promoters may be indeed responsible for expression of *cbbLS* (Panel C), in combination with differential mRNA stability. A TSS associated with RpoD was responsible for transcription initiation of Op0425f_1 *cbbS*Δ*E1F2PT1Z1G1-pgk1-cbbYA2-pykA2JiI1* containing genes for additional Calvin cycle enzymes (continues in Panel E).

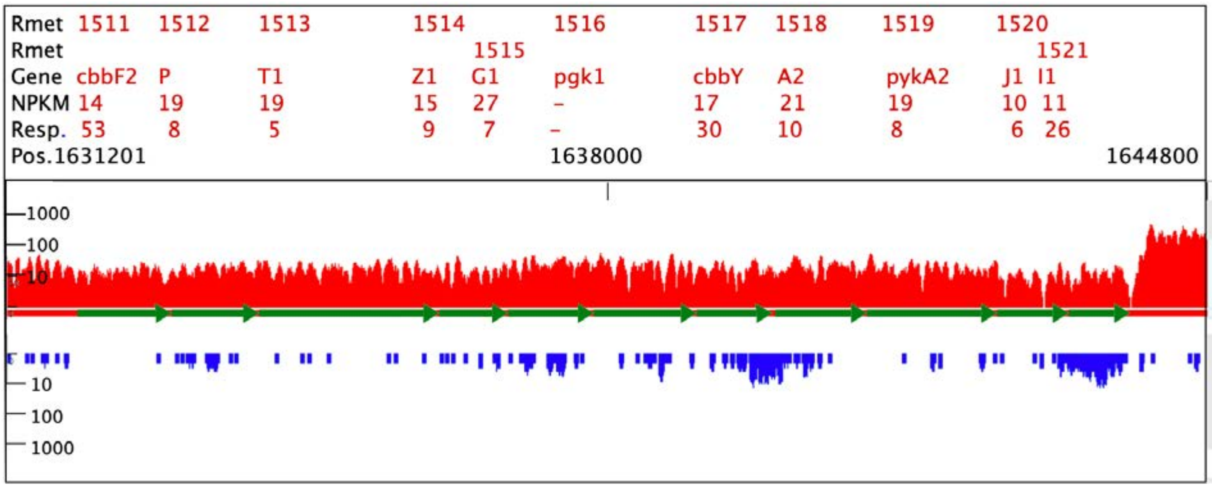

Panel E. The region encoding the soluble hydrogenase, part 3. A TSS associated with RpoD (Panel C) was responsible for transcription initiation of Op0425f_1 *cbbS*Δ*E1F2PT1Z1G1-pgk1-cbbYA2-pykA2JiI1* containing genes for additional Calvin cycle enzymes and Op0425f_2 containing *hoxFUYHWI* genes for hydrogenase maturation and synthesis (continues in Panel F).

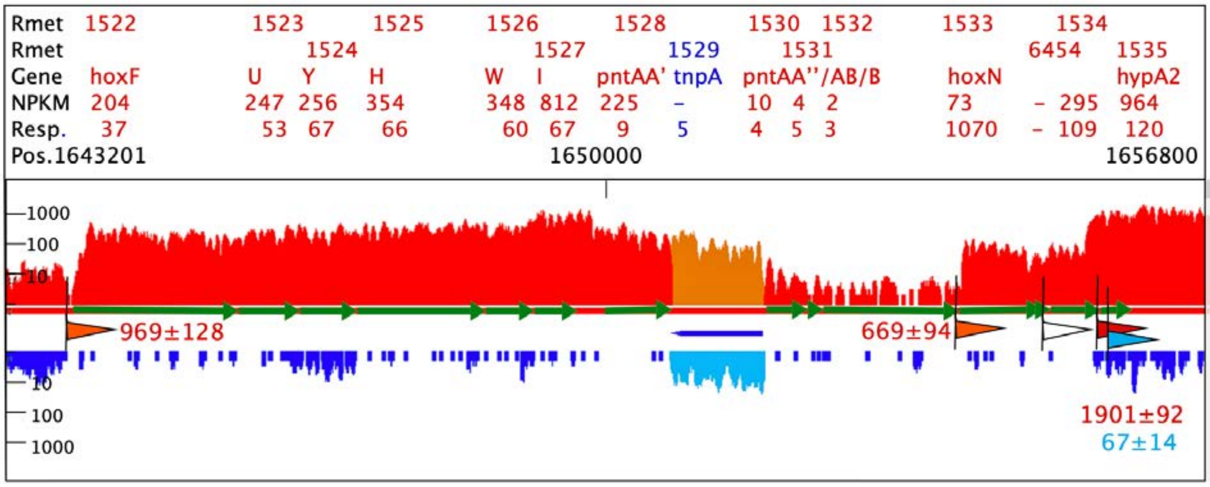

Panel F. The region encoding the soluble hydrogenase, part 4. A TSS associated with RpoD (Panel C) was responsible for transcription initiation of Op0425f_1 *cbbS*Δ*E1F2PT1Z1G1-pgk1-cbbYA2-pykA2JiI1* containing genes for additional Calvin cycle enzymes and Op0425f_2 containing *hoxFUYHWI* genes for hydrogenase maturation and synthesis.

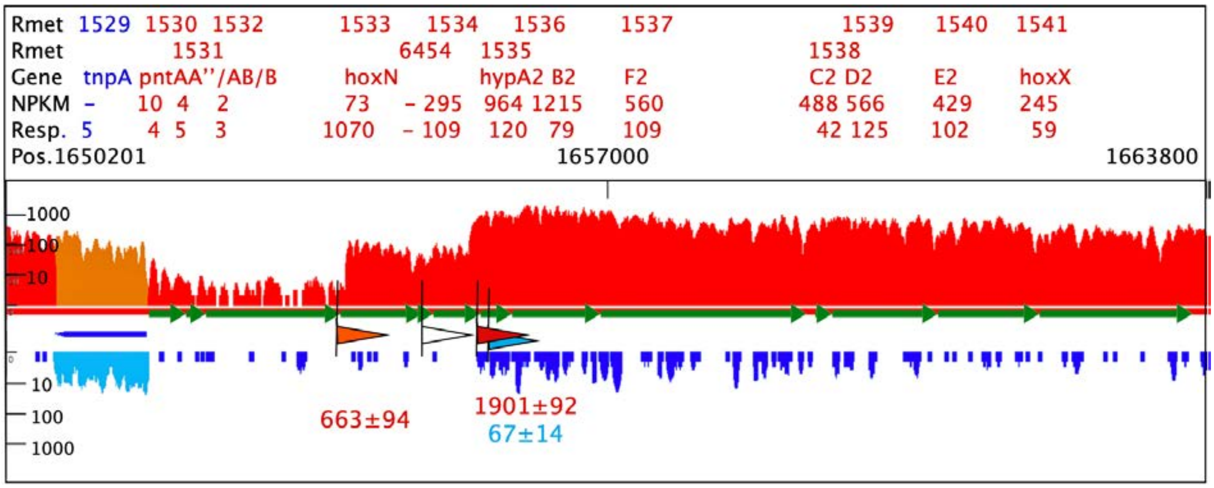

Panel G. The region encoding the soluble hydrogenase, part 5. A putative ancestral operon Op0425f-Op0427f seems to have been interrupted by transposon insertion into a *pntAA* gene

for an alanine dehydrogenase. The transposase gene is now region Op0426r. The *pntAA''/AB/B* genes for the remaining components of the dehydrogenase were on a low expression level in operon Op0427f_1 (Panel F). One TSS associated to RpoD initiated transcription of *hoxN* for a nickel importer in Op0427f_2 on the same DNA strand.

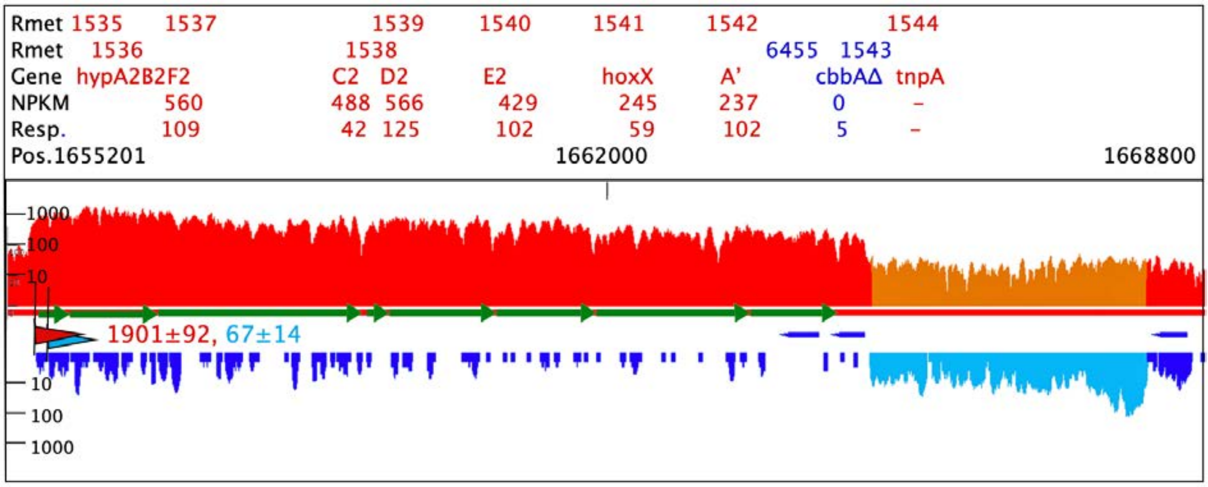

Panel H. The region encoding the soluble hydrogenase, part 6. A very strong and RpoD-associated TSS initiated transcription of the main structural genes *hypA2B2F2C2D2E2XA'* for the soluble hydrogenase on Op0427f_4.

Literature for this figure.

1. Yamamoto H, Wittek D, Gupta R, Qin B, Ueda T, Krause R, Yamamoto K, Albrecht R, Pech M, Nierhaus KH. 2016. 70S-scanning initiation is a novel and frequent initiation mode of ribosomal translation in bacteria. Proc Natl Acad Sci U S A 113:E1180-E1189.
2. Owolabi JB, Rosen BP. 1990. Differential mRNA stability controls relative gene expression within the plasmid-encoded arsenical resistance operon. J Bacteriol 172:2367 2371.

Supplementary Table S1. Transcriptional start points in the transcriptome of TMM-grown *Cupriavidus metallidurans* CH34 cells. The TSS were indexed according to the mean value of the TSS determination score, which is indicated (Mean) with the accompanying deviation (SDEV) of three biological repeats. The TSS name gives the position in the replicon, the direction (-/+) and the replicon number (last digit X of the data base number "CP00035X"). The upper 3000 TSSs were used in a hidden Markov-model for motif discovery for possible -10 motifs and sorted into the initial component groups (Comp_0) 0,1 to 0,3. The motifs were at the positions "-10@0" upstream of the transcriptional start point +1. The "DistFlag0" indicates, if this position could be at the position of a usual -10 region between -10 and -18 bp upstream. Hidden Markov-modelling was continued for all "0,1" components, leading to the discovery of putative promoter regions, indicated with the position, sequence, score and LRR value. All TSS were subsequently screened with the "0,1" model. Next, a score for a RpoD-dependent promoter was developed and used. The score indicates a possible strong (s), medium (m), weak (w) or no RpoD-dependent promoter. Light field and s/m/w/no RpoD promoters in italic indicate wrong positions of the -10 and -35 region (sba, sliding-blocking-anotherSigma; ba, blocking-anotherSigma; nu, TSS not used by RpoD). Last, the distance to the next open reading frame downstream of the TSS is indicated with the locus tag, a gene name, if available, the predicted operon of this gene and the KEGG orthology to the third level. Rmet on a blue field: horizontally acquired gene.

Ind	TSS_Name	Mean	SDEV	Comp_0	-10@0	DistFlag0	St-35	Sequence-35	St-10	Sequence-10	Score	comp	LRR	RpoD_score	Value	Dist to	Locus	Gene	Operon	KO
1	TSS_769713-2	29914	8216	0,3	-22	0	-34	TTGCCCCTGG	-12	TCTTTGTTGA	-149	1,1	0.88	9.20	s	42	Rmet_0697	*pilA*	Op0196r_2	GIP-PTL-T2S
2	TSS_436828+2	20158	1746	0,1	-17	1	-35	TTGCAACGGC	-15	TGTATAATTC	-141	1,1	11.86	12.60	s	14	Rmet_6415	*0*	Op0113f_1	NA
3	TSS_3511528-2	18541	3109	0,1	-16	1	-48	GGCGAAAAAG	-14	CACATAATAC	-144	4,1	8.10	-16.60	*no*	14	Rmet_3234	*0*	Op0918r_4	NA
4	TSS_3547712-2	15836	3055	0,1	-17	1	-36	TTGCCAGCGC	-15	GTAAGAATCG	-142	1,1	7.74	12.80	s	90	Rmet_3272	*pilM*	Op0920r_2	GIP-PTL-T2S
5	TSS_1049887-2	15310	1723	0,1	-15	1	-38	TTGTCGTGAT	-13	TTTAGCCTCG	-142	1,1	6.68	6.40	s	22	Rmet_0965	*0*	Op0268r_1	NA
6	TSS_2579185+3	14841	1967	0,3	-4	0	-37	TTGACGGGAG	-16	CGTATAAATC	-143	1,1	5.91	11.80	s	141	Rmet_5816	*cspA*	Op1756f_1	NA
7	TSS_3120631+2	14701	3240	0,1	-15	1	-35	TTGATTCGTC	-13	TCTATAATGT	-138	1,1	10.36	14.20	s	94	Rmet_2870	*rpmB*	Op0807f_1	GIP-TL-Ribo
8	TSS_2929477-2	14585	1748	0,1	-15	1	-34	TTGACGGGTT	-13	CCTACAATCC	-142	1,1	10.65	12.60	s	344	Rmet_2697	*0*	Op0760r_1	NA
9	TSS_996670+2	13541	3208	0,1	-17	1	-43	TTGCCCATAG	-15	AGTATCATCG	-139	1,1	9.25	0.40	*(w)-sba*	136	Rmet_0911	*ilvI*	Op0263f_2	MET-CAH-But
10	TSS_2334087-2	12911	2247	0,1	-15	1	-35	TTGACATGTC	-13	TTTAAGATCG	-143	1,1	9.52	14.20	s	187	Rmet_2142	*0*	Op0618r_2	NA
11	TSS_3612514-2	12908	2431	0,1	-16	1	-35	TTGACAGCCA	-14	ACCATAATCA	-141	1,1	8.50	14.60	s	7	Rmet_R0059	*0*	Op0930r_2	NA
12	TSS_1071434-3	12866	1637	0,1	-15	1	-36	TATTGTTGCG	-13	GGTAAAATGC	-139	2,1	14.45	8.40	s	231	Rmet_4472	*sulP*	Op1275r_1	NA
13	TSS_1221419+3	12862	1850	0,1	-16	1	-37	TATTGTTGCG	-14	GGTAAAATGC	-139	2,1	14.45	7.40	s	230	Rmet_4600	*sulP*	Op1324f_1	NA
14	TSS_2323167+2	12552	2680	0,1	-17	1	-35	TGGCGCACCG	-15	GCTATGATGC	-136	1,1	8.67	9.10	s	81	Rmet_2135	*rho*	Op0615f_1	GIP-TK-Assoc
15	TSS_277708+2	12521	4061	0,1	-16	1	-35	TTGTTCAGGT	-14	CATATAATGC	-140	1,1	9.82	12.60	s	109	Rmet_0260	*0*	Op0075f_2	NA
16	TSS_2559227-2	12081	778	0,1	-16	1	-35	TTGCGTTTTT	-14	AATATGATCG	-141	1,1	8.61	11.60	s	27	Rmet_2344	*0*	Op0662r_1	NA
17	TSS_3329116-2	11351	1297	0,1	-16	1	-48	TTGCACTTTT	-14	ATTATGATTC	-144	1,1	9.55	-11.60	*no*	128	Rmet_3072	*0*	Op0862r_2	NA
18	TSS_825489+2	11075	879	0,1	-16	1	-36	TTTGTTTGGG	-14	GCTATACTCT	-137	2,1	13.09	10.90	s	348	Rmet_0749	*rimM*	Op0213f_1	GIP-TL-Ass
19	TSS_3792080-2	10948	1921	0,1	-15	1	-35	TTGACTGATG	-13	AATAGAATCG	-143	1,1	9.03	14.20	s	56	Rmet_3501	*atpI*	Op0970r_1	MET-EN-OxPhos
20	TSS_2637887-2	10000	1532	0,1	-15	1	-44	TCGGCAGGCG	-13	GCTAAAATGC	-141	5,1	12.93	-4.10	*no*	30	Rmet_2427	*acpP*	Op0680r_3	MET-LIP-FASyn
21	TSS_1581484+2	9734	1921	0,1	-14	1	-35	CGCATCAGCA	-12	TGTATAATCT	-146	1,1	3.25	7.20	s	21	Rmet_1461	*guaB*	Op0409f_1	MET-NUC-Pur
22	TSS_1094334-3	9705	731	0,1	-15	1	-45	TCGCCTCTTT	-13	GCTATACTTA	-145	1,1	8.55	-7.10	*no*	116	Rmet_4494	*0*	Op1279r_1	NA
23	TSS_2575204-2	9391	302	0,1	-28	0	-35	TTGACAAAAG	-14	ACTAGAGTGA	-141	1,1	7.96	13.60	s	26	Rmet_2363	*0*	Op0664r_1	NA
24	TSS_195952+2	9040	1634	0,1	-16	1	-35	TTGACATAGG	-14	TCCACTATTT	-141	1,1	7.62	12.60	s	213	Rmet_0188	*0*	Op0057f_1	NA
25	TSS_3806993-2	8904	1890	0,1	-15	1	-42	TTGCTGACTT	-13	GTTACATTCC	-142	1,1	8.53	-1.60	*no*	232	Rmet_3515	*0*	Op0974r_5	NA
26	TSS_2406231-2	8646	1226	0,1	-15	1	-34	TTGAGAAATT	-13	GATATAATTC	-138	1,1	12.16	13.60	s	217	Rmet_2195	*carA*	Op0630r_3	MET-NUC-Pyr
27	TSS_1563989+2	8088	1062	0,1	-16	1	-41	TTCCGGTTCT	-14	TGTATTATTG	-143	1,1	8.12	0.90	*(w)-sba*	210	Rmet_1443	*yaeT*	Op0403f_3	NA
28	TSS_137004-5	8047	964	0,1	-15	1	-34	TTGCCAGGCC	-13	TGTAGAGTTA	-140	1,1	8.00	11.60	s	250	Rmet_6281	*0*	Op1915r_2	NA
29	TSS_2205326-2	8015	1211	0,1	-15	1	-35	TTGCAGGGCG	-13	CATACAATCG	-139	1,1	9.56	12.20	s	501	Rmet_2035	*0*	Op0594r_2	DIV-Division
30	TSS_3235249+2	7990	1481	0,1	-16	1	-45	TGGCACCGGG	-14	CTTATGATTC	-140	1,1	7.91	-7.10	*no*	31	Rmet_2976	*fur*	Op0837f_1	NA
31	TSS_2325559+2	7965	1233	0,1	-15	1	-35	TTGCACCTGC	-13	GCCATAATCC	-140	1,1	10.02	12.20	s	92	Rmet_2137	*0*	Op0615f_2	GIP-TL-Ribo
32	TSS_1775451-2	7915	997	0,1	-17	1	-40	CCGGCCGGAT	-15	CATACAATCC	-140	5,1	11.61	1.90	*(w)-sba*	465	Rmet_1641	*0*	Op0462r_3	NA
33	TSS_3365432-2	7225	1186	0,1	-16	1	-35	TTGCAGTTTT	-14	TTTATAATCA	-139	1,1	10.84	12.60	s	111	Rmet_3106	*rplU*	Op0874r_1	GIP-TL-Ribo
34	TSS_90669-5	7214	876	0,1	-16	1	-35	TTGCCTGTTA	-14	GGTATTCTTC	-140	1,1	8.67	11.60	s	119	Rmet_6241	*parB2*	Op1901r_1	NA
35	TSS_727772-3	6959	1110	0,1	-15	1	-33	TTGACAGCCA	-13	GTCAAAATGG	-140	1,1	9.43	10.60	s	178	Rmet_4152	*tnp*	Op1169r_1	NA
36	TSS_2787446+2	6818	1157	0,1	-17	1	-37	GCAACAAAAT	-15	GCTATACTCC	-140	4,1	11.92	8.40	s	372	Rmet_2564	*rhlE2*	Op0709f_2	NA
37	TSS_2201889-2	6808	1244	0,1	-15	1	-34	TTGCCTTTTG	-13	CGTATAATCA	-139	1,1	10.40	12.60	s	225	Rmet_2033	*0*	Op0594r_2	NA
38	TSS_3158328-2	6332	1043	0,1	-16	1	-36	TTGCCTGTCC	-14	TGTATAATGC	-135	1,1	10.90	14.40	s	125	Rmet_2904	*rpsT*	Op0820r_1	GIP-TL-Ribo
39	TSS_83384-5	6326	1015	0,1	-15	1	-35	TTGCTGAAAG	-13	GCTAAGATTG	-138	1,1	9.14	11.20	s	121	Rmet_6233	*slyB*	Op1901r_3	NA
40	TSS_2422112+2	6239	1290	0,1	-16	1	-47	TCGAAAAAGG	-14	GGCATAATCT	-141	1,1	8.97	-9.10	*no*	42	Rmet_R0035	*0*	Op0631f_3	NA
41	TSS_3561109-2	5945	703	0,1	-15	1	-44	CTGATCTAAG	-13	TGTACAATAA	-145	1,1	4.75	-6.60	*no*	212	Rmet_3284	*0*	Op0924r_1	NA
42	TSS_3157128-2	5923	1482	0,1	-15	1	-34	TCGCCAAAGC	-13	CTTATGATTC	-138	1,1	8.51	11.10	s	114	Rmet_2902	*argF*	Op0818r_1	NA
43	TSS_972109-2	5676	1297	0,1	-16	1	-35	TGGACTTCGG	-14	GCTAAAATGC	-138	1,1	10.16	12.10	s	95	Rmet_0887	*0*	Op0258r_3	NA
44	TSS_2642334-2	5575	525	0,1	-15	1	-30	TTGCGCAGGA	-5	CGCAAAATTC	-139	1,1	8.64	3.20	*(w)-nu*	18	Rmet_2432	*rpmF*	Op0680r_2	GIP-TL-Ribo
45	TSS_1114672+2	5538	642	0,1	-16	1	-36	TTGACTGATT	-14	CTTATACTCG	-141	1,1	9.52	14.40	s	28	Rmet_1024	*iscR*	Op0295f_3	NA
46	TSS_2655559+2	5490	704	0,1	-16	1	-42	GGCAGACTAG	-14	GCTACACTTG	-139	4,1	8.48	-4.60	*no*	36	Rmet_2444	*0*	Op0683f_2	NA
47	TSS_93740-5	5404	782	0,1	-15	1	-34	TTGTAGGTTG	-13	GGTAAGATTC	-137	1,1	8.80	9.60	s	83	Rmet_6245	*0*	Op1903r_2	NA
48	TSS_318921-2	5148	1390	0,1	-15	1	-48	TCGCGATGCG	-13	GATACACTGG	-141	1,1	8.40	-14.10	*no*	354	Rmet_0302	*rpoX*	Op0086r_2	GIP-TL-Ass
49	TSS_2153993-2	5129	797	0,1	-15	1	-35	TTGCGGGAAG	-13	GCTATACTCG	-135	1,1	10.10	12.20	s	60	Rmet_1979	*rpsF*	Op0576r_2	GIP-TL-Ribo
50	TSS_138295-4	4924	612	0,1	-15	1	-33	TTGAATCTTG	-13	TGTAGAATCA	-144	1,1	9.35	9.60	s	85	Rmet_6162	*0*	Op1884r_1	NA
51	TSS_942026+2	4856	1101	0,1	-17	1	-36	TTGCGATGCT	-15	GTCATAATCT	-139	1,1	10.00	12.80	s	5	Rmet_R0010	*0*	Op0247f_3	NA
52	TSS_3308560+2	4682	1248	0,1	-17	1	-42	TTCACTTAAC	-15	GCTACACTCG	-137	1,1	8.97	0.90	*(w)-sba*	452	Rmet_3052	*corA*	Op0859f_1	EIP-TRA-Ion

53	TSS_798805+2	4603	1306	0,1	-15	1	-34	TTGATTGATA	-13	GCTAATATCG	-141	1,1	8.27	10.60	s	300	Rmet_0722	*rpsA*	Op0205f_2	GIP-TL-Ribo
54	TSS_439702+2	4518	402	0,1	-16	1	-36	TCGGCACGAT	-14	CTTAGAATAC	-139	5,1	10.76	12.90	s	20	Rmet_0414	*erpA*	Op0113f_3	NA
55	TSS_3402278-2	4468	897	0,1	-16	1	-35	TTGACCCGCC	-14	TCTAAAGTGG	-143	1,1	8.48	12.60	s	108	Rmet_3137	*mraZ*	Op0878r_2	NA
56	TSS_1764239-3	4390	927	0,1	-17	1	-36	TTTACTTCAT	-15	GCTAGCATCC	-149	1,1	2.40	11.30	s	322	Rmet_5075	*msuE1*	Op1493r_1	NA
57	TSS_437144+2	4385	822	0,1	-17	1	-41	TTGCTTGTGC	-15	CGTATAATGG	-138	1,1	10.45	4.40	*(m)-sba*	142	Rmet_0410	*rplM*	Op0113f_2	GIP-TL-Ribo
58	TSS_785879-2	4284	769	0,1	-15	1	-45	GGAACCGAAC	-13	AGTACAATCC	-140	4,1	10.28	-10.60	*no*	23	Rmet_R0007	*ssrA*	Op0202r_2	NA
59	TSS_2145829+2	4253	1046	0,1	-17	1	-36	TTGCCAAACC	-15	GGTAGATTGG	-139	1,1	9.55	12.80	s	251	Rmet_1970	*0*	Op0575f_2	NA
60	TSS_3576368-2	4225	999	0,1	-16	1	-34	TTGCAAGTCC	-14	GCTATAATCC	-138	1,1	12.03	11.60	s	221	Rmet_3307	*rplN*	Op0926r_2	GIP-TL-Ribo
61	TSS_427162+2	4178	553	0,1	-15	1	-34	TTGTGCTGGG	-13	GCAATACTGT	-141	1,1	6.64	9.60	s	54	Rmet_0399	*gltI*	Op0109f_4	EIP-TRA-ABC
62	TSS_1759981+3	4112	325	0,1	-16	1	-36	TTGCGGCAGA	-14	TGTATATTTG	-139	1,1	8.87	12.40	s	69	Rmet_5071	*0*	Op1492f_3	NA
63	TSS_1055687-3	4079	257	0,1	-15	1	-49	TCAGCACGCG	-13	CCTATGCTTG	-139	5,1	8.84	-16.60	*no*	3270	Rmet_4455	*0*	Op1267r_1	NA
64	TSS_875216+2	4045	845	0,1	-16	1	-43	TCGCGAAGAT	-14	TGTATAATAG	-141	1,1	9.67	-1.10	*no*	401	Rmet_0799	*0*	Op0227f_1	EIP-TRA-ABC
65	TSS_1072760+2	4033	678	0,1	-16	1	-36	TGGACTGGCG	-14	GGTACAATGC	-135	1,1	10.12	12.90	s	227	Rmet_0987	*0*	Op0279f_1	NA
66	TSS_3130198+2	3923	187	0,1	-17	1	-36	TTGTGACGCG	-15	GTTAGAGTGG	-138	1,1	7.47	11.80	s	119	Rmet_2879	*tnpA*	Op0809f_1	NA
67	TSS_3832600+2	3852	400	0,1	-17	1	-35	GGCAGACAAC	-15	TGTACAATCA	-137	4,1	10.20	6.60	s	31	Rmet_3535	*phhR*	Op0977f_3	NA
68	TSS_1276983+2	3807	801	0,1	-19	0	-35	TTGCGTTCCG	-14	AAGATACTGG	-139	2,1	9.45	10.60	s	170	Rmet_1166	*ihfA*	Op0333f_3	GIP-REP-Recomb
69	TSS_1002655+2	3705	453	0,1	-17	1	-36	TTGCACACAC	-15	GATATAGTCG	-139	1,1	9.14	11.80	s	243	Rmet_0918	*leuA*	Op0263f_4	MET-CAH-Pyr
70	TSS_16388+5	3702	544	0,1	-16	1	-38	TGGAGGGTAG	-14	GCTAGCATTC	-141	1,1	7.67	6.90	s	142	Rmet_6327	*0*	Op1922f_1	NA
71	TSS_2635570-2	3632	482	0,1	-24	0	-25	TTGCGGACGG	-2	GATATCAATA	-143	1,1	3.72	-0.80	*no*	388	Rmet_2424	*rseA*	Op0680r_4	NA
72	TSS_2206187+2	3627	648	0,1	-16	1	-49	TGGCAACTGT	-14	TGTAAAATGC	-141	1,1	10.23	-14.10	*no*	68	Rmet_2037	*fnrL*	Op0595f_1	NA
73	TSS_2340046-2	3627	702	0,1	-15	1	-36	TGGCTGTTGG	-13	TGTAAAATTG	-140	1,1	9.07	9.90	s	1966	Rmet_2145	*yejB*	Op0618r_1	NA
74	TSS_225245+3	3605	287	0,1	-7	0	-35	TGGCATACGA	-14	GATATAAACG	-146	1,1	4.52	9.10	s	181	Rmet_3685	*flhD2*	Op1026f_3	EIP-SIG-2Comp
75	TSS_3784022+2	3586	128	0,1	-16	1	-41	TTGTTACTGC	-14	CCCACAATGC	-143	1,1	7.77	2.40	*(w)-sba*	8149	Rmet_3502	*0*	Op0971f_1	NA
76	TSS_538405+3	3584	299	0,1	-16	1	-34	TTGCATTTTC	-14	GTCACACTTG	-140	1,1	8.19	7.60	s	44	Rmet_3974	*0*	Op1114f_1	NA
77	TSS_3633707+2	3581	221	0,1	-17	1	-36	TTGCTTTAAT	-15	GCTAAGATGC	-141	1,1	9.49	10.80	s	144	Rmet_3358	*0*	Op0935f_3	NA
78	TSS_3371920+2	3554	622	0,2	-16	1	-35	TTGTCCTCGG	-14	CATAGAATGC	-137	1,1	8.95	12.60	s	154	Rmet_3112	*0*	Op0875f_3	NA
79	TSS_424374-2	3540	575	0,1	-16	1	-40	TTGCATCATT	-14	CCTATGATGG	-142	1,1	9.25	4.40	*(m)-sba*	25	Rmet_0396	*0*	Op0108r_1	NA
80	TSS_3192126-2	3510	359	0,1	-16	1	-36	TTGCAAGTTG	-14	GACATAATCC	-138	1,1	9.76	13.40	s	53	Rmet_2936	*pilT*	Op0828r_1	GIP-PTL-T2S
81	TSS_1094302+3	3464	586	0,1	-17	1	-43	GCAACGGTTT	-15	CCTACACTTC	-145	4,1	6.15	-5.60	*no*	2	Rmet_4495	*0*	Op1280f_1	NA
82	TSS_499961-3	3464	581	0,1	-15	1	-45	TTGAGCATTA	-13	ATTAAGATTT	-146	1,1	8.21	-6.60	*no*	405	Rmet_3940	*0*	Op1101r_2	NA
83	TSS_928+3	3457	250	0,2	-17	1	-42	TTCTGCGTGA	-15	CGTACAAAGG	-147	1,1	2.83	-2.10	*no*	17	Rmet_5817	*repA*	Op1756f_2	NA
84	TSS_3320089+2	3447	337	0,1	-26	0	-49	TTGTATCCCA	-24	TCTAAGCTAC	-144	1,1	5.65	-5.60	*no*	25	Rmet_3063	*aroQ*	Op0861f_3	MET-AA-PheTyrTrp
85	TSS_3297345-2	3426	338	0,1	-15	1	-21	AAAATCGTCG	-1	ATACGCGATC	-146	3,0	9.13	-15.40	*no*	37	Rmet_3041	*0*	Op0854r_1	NA
86	TSS_2053273-2	3424	619	0,1	-14	1	-35	TGCCGGAAGC	-12	GCGATAATCC	-139	1,1	6.98	8.20	s	104	Rmet_1886	*tig*	Op0544r_2	GIP-PTL-Fold
87	TSS_2503888-2	3423	909	0,1	-15	1	-40	TGGAATGCAT	-13	GATATACTTG	-138	1,1	8.89	2.90	*(w)-sba*	32	Rmet_2281	*0*	Op0652r_3	NA
88	TSS_3538454-2	3329	1236	0,1	-15	1	-35	TTGCAAGGGC	-13	TTTATAGTTG	-143	1,1	9.32	13.20	s	11	Rmet_3263	*gltB*	Op0920r_3	MET-EN-Nitrog
89	TSS_935510-2	3266	533	0,1	-17	1	-39	TTCTTTACAA	-15	CGGAGAATCC	-146	1,1	4.52	4.90	m	19	Rmet_0854	*0*	Op0244r_2	NA
90	TSS_1296632-2	3262	745	0,1	-15	1	-39	TCGCTAGGCG	-13	GGTACAGTAC	-143	1,1	6.63	3.90	m	61	Rmet_1183	*0*	Op0342r_2	NA
91	TSS_2332719+3	3254	605	0,1	-17	1	-39	TCCGCGCCGC	-15	GGCATAGTTG	-143	4,1	5.48	4.40	m	280	Rmet_5580	*dadA*	Op1660f_1	MET-EN-Nitrog
92	TSS_30051-5	3220	575	0,1	-15	1	-34	TGGCCGCCTG	-13	GATATGATCA	-141	1,1	7.66	10.10	s	392	Rmet_6340	*0*	Op1927r_2	NA
93	TSS_1075252-2	3139	393	0,1	-12	1	-34	TTGCCATCTT	-13	GATATAAAGA	-146	1,1	6.47	11.60	s	97	Rmet_6435	*0*	Op0280r_1	NA
94	TSS_3071672+2	3046	273	0,1	-16	1	-35	TTGACTTGTG	-14	GCGATACTTT	-141	1,1	8.25	12.60	s	289	Rmet_2824	*0*	Op0795f_3	NA
95	TSS_110631-3	3020	183	0,1	-15	1	-34	TTGAGACCTG	-13	TGTATATTTA	-142	1,1	8.53	12.60	s	25	Rmet_5910	*0*	Op1793r_2	NA
96	TSS_1145322+2	3008	583	0,1	-15	1	-49	TTCACAAATT	-13	TGTATAATCC	-136	1,1	10.88	-12.10	*no*	20	Rmet_1052	*pyrG*	Op0301f_2	MET-NUC-Pyr
97	TSS_648788-3	2954	377	0,1	0	0	-35	TTGAAGTCAG	-14	GCTAAGGTTC	-142	1,1	7.55	10.60	s	372	Rmet_4078	*oxlT*	Op1145r_1	NA
98	TSS_3011093+2	2953	1048	0,1	-16	1	-49	TCCACACGCA	-14	GCTAAAATAG	-138	1,1	7.94	-13.60	*no*	69	Rmet_2769	*metG*	Op0777f_1	MET-AA-Met
99	TSS_396571+2	2935	548	0,1	-12	1	-35	TGGAACAAGT	-10	CGAATACTTC	-143	1,1	6.24	7.70	s	109	Rmet_0372	*0*	Op0103f_4	NA
100	TSS_2978752-2	2927	302	0,1	-16	1	-39	TTACGGGAAG	-14	GGTAGAATGC	-141	4,1	8.78	4.90	m	553	Rmet_2742	*0*	Op0772r_2	GIP-REP-Recomb
101	TSS_3867632+2	2897	290	0,1	-16	1	-35	TTGCGGCGAC	-14	TGCAGAATCG	-137	1,1	8.04	10.60	s	182	Rmet_3566	*0*	Op0989f_1	NA
102	TSS_84558-5	2894	522	0,1	-15	1	-35	TTGCAATCGC	-13	GCTACTATGC	-138	1,1	10.17	12.20	s	109	Rmet_6234	*0*	Op1901r_2	NA
103	TSS_327445+2	2878	293	0,1	-18	1	-37	TTGCTCCCTT	-16	CGCATAATGC	-138	1,1	8.63	10.80	s	75	Rmet_0312	*0*	Op0089f_1	EIP-TRA-Ion
104	TSS_1012188+2	2868	740	0,1	-17	1	-36	TTGAACAAAT	-15	GTTAGAATGC	-141	1,1	11.42	12.80	s	105	Rmet_R0013	*0*	Op0267f_3	NA
105	TSS_210115-3	2842	392	0,1	-27	0	-49	GTAAAAAAAT	-25	CCGATAATCT	-147	4,1	9.77	-5.10	*no*	72	Rmet_3672	*0*	Op1021r_1	NA
106	TSS_1951509+3	2840	471	0,1	-17	1	-46	GCAGCCGCTC	-15	GTTACTCTCC	-146	5,1	7.70	-13.60	*no*	257	Rmet_5251	*0*	Op1550f_1	NA
107	TSS_3071407+2	2829	394	0,1	-17	1	-36	TTGTCCGACT	-15	GGCATAATCC	-134	1,1	9.36	12.80	s	43	Rmet_R0048	*0*	Op0795f_2	NA
108	TSS_97914+2	2828	406	0,1	-12	1	-46	TCAGCCTGAG	-10	ACTACAATGC	-140	5,1	9.89	-13.60	*no*	59	Rmet_0090	*0*	Op0025f_2	NA
109	TSS_1126276-2	2820	452	0,1	-15	1	-42	GCCCCACCCC	-13	GCTATAATTC	-139	4,1	10.69	-3.60	*no*	56	Rmet_1036	*prfB*	Op0296r_1	NA
110	TSS_947213+3	2816	288	0,1	-15	1	-35	GCTGCATTGC	-13	GCTACAATTC	-140	5,1	11.04	8.20	s	37	Rmet_4351	*0*	Op1240f_2	NA
111	TSS_2029923-3	2807	1154	0,1	-15	1	-34	TAGACCGTGA	-13	CTCATCATTT	-145	1,1	3.05	10.10	s	28	Rmet_5320	*zniB*	Op1571r_1	EIP-TRA-Ion
112	TSS_225335+4	2794	520	0,1	-15	1	-46	CCAGCCGGGG	-13	GGTATGATGC	-141	5,1	11.29	-13.60	*no*	359	Rmet_6076	*0*	Op1851f_1	NA
113	TSS_3235250-2	2782	459	0,1	-15	1	-36	TCGGCATGGG	-13	AGTACAATGT	-139	5,1	10.59	11.90	s	46	Rmet_2975	*omlA*	Op0836r_1	GIP-TL-Ass

114	TSS_3360887+2	2779	529	0,1	-16	1	-39	CCATCAGGCC	-14	TTTATAATCG	-138	5,1	9.93	3.40	m	25	Rmet_3101	*nudH*	Op0873f_1	NA
115	TSS_1350992+2	2772	288	0,1	-16	1	-35	TGGTCGTCTT	-14	TCTAGAATGC	-140	1,1	8.51	11.10	s	104	Rmet_1231	*trkD*	Op0355f_3	EIP-TRA-Ion
116	TSS_1408943+3	2764	489	0,1	-15	1	-45	GCCACCAAGG	-13	GGTATGCTCT	-142	4,1	7.93	-11.60	no	34	Rmet_4765	*0*	Op1386f_2	NA
117	TSS_3507377+2	2750	591	0,1	-24	0	-36	TTGCACTCGA	-5	GTCAAACTCA	-142	1,1	7.51	1.40	(w)-ba	41	Rmet_3231	*mscL*	Op0915f_1	EIP-TRA-Pores
118	TSS_2792546+2	2733	552	0,1	-17	1	-48	TTGTCGGTGA	-15	GGTATAATCG	-140	1,1	11.04	-8.60	no	300	Rmet_2567	*0*	Op0711f_1	NA
119	TSS_1450306+3	2709	242	0,1	-24	0	-45	GCAACCGGGC	-22	GGCAATATGG	-141	5,1	6.12	-3.60	no	117	Rmet_4802	*0*	Op1396f_6	NA
120	TSS_2456529+2	2656	533	0,1	-17	1	-49	TCGACGATAC	-15	GCGATAATGC	-140	1,1	8.63	-12.10	no	19	Rmet_2240	*0*	Op0641f_2	NA
121	TSS_3096798-2	2654	269	0,1	-16	1	-49	TTGAGTCCCC	-14	TGTACAATGA	-140	1,1	9.71	-12.60	no	29	Rmet_2847	*int*	Op0802r_2	NA
122	TSS_1402974-3	2643	183	0,1	-14	1	-48	TTGCGACACC	-12	GTTAAGGTTT	-145	1,1	7.04	-14.60	no	6	Rmet_4755	*mdoG*	Op1381r_1	NA
123	TSS_169780+2	2599	289	0,4	-18	1	-36	TGGCGTCAGG	-14	CTTAAATTCA	-144	1,1	5.60	9.90	s	230	Rmet_0162	*thiC*	Op0049f_1	MET-COF-Thia
124	TSS_3338201+2	2577	279	0,1	-16	1	-34	TTGCGGAAGT	-14	CCTATGCTTT	-142	1,1	8.25	8.60	s	880	Rmet_3084	*rdgC*	Op0867f_1	GIP-REP-Recomb
125	TSS_1098173-2	2570	239	0,1	-16	1	-34	GGCAGAGCCG	-14	ACTACAATAG	-140	4,1	8.29	5.60	m	3	Rmet_1007	*0*	Op0288r_1	NA
126	TSS_84581-2	2551	185	0,1	-15	1	-45	GTAGAGAAAT	-13	GGTAGAATCC	-143	4,1	11.21	-11.10	no	19	Rmet_0077	*0*	Op0022r_3	NA
127	TSS_435680+2	2513	57	0,1	-17	1	-38	GCTGACCGGC	-15	GTTAGACTGC	-134	5,1	10.05	2.40	w	45	Rmet_R0004	*mpB*	Op0111f_2	NA
128	TSS_1171582-2	2513	333	0,1	-16	1	-43	TTGTCGACTC	-14	TATACAATGC	-138	1,1	9.72	-0.60	no	154	Rmet_1078	*suhB*	Op0310r_1	MET-CAH-Inos
129	TSS_287256+2	2504	1613	0,1	-17	1	-49	TTGCCGCGCC	-15	GCGATACTTT	-138	1,1	8.09	-12.60	no	20	Rmet_0271	*sco2*	Op0077f_2	NA
130	TSS_197014+4	2496	251	0,1	-17	1	-43	GCCCGAGCGT	-15	GGCATAATCC	-138	4,1	10.24	-5.60	no	439	Rmet_6103	*0*	Op1863f_1	NA
131	TSS_1516769-3	2494	634	0,1	-15	1	-35	TTGTCGCCCC	-13	GCGACCATGG	-142	1,1	6.20	11.20	s	92	Rmet_4857	*0*	Op1417r_1	NA
132	TSS_160382+2	2481	280	0,1	-17	1	-43	TGGCACGCTG	-15	CGTATAATAG	-137	1,1	8.72	-1.10	no	127	Rmet_0156	*metK*	Op0045f_1	MET-AA-Met
133	TSS_2973921-2	2472	586	0,1	-7	0	-35	TGAAAACCCT	-5	GTTACGTTTT	-149	4,1	5.92	1.20	(w)-ba	125	Rmet_2737	*0*	Op0772r_2	NA
134	TSS_735967+2	2458	631	0,1	-17	1	-36	TTGACGGCCT	-15	GTCACACTTT	-139	1,1	8.03	11.80	s	270	Rmet_0669	*pilG*	Op0187f_1	GIP-PTL-T2S
135	TSS_2292501-2	2453	450	0,1	-15	1	-34	TCGAAACCGT	-13	GCTAGAATTG	-139	1,1	9.68	11.10	s	40	Rmet_2110	*ndk*	Op0608r_1	MET-NUC-Pur
136	TSS_2398060-2	2425	411	0,1	-16	1	-44	TTCAGATTTC	-14	ATTACAATGC	-139	1,1	9.50	-3.10	no	2053	Rmet_2187	*folP*	Op0628r_2	MET-COF-Folate
137	TSS_442739+2	2387	290	0,1	-16	1	-36	TCGCGGAGAC	-14	CGTAGAATTC	-140	1,1	8.54	10.90	s	201	Rmet_0417	*tyrS*	Op0115f_1	GIP-TL-Ass
138	TSS_269570+2	2378	503	0,1	-16	1	-45	CCGGCCCCGG	-14	TGTACCATAT	-139	5,1	8.99	-10.10	no	1151	Rmet_0253	*0*	Op0073f_2	GIP-REP-Recomb
139	TSS_151532+2	2366	221	0,1	-16	1	-40	TGGCGAGGAC	-14	TGTATTATTG	-140	1,1	8.32	3.90	(m)-sba	175	Rmet_0147	*0*	Op0041f_1	NA
140	TSS_315583-2	2355	119	0,1	-15	1	-48	TTAGGGAAGG	-13	GCTATGCTTG	-144	4,1	7.08	-15.10	no	3	Rmet_0297	*0*	Op0086r_3	NA
141	TSS_4804+5	2342	414	0,1	-16	1	-35	TTGCTTTCAT	-14	ACTATCATGT	-144	1,1	8.45	11.60	s	679	Rmet_6319	*0*	Op1918f_5	NA
142	TSS_2662753+2	2326	440	0,1	-17	1	-39	TCGCTATTCT	-15	GCTACAATAT	-144	1,1	9.05	6.90	s	150	Rmet_2451	*fdxA*	Op0685f_2	NA
143	TSS_3020130+2	2315	122	0,1	-18	1	-45	TTCAAAATTC	-16	GGTAAAATCC	-139	1,1	10.67	-3.10	no	38	Rmet_6558	*0*	Op0779f_1	NA
144	TSS_3304140-2	2313	397	0,1	-16	1	-35	TTGTGAAATA	-14	TATAAAATGC	-143	1,1	9.71	12.60	s	2619	Rmet_3045	*int*	Op0856r_1	NA
145	TSS_2393528-2	2300	320	0,1	-16	1	-34	TGGAACTGTC	-14	CATACAGTTC	-144	1,1	7.02	8.10	s	43	Rmet_2185	*pstS*	Op0628r_4	EIP-TRA-ABC
146	TSS_2222532-2	2292	329	0,1	-15	1	-47	TCACGAAAAG	-13	GCTATGCTTC	-143	4,1	8.64	-13.60	no	212	Rmet_2050	*sucA*	Op0596r_2	NA
147	TSS_2215251-2	2283	415	0,4	0	0	-48	TTGATCTTTA	-14	CCTAGTATTC	-144	1,1	7.69	-11.60	no	16	Rmet_2046	*rdxI*	Op0596r_2	MET-EN-P_ATPase
148	TSS_449051+3	2262	471	0,1	-17	1	-35	TGCACATAGT	-15	TGTAAGGTGT	-143	1,1	5.43	8.60	s	272	Rmet_3904	*yeaG*	Op1092f_1	NA
149	TSS_142426-2	2260	1504	0,1	-15	1	-37	TTGCAAGCGC	-13	GCTACACTCG	-134	1,1	10.24	9.40	s	0	Rmet_0136	*regB*	Op0036r_1	NA
150	TSS_3201487+2	2259	366	0,1	-16	1	-38	GCCAAGGCGG	-14	GATAGAATGC	-139	4,1	10.20	3.40	m	32	Rmet_2945	*yajC*	Op0831f_2	GIP-PTL-Exp
151	TSS_3927958+2	2244	254	0,1	-18	1	-38	TTGTGGATAA	-16	GGTACAATCC	-140	1,1	10.42	10.40	s	234	Rmet_0001	*dnaA*	Op0001f_1	GIP-REP-Complex
152	TSS_1715990+3	2235	160	0,1	-17	1	-35	TTGCTTTCAA	-15	GTTATGCTTG	-142	1,1	8.05	9.60	s	20	Rmet_5030	*cusD*	Op1480f_2	NA
153	TSS_2050840-2	2222	142	0,1	-15	1	-41	TTGCCGCCGA	-13	CGTATGATGC	-140	1,1	9.44	2.40	(w)-sba	61	Rmet_1884	*clpX*	Op0544r_2	GIP-PTL-Fold
154	TSS_2372359-3	2219	432	0,1	-16	1	-35	TTCCCATGTC	-14	TGCATAATTT	-141	1,1	8.73	12.10	s	24	Rmet_6752	*0*	Op1675r_2	NA
155	TSS_3166533-2	2206	822	0,1	-14	1	-34	TGGTCGCCGG	-12	GTTAAAATTG	-140	1,1	8.70	10.70	s	103	Rmet_2911	*purM*	Op0824r_1	MET-NUC-Pur
156	TSS_50872+4	2203	537	0,1	-16	1	-34	TGCCGTTATT	-14	TGTATAATGT	-141	1,1	8.60	7.60	s	44	Rmet_6006	*int*	Op1831f_1	NA
157	TSS_117544+5	2196	467	0,1	-16	1	-47	TGCATTACAA	-14	TTTAAACTCA	-149	1,1	5.28	-12.60	no	49	Rmet_6263	*rhsA*	Op1910f_1	NA
158	TSS_2700218+2	2192	269	0,1	-16	1	-35	TTGCATTGGC	-14	GGTACCGTGG	-140	1,1	7.83	9.60	s	1522	Rmet_2489	*mdh*	Op0689f_1	MET-CAH-TCA
159	TSS_3055460+2	2188	122	0,1	-17	1	-38	TTGCTCAGAT	-15	GCTAAATTCC	-140	1,1	8.89	8.40	s	160	Rmet_2808	*yjgP*	Op0791f_1	NA
160	TSS_3211786-2	2185	518	0,1	-14	1	-34	TTGTACGGTT	-12	AGTACAATGG	-139	1,1	9.78	11.20	s	130	Rmet_2952	*phcB*	Op0832r_3	NA
161	TSS_1165160+2	2178	204	0,1	-15	1	-33	TTCCGGAGGC	-13	CGTATAATGC	-134	1,1	10.04	8.10	s	6495	Rmet_1079	*0*	Op0311f_1	GIP-TL-Ass
162	TSS_2155214+2	2158	239	0,1	-16	1	-34	TTGCACTTCA	-14	TATAAAATCA	-140	1,1	9.61	9.60	s	43	Rmet_1981	*lexA*	Op0577f_1	GIP-TK-Assoc
163	TSS_2859546+2	2157	784	0,1	-16	1	-45	GCACGAGAGC	-14	GCTAGAATGG	-135	4,1	12.22	-10.60	no	86	Rmet_2626	*0*	Op0735f_1	NA
164	TSS_2322613+2	2129	620	0,1	-16	1	-41	TGGCGAGGAC	-14	GGTAACATAT	-140	1,1	7.78	0.90	(w)-sba	127	Rmet_2134	*trxA*	Op0615f_1	GIP-PTL-Fold
165	TSS_2700277-2	2126	194	0,1	-15	1	-38	TATTGGTGCG	-13	GCTAAAATCA	-138	2,1	12.65	4.40	m	156	Rmet_2486	*sdhC*	Op0688r_2	MET-CAH-TCA
166	TSS_388763+2	2125	699	0,1	-15	1	-40	TTGCGGTGCG	-13	AGTACAATCG	-138	2,1	13.32	3.40	(m)-sba	52	Rmet_0367	*0*	Op0103f_2	NA
167	TSS_1869264-3	2106	327	0,1	-10	1	-35	TTGACCGAGG	-8	AGTATGATCG	-141	1,1	9.37	9.20	(s)-ba	5364	Rmet_6711	*0*	Op2086r_1	NA
168	TSS_966140+3	2104	623	0,1	-16	1	-34	TTACAGACGC	-14	CATATACTGA	-145	4,1	8.56	8.10	s	28	Rmet_4368	*0*	Op1246f_2	NA
169	TSS_1325581+2	2103	303	0,1	-16	1	-37	TGCCCGCAGC	-14	GGCATAATTG	-138	1,1	7.73	8.40	s	26	Rmet_1206	*ykgE*	Op0351f_1	NA
170	TSS_558408-2	2081	545	0,1	-27	0	-46	GCAGATACGG	-25	TTCAGAATTC	-146	5,1	6.55	-5.20	no	43	Rmet_0522	*0*	Op0140r_1	NA
171	TSS_2679759-2	2076	460	0,1	-15	1	-43	TGCCGCACGT	-13	GGTAGAATTC	-138	1,1	8.47	-5.60	no	385	Rmet_2467	*trpB*	Op0688r_3	MET-AA-PheTyrTrp
172	TSS_2357290-3	2069	191	0,1	-16	1	-36	TTGACATAGT	-14	GTGACACTCA	-140	1,1	8.24	13.40	s	166	Rmet_5602	*0*	Op1669r_1	NA
173	TSS_2643125-2	2066	188	0,1	-15	1	-35	TTGACCGCTT	-13	CGTATAATCG	-139	1,1	10.96	15.20	s	71	Rmet_2433	*0*	Op0680r_1	NA
174	TSS_320096-2	2042	730	0,2	-16	1	-35	TTCCAACAAC	-14	CATAGAATAG	-142	1,1	7.91	11.10	s	0	Rmet_0303	*rpoN*	Op0086r_1	GIP-TK-RNAP

175	TSS_1464185+2	2035	442	0,1	-29	0	-36	TTGACAGCGT	-14	GCAACAATGC	-140	1,1	9.04	14.40	s	2158	Rmet_1357	*phaA*	Op0383f_2	MET-CAH-Pyr
176	TSS_3240890-2	2034	564	0,1	-14	1	-40	ATCCGTCCCG	-12	GCTAAAATTG	-138	2,1	11.73	-2.10	*no*	80	Rmet_2980	*cbbT2*	Op0840r_1	MET-CAH-PP_KDPG
177	TSS_478461-2	2024	350	0,1	-16	1	-46	CCGGCCCTGT	-14	TGTATGCTTC	-138	5,1	9.45	-12.10	*no*	25	Rmet_0450	*corC*	Op0118r_2	EIP-TRA-Other
178	TSS_2991830+2	2017	322	0,1	-17	1	-43	TCCCCTAGCG	-15	GATACACTAT	-144	1,1	5.92	-3.60	*no*	21	Rmet_2752	*0*	Op0775f_2	NA
179	TSS_1320500-2	2016	213	0,1	-16	1	-34	TTGACACTCA	-14	CTTAGAATTG	-140	1,1	9.97	12.60	s	145	Rmet_1200	*phaP*	Op0348r_1	NA
180	TSS_2300567-2	2016	266	0,1	-16	1	-34	CTCCCGCGCC	-14	GGTACAATCG	-140	1,1	5.18	6.10	m	0	Rmet_2118	*surE*	Op0612r_1	MET-NUC-Pur
181	TSS_3149724-2	2010	410	0,1	-16	1	-34	TTGCAGACTT	-14	GTTAGAATAA	-139	1,1	9.75	9.60	s	0	Rmet_2893	*clpS*	Op0812r_1	GIP-PTL-Fold
182	TSS_1393009-3	2007	16	0,1	-15	1	-44	TGGACCGGGC	-13	GTTACACTGC	-137	1,1	9.30	-5.10	*no*	6	Rmet_4748	*0*	Op1377r_2	NA
183	TSS_3478565+2	2006	253	0,1	-17	1	-35	TGAAGGCAGG	-15	GGTAAAATCC	-138	4,1	8.87	8.60	s	17	Rmet_3204	*ydhD*	Op0901f_3	GIP-PTL-Fold
184	TSS_2845123-2	1990	428	0,1	-15	1	-38	GCAGAAACAT	-13	CGTAAAATCG	-140	4,1	11.93	2.40	w	154	Rmet_2614	*0*	Op0732r_2	NA
185	TSS_827913+2	1985	320	0,1	-17	1	-43	TTGCTGCCAT	-15	GTCATACTTG	-141	1,1	7.98	-1.60	*no*	1713	Rmet_0754	*dnaE*	Op0215f_1	GIP-REP-DNA_Pol
186	TSS_1403208+3	1984	209	0,1	-16	1	-38	TTGACGCAA	-14	CTTACAATCG	-136	1,1	9.70	8.40	s	29	Rmet_4756	*0*	Op1382f_1	NA
187	TSS_1179001-3	1978	312	0,1	-17	1	-37	TTGACAGCCG	-15	CACATTATTC	-141	1,1	6.97	13.40	s	75	Rmet_4566	*0*	Op1315r_4	NA
188	TSS_824075-2	1965	537	0,1	-15	1	-39	TGGCATAGGC	-13	CGTAACATGA	-142	1,1	7.29	2.90	w	41	Rmet_0746	*0*	Op0212r_2	NA
189	TSS_2380493+2	1952	373	0,1	-15	1	-46	ATCCTCCGCG	-13	GCTATAATCC	-137	2,1	12.44	-12.10	*no*	244	Rmet_2174	*infA*	Op0625f_3	NA
190	TSS_928680-2	1951	393	0,1	-15	1	-34	TTGACATCAG	-13	CCGATTCTTA	-144	1,1	5.58	11.60	s	28	Rmet_6428	*0*	Op0242r_1	NA
191	TSS_1468598+2	1937	525	0,1	-16	1	-43	TTGCCGTCTT	-14	CATAGAATCA	-143	1,1	9.17	-0.60	*no*	152	Rmet_1359	*phaR*	Op0383f_2	NA
192	TSS_571926-2	1933	242	0,1	-16	1	-35	TTGCCCCGCG	-14	GGTAGAGTGG	-135	1,1	8.57	11.60	s	115	Rmet_0537	*exbB2*	Op0148r_1	NA
193	TSS_472867+3	1933	364	0,1	-16	1	-35	TTGACGAAAC	-14	TGCATAATCT	-140	1,1	9.71	13.60	s	277	Rmet_R0075	*0*	Op1098f_1	NA
194	TSS_473819+3	1928	376	0,1	-17	1	-48	TTGTGACTGC	-15	GGTAGAATTC	-139	1,1	10.38	-9.60	*no*	916	Rmet_R0076	*0*	Op1098f_1	NA
195	TSS_3224095+2	1920	269	0,1	-16	1	-34	TTCCTGCGCG	-14	GCTATACTCG	-135	2,1	13.01	8.10	s	52	Rmet_2966	*0*	Op0835f_1	NA
196	TSS_3380807-2	1914	435	0,1	-14	1	-43	TGGCAGCGGT	-12	GATAAACTCG	-138	1,1	8.48	-6.10	*no*	75	Rmet_3118	*secA*	Op0876r_2	GIP-PTL-Exp
197	TSS_1655372+2	1901	92	0,1	-16	1	-36	TGGACACGGC	-14	GCTACACTGC	-134	1,1	9.78	12.90	s	188	Rmet_1535	*hypA2*	Op0427f_4	GIP-PTL-Fold
198	TSS_177419+2	1900	251	0,1	-16	1	-41	TTCACGCCGC	-14	GCTACAATAC	-138	1,1	9.53	2.90	*(w)-sba*	210	Rmet_0170	*ahcY*	Op0051f_1	MET-AA-Met
199	TSS_500-5	1898	740	0,1	-13	1	-38	TCGCCCGCCA	-11	GGCATAATGG	-140	1,1	8.24	4.90	m	14600	Rmet_6301	*trbF*	Op1917r_1	NA
200	TSS_3134530+2	1896	491	0,1	-16	1	-35	GGCGGCAAAC	-14	CTTATAATGC	-137	4,1	10.60	6.60	s	1150	Rmet_2883	*0*	Op0811f_1	GIP-REP-Recomb
201	TSS_3561131+2	1893	302	0,1	-15	1	-37	TCAGGAGACC	-13	TGTACAATAA	-142	4,1	9.02	7.40	s	28	Rmet_3285	*engB*	Op0925f_1	NA
202	TSS_149269+3	1893	319	0,1	-17	1	-48	TTGTGACTGC	-15	GGTAGAATTC	-139	1,1	10.38	-9.60	*no*	916	Rmet_R0071	*tRNA-I*	Op1006f_1	NA
203	TSS_148317+3	1889	369	0,1	-16	1	-35	TTGACGAAAC	-14	TGCATAATCT	-140	1,1	9.71	13.60	s	277	Rmet_R0070	*0*	Op1006f_1	NA
204	TSS_2676292-2	1871	323	0,1	-15	1	-39	TTGCTTCGGT	-13	GGTAAATTCG	-140	1,1	8.49	4.40	m	79	Rmet_2464	*accD*	Op0688r_4	MET-CAH-Pyr
205	TSS_288939-2	1867	386	0,1	-16	1	-43	TTCTAACTTA	-14	GCTAATATGC	-144	1,1	7.46	-3.10	*no*	59	Rmet_0272	*rpoH*	Op0078r_1	GIP-TK-RNAP
206	TSS_508495-3	1865	419	0,1	-15	1	-36	TCAGGGATGC	-13	GGTATCGTTA	-146	4,1	6.81	7.40	s	145	Rmet_3950	*0*	Op1107r_2	NA
207	TSS_80790-3	1850	191	0,1	-17	1	-37	TTGACGCAGC	-15	GCTACACTCT	-135	1,1	10.12	12.40	s	106	Rmet_5884	*0*	Op1781r_1	NA
208	TSS_1010917+2	1843	588	0,1	-16	1	-49	TTGCCCGCAC	-14	GCTACAATAG	-137	1,1	10.58	-12.60	*no*	22	Rmet_0925	*cbbJ2*	Op0267f_2	MET-CAH-FbP
209	TSS_57758+2	1835	456	0,1	-17	1	-47	GGCCAAACGC	-15	GGTATGATTA	-140	4,1	10.93	-13.60	*no*	728	Rmet_0051	*mreB*	Op0017f_1	DIV-Division
210	TSS_3432046-2	1831	322	0,1	-16	1	-47	TTGTGACTGC	-14	GGTAGAATTC	-139	1,1	10.38	-8.60	*no*	917	Rmet_R0053	*0*	Op0886r_1	NA
211	TSS_425585+2	1829	185	0,1	-17	1	-37	TGGATTCGGG	-15	GATATGCTCC	-142	1,1	6.64	9.90	s	20	Rmet_0398	*gdhA*	Op0109f_3	MET-EN-Nitrog
212	TSS_3617279-2	1829	286	0,1	-16	1	-47	TTGTGACTGC	-14	GGTAGAATTC	-139	1,1	10.38	-8.60	*no*	917	Rmet_R0063	*0*	Op0930r_1	NA
213	TSS_3297357+2	1816	260	0,1	-16	1	-36	GTAGACGACC	-14	CGTATAGTTA	-144	4,1	7.72	7.90	s	40	Rmet_3042	*0*	Op0855f_1	NA
214	TSS_698937-3	1811	433	0,3	-14	1	-35	TTGTGCAGTC	-14	CGCAAAATGC	-140	1,1	7.90	10.60	s	5359	Rmet_4122	*tnpA*	Op2067r_1	NA
215	TSS_574314+2	1810	358	0,1	-17	1	-36	TTGCGCGGGT	-15	CCTACAATCG	-135	1,1	10.22	11.80	s	30	Rmet_0539	*sodB*	Op0149f_4	NA
216	TSS_2635929-2	1805	205	0,1	-16	1	-37	TTGTCTTGAC	-14	GATACACTGC	-141	1,1	9.34	10.40	s	11	Rmet_2425	*rpoE*	Op0680r_3	GIP-TK-RNAP
217	TSS_1337781-2	1763	330	0,1	-16	1	-41	TTGCAGCTTC	-14	CCTATAATGT	-141	1,1	10.75	3.40	*(m)-sba*	20	Rmet_1219	*0*	Op0354r_2	NA
218	TSS_2298107-2	1758	466	0,1	-16	1	-35	TTGCGCGGCT	-14	GTCATCATCA	-140	1,1	7.34	10.60	s	192	Rmet_2115	*rpoS*	Op0612r_2	GIP-TK-RNAP
219	TSS_3591955-2	1743	319	0,1	-15	1	-34	TTGACCATTG	-13	GCTAGAGTGC	-137	1,1	9.05	11.60	s	232	Rmet_3327	*rpsL*	Op0928r_2	GIP-TL-Ribo
220	TSS_3244994-2	1738	312	0,1	-15	1	-34	TTGATTAATG	-13	CGTAACCTTA	-145	1,1	6.44	9.60	s	118	Rmet_2986	*0*	Op0842r_4	NA
221	TSS_310581+2	1723	460	0,1	-16	1	-38	TTCCACAAAA	-14	TGTATCGTGT	-143	1,1	6.13	5.90	m	86	Rmet_0294	*0*	Op0085f_2	NA
222	TSS_100834+2	1723	225	0,1	-16	1	-41	ATTCGGTGCC	-14	CCTACGATGA	-139	2,1	10.32	-3.10	*no*	266	Rmet_0094	*0*	Op0027f_2	NA
223	TSS_937353+2	1721	308	0,1	-18	1	-38	CCGGAAGGTT	-16	TGTAAAATAC	-138	5,1	10.82	6.90	s	32	Rmet_0857	*rpoZ*	Op0245f_2	GIP-TK-RNAP
224	TSS_1355701+2	1721	315	0,1	-16	1	-43	TCGCCCAGGG	-14	GTTAGAATTC	-138	1,1	9.45	-2.10	*no*	800	Rmet_1234	*0*	Op0357f_1	NA
225	TSS_1136853-2	1706	472	0,1	-15	1	-33	CCGGCAACCC	-13	TGTAGAATCT	-139	5,1	11.30	6.10	m	91	Rmet_1044	*yebK*	Op0300r_1	NA
226	TSS_2045560+3	1706	612	0,1	-17	1	-41	TTCAGTGTAC	-15	GATACACTGC	-138	1,1	8.34	1.90	*(w)-sba*	552	Rmet_5334	*0*	Op1580f_1	EIP-TRA-Other
227	TSS_900814-2	1700	120	0,1	-16	1	-48	GCAGACATAT	-27	GAAAGGATGC	-144	4,1	6.22	-8.20	*no*	116	Rmet_0826	*0*	Op0234r_1	NA
228	TSS_139828-2	1699	1337	0,1	-17	1	-40	TCGCACGGCG	-15	GCTAGACTGA	-137	1,1	7.43	2.90	*(w)-sba*	29	Rmet_0133	*0*	Op0034r_1	NA
229	TSS_497506+2	1695	200	0,1	-16	1	-34	CGGTCAAAGC	-14	GTTAAACTCC	-143	1,1	3.87	6.10	m	23	Rmet_0468	*0*	Op0123f_2	NA
230	TSS_3120362-2	1691	236	0,1	-15	1	-40	GCAGCCCAAC	-13	CATACAATGG	-140	4,1	9.48	-1.60	*no*	510	Rmet_2868	*ispH*	Op0806r_1	NA
231	TSS_2278214+3	1683	288	0,1	-16	1	-38	TGCCGTATGA	-14	GATAACATAT	-144	1,1	5.52	4.40	m	842	Rmet_5532	*0*	Op1644f_1	NA
232	TSS_264262+2	1682	376	0,1	-16	1	-49	TCGAAATTTC	-14	GCCATAATGG	-141	1,1	9.29	-13.10	*no*	193	Rmet_0248	*bfr*	Op0071f_4	NA
233	TSS_3654043+2	1682	329	0,1	-24	0	-37	GCACAGGCAT	-14	GTTATTTTTA	-143	4,1	7.80	3.40	m	5063	Rmet_3384	*gspG*	Op0941f_1	GIP-PTL-T2S
234	TSS_59263+4	1681	235	0,1	-16	1	-45	TTCTCTTTCG	-14	GCGACAATAC	-142	1,1	6.15	-7.10	*no*	4547	Rmet_5994	*gntR*	Op1827f_1	NA
235	TSS_3618231-2	1676	296	0,1	-15	1	-34	TTGACGAAAC	-13	TGCATAATCT	-140	1,1	9.71	12.60	s	278	Rmet_R0064	*0*	Op0930r_1	NA

236	TSS_29976+4	1662	355	0,1	-16	1	-38	GCACCAATCA	-14	GCTATAATTT	-143	4,1	9.97	5.40	m	19	Rmet_6033	*0*	Op1837f_1	NA
237	TSS_3432998-2	1652	342	0,1	-15	1	-34	TTGACGAAAC	-13	TGCATAATCT	-140	1,1	9.71	12.60	s	278	Rmet_R0054	*0*	Op0886r_1	NA
238	TSS_318024-2	1645	183	0,1	-16	1	-34	TCGCAACTGA	-14	TGTATGCTGC	-139	1,1	7.48	8.10	s	248	Rmet_0300	*ptsN*	Op0086r_3	EIP-TRA-Pts
239	TSS_1323277+2	1644	125	0,1	-16	1	-35	TTGCTAACAA	-14	CCTAAACTAG	-145	1,1	8.16	11.60	s	24	Rmet_1204	*0*	Op0349f_2	NA
240	TSS_753390+3	1623	200	0,1	-16	1	-34	CCGGCCTCGC	-14	TATATACTGG	-137	5,1	9.44	6.10	m	37	Rmet_4167	*ttg2*	Op1172f_1	NA
241	TSS_2504460-2	1606	338	0,1	-15	1	-35	TTCACCTGAA	-13	TCTACAATTC	-140	1,1	9.81	12.70	s	30	Rmet_2282	*0*	Op0652r_2	NA
242	TSS_531004+2	1605	435	0,1	-15	1	-33	TCGTACGGTA	-13	GGTAAAATGC	-139	1,1	8.92	7.10	s	1332	Rmet_0502	*pykA1*	Op0135f_1	MET-CAH-FbP
243	TSS_2912871+2	1585	70	0,1	-17	1	-36	TTGCAGCAAG	-15	CGTAAACTCT	-135	1,1	8.72	10.80	s	11	Rmet_2680	*glyA*	Op0757f_1	MET-EN-Methane
244	TSS_2890921+2	1583	241	0,1	-5	0	-35	TTGCCTTCCA	-14	TATAAAGTCA	-145	1,1	7.03	11.60	s	237	Rmet_2655	*elaB*	Op0753f_1	NA
245	TSS_2152353-2	1580	137	0,1	-17	1	-39	TTTGTTTGCG	-15	GCTATAATTG	-134	2,1	15.44	6.90	s	0	Rmet_1975	*dnaB*	Op0576r_2	GIP-REP-Complex
246	TSS_326585-2	1575	181	0,1	-15	1	-40	GCTACCACCC	-13	CGCACAATGG	-144	5,1	7.19	-1.60	*no*	680	Rmet_0309	*0*	Op0088r_1	NA
247	TSS_2912710-2	1573	33	0,1	-16	1	-49	TTGCAAAGTC	-14	GTTACAATGC	-137	1,1	11.97	-12.60	*no*	0	Rmet_2679	*ybgC*	Op0756r_1	NA
248	TSS_1312058-2	1568	184	0,1	-15	1	-42	TTCCCCAAGC	-13	GATAAACTGC	-138	1,1	9.00	-2.10	*no*	17	Rmet_1195	*0*	Op0346r_1	NA
249	TSS_505817+3	1567	383	0,1	-17	1	-37	GGAAAAGCGC	-15	GTTATGCTGC	-143	4,1	10.78	6.40	s	45	Rmet_3949	*phaZ3*	Op1106f_1	NA
250	TSS_484090+2	1565	245	0,1	-17	1	-41	TATCGCTCCG	-15	GGTACACTAC	-136	2,1	11.66	-0.60	*no*	127	Rmet_0456	*rhlE1*	Op0119f_1	NA
251	TSS_2807646-2	1561	283	0,1	-16	1	-47	TAGCGTCCGG	-14	GGTAGAATCC	-141	2,1	10.80	-11.10	*no*	3587	Rmet_2579	*0*	Op0716r_1	NA
252	TSS_3346716-2	1561	170	0,1	-15	1	-43	TTGTCAAGAC	-13	ACTATACTTA	-140	1,1	9.12	-0.60	*no*	266	Rmet_3088	*nrdA*	Op0870r_3	MET-NUC-Pur
253	TSS_3612660-2	1538	221	0,1	-9	0	-31	GCTGAAGCGG	-7	TATAGGGTGA	-145	5,1	5.91	-0.80	*no*	153	Rmet_R0059	*0*	Op0930r_2	NA
254	TSS_1744679-3	1527	105	0,1	-16	1	-36	TCTGCACGCG	-14	GTTACACTAC	-135	5,1	10.55	10.40	s	117	Rmet_5053	*0*	Op1487r_1	NA
255	TSS_996501-2	1526	238	0,1	-16	1	-36	TGGCAGAGTC	-14	GCTAGCATCG	-143	1,1	7.90	9.90	s	73	Rmet_0910	*rpoR*	Op0262r_1	GIP-TK-RNAP
256	TSS_1160361+2	1524	232	0,1	-17	1	-47	TTCCGTGGGT	-15	GGTACAATCG	-138	1,1	9.75	-10.10	*no*	24	Rmet_1068	*dapA*	Op0307f_2	MET-AA-Lys_Syn
257	TSS_1172770+3	1522	168	0,1	-17	1	-36	TTGATTCATT	-15	CCGAAACTGT	-147	1,1	6.07	10.80	s	28	Rmet_4564	*metE*	Op1314f_1	MET-AA-Met
258	TSS_2442279+3	1522	165	0,1	-17	1	-36	TTGACCCTTC	-15	CATACACTCG	-140	1,1	8.79	12.80	s	51	Rmet_5684	*pilE*	Op1698f_1	GIP-PTL-T2S
259	TSS_3606599+2	1520	301	0,1	-24	0	-42	TTCCGCTTGC	-22	TGTAGGATTC	-139	1,1	7.62	2.30	*w*	11417	Rmet_6590	*0*	Op0931f_1	NA
260	TSS_1764792+3	1508	134	0,1	-16	1	-36	TTGCGGGTTC	-14	TGTATATTTA	-140	1,1	8.50	12.40	s	86	Rmet_5076	*0*	Op1494f_1	NA
261	TSS_35575-2	1502	301	0,1	-15	1	-47	TTGCTTGGTT	-13	GTTAAATTTT	-144	1,1	8.09	-11.60	*no*	77	Rmet_0032	*0*	Op0012r_3	NA
262	TSS_3751974+2	1500	107	0,1	-16	1	-39	TGGCCCCCGG	-14	TCTATAATCG	-137	1,1	9.50	6.90	s	248	Rmet_3473	*0*	Op0965f_2	NA
263	TSS_24767+2	1487	116	0,1	-18	1	-43	TTCCAGGTTT	-16	GAAATACTGT	-145	1,1	5.91	-2.10	*no*	23	Rmet_0021	*0*	Op0003f_4	MET-AA-Phe
264	TSS_1838974+2	1487	137	0,1	-16	1	-41	TTGCGATTGC	-14	GTTAATTTTG	-145	1,1	7.58	1.40	*(w)-sba*	403	Rmet_1697	*0*	Op0481f_1	NA
265	TSS_3383634-2	1478	206	0,1	-15	1	-39	TGGTGCAGCG	-13	GGTAGTATCG	-146	1,1	6.49	2.90	w	45	Rmet_3121	*lpxC*	Op0878r_4	MET-GLYC-LPS
266	TSS_23019-4	1474	89	0,1	-15	1	-44	TTGAGCACAC	-13	GTTATACTTG	-139	1,1	10.42	-3.60	*no*	43	Rmet_6352	*0*	Op1842r_2	NA
267	TSS_98451+5	1468	144	0,1	-17	1	-40	TCTGCTGCGT	-15	GATATAATGC	-139	5,1	10.78	4.40	*(m)-sba*	296	Rmet_6252	*int*	Op1904f_4	NA
268	TSS_1241770+2	1467	272	0,1	-15	1	-39	TCGCTTTACT	-13	GCTAGGATCG	-138	1,1	6.97	2.90	w	74	Rmet_1135	*0*	Op0325f_1	NA
269	TSS_154592+2	1464	176	0,1	-16	1	-35	TTGAGGTTGG	-14	CGTAACATCT	-141	1,1	8.49	11.60	s	23	Rmet_0150	*pyrE*	Op0043f_1	MET-NUC-Pyr
270	TSS_68280+2	1458	212	0,1	-17	1	-48	CCGGCCCGCG	-15	GTTACACTCC	-137	5,1	11.27	-15.10	*no*	804	Rmet_0061	*lipA*	Op0019f_2	NA
271	TSS_2971582-2	1451	218	0,1	-15	1	-46	TGGACGGGAG	-13	GATAGAATAC	-141	1,1	8.79	-8.10	*no*	107	Rmet_2735	*rfbB*	Op0770r_1	MET-CAH-NucSug
272	TSS_2122846+2	1440	138	0,1	-16	1	-43	TTGTACCGAT	-14	CCTACAATCC	-140	1,1	9.59	-1.60	*no*	22	Rmet_1952	*0*	Op0571f_2	NA
273	TSS_1163887-2	1435	105	0,1	-16	1	-42	TTGACTGTAC	-14	GATATAATCG	-139	1,1	11.35	3.40	*(m)-sba*	165	Rmet_1071	*0*	Op0308r_2	NA
274	TSS_590915+3	1425	182	0,1	-22	0	-36	TTGCCAGAAT	-15	GATAGTCTTC	-141	1,1	8.11	11.80	s	33	Rmet_4020	*0*	Op1128f_2	NA
275	TSS_236869-2	1418	286	0,1	-15	1	-35	GGCGCCACGC	-13	CCTATAATCG	-140	4,1	9.89	8.20	s	32	Rmet_0222	*nppD*	Op0066r_1	MET-EN-Nitrog
276	TSS_1072661-2	1408	335	0,1	-15	1	-43	TCCCCCACTG	-13	CTTATACTGG	-140	1,1	6.63	-4.60	*no*	116	Rmet_0986	*0*	Op0278r_1	NA
277	TSS_791288+2	1403	197	0,1	-1	0	-33	GAACTCGCCA	-13	CGGACTAATC	-143	3,0	6.89	-1.40	no	21	Rmet_0714	*0*	Op0203f_2	NA
278	TSS_1629844-2	1400	214	0,1	-15	1	-38	CCGTGAAAGT	-13	GGTATAATCG	-142	1,1	5.31	4.90	m	42	Rmet_6453	*0*	Op0424r_2	NA
279	TSS_3860317-2	1400	419	0,1	-15	1	-46	GGCGGCGCCC	-13	TGTAGAATAC	-141	4,1	8.14	-14.60	*no*	307	Rmet_3559	*0*	Op0988r_3	NA
280	TSS_944957+2	1397	192	0,1	-16	1	-48	TTGAATCACA	-14	GCTAAAATAG	-137	1,1	10.17	-10.60	*no*	44	Rmet_0866	*aspH*	Op0249f_2	NA
281	TSS_1178983+2	1393	178	0,1	-17	1	-35	TCGGAAACGG	-15	GGTAGAATGC	-141	5,1	11.45	10.10	s	22	Rmet_1087	*accA*	Op0313f_2	MET-CAH-Pyr
282	TSS_754395+2	1393	150	0,1	-15	1	-41	TGCACGCAAT	-13	GCTAGAATCC	-141	1,1	8.51	0.40	*(w)-sba*	32	Rmet_0681	*glnB*	Op0191f_2	EIP-SIG-2Comp
283	TSS_788155-2	1393	370	0,1	-16	1	-38	TGGATCATGC	-14	GTTATACTCC	-141	1,1	9.12	7.90	s	68	Rmet_0712	*ompA*	Op0202r_2	EIP-TRA-Pores
284	TSS_3065559-2	1380	213	0,1	-15	1	-33	TTGAGGTATG	-13	ATTACGATTC	-146	1,1	8.59	8.60	s	100	Rmet_2817	*0*	Op0792r_1	NA
285	TSS_165121-3	1359	290	0,1	-15	1	-34	TGGCGCACTG	-13	TGTATACTGT	-141	1,1	8.38	9.10	s	15	Rmet_3628	*0*	Op1011r_3	NA
286	TSS_3508602+2	1358	200	0,1	-16	1	-45	TTGAAACGGG	-14	AGTACAATTC	-139	1,1	10.92	-3.60	*no*	33	Rmet_3233	*0*	Op0917f_1	NA
287	TSS_784535-2	1356	216	0,1	-14	1	-32	CCAGCACGCT	-12	GCTACAATAG	-138	5,1	11.80	3.60	m	31	Rmet_0707	*0*	Op0200r_2	NA
288	TSS_1336320-2	1352	168	0,1	-16	1	-43	GGCGCCATAC	-14	GATACACTTG	-138	4,1	8.44	-7.60	*no*	22	Rmet_1217	*0*	Op0354r_1	NA
289	TSS_1393475-3	1346	210	0,1	-15	1	-35	TTGCCCAAGG	-13	ATTAGACTGC	-139	1,1	8.94	12.20	s	60	Rmet_4749	*hupB*	Op1377r_2	GIP-REP-Recomb
290	TSS_829538-2	1345	199	0,1	-15	1	-33	GTCGGGATGC	-13	GCTATAATTT	-138	4,1	10.75	5.10	m	62	Rmet_0753	*0*	Op0214r_1	NA
291	TSS_2189998-2	1342	227	0,1	-14	1	-42	GCAAAAGCAC	-12	GCTAGAATGT	-141	4,1	12.89	-5.60	*no*	40	Rmet_2024	*bipA*	Op0592r_1	NA
292	TSS_3468889+2	1330	229	0,1	-16	1	-40	CCGATAGGGT	-14	GTTACAATTG	-143	1,1	5.06	1.90	*(w)-sba*	158	Rmet_3193	*ychF*	Op0897f_3	NA
293	TSS_3236872-2	1329	315	0,1	-16	1	-39	TTGCTTTTCG	-14	CTTACACTCG	-145	2,1	11.54	5.40	m	78	Rmet_2977	*phcA*	Op0838r_2	NA
294	TSS_3130101+2	1324	275	0,4	-17	1	-36	TTGCAAACCA	-15	TACTTAATTA	-149	1,1	2.39	10.80	s	216	Rmet_2879	*tnpA*	Op0809f_1	NA
295	TSS_1795637+3	1324	182	0,1	-16	1	-35	TTGACGCACC	-14	CATACCATTC	-138	1,1	8.42	12.60	s	46	Rmet_5104	*0*	Op1504f_2	NA
296	TSS_2181681+2	1319	173	0,1	-17	1	-41	TCGTCTGTCT	-15	GCTACGATAC	-139	1,1	6.68	1.90	*(w)-sba*	1542	Rmet_6502	*0*	Op2024f_1	NA

297	TSS_2069443+3	1314	320	0,1	-8	0	-27	TGGCACGGGT	-6	GCCCCGATCA	-147	1,1	1.31	-1.90	no	25	Rmet_6720	0	Op1590f_1	NA
298	TSS_2796437-2	1314	398	0,1	-14	1	-38	TCGCGTCATG	-12	GCTATGATTC	-141	1,1	8.00	4.90	m	27	Rmet_2570	*pmbA*	Op0712r_1	NA
299	TSS_2144527-2	1312	240	0,1	-15	1	-35	GCAAGAATCC	-13	GTCATAATCC	-139	4,1	10.64	8.20	s	11	Rmet_1967	*aatA*	Op0574r_1	NA
300	TSS_3819200-2	1312	146	0,1	-17	1	-40	TTGAAACAGT	-15	AGGATAATGC	-139	1,1	9.53	7.40	(s)-sba	39	Rmet_3526	*ompW1*	Op0976r_3	NA
301	TSS_1555971-2	1305	434	0,1	-13	1	-38	TTCCCCCTGC	-11	GCGATAATCC	-139	1,1	8.26	4.90	m	59	Rmet_1434	*map*	Op0402r_1	GIP-TL-Ass
302	TSS_3840763-2	1300	95	0,1	-11	1	-47	TCGAGCATTT	-9	CCAACAGTTC	-145	1,1	3.76	-17.10	no	3823	Rmet_3539	0	Op0980r_1	NA
303	TSS_37918-2	1292	117	0,1	-14	1	-38	TTGTTACAGT	-12	GTTAGAATGT	-136	1,1	9.76	7.40	s	20	Rmet_0035	*minC*	Op0012r_1	DIV-Division
304	TSS_2363673+2	1292	158	0,1	-16	1	-34	TTGCAACGAA	-14	GCTATCCTGC	-144	1,1	9.36	9.60	s	21	Rmet_2163	0	Op0623f_1	NA
305	TSS_1151894-3	1291	223	0,1	-16	1	-35	TTGCTCCGAG	-14	CATAGAGTCC	-139	1,1	6.44	10.60	s	49	Rmet_4545	0	Op1303r_2	NA
306	TSS_3111668+2	1291	43	0,1	-17	1	-36	TTGACACCCG	-15	CTTATATTGG	-138	1,1	8.76	14.80	s	167	Rmet_2862	*argD*	Op0805f_1	MET-AA-Lys_Syn
307	TSS_3628184-2	1290	223	0,1	-15	1	-34	TATCGGATCC	-13	GATATGATTC	-140	2,1	9.67	7.60	s	1006	Rmet_3350	0	Op0932r_1	NA
308	TSS_3241850+2	1284	103	0,1	-16	1	-35	TCGACATGCG	-14	CGTAACATGC	-136	1,1	7.61	12.10	s	55	Rmet_2982	0	Op0841f_2	NA
309	TSS_1982792-2	1270	221	0,4	-16	1	-40	TCGCGGTAGG	-13	GGTACAAGTC	-147	1,1	1.67	-0.10	no	56	Rmet_1826	*pilZ*	Op0530r_2	GIP-PTL-T2S
310	TSS_1412143+3	1266	97	0,1	-16	1	-34	TCGACAAGTA	-14	GATAAAATGT	-141	1,1	9.87	11.10	s	29	Rmet_4767	*ecnAB*	Op1386f_3	NA
311	TSS_1114985-2	1264	303	0,1	-13	1	-36	TGCAGCGTCC	-11	GTTACAATTG	-140	1,1	8.31	7.40	s	5919	Rmet_1018	*tyrB*	Op0294r_1	MET-AA-Met
312	TSS_301988+2	1260	262	0,1	-17	1	-35	TTCCAATTGC	-15	GTTACACTAC	-138	1,1	8.94	9.10	s	93	Rmet_0285	*coaD*	Op0083f_3	MET-COF-Pant
313	TSS_283685-2	1260	241	0,1	-15	1	-33	TTCCCTCCGG	-13	GCGATAATGG	-136	1,1	7.80	8.10	s	20	Rmet_0266	0	Op0076r_1	NA
314	TSS_1226053-3	1258	555	0,1	-28	0	-46	GCAAGAAATT	-26	TCCAGCATGG	-145	4,1	5.74	-5.20	no	4817	Rmet_4599	*tnpR*	Op1323r_1	NA
315	TSS_2523175-2	1256	163	0,1	-15	1	-34	TTGACAAGGT	-13	GCCATCATTA	-140	1,1	9.01	12.60	s	43	Rmet_2302	*pbrR2*	Op0658r_1	NA
316	TSS_3870556+2	1252	194	0,1	-17	1	-37	TTGCACCGCC	-15	CTTATTATTG	-136	1,1	8.66	11.40	s	90	Rmet_3569	*topB*	Op0989f_2	GIP-REP-Complex
317	TSS_3507238-2	1249	257	0,1	-17	1	-37	TATCGTCGCC	-15	GGTATAATTT	-139	2,1	14.65	9.40	s	39	Rmet_3230	*petA*	Op0914r_1	MET-EN-OxPhos
318	TSS_2707896+2	1240	172	0,1	-14	1	-49	TTGAGGGTTC	-12	AGTAGAATAC	-141	1,1	9.24	-14.60	no	29	Rmet_2494	0	Op0689f_3	NA
319	TSS_221398+4	1234	146	0,1	-16	1	-34	TTGATCTTCT	-14	AGTATCATGC	-137	1,1	9.27	10.60	s	104	Rmet_6080	0	Op1853f_3	NA
320	TSS_1723457-3	1233	204	0,2	-15	1	-44	AAACGTCCTA	-24	TGCGGATACC	-146	3,0	4.45	-9.20	no	24	Rmet_5036	0	Op1481r_3	NA
321	TSS_771602-2	1232	177	0,1	-25	0	-45	CCGGCACGCG	-23	TCGACGCTCG	-139	5,1	7.03	-2.10	no	355	Rmet_0698	0	Op0196r_1	NA
322	TSS_520929-2	1218	275	0,1	-15	1	-33	TGGCACGCAA	-13	CTTACAATGG	-138	1,1	8.72	7.10	s	37	Rmet_0493	*ilvE*	Op0132r_1	MET-AA-ValLeuIle
323	TSS_80346-2	1215	292	0,1	-15	1	-35	TCGCCATGCC	-13	CATAGAATCG	-137	1,1	8.39	12.70	s	22	Rmet_0072	0	Op0020r_1	EIP-TRA-ABC
324	TSS_3329237-2	1213	136	0,1	-19	0	-27	TGGAGCGTGT	-5	ATCAACATGT	-142	1,1	5.33	1.70	(w)-nu	249	Rmet_3072	0	Op0862r_2	NA
325	TSS_560616-2	1208	335	0,1	-15	1	-44	GGCAGAATGC	-13	CGTACAATTC	-137	4,1	11.04	-8.60	no	51	Rmet_0524	0	Op0142r_1	NA
326	TSS_819884-2	1192	389	0,1	-15	1	-43	AAATTCGGTA	-23	CCTTGAAGTC	-147	3,0	8.02	-6.20	no	42	Rmet_0740	*dadA*	Op0208r_1	MET-EN-Nitrog
327	TSS_1143461+2	1185	97	0,1	-15	1	-43	TTCATACACC	-13	GCTAGAATGG	-137	1,1	9.31	-2.10	no	30	Rmet_1050	0	Op0301f_1	GIP-PTL-Fold
328	TSS_3796892-2	1184	117	0,1	-14	1	-41	GTCCGACCGA	-12	GCTATAATTC	-141	4,1	9.57	-2.10	no	41	Rmet_3506	*gidA*	Op0972r_2	DIV-Division
329	TSS_407971-2	1183	209	0,1	-15	1	-36	TGCACATGGC	-13	GATATTGTCG	-142	1,1	5.65	10.40	s	27	Rmet_0382	*mobB*	Op0104r_1	MET-COF-Folate
330	TSS_3431821-2	1183	295	0,3	-13	1	-35	TTGACCGCCT	-13	CGCAAGATTA	-145	1,1	6.59	12.20	s	692	Rmet_R0053	0	Op0886r_1	NA
331	TSS_2273426+2	1183	219	0,1	-16	1	-34	TTGACAAACG	-14	GCTATAATGG	-139	1,1	12.49	13.60	s	18	Rmet_R0025	0	Op0607f_1	NA
332	TSS_474044+3	1177	267	0,3	-14	1	-36	TTGACCGCCT	-14	CGCAAGATTA	-145	1,1	6.59	12.40	s	691	Rmet_R0076	0	Op1098f_1	NA
333	TSS_3680608+2	1177	176	0,1	-15	1	-36	TCGACAGGCG	-13	CCTATGATGG	-137	1,1	8.15	12.90	s	412	Rmet_3403	*proP*	Op0945f_2	NA
334	TSS_3617054-2	1173	279	0,3	-13	1	-35	TTGACCGCCT	-13	CGCAAGATTA	-145	1,1	6.59	12.20	s	692	Rmet_R0063	0	Op0930r_1	NA
335	TSS_2399025+2	1172	117	0,1	-17	1	-49	TGGAGATCTT	-25	GATATTTTGG	-142	1,1	6.74	-3.10	no	19	Rmet_2190	0	Op0629f_1	NA
336	TSS_2227538-3	1168	162	0,1	-15	1	-35	TTGCACGTTT	-13	GCTATGCTGC	-139	1,1	9.39	11.20	s	2321	Rmet_5481	*tnmB*	Op2095r_1	NA
337	TSS_1265911-2	1166	115	0,1	-15	1	-41	TGGCCAAAGC	-13	AGTACAATGC	-138	1,1	10.22	1.90	(w)-sba	31	Rmet_1156	*bdhA*	Op0332r_1	MET-CAH-But
338	TSS_58980-3	1165	239	0,1	-15	1	-34	TTGCAATCAG	-13	CATAAGATGA	-142	1,1	8.30	10.60	s	224	Rmet_5861	0	Op1773r_3	NA
339	TSS_2322680+2	1162	177	0,1	-16	1	-44	GCCGCCGCGC	-14	TGTAAGGTAG	-141	4,1	5.48	-10.60	no	60	Rmet_2134	*trxA*	Op0615f_1	GIP-PTL-Fold
340	TSS_149494+3	1159	251	0,3	-14	1	-36	TTGACCGCCT	-14	CGCAAGATTA	-145	1,1	6.59	12.40	s	691	Rmet_R0071	*tRNA-I*	Op1006f_1	NA
341	TSS_1092055+2	1157	233	0,1	-16	1	-44	TCCAGGTGAC	-14	ATTAAAATCG	-141	1,1	7.14	-5.60	no	2	Rmet_1000	0	Op0285f_1	NA
342	TSS_2803220-2	1156	184	0,1	-20	0	-36	TTGCAAAGCC	-13	GCTATCGTCT	-143	1,1	8.11	11.40	s	55	Rmet_2578	0	Op0716r_2	MET-COF-Porph
343	TSS_2273779+2	1150	230	0,1	-16	1	-35	TTGACGCGCC	-14	ACTACCATAG	-141	1,1	8.00	12.60	s	15	Rmet_2093	0	Op0607f_2	NA
344	TSS_3134344-2	1145	282	0,1	-16	1	-41	TCGCAATGGC	-14	GGCATGATAT	-141	1,1	7.04	0.90	(w)-sba	4313	Rmet_2878	*purN*	Op0808r_1	NA
345	TSS_1540377+2	1140	156	0,1	-17	1	-39	GCGGCCGGCC	-15	GGTATGATTA	-137	5,1	11.53	3.90	m	108	Rmet_1426	*smc*	Op0399f_1	DIV-Division
346	TSS_3548056+2	1134	25	0,1	-16	1	-36	GCCGGGCCGC	-14	GATATAATCC	-137	4,1	10.61	7.40	s	20	Rmet_3274	*mrcA*	Op0921f_1	NA
347	TSS_3153081+2	1129	308	0,1	-2	0	-22	TTGCCGCTTC	0	GACACGTTCT	-144	1,1	5.15	-2.80	no	62	Rmet_2897	0	Op0815f_2	NA
348	TSS_75122+4	1114	145	0,1	-16	1	-34	TGCTGGTGAT	-14	TGTAAACTTC	-142	1,1	6.40	5.60	m	53	Rmet_5983	*czcI*	Op1820f_1	NA
349	TSS_204268+2	1112	131	0,1	-17	1	-37	CCTGCAGGTT	-15	GATACACTGC	-137	5,1	10.92	6.40	s	38	Rmet_R0002	0	Op0057f_1	NA
350	TSS_1697045-2	1108	135	0,1	-28	0	-49	TTCTCGTTAG	-26	TGCAATATTT	-147	1,1	4.87	-4.10	no	164	Rmet_1576	0	Op0434r_1	NA
351	TSS_1061141-2	1101	362	0,1	-26	0	-41	GCGGCAAGTG	-13	GCTAATTTAA	-143	5,1	7.33	-3.10	no	18	Rmet_0974	*paaH*	Op0272r_1	MET-CAH-But
352	TSS_3631209+2	1097	237	0,1	-16	1	-35	TTGACACGGA	-14	GGAAAACTAA	-143	1,1	6.56	12.60	s	343	Rmet_3355	0	Op0935f_2	NA
353	TSS_3091113-2	1094	169	0,1	-15	1	-28	TTTCGTATCG	-8	GATACGATGA	-144	2,1	11.87	1.10	(w)-nu	597	Rmet_2842	*tnpA*	Op2044r_1	NA
354	TSS_540999-2	1088	177	0,1	-16	1	-34	TTGCGATTAG	-14	GATAGAATGC	-137	1,1	10.91	10.60	s	23	Rmet_0509	0	Op0136r_1	NA
355	TSS_2956853+2	1087	192	0,1	-16	1	-47	CCTGCTGGGT	-14	GGTAGCATTT	-143	5,1	8.94	-15.60	no	77	Rmet_2722	0	Op0769f_2	NA
356	TSS_1504146+2	1086	71	0,1	-16	1	-38	CCTTACAGCT	-14	GATACAATCG	-137	5,1	9.50	2.40	w	757	Rmet_1396	*rimI*	Op0391f_1	GIP-TL-Ass
357	TSS_1461800-2	1085	118	0,1	-16	1	-42	CCGGCCCGGT	-14	GCTATAATGC	-135	5,1	13.40	-2.10	no	23	Rmet_1352	*comL*	Op0380r_1	NA

358	TSS_2247091+2	1080	162	0,1	-17	1	-37	TCCCGCCCGG	-15	GCTACAATAG	-138	1,1	7.16	8.40	s	563	Rmet_2072	*0*	Op0601f_2	NA
359	TSS_442871-2	1080	92	0,1	-15	1	-39	TTGCGACGGA	-13	GGTATAATCC	-139	1,1	11.96	7.40	s	149	Rmet_0416	*0*	Op0114r_1	MET-AA-PheTyrTrp
360	TSS_3672158+2	1075	189	0,1	-17	1	-44	TCGCACGGTA	-15	GCTACCATGC	-137	1,1	8.01	-5.10	*no*	97	Rmet_3396	*porG*	Op0943f_1	MET-CAH-PyrOx
361	TSS_63772+4	1072	356	0,1	-16	1	-47	TTGCGAAAAA	-14	GCTAGAATTT	-143	1,1	10.90	-8.60	*no*	38	Rmet_5994	*gntR*	Op1827f_1	NA
362	TSS_102323-2	1067	174	0,1	-15	1	-45	TGGAAATCGG	-13	GCTATGCTGA	-139	1,1	8.05	-7.10	*no*	56	Rmet_0095	*0*	Op0028r_1	NA
363	TSS_791999+2	1066	414	0,1	-16	1	-34	TCCAAGGGGC	-14	GGGATAATGG	-138	1,1	7.09	7.60	s	37	Rmet_0715	*serC*	Op0203f_2	MET-AA-GlySerThr
364	TSS_2155178-3	1066	108	0,1	-16	1	-36	GGAGGACCGA	-14	GCTACAATTT	-139	4,1	9.64	7.40	s	20	Rmet_5412	*0*	Op1611r_1	NA
365	TSS_757383+2	1065	120	0,1	-17	1	-43	TTCCAATGAG	-15	GTTAAAATGG	-139	1,1	10.36	-1.10	*no*	35	Rmet_0685	*ftsK*	Op0193f_1	EIP-TRA-Other
366	TSS_457603-2	1062	163	0,1	-15	1	-38	GCGGCCCGCT	-13	TTTAGAATCG	-135	5,1	11.36	3.90	m	23	Rmet_0432	*pepP*	Op0116r_2	NA
367	TSS_551895-3	1059	193	0,1	-15	1	-35	TTGCGCTGCA	-13	ACGATAATGG	-139	1,1	8.32	12.20	s	59	Rmet_3984	*yebQ*	Op1117r_2	NA
368	TSS_1107659-2	1055	66	0,1	-16	1	-39	TGCAAAGCAC	-14	TGCATAATCG	-143	4,1	9.07	5.40	m	42	Rmet_1017	*phaZ1*	Op0294r_1	NA
369	TSS_2891125+2	1051	177	0,1	-16	1	-49	GGACGACAAG	-14	GGCAAAATGT	-143	4,1	8.67	-19.60	*no*	33	Rmet_2655	*elaB*	Op0753f_1	NA
370	TSS_1488403-3	1051	108	0,1	-16	1	-42	CCGGCCCTGT	-14	ACTACACTGA	-138	5,1	9.58	-4.10	*no*	5560	Rmet_4828	*0*	Op1407r_1	NA
371	TSS_3513971-2	1051	166	0,1	-15	1	-38	TGGAGTGTTC	-13	TGTATCGTAT	-143	1,1	4.99	5.90	m	129	Rmet_3238	*0*	Op0918r_3	EIP-TRA-ABC
372	TSS_1404341-3	1048	94	0,1	-15	1	-33	TCGTCGCGCG	-13	GCGAAAATGC	-141	1,1	6.38	7.10	s	29	Rmet_4757	*0*	Op1383r_2	NA
373	TSS_1685062+3	1044	88	0,1	-12	1	-45	TCGCCATCCC	-10	CGTCCAATGC	-143	1,1	4.46	-11.10	*no*	1316	Rmet_5007	*0*	Op1470f_1	NA
374	TSS_2045375+2	1043	197	0,5	-5	0	-36	TTGCTCGTCA	-14	TGTATGTTGC	-141	1,1	6.98	11.40	s	10085	Rmet_1888	*yhiR*	Op0545f_1	NA
375	TSS_750255+2	1034	31	0,1	-17	1	-39	TCAGCCAGAG	-15	GCTATAATGC	-135	5,1	12.66	6.40	s	30	Rmet_0678	*ppa*	Op0189f_1	MET-EN-OxPhos
376	TSS_2361799-2	1033	201	0,1	-15	1	-39	TCGATGACAG	-13	GATAATATAG	-143	1,1	6.16	3.90	m	42	Rmet_2161	*0*	Op0622r_1	NA
377	TSS_725748-3	1029	123	0,1	-15	1	-40	TCACAAATCC	-13	GCTATAATCG	-138	4,1	11.28	2.40	*(w)-sba*	92	Rmet_4149	*crtB*	Op1169r_3	MET-LIP-Ster
378	TSS_884464+2	1025	211	0,1	-17	1	-39	TGGCCCGCAC	-15	GATAAAATCG	-139	1,1	9.31	6.90	s	33	Rmet_0808	*0*	Op0229f_2	NA
379	TSS_2442033-3	1025	113	0,1	-17	1	-46	CCTGCCACCG	-15	GCTACAATCC	-138	5,1	11.94	-11.60	*no*	0	Rmet_5683	*0*	Op1697r_1	NA
380	TSS_2334425-3	1024	225	0,1	-16	1	-34	TTGCAAATTC	-14	TGTAGAATTC	-136	1,1	10.96	10.60	s	7	Rmet_R0086	*0*	Op1661r_1	NA
381	TSS_1539116-2	1023	219	0,1	-15	1	-42	TTCACGCGCG	-13	TTTATAATGC	-137	1,1	9.78	0.90	*(w)-sba*	1325	Rmet_1423	*dapD*	Op0398r_2	MET-AA-Lys_Syn
382	TSS_2839437-2	1022	86	0,1	-15	1	-33	TGCAGCTTGG	-13	GCTAGAATCG	-136	1,1	8.17	6.60	s	69	Rmet_2609	*rpsU2*	Op0730r_2	GIP-TL-Ribo
383	TSS_785856-2	1018	78	0,1	-9	0	-45	TCGAAAGCGA	-7	CGCACATTGG	-145	1,1	5.36	-14.10	*no*	0	Rmet_R0007	*ssrA*	Op0202f_2	NA
384	TSS_104375+2	1014	68	0,1	-17	1	-42	TCTGAAGCCG	-15	TCTATAATCC	-137	5,1	11.21	0.40	*(w)-sba*	17	Rmet_0097	*0*	Op0029f_1	NA
385	TSS_2786174-2	1013	91	0,1	-16	1	-41	GCTGCCGCCG	-14	GCTATACTGC	-138	5,1	11.54	-2.60	*no*	52	Rmet_R0042	*0*	Op0708r_1	NA
386	TSS_2883532+2	1009	52	0,1	-17	1	-39	TTCAGCGGTG	-15	GCTACAATCG	-137	1,1	9.47	6.90	s	73	Rmet_2646	*0*	Op0747f_1	NA
387	TSS_1120139+3	1009	266	0,2	-5	0	-37	TGGCGCCACC	-15	CGCATAATTA	-141	1,1	7.09	9.90	s	63	Rmet_4515	*0*	Op1290f_1	NA
388	TSS_2924293+2	1005	142	0,1	-16	1	-44	GCCAGAGCGG	-14	CGTACAATAG	-135	4,1	9.80	-7.60	*no*	85	Rmet_2693	*ribBA*	Op0759f_1	MET-COF-Ribo
389	TSS_2398110-2	998	268	0,1	-16	1	-36	TTGCCCTGAA	-14	GGCACTATCA	-137	1,1	7.43	11.40	s	41	Rmet_2188	*ftsH*	Op0628r_2	GIP-PTL-Fold
390	TSS_2400744-2	995	185	0,1	-15	1	-35	TTGATACCGG	-13	CCTACACTAC	-137	1,1	8.27	13.20	s	96	Rmet_2192	*greA*	Op0630r_3	GIP-TK-Assoc
391	TSS_3365760+2	994	63	0,1	-16	1	-34	CCAAACGCAC	-14	CATATAATCC	-141	5,1	7.85	5.60	m	22	Rmet_3107	*ispB*	Op0875f_1	NA
392	TSS_3142117+2	984	103	0,1	-16	1	-38	TTCCAGCGCG	-14	GGTATCATGC	-136	1,1	8.79	6.90	s	27	Rmet_2887	*dfp*	Op0811f_1	NA
393	TSS_3166540+2	980	227	0,1	-15	1	-38	TGGCGTAGAA	-13	AGTAAAATCG	-140	1,1	8.56	5.90	m	82	Rmet_2912	*hda*	Op0825f_1	NA
394	TSS_171373+5	976	170	0,1	-17	1	-36	TTGCGTATAC	-15	GCTAACCTTC	-140	1,1	8.21	9.80	s	86	Rmet_6315	*parA*	Op1918f_4	NA
395	TSS_2277098-2	972	385	0,1	-12	1	-41	GCCCGAAAAC	-10	AGTAGAATAC	-141	4,1	10.47	-6.60	*no*	25	Rmet_2096	*purA*	Op0608r_4	MET-NUC-Pur
396	TSS_144117-2	971	170	0,1	-16	1	-35	GCCCAAACAC	-14	TTTAACATTC	-141	4,1	10.22	5.60	m	0	Rmet_0138	*0*	Op0038r_2	NA
397	TSS_1777944-2	969	150	0,1	-16	1	-41	TTGCACGGTT	-14	GGTATGCTTG	-141	1,1	9.32	1.40	*(w)-sba*	40	Rmet_1644	*0*	Op0462r_2	NA
398	TSS_1643970+2	969	128	0,1	-16	1	-36	TTGCAAGATC	-14	GCGACAATGC	-140	1,1	9.52	12.40	s	46	Rmet_1522	*hoxF*	Op0425f_2	MET-CAH-DiCarb
399	TSS_733026+2	966	99	0,1	-17	1	-36	TTGACCACGC	-15	GCTAGATTCT	-134	1,1	8.76	12.80	s	230	Rmet_0666	*dapA*	Op0185f_1	MET-AA-Lys_Syn
400	TSS_1301089+2	959	52	0,1	-15	1	-38	TCGAAGACGA	-13	GTTAGGATGC	-143	1,1	7.82	5.90	m	24	Rmet_1187	*0*	Op0343f_2	NA
401	TSS_2864575+2	956	386	0,1	-16	1	-43	GCCGAAATGC	-14	CCTATAATTT	-136	4,1	11.61	-5.60	*no*	76	Rmet_2630	*valS*	Op0737f_1	MET-AA-ValLeuIle
402	TSS_1943913-3	956	119	0,1	-15	1	-40	GCCGACAAGC	-13	CATAAAATAG	-138	4,1	9.78	-2.60	*no*	61	Rmet_5244	*0*	Op1545r_2	NA
403	TSS_1530537-3	952	111	0,1	-16	1	-49	TGGCAATAGC	-14	ACCATAATTC	-144	1,1	8.61	-14.10	*no*	44	Rmet_4868	*llpE*	Op1419r_2	MET-CAH-But
404	TSS_3645289-2	950	323	0,1	-15	1	-35	TCGAGTGGCC	-13	GCTACATTAG	-140	1,1	6.55	10.70	s	217	Rmet_3368	*0*	Op0936r_1	NA
405	TSS_1540149-2	949	255	0,1	-15	1	-37	GCCAGGAAAC	-13	GCTAGTCTCT	-142	4,1	8.15	2.40	w	118	Rmet_1425	*0*	Op0398r_1	NA
406	TSS_3623853-2	949	134	0,1	-15	1	-37	TTCCTTATAA	-13	GGCAAGATGT	-143	1,1	5.90	5.90	m	192	Rmet_3346	*uspA8*	Op0932r_3	NA
407	TSS_476573+3	944	133	0,1	-16	1	-42	GCAGAGATGC	-14	GTTAATATTC	-140	4,1	10.08	-6.60	*no*	1605	Rmet_R0079	*0*	Op1098f_1	NA
408	TSS_2666395+2	944	151	0,1	-16	1	-40	TGGCGGTCGT	-14	TGTATGATCT	-140	1,1	8.25	2.90	*(w)-sba*	294	Rmet_2455	*rpsU1*	Op0685f_3	GIP-TL-Ribo
409	TSS_2404058-3	943	362	0,1	-14	1	-49	TTGTACGAAA	-28	GCCACTTTCG	-146	1,1	4.37	-4.20	*no*	69	Rmet_5642	*fliD3*	Op1683r_3	DIV-MOT-Flagel
410	TSS_2340060+2	943	185	0,1	-15	1	-33	TTGTGGCCGG	-13	GCGACAATTT	-139	1,1	7.37	7.60	s	134	Rmet_2147	*fabI*	Op0619f_1	MET-LIP-FASyn
411	TSS_152023+3	942	208	0,1	-16	1	-42	GCAGAGATGC	-14	GTTAATATTC	-140	4,1	10.08	-6.60	*no*	1605	Rmet_R0074	*0*	Op1006f_1	NA
412	TSS_3740560+2	941	73	0,1	-17	1	-46	GGACGACCGT	-15	TGTAGAATCG	-137	4,1	9.91	-11.60	*no*	558	Rmet_3464	*htpX*	Op0963f_2	GIP-PTL-Fold
413	TSS_272516+2	939	144	0,1	-17	1	-41	TTCTTAGGGG	-15	GGTATGCTCC	-141	1,1	6.74	1.90	*(w)-sba*	79	Rmet_0254	*secB*	Op0073f_2	GIP-PTL-Exp
414	TSS_400237+2	938	125	0,1	-15	1	-37	TTGCCCCCCG	-13	GCTATAATCC	-136	1,1	11.26	11.40	s	178	Rmet_0375	*0*	Op0103f_4	NA
415	TSS_3304224+2	938	118	0,1	-17	1	-40	TTTGTTTGGG	-15	GGTACAATAT	-142	2,1	13.43	3.90	*(m)-sba*	75	Rmet_3047	*thiL*	Op0857f_1	MET-COF-Thia
416	TSS_29818+2	937	317	0,1	-17	1	-39	TGGTCATCCT	-15	GGCATCATGG	-139	1,1	6.99	6.90	s	342	Rmet_0026	*bug*	Op0007f_2	NA
417	TSS_3424092+2	936	350	0,1	-17	1	-36	TGGCGCTTTG	-15	CGTATGATCG	-138	1,1	7.86	10.30	s	31	Rmet_3159	*hipO*	Op0885f_1	MET-AA-Phe
418	TSS_1595226-2	935	21	0,1	-26	0	-35	TTGACATTAC	-13	GCTGTAATGC	-144	1,1	6.35	14.20	s	356	Rmet_1474	*0*	Op0412r_1	NA

419	TSS_1176505+2	932	98	0,1	-17	1	-44	GCAGAGGCAG	-15	GTTATACTCC	-138	4,1	10.30	-8.60	no	95	Rmet_1085	*cysS*	Op0313f_1	MET-AA-Cys
420	TSS_2384478+2	932	125	0,1	-29	0	-44	CAAAGTGTCA	-24	ACGATTCTAT	-152	3,0	3.77	-4.20	no	22	Rmet_2178	*ppk*	Op0627f_1	MET-EN-OxPhos
421	TSS_2458171-2	931	104	0,1	-15	1	-33	GGCAGCCACC	-13	GCTAGAGTTC	-142	4,1	6.92	2.60	w	37	Rmet_2243	*0*	Op0642r_1	NA
422	TSS_2903707-2	929	188	0,1	-16	1	-35	TTGATCTGGC	-14	ACGATCATGT	-140	1,1	6.67	11.60	s	249	Rmet_2669	*0*	Op0756r_5	NA
423	TSS_1334171-2	926	179	0,1	-16	1	-39	TTGCAGACGT	-14	GATAGACTGC	-136	1,1	9.76	5.40	m	24	Rmet_1214	*ate*	Op0352r_1	GIP-PTL-Fold
424	TSS_781322-2	920	186	0,1	-15	1	-33	GTCACAACCA	-13	GGTAAAATGC	-139	4,1	9.41	7.10	s	421	Rmet_0704	*cphA2*	Op0198r_1	NA
425	TSS_1951037+3	917	532	0,4	-28	0	-37	TGGATTGACT	-14	CATCTAATCC	-146	1,1	3.51	8.90	s	729	Rmet_5251	*0*	Op1550f_1	NA
426	TSS_3429292-2	917	228	0,1	-15	1	-41	GCAGAGATGC	-13	GTTAATATTC	-140	4,1	10.08	-5.60	no	1606	Rmet_R0050	*0*	Op0886r_1	NA
427	TSS_333592+2	913	89	0,1	-16	1	-47	TTCATAGGGT	-14	CTTATAATCG	-137	1,1	9.45	-8.10	no	332	Rmet_0315	*ssb1*	Op0091f_2	GIP-REP-Complex
428	TSS_3025025-2	912	124	0,1	-15	1	-45	TCGCGCCGCT	-13	GCTACACTTG	-136	1,1	8.23	-9.10	no	102	Rmet_2777	*cbiA*	Op0782r_2	DIV-Division
429	TSS_3865822-2	912	4	0,1	-4	0	-34	TTGAATTTCC	-2	GTTATCGTGT	-143	1,1	7.79	1.20	(w)-ba	56	Rmet_3563	*0*	Op0988r_2	GIP-PTL-Fold
430	TSS_3631981+2	911	138	0,1	-16	1	-36	TTCCCTCGAA	-14	GGCATAGTCT	-141	1,1	6.01	10.90	s	4	Rmet_3356	*0*	Op0935f_2	NA
431	TSS_2292749+2	910	152	0,1	-16	1	-35	TTGCAAATCC	-14	GCCACATTGG	-140	1,1	8.01	10.60	s	52	Rmet_2111	*0*	Op0609f_1	NA
432	TSS_2093592-2	909	115	0,1	-16	1	-35	TGGATTCCCC	-14	GTTACAATCC	-136	1,1	8.76	11.10	s	128	Rmet_R0022	*0*	Op0562r_2	NA
433	TSS_1309316-2	909	110	0,1	-16	1	-46	TTCACCCGGG	-14	GTTACACTTC	-137	1,1	8.87	-8.10	no	25	Rmet_1194	*0*	Op0346r_2	NA
434	TSS_3614525-2	903	160	0,1	-15	1	-41	GCAGAGATGC	-13	GTTAATATTC	-140	4,1	10.08	-5.60	no	1606	Rmet_R0060	*0*	Op0930r_1	NA
435	TSS_2354-5	903	51	0,1	-15	1	-33	TTGACTCAGG	-13	GCTACACTCC	-140	1,1	9.97	9.60	s	16454	Rmet_6301	*trbF*	Op1917r_1	NA
436	TSS_943084+2	900	282	0,1	-3	0	-35	TTGACAAGCT	-1	CATATGCTGC	-139	1,1	8.88	2.20	(w)-ba	1487	Rmet_0865	*0*	Op0249f_1	NA
437	TSS_983219+2	897	116	0,1	-16	1	-36	TTGCCATAGC	-14	TGGAGAATGC	-142	1,1	9.04	13.40	s	70	Rmet_0896	*0*	Op0261f_1	NA
438	TSS_1664-5	892	177	0,1	-4	0	-32	AAACTTCTCG	-12	AATCTGACTG	-144	3,0	8.22	-2.40	no	15764	Rmet_6301	*trbF*	Op1917r_1	NA
439	TSS_2986852-2	889	179	0,1	-15	1	-41	TCAGGGGCCT	-13	GCTAACATGC	-142	4,1	7.72	-2.60	no	0	Rmet_2749	*hemC*	Op0774r_1	MET-COF-Porph
440	TSS_1731447-3	889	95	0,1	-27	0	-42	AACATCGGTG	-22	CCATTCGTCG	-146	3,0	4.51	-4.20	no	37	Rmet_5044	*0*	Op1485r_4	NA
441	TSS_2046187-2	887	126	0,1	-15	1	-39	TTCAAGCAGT	-13	GATAGAATCG	-142	1,1	9.29	4.90	m	36	Rmet_1882	*ppiD*	Op0544r_2	GIP-PTL-Fold
442	TSS_3178581-2	878	93	0,1	-17	1	-48	TTGCGAATTT	-15	GCCACATTTC	-144	1,1	7.96	-11.60	no	54	Rmet_2922	*dnaK*	Op0826r_2	GIP-PTL-Fold
443	TSS_1907983+2	877	48	0,1	-16	1	-35	TCGCGGATCC	-14	GTTAGTCTCA	-142	1,1	5.29	8.10	s	23	Rmet_1758	*0*	Op0501f_2	NA
444	TSS_1893666-2	873	118	0,1	-16	1	-46	GCAAACCAGC	-14	GTTACCCTTG	-142	4,1	8.88	-14.60	no	26	Rmet_1745	*0*	Op0496r_1	NA
445	TSS_3531278-2	870	151	0,1	-15	1	-39	TGGTCTGTGC	-13	TCTACAATGG	-140	1,1	8.66	4.90	m	81	Rmet_3260	*bug*	Op0918r_1	NA
446	TSS_1415492+3	869	201	0,1	-15	1	-35	TTGACGTAAT	-13	ACTACGATAT	-144	1,1	8.64	13.20	s	69	Rmet_4771	*0*	Op1388f_1	NA
447	TSS_275454-3	868	156	0,1	-15	1	-35	TTGCGGAACC	-13	TATACACTCG	-137	1,1	8.82	11.20	s	1420	Rmet_3729	*icdA*	Op1041r_3	MET-CAH-TCA
448	TSS_862285-2	867	201	0,1	-17	1	-42	TGGCGCTTCG	-15	GATAAAATCC	-137	1,1	9.25	-0.10	no	144	Rmet_0785	*0*	Op0222r_1	NA
449	TSS_2465033+2	866	86	0,1	-16	1	-42	TTGCCATGCA	-14	GATATATTCG	-144	1,1	9.70	2.40	(w)-sba	72	Rmet_2250	*bcsB*	Op0645f_1	NA
450	TSS_2694694-2	862	106	0,1	-15	1	-40	TTCACTCTAT	-13	TTTATAATGC	-142	1,1	10.02	4.90	(m)-sba	304	Rmet_2480	*livK1*	Op0688r_2	EIP-TRA-ABC
451	TSS_2282612-2	862	101	0,1	-16	1	-34	TTGAACCGGG	-14	CCCATGATCC	-143	1,1	7.48	9.60	s	25	Rmet_2101	*hfq*	Op0608r_2	NA
452	TSS_971694+3	857	213	0,1	-17	1	-47	TTCCAGAAGA	-15	TCTATACTCG	-142	1,1	8.74	-10.10	no	40	Rmet_4373	*0*	Op1248f_1	NA
453	TSS_801392+2	856	97	0,1	-16	1	-41	TGGTTACCGT	-14	GTTACAATGC	-141	1,1	9.04	1.90	(w)-sba	77	Rmet_0724	*0*	Op0205f_2	NA
454	TSS_1694401+2	853	145	0,1	-15	1	-43	GCCCGCGACT	-13	GGTAAAATAT	-140	4,1	9.03	-8.60	no	99	Rmet_1575	*0*	Op0433f_2	NA
455	TSS_971452-3	852	199	0,1	-16	1	-34	TTGACAAACC	-14	GCTATTCTCA	-141	1,1	8.92	11.60	s	818	Rmet_4371	*0*	Op1247r_1	NA
456	TSS_2869150+2	851	142	0,1	-17	1	-39	TTGCGACGCA	-15	ATTATGCTGT	-142	1,1	7.66	7.40	s	788	Rmet_2634	*0*	Op0739f_1	NA
457	TSS_428710+3	844	119	0,1	-16	1	-44	CCAAAAGGCT	-14	GATACATTGG	-143	5,1	7.25	-8.60	no	5034	Rmet_3889	*0*	Op1084f_1	NA
458	TSS_1841486+2	842	198	0,1	-17	1	-35	TTGCGATTTA	-15	GGCACACTCT	-141	1,1	8.36	9.60	s	57	Rmet_1700	*0*	Op0481f_2	NA
459	TSS_157548-2	833	251	0,1	-15	1	-35	TTGACCTCTC	-13	CTGACCATTG	-139	1,1	6.39	12.20	s	45	Rmet_0152	*0*	Op0044r_3	NA
460	TSS_3751839-2	832	188	0,1	-16	1	-35	TGGACGGGCT	-14	AGTATCCTAT	-139	1,1	6.30	11.10	s	43	Rmet_R0066	*0*	Op2056r_1	NA
461	TSS_2315710+3	828	131	0,1	-17	1	-44	TTCCCCGACG	-15	CGTAAAATGG	-141	1,1	8.95	-3.10	no	3414	Rmet_5565	*0*	Op1654f_1	NA
462	TSS_735657+2	827	127	0,1	-16	1	-35	TGGCGTCATC	-14	GGTACCATCT	-140	1,1	7.59	9.10	s	8	Rmet_0668	*rubA*	Op0187f_1	NA
463	TSS_973445-2	827	163	0,1	0	0	-48	TTGACCATAA	-14	TCTAGCCTGT	-144	1,1	7.57	-11.60	no	57	Rmet_0888	*0*	Op0258r_2	NA
464	TSS_1288970-2	825	61	0,1	-15	1	-41	CCGGCACGTG	-13	GATATACTGT	-139	5,1	11.18	-1.10	no	60	Rmet_1177	*0*	Op0340r_1	NA
465	TSS_224481+4	823	182	0,1	-16	1	-35	TTGCACAGAT	-14	GCTAATATCT	-137	1,1	9.43	10.60	s	109	Rmet_6077	*0*	Op1851f_1	NA
466	TSS_2904834-2	822	153	0,1	-16	1	-47	TTGCAGTGGG	-14	GCTATAATTT	-135	1,1	11.93	-8.60	no	64	Rmet_2670	*0*	Op0756r_5	NA
467	TSS_2030373-2	820	237	0,1	-15	1	-43	TTGCGCAATG	-13	GGTACAATAG	-137	1,1	10.60	-2.60	no	80	Rmet_1870	*purL*	Op0540r_2	MET-NUC-Pur
468	TSS_1514264+2	818	306	0,1	-16	1	-49	TTGCCGGGGC	-24	ATGAAAATGG	-140	1,1	8.02	-3.60	no	23	Rmet_1403	*dsbB*	Op0395f_1	GIP-PTL-Fold
469	TSS_93181+2	814	146	0,1	-17	1	-44	CCTGACCGCC	-15	GATAAAATCG	-138	5,1	11.14	-8.60	no	55	Rmet_0087	*bhmT*	Op0025f_1	MET-AA-Met
470	TSS_3851961+2	810	35	0,1	-16	1	-37	TTGGGCAGGG	-14	TGTATCATTG	-139	2,1	8.30	10.40	s	31	Rmet_3552	*0*	Op0985f_1	NA
471	TSS_911276-2	810	335	0,1	-15	1	-34	TCGTCGTATG	-13	AGTATAATGA	-140	1,1	8.35	11.10	s	976	Rmet_0832	*aldH*	Op0238r_1	NA
472	TSS_1471503+2	808	229	0,1	-17	1	-35	TTCGTTTTCC	-15	TCTATACTGT	-142	2,1	10.35	9.10	s	25	Rmet_1362	*bktB*	Op0383f_4	MET-CAH-Pyr
473	TSS_3023593+2	800	208	0,1	-16	1	-48	TTACGAAAAA	-14	TGTAGGATTT	-146	4,1	6.76	-13.10	no	120	Rmet_2776	*0*	Op0781f_1	NA
474	TSS_2633434-2	798	213	0,1	-14	1	-44	GCGGCCGGCG	-12	GGTACACTGA	-137	5,1	11.48	-10.10	no	126	Rmet_2422	*mucD*	Op0680r_4	NA
475	TSS_1766989-2	794	100	0,1	-15	1	-33	TTGAAAATTC	-13	TATATATTTA	-141	1,1	9.01	10.60	s	43	Rmet_1637	*0*	Op0460r_1	NA
476	TSS_3658916-2	794	35	0,1	-16	1	-35	TTGCATTGGC	-14	TCTATGATTC	-141	1,1	9.90	11.60	s	480	Rmet_3382	*hmp*	Op0940r_1	NA
477	TSS_2836371-2	793	117	0,1	-15	1	-40	TGACAGGAAG	-13	GCTACAATGC	-142	4,1	9.72	0.40	(w)-sba	50	Rmet_2606	*rpoD1*	Op0730r_3	GIP-TK-RNAP
478	TSS_3736971+2	791	118	0,1	-16	1	-36	TCGTTGAAGG	-14	TGTAGACTCC	-139	1,1	6.23	9.90	s	264	Rmet_3461	*0*	Op0963f_1	MET-AA-Met
479	TSS_2684092-2	788	54	0,1	-15	1	-35	TGGCTATTTT	-13	GTCACAATAC	-138	1,1	7.77	10.70	s	168	Rmet_2470	*0*	Op0688r_3	NA

480	TSS_460609-2	786	77	0,1	-16	1	-47	TCCCGGAGGC	-14	GGTATTATAC	-138	1,1	6.77	-12.60	no	21	Rmet_0435	0	Op0116r_1	NA
481	TSS_474378+3	780	237	0,1	-17	1	-43	TGGCCCTTAT	-15	CATACAATGG	-139	1,1	9.41	-1.10	no	357	Rmet_R0076	0	Op1098f_1	NA
482	TSS_355051-3	779	495	0,1	-14	1	-34	TGGCGTGAAT	-12	GCTACACTTC	-140	1,1	8.67	8.70	s	75	Rmet_6628	0	Op1061r_1	NA
483	TSS_3314978-2	778	105	0,1	-15	1	-34	GCGGCCGCGG	-13	GGTATCATCC	-136	5,1	11.05	7.10	s	59	Rmet_3056	0	Op0860r_2	GIP-TL-Ass
484	TSS_3561089-2	778	129	0,1	-20	0	-49	CCGGAAAGCG	-18	TTTACGGTCT	-144	5,1	7.61	-15.10	no	192	Rmet_3284	0	Op0924r_1	NA
485	TSS_149828+3	777	220	0,1	-17	1	-43	TGGCCCTTAT	-15	CATACAATGG	-139	1,1	9.41	-1.10	no	357	Rmet_R0071	tRNA-I	Op1006f_1	NA
486	TSS_1298152+2	775	49	0,1	-16	1	-46	TCGCAATTTT	-14	CTTACAATGC	-142	1,1	9.60	-8.10	no	1039	Rmet_1186	atzB	Op0343f_1	MET-XEN-Others
487	TSS_323357-2	774	47	0,1	-17	1	-38	GGCCAGACGC	-15	GGTATACTCC	-137	4,1	10.94	3.40	m	0	Rmet_0307	kdsD2	Op0086r_1	NA
488	TSS_2121450+2	771	130	0,5	-14	1	-43	TTGCATCAGG	-10	CTCATTCTGT	-145	1,1	5.48	-7.60	no	21	Rmet_1950	ahpC	Op0571f_2	GIP-PTL-Fold
489	TSS_572795+2	770	137	0,1	-16	1	-23	GACATTGCCG	-3	GACATGTCCA	-145	3,0	4.37	-10.40	no	1454	Rmet_6423	0	Op0149f_3	NA
490	TSS_1083252-2	768	172	0,1	-19	0	-44	TTGCCCTCGG	-17	GGGACAGTCA	-140	1,1	6.41	-1.60	no	191	Rmet_0993	0	Op0282r_1	NA
491	TSS_1578776+2	764	142	0,1	-15	1	-40	CCGGACGTGC	-13	GCTAAAATTG	-135	5,1	11.41	-1.10	no	55	Rmet_1457	yfjG	Op0407f_2	NA
492	TSS_3718723-2	763	203	0,1	-16	1	-40	TTGTATGCAA	-14	GCTAACATGA	-145	1,1	7.75	3.40	(m)-sba	99	Rmet_3443	0	Op0958r_1	MET-NUC-Pur
493	TSS_122854+3	763	31	0,1	-16	1	-48	TTCTGTCCGC	-14	CATATACTGG	-139	1,1	6.63	-13.10	no	265	Rmet_5921	0	Op1798f_2	NA
494	TSS_1397555-3	757	37	0,4	-16	1	-28	GCATTTGCCG	-8	AATTGCGCCT	-143	3,0	5.42	-8.40	no	3781	Rmet_6684	0	Op1377r_1	NA
495	TSS_454384-2	756	44	0,1	-16	1	-37	TTGAGCAACC	-14	GGTAAAATCC	-137	1,1	11.27	11.40	s	16	Rmet_0429	dusB	Op0116r_2	MET-COF-CoQ
496	TSS_3263756-2	756	80	0,1	-6	0	-35	TTGACGACCA	-14	TGCACACTGT	-143	1,1	7.73	11.60	s	28	Rmet_3007	tniR	Op0846r_1	NA
497	TSS_2848469-2	752	74	0,1	-15	1	-37	TTCTGCGACC	-13	CGTAAAATCC	-140	1,1	7.77	7.90	s	21	Rmet_2617	xseB	Op0732r_1	GIP-REP-Recomb
498	TSS_3836350+2	751	204	0,1	-17	1	-38	TTCCGGTGCA	-15	GGTATGATGT	-139	1,1	8.79	7.90	s	82	Rmet_3538	hup	Op0979f_2	GIP-REP-Recomb
499	TSS_288971-2	750	52	0,1	-12	1	-39	TTGCCTGGAA	-10	TCTAACTTAG	-144	1,1	5.88	1.40	w	91	Rmet_0272	rpoH	Op0078r_1	GIP-TK-RNAP
500	TSS_1955883+2	749	153	0,1	-9	0	-35	TGCATCGGCT	-15	CGTATTCTGG	-141	1,1	5.05	7.60	s	83	Rmet_1803	ynjF	Op0523f_2	NA
501	TSS_3616720-2	748	238	0,1	-16	1	-42	TGGCCCTTAT	-14	CATACAATGG	-139	1,1	9.41	-0.10	no	358	Rmet_R0063	0	Op0930r_1	NA
502	TSS_1907541-3	748	72	0,1	-15	1	-49	CTCACATGGG	-13	CGTATAATTG	-149	1,1	5.84	-15.10	no	31	Rmet_5213	0	Op1535r_1	NA
503	TSS_2421305+2	745	129	0,1	-17	1	-42	TGGCGTCGAT	-15	TTTACAATCC	-137	1,1	8.84	-0.10	no	849	Rmet_R0035	0	Op0631f_3	NA
504	TSS_2848710+2	745	71	0,1	-17	1	-48	GCAAAAAAAT	-15	GGTACAATCC	-137	4,1	13.55	-14.60	no	196	Rmet_2618	0	Op0733f_1	NA
505	TSS_1652784+3	744	126	0,1	-7	0	-36	TTGACGACCA	-15	TGCACACTGT	-143	1,1	7.73	11.80	s	27	Rmet_4975	tniR	Op1460f_1	NA
506	TSS_1215464-3	743	13	0,4	0	0	-36	TTGACCCTGT	-13	TCTTCAATGG	-145	1,1	4.89	11.40	s	12	Rmet_4594	zntA	Op1321r_1	MET-EN-P_ATPase
507	TSS_1278448+2	742	152	0,1	-21	0	-35	TCGACAGGCT	-14	GCTAAAAACG	-145	1,1	4.77	11.10	s	24	Rmet_1168	0	Op0333f_4	NA
508	TSS_3659038+2	741	159	0,1	-16	1	-49	TCTGCCTCAG	-14	GTTATACTTT	-143	5,1	9.60	-15.60	no	68	Rmet_3384	gspG	Op0941f_1	GIP-PTL-T2S
509	TSS_957148-2	741	49	0,1	-16	1	-37	TGGCATGTCC	-14	TGTAGAATTG	-137	1,1	8.68	8.90	s	19	Rmet_0875	cbbF3	Op0254r_3	MET-CAH-FbP
510	TSS_1489524+3	739	129	0,1	-16	1	-31	TTCCCTGCCC	-7	TTCACACTTT	-146	1,1	5.53	3.70	(m)-ba	63	Rmet_4835	0	Op1410f_1	NA
511	TSS_451290-2	739	120	0,1	-15	1	-40	GCAACCCCCT	-13	TCTACACTAC	-138	4,1	7.49	-1.60	no	21	Rmet_0426	ruvC	Op0116r_3	MET-NUC-Pur
512	TSS_3737122-2	739	83	0,1	-16	1	-40	TTGATATTGG	-14	GCTATACTTT	-137	1,1	10.66	6.40	(s)-sba	90	Rmet_3460	0	Op0962r_1	NA
513	TSS_3220745+2	738	153	0,4	-5	0	-42	CCGGCCAGTC	-12	GGTAAGCTGC	-142	5,1	9.69	-7.10	no	4	Rmet_2963	pntAa	Op0835f_1	MET-COF-NicAm
514	TSS_1160914-3	734	44	0,1	-15	1	-48	TGCGGACCAG	-13	AGTATGATGC	-144	4,1	6.77	-14.60	no	117	Rmet_4549	uvrA2	Op1305r_1	GIP-REP-Recomb
515	TSS_2618572-2	732	117	0,1	-15	1	-35	TTGACGAGCC	-13	GCAATAATCT	-140	1,1	8.78	14.20	s	125	Rmet_R0040	0	Op0678r_2	NA
516	TSS_2227649-2	724	151	0,1	-16	1	-34	TTGCCGTGAC	-14	CGTAACATGC	-140	1,1	9.32	9.60	s	74	Rmet_2055	ugpB	Op0596r_1	EIP-TRA-ABC
517	TSS_3160101+2	723	109	0,1	-17	1	-35	TTGCGGCGAC	-15	GGTACACTGT	-133	1,1	9.97	9.60	s	54	Rmet_2906	0	Op0821f_2	NA
518	TSS_3121284+2	723	125	0,1	-17	1	-37	GTAACAGTCT	-15	TGTAGACTTG	-140	4,1	7.98	8.90	s	40	Rmet_2872	nadB	Op0807f_1	MET-AA-AlaAsp
519	TSS_3733428-2	720	159	0,1	-15	1	-35	TCTGCAGGGT	-13	GCTACACTTC	-135	5,1	11.47	10.20	s	33	Rmet_3456	pbrR3	Op0962r_3	NA
520	TSS_3431487-2	715	200	0,1	-16	1	-42	TGGCCCTTAT	-14	CATACAATGG	-139	1,1	9.41	-0.10	no	358	Rmet_R0053	0	Op0886r_1	NA
521	TSS_814756+2	714	185	0,1	-16	1	-43	TTGCCGGCTA	-14	TATAAAATAG	-141	1,1	9.23	-0.60	no	40	Rmet_0737	etfB	Op0207f_3	NA
522	TSS_2858351+2	712	122	0,1	-17	1	-41	TTGCCCTTGG	-15	GATATACTGT	-138	1,1	10.58	4.40	(m)-sba	387	Rmet_2625	0	Op0735f_1	NA
523	TSS_2117434+2	709	98	0,1	-17	1	-45	GCACAAAAGG	-15	GGTAAAGTAT	-144	4,1	9.44	-10.60	no	48	Rmet_1947	ompR	Op0571f_1	EIP-SIG-2Comp
524	TSS_2619284-2	707	107	0,1	-15	1	-48	TTTCGGCCCG	-13	GCGACAAATCG	-139	2,1	11.50	-15.10	no	57	Rmet_2409	pgsA	Op0678r_1	MET-LIP-GlycPLip
525	TSS_935423+2	703	144	0,1	-16	1	-48	GTAGACGAAA	-14	TTTACAATAC	-141	4,1	8.45	-16.10	no	287	Rmet_0855	0	Op0245f_1	NA
526	TSS_3581317-2	703	110	0,1	-15	1	-34	TTGCCCTTTC	-13	GTTATAGTGT	-139	1,1	9.76	11.60	s	126	Rmet_3317	rplC	Op0926r_2	GIP-TL-Ribo
527	TSS_814806+2	701	87	0,1	-17	1	-38	TCAAAAGCGC	-15	GTTATAATGG	-140	4,1	12.66	9.40	s	812	Rmet_0738	etfA	Op0207f_3	NA
528	TSS_1580409-2	700	100	0,1	-17	1	-37	TTGCACGCGA	-15	AGTAGACTAA	-138	1,1	7.44	10.40	s	83	Rmet_1459	0	Op0408r_2	NA
529	TSS_3525592-2	699	141	0,1	-16	1	-43	TCGAGCAAAG	-14	CCTATAATTC	-136	1,1	9.36	-1.10	no	129	Rmet_3253	yadG	Op0918r_3	NA
530	TSS_1200934+3	699	213	0,1	-17	1	-36	TTCTAACGGG	-15	TATACCCTCG	-143	1,1	5.34	9.30	s	20	Rmet_4583	gltI	Op1318f_2	NA
531	TSS_518700-2	698	105	0,1	-15	1	-47	TCCGCGCCAC	-13	AGTATCATGT	-140	4,1	6.75	-12.60	no	131	Rmet_0490	0	Op0132r_1	NA
532	TSS_3126524-2	698	144	0,1	-16	1	-39	GCGGATGTCT	-14	GGTAGAATAT	-139	5,1	9.36	1.90	w	170	Rmet_2875	scd	Op0808r_2	DIV-Ass
533	TSS_608912-3	698	78	0,1	-6	0	-35	TTGACGACCA	-14	TGCACACTGT	-143	1,1	7.73	11.60	s	28	Rmet_4042	tniR	Op1139r_1	NA
534	TSS_1141359-3	696	56	0,1	-15	1	-48	TCGAAAAATC	-13	CGTAACCTGC	-140	1,1	6.84	-14.10	no	51	Rmet_4533	0	Op1297r_2	NA
535	TSS_308733-3	695	79	0,1	-16	1	-38	TTGCGGAAGG	-14	GGTATCATCA	-139	1,1	9.62	8.40	s	2236	Rmet_3769	alkB1	Op1047r_1	NA
536	TSS_1245102+2	694	86	0,1	-17	1	-39	GCAGCAATAG	-15	CGTACAATGT	-136	5,1	11.06	3.40	m	26	Rmet_1138	aroC	Op0325f_2	MET-AA-PheTyrTrp
537	TSS_1827228-2	690	178	0,1	-14	1	-48	TTCATGTCGT	-12	GATATTCTGG	-146	1,1	7.04	-15.10	no	75	Rmet_1686	0	Op0476r_2	NA
538	TSS_436593-2	690	174	0,1	-15	1	-38	TGGCCGGAGG	-13	CATATACTCA	-142	1,1	7.58	6.90	s	66	Rmet_0408	0	Op0112r_2	NA
539	TSS_580868-3	687	75	0,1	-15	1	-44	GCCCAGGCGC	-13	GCTACCGTTC	-138	4,1	7.70	-12.60	no	674	Rmet_4009	0	Op1125r_2	NA
540	TSS_1794306-2	684	32	0,1	-15	1	-40	TTGCGAGTCG	-13	CATAAAATTC	-142	1,1	9.80	4.40	(m)-sba	145	Rmet_1658	0	Op0468r_4	NA

541	TSS_3628285+2	684	102	0,1	-17	1	-38	TCGATGATGG	-15	GCTATAATTG	-136	1,1	9.97	9.90	s	113	Rmet_3352	0	Op0933f_1	NA
542	TSS_3831163+2	684	140	0,1	-16	1	-40	TTGCGGCCTG	-14	GCTAGACTAT	-144	1,1	8.11	3.40	(m)-sba	23	Rmet_3533	phhA	Op0977f_1	MET-AA-PheTyrTrp
543	TSS_1257859-2	683	161	0,1	-15	1	-34	TTGCCTGAGG	-13	CGGACAATGC	-142	1,1	8.20	10.60	s	183	Rmet_1146	etfD	Op0328r_1	NA
544	TSS_1087754+3	681	106	0,1	-17	1	-38	TTGTATAGAA	-15	GTTATTCTAC	-142	1,1	7.09	8.40	s	4714	Rmet_6669	0	Op2073f_1	NA
545	TSS_777891+3	681	104	0,1	-17	1	-41	TTGTGCTGCG	-15	GCTATGGTTT	-144	1,1	6.77	2.40	(w)-sba	213	Rmet_4193	0	Op1186f_1	NA
546	TSS_891425-2	680	90	0,1	-15	1	-35	TGCCGGGTTA	-13	GGTAAAATCG	-137	1,1	8.15	9.20	s	90	Rmet_0818	glnS	Op0230r_1	MET-AA-GluGSH
547	TSS_3167454+2	680	49	0,1	-18	1	-46	TCCCGGCCCC	-16	TGTAAAATCG	-137	1,1	6.92	-8.60	no	675	Rmet_2914	pcnB	Op0825f_2	GIP-TL-Ass
548	TSS_784790+3	678	83	0,1	-17	1	-48	TTTCGTCGCG	-15	GATACACTAA	-138	2,1	13.84	-13.10	no	24	Rmet_4200	0	Op1190f_1	NA
549	TSS_2387555+3	677	111	0,1	-16	1	-34	TTGCCCAAGA	-14	AATACAATAC	-140	1,1	9.71	10.60	s	16	Rmet_5631	0	Op1680f_1	NA
550	TSS_1367647-3	673	46	0,1	-16	1	-31	TGGAAGTGAT	-9	AATAGCGTCC	-144	1,1	4.78	5.70	(m)-ba	1566	Rmet_4723	0	Op1363r_1	NA
551	TSS_1653951+2	669	94	0,1	-16	1	-37	TTGATACAGT	-14	AGTAGAATTT	-142	1,1	9.88	12.40	s	22	Rmet_1533	hoxN	Op0427f_2	EIP-TRA-Ion
552	TSS_165563-4	668	74	0,1	-16	1	-35	TTGATCCCCG	-14	TATATGATTC	-140	1,1	8.27	12.60	s	3498	Rmet_6138	0	Op1876r_1	NA
553	TSS_250948-2	666	248	0,1	-16	1	-38	TTGCCACTGC	-14	AGTAGAATAG	-138	1,1	9.84	10.40	s	35	Rmet_0233	0	Op0068r_2	NA
554	TSS_3498731-2	664	87	0,1	-15	1	-38	TGGCCGGGGC	-13	GCTACACTCC	-137	1,1	9.06	5.90	m	3023	Rmet_6586	0	Op0908r_1	NA
555	TSS_960901+2	662	58	0,1	-16	1	-36	CCTGCCGCCG	-14	GCTAGAATGG	-137	5,1	11.56	7.40	s	35	Rmet_0878	maeB	Op0255f_2	MET-CAH-Pyr
556	TSS_2021620-2	661	110	0,1	-15	1	-34	TTGAGACACA	-13	AGTATTGTAC	-143	1,1	6.24	11.60	s	268	Rmet_1863	0	Op0538r_1	MET-LIP-FASyn
557	TSS_859996-2	660	104	0,1	-15	1	-44	TCGCCCCACG	-13	GGTACAATCA	-138	1,1	9.00	-5.10	no	68	Rmet_0783	hemF	Op0222r_1	MET-COF-Porph
558	TSS_2378732-3	658	161	0,1	-15	1	-35	TTGTCGTGGG	-13	TTTATACTGA	-141	1,1	9.04	13.20	s	34	Rmet_5621	0	Op1677r_1	NA
559	TSS_263958+2	658	63	0,1	-17	1	-36	TTGTTATCAT	-15	GATATTCTGC	-144	1,1	7.78	11.80	s	46	Rmet_0247	bfd	Op0071f_3	NA
560	TSS_2161645+2	657	126	0,2	-14	1	-31	AACTCTTTCA	-11	GTCGAAGCGC	-154	3,0	-0.05	-4.40	no	1554	Rmet_1990	0	Op0583f_1	NA
561	TSS_2483053+2	657	167	0,1	-15	1	-33	TGGACAGCCG	-13	GCTATAATCG	-136	1,1	10.49	11.10	s	6	Rmet_R0036	0	Op0645f_4	NA
562	TSS_3450062+2	655	47	0,1	-17	1	-46	TGGCAATCCT	-15	GGTATAGTCG	-139	1,1	8.80	-7.10	no	509	Rmet_3178	trpE	Op0893f_1	MET-AA-PheTyrTrp
563	TSS_785892-2	654	67	0,1	-2	0	-32	GGAACCGAAC	0	AGTACAATCC	-139	4,1	10.28	-4.80	no	36	Rmet_R0007	ssrA	Op0202r_2	NA
564	TSS_233681+4	654	11	0,1	-16	1	-35	TTGCCCCGCT	-14	CCTAGAATCG	-141	1,1	9.76	12.60	s	1160	Rmet_6062	parB	Op1847f_1	NA
565	TSS_3065779+2	653	116	0,1	-11	1	-29	TGGAAGAGGA	-9	GATATCTTCA	-142	1,1	6.21	3.10	(w)-nu	824	Rmet_2819	hemK1	Op0793f_1	NA
566	TSS_500350+3	653	109	0,1	-16	1	-36	TTGCCTTTTG	-14	GCTACCTTAC	-141	1,1	6.93	11.40	s	1432	Rmet_3944	0	Op1104f_1	GIP-REP-Recomb
567	TSS_362332-2	651	126	0,4	-16	1	-38	GGCGCCCTGC	-13	GCAACAATCG	-143	4,1	5.66	1.40	w	23	Rmet_0343	0	Op0096r_1	NA
568	TSS_401959+2	648	151	0,1	-17	1	-43	TTCCGGTCGG	-15	TTTATAATCA	-139	1,1	8.86	-1.10	no	116	Rmet_0376	0	Op0103f_5	NA
569	TSS_2312465-2	648	104	0,1	-16	1	-35	TCTGACCCAG	-14	GCTACAATCG	-138	5,1	10.82	8.60	s	0	Rmet_2129	dnaX	Op0614r_2	GIP-REP-DNA_Pol
570	TSS_110682+2	647	92	0,1	-16	1	-42	TGCCGCCATT	-14	TTTACAATCT	-140	1,1	7.38	-2.60	no	43	Rmet_0104	aceK	Op0031f_2	NA
571	TSS_3161819+2	647	64	0,1	-17	1	-38	TGCCGAATTC	-15	CGTATAATCG	-137	1,1	8.62	8.40	s	1983	Rmet_2909	miaA	Op0823f_1	GIP-TL-Ass
572	TSS_2509468+3	646	75	0,1	-20	0	-43	TTCCCGCGTC	-18	TACAGCGTTG	-145	1,1	2.70	-1.10	no	2279	Rmet_5751	0	Op1722f_1	NA
573	TSS_3885267+2	646	78	0,1	-16	1	-34	TGGCCGCCAC	-14	GCTAATGTTT	-143	1,1	5.52	7.10	s	200	Rmet_3581	0	Op0995f_2	NA
574	TSS_1602741-2	645	65	0,1	-16	1	-35	TGGTTTTAAT	-14	TTGACGCTTC	-150	1,1	3.08	7.10	s	0	Rmet_1481	ydcN	Op0418r_1	NA
575	TSS_1297787+3	645	120	0,1	-12	1	-35	TTGTGTATTA	-10	CATACAATAC	-145	1,1	8.23	9.20	s	8167	Rmet_4673	0	Op1352f_1	NA
576	TSS_1095052-2	644	95	0,1	-15	1	-34	TTGCTTTGCT	-13	ACTAAGATTC	-144	1,1	8.19	9.60	s	34	Rmet_1003	0	Op0286r_1	NA
577	TSS_3504190-2	644	110	0,1	-16	1	-36	TTGCTTTTCG	-14	GACAGAATGT	-141	2,1	11.23	11.40	s	74	Rmet_3227	sspA	Op0914r_1	GIP-PTL-Fold
578	TSS_2875676-2	642	25	0,1	-15	1	-40	TGGCGCCGAC	-13	CATAAAATGC	-138	1,1	8.58	1.90	(w)-sba	1601	Rmet_2637	0	Op0742r_1	NA
579	TSS_965181-2	641	110	0,1	-15	1	-35	TTGCGCAGTT	-13	CTTAGTATCC	-142	1,1	7.86	11.20	s	198	Rmet_0880	0	Op0256r_2	NA
580	TSS_991940-3	641	36	0,1	-16	1	-36	TCGACCGGAC	-14	GCTACAGTCC	-137	1,1	7.71	11.90	s	20434	Rmet_4372	0	Op1247r_1	NA
581	TSS_788450+2	640	116	0,1	-15	1	-46	TTCCCGCGGG	-13	GATAGAATGC	-136	1,1	9.31	-9.10	no	83	Rmet_0713	gyrA	Op0203f_1	GIP-REP-Complex
582	TSS_1157312-2	639	190	0,1	-15	1	-37	TGCCCGGATT	-13	GGTATGATGG	-139	1,1	8.12	7.40	s	30	Rmet_1063	trxB1	Op0306r_1	MET-NUC-Pyr
583	TSS_495487+2	638	89	0,1	-17	1	-36	TTGCCAAGCA	-15	TATACAGTAC	-137	1,1	8.02	12.80	s	144	Rmet_0466	recA	Op0123f_1	GIP-REP-Recomb
584	TSS_2233019+3	638	122	0,1	-16	1	-36	TTGCGTAGCA	-14	TGTATATTGG	-139	1,1	8.83	12.40	s	306	Rmet_5489	0	Op1630f_1	NA
585	TSS_783137+3	637	143	0,1	-26	0	-36	TTGACTTTAT	-10	CGTAAGATGC	-143	1,1	9.51	9.40	s	25	Rmet_4198	0	Op1190f_1	NA
586	TSS_2331834+3	637	135	0,1	-16	1	-36	TTGAGAATGG	-14	GTTAGGATGC	-135	1,1	9.75	13.40	s	34	Rmet_5578	0	Op1660f_1	NA
587	TSS_646721-3	637	170	0,1	-15	1	-35	TTGAGCCGCG	-13	GGTATCGTAG	-144	1,1	6.96	12.20	s	86	Rmet_4077	0	Op1145r_1	NA
588	TSS_732887-2	635	100	0,1	-17	1	-41	TTGACGATGT	-15	GCTACACTTC	-135	1,1	10.98	4.40	(m)-sba	30	Rmet_0665	lemA	Op0184r_1	NA
589	TSS_1201942+3	634	92	0,1	-16	1	-35	TTGACGCCCC	-14	GACACACTGG	-138	1,1	7.93	11.60	s	82	Rmet_4584	0	Op1318f_3	NA
590	TSS_1757588+2	630	154	0,1	-16	1	-35	TCCTGTTCTC	-14	AGTACAATCT	-142	1,1	5.86	8.60	s	1011	Rmet_1630	0	Op0457f_1	NA
591	TSS_2235855-2	629	94	0,1	-14	1	-44	GCGGAAAGGT	-12	GCTAACATTC	-140	5,1	10.83	-10.10	no	89	Rmet_2062	glnA	Op0598r_3	MET-EN-Nitrog
592	TSS_3360881-2	627	170	0,1	-15	1	-38	TCGAGCATGA	-13	TTTAGAATTA	-142	1,1	8.07	6.90	s	30	Rmet_3100	proS	Op0872r_1	MET-AA-ArgPro
593	TSS_112281-5	624	95	0,1	-15	1	-42	GTCAAGAAAC	-13	GCTACAATCT	-138	4,1	11.49	-4.10	no	2180	Rmet_6257	0	Op1905r_1	NA
594	TSS_2063476-3	621	23	0,1	-16	1	-36	GCAGACGGAG	-14	CGTAGAATCC	-138	5,1	10.20	6.40	s	77	Rmet_5343	0	Op1585r_1	NA
595	TSS_3090961-2	621	88	0,5	-15	1	-34	TTGACGGCGA	-13	GTCTCAATGA	-143	1,1	2.71	9.60	s	445	Rmet_2842	tnpA	Op2044r_1	NA
596	TSS_211786+2	620	40	0,1	-17	1	-38	GCAAACATAC	-15	TGTATAATGC	-142	4,1	12.66	5.40	m	165	Rmet_0203	0	Op0059f_3	NA
597	TSS_2138+2	619	201	0,1	-15	1	-35	TTCACCGCAG	-13	GGTACAATGC	-137	1,1	10.16	12.70	s	81	Rmet_0002	dnaN	Op0001f_2	GIP-REP-DNA_Pol
598	TSS_66429-3	617	57	0,1	-20	0	-41	TTGATGCTGG	-18	TTTAAAGTAC	-142	1,1	7.11	6.40	(s)-sba	35	Rmet_5870	0	Op1777r_1	NA
599	TSS_3158444-2	615	39	0,1	-15	1	-44	TTCCTGCGCG	-13	GGTATTATAC	-137	2,1	12.08	-6.10	no	241	Rmet_2904	rpsT	Op0820r_1	GIP-TL-Ribo
600	TSS_4852-4	615	36	0,1	-15	1	-34	TTGACTCATT	-13	GAGATATTAC	-145	1,1	6.58	11.60	s	29	Rmet_6060	repA	Op1846r_1	NA
601	TSS_3561066-2	614	145	0,1	-15	1	-39	TACGGTCTCC	-13	GGCATCCTTG	-146	2,1	6.47	0.40	w	169	Rmet_3284	0	Op0924r_1	NA

602	TSS_298217+2	612	133	0,1	-16	1	-36	TTGGGAAAGG	-14	GGTAGAATCA	-145	1,1	4.03	13.40	s	24	Rmet_0282		*0*	Op0083f_2	NA
603	TSS_1501666+2	610	25	0,1	-16	1	-46	TCAAAAGCCA	-14	CTCATAATAC	-142	4,1	6.81	-8.60	*no*	488	Rmet_1393		*0*	Op0389f_1	NA
604	TSS_1168604+3	609	56	0,1	-16	1	-37	TTGCATCGCG	-14	AGTAATATAC	-140	1,1	7.33	9.40	s	17	Rmet_4558		*0*	Op1308f_1	NA
605	TSS_3126570-2	609	61	0,1	-13	1	-34	TTGAACGTTT	-11	CTCAAAGTCG	-140	1,1	6.15	9.20	s	216	Rmet_2875	*scd*		Op0808r_2	DIV-Ass
606	TSS_3427431-2	608	76	0,1	-16	1	-35	TTCCTCCGGC	-14	GATATGTTTC	-143	1,1	6.04	9.10	s	121	Rmet_3161		*0*	Op0886r_2	NA
607	TSS_405397-3	607	118	0,1	-15	1	-34	TTGCGCCGCA	-13	TCTATCGTCG	-137	1,1	6.49	9.60	s	74	Rmet_3861		*0*	Op1073r_2	NA
608	TSS_877359+3	606	75	0,1	-15	1	-35	TTCACAAATA	-13	GTCACAATAC	-141	1,1	8.16	12.70	s	6125	Rmet_4292	*prpR*		Op1220f_1	NA
609	TSS_1091957-2	606	88	0,1	-20	0	-44	GGCCCAGTGC	-18	GGTATGGTCT	-142	4,1	7.05	-4.60	*no*	998	Rmet_0998	*recN*		Op0284r_1	GIP-REP-Recomb
610	TSS_230079-4	605	19	0,1	-15	1	-36	TGGCCCGGCT	-13	GTTATCCTGT	-140	1,1	7.62	9.90	s	1342	Rmet_6072		*0*	Op1850r_1	NA
611	TSS_44446+2	604	77	0,1	-17	1	-38	GTACGCATGG	-15	GGCACAATCA	-140	4,1	7.62	3.90	m	2864	Rmet_0042	*kdpE*		Op0015f_3	EIP-SIG-2Comp
612	TSS_503367+2	600	114	0,1	-16	1	-35	TCGGCACCCC	-14	GCTATCATGT	-137	5,1	10.96	12.10	s	20	Rmet_0474		*0*	Op0123f_4	NA
613	TSS_2950752+2	600	76	0,1	-16	1	-42	TTGTGCCGGA	-14	TTTATAATCC	-137	1,1	9.78	1.40	*(w)-sba*	80	Rmet_2717		*0*	Op0769f_1	NA
614	TSS_2631643-2	599	157	0,1	-15	1	-35	GGACGGAAGT	-13	GGTAAAATCG	-137	4,1	11.26	6.20	m	88	Rmet_2421	*lepA*		Op0680r_4	NA
615	TSS_785015+2	598	61	0,1	-16	1	-49	TTGCGTGAAT	-27	TTTAAGTTTT	-145	1,1	6.26	-2.60	*no*	66	Rmet_0709		*0*	Op0201f_1	NA
616	TSS_3150024+2	598	94	0,1	-15	1	-34	TTGACAGTAT	-13	GAAAGAATCG	-139	1,1	7.95	12.60	s	264	Rmet_2894	*cspE*		Op0813f_1	NA
617	TSS_2064999+3	597	51	0,1	-17	1	-42	CCTGCAACAG	-15	TGTATTCTCT	-141	5,1	8.91	-3.60	*no*	54	Rmet_5346		*0*	Op1588f_1	NA
618	TSS_770356-3	597	55	0,1	-14	1	-33	TTCCCGACGG	-12	GATAGGGTTA	-145	1,1	4.96	7.10	s	26	Rmet_4186		*0*	Op1183r_1	NA
619	TSS_1714727-2	594	103	0,1	-15	1	-34	TCGCCCCGAC	-13	TCTATGATTG	-140	1,1	8.07	10.10	s	28	Rmet_1589		*0*	Op0440r_2	NA
620	TSS_2357509-2	594	116	0,1	-18	1	-41	TTTCGTAGGG	-16	TCTACGATAT	-145	2,1	11.11	1.90	*(w)-sba*	87	Rmet_6509		*0*	Op2029r_1	NA
621	TSS_431339+2	592	95	0,1	-16	1	-37	GCGGCAGGGG	-14	AATAGAATGC	-137	5,1	11.27	7.90	s	152	Rmet_0404	*ttuC*		Op0111f_1	MET-CAH-But
622	TSS_563649+3	588	143	0,1	-17	1	-43	TGGACTGGGC	-15	TGTACGATAT	-141	1,1	7.36	-1.10	*no*	32	Rmet_3994		*0*	Op1120f_1	NA
623	TSS_1182970+2	586	64	0,1	-16	1	-35	TTGACCAGTA	-14	GCTAGAATAC	-138	1,1	10.58	13.60	s	34	Rmet_R0014		*0*	Op0313f_3	NA
624	TSS_759811-3	585	49	0,1	-15	1	-49	TTGAACGACA	-13	GATACAATGG	-136	1,1	10.90	-13.60	*no*	23	Rmet_4172		*0*	Op1175r_2	NA
625	TSS_487426-2	583	576	0,1	-15	1	-47	TTGACCTAAG	-13	GCCATGATGG	-144	1,1	8.92	-9.60	*no*	39	Rmet_0458	*uspA1*		Op0120r_1	NA
626	TSS_3416557-2	581	41	0,2	-16	1	-41	GCGGAACGCG	-14	AATACAATCG	-138	5,1	10.79	-1.10	*no*	167	Rmet_3151		*0*	Op0882r_2	NA
627	TSS_189034-2	579	70	0,1	-16	1	-40	TGCCCATAGT	-14	GCTACAATTC	-139	1,1	9.84	3.40	*(m)-sba*	0	Rmet_0181		*0*	Op0054r_1	NA
628	TSS_1272950+3	578	53	0,1	-25	0	-44	TCCTACTAAC	-23	CCGATTCTGC	-147	1,1	2.24	-1.20	*no*	4214	Rmet_4647		*0*	Op1342f_1	NA
629	TSS_2380133+2	577	85	0,1	-16	1	-47	GTACGGACGC	-14	AGTACCATAC	-139	4,1	8.51	-15.10	*no*	22	Rmet_6512		*0*	Op0625f_2	NA
630	TSS_355652-2	577	38	0,1	-15	1	-45	TGGCAACAGG	-13	ATTACCATTT	-139	1,1	7.27	-8.10	*no*	20	Rmet_0337		*0*	Op0094r_1	NA
631	TSS_13858-4	574	87	0,1	-15	1	-33	TTGACACGTG	-13	GGTATAATTG	-138	1,1	12.30	12.60	s	39	Rmet_6054		*0*	Op1844r_4	NA
632	TSS_3920782-2	573	47	0,1	-27	0	-34	TTGACTTGGC	-13	CAGACCATCC	-140	1,1	6.33	10.60	s	51	Rmet_3608		*0*	Op1004r_2	NA
633	TSS_2796468+2	572	70	0,1	-17	1	-37	GTCCAAACGG	-15	GATAAAATGC	-140	4,1	10.74	7.90	s	595	Rmet_2572	*mogA*		Op0713f_1	MET-COF-Folate
634	TSS_475503-2	572	134	0,1	-15	1	-39	TTGCGCGGGG	-13	GATAGAATTC	-137	1,1	10.23	5.40	m	63	Rmet_0448	*glyQ*		Op0118r_3	MET-AA-GlySerThr
635	TSS_1894677-2	571	23	0,1	-16	1	-35	TTGCCTGGCG	-14	TTTAAGCTAA	-142	1,1	6.21	10.60	s	138	Rmet_1747		*0*	Op0498r_2	NA
636	TSS_1297768-3	570	95	0,1	-15	1	-36	TTCCATGTGT	-13	TGTATGATAA	-145	1,1	7.44	9.90	s	126	Rmet_4664		*0*	Op1349r_1	NA
637	TSS_241163-2	569	119	0,1	-16	1	-44	TCGTGTTACG	-14	CGTATAATTC	-138	1,1	8.19	-4.10	*no*	517	Rmet_0226		*0*	Op0066r_1	NA
638	TSS_459260+3	569	136	0,1	-16	1	-36	TTGCAGAGCT	-14	GTTACCATGA	-136	1,1	9.42	11.40	s	19	Rmet_3911		*0*	Op1094r_2	NA
639	TSS_2063461+3	569	38	0,1	-16	1	-48	GCCGGGGCGC	-14	GCTATCATCC	-139	4,1	9.84	-17.60	*no*	33	Rmet_5344		*0*	Op1586f_1	NA
640	TSS_2380172+3	569	133	0,1	-16	1	-35	CCTGCACGCC	-14	GACAGAATGG	-137	5,1	10.08	6.60	s	29	Rmet_5624		*0*	Op1678f_1	NA
641	TSS_3233458-2	569	173	0,1	-15	1	-45	TTCCGTGCGA	-13	GTTATAATCG	-136	1,1	9.45	-7.10	*no*	76	Rmet_2973	*leuS*		Op0836r_1	MET-AA-ValLeuIle
642	TSS_3710506-2	568	15	0,1	-15	1	-49	GTAACAAAAT	-13	GTTAGACTTC	-144	4,1	10.37	-17.10	*no*	2	Rmet_3432		*0*	Op0952r_1	NA
643	TSS_788219-2	567	87	0,1	-15	1	-48	TCGAATATAT	-13	GGCACAATTC	-143	1,1	8.47	-14.10	*no*	132	Rmet_0712	*ompA*		Op0202r_2	EIP-TRA-Pores
644	TSS_2707640+2	566	416	0,1	-16	1	-42	TCGCGTAAGT	-14	GCCAGACTCG	-142	1,1	6.10	-3.10	*no*	46	Rmet_2493		*0*	Op0689f_2	NA
645	TSS_447500-2	565	49	0,1	-15	1	-34	TTGCTGGCCT	-13	TCCACAATGG	-141	1,1	8.13	9.60	s	45	Rmet_0422		*0*	Op0116r_4	GIP-REP-Recomb
646	TSS_527162+2	564	153	0,1	-15	1	-33	TTACCAGCGG	-13	CCTATAATGG	-140	4,1	8.89	10.10	s	97	Rmet_0497		*0*	Op0133f_2	NA
647	TSS_2879614+2	564	94	0,1	-16	1	-46	GCGGCAACGC	-14	GCTATACTCG	-137	5,1	11.71	-10.10	*no*	51	Rmet_2642	*pdxH*		Op0745f_2	MET-COF-B6
648	TSS_1715616+2	564	107	0,1	-16	1	-37	TTCCCCATTG	-14	GGCAAAATGC	-143	1,1	8.51	8.90	s	1634	Rmet_1593		*0*	Op0443f_1	NA
649	TSS_3412256-2	564	132	0,1	-27	0	-32	TTGTCTCGAT	-11	TTTAGCATTG	-143	1,1	7.43	8.60	s	38	Rmet_3148	*fadD*		Op0882r_3	MET-LIP-FAMet
650	TSS_2655232+2	563	74	0,2	-5	0	-36	CCGGAACCAT	-8	TACCGAATAC	-144	5,1	3.07	-0.10	*no*	363	Rmet_2444		*0*	Op0683f_2	NA
651	TSS_309285-2	563	94	0,1	-15	1	-38	GGCAAGCAGC	-13	GCTACAATGC	-137	4,1	11.12	2.40	w	39	Rmet_0292		*0*	Op0084r_1	NA
652	TSS_298204+2	562	49	0,1	-3	0	-48	TTGCGCTTCT	-1	GGTAGAATCA	-139	1,1	10.41	-24.60	*no*	37	Rmet_0282		*0*	Op0083f_2	NA
653	TSS_3893127+2	562	96	0,1	-16	1	-42	CGGCGGGTGT	-14	CCTATAATGG	-138	1,1	5.06	-3.10	*no*	82	Rmet_3586		*0*	Op0997f_1	EIP-TRA-ABC
654	TSS_2560229-3	561	164	0,1	-10	1	-38	TGGCCGGAAC	-8	TCAATAGTTG	-140	1,1	4.72	0.90	*(w)-ba*	1570	Rmet_5800		*0*	Op1747r_1	MET-CAH-PP_KDPG
655	TSS_3498809+2	560	98	0,1	-16	1	-45	CCGGCCACAC	-14	AGTACAATAT	-137	5,1	10.71	-9.10	*no*	31	Rmet_3222		*0*	Op0911f_1	NA
656	TSS_1325403-2	558	80	0,1	-14	1	-32	TTGAAATTTT	-12	GATAGAATCA	-141	1,1	10.77	9.60	s	36	Rmet_1205		*0*	Op0350r_1	NA
657	TSS_2986728+2	557	76	0,1	-19	0	-35	TGGACACATC	-14	AGTATTGTTG	-143	1,1	6.07	12.10	s	248	Rmet_2750	*ppc*		Op0775f_1	MET-CAH-Pyr
658	TSS_949896-3	557	57	0,1	-15	1	-39	TTTCGTTTCG	-13	GCTACGATCC	-137	2,1	14.37	2.90	w	752	Rmet_4353		*0*	Op1241r_2	NA
659	TSS_3867614-2	556	77	0,1	-15	1	-34	TTGAATTTTT	-13	CCGATTCTGC	-141	1,1	6.00	9.60	s	63	Rmet_3565		*0*	Op0988r_1	NA
660	TSS_1693375+2	556	204	0,1	-16	1	-44	TTGCAGGTCT	-14	GGGAAAATTC	-137	1,1	9.12	-4.60	*no*	20	Rmet_1574	*ldcA*		Op0433f_2	NA
661	TSS_3356616+2	554	95	0,1	-17	1	-39	TTGCCTTTCT	-15	TTTAGAATCG	-139	1,1	10.01	8.40	s	4296	Rmet_3101	*nudH*		Op0873f_1	NA
662	TSS_2011613-2	554	28	0,1	-15	1	-34	TTGAACGACC	-13	TGTATGCTTG	-141	1,1	8.56	10.60	s	130	Rmet_1855	*fadB*		Op0536r_1	MET-CAH-But

663	TSS_2621435-2	550	74	0,1	-15	1	-38	GCGGAAAGTC	-13	TCTAAACTTG	-140	5,1	9.95	2.90	w	53	Rmet_2410	*uvrC*	Op0678r_1	GIP-REP-Recomb
664	TSS_3037744+2	549	75	0,1	-16	1	-34	TGGCCGAGCC	-14	GCTAGAATCC	-136	1,1	9.73	9.10	s	35	Rmet_2790	*0*	Op0785f_1	NA
665	TSS_2545975-3	547	34	0,1	-15	1	-39	TCGCCAAGCA	-13	GCTAACCTGT	-139	1,1	7.13	3.90	m	30	Rmet_5788	*0*	Op1741r_1	NA
666	TSS_2377792-2	545	89	0,1	-15	1	-44	TCGGAAGCT	-13	GCTAAGCTGG	-139	5,1	9.69	-7.10	*no*	21	Rmet_2172	*int*	Op0624r_1	NA
667	TSS_166114-3	545	96	0,1	-15	1	-45	TCTACCCTAT	-13	TATACAATAG	-140	5,1	7.70	-7.60	*no*	58	Rmet_3629	*0*	Op1011r_2	NA
668	TSS_1506700-3	544	75	0,1	-16	1	-40	TGGCCGGGAG	-14	GGTATACTTC	-136	1,1	9.61	3.90	*(m)-sba*	10749	Rmet_4838	*0*	Op1411r_1	NA
669	TSS_3685381+2	544	68	0,1	-16	1	-35	TGGCGCAACG	-14	GCAATAATAC	-141	1,1	6.65	10.10	s	128	Rmet_3407	*0*	Op0947f_1	MET-LIP-Others
670	TSS_1928501+3	540	260	0,1	-15	1	-36	TTGAGCACGA	-13	ACTAAACTTT	-140	1,1	8.69	11.40	s	24	Rmet_5231	*0*	Op1544f_1	NA
671	TSS_267707-2	536	104	0,1	-16	1	-46	TGGCGGCCCG	-14	CTTATACTGT	-138	1,1	7.43	-9.10	*no*	72	Rmet_0250	*ctpA*	Op0072r_1	NA
672	TSS_472637-3	535	79	0,1	-15	1	-33	GTAAAAGAAT	-13	ATTAGAATCG	-141	4,1	10.54	6.10	m	0	Rmet_3922	*ydjQ*	Op1097r_2	GIP-REP-DNA_Pol
673	TSS_1461952-2	535	100	0,1	-15	1	-41	TCGCCGTGAG	-13	TGTACAGTGG	-139	1,1	7.34	-0.10	*no*	175	Rmet_1352	*comL*	Op0380r_1	NA
674	TSS_3403505+2	535	25	0,1	-17	1	-45	TTACCACAGG	-15	GTTAGAGTGA	-148	4,1	5.47	-5.10	*no*	44	Rmet_3139	*0*	Op0879f_1	NA
675	TSS_1152532-2	533	46	0,1	-15	1	-41	TTTCGGCGGG	-13	CGTAAAATCC	-135	2,1	13.67	-0.10	*no*	108	Rmet_1059	*paaY*	Op0302r_1	NA
676	TSS_51206-4	533	87	0,4	-16	1	-34	TTGCATCGGC	-13	GTTTGAATAC	-143	1,1	3.71	8.60	s	715	Rmet_6007	*0*	Op1832r_1	NA
677	TSS_3154627-2	533	73	0,1	-15	1	-44	TCGAACCCGA	-13	GATAGAATTC	-139	1,1	8.74	-5.10	*no*	145	Rmet_2899	*0*	Op0816r_1	NA
678	TSS_1858445+3	533	37	0,1	-16	1	-35	GCCCCAGCAC	-14	GCTATGCTGG	-137	4,1	9.37	6.60	s	38	Rmet_5159	*0*	Op1518f_1	NA
679	TSS_1850192+2	532	125	0,1	-15	1	-45	TTGCAGAATC	-13	TTTACCATAA	-141	1,1	7.97	-7.60	*no*	84	Rmet_1708	*0*	Op0483f_1	NA
680	TSS_22805+2	531	5	0,5	-22	0	-49	TTGATCACCG	-15	ATTACTTTCG	-147	1,1	5.22	-13.60	*no*	47	Rmet_0019	*0*	Op0003f_3	NA
681	TSS_3542389-2	531	194	0,1	-14	1	-32	GGACCAGCGC	-12	TGCACAATGG	-139	4,1	9.46	2.60	w	669	Rmet_3266	*aroB*	Op0920r_2	MET-AA-PheTyrTrp
682	TSS_1684461-3	531	65	0,1	-15	1	-34	TTGACCTGCC	-13	GGTAACCTTG	-139	1,1	8.83	10.60	s	22	Rmet_5003	*0*	Op1467r_1	NA
683	TSS_2163228-2	530	36	0,1	-16	1	-42	TTGCTTCAGG	-14	CATATACTTC	-140	1,1	8.52	0.40	*(w)-sba*	114	Rmet_1989	*dus*	Op0582r_1	GIP-TL-Ass
684	TSS_2030010+3	530	145	0,1	-16	1	-35	TGGCCTGGCC	-14	CATAGAATCC	-139	1,1	7.95	11.10	s	54	Rmet_5321	*zniC*	Op1572f_1	NA
685	TSS_1714034-2	529	61	0,1	-16	1	-35	TTGCGAAGGC	-14	GGTACTGTTG	-138	1,1	7.83	10.60	s	3063	Rmet_1587	*prpB*	Op0438r_1	MET-CAH-Prop
686	TSS_2709910+2	529	67	0,1	-5	0	-35	TTGTACTGGG	-15	TGTATAGTTA	-141	1,1	8.07	10.60	s	4	Rmet_2496	*0*	Op0691f_1	EIP-TRA-ABC
687	TSS_126686+3	528	95	0,1	-17	1	-43	TTGCCGTTTG	-15	GTCACAATTC	-139	1,1	9.60	-0.60	*no*	89	Rmet_5926	*argG*	Op1800f_1	MET-AA-AlaAsp
688	TSS_2463903-2	527	136	0,1	-16	1	-46	TGACAAGCGT	-14	GCTAGGATGG	-138	4,1	8.89	-10.60	*no*	25	Rmet_2248	*0*	Op0644r_1	NA
689	TSS_1955997+2	526	92	0,1	-17	1	-37	TTGACGGATG	-15	CATAAATTCC	-139	1,1	7.75	12.40	s	646	Rmet_1804	*0*	Op0523f_2	NA
690	TSS_2659855-2	526	62	0,1	-15	1	-39	TTGAGACCTT	-13	TTTACACTGG	-138	1,1	9.49	6.40	s	27	Rmet_2447	*0*	Op0684r_1	NA
691	TSS_3633099+2	524	34	0,1	-8	0	-36	TTGACGCGCT	-6	AGCAAAATGA	-143	1,1	8.38	5.40	*(m)-ba*	752	Rmet_3358	*0*	Op0935f_3	NA
692	TSS_64803+2	524	82	0,1	-17	1	-44	TTGATACAAA	-15	GCTACACTGC	-141	1,1	10.09	-1.60	*no*	22	Rmet_0056	*0*	Op0017f_2	NA
693	TSS_810975+2	524	84	0,1	-16	1	-38	GTAGAAGCGC	-14	GCTAACGTAG	-140	4,1	7.68	2.90	w	27	Rmet_0733	*0*	Op0207f_1	NA
694	TSS_3318577+2	523	72	0,1	-16	1	-40	TCGAAGGCCC	-14	GGTATCATCG	-136	1,1	8.28	3.90	*(m)-sba*	765	Rmet_3062	*0*	Op0861f_2	NA
695	TSS_2174110+3	523	95	0,1	-16	1	-36	CCGGCAGCAG	-14	GGTATCATCC	-135	5,1	11.44	9.90	s	345	Rmet_5426	*0*	Op1614f_2	NA
696	TSS_1312266+2	523	52	0,1	-16	1	-36	TTGTCAGCAC	-14	CATAGAATCG	-145	1,1	8.76	14.40	s	212	Rmet_1196	*aceE*	Op0347f_2	MET-CAH-FbP
697	TSS_3402219-2	523	10	0,1	-16	1	-39	TTGTGTGAAA	-14	GCGATGATTT	-145	1,1	6.33	5.40	m	49	Rmet_3137	*mraZ*	Op0878r_2	NA
698	TSS_2245575+2	521	41	0,1	-17	1	-35	TTGTGCTGAC	-15	ATTATAATCC	-135	1,1	9.81	11.60	s	1466	Rmet_2071	*yggX*	Op0601f_2	NA
699	TSS_2502871+2	519	125	0,1	-17	1	-36	TTGTAATGAT	-15	TTTATGATCA	-142	1,1	8.89	12.80	s	40	Rmet_2280	*hemP*	Op0651f_3	NA
700	TSS_2140250-2	519	75	0,1	-16	1	-36	TCGGCCGGTC	-14	GCTACAATCG	-136	5,1	11.76	11.90	s	84	Rmet_1964	*moeA*	Op0574r_1	MET-COF-Folate
701	TSS_2238508-2	518	54	0,1	-15	1	-37	CCGGAAACAC	-13	GCTAGGCTCC	-139	5,1	8.73	3.90	m	22	Rmet_2065	*0*	Op0600r_2	NA
702	TSS_570664-2	516	305	0,1	-15	1	-35	TTGCGACAAG	-13	CCCATAATGT	-136	1,1	8.95	13.20	s	54	Rmet_0535	*lpxK*	Op0148r_1	MET-GLYC-LPS
703	TSS_2278801-2	515	42	0,1	-16	1	-35	TCGACTGGCG	-14	GAGAAAATCC	-139	1,1	6.56	11.10	s	397	Rmet_2097	*hisZ*	Op0608r_3	GIP-TL-Ass
704	TSS_9289+5	514	132	0,1	-16	1	-37	TGGCTTGACA	-14	GATACAATGG	-139	1,1	8.71	8.90	s	590	Rmet_6393	*0*	Op1920f_2	NA
705	TSS_3365635-2	514	117	0,1	-15	1	-46	TTGCCGTGGC	-13	TCTATAATCC	-138	1,1	11.64	-6.60	*no*	314	Rmet_3106	*rplU*	Op0874r_1	GIP-TL-Ribo
706	TSS_3500775+2	514	78	0,1	0	0	-36	TTGCCTCCGT	-12	TCGATTGTGA	-146	1,1	4.27	9.40	s	99	Rmet_3225	*0*	Op0913f_1	NA
707	TSS_108797-2	513	101	0,1	-14	1	-34	TCGTTGCGCA	-12	TCTAAGATGA	-144	1,1	5.31	8.70	s	69	Rmet_0101	*0*	Op0030r_1	NA
708	TSS_3467479-2	512	150	0,1	-15	1	-36	GCGGATGGAG	-13	GCTATAATTC	-135	5,1	11.52	7.90	s	1420	Rmet_3190	*dsbC*	Op0896r_1	GIP-PTL-Fold
709	TSS_9775+5	512	58	0,1	-17	1	-49	TTCTATATGA	-15	TTTACAATAG	-143	1,1	7.72	-14.10	*no*	104	Rmet_6393	*0*	Op1920f_2	NA
710	TSS_1097655-2	511	4	0,1	-16	1	-41	TCGTCACATG	-14	GGTACGCTTG	-139	1,1	6.44	0.90	*(w)-sba*	2637	Rmet_1003	*0*	Op0286r_1	NA
711	TSS_3050376-2	511	40	0,1	-16	1	-37	GCCGAAGCGC	-14	GACATCATTA	-139	4,1	8.46	4.40	m	352	Rmet_2802	*lgt*	Op0788r_2	NA
712	TSS_3683457+2	510	82	0,1	-16	1	-36	TTTCTTTCCG	-14	GGTAAGCTTG	-140	2,1	12.17	8.90	s	119	Rmet_3405	*0*	Op0945f_2	NA
713	TSS_1271827+2	509	32	0,1	-18	1	-37	TTGCGTAACG	-16	TATAAAATTC	-142	1,1	9.73	10.80	s	261	Rmet_1161	*infC*	Op0333f_3	NA
714	TSS_2273641-2	506	46	0,1	-16	1	-47	TGGCGAGATA	-14	GATACACTCA	-141	1,1	7.87	-11.10	*no*	266	Rmet_2092	*mr*	Op0606r_1	NA
715	TSS_458129-3	506	9	0,1	-16	1	-41	TCGCACACGT	-14	GCTACAGTGA	-139	1,1	7.47	-0.10	*no*	49	Rmet_3908	*0*	Op1093r_1	NA
716	TSS_148041-3	505	102	0,1	-15	1	-37	CTCAATGGGG	-13	TATAGAATCT	-149	1,1	3.32	5.90	m	38	Rmet_3616	*0*	Op1005r_1	GIP-PTL-Fold
717	TSS_3784310-2	505	29	0,1	-16	1	-48	GCAGCCCGCG	-14	GCTATATTTC	-140	5,1	10.51	-16.60	*no*	621	Rmet_3492	*0*	Op0970r_2	MET-CAH-But
718	TSS_291990-3	504	58	0,1	-16	1	-35	TGGTCAGATG	-14	GTTAGCATTG	-144	1,1	7.09	11.10	s	21	Rmet_3747	*0*	Op1043r_1	NA
719	TSS_291140-2	502	73	0,1	-16	1	-49	TTGACTCACG	-14	GCTATCATGA	-137	1,1	9.45	-11.60	*no*	23	Rmet_0274	*0*	Op0080r_3	MET-AA-PheTyrTrp
720	TSS_735652-2	501	75	0,1	-16	1	-42	TTCCATATGC	-14	ATTAAAATCA	-142	1,1	8.26	-1.10	*no*	208	Rmet_0667	*hemL*	Op0186r_1	MET-COF-Porph
721	TSS_3623890+2	500	83	0,1	-17	1	-38	TTGCGCACAT	-15	GCTAGAGTTA	-142	1,1	8.09	8.40	s	4508	Rmet_3352	*0*	Op0933f_1	NA
722	TSS_573202-3	500	66	0,1	-15	1	-34	TTGCATGACT	-13	CATACAATAA	-139	1,1	8.78	10.60	s	56	Rmet_4001	*rpoK*	Op1123r_1	GIP-TK-RNAP
723	TSS_2000168-3	500	85	0,1	-16	1	-35	TTGATCTGGC	-14	TCTACAATCA	-141	1,1	10.04	12.60	s	72	Rmet_5296	*acnB*	Op1565r_1	MET-CAH-TCA

724	TSS_3927637-2	498	84	0,1	-25	0	-35	TTGACAGGCT	-14	GGTTAAATGG	-140	1,1	5.78	12.60	s	1024	Rmet_3614	*0*	Op1004r_1	NA
725	TSS_1873559+2	497	65	0,1	-20	0	-24	AGATTTGTTA	-4	CGGTATATGC	-143	3,0	7.38	-10.40	*no*	746	Rmet_6474	*0*	Op0489f_1	NA
726	TSS_2517-3	497	60	0,1	-15	1	-35	TTGAAAAGCG	-13	CCTAAAGTAG	-140	1,1	7.91	13.20	s	171	Rmet_5818	*csp*	Op1757r_1	NA
727	TSS_6802+2	497	51	0,1	-16	1	-49	TTTCGTCGCT	-14	GGTATTCTGT	-140	2,1	11.95	-16.10	*no*	187	Rmet_0004	*0*	Op0001f_2	NA
728	TSS_507285+2	497	67	0,1	-17	1	-48	TTCATGCGCT	-15	GGCATAATGT	-141	1,1	8.30	-11.10	*no*	30	Rmet_0479	*0*	Op0125f_1	NA
729	TSS_30839+5	496	105	0,1	-16	1	-40	TTGTGCGGAA	-14	GCTACCATAC	-140	1,1	7.87	3.40	*(m)-sba*	24	Rmet_6343	*tnpR*	Op1928f_1	NA
730	TSS_377775+3	495	50	0,1	0	0	-39	TGAAAGGTAT	-18	TTCAGGCTGA	-148	4,1	3.86	3.80	m	181	Rmet_3836	*hmvC*	Op1066f_2	EIP-TRA-Ion
731	TSS_3416589+2	495	65	0,1	-16	1	-34	TTTGGCTCCG	-14	ATTATGATGT	-140	2,1	10.59	8.10	s	121	Rmet_3153	*0*	Op0883f_2	NA
732	TSS_905935+2	494	113	0,1	-16	1	-35	TGCCCCGGGA	-14	CGTATAATCA	-137	1,1	7.94	10.60	s	28	Rmet_0831	*alaS*	Op0237f_1	MET-AA-AlaAsp
733	TSS_455641-3	494	61	0,1	-15	1	-34	TTGAAATACA	-13	ATTACTATCT	-143	1,1	8.44	11.60	s	251	Rmet_3907	*0*	Op1093r_1	NA
734	TSS_768306+3	493	192	0,1	-19	0	-45	TTTCGGAGGG	-17	GCTCCGTTAG	-147	2,1	3.89	-8.10	*no*	63	Rmet_4185	*cheD*	Op1182f_2	DIV-MOT-Chemotax
735	TSS_1032959+2	492	70	0,1	-17	1	-24	TTGCGTTGCG	-3	CCCACGATGT	-143	2,1	10.88	-1.40	*no*	391	Rmet_0948	*cyoA*	Op0267f_4	MET-EN-OxPhos
736	TSS_767856-3	491	73	0,1	-16	1	-36	TCGTGTACCT	-14	TCTATATTTG	-141	1,1	6.18	10.90	s	59	Rmet_4183	*0*	Op1181r_2	NA
737	TSS_2997288-2	491	78	0,1	-16	1	-44	GCCGGAACAA	-14	TGTACAATGC	-138	4,1	10.31	-8.60	*no*	44	Rmet_2755	*dcd*	Op0776r_2	MET-NUC-Pyr
738	TSS_2448958+3	491	173	0,1	-16	1	-49	TTGCAATCCC	-14	CCTAAGATTC	-139	1,1	9.08	-13.60	*no*	23	Rmet_5690	*0*	Op1698f_1	NA
739	TSS_289181+2	489	85	0,1	-16	1	-49	TCGCAGCGAT	-14	GCTACACTGA	-142	1,1	8.37	-16.10	*no*	30	Rmet_0273	*0*	Op0079f_1	NA
740	TSS_2613866+2	489	25	0,4	-7	0	-41	TGCAGGTAGG	-3	CATAAGCAGC	-149	1,1	-0.42	-14.60	*no*	102	Rmet_2404	*tnmB*	Op2033f_1	NA
741	TSS_865051-2	488	41	0,1	-15	1	-44	TGGACTGACC	-13	ATTACAATCG	-138	1,1	8.59	-4.10	*no*	54	Rmet_0788	*upp*	Op0224r_1	MET-NUC-Pyr
742	TSS_1342323-2	488	45	0,1	-16	1	-35	TTGCATCGCC	-14	ACTATATTTC	-141	1,1	8.40	11.60	s	40	Rmet_1222	*0*	Op0354r_1	MET-CAH-Prop
743	TSS_134842-2	488	116	0,1	-21	0	-35	TTGCATCGGG	-14	GGTTAAATCC	-143	1,1	4.67	9.60	s	0	Rmet_0128	*zur*	Op0032r_1	NA
744	TSS_945936+3	488	44	0,2	-10	1	-35	TGGACAGACT	-14	GATATGAATA	-144	1,1	3.53	11.10	s	961	Rmet_6661	*0*	Op1240f_1	NA
745	TSS_3169649+2	487	47	0,1	-18	1	-41	TCGCCGACGC	-16	GATAAAGTGT	-137	1,1	7.22	2.90	*(w)-sba*	71	Rmet_2915	*folK*	Op0825f_2	MET-COF-Folate
746	TSS_600128+3	487	30	0,1	-17	1	-38	GTACGCGTAT	-15	TGTACAGTAC	-143	4,1	6.87	3.90	m	79	Rmet_4030	*0*	Op1136f_1	NA
747	TSS_2514870+3	487	150	0,1	-16	1	-48	TTGCTCCCGT	-14	GCTATCCTTG	-138	1,1	8.31	-12.60	*no*	45	Rmet_5754	*fumCa*	Op1724f_1	NA
748	TSS_3477069+2	486	23	0,1	-17	1	-47	TTGAAACAGC	-15	TCTATAATCG	-139	1,1	11.59	-5.60	*no*	93	Rmet_3202	*0*	Op0901f_2	NA
749	TSS_1439419-3	485	169	0,1	-16	1	-35	TTGCATCCTA	-14	TCTATCATTC	-144	1,1	8.84	11.60	s	0	Rmet_4794	*slyA*	Op1395r_1	NA
750	TSS_1888402+2	485	48	0,1	-16	1	-35	GCGGCAAGTT	-14	ATTACACTTG	-140	5,1	10.25	8.10	s	29	Rmet_1741	*uspA6*	Op0495f_1	NA
751	TSS_2880393+2	485	58	0,1	-16	1	-34	TGCACAATTT	-14	CGTAAAGTTT	-144	1,1	6.40	8.60	s	125	Rmet_2643	*cfa*	Op0745f_3	NA
752	TSS_2369798-3	485	103	0,1	-14	1	-37	TTCTCAAGTT	-12	GTTAGCTTTT	-143	1,1	5.34	6.90	s	106	Rmet_5612	*tar*	Op1675r_3	DIV-MOT-Chemotax
753	TSS_3004496-2	484	31	0,1	-13	1	-47	TTGCGGCCGC	-11	ACTAGAATAT	-140	1,1	8.59	-12.60	*no*	53	Rmet_2762	*0*	Op0776r_1	NA
754	TSS_100701-2	482	66	0,1	-16	1	-46	GCCACACCGG	-14	GCTACGATTG	-138	4,1	8.61	-11.60	*no*	76	Rmet_0092	*0*	Op0026r_1	NA
755	TSS_2450572+2	481	62	0,1	-17	1	-36	TTTGTTTTCG	-15	ACTAGAATGC	-142	2,1	12.73	10.30	s	2750	Rmet_2238	*glpK*	Op0641f_1	MET-LIP-GlycLip
756	TSS_1576539+2	481	94	0,1	-16	1	-45	TGGCAGCAAC	-14	GTCACAATAG	-143	1,1	7.47	-8.10	*no*	155	Rmet_1454	*0*	Op0405f_1	NA
757	TSS_2174604+2	481	73	0,4	-7	0	-44	TGCCCGCTGC	-21	GCGACACTGC	-138	1,1	5.59	-0.60	*no*	102	Rmet_2007	*tnmB*	Op0587f_1	NA
758	TSS_2799455+2	479	153	0,1	-16	1	-41	TTACCCAAGG	-14	TTTATAATTC	-141	4,1	8.56	2.90	*(w)-sba*	1309	Rmet_2576	*rsgA*	Op0715f_1	NA
759	TSS_1012336+3	477	35	0,1	-16	1	-38	TCGCAGCCAA	-14	CGTACAATGC	-134	1,1	8.71	6.90	s	25	Rmet_4416	*0*	Op1258f_2	NA
760	TSS_1917598+3	477	196	0,1	-16	1	-35	TAGACCGCGG	-14	CTCATCATAC	-147	1,1	2.44	11.10	s	159	Rmet_5223	*chrF3*	Op1540f_2	NA
761	TSS_174805-3	476	6	0,1	-16	1	-35	TGGCAAAGCC	-14	TCTACCCTTT	-139	1,1	6.87	9.10	s	3723	Rmet_3634	*0*	Op1011r_1	MET-AA-ArgPro
762	TSS_2126744-3	475	72	0,1	-15	1	-40	TTCAAGTTCC	-16	CGTAGTATCC	-143	1,1	7.02	4.90	*(m)-sba*	19	Rmet_6724	*0*	Op2090r_1	NA
763	TSS_2148342+2	474	83	0,1	-16	1	-41	GCTGCCTCAT	-14	GCTACAATAT	-138	5,1	10.23	-2.60	*no*	119	Rmet_1972	*0*	Op0575f_2	NA
764	TSS_3448622+2	473	53	0,1	-17	1	-42	TCGTCCCGAC	-15	CGTAAAATCC	-136	1,1	7.53	0.90	*(w)-sba*	749	Rmet_3177	*cbbZ2*	Op0893f_1	MET-CAH-DiCarb
765	TSS_2662575-2	473	27	0,1	-16	1	-42	TTCAGGGCAT	-14	GCTAATATTC	-139	1,1	7.88	-1.10	*no*	90	Rmet_2450	*0*	Op0684r_1	NA
766	TSS_1522284-3	473	83	0,1	-16	1	-38	TGGCTGAGCC	-14	GCTAAGGTAG	-141	1,1	5.11	4.90	m	70	Rmet_4862	*0*	Op1419r_4	NA
767	TSS_717258+3	473	48	0,1	-16	1	-46	TTGTCGGGGG	-14	GATAAGATGC	-136	1,1	8.68	-7.60	*no*	92	Rmet_4143	*0*	Op1168f_1	NA
768	TSS_1702959-2	471	61	0,1	-15	1	-31	TTCCGGATCG	-11	TATAATGTGC	-144	1,1	4.63	3.10	w	32	Rmet_1581	*0*	Op0436r_1	NA
769	TSS_3352511+2	470	111	0,1	-15	1	-38	GTAGAGAAGT	-13	AGTACAATGG	-137	4,1	10.80	2.90	w	117	Rmet_3094	*ffh*	Op0871f_1	GIP-PTL-Exp
770	TSS_2628986-2	469	54	0,1	-16	1	-35	TTCCGTTCCG	-14	CTTACAATCA	-138	2,1	12.39	10.10	s	223	Rmet_2419	*0*	Op0680r_4	NA
771	TSS_1375651+3	469	59	0,1	-17	1	-37	TTGACAATTG	-15	GGGACGATTC	-142	1,1	8.27	12.40	s	187	Rmet_4732	*0*	Op1368f_2	NA
772	TSS_2909981-2	469	89	0,1	-4	0	-45	TTGCAACATA	-2	GCTACCATGA	-142	1,1	9.40	-17.60	*no*	138	Rmet_2675	*tolB*	Op0756r_3	NA
773	TSS_3368884+2	469	82	0,1	-16	1	-49	TTGCAGATTG	-14	GCTATCGTTG	-140	1,1	7.93	-14.60	*no*	99	Rmet_3109	*pilC*	Op0875f_2	GIP-PTL-T2S
774	TSS_436978-2	469	67	0,4	-23	0	-33	TTGCAAAAGA	-4	GTTGGACTTG	-145	1,1	3.45	1.20	*(w)-ba*	451	Rmet_0408	*0*	Op0112r_2	NA
775	TSS_3830498-2	468	80	0,1	-15	1	-34	TTGCGCTTGC	-13	GTCACCCTCG	-141	1,1	6.44	7.60	s	23	Rmet_3531	*0*	Op0976r_2	NA
776	TSS_2839151-2	468	47	0,1	-15	1	-35	GCAGCCAGAT	-13	GCTATACTGA	-137	5,1	11.40	7.20	s	120	Rmet_2608	*0*	Op0730r_2	NA
777	TSS_65906-5	468	90	0,1	-15	1	-35	TTGCACAGTC	-13	GGCACAATTA	-141	1,1	9.34	11.20	s	42	Rmet_6215	*0*	Op1899r_4	NA
778	TSS_448743-2	466	20	0,1	-15	1	-43	TCGCGAACGG	-13	CGTAGAATGG	-137	1,1	8.60	-3.10	*no*	18	Rmet_0423	*ruvB*	Op0116r_3	GIP-REP-Recomb
779	TSS_1336534-2	466	115	0,1	-15	1	-34	TTGCATTGCG	-13	CCTATAATCA	-139	1,1	10.09	11.60	s	9	Rmet_R0019	*0*	Op0352r_1	NA
780	TSS_1161428+3	466	137	0,1	-17	1	-48	TTGAGGAGAC	-15	AGTACACTCG	-136	1,1	9.26	-10.60	*no*	55	Rmet_4550	*0*	Op1306f_2	NA
781	TSS_1952221+3	466	229	0,1	-14	1	-47	GCAAAGATCA	-12	GATAACCTTT	-143	4,1	6.57	-18.60	*no*	88	Rmet_5252	*fliC2*	Op1550f_2	EIP-SIG-2Comp
782	TSS_1985627-2	465	71	0,1	-15	1	-43	TTGCGGTGAC	-13	GGTATTATAT	-137	1,1	9.40	-2.60	*no*	80	Rmet_1829	*yceG*	Op0530r_1	MET-NUC-Pyr
783	TSS_2635967-2	464	66	0,1	-16	1	-41	GCGACACCCG	-14	GGTAAAATAC	-141	5,1	9.58	0.90	*(w)-sba*	49	Rmet_2425	*rpoE*	Op0680r_3	GIP-TK-RNAP
784	TSS_22259+5	464	84	0,4	-7	0	-35	TGGACAAACC	-14	GCTATGGTGT	-141	1,1	7.49	12.10	s	102	Rmet_6334	*tnmB*	Op1926f_1	NA

785	TSS_3161714-2	463	94	0,1	-16	1	-37	TGACGGATCA	-14	TGTATGATGT	-143	4,1	5.97	7.40	s	20	Rmet_2907	*dedA*	Op0822r_1	NA
786	TSS_1137002+2	463	160	0,1	-16	1	-36	TTCCGGTAGC	-14	GATATAATGT	-140	1,1	10.25	11.90	s	58	Rmet_1045	*edd*	Op0301f_1	MET-CAH-PP_KDPG
787	TSS_538288-3	462	73	0,1	-21	0	-35	TTGAAATGGC	-14	AGTACCCTAC	-138	1,1	7.96	11.60	s	1760	Rmet_3972	*0*	Op1113r_2	NA
788	TSS_3513988+2	461	74	0,1	-15	1	-45	TTGCGATCAT	-13	GATACGATAC	-141	1,1	9.22	-6.60	*no*	17381	Rmet_3261	*0*	Op0919f_1	NA
789	TSS_427191+2	461	83	0,1	-16	1	-38	TACTGTTTCG	-14	GATACAGTCC	-143	2,1	9.45	4.40	m	25	Rmet_0399	*gltI*	Op0109f_4	EIP-TRA-ABC
790	TSS_2905079-2	461	61	0,1	-16	1	-35	TTGACAGTCG	-14	CCTATAATCG	-136	1,1	11.04	15.60	s	11	Rmet_R0045	*0*	Op0756r_4	NA
791	TSS_2929725+2	460	106	0,1	-17	1	-41	TTTTTTTGCG	-15	TGTATCGTCA	-142	2,1	10.43	0.90	*(w)-sba*	16	Rmet_2698	*ubiD*	Op0761f_1	MET-COF-CoQ
792	TSS_2956901+2	460	72	0,1	-16	1	-28	GCGGCAGTAG	-5	GTGAAACTCT	-144	5,1	8.47	-0.30	*no*	29	Rmet_2722	*0*	Op0769f_2	NA
793	TSS_1967772+3	459	33	0,1	-16	1	-36	TTGCCTGCAC	-14	ACTACGATAG	-138	1,1	7.76	12.40	s	2078	Rmet_5271	*0*	Op1554f_1	MET-AA-ValLeuIle
794	TSS_1578769-2	457	206	0,1	-14	1	-45	GCACCGGCGG	-12	GCTAAAATCG	-139	4,1	10.05	-12.60	*no*	114	Rmet_1456	*smpB*	Op0406r_1	GIP-PTL-Fold
795	TSS_2147911+2	456	59	0,1	-16	1	-42	TTGCTGACAG	-14	GTTATGATTT	-136	1,1	9.24	0.40	*(w)-sba*	25	Rmet_1971	*0*	Op0575f_2	NA
796	TSS_3516968-2	456	94	0,1	-18	1	-45	TGGCGTCGTG	-16	GCGATAATAT	-141	1,1	6.75	-5.10	*no*	254	Rmet_3243	*hisA*	Op0918r_3	MET-AA-His
797	TSS_438012+3	456	113	0,1	-16	1	-42	TCGTCTCTAT	-14	GATAATATTT	-146	1,1	6.33	-1.10	*no*	166	Rmet_3894	*0*	Op1086f_2	NA
798	TSS_2040924+2	455	51	0,1	-16	1	-42	TTGCCGATAG	-14	GCTAAGGTAA	-139	1,1	6.60	-0.60	*no*	141	Rmet_1879	*pgi*	Op0541f_6	MET-CAH-FbP
799	TSS_319474+3	455	80	0,1	-16	1	-34	TGGCGTGTGG	-14	GATAGAATTG	-137	1,1	8.65	8.10	s	998	Rmet_3784	*bugT*	Op1050f_2	NA
800	TSS_3065585+2	454	76	0,1	-16	1	-45	GGCCGAACGT	-14	AATAAACTCG	-143	4,1	8.57	-11.60	*no*	46	Rmet_2818	*cysB*	Op0793f_1	NA
801	TSS_158425-2	454	85	0,1	-16	1	-42	GCCCGCCCGG	-14	ATTACAATTC	-141	4,1	8.19	-5.60	*no*	0	Rmet_0153	*dapF*	Op0044r_2	MET-AA-Lys_Syn
802	TSS_70296+2	453	256	0,1	-16	1	-35	TGCCGTGCAC	-14	GATACCATGA	-145	1,1	5.65	7.60	s	121	Rmet_0062	*0*	Op0019f_2	NA
803	TSS_408038+2	453	94	0,1	-16	1	-47	TCGCCAGGGC	-14	CCGATAATCG	-141	1,1	7.41	-9.10	*no*	1683	Rmet_0384	*0*	Op0105f_2	NA
804	TSS_1227516-2	453	41	0,1	-16	1	-35	TTGCGACGCC	-14	TATATAATCC	-135	1,1	10.70	13.60	s	227	Rmet_1121	*acrD*	Op0320r_1	EIP-TRA-Ion
805	TSS_2306613-3	453	92	0,1	-15	1	-35	TTGCGGGCCA	-13	GCTATGCTGC	-137	1,1	8.53	11.20	s	39	Rmet_5558	*0*	Op1649r_1	NA
806	TSS_29830-4	453	118	0,1	-15	1	-40	TGGCGAGGGT	-13	ACTAGAATGG	-144	1,1	9.02	2.90	*(w)-sba*	0	Rmet_6034	*0*	Op1838r_1	NA
807	TSS_2874184+2	452	78	0,1	-16	1	-40	TTCCCGCAAA	-14	GTTATCCTTG	-141	1,1	7.37	2.90	*(w)-sba*	24	Rmet_2638	*0*	Op0743f_1	NA
808	TSS_3767804+2	452	49	0,1	-16	1	-34	TTGCAGGCAC	-14	CGTATGATTG	-138	1,1	9.25	9.60	s	28	Rmet_3484	*0*	Op0969f_2	MET-CAH-Pyr
809	TSS_2268109-2	451	91	0,1	-14	1	-44	TGGCGTAAAC	-12	GCTACACTAC	-139	1,1	8.19	-8.10	*no*	27	Rmet_2088	*cbbI2*	Op0604r_1	MET-CAH-PP_KDPG
810	TSS_756434-3	451	1	0,1	-16	1	-46	TTCCCACGCG	-14	TATATAGTAA	-142	1,1	6.36	-7.10	*no*	80	Rmet_4169	*ispH*	Op1173r_1	NA
811	TSS_668536-3	451	28	0,1	-15	1	-33	TCAGCAAGCG	-13	CGTAGAATCG	-140	5,1	10.73	7.60	s	22	Rmet_6647	*0*	Op2066r_1	NA
812	TSS_210302+3	449	66	0,1	-16	1	-40	TCGGCATGAC	-14	GTTACCATAG	-141	5,1	9.25	3.90	*(m)-sba*	67	Rmet_3673	*0*	Op1022f_1	NA
813	TSS_1001281-3	449	59	0,1	-17	1	-36	TTGAAAACAA	-15	GGTATCTTCC	-141	1,1	8.51	12.80	s	578	Rmet_4403	*shc*	Op1253r_1	NA
814	TSS_563822-2	449	38	0,1	-16	1	-35	TTGCATTCTA	-14	GGTCCAATAC	-147	1,1	5.52	9.60	s	3257	Rmet_0524	*0*	Op0142r_1	NA
815	TSS_241467+2	448	201	0,3	-24	0	-48	TTGCGTCTGG	-15	CAAATAGTTA	-146	1,1	4.19	-11.60	*no*	83	Rmet_0228	*dctA*	Op0067f_1	NA
816	TSS_2981229+2	448	85	0,1	-16	1	-41	TCGACCAATG	-14	GGCATAATCG	-138	1,1	8.65	2.90	*(w)-sba*	18	Rmet_2746	*0*	Op0773f_1	NA
817	TSS_2221236+3	447	56	0,1	-16	1	-36	TCGATAGTGC	-14	GGTAGAATGG	-138	1,1	9.50	12.90	s	122	Rmet_5477	*0*	Op1626f_2	NA
818	TSS_568394-2	445	80	0,1	-16	1	-35	GGCCGAATCC	-14	GGCATAATGC	-139	4,1	9.61	6.60	s	254	Rmet_0532	*adk*	Op0148r_1	MET-NUC-Pur
819	TSS_2292662+2	445	54	0,1	-14	1	-49	TTGCCCAGGA	-12	CGTCAAATGT	-145	1,1	5.62	-16.60	*no*	139	Rmet_2111	*0*	Op0609f_1	NA
820	TSS_2500718+2	445	92	0,1	-16	1	-36	TCGAGGTATC	-14	GTTATCATCA	-141	1,1	7.92	11.90	s	94	Rmet_2277	*tonB*	Op0651f_2	NA
821	TSS_630675-2	444	78	0,1	-16	1	-45	TTGCACTCAC	-14	CGTACGTTAA	-145	1,1	5.90	-7.60	*no*	329	Rmet_0587	*0*	Op0166r_3	NA
822	TSS_2838453-2	440	93	0,1	-15	1	-37	TCCCAAGTGG	-13	ACTACAATGA	-143	1,1	7.36	7.40	s	99	Rmet_2607	*dnaG*	Op0730r_2	GIP-REP-Complex
823	TSS_2581988+2	439	207	0,1	-16	1	-34	TTGATATTTT	-14	GCTAAATTAG	-143	1,1	8.81	10.60	s	18	Rmet_2370	*ygaV*	Op0669f_1	NA
824	TSS_1066682-2	439	84	0,1	-12	1	-46	GTAACAACAT	-10	TCTATCATGG	-142	4,1	10.21	-13.10	*no*	88	Rmet_0980	*0*	Op0276r_2	NA
825	TSS_3541815-2	438	110	0,1	-16	1	-40	CCGGCAAGCC	-14	GCTAATATGG	-138	5,1	11.25	0.90	*(w)-sba*	95	Rmet_3266	*aroB*	Op0920r_2	MET-AA-PheTyrTrp
826	TSS_3593903-2	438	64	0,1	-15	1	-49	TTGACACGGA	-13	GGTATGGTTA	-140	1,1	8.29	-12.60	*no*	2180	Rmet_3327	*rpsL*	Op0928r_2	GIP-TL-Ribo
827	TSS_128318-5	438	82	0,1	-15	1	-48	GCCCGGGCAT	-13	GGTAGAATCG	-138	4,1	10.20	-18.60	*no*	39	Rmet_6272	*0*	Op1913r_2	NA
828	TSS_995983-2	437	40	0,1	-7	0	-43	GCCCACGCGT	-5	GGGAGGATAT	-144	4,1	5.91	-18.60	*no*	118	Rmet_0909	*0*	Op0262r_2	NA
829	TSS_2282724-2	436	29	0,1	-15	1	-34	TTGCGTTCGT	-13	GCTAAATTCA	-141	1,1	8.37	9.60	s	137	Rmet_2101	*hfq*	Op0608r_2	NA
830	TSS_110705+5	435	57	0,1	-17	1	-37	TACATGTCGA	-15	CGTAGACTTC	-146	1,1	2.97	8.40	s	64	Rmet_6258	*rhsC*	Op1906f_1	NA
831	TSS_3009998-2	434	104	0,1	-15	1	-41	TCGGCACGCG	-13	ACTACAATAG	-139	5,1	11.04	1.90	*(w)-sba*	170	Rmet_2767	*mrp*	Op0776r_1	DIV-Division
832	TSS_163934+3	434	11	0,1	-17	1	-43	CCAGCCCGCG	-15	GGCATCATCG	-140	5,1	8.94	-6.60	*no*	40	Rmet_3627	*0*	Op1010f_1	NA
833	TSS_66019+4	433	26	0,1	-17	1	-35	TTGAACCTGT	-15	GTGACTCTAA	-144	1,1	5.07	8.60	s	31	Rmet_5990	*merR*	Op1825f_1	NA
834	TSS_2340171+2	433	33	0,1	-16	1	-38	TTGTGCATCG	-14	CATAAAATAC	-140	1,1	8.23	8.40	s	23	Rmet_2147	*fabI*	Op0619f_1	MET-LIP-FASyn
835	TSS_2493709+2	433	57	0,1	-16	1	-48	GCACAAGTGC	-14	GCTATAATGC	-136	4,1	13.23	-15.60	*no*	102	Rmet_2271	*0*	Op0649f_1	NA
836	TSS_3072301-2	433	45	0,1	-15	1	-48	TTCAGAAGTA	-13	GGCAAGCTGA	-144	1,1	5.46	-15.10	*no*	1411	Rmet_2822	*0*	Op0794r_1	NA
837	TSS_3672040-2	432	23	0,1	-16	1	-39	TTCCCGTATA	-14	TGTACACTGC	-137	1,1	8.68	4.90	m	92	Rmet_3395	*0*	Op0942r_1	NA
838	TSS_126233+5	432	105	0,1	-16	1	-49	TCAGCCGCAT	-28	GGTAGAGTTT	-143	5,1	8.79	-4.20	*no*	4380	Rmet_6274	*0*	Op1914f_1	NA
839	TSS_3676895+2	431	114	0,1	-16	1	-36	TTCCCGCTGG	-14	GATACAATCT	-137	1,1	9.29	11.90	s	53	Rmet_3399	*0*	Op0943f_1	NA
840	TSS_3186986+2	430	54	0,1	-16	1	-34	TTGCACGCAT	-14	AGTACCATGT	-137	1,1	8.59	8.60	s	23	Rmet_2930	*lrgA*	Op0827f_1	EIP-TRA-Pores
841	TSS_1503983-2	429	23	0,1	-16	1	-45	TTTTTTTGCG	-14	CTTATCATCG	-138	2,1	12.49	-7.10	*no*	32	Rmet_1394	*0*	Op0390r_1	NA
842	TSS_929936-2	429	43	0,1	-15	1	-37	GCGGCCGGGC	-13	GCTACAATGC	-136	5,1	12.94	5.90	m	16	Rmet_R0008	*0*	Op0242r_1	NA
843	TSS_929692+2	428	85	0,1	-17	1	-36	TTGTCAGTGC	-15	CTAATAATGT	-143	1,1	7.34	13.80	s	467	Rmet_0849	*0*	Op0243f_1	NA
844	TSS_2051890-2	428	89	0,1	-15	1	-41	TGGAAGCCTA	-13	GGTAAATTTC	-141	1,1	7.73	-0.10	*no*	180	Rmet_1885	*clpP*	Op0544r_2	GIP-PTL-Fold
845	TSS_2359627-2	427	40	0,1	-15	1	-47	TCGAAGACAG	-26	GACAAACTTC	-143	1,1	6.36	-1.70	*no*	2205	Rmet_6509	*0*	Op2029r_1	NA

846	TSS_3615339-2	427	73	0,1	-14	1	-37	TGGAAACGGG	-12	TGTATAATGG	-139	1,1	10.59	9.90	s	#NV	#NV	#NV	#NV	#NV
847	TSS_627618+2	425	29	0,1	-16	1	-43	TTGTTAGCTG	-14	ACCAGAATTC	-145	1,1	6.53	-1.60	no	34	Rmet_0585	0	Op0165f_1	NA
848	TSS_2056261-2	425	87	0,1	-11	1	-32	GAACTTCGCG	-12	GACCAAACAG	-146	3,0	5.82	-3.40	no	924	Rmet_1887	trmC	Op0544r_1	NA
849	TSS_1996196+3	425	54	0,1	-16	1	-34	TTGACAACCC	-14	TCTATCATGG	-139	1,1	10.06	12.60	s	20	Rmet_5295	0	Op1564f_2	NA
850	TSS_705463-2	424	45	0,1	-16	1	-35	TTGACCCGCG	-14	TGAAAAATAC	-139	1,1	6.93	12.60	s	47	Rmet_0638	0	Op0180r_4	NA
851	TSS_787368-2	424	57	0,1	-15	1	-49	TGAAAAAAAG	-13	GGTAAAGTTG	-142	4,1	8.92	-16.60	no	35	Rmet_0711	ubiG	Op0202r_2	MET-COF-CoQ
852	TSS_821594-2	424	6	0,1	-17	1	-35	TGGTATCTCG	-15	TGTATATTTA	-143	1,1	6.12	9.10	s	29	Rmet_0742	splB	Op0210r_1	NA
853	TSS_780951-2	423	24	0,5	-20	0	-43	GCAGCCAAGG	-16	CGTAACGTTA	-143	4,1	5.32	-6.60	no	50	Rmet_0704	cphA2	Op0198r_1	NA
854	TSS_151209+3	422	68	0,1	-15	1	-38	TGGAAACGGG	-13	TGTATAATGG	-139	1,1	10.59	8.90	s	#NV	#NV	#NV	#NV	#NV
855	TSS_304521-2	421	85	0,1	-16	1	-42	CTGATCGGGA	-14	GCTAGAATAT	-143	1,1	4.87	-1.60	no	121	Rmet_0288	rplY	Op0084r_4	GIP-TL-Ribo
856	TSS_268700+2	420	60	0,1	-16	1	-48	GGACAAATAT	-14	TTTATAATGC	-135	4,1	11.98	-15.60	no	23	Rmet_6408	0	Op0073f_1	NA
857	TSS_478638+3	419	74	0,1	-18	1	-45	TGCGTCAGGA	-16	GGTAGGATGA	-143	1,1	8.07	-6.10	no	8	Rmet_6634	0	Op1098f_2	NA
858	TSS_3055489-2	419	70	0,1	-14	1	-33	GCGGATCGCG	-12	CGGATAATGG	-139	5,1	8.23	5.10	m	33	Rmet_2807	pepA	Op0790r_1	NA
859	TSS_2890866-2	418	70	0,1	-16	1	-40	ATTCGTTTGG	-14	ACTAAGCTCG	-146	2,1	9.32	-2.10	no	55	Rmet_2654	0	Op0752r_2	NA
860	TSS_2241490-2	417	36	0,1	-15	1	-49	TTGATGATTC	-13	GTTAGAATGC	-143	1,1	10.62	-13.60	no	22	Rmet_2068	0	Op0600r_1	NA
861	TSS_2701465+2	417	48	0,1	-17	1	-36	TTGACATCAA	-15	CCTACACTCG	-141	1,1	9.57	13.80	s	275	Rmet_2489	mdh	Op0689f_1	MET-CAH-TCA
862	TSS_2473724-3	417	79	0,1	-14	1	-44	TTGCAGCAAC	-12	GTTATCCTAG	-141	1,1	8.17	-6.60	no	124	Rmet_5714	bvgA	Op1709r_2	EIP-SIG-2Comp
863	TSS_2378011-2	415	81	0,1	-12	1	-40	TTCTTATAGC	-10	TGTAAAATTG	-143	1,1	8.69	-0.10	no	12	Rmet_R0033	0	Op0624r_1	NA
864	TSS_3026657+2	415	27	0,1	-17	1	-36	TCTGCAAGAT	-15	GCTACAATAC	-136	5,1	12.23	10.80	s	71	Rmet_2779	cobU	Op0783f_1	MET-COF-Porph
865	TSS_371512+2	413	16	0,1	-17	1	-43	TTCACGGTAG	-15	GCTAAGCTGC	-142	1,1	7.42	-2.10	no	4020	Rmet_0355	otsB	Op0101f_1	MET-CAH-Starch
866	TSS_2666446+2	413	66	0,5	-3	0	-37	TTGACTCACC	-13	TAAAAGCTTA	-146	1,1	3.71	8.40	s	243	Rmet_2455	rpsU1	Op0685f_3	GIP-TL-Ribo
867	TSS_2363154-2	413	66	0,1	-16	1	-44	GCGGCAGGCT	-14	TTTACAATGG	-138	5,1	12.56	-6.10	no	81	Rmet_2162	0	Op0622r_1	NA
868	TSS_1559182+2	412	49	0,1	-16	1	-40	TGGATGCGGA	-14	AGTAGAATGT	-140	1,1	8.09	3.90	(m)-sba	50	Rmet_1438	frr	Op0403f_2	NA
869	TSS_1297772+3	412	111	0,1	-16	1	-47	TTGTGAGATC	-17	TGTATTATGA	-144	1,1	8.34	-5.60	no	8182	Rmet_4673	0	Op1352f_1	NA
870	TSS_443462-2	412	101	0,2	-13	1	-25	TGGACCGAAA	0	GAAACCGTTT	-146	1,1	2.59	-3.30	no	740	Rmet_0416	0	Op0114r_1	MET-AA-PheTyrTrp
871	TSS_2112156+3	412	35	0,1	-16	1	-48	TTGCCGTCAT	-14	GGTAGAATCC	-137	1,1	11.21	-10.60	no	29	Rmet_5387	0	Op1604f_1	NA
872	TSS_3867716-2	411	81	0,1	-15	1	-37	GCGGCCCGAC	-13	GGTAAGATCG	-138	5,1	11.02	4.90	m	165	Rmet_3565	0	Op0988r_1	NA
873	TSS_3038511+2	410	49	0,1	-10	1	-30	TTGCGCGGCC	-8	GCCCGGATGT	-143	1,1	1.94	3.20	(w)-nu	538	Rmet_2792	0	Op0785f_1	NA
874	TSS_475759+3	410	68	0,1	-15	1	-38	TGGAAACGGG	-13	TGTATAATGG	-139	1,1	10.59	8.90	s	#NV	#NV	#NV	#NV	#NV
875	TSS_1187915-3	410	87	0,1	-16	1	-40	TCCACATTTT	-14	GGTACAATGC	-140	1,1	9.72	4.40	(m)-sba	23	Rmet_4573	0	Op1315r_1	NA
876	TSS_3380922-2	410	117	0,1	-10	1	-38	GCAGCGGAAC	-18	GTGAGGTTGT	-143	4,1	4.74	0.80	w	190	Rmet_3118	secA	Op0876r_2	GIP-PTL-Exp
877	TSS_812032+2	409	49	0,1	-16	1	-34	TTGAGGTATG	-14	CATAAAATGC	-142	1,1	10.05	10.60	s	67	Rmet_0734	metN	Op0207f_1	EIP-TRA-ABC
878	TSS_2037628+2	409	73	0,1	-15	1	-38	TGGTACAGGC	-13	GGTATGGTTT	-140	1,1	6.32	4.90	m	252	Rmet_1877	dadA	Op0541f_4	MET-EN-Nitrog
879	TSS_1181592+2	409	11	0,1	0	0	-41	TGGCCTTTAC	-15	TGTAATTTCA	-146	1,1	5.06	0.90	(w)-sba	28	Rmet_1089	lysC	Op0313f_3	MET-AA-GlySerThr
880	TSS_770554+3	409	270	0,1	-17	1	-36	TTGCTTATCT	-15	GATAGTATTC	-144	1,1	8.09	10.80	s	232	Rmet_4187	mmtQ	Op1184f_1	NA
881	TSS_2025727+2	408	59	0,1	-18	1	-36	TTCCACTTGG	-16	GGTAAGATCA	-139	1,1	8.09	8.30	s	43	Rmet_1869	0	Op0539f_2	NA
882	TSS_3430106-2	408	100	0,1	-14	1	-37	TGGAAACGGG	-12	TGTATAATGG	-139	1,1	10.59	9.90	s	#NV	#NV	#NV	#NV	#NV
883	TSS_3895221-2	408	24	0,1	-16	1	-37	TCGAGCACGA	-14	GGTACGATGC	-140	1,1	8.34	8.90	s	100	Rmet_3587	acrR	Op0998r_1	NA
884	TSS_3149749-2	407	51	0,1	-10	1	-34	TTGAATAACA	-8	TGCAGACTTG	-141	1,1	7.17	6.20	(m)-ba	25	Rmet_2893	clpS	Op0812r_1	GIP-PTL-Fold
885	TSS_1975511+3	406	12	0,1	-16	1	-37	TCAGACATGA	-14	GCTATGCTCC	-142	4,1	6.77	6.40	s	107	Rmet_5276	0	Op1554f_2	NA
886	TSS_1269598+2	404	67	0,1	-16	1	-36	TTGCACGCGA	-14	GTTATACTTG	-139	1,1	10.23	12.40	s	5	Rmet_R0015	0	Op0333f_1	NA
887	TSS_1975305+2	404	27	0,1	-15	1	-34	TCGCGCCGGG	-13	TATACAATCC	-138	1,1	8.27	9.10	s	25	Rmet_1820	purT	Op0527f_2	MET-NUC-Pur
888	TSS_3526826-2	404	39	0,4	0	0	-46	GAATGTGGTG	-26	TTCGCGCCCC	-146	3,0	5.09	-12.20	no	64	Rmet_3255	ttg2D	Op0918r_3	EIP-TRA-ABC
889	TSS_2452516-2	403	79	0,1	-15	1	-33	TCGATCCGGA	-13	GCTATCATGG	-138	1,1	8.09	8.10	s	126	Rmet_2236	purU	Op0640r_2	MET-CAH-DiCarb
890	TSS_3157265+2	403	116	0,1	-16	1	-41	TTCCCGCTAT	-14	GATATAATAC	-138	1,1	9.85	2.90	(w)-sba	25	Rmet_2903	0	Op0819f_1	NA
891	TSS_2729655+2	402	56	0,1	-16	1	-33	TCGAACACTC	-3	GCGAGAGTGT	-145	1,1	4.72	-0.30	no	336	Rmet_2515	lldP	Op0695f_3	EIP-TRA-Ion
892	TSS_1152529-3	401	46	0,1	-15	1	-42	TTCCAGATCG	-13	GGTATCATCA	-142	1,1	8.30	-2.10	no	684	Rmet_4545	0	Op1303r_2	NA
893	TSS_1223741-2	401	96	0,2	-25	0	-35	TTGACAGCAT	-13	CGCACCCTCC	-140	1,1	6.29	12.20	s	290	Rmet_1119	0	Op0320r_1	GIP-TK-RNAP
894	TSS_2220168+3	401	21	0,1	-18	1	-36	TTTCTTTCGG	-16	GATACACTGT	-138	2,1	12.43	8.30	s	296	Rmet_5475	0	Op1626f_1	NA
895	TSS_17860+2	400	42	0,1	-16	1	-37	TTCCGCCACG	-14	GCTAGACTTC	-137	1,1	7.76	7.90	s	23	Rmet_0014	0	Op0003f_1	NA
896	TSS_2872562-2	399	3	0,1	-14	1	-34	TTGAAAATAG	-12	GTTATATTGC	-139	1,1	10.42	13.20	s	78	Rmet_2635	fadD	Op0740r_1	MET-LIP-FASyn
897	TSS_2377428-2	398	53	0,2	-2	0	-24	TTGCCGCCTG	0	AGTAGCGTGG	-142	1,1	5.67	-1.80	no	1433	Rmet_2171	0	Op0624r_1	NA
898	TSS_289100-2	398	65	0,1	-13	1	-43	GAAATCGCTG	-23	TCGGGGCTGC	-145	3,0	6.85	-6.20	no	220	Rmet_0272	rpoH	Op0078r_1	GIP-TK-RNAP
899	TSS_130858-5	398	45	0,1	-15	1	-35	TTGCCAGTGA	-13	TTTATGCTTG	-140	1,1	8.72	13.20	s	146	Rmet_6399	0	Op1913r_1	NA
900	TSS_1684650+3	398	90	0,1	-16	1	-45	TCCATACACC	-14	GCTAGGATTT	-143	1,1	5.88	-7.60	no	28	Rmet_5004	0	Op1468f_1	NA
901	TSS_866219+2	397	18	0,1	-16	1	-43	GCGGCCCCGG	-14	CATACAATCG	-135	5,1	10.54	-5.10	no	102	Rmet_0790	0	Op0225f_1	NA
902	TSS_179156+2	396	75	0,1	-17	1	-46	CCGGCCGTGC	-15	TGCACAATTA	-136	5,1	9.37	-11.10	no	25	Rmet_0171	0	Op0051f_1	NA
903	TSS_603952+2	395	57	0,1	-16	1	-35	TTGCCATGTC	-14	GGTAGATTTT	-139	1,1	9.42	12.60	s	101	Rmet_0564	0	Op0159f_1	NA
904	TSS_2773893-2	394	93	0,1	-15	1	-48	TATCGCCGGG	-13	GGTAACATGC	-139	2,1	11.91	-16.60	no	327	Rmet_6533	0	Op2035r_1	NA
905	TSS_1002059+2	394	73	0,1	-3	0	-24	TTGCTTTTCT	-1	TGTACGTTTC	-141	1,1	6.71	-1.80	no	29	Rmet_0917	0	Op0263f_4	NA
906	TSS_2661900-2	394	34	0,1	-15	1	-39	GCCAGCGCGC	-13	GTTAGCATGA	-138	4,1	8.69	-0.60	no	12	Rmet_2449	pncB	Op0684r_1	MET-COF-NicAm

907	TSS_455512-3	393	91	0,1	-18	1	-33	TTCCAGCCTC	-13	ACGATCATGG	-140	1,1	5.19	6.10	m	122	Rmet_3907		*0*	Op1093r_1	NA	
908	TSS_942924-2	393	52	0,1	-15	1	-33	AAATCTGTCA	-13	TGTAAGCTGT	-142	3,1	11.30	1.60	w	36	Rmet_0863		*0*	Op0248r_2	NA	
909	TSS_886408+2	392	53	0,1	-15	1	-33	TTCAGCCTGC	-13	GCTACAATAC	-137	1,1	9.40	8.10	s	26	Rmet_0811	*sirA*		Op0229f_2	GIP-PTL-Fold	
910	TSS_15087+4	392	103	0,1	-14	1	-35	TTGATCGGTT	-12	TGTAAACTGA	-140	1,1	7.25	9.70	s	1333	Rmet_6049		*0*	Op1843f_1	NA	
911	TSS_258840+2	390	26	0,1	-17	1	-37	CCTGCAGCCT	-15	ACTACAATCG	-138	5,1	11.20	7.40	s	28	Rmet_0242	*gshA*		Op0071f_3	MET-AA-GluGSH	
912	TSS_1756624+2	390	465	0,1	-17	1	-42	TCGAAAGCGA	-15	AATACCATCC	-142	1,1	6.79	0.90	*(w)-sba*	33	Rmet_1628		*0*	Op0455f_1	NA	
913	TSS_3307409-2	390	137	0,1	-15	1	-42	TTGCAGCCAA	-13	GTTATGCTGT	-137	1,1	8.49	-1.60	*no*	50	Rmet_3050	*pyrF*		Op0858r_1	MET-NUC-Pyr	
914	TSS_234895+3	390	48	0,1	-16	1	-37	TTCAGCGTAC	-14	GGCACAATCG	-140	1,1	8.04	8.90	s	33	Rmet_3696		*0*	Op1026f_3	NA	
915	TSS_87181-2	388	43	0,1	-17	1	-40	TTGCTGTGCG	-15	AGTACTATCT	-138	2,1	10.84	4.40	*(m)-sba*	337	Rmet_0079		*0*	Op0022r_2	NA	
916	TSS_3367142+2	387	73	0,1	-16	1	-45	GCAACCGAGC	-14	GGCAAACTGC	-139	4,1	8.93	-11.60	*no*	90	Rmet_3108	*pilB*		Op0875f_2	GIP-PTL-T2S	
917	TSS_3874333-2	387	48	0,1	-15	1	-38	TTCAACAATA	-13	GTAAAAATTC	-145	1,1	6.88	5.90	m	20	Rmet_3570		*0*	Op0990r_1	NA	
918	TSS_81731-5	387	17	0,1	-16	1	-34	TGCCTACTCC	-14	TGTACTATTT	-142	1,1	5.75	6.60	s	274	Rmet_6230		*0*	Op1901r_3	NA	
919	TSS_321799-2	386	99	0,1	-15	1	-33	TTCAACAACG	-13	AGTACAATTG	-138	1,1	9.14	8.10	s	118	Rmet_0305		*0*	Op0086r_1	NA	
920	TSS_960045+2	385	10	0,1	0	0	-46	AGATGCGGCG	-26	AAAGGGCTGT	-146	3,0	4.34	-10.20	*no*	53	Rmet_0877		*0*	Op0255f_1	NA	
921	TSS_829654+2	385	31	0,1	-18	1	-49	GCAAAAACCC	-16	GGTACAATGG	-133	4,1	13.30	-15.60	*no*	3495	Rmet_0755		*0*	Op0215f_1	NA	
922	TSS_149360+2	385	64	0,2	-16	1	-35	TTGACGCAAG	-14	CATACCGTAC	-141	1,1	5.60	11.60	s	28	Rmet_0145		*0*	Op0039f_1	NA	
923	TSS_431332-2	384	83	0,1	-15	1	-33	TGGCAGCGCC	-13	AGGATAATGC	-137	1,1	7.40	7.10	s	45	Rmet_0403	*yafJ*		Op0110r_1	NA	
924	TSS_1675145+3	384	43	0,1	-16	1	-30	TTGCGTTGCG	-1	GGCACAATTA	-138	2,1	13.15	-0.80	*no*	9533	Rmet_5004		*0*	Op1468f_1	NA	
925	TSS_375550+2	383	24	0,1	-17	1	-38	GCTGACACCT	-15	GTTACGGTCT	-142	5,1	7.35	1.40	w	797	Rmet_0356		*0*	Op0101f_1	NA	
926	TSS_89946+2	382	55	0,1	-16	1	-42	TCGTGCTCGG	-14	TGCAGAATGG	-140	1,1	5.87	-2.10	*no*	155	Rmet_0083		*0*	Op0023f_1	NA	
927	TSS_785024+2	382	137	0,1	-25	0	-45	TTCCGATTTT	-23	CGAAGAATGC	-145	1,1	6.27	1.90	*w*	57	Rmet_0709		*0*	Op0201f_1	NA	
928	TSS_2652208+2	382	49	0,1	-17	1	-38	TCCACGATGC	-15	GCTAGAATGG	-139	1,1	8.59	8.40	s	1215	Rmet_2442	*mobA*		Op0683f_1	MET-COF-Folate	
929	TSS_1552459+3	382	40	0,1	-16	1	-34	TTCTTGCCGC	-14	GCTATCATTC	-142	1,1	7.27	8.10	s	40	Rmet_4890		*0*	Op1430f_1	NA	
930	TSS_3874375-2	381	42	0,1	-19	0	-43	TTCCCTGATA	-17	TCCACAATGA	-144	1,1	6.86	-0.10	*no*	62	Rmet_3570		*0*	Op0990r_1	NA	
931	TSS_597735-2	379	64	0,1	-16	1	-38	TTCCCAGACG	-14	GGCACAATAC	-139	1,1	7.96	7.90	s	0	Rmet_0558		*0*	Op0156r_1	NA	
932	TSS_147293+2	378	20	0,1	-16	1	-36	GCAGGCACAA	-14	ACTATAATGT	-140	4,1	9.85	7.40	s	222	Rmet_0143	*metX*		Op0039f_1	MET-EN-Sulf	
933	TSS_1557490+2	378	43	0,4	-12	1	-39	TCGAACTGAA	-10	TCGAAGAACG	-148	1,1	-0.12	-2.10	no	857	Rmet_1437	*pyrH*		Op0403f_1	NA	
934	TSS_2483509-2	378	16	0,1	-16	1	-36	TTGCTGGCTG	-14	TATACAGTGC	-141	1,1	7.24	11.40	s	0	Rmet_6514		*0*	Op2031r_1	NA	
935	TSS_2049277-2	376	13	0,1	-20	0	-25	GAATTCCGCG	-5	CGCCTTAAAT	-147	3,0	6.69	-10.40	*no*	22	Rmet_1883	*lon*		Op0544r_2	GIP-PTL-Fold	
936	TSS_689178+3	375	63	0,1	-13	1	-33	GAACTTGCCG	-13	GGTCCATTGT	-138	3,0	7.23	-0.40	no	111	Rmet_4119	*hmyF*		Op1160f_1	NA	
937	TSS_593804+3	375	36	0,1	-16	1	-36	CCGGCAGGCT	-14	TTCACAATAG	-137	5,1	10.01	8.90	s	221	Rmet_4025		*0*	Op1132f_1	NA	
938	TSS_3020033-2	374	16	0,1	-15	1	-25	TTGCGGCGGT	-4	GCGACTTTGC	-145	1,1	5.15	-1.40	*no*	1947	Rmet_2771		*0*	Op0778r_1	NA	
939	TSS_2937767+2	373	46	0,1	-18	1	-36	TGGCCTCTCG	-16	GCTACAATCC	-136	1,1	9.51	10.30	s	10	Rmet_R0046		*0*	Op0765f_1	NA	
940	TSS_39893+5	373	48	0,1	-17	1	-35	CCAACAGTAC	-15	GCTAGGATTC	-141	5,1	7.53	6.60	s	28	Rmet_6190		*0*	Op1894f_5	NA	
941	TSS_808726+2	372	66	0,1	-17	1	-44	CCGGCCGCCG	-15	ATTACAATGG	-137	5,1	11.19	-6.10	*no*	2276	Rmet_0733		*0*	Op0207f_1	NA	
942	TSS_142520+2	371	270	0,1	-17	1	-35	TCTGAACGGC	-15	GCCAGACTGC	-138	5,1	8.39	6.60	s	87	Rmet_0137		*0*	Op0037f_1	NA	
943	TSS_35955+4	370	67	0,1	-26	0	-35	TTGCAAAGGG	-14	TGTCTAATAG	-142	1,1	6.24	11.60	s	533	Rmet_6023		*0*	Op1835f_1	NA	
944	TSS_3283818+2	369	44	0,1	-16	1	-43	TCGAACTAGC	-14	GGTAGATTAG	-143	1,1	7.19	-3.10	*no*	808	Rmet_3028		*0*	Op0849f_1	NA	
945	TSS_98414+5	368	35	0,1	-16	1	-44	TGGACCGACC	-14	GTAACCATCT	-145	1,1	4.87	-5.10	*no*	333	Rmet_6252	*int*		Op1904f_4	NA	
946	TSS_1171246+2	368	66	0,1	-16	1	-47	TCGCCGTCGG	-14	GCTAGAATGG	-137	1,1	9.41	-10.10	*no*	409	Rmet_1079		*0*	Op0311f_1	GIP-TL-Ass	
947	TSS_3021362+2	367	54	0,1	-16	1	-41	TCGCGCGGCC	-14	TCTACAATCC	-137	1,1	8.62	0.90	*(w)-sba*	87	Rmet_2774	*panC*		Op0779f_1	MET-OAA-Other	
948	TSS_154779-2	367	57	0,1	-15	1	-47	TCGAACTGCA	-13	CGTAGAATTG	-137	1,1	8.55	-11.10	*no*	16	Rmet_0149	*xthA1*		Op0042r_1		0
949	TSS_2121377+2	367	78	0,1	-15	1	-48	TTGAGAATAT	-13	CGCATAATCC	-138	1,1	9.84	-10.60	*no*	94	Rmet_1950	*ahpC*		Op0571f_2	GIP-PTL-Fold	
950	TSS_1346052+2	367	55	0,1	-7	0	-36	TTGACGGCGG	-5	CACATACTTC	-141	1,1	7.58	4.40	*(m)-ba*	102	Rmet_1226		*0*	Op0355f_2	EIP-TRA-ABC	
951	TSS_124007+3	366	54	0,1	-16	1	-35	TTGACCACGG	-14	GCTAGGATCG	-134	1,1	9.25	12.60	s	189	Rmet_5922		*0*	Op1798f_2	NA	
952	TSS_1689288-2	365	33	0,1	-19	0	-34	TTGCACAATA	-14	ACTATAAATA	-144	1,1	5.11	8.60	s	30	Rmet_1567		*0*	Op0432r_1	NA	
953	TSS_3611196-2	365	41	0,1	-16	1	-37	GCACACGCAC	-14	TGTACATTCT	-140	4,1	9.19	3.40	m	329	Rmet_R0056		*0*	Op0930r_4	NA	
954	TSS_3617958-2	365	47	0,1	-8	0	-35	TCGAAAGAGC	-6	CAGAGATTGA	-145	1,1	3.81	3.70	*(m)-ba*	5	Rmet_R0064		*0*	Op0930r_1	NA	
955	TSS_1722908+2	364	61	0,1	-16	1	-43	TTGATCTGCC	-14	CTTATGATGG	-136	1,1	8.99	-0.60	*no*	23	Rmet_1598		*0*	Op0443f_2	EIP-TRA-ABC	
956	TSS_2117483+2	364	54	0,1	-16	1	-46	TTGTTAAGCC	-14	GCTAAGATAC	-144	1,1	8.20	-7.60	*no*	748	Rmet_1948	*ompB*		Op0571f_1	EIP-SIG-2Comp	
957	TSS_744500+3	364	49	0,1	-6	0	-26	TCGACGGGCC	-4	GGTAACATAA	-142	1,1	7.12	2.70	*(w)-nu*	47	Rmet_4162		*0*	Op1170f_4	NA	
958	TSS_798158+2	363	52	0,1	-17	1	-40	TGCCGAAGGT	-15	GGTAGATTGC	-140	1,1	7.12	2.40	*(w)-sba*	144	Rmet_0721	*cmk*		Op0205f_1	MET-NUC-Pyr	
959	TSS_2236128+3	363	28	0,1	-16	1	-37	TTGACACGCA	-14	GATATAATCA	-138	1,1	11.17	14.40	s	35	Rmet_5491	*bug*		Op1632f_2	NA	
960	TSS_1460704+2	363	42	0,1	-16	1	-36	TGGAGGCCGC	-14	GCTATGATGA	-137	1,1	8.41	11.90	s	1084	Rmet_1353	*rluD*		Op0381f_1	GIP-TL-Ass	
961	TSS_1376733+3	362	49	0,1	-15	1	-38	TTGCGGGCAC	-13	CGTACGATGC	-137	1,1	8.69	6.40	s	3852	Rmet_4736	*kdgD*		Op1370f_1	MET-CAH-Ascorb	
962	TSS_3623687-2	361	71	0,1	-15	1	-49	TTCAGAATCA	-13	TGTAGTATGA	-143	1,1	6.97	-15.10	*no*	26	Rmet_3346	*uspA8*		Op0932r_3	NA	
963	TSS_779818-3	361	40	0,1	-16	1	-47	TCAGCACTCC	-14	GCTACGCTGT	-141	5,1	8.69	-12.60	*no*	54	Rmet_4194		*0*	Op1187r_1	NA	
964	TSS_814700+2	361	87	0,1	-16	1	-48	TTCATCCAAA	-14	GGCACAATGG	-143	1,1	7.76	-13.10	*no*	96	Rmet_0737	*etfB*		Op0207f_3	NA	
965	TSS_1350926+2	360	40	0,1	-16	1	-35	TTGACCATGG	-14	TCCATGCTTG	-140	1,1	7.02	11.60	s	170	Rmet_1231	*trkD*		Op0355f_3	EIP-TRA-Ion	
966	TSS_1836415-2	360	67	0,1	-23	0	-35	TTGCTAGGAA	-14	TCCATGCTCC	-141	1,1	6.24	10.60	s	113	Rmet_1694		*0*	Op0480r_1	NA	
967	TSS_3037561-2	359	179	0,1	-16	1	-35	TTGACGCCAA	-14	CATACACTCG	-140	1,1	8.49	12.60	s	247	Rmet_2789	*btuB*		Op0784r_1	EIP-TRA-Pores	

968	TSS_1157404+2	358	95	0,1	-17	1	-41	TGGCAGGGCT	-15	AGTAGAATGG	-139	1,1	8.80	1.90	(w)-sba	866	Rmet_1065	0	Op0307f_1	GIP-TL-Ass
969	TSS_1372778-2	358	17	0,1	-11	1	-36	TTGTAGTCCT	-9	GTCATCTTGG	-148	1,1	5.39	5.40	(m)-ba	20	Rmet_1249	0	Op0360r_1	NA
970	TSS_1690656-2	357	95	0,1	-15	1	-34	TTGCGCGCCA	-13	GGTAATATTT	-143	1,1	8.50	9.60	s	26	Rmet_1569	0	Op0432r_1	NA
971	TSS_699108+3	357	41	0,1	-25	0	-43	GCTGCAGGCT	-23	TCGATACTGT	-142	5,1	9.27	-1.20	no	52	Rmet_4128	imuA	Op1164f_1	NA
972	TSS_3312730-2	356	93	0,1	-16	1	-42	TTGAGTCTTC	-14	CTTACAATCG	-138	1,1	9.61	1.40	(w)-sba	26	Rmet_3055	0	Op0860r_2	NA
973	TSS_1053099+2	356	18	0,1	-16	1	-42	TGGTCAGTAT	-14	GACAGAATGC	-147	1,1	7.39	-0.10	no	104	Rmet_0969	0	Op0271f_1	NA
974	TSS_205236+2	356	68	0,1	-16	1	-36	TCGGCAGGAT	-14	TCAATAATCG	-140	5,1	9.60	12.90	s	31	Rmet_0197	qor	Op0057f_3	NA
975	TSS_2419112-2	355	34	0,1	-15	1	-34	TTGACGCGAG	-13	CATAGCATCA	-142	1,1	7.25	11.60	s	30	Rmet_2205	0	Op0630r_1	NA
976	TSS_2318542+3	355	59	0,1	-23	0	-44	TCGACAACAA	-21	CCAAGAGTTG	-145	1,1	3.43	2.90	w	582	Rmet_5565	0	Op1654f_1	NA
977	TSS_2200819-2	355	86	0,1	-16	1	-39	TATCGGCGCG	-14	GTTATCCTGC	-138	2,1	12.06	2.40	w	1199	Rmet_2031	infB	Op0594r_2	NA
978	TSS_2043617-2	355	97	0,1	-15	1	-39	TCGCCACTGA	-13	GCTATAATCG	-137	1,1	10.46	6.90	s	323	Rmet_1880	ybbA	Op0542r_1	EIP-TRA-ABC
979	TSS_3587414-2	354	39	0,1	-16	1	-37	GCACACGCAC	-14	TGTACATTCT	-140	4,1	9.19	3.40	m	324	Rmet_3323	rpsJ	Op0928r_3	GIP-TL-Ribo
980	TSS_1155292-2	354	55	0,1	-15	1	-45	TGGCGCACGT	-13	GATAAATTCG	-139	1,1	7.35	-9.10	no	107	Rmet_1061	0	Op0304r_1	MET-CAH-FbP
981	TSS_121660-4	354	56	0,4	-6	0	-37	TGCCAACAGC	-13	ACTATCGTGT	-143	1,1	4.69	6.40	s	103	Rmet_6181	tnmB	Op1892r_1	NA
982	TSS_2450485-2	353	13	0,4	-16	1	-34	TTGTTTTGTG	-13	GCTAAAACGA	-151	1,1	1.05	8.60	s	76	Rmet_2234	0	Op0638r_1	EIP-TRA-ABC
983	TSS_1172735-3	353	176	0,1	-14	1	-34	TGGCGCACAG	-12	GGTAAAATGA	-141	1,1	9.39	9.70	s	58	Rmet_4563	0	Op1313r_1	NA
984	TSS_1472724+2	352	44	0,1	-17	1	-36	TTGCCGCGAT	-15	TTTAAAGTCG	-138	1,1	8.25	11.80	s	64	Rmet_1363	0	Op0383f_4	NA
985	TSS_1105529-2	352	39	0,1	-16	1	-38	TTTTTCTGGC	-14	GGGATAATCC	-138	2,1	10.26	6.90	s	62	Rmet_1015	mfB	Op0292r_1	NA
986	TSS_1522805-3	352	39	0,1	-15	1	-34	TAGCGCCGCC	-13	AGCACAATGG	-139	2,1	8.96	8.10	s	24	Rmet_4863	0	Op1419r_3	NA
987	TSS_286198+2	351	85	0,1	-16	1	-48	GCAGCCCTGC	-14	ACTACAATAT	-138	5,1	9.53	-16.60	no	55	Rmet_0270	ctaB	Op0077f_1	MET-EN-OxPhos
988	TSS_2171846-2	351	54	0,4	-6	0	-32	TCGCCCCGGC	-11	TTCAGTGTGC	-144	1,1	2.85	5.10	m	103	Rmet_2001	tnmB	Op2023r_1	NA
989	TSS_3432725-2	351	12	0,1	-8	0	-35	TCGAAAGAGC	-6	CAGAGATTGA	-145	1,1	3.81	3.70	(m)-ba	5	Rmet_R0054	0	Op0886r_1	NA
990	TSS_3429870-2	351	69	0,1	-2	0	-20	TGGAAATAGC	0	CGAAAACTAT	-146	1,1	5.04	-5.90	no	#NV	#NV	#NV	#NV	#NV
991	TSS_1165281-2	350	61	0,1	-16	1	-35	TTCCTCGATC	-14	GCCATCATAT	-141	1,1	5.57	9.10	s	194	Rmet_1072	0	Op0308r_1	NA
992	TSS_316125-2	350	70	0,4	-15	1	-43	TCGACGCGC	-12	GCTTCAATGG	-143	4,1	4.95	-9.10	no	59	Rmet_0298	0	Op0086r_3	NA
993	TSS_1792749-3	349	107	0,1	-14	1	-47	GCGGCAACTC	-12	AGTAGAATGC	-136	5,1	10.78	-14.10	no	57	Rmet_5100	0	Op1501r_2	NA
994	TSS_1928113-3	349	142	0,1	-17	1	-40	TGGAAGCAAT	-15	CGTAGCATTT	-146	1,1	7.14	3.90	(m)-sba	16	Rmet_5230	iscS2	Op1543r_1	NA
995	TSS_3070937-2	347	23	0,1	-16	1	-40	TGGAGTCAGG	-14	GGTAGGATAG	-138	1,1	7.06	2.90	(w)-sba	47	Rmet_2822	0	Op0794r_1	NA
996	TSS_1724492+2	347	15	0,1	-15	1	-43	TCGAAAGCGT	-13	GTAACAATGC	-141	1,1	7.20	-3.10	no	8	Rmet_1599	0	Op0443f_3	NA
997	TSS_32111-2	347	66	0,1	-15	1	-35	TTTCGTTTGG	-13	CCTACCATGA	-141	2,1	12.15	9.70	s	33	Rmet_0027	0	Op0008r_1	NA
998	TSS_2509365-3	346	19	0,1	-14	1	-36	TTGCTGTATG	-12	CATACGATAC	-141	1,1	7.41	9.40	s	153	Rmet_6765	0	Op1719r_1	NA
999	TSS_2135228-2	346	42	0,1	-15	1	-33	TTGAAATGTC	-13	CCTATCTTTC	-142	1,1	7.94	9.60	s	93	Rmet_1959	clpB	Op0574r_2	GIP-PTL-Fold
1000	TSS_2778362-2	346	73	0,4	-6	0	-37	CTGAGTTTTA	-16	CCGATGCTGA	-151	1,1	0.56	6.80	s	103	Rmet_2553	tnmB	Op0708r_4	NA
1001	TSS_2347682+3	345	51	0,1	-17	1	-35	TTGATAAAAA	-15	GGTATATTTC	-141	1,1	10.02	12.60	s	24	Rmet_5593	0	Op1666f_1	NA
1002	TSS_225102-3	344	45	0,4	-18	1	-47	TTGTAACAGG	-13	ATCAATATTG	-147	1,1	5.71	-11.60	no	10261	Rmet_3677	stpA	Op1025r_1	NA
1003	TSS_358373-3	343	54	0,1	-15	1	-41	CCTGCAAGGG	-13	GTTATAATCC	-134	5,1	12.98	-1.60	no	140	Rmet_3816	0	Op1061r_1	NA
1004	TSS_2349690+3	343	40	0,1	-16	1	-36	TGGCGCAAGC	-14	TATATAGTCT	-140	1,1	7.15	10.90	s	24	Rmet_5595	0	Op1666f_1	NA
1005	TSS_1028763+2	343	52	0,1	-16	1	-35	TTCACAAAAT	-14	GCTAATATTG	-141	1,1	8.99	12.10	s	15	Rmet_0943	0	Op0267f_3	NA
1006	TSS_1487747+3	343	52	0,1	-16	1	-49	TTCTGTTGCG	-14	GCTAGAATGA	-139	2,1	13.28	-15.10	no	21	Rmet_4833	0	Op1408f_2	NA
1007	TSS_3645342+2	341	27	0,1	-16	1	-40	GCACGGGCAC	-14	AGTACAGTCG	-141	4,1	8.60	-2.60	no	455	Rmet_3369	paaA	Op0937f_1	NA
1008	TSS_1005102-2	341	24	0,1	-15	1	-35	TTCACACGAA	-13	GCTAAACTGC	-138	1,1	9.34	12.70	s	32	Rmet_0919	0	Op0264r_1	NA
1009	TSS_1083186+2	341	61	0,1	-19	0	-40	GCGGCTGGAC	-17	CCGACAATAC	-140	5,1	7.77	2.90	(w)-sba	22	Rmet_0994	glnE	Op0283f_1	GIP-PTL-Fold
1010	TSS_2560261-3	340	82	0,1	-15	1	-41	TTCCCCCTGC	-13	CGTACCATGG	-137	1,1	7.72	-0.10	no	30	Rmet_5801	zwf	Op1747r_1	MET-CAH-PP_KDPG
1011	TSS_2092944-2	340	62	0,1	-16	1	-43	TCGCATAGAT	-14	GGCAAAATCC	-141	1,1	8.09	-4.10	no	720	Rmet_6493	0	Op0562r_2	NA
1012	TSS_607828+3	339	53	0,1	-16	1	-49	ATTCGGCGGG	-14	GCTATCCTTG	-141	2,1	10.52	-19.10	no	5037	Rmet_4046	ggt	Op1140f_1	MET-LIP-Others
1013	TSS_409586+2	338	39	0,1	-19	0	-35	TTGATGCCAA	-14	AGAATAATCT	-143	1,1	6.65	12.60	s	135	Rmet_0384	0	Op0105f_2	NA
1014	TSS_1078005-2	338	73	0,1	-15	1	-35	CCGGCACTGC	-13	TGTAGGATTG	-139	5,1	9.65	8.70	s	57	Rmet_0991	tldD	Op0282r_2	NA
1015	TSS_2225320-3	338	52	0,4	-6	0	-33	TGCCGTGGCT	-11	GCGAGTGTGC	-143	1,1	1.53	4.20	m	103	Rmet_5481	tnmB	Op2095r_1	NA
1016	TSS_2621444+2	337	34	0,1	-16	1	-34	CGCCAACGTG	-14	TCTAGAATCG	-144	1,1	2.82	4.60	m	50	Rmet_2411	0	Op0679f_1	NA
1017	TSS_752222+2	337	28	0,1	-16	1	-36	GCAGCACGCG	-14	CGTAGAATGC	-135	5,1	11.18	8.40	s	2205	Rmet_0681	glnB	Op0191f_2	EIP-SIG-2Comp
1018	TSS_2990209+2	337	73	0,1	-15	1	-37	GCAGCAGTTC	-13	GATATAATCG	-136	5,1	11.21	6.40	s	25	Rmet_2751	argH	Op0775f_1	MET-AA-AlaAsp
1019	TSS_214879-3	335	20	0,1	-16	1	-34	TTGCAGCGGC	-14	GTTATAATTC	-140	1,1	11.86	10.60	s	38	Rmet_3677	stpA	Op1025r_1	NA
1020	TSS_2388056-2	335	24	0,1	-16	1	-48	TCGAACAGGA	-14	GGCAAACTCC	-137	1,1	7.06	-14.10	no	33	Rmet_2179	phoR	Op0628r_4	EIP-SIG-2Comp
1021	TSS_3907683-2	335	61	0,1	-14	1	-34	TTGTCAGTAT	-12	CGCAGAATGT	-141	1,1	7.95	12.20	s	230	Rmet_3595	0	Op1000r_2	NA
1022	TSS_3848372-2	334	45	0,1	-16	1	-40	TTGACTTGGC	-14	ACTACGATGG	-140	1,1	9.23	5.40	(m)-sba	109	Rmet_3548	0	Op0984r_1	NA
1023	TSS_2939902-2	334	56	0,1	-15	1	-35	TCGCCAGCTC	-13	GGTATTCTCT	-139	1,1	7.19	11.70	s	215	Rmet_2705	bacA	Op0766r_2	MET-PepGlyc
1024	TSS_1021122-3	334	48	0,4	-6	0	-24	GACTTCGTCG	-4	TGCATAAGCA	-141	3,0	6.11	-8.40	no	103	Rmet_4425	tnmB	Op1259r_1	NA
1025	TSS_1998331-2	334	130	0,1	-15	1	-45	TTGAATACGT	-13	GGCATGATAT	-143	1,1	8.03	-6.60	no	1131	Rmet_1842	bug	Op0534r_2	NA
1026	TSS_1856465-2	333	17	0,1	-15	1	-35	TTGCACGGCA	-13	CCTAGTCTGA	-138	1,1	6.21	10.20	s	6279	Rmet_1707	0	Op0482r_1	NA
1027	TSS_2000526+3	333	149	0,1	-16	1	-40	TCGGCCCTTT	-14	GGAAGAATGC	-142	5,1	8.04	2.90	(w)-sba	653	Rmet_5298	fliM	Op1566f_1	DIV-MOT-Chemotax
1028	TSS_3276994+2	332	152	0,1	-14	1	-33	TGGATTGATC	-12	GATAAAATCT	-145	1,1	8.56	9.10	s	7632	Rmet_3028	0	Op0849f_1	NA

1029	TSS_1552297-3	332	179	0,1	-16	1	-49	TTGAAACGAT	-14	TTCAGAATCC	-146	1,1	8.77	-12.60	no	46	Rmet_4889	prx5	Op1429r_1	GIP-PTL-Fold
1030	TSS_3158463+2	332	117	0,1	-17	1	-35	TCGATCCGCA	-15	CGTATAATAC	-137	1,1	8.14	11.10	s	74	Rmet_2905	mviN	Op0821f_2	EIP-TRA-Ion
1031	TSS_1653868-3	332	48	0,1	-15	1	-48	ATTCGGCGGG	-13	GCTATCCTTG	-141	2,1	10.52	-18.10	no	4940	Rmet_4971	0	Op1459r_1	NA
1032	TSS_3262672+2	331	67	0,1	-16	1	-49	ATTCGGCGGG	-14	GCTATCCTTG	-141	2,1	10.52	-19.10	no	9384	Rmet_3013	yodB	Op0847f_1	NA
1033	TSS_512727+3	331	22	0,1	-17	1	-37	TGGTGACCGT	-15	CGTATCATGC	-140	1,1	7.52	10.90	s	28	Rmet_3952	0	Op1108f_1	NA
1034	TSS_340963-2	331	35	0,4	-6	0	-47	TGGAGACCTC	-26	GCGCGAATTG	-146	1,1	2.07	-1.70	no	103	Rmet_0322	tnmB	Op0092r_1	NA
1035	TSS_387990-2	331	43	0,4	-16	1	-49	GCAGGAGAAC	-3	GAGAGCCTTT	-144	4,1	6.37	-32.60	no	145	Rmet_0365	coxS	Op0102r_1	MET-EN-Methane
1036	TSS_125974+5	330	65	0,1	-21	0	-43	TCGTGCTAGG	-19	GGTATGCTAC	-143	1,1	6.25	0.90	(w)-nu	29	Rmet_6271	0	Op1912f_1	NA
1037	TSS_92161+2	330	23	0,1	-22	0	-43	TCGTACAACT	-20	ACTACCCTCA	-143	1,1	4.63	0.90	w	83	Rmet_0085	0	Op0023f_2	NA
1038	TSS_1009723+2	329	50	0,1	-17	1	-39	TGGCGATGTC	-15	TTTACACTGC	-139	1,1	8.66	5.90	m	84	Rmet_0924	0	Op0267f_1	NA
1039	TSS_3087603-2	329	37	0,4	-6	0	-36	TGCAGTCCAT	-16	GCCATGCAGG	-149	1,1	-0.98	4.80	m	103	Rmet_2838	tnmB	Op0798r_1	NA
1040	TSS_1245731-3	328	130	0,1	-15	1	-35	GTAGACGCCT	-13	TTCATCATGC	-142	4,1	6.57	6.70	s	27	Rmet_4618	0	Op1331r_3	NA
1041	TSS_3915522+2	328	34	0,1	-15	1	-41	TGGCGAAAAT	-13	GTTACAATGC	-137	1,1	10.67	0.90	(w)-sba	18	Rmet_3603	0	Op1003f_1	NA
1042	TSS_149551+3	327	29	0,1	-27	0	-48	TTGACGGGGA	-25	TGGATGATGT	-142	1,1	7.58	0.40	w	634	Rmet_R0071	tRNA-I	Op1006f_1	NA
1043	TSS_556504-2	326	62	0,1	-14	1	-39	TTGCATTCGA	-12	CATAAAATCG	-138	1,1	9.54	4.40	m	26	Rmet_0520	ogt1	Op0140r_2	GIP-REP-Recomb
1044	TSS_1462203-2	326	64	0,1	-15	1	-39	CCGGCAGGCT	-13	GGCATCATCC	-137	5,1	10.31	1.90	w	426	Rmet_1352	comL	Op0380r_1	NA
1045	TSS_439685+2	325	38	0,1	-19	0	-48	GGCCCAGAGC	-17	GGCACGATTT	-140	4,1	7.61	-14.60	no	37	Rmet_0414	erpA	Op0113f_3	NA
1046	TSS_2117866+3	325	32	0,1	-13	1	-31	TCGCGACAGG	-11	AATATCATGT	-142	1,1	7.09	6.10	m	22	Rmet_5392	0	Op1606f_1	EIP-SIG-2Comp
1047	TSS_3407314-2	324	44	0,4	-6	0	-29	CCGGACAGGC	-2	CATAAGCAGC	-144	5,1	2.88	-7.30	no	103	Rmet_3143	tnmB	Op0880r_1	NA
1048	TSS_3551007-2	324	45	0,1	-15	1	-49	CCGGCCAGGG	-13	GATACAATCG	-134	5,1	12.18	-18.10	no	144	Rmet_3275	cyaY	Op2053r_1	NA
1049	TSS_3915481-2	324	18	0,1	-16	1	-40	TGCCGCATTG	-14	GTTAGAATTT	-141	1,1	7.68	1.40	(w)-sba	5640	Rmet_3597	0	Op1000r_1	NA
1050	TSS_490776-3	324	42	0,4	-6	0	-48	TGCAGGCACT	-28	GGCATCGTGC	-140	1,1	3.69	-5.20	no	103	Rmet_3932	tnmB	Op1099r_1	NA
1051	TSS_2662730+2	323	26	0,1	-16	1	-38	TATCTTACCC	-14	GCTATTCTTG	-147	2,1	8.20	4.40	m	173	Rmet_2451	fdxA	Op0685f_2	NA
1052	TSS_3875354-2	323	47	0,1	-17	1	-42	TCGTTACAGC	-15	TCTACACTCT	-142	1,1	6.58	-0.10	no	27	Rmet_3572	0	Op0992r_2	NA
1053	TSS_2740429-2	323	54	0,1	-16	1	-46	TTGCCGGCGC	-14	ATTACAATGC	-135	1,1	10.32	-6.60	no	2055	Rmet_2520	0	Op0696r_1	NA
1054	TSS_1027555+2	322	57	0,1	-16	1	-40	TGGACCTGTG	-14	GCTACGCTGG	-139	1,1	7.72	2.90	(w)-sba	24	Rmet_0941	0	Op0267f_3	NA
1055	TSS_474101+3	322	13	0,1	-27	0	-48	TTGACGGGGA	-25	TGGATGATGT	-142	1,1	7.58	0.40	w	634	Rmet_R0076	0	Op1098f_1	NA
1056	TSS_3509010-2	320	117	0,1	-15	1	-45	TTTCGTCGGC	-13	GTTAGAATGT	-137	2,1	13.73	-8.10	no	395	Rmet_3232	0	Op0916r_1	NA
1057	TSS_1505-5	320	52	0,1	-15	1	-37	TCGAAGCCTC	-13	CATACAATCA	-138	1,1	7.64	8.90	s	15605	Rmet_6301	trbF	Op1917r_1	NA
1058	TSS_117340-5	320	47	0,1	-7	0	-34	TTGAAGGCGA	-5	GGGACGATGG	-141	1,1	7.53	3.20	(w)-ba	60	Rmet_6262	0	Op1909r_1	NA
1059	TSS_2508309-2	320	54	0,1	-14	1	-49	TTGCGACTTC	-12	GTTACATTAC	-143	1,1	8.58	-15.60	no	256	Rmet_2285	0	Op0654r_2	EIP-TRA-Ion
1060	TSS_2324557+2	319	64	0,1	-15	1	-37	TGGTAGTCCA	-13	CGTACCCTTG	-143	1,1	5.29	5.90	m	36	Rmet_2136	0	Op0615f_1	NA
1061	TSS_2685581-2	319	72	0,1	-16	1	-41	TTCCCCTTCG	-14	TGTAGACTGT	-140	1,1	7.82	0.90	(w)-sba	219	Rmet_2471	asd	Op0688r_3	MET-AA-GlySerThr
1062	TSS_1242929+3	319	88	0,1	-16	1	-44	TAGCGAACGT	-14	AATACAATTC	-145	1,1	6.20	-4.10	no	100	Rmet_4617	piuA	Op1330f_1	EIP-TRA-Pores
1063	TSS_121818-4	319	68	0,1	-15	1	-38	TCGCAGATCT	-13	GCTACCATGG	-137	1,1	8.37	4.90	m	261	Rmet_6181	tnmB	Op1892r_1	NA
1064	TSS_2031167-2	318	50	0,1	-16	1	-41	TGGCGGCGGA	-14	GATAAACTGT	-139	1,1	8.04	-0.10	no	59	Rmet_1871	0	Op0540r_1	NA
1065	TSS_1175230-2	317	32	0,1	-16	1	-45	TCGGCAACCG	-14	TGGATAATCC	-139	5,1	9.96	-5.10	no	224	Rmet_1082	ppiB	Op0312r_3	GIP-PTL-Fold
1066	TSS_3615103-2	317	60	0,1	-2	0	-20	TGGAAATAGC	0	CGAAAACTAT	-146	1,1	5.04	-5.90	no	#NV	#NV	#NV	#NV	#NV
1067	TSS_757297-2	317	111	0,1	-14	1	-40	TTGCAACAGT	-12	CTTATAATGG	-138	1,1	11.44	4.40	(m)-sba	60	Rmet_0684	trxB	Op0192r_1	MET-NUC-Pyr
1068	TSS_1221261-3	317	65	0,1	-16	1	-35	TTGACATACG	-14	CATAGATTTC	-142	1,1	8.09	13.60	s	25	Rmet_4599	tnpR	Op1323r_1	NA
1069	TSS_2937085-2	316	13	0,1	-16	1	-44	TCAAAAATCT	-14	CGCACAATAC	-142	4,1	7.76	-5.60	no	26	Rmet_2703	0	Op0764r_1	NA
1070	TSS_1828740+3	316	94	0,3	-14	1	-21	TCGACAGCGC	-1	GACATTCTCA	-141	1,1	4.91	-3.90	no	4172	Rmet_5135	0	Op1514f_1	NA
1071	TSS_1512342+3	316	35	0,2	-22	0	-36	TGGACAAGCA	-15	AGTCCAATCG	-142	1,1	4.54	11.30	s	23	Rmet_4854	0	Op1416f_2	NA
1072	TSS_224037-4	316	11	0,1	-16	1	-35	TTCCAAACGG	-14	GGTAGATTGT	-138	1,1	7.81	10.10	s	743	Rmet_6388	0	Op2115r_1	NA
1073	TSS_1566184+2	315	60	0,1	-5	0	-36	TGGCCTTCAA	-15	CCTATGGTCA	-142	1,1	5.09	10.30	s	412	Rmet_1444	hlpA	Op0403f_3	NA
1074	TSS_3842300+2	315	16	0,1	-16	1	-37	TCACACGCGC	-14	GGTAGAATCC	-136	4,1	10.43	7.40	s	30	Rmet_3544	0	Op0983f_2	EIP-TRA-Ion
1075	TSS_2785936-2	315	63	0,1	-15	1	-35	TTTCGGTGGG	-13	GATAGACTAC	-142	2,1	12.55	9.70	s	1561	Rmet_2560	0	Op0708r_1	NA
1076	TSS_1798329+3	315	41	0,1	-16	1	-35	TTGCTTCCCA	-14	TGCAGCATGG	-141	1,1	5.47	9.60	s	82	Rmet_5106	0	Op1504f_3	NA
1077	TSS_744338+3	314	6	0,1	-15	1	-36	TTGACCGTCT	-13	TATATGCTCC	-142	1,1	8.25	12.40	s	209	Rmet_4162	0	Op1170f_4	NA
1078	TSS_963521+3	314	27	0,1	-16	1	-37	ATGCTGTGCT	-14	AGTAGCATCA	-151	2,1	6.50	6.40	s	123	Rmet_4366	0	Op1246f_1	NA
1079	TSS_301238+2	313	72	0,1	-16	1	-38	TTGCTGTGCC	-14	TCTACAATCA	-135	1,1	9.66	8.40	s	843	Rmet_0285	coaD	Op0083f_3	MET-COF-Pant
1080	TSS_3657060-2	312	27	0,1	-15	1	-41	TGGCAGACGA	-13	GGTACGCTAG	-142	1,1	6.84	-2.10	no	84	Rmet_3381	gspC	Op0940r_2	GIP-PTL-T2S
1081	TSS_137286+2	311	43	0,1	-18	1	-32	AAAGCTGCCG	-12	ACCTACGCGG	-144	3,0	4.31	-4.40	no	21	Rmet_0131	hslV	Op0033f_2	GIP-PTL-Fold
1082	TSS_944590+3	311	66	0,1	-16	1	-44	TTCTCATGAC	-14	AGTAATATTG	-142	1,1	7.02	-4.10	no	602	Rmet_4349	0	Op1238f_1	NA
1083	TSS_1518530-3	311	45	0,1	-16	1	-45	CCGGCAGGGT	-14	ACTACAATCG	-136	5,1	11.90	-8.10	no	28	Rmet_4859	0	Op1419r_5	NA
1084	TSS_1583482+3	310	38	0,1	-16	1	-46	GCAAAACAAG	-14	GCTATCATGG	-135	4,1	10.71	-11.60	no	351	Rmet_4920	0	Op1440f_3	NA
1085	TSS_3566782-2	310	80	0,1	-15	1	-38	TCGCTGACGG	-13	ACTACAATCG	-140	1,1	7.89	5.90	m	53	Rmet_3289	cutA	Op0926r_2	NA
1086	TSS_3083138-2	309	51	0,1	-2	0	-34	TTGACTGGGC	0	CGTAATTTCT	-144	1,1	6.24	-0.80	no	5438	Rmet_2828	0	Op0796r_1	NA
1087	TSS_2495509+2	309	52	0,1	-17	1	-37	GCGGCAGCCG	-15	TGTATGATTC	-136	5,1	11.10	8.90	s	1729	Rmet_2274	murI	Op0649f_3	MET-AA-GluGSH
1088	TSS_2572327-3	309	45	0,1	-16	1	-35	TCCCGCGCAG	-14	GGTAAAATAG	-139	1,1	7.16	8.60	s	91	Rmet_5808	prfC	Op1751r_2	NA
1089	TSS_226170+3	308	103	0,1	-9	0	-27	TTCACCCGTG	-7	AGTAAGGTTT	-144	1,1	4.98	1.10	(w)-nu	262	Rmet_3687	motA	Op1026f_3	EIP-TRA-Pores

1090	TSS_155198-3	308	94	0,4	-17	1	-33	TTGCGAACAA	-13	TATAAGATAT	-145	1,1	7.87	8.60	s	544	Rmet_3618	*ohrB*	Op1007r_3	NA	
1091	TSS_335337-2	307	32	0,1	-11	1	-22	TTGCAATCAG	0	GAAACGATAG	-143	1,1	5.94	-1.80	*no*	3219	Rmet_0313	*uvrA1*	Op0090r_1	GIP-REP-Recomb	
1092	TSS_3507532-2	306	79	0,1	-15	1	-36	TTCCCACGAT	-13	GGCATGATCA	-141	1,1	7.28	10.90	s	333	Rmet_3230	*petA*	Op0914r_1	MET-EN-OxPhos	
1093	TSS_38076+2	306	16	0,1	-15	1	-35	TTCACATCAA	-13	GTTACGATTG	-138	1,1	8.78	12.70	s	1215	Rmet_0037	*0*	Op0014f_1	NA	
1094	TSS_3382021+2	306	41	0,1	-16	1	-35	TCGGACGGGC	-14	GCTAGAATGC	-134	5,1	11.57	10.10	s	21528	Rmet_3139	*0*	Op0879f_1	NA	
1095	TSS_1030074+3	306	10	0,1	-17	1	-35	CCGGAAGTGG	-15	CGTAGACTGC	-136	5,1	9.50	6.10	m	82	Rmet_4434	*0*	Op1262f_1	NA	
1096	TSS_475995+3	306	34	0,1	-3	0	-21	TGGAAATAGC	-1	CGAAAACTAT	-147	1,1	5.04	-4.90	*no*	#NV	#NV	#NV	#NV	#NV	
1097	TSS_395990-3	306	90	0,1	-16	1	-34	TTGACAGAAA	-14	AATAGAATGC	-138	1,1	10.10	12.60	s	631	Rmet_3852	*0*	Op1071r_1	NA	
1098	TSS_2198172-3	305	125	0,2	-15	1	-34	TTGACACCAC	-13	CATACATTCG	-139	1,1	8.09	12.60	s	91	Rmet_5446	*0*	Op1619r_2	NA	
1099	TSS_3201455+2	305	45	0,1	-16	1	-45	GGAACGCACC	-14	CCTAGAATGC	-139	4,1	8.76	-9.60	*no*	64	Rmet_2945	*yajC*	Op0831f_2	GIP-PTL-Exp	
1100	TSS_2234245-2	305	19	0,1	-14	1	-34	TTGTTTTGCG	-12	GCTAGAATGG	-137	2,1	12.93	11.20	s	124	Rmet_2061	*0*	Op0598r_3	NA	
1101	TSS_57744+2	304	75	0,1	-3	0	-33	GGCCAAACGC	-1	GGTATGATTA	-139	4,1	10.93	-4.80	*no*	742	Rmet_0051	*mreB*	Op0017f_1	DIV-Division	
1102	TSS_1296760-2	304	39	0,4	-28	0	-34	TGGAACTGCG	-5	CAGAACATTA	-143	1,1	4.30	1.70	*(w)-ba*	189	Rmet_1183	*0*	Op0342r_2	NA	
1103	TSS_1071592+3	304	54	0,1	-17	1	-36	TTGACATACG	-15	CATAGATTTC	-142	1,1	8.09	13.80	s	24	Rmet_4473	*tnpR*	Op1276f_1	NA	
1104	TSS_2185890-2	304	48	0,2	-15	1	-37	TGGCTACGCT	-13	AGTAGAGTGT	-140	1,1	6.16	7.90	s	75	Rmet_2021	*0*	Op0590r_2	NA	
1105	TSS_2430454-2	304	33	0,1	-15	1	-34	GCAACGGAAC	-13	GCTATGTTTG	-138	4,1	8.72	5.60	m	62	Rmet_2216	*0*	Op0634r_2	NA	
1106	TSS_2464823-2	304	45	0,1	-4	0	-40	TTGCATAAAG	-14	GAAAGAATTC	-141	1,1	7.31	3.40	*(m)-sba*	27	Rmet_2249	*dat*	Op0644r_1	MET-AA-Lys_Deg	
1107	TSS_3448526-2	303	36	0,1	-16	1	-35	TTGCCCCTGC	-14	CGCACAATGC	-134	1,1	9.05	11.60	s	46	Rmet_3175	*apaG*	Op0892r_1	NA	
1108	TSS_3792512-2	303	61	0,1	-15	1	-39	TGGCGCGGCT	-13	GATACGCTGG	-140	1,1	6.82	1.90	w	488	Rmet_3501	*atpI*	Op0970r_1	MET-EN-OxPhos	
1109	TSS_102449-2	303	43	0,1	-4	0	-26	GCGGCAAGTT	-2	GGCAGCATGG	-138	5,1	8.83	-3.30	*no*	30	Rmet_0095	*0*	Op0028r_1		0
1110	TSS_250260-2	303	20	0,3	-3	0	-36	TTGCCTGTCA	-14	TATACAGTGA	-141	1,1	7.36	12.40	s	40	Rmet_0232	*0*	Op0068r_2	GIP-REP-Recomb	
1111	TSS_2318602-3	303	49	0,1	-15	1	-34	TTGCAGTTCT	-13	GATATTCTCG	-140	1,1	8.55	9.60	s	3036	Rmet_5562	*0*	Op1651r_1	NA	
1112	TSS_578600-2	302	66	0,1	-4	0	-34	TTGCATTAAT	-14	ATTATTCTTC	-143	1,1	7.91	8.60	s	100	Rmet_0542	*0*	Op0150r_1	NA	
1113	TSS_2286424-2	302	79	0,1	-16	1	-36	TGCAAGTGGC	-14	GTTATCATTG	-139	1,1	7.94	10.40	s	236	Rmet_2104	*0*	Op0608r_1	NA	
1114	TSS_2331998-2	302	33	0,1	-14	1	-41	TTCCAGGGCG	-12	CGTATAATGC	-138	1,1	9.62	-0.10	*no*	58	Rmet_2140	*gltX*	Op0616r_1	MET-AA-GluGSH	
1115	TSS_630974-3	302	49	0,4	-29	0	-36	TTGACACTTT	-15	TCTTTAATAA	-144	1,1	4.50	13.80	s	47	Rmet_4065	*napE*	Op1141r_1	MET-EN-Nitrog	
1116	TSS_1969681-3	301	105	0,1	-15	1	-35	TTGTCATGAA	-13	GATAGATTTC	-144	1,1	8.24	13.20	s	19	Rmet_5270	*0*	Op1553r_1	NA	
1117	TSS_2965981-2	301	40	0,1	-27	0	-43	TTGCGGGGGG	-12	GCTATGATGG	-142	1,1	10.07	-3.60	*no*	67	Rmet_2729	*0*	Op0770r_1	NA	
1118	TSS_3556242-2	301	93	0,1	-25	0	-44	TTGAGGTGGG	-23	CATCACATCG	-148	1,1	3.09	1.80	*w*	46	Rmet_3281	*0*	Op0924r_2	NA	
1119	TSS_75119-5	301	21	0,1	-15	1	-39	TCGTCAGTTT	-13	GCCACAATGC	-143	1,1	7.83	4.90	m	316	Rmet_6222	*0*	Op1899r_1	NA	
1120	TSS_768464+2	300	55	0,1	-15	1	-35	TTGCGCTCCG	-13	CCTATAATCT	-139	1,1	10.07	13.20	s	32	Rmet_R0006	*0*	Op2002f_1	NA	
1121	TSS_1536172+3	300	47	0,1	-17	1	-47	CTGAGTGAGG	-15	ACCATACTGG	-144	1,1	2.82	-11.60	*no*	35	Rmet_6692	*0*	Op1424f_1	NA	
1122	TSS_48568+5	300	44	0,1	-17	1	-41	TTCCGCCTAA	-15	CGTACAATGC	-139	1,1	8.94	1.90	*(w)-sba*	3732	Rmet_6204	*chrI*	Op1896f_1	NA	
1123	TSS_1063784-2	300	46	0,1	-16	1	-39	TGGCGGGAGA	-14	GGCAAAATCG	-139	1,1	7.83	3.90	m	25	Rmet_0977	*0*	Op0274r_1	NA	
1124	TSS_3558122-2	300	83	0,1	-14	1	-46	TTCCAGATGA	-12	TGTATCTTGG	-142	1,1	6.50	-12.10	*no*	409	Rmet_3282	*0*	Op0924r_1	NA	
1125	TSS_403583-3	300	22	0,1	-16	1	-45	TCGCACGACG	-14	TGTAAAGTAC	-142	1,1	6.06	-8.10	*no*	33	Rmet_3860	*selD*	Op1073r_2	MET-OAA-Other	
1126	TSS_163786-3	299	36	0,1	-15	1	-47	GCGGAAACCC	-13	GCTACAATGG	-135	5,1	12.16	-14.10	*no*	65	Rmet_3626	*moxR*	Op1009r_1	NA	
1127	TSS_148590+3	298	31	0,1	-9	0	-36	TCGAAAGAGC	-7	CAGAGATTGA	-145	1,1	3.81	3.90	*(m)-ba*	4	Rmet_R0070	*0*	Op1006f_1	NA	
1128	TSS_473140+3	298	40	0,1	-9	0	-36	TCGAAAGAGC	-7	CAGAGATTGA	-145	1,1	3.81	3.90	*(m)-ba*	4	Rmet_R0075	*0*	Op1098f_1	NA	
1129	TSS_2338217-2	298	15	0,1	-16	1	-37	TGGCTACTAC	-14	GGTACAGTGC	-138	1,1	7.96	8.90	s	137	Rmet_2145	*yejB*	Op0618r_1	NA	
1130	TSS_2500463-2	298	97	0,1	-15	1	-42	TCGCCGATGC	-13	GCTAGGATTA	-139	1,1	7.68	-2.10	*no*	87	Rmet_2275	*0*	Op0650r_1	NA	
1131	TSS_1479861-3	298	56	0,1	-15	1	-34	TTGCCAGCGC	-13	CCTATGGTTG	-135	1,1	7.46	11.60	s	41	Rmet_4826	*mdtD*	Op1407r_1	NA	
1132	TSS_2102208-2	297	51	0,2	-4	0	-31	GAATTGCTCG	-11	TGTCGAACCC	-143	3,0	5.01	-3.40	no	2103	Rmet_1931	*amaB-2*	Op0564r_1	MET-NUC-Pyr	
1133	TSS_2962490-2	297	61	0,1	-15	1	-33	TCGAGCGGAG	-13	GATAGAATGA	-140	1,1	8.27	8.10	s	204	Rmet_2726	*0*	Op0770r_1	NA	
1134	TSS_994543-2	296	45	0,1	-15	1	-49	CCAGACCGCG	-13	GGCATAATGG	-136	5,1	10.33	-20.60	*no*	21	Rmet_0907	*0*	Op0262r_2	NA	
1135	TSS_1415458-3	296	55	0,1	-15	1	-45	TTCTAACAAG	-13	GCTATCATTA	-144	1,1	7.69	-7.10	*no*	29	Rmet_4770	*0*	Op1387r_1	MET-AA-GlySerThr	
1136	TSS_40521+4	296	42	0,1	-16	1	-46	TTGCGCGTGA	-14	CGTATCCTTT	-140	1,1	7.69	-8.60	*no*	250	Rmet_6017	*0*	Op1835f_1	NA	
1137	TSS_2150912-2	295	55	0,1	-16	1	-47	TCGCCGTGAT	-14	TGTAAAATAT	-141	1,1	8.90	-10.10	*no*	101	Rmet_1974	*0*	Op0576r_2	NA	
1138	TSS_3616997-2	294	42	0,1	-26	0	-47	TTGACGGGGA	-24	TGGATGATGT	-142	1,1	7.58	1.40	*w*	635	Rmet_R0063	*0*	Op0930r_1	NA	
1139	TSS_3529965-2	294	39	0,1	-15	1	-41	TGGAACCGGC	-13	CTTATAATGG	-141	1,1	9.81	1.90	*(w)-sba*	21	Rmet_3259	*yrbF*	Op0918r_2	EIP-TRA-ABC	
1140	TSS_1265940-2	294	18	0,1	-7	0	-33	TTGAACCAAA	-5	AGCAGGATTG	-143	1,1	6.46	3.20	*(w)-ba*	60	Rmet_1156	*bdhA*	Op0332r_1	MET-CAH-But	
1141	TSS_2307998-2	293	111	0,1	-15	1	-35	TTCCGCATCA	-13	GGCACACTCC	-139	1,1	6.78	8.70	s	48	Rmet_2125	*0*	Op0614r_2	NA	
1142	TSS_151445+3	293	74	0,1	-3	0	-21	TGGAAATAGC	-1	CGAAAACTAT	-147	1,1	5.04	-4.90	*no*	#NV	#NV	#NV	#NV	#NV	
1143	TSS_1355545-2	293	52	0,1	-16	1	-41	CCGTCACCAG	-14	GGCACAATGC	-139	5,1	8.87	-1.10	*no*	22	Rmet_1232	*yhgF*	Op0356r_1	NA	
1144	TSS_1130122-2	292	20	0,1	-15	1	-33	TGGCGCCGGC	-13	TTTACAATCT	-139	1,1	8.76	7.10	s	4	Rmet_1039	*0*	Op0298r_1	NA	
1145	TSS_2255934+3	292	46	0,1	-16	1	-40	TCGCCACCAT	-14	GAGAGAATAT	-143	1,1	6.11	3.90	*(m)-sba*	2151	Rmet_5512	*0*	Op1640f_1	MET-CAH-Prop	
1146	TSS_2511525-3	292	12	0,1	-16	1	-46	TCGACCGCTA	-14	GGTATAGTGC	-141	1,1	8.16	-7.10	*no*	2313	Rmet_6765	*0*	Op1719r_1	NA	
1147	TSS_1063776+2	292	36	0,1	-16	1	-39	TGGCCGGTGT	-14	GCTACAATCT	-136	1,1	10.12	5.90	m	23	Rmet_0978	*0*	Op0275f_1	NA	
1148	TSS_89166+2	291	1	0,1	-17	1	-35	GGAGCGGCGC	-15	TATAGAATGG	-139	4,1	9.48	5.60	m	25	Rmet_0082	*0*	Op0023f_1	NA	
1149	TSS_1509120-2	291	41	0,1	-20	0	-46	GGACGAAAAT	-18	GACATGCTGT	-142	4,1	7.74	-10.60	*no*	240	Rmet_1399	*0*	Op0392r_1	NA	
1150	TSS_2523446+3	291	87	0,1	-18	1	-38	TGGCTGCGGG	-16	GTGATAGTTT	-142	1,1	5.05	7.90	s	8	Rmet_5762	*0*	Op1728f_2	NA	

1151	TSS_2648107-2	290	42	0,1	-15	1	-42	GGACGGGCCG	-13	TGTAAAATGC	-139	4,1	9.06	-6.60	no	180	Rmet_2439	*rluC*	Op0682r_1	GIP-TL-Ass
1152	TSS_3236634+2	290	17	0,1	-15	1	-37	TTCCAGCGCG	-13	GAGACCATGA	-141	1,1	4.91	5.90	m	445	Rmet_2978	*0*	Op0839f_1	NA
1153	TSS_1501648-2	290	13	0,1	-15	1	-37	TTGATTCTCC	-13	CGTATTATGA	-140	1,1	7.96	10.40	s	205	Rmet_1391	*0*	Op0388r_1	NA
1154	TSS_3710752+2	290	30	0,1	-15	1	-46	TCGATGACCG	-13	GCTACAATGA	-139	1,1	8.66	-9.10	no	30	Rmet_3433	*ycbL*	Op0953f_1	MET-CAH-Pyr
1155	TSS_3192087+2	289	90	0,1	-16	1	-48	TTGACCGCAA	-14	TCTATAATGA	-139	1,1	10.73	-8.60	no	49	Rmet_2937	*yggS*	Op0829f_1	NA
1156	TSS_2160537-2	289	86	0,1	-15	1	-48	TTCCCGGAAT	-13	GCTACTATCA	-141	1,1	7.77	-14.10	no	27	Rmet_1986	*0*	Op0580r_1	NA
1157	TSS_1967001+3	288	20	0,1	-17	1	-47	TGACGGGCGG	-15	GGTACCATCC	-140	4,1	7.41	-12.60	no	6	Rmet_5268	*alkB2*	Op1552f_2	GIP-REP-Recomb
1158	TSS_607265+2	288	39	0,1	-16	1	-39	GCACACGACG	-14	CCTACAATAC	-139	4,1	8.78	0.40	w	28	Rmet_0566	*moaA1*	Op0159f_1	NA
1159	TSS_3620385+2	288	29	0,1	-16	1	-49	TCGCACAGGG	-14	TGTATAGTGA	-142	1,1	7.40	-15.10	no	493	Rmet_3344	*0*	Op0931f_4	NA
1160	TSS_1000344+2	287	28	0,1	-16	1	-37	TGGCGGGGGC	-14	GCTATCCTTG	-138	1,1	7.57	7.90	s	152	Rmet_0915	*psd*	Op0263f_3	MET-LIP-GlycPLip
1161	TSS_3179359+2	287	24	0,1	-16	1	-49	GCGTACCGGG	-14	CGTAGAATGG	-139	5,1	8.67	-18.10	no	1472	Rmet_2925	*0*	Op0827f_1	NA
1162	TSS_1290271-3	287	38	0,1	-28	0	-44	TTGTCGATCT	-24	AAGAATATCA	-144	1,1	4.51	1.80	w	3462	Rmet_4654	*0*	Op1345r_1	NA
1163	TSS_3431764-2	286	17	0,1	-26	0	-47	TTGACGGGGA	-24	TGGATGATGT	-142	1,1	7.58	1.40	w	635	Rmet_R0053	*0*	Op0886r_1	NA
1164	TSS_2508711-2	286	39	0,1	-16	1	-35	TTGCCGAGGG	-14	GCTATAATTG	-138	1,1	12.13	13.60	s	192	Rmet_R0038	*0*	Op0654r_2	NA
1165	TSS_1146291+3	286	85	0,1	-16	1	-37	TTGACCTCAA	-14	TTGATACTGG	-140	1,1	7.78	11.40	s	21	Rmet_4539	*0*	Op1300f_1	NA
1166	TSS_2667729+2	285	62	0,1	-17	1	-39	GCAACAATAC	-15	TGCATAATGG	-140	4,1	10.83	4.40	m	21	Rmet_2457	*nodI*	Op0687f_1	NA
1167	TSS_1099317+2	284	39	0,1	-17	1	-39	TTGTGGGCGC	-15	GGTACAATCG	-137	1,1	10.08	7.40	s	29	Rmet_1009	*rutE*	Op0289f_2	NA
1168	TSS_3434147-2	284	56	0,1	-13	1	-36	TTGACCTGCT	-14	GCCAGTATCG	-138	1,1	7.47	12.40	s	46	Rmet_3162	*paaF*	Op0886r_1	MET-CAH-Prop
1169	TSS_268677-2	284	51	0,1	-15	1	-38	TTCCGTTGGG	-13	TATAAAATAC	-139	2,1	13.06	5.90	m	98	Rmet_0251	*gpmA*	Op0072r_1	MET-CAH-FbP
1170	TSS_1724117-3	284	48	0,1	-16	1	-37	CCGGCTCGCC	-14	GCCACAATCC	-138	5,1	9.48	5.90	m	30	Rmet_5037	*0*	Op1481r_2	NA
1171	TSS_968454+3	284	79	0,1	-16	1	-34	AATCGTCGGG	-14	ACTAGCATCC	-144	2,1	9.30	2.60	w	24	Rmet_4370	*0*	Op1246f_3	MET-CAH-FbP
1172	TSS_2500636+2	283	27	0,1	-16	1	-48	TTGCACAATG	-14	TTTATACTGT	-142	1,1	9.84	-11.60	no	176	Rmet_2277	*tonB*	Op0651f_2	NA
1173	TSS_3750874+2	283	42	0,1	-16	1	-34	TGGCAGACAC	-14	ACTAATATGG	-141	1,1	7.18	7.10	s	45	Rmet_3471	*0*	Op0965f_1	NA
1174	TSS_2049372-2	283	60	0,1	-16	1	-41	TTCCACAAAC	-14	GGTATGCTGT	-141	1,1	8.13	-0.10	no	117	Rmet_1883	*lon*	Op0544r_2	GIP-PTL-Fold
1175	TSS_258597+2	283	29	0,2	-16	1	-35	TTGTCTTTAA	-14	CATAGAGTGC	-144	1,1	6.69	11.60	s	271	Rmet_0242	*gshA*	Op0071f_3	MET-AA-GluGSH
1176	TSS_559247-3	283	37	0,1	-14	1	-40	TTGCAGGTTG	-12	CATATAGTGA	-138	1,1	7.79	2.40	(w)-sba	44	Rmet_3990	*folE*	Op1119r_2	MET-COF-Folate
1177	TSS_600076-3	283	25	0,1	-15	1	-40	GTACACCCCG	-13	CGTATAATGC	-142	4,1	8.90	-0.10	no	134	Rmet_4029	*0*	Op1135r_1	NA
1178	TSS_3467751+2	282	45	0,1	-16	1	-36	TTGCCTGGAT	-14	TGTACGCTGG	-139	1,1	8.21	11.40	s	163	Rmet_3192	*0*	Op0897f_2	NA
1179	TSS_1115289+2	282	101	0,1	-16	1	-47	TCGCGCAGTC	-14	AGTAAACTAT	-142	1,1	6.73	-12.10	no	41	Rmet_1025	*iscS*	Op0295f_3	NA
1180	TSS_873849-2	281	62	0,1	-16	1	-45	GGCAAAGTGA	-14	TATATAATTC	-140	4,1	9.62	-8.60	no	77	Rmet_0797	*0*	Op0226r_1	EIP-TRA-ABC
1181	TSS_3434284+2	280	25	0,1	-16	1	-45	TGGTCGGTTA	-14	ACTACAATGG	-140	1,1	8.18	-6.10	no	44	Rmet_3163	*paaG*	Op0887f_1	MET-CAH-Prop
1182	TSS_495595-2	280	28	0,4	-17	1	-46	TTGTCGTCCA	-15	TGTATAAATA	-143	1,1	4.60	-6.60	no	203	Rmet_0465	*0*	Op0122r_1	EIP-SIG-2Comp
1183	TSS_3307493-2	279	51	0,1	-16	1	-36	TGCAAACAAT	-14	GGTAAAATTG	-138	1,1	9.38	11.40	s	134	Rmet_3050	*pyrF*	Op0858r_1	MET-NUC-Pyr
1184	TSS_1913341-3	279	63	0,1	-14	1	-49	GCAGGCCCAC	-12	GATATACTTT	-140	4,1	9.91	-21.60	no	775	Rmet_5217	*0*	Op1537r_1	NA
1185	TSS_1155186+2	279	69	0,1	-17	1	-45	TTGTCGGTCG	-15	GCTATAATCC	-135	1,1	10.72	-2.60	no	84	Rmet_1062	*0*	Op0305f_1	NA
1186	TSS_1459650+3	278	83	0,1	-16	1	-41	TCGCCCTGAT	-14	TCTACAATGC	-138	1,1	9.82	1.90	(w)-sba	575	Rmet_4811	*0*	Op1400f_1	NA
1187	TSS_137306-2	278	59	0,1	-17	1	-47	TTGCCGCGGC	-15	GTGATACTGT	-137	1,1	8.31	-8.60	no	2464	Rmet_0128	*zur*	Op0032r_1	NA
1188	TSS_2033306+3	278	19	0,1	-17	1	-37	TTGACGGGTT	-15	CCTACAGTAG	-140	1,1	7.89	12.40	s	3	Rmet_5323	*zniR*	Op1574f_1	NA
1189	TSS_409616+2	277	50	0,1	-16	1	-37	TCTGAACGAC	-14	CTTAGACTTC	-143	5,1	9.15	7.40	s	105	Rmet_0384	*0*	Op0105f_2	NA
1190	TSS_3009868-2	276	26	0,1	-14	1	-43	GGCCACAAGA	-12	GTTAGCATTG	-142	4,1	7.29	-10.60	no	40	Rmet_2767	*mrp*	Op0776r_1	DIV-Division
1191	TSS_2018023-3	276	37	0,1	-21	0	-44	TGGTATGGGT	-19	GCTATGCTGA	-138	1,1	6.74	-1.10	no	45	Rmet_5310	*0*	Op1569r_1	NA
1192	TSS_2373750-3	276	61	0,1	-16	1	-33	TCGAACCTCG	-7	TCTCAAATGA	-147	1,1	3.35	3.70	(m)-ba	43	Rmet_5616	*bug*	Op1675r_1	NA
1193	TSS_2171411+3	275	62	0,1	-17	1	-47	GCAGACACGT	-15	GCTAAGATCT	-137	4,1	10.05	-15.60	no	119	Rmet_5424	*0*	Op1614f_1	NA
1194	TSS_3137477+2	275	55	0,1	-17	1	-42	TTCTTAGGGG	-15	GGTATGCTCC	-142	1,1	6.74	-0.10	no	1130	Rmet_2885	*ileS*	Op0811f_1	MET-AA-ValLeuIle
1195	TSS_2372608-3	275	57	0,1	-16	1	-39	TTCCTGATGA	-14	GGTATGCTGT	-139	1,1	7.49	3.90	m	273	Rmet_6752	*0*	Op1675r_2	NA
1196	TSS_336462+2	274	31	0,1	-17	1	-35	TTGCGGACTG	-15	GGTACGATTC	-138	1,1	9.51	9.60	s	487	Rmet_0318	*0*	Op0091f_2	NA
1197	TSS_2565786-3	274	33	0,1	-16	1	-36	TCAGCACTTC	-14	TTTAGAATCA	-143	5,1	8.90	11.40	s	102	Rmet_5805	*0*	Op1749r_1	NA
1198	TSS_438027+3	274	56	0,1	-10	1	-46	TTGTAACGGC	-8	GAAATATTAC	-148	1,1	5.37	-13.60	no	151	Rmet_3894	*0*	Op1086f_2	NA
1199	TSS_2560413+3	273	29	0,5	-19	0	-37	TGGCGGAACA	-17	GCGTCAATCC	-143	1,1	1.09	5.30	m	5545	Rmet_5806	*fecA1*	Op1750f_1	EIP-TRA-Pores
1200	TSS_23533+4	273	13	0,1	-8	0	-36	TTGACAAATA	-6	CATAACATGT	-141	1,1	8.79	6.40	(s)-ba	142	Rmet_6041	*0*	Op1841f_2	NA
1201	TSS_115102+4	273	29	0,1	-17	1	-37	TGGCATCGTG	-15	ATTACAATCA	-141	1,1	7.81	9.90	s	39	Rmet_5945	*pbrT*	Op1807f_2	EIP-TRA-Ion
1202	TSS_687468-3	273	43	0,1	-17	1	-45	GCAGAAACGG	-15	ATTAGAATTC	-141	4,1	10.72	-9.60	no	55	Rmet_4116	*psd*	Op1159r_3	MET-LIP-GlycPLip
1203	TSS_2292021-2	272	38	0,1	-19	0	-43	TTCCCGGGCA	-17	CTGATTGTCT	-145	1,1	2.49	-1.10	no	35	Rmet_2109	*yfgB*	Op0608r_1	NA
1204	TSS_856331-2	272	33	0,1	-15	1	-42	TTGTCGAGCG	-13	GGTATCATGG	-136	1,1	9.57	0.40	(w)-sba	110	Rmet_0778	*rng*	Op0222r_3	GIP-TL-Ass
1205	TSS_2181963-2	272	42	0,1	-15	1	-34	TTGCCTTCCT	-13	GTTAGCGTAT	-145	1,1	5.91	9.60	s	10220	Rmet_2001	*tnmB*	Op2023r_1	NA
1206	TSS_1855859+3	272	52	0,1	-12	1	-37	TTGACCGACT	-10	AATAGAATTT	-145	1,1	9.48	8.40	s	52	Rmet_5157	*0*	Op1518f_1	NA
1207	TSS_148625+3	271	42	0,1	-24	0	-36	TTGAACTGAA	-12	CTCAGATTGA	-142	1,1	5.81	9.40	s	1560	Rmet_R0071	*tRNA-I*	Op1006f_1	NA
1208	TSS_2493677-2	271	39	0,1	-15	1	-45	TTCAGGTATA	-13	GCTAACTTAG	-142	1,1	5.42	-9.10	no	162	Rmet_2270	*acs*	Op0648r_2	MET-CAH-FbP
1209	TSS_3870054+2	270	58	0,1	-17	1	-37	CCGGCAATCG	-15	GGCATGATCC	-138	5,1	9.76	7.90	s	27	Rmet_3568	*0*	Op0989f_1	NA
1210	TSS_605278+3	269	73	0,1	-16	1	-38	TTGCGGATCG	-14	GGTACGCTGG	-139	1,1	8.50	6.40	s	471	Rmet_4039	*mmfR*	Op2064f_1	NA
1211	TSS_1005260+2	269	102	0,1	-17	1	-37	TTGCCGGTCC	-15	CTTAACATCG	-140	1,1	8.21	11.40	s	47	Rmet_0920	*0*	Op0265f_1	EIP-TRA-ABC

1212	TSS_2903612+2	269	36	0,1	-16	1	-42	TAGACAGAGA	-14	TGTATCCTAT	-146	1,1	4.05	0.90	(w)-sba	9270	Rmet_2680	glyA	Op0757f_1	MET-EN-Methane
1213	TSS_19632+2	268	58	0,1	-16	1	-42	GTCACCCTGT	-14	GCTAATCTGA	-145	4,1	5.28	-4.10	no	88	Rmet_0016	0	Op0003f_2	NA
1214	TSS_988814+2	268	23	0,1	-16	1	-49	TCGCCGCACG	-14	CTTATAATCG	-138	1,1	8.72	-13.10	no	28	Rmet_0901	0	Op0261f_2	NA
1215	TSS_959957-2	268	110	0,1	-15	1	-41	TGGCATCCCG	-13	GCTACAATGA	-139	1,1	8.93	-0.10	no	68	Rmet_0876	pepN	Op0254r_2	MET-OAA-GSH
1216	TSS_1656418-3	268	62	0,1	-15	1	-37	TTGCGGATCG	-13	GGTACGCTGG	-139	1,1	8.50	7.40	s	472	Rmet_4978	mmfR	Op1461r_1	EIP-SIG-2Comp
1217	TSS_1955917+2	267	64	0,1	-5	0	-38	AAACTTGTCA	-18	GCAGGCGTAG	-140	3,0	8.88	-2.20	no	49	Rmet_1803	ynjF	Op0523f_2	NA
1218	TSS_1931206-2	267	68	0,1	-15	1	-42	GCGGCCGCCG	-13	GGCATGATTG	-145	5,1	9.56	-5.10	no	40	Rmet_1778	0	Op0516r_1	NA
1219	TSS_2473454+3	267	81	0,1	-16	1	-35	TTGATCTTTG	-14	TCTAGGATAA	-141	1,1	7.16	11.60	s	943	Rmet_5716	0	Op1710f_1	NA
1220	TSS_3366824+2	267	59	0,1	-17	1	-36	TTCCCATCCG	-15	GCTAAACTTT	-140	1,1	8.75	11.30	s	40	Rmet_R0049	0	Op0875f_1	NA
1221	TSS_3513635-2	267	32	0,1	-16	1	-42	TTGCGGTCCC	-14	GCTAGAATGA	-140	1,1	10.17	0.40	(w)-sba	303	Rmet_3237	tatA	Op0918r_3	GIP-PTL-Exp
1222	TSS_3685214-2	265	9	0,1	-15	1	-43	TTCAGACCAC	-21	GTAAAAGTGC	-147	1,1	4.59	3.90	m	4825	Rmet_3401	0	Op0944r_1	NA
1223	TSS_3175175-2	265	37	0,1	-15	1	-39	TGGCCGGTTT	-13	GCGATAATGC	-139	1,1	8.66	4.90	m	8745	Rmet_2911	purM	Op0824r_1	MET-NUC-Pur
1224	TSS_1473469+2	265	24	0,1	-17	1	-35	TGGCCTGACT	-15	GGTAAGCTCG	-139	1,1	7.07	8.10	s	27	Rmet_1364	metC	Op0383f_4	MET-EN-Nitrog
1225	TSS_1051553-2	265	38	0,1	-15	1	-38	TGCCCAACAG	-13	GCTCTAATGC	-144	1,1	4.75	5.40	m	27	Rmet_0967	gstI	Op0270r_1	MET-OAA-GSH
1226	TSS_16390+4	265	74	0,1	-16	1	-35	TTGATTGATC	-14	CGTATGATGG	-143	1,1	8.85	12.60	s	30	Rmet_6049	0	Op1843f_1	NA
1227	TSS_3790329+2	264	44	0,1	-24	0	-35	TGGATGTTGG	-14	TGCATGATGA	-143	1,1	6.16	10.10	s	1842	Rmet_3502	0	Op0971f_1	NA
1228	TSS_781051-2	264	108	0,1	-14	1	-33	TGGCGGCCCG	-12	GCAAGTATAC	-146	1,1	3.19	6.10	m	150	Rmet_0704	cphA2	Op0198r_1	NA
1229	TSS_2950703-2	264	47	0,1	-14	1	-32	TGGCGCTGAC	-12	GGCACAATAC	-139	1,1	7.95	5.10	m	82	Rmet_2716	cpsG	Op0768r_1	MET-CAH-FbP
1230	TSS_2369648+2	263	35	0,1	-17	1	-40	TCCACAAGCT	-15	CCTATAATCG	-139	1,1	8.69	6.40	(s)-sba	288	Rmet_2167	0	Op0623f_1	GIP-PTL-Exp
1231	TSS_1126217+3	263	43	0,1	-17	1	-35	TTCAGCCTTT	-15	GGCATACTTC	-140	1,1	7.65	9.10	s	71	Rmet_4521	0	Op1294f_1	NA
1232	TSS_1592523+3	261	28	0,1	-16	1	-36	TCGGCAACCT	-14	TTTACCATCC	-138	5,1	9.93	11.90	s	25	Rmet_4929	gst	Op1444f_3	MET-OAA-GSH
1233	TSS_132069-2	261	69	0,1	-7	0	-40	TTGCCTTGAT	-13	ACTACCATTG	-141	1,1	8.89	3.40	(m)-sba	79	Rmet_0125	cobW3	Op0032r_3	NA
1234	TSS_1469106+2	260	51	0,1	-17	1	-36	TCGACATCCA	-15	CCGAGAATTC	-142	1,1	6.41	12.30	s	322	Rmet_1360	0	Op0383f_2	NA
1235	TSS_2642936+2	260	27	0,1	-17	1	-37	TGGCGCATCG	-15	GCTAAGATGC	-136	1,1	8.27	8.90	s	178	Rmet_6526	0	Op0681f_1	NA
1236	TSS_3403347-2	260	88	0,1	-15	1	-38	TTGCGGGCCT	-13	GGTACAATGG	-135	1,1	11.08	7.40	s	82	Rmet_3138	0	Op0878r_1	MET-COF-CoQ
1237	TSS_914863+2	260	33	0,1	-24	0	-37	TTGACAATGT	-15	TTTACATTTA	-144	1,1	9.10	13.40	s	275	Rmet_0837	0	Op0239f_1	EIP-TRA-Pores
1238	TSS_124451-5	259	25	0,1	-15	1	-37	TGGACCCGAA	-13	TGGACAATGT	-139	1,1	7.22	8.90	s	7171	Rmet_6262	0	Op1909r_1	NA
1239	TSS_2315075-3	258	77	0,1	-15	1	-33	TTGACGCCCT	-13	GTCACCATGA	-143	1,1	7.27	8.60	s	23	Rmet_5561	0	Op1651r_1	NA
1240	TSS_575056+2	257	48	0,1	-17	1	-43	TCGTCGTGTT	-15	CGCATAATCC	-141	1,1	7.21	-1.10	no	26	Rmet_0540	0	Op0149f_4	NA
1241	TSS_546070+3	257	15	0,1	-16	1	-43	TTCCCATTTC	-21	TAGATGATGT	-142	1,1	6.25	4.90	m	1540	Rmet_3981	0	Op1116f_1	NA
1242	TSS_334666+2	257	21	0,1	-1	0	-40	TCGACGTCCT	-15	GTTAGGATTC	-143	1,1	7.88	4.90	(m)-sba	36	Rmet_0316	0	Op0091f_2	NA
1243	TSS_2304624-2	257	39	0,1	-15	1	-43	TTGATCCCGA	-13	GCTAGCATCG	-137	1,1	8.14	-2.60	no	58	Rmet_2122	0	Op0614r_2	EIP-TRA-ABC
1244	TSS_3198998+2	255	52	0,1	-16	1	-35	GGCGAAGCCT	-14	CTTACAATGT	-138	4,1	9.43	6.60	s	90	Rmet_2943	queA	Op0831f_1	GIP-TL-Ass
1245	TSS_1460596-2	255	83	0,1	-15	1	-34	TGGATGCCGT	-13	GCCATCATGT	-140	1,1	6.53	9.10	s	12314	Rmet_1338	0	Op0378r_1	NA
1246	TSS_3748195-2	255	4	0,1	-16	1	-41	TTGCTCCACA	-14	GCTATGATGC	-137	1,1	9.53	2.40	(w)-sba	611	Rmet_3467	0	Op0964r_2	NA
1247	TSS_1841471+2	255	51	0,1	-2	0	-20	TTGCGATTTA	0	GGCACACTCT	-141	1,1	8.36	-5.40	no	72	Rmet_1700	0	Op0481f_2	NA
1248	TSS_2664646+2	254	105	0,1	-25	0	-45	TTGCACGAAAC	-23	GTTATAATTT	-141	1,1	12.04	6.40	s	220	Rmet_2453	0	Op0685f_2	NA
1249	TSS_888158-2	254	21	0,1	-15	1	-45	GCCCCGGCGT	-13	AGTAAAATCA	-141	4,1	8.68	-11.60	no	57	Rmet_0814	0	Op0230r_1	NA
1250	TSS_3077691+2	253	31	0,1	-16	1	-35	TTGCAGTGTG	-14	AATATTATGA	-142	1,1	8.31	11.60	s	23	Rmet_2829	0	Op0797f_1	NA
1251	TSS_2544940+3	253	73	0,1	-16	1	-40	GCTGAACGTC	-14	ACTACAATGC	-137	5,1	10.57	-0.60	no	26	Rmet_5787	0	Op1740f_2	NA
1252	TSS_1144204-3	253	58	0,1	-15	1	-39	GCTGAATTCG	-13	GAGACGATGC	-143	5,1	6.38	-1.60	no	45	Rmet_4536	0	Op1297r_1	NA
1253	TSS_1105642+2	253	37	0,1	-16	1	-45	GGAGGAGCAG	-14	GCCATAATCC	-139	4,1	9.68	-10.60	no	19	Rmet_1016	0	Op0293f_1	NA
1254	TSS_3895346-2	253	61	0,1	-15	1	-34	TGGCTGAAAC	-13	CGTATTATAG	-141	1,1	6.98	9.10	s	225	Rmet_3587	acrR	Op0998r_1	NA
1255	TSS_80923+3	253	26	0,1	-17	1	-37	CTGTCGTGTC	-15	GGTATGATGC	-139	1,1	5.42	9.40	s	4845	Rmet_5888	0	Op1784f_1	NA
1256	TSS_276368-3	252	48	0,2	-18	1	-44	CCGGAAATGC	-16	CATCCGATAA	-145	5,1	2.89	-8.10	no	349	Rmet_3731	flgN	Op1041r_2	DIV-MOT-Flagel
1257	TSS_1163950-2	252	39	0,1	-14	1	-38	TGGCTCCAGA	-12	GTAATAATGC	-143	1,1	6.85	4.90	m	228	Rmet_1071	0	Op0308r_2	NA
1258	TSS_2024671+2	251	44	0,1	-15	1	-36	TCGCGTAGCC	-13	TGTATGATGC	-137	1,1	8.01	9.90	s	115	Rmet_1868	0	Op0539f_2	GIP-PTL-Fold
1259	TSS_3326731+2	251	33	0,1	-20	0	-33	GAACTTGCCA	-13	GGTCAATGAC	-143	3,0	6.95	-2.40	no	421	Rmet_3070	0	Op0861f_5	NA
1260	TSS_3539346-2	251	45	0,1	-15	1	-40	GCCCACCAAT	-13	CGTATCATTG	-141	4,1	8.40	-2.60	no	21	Rmet_3264	0	Op0920r_2	GIP-REP-Recomb
1261	TSS_3722348-2	251	23	0,1	-15	1	-48	TAGCGCCCCG	-13	CTCACAATGT	-140	2,1	8.19	-15.10	no	3724	Rmet_3443	0	Op0958r_1	MET-NUC-Pur
1262	TSS_1406629+3	251	174	0,1	-16	1	-34	TTCCAAGCTG	-14	CACAAAATGT	-143	1,1	6.63	8.10	s	44	Rmet_4761	0	Op1386f_1	NA
1263	TSS_1414256-3	251	30	0,1	-16	1	-36	TGGCGATGAC	-14	GATATGATTG	-139	1,1	9.09	11.90	s	156	Rmet_4769	kdgK	Op1387r_1	MET-CAH-PP_KDPG
1264	TSS_2483614+2	250	34	0,1	-17	1	-48	TCCATCGGTG	-15	GTTACAATGC	-142	1,1	7.77	-12.60	no	1250	Rmet_6515	0	Op0645f_5	NA
1265	TSS_607620+2	250	5	0,1	-15	1	-35	TATCGGCGCG	-13	GGTACCATAC	-137	2,1	12.28	8.20	s	26	Rmet_0567	modE	Op0159f_1	EIP-TRA-ABC
1266	TSS_3848536-2	250	10	0,1	-20	0	-37	TGGCCCGAAT	-11	TGTCAAATGC	-144	1,1	4.85	4.90	m	273	Rmet_3548	0	Op0984r_1	NA
1267	TSS_3432690-2	250	22	0,1	-23	0	-35	TTGAACTGAA	-11	CTCAGATTGA	-143	1,1	5.81	9.20	s	1561	Rmet_R0053	0	Op0886r_1	NA
1268	TSS_3814066-2	250	32	0,1	-16	1	-34	GCGGAAGCAC	-14	GCGATGATTT	-138	5,1	8.49	5.10	m	43	Rmet_3521	betA2	Op0974r_3	MET-AA-GlySerThr
1269	TSS_1299181+2	249	150	0,1	-17	1	-36	TTTGGGTGCG	-15	CGTAGACTGC	-139	2,1	12.22	9.30	s	10	Rmet_1186	atzB	Op0343f_1	MET-XEN-Others
1270	TSS_2793611+2	249	30	0,1	-16	1	-36	TCGTGCGGCT	-14	GCTAAAATCC	-137	1,1	8.46	10.90	s	41	Rmet_2568	thyA	Op0711f_1	MET-NUC-Pyr
1271	TSS_473175+3	249	29	0,1	-24	0	-36	TTGAACTGAA	-12	CTCAGATTGA	-142	1,1	5.81	9.40	s	1560	Rmet_R0076	0	Op1098f_1	NA
1272	TSS_1165041+2	248	12	0,1	-14	1	-48	TCGCGCGACA	-12	TGTCGAATCA	-144	1,1	2.51	-17.10	no	111	Rmet_1073	slyD	Op0309f_1	GIP-PTL-Fold

1273	TSS_3762732+2	247	30	0,1	-17	1	-43	TCGCGACCGC	-15	GTTAAACTGC	-138	1,1	8.44	-2.10	*no*	132	Rmet_3481	*gcvH*		Op0969f_2	NA
1274	TSS_269105-3	247	39	0,1	-16	1	-41	TTCCATATGC	-14	ACTACACTAA	-139	1,1	6.90	-0.10	*no*	3864	Rmet_3723		*0*	Op1037r_1	DIV-MOT-Chemotax
1275	TSS_33968+2	247	48	0,1	-16	1	-44	TTGCCCCCGC	-14	GTTAAACTGA	-139	1,1	9.49	-3.60	*no*	11	Rmet_0030	*tnpA*		Op2000f_1	NA
1276	TSS_60360+4	247	63	0,1	-17	1	-37	TTCCAAGCTC	-15	AATACAATCA	-141	1,1	8.10	10.90	s	3450	Rmet_5994	*gntR*		Op1827f_1	NA
1277	TSS_729499+3	246	34	0,1	-16	1	-41	GCAGACATCT	-14	TGTAACATCT	-142	5,1	8.52	-4.60	*no*	326	Rmet_4154	*tnpA*		Op1170f_1	NA
1278	TSS_1362491+3	246	43	0,1	-16	1	-34	TGCAGATTTC	-14	CGTATGATGC	-135	1,1	7.59	8.60	s	43	Rmet_4720		*0*	Op1362f_1	MET-EN-Sulf
1279	TSS_1119638-3	246	63	0,1	-15	1	-33	TCGCAATGGC	-13	GTTAAGATTC	-143	1,1	8.78	7.10	s	0	Rmet_4514	*int*		Op1289r_1	NA
1280	TSS_2573319-3	246	46	0,1	-15	1	-34	TTGACGGTGC	-13	CTTATGCTTG	-138	1,1	8.35	11.60	s	21	Rmet_5809		*0*	Op1751r_1	NA
1281	TSS_2799440-2	246	67	0,1	-15	1	-32	TGGCCATCGG	-5	TATAAATTGT	-141	1,1	7.26	3.70	*(m)-ba*	75	Rmet_2574	*orn*		Op0714r_1	NA
1282	TSS_3624459+2	245	45	0,1	-16	1	-35	TCGACTGGTC	-14	GATAGGCTGC	-143	1,1	6.28	10.10	s	3939	Rmet_3352		*0*	Op0933f_1	NA
1283	TSS_904558-2	245	25	0,1	-16	1	-35	TGGTGCTTGG	-14	GCTACAGTGC	-137	1,1	7.18	9.10	s	136	Rmet_0829		*0*	Op0236r_2	NA
1284	TSS_1121556-2	244	32	0,1	-14	1	-47	TTGAACCAGG	-12	GTTAAGATGG	-140	1,1	9.60	-11.60	*no*	12490	Rmet_1018	*tyrB*		Op0294r_1	MET-AA-Met
1285	TSS_2647940+2	244	7	0,1	-16	1	-46	TTGCAATTTG	-14	AATATAATTT	-139	1,1	10.72	-5.60	*no*	962	Rmet_2440	*rne*		Op0683f_1	GIP-TL-Ass
1286	TSS_1109212-2	244	24	0,1	-15	1	-33	TGAGGAAAAC	-13	GCTAGAATCT	-139	4,1	10.54	6.60	s	146	Rmet_1018	*tyrB*		Op0294r_1	MET-AA-Met
1287	TSS_306660+3	244	46	0,1	-16	1	-44	TTGCCCCCGC	-14	GTTAAACTGA	-140	1,1	9.49	-3.60	*no*	11	Rmet_3770	*tnpA*		Op1048f_1	NA
1288	TSS_1142288+2	244	34	0,1	-16	1	-45	CCAGCAGTCG	-14	GGTAAACTTC	-138	5,1	10.77	-10.60	*no*	195	Rmet_1049		*0*	Op0301f_1	NA
1289	TSS_163664-4	244	50	0,1	-15	1	-34	TGGACAGCTA	-13	CCGAAAATCC	-138	1,1	6.79	11.10	s	1599	Rmet_6138		*0*	Op1876r_1	NA
1290	TSS_2945060+2	243	31	0,1	-16	1	-36	GCACGCCAAC	-14	GTTACCTTGC	-143	4,1	7.39	4.40	m	5772	Rmet_2717		*0*	Op0769f_1	NA
1291	TSS_1979424+2	243	23	0,1	-15	1	-48	TGGAGGCATC	-13	GCGACAATGC	-141	1,1	7.71	-14.10	*no*	134	Rmet_1823		*0*	Op0529f_1	NA
1292	TSS_2190132+2	243	47	0,1	-17	1	-36	TTGCCGTATC	-15	CTTATATTTG	-143	1,1	8.98	12.80	s	553	Rmet_2026		*0*	Op0593f_1	NA
1293	TSS_3179276-2	243	72	0,1	-15	1	-44	TCCCCGGTAC	-13	TCCATAATTG	-140	1,1	6.55	-6.60	*no*	32	Rmet_2923	*cobO*		Op0826r_1	MET-COF-Porph
1294	TSS_3568567+2	242	35	0,2	-16	1	-34	TCGATTCGGC	-14	AATACAATTC	-141	1,1	7.80	9.10	s	643	Rmet_6589		*0*	Op2054f_1	NA
1295	TSS_858710+3	242	53	0,1	-17	1	-36	TTGCATCGAT	-15	TCTAGTATAT	-146	1,1	7.40	10.80	s	40	Rmet_4268	*citA*		Op1212f_1	MET-CAH-TCA
1296	TSS_3196667-2	242	55	0,1	-16	1	-47	TTGCCTGTCG	-14	GCTAAAATCT	-141	1,1	10.46	-8.60	*no*	20	Rmet_2941	*oxyR*		Op0830r_2	NA
1297	TSS_1425877+3	242	6	0,1	-16	1	-41	TGCCGGTTTA	-14	GCTAAGGTCG	-141	1,1	4.51	-2.60	*no*	22	Rmet_4782		*0*	Op1392f_1	NA
1298	TSS_3284609+2	242	30	0,1	-13	1	-38	TTGTAGCCCT	-11	GTCATCTTGG	-146	1,1	5.05	3.40	m	17	Rmet_3028		*0*	Op0849f_1	NA
1299	TSS_2175926-3	241	41	0,1	-16	1	-36	TCACGCCTGG	-14	AGTAGAGTGA	-146	4,1	4.33	8.40	s	28	Rmet_5429		*0*	Op1615r_1	NA
1300	TSS_110254-5	241	48	0,1	-15	1	-42	GCAGCAACAA	-13	GCTATGATCT	-141	4,1	9.54	-4.60	*no*	153	Rmet_6257		*0*	Op1905r_1	NA
1301	TSS_2144535+2	241	63	0,1	-16	1	-36	TTGACAACCG	-14	TATAAGATAT	-141	1,1	6.64	9.90	s	156	Rmet_1968		*0*	Op0575f_1	NA
1302	TSS_1764283+3	241	45	0,1	-14	1	-45	GTAGGAGCGG	-12	AGTAAAATGG	-142	4,1	9.92	-11.10	*no*	595	Rmet_5076		*0*	Op1494f_1	NA
1303	TSS_522129+3	240	35	0,1	-17	1	-36	TGGCATGGTG	-15	ACTAGACTGA	-138	1,1	6.61	9.30	s	40	Rmet_3962		*0*	Op1112f_1	NA
1304	TSS_41175-5	240	6	0,1	-15	1	-37	GGCGAGCAGG	-13	CGTATCCTTG	-144	4,1	6.21	2.40	w	9461	Rmet_6344	*merR*		Op1929r_1	NA
1305	TSS_1729184-3	240	11	0,1	-14	1	-46	TTGGCAGACC	-12	GAGACAATGG	-141	1,1	8.81	-9.60	*no*	41	Rmet_5041		*0*	Op1483r_1	NA
1306	TSS_1478092-2	239	50	0,1	-15	1	-36	GCGGAAGCAG	-13	GTTACAATAC	-139	5,1	11.33	7.90	s	75	Rmet_1368	*serB*		Op0384r_1	MET-AA-GlySerThr
1307	TSS_574632-3	239	28	0,1	-15	1	-38	TGGCCTGTCC	-13	CGTAGAATTC	-141	1,1	8.59	6.90	s	34	Rmet_4002		*0*	Op1123r_1	NA
1308	TSS_963458-3	239	47	0,1	-16	1	-48	TTCAAACTTA	-17	TTTATTATGA	-147	1,1	7.75	-8.10	*no*	30	Rmet_4365	*cbbR3*		Op1245r_1	NA
1309	TSS_110510-5	239	28	0,1	-16	1	-36	TTGCGGCCAG	-14	CTTAACCTGA	-140	1,1	6.22	10.40	s	409	Rmet_6257		*0*	Op1905r_1	NA
1310	TSS_1831610+2	239	38	0,1	-21	0	-41	TGCAGGAGGA	-19	GAAAGAGTGT	-146	1,1	2.60	3.40	*(m)-nu*	1647	Rmet_6470		*0*	Op2014f_1	NA
1311	TSS_3218255-2	238	45	0,1	-15	1	-33	TGCACGTTCG	-13	GGTAAAATGC	-138	1,1	9.18	7.60	s	11	Rmet_2959		*0*	Op0834r_2	NA
1312	TSS_3617923-2	238	51	0,1	-23	0	-35	TTGAACTGAA	-11	CTCAGATTGA	-143	1,1	5.81	9.20	s	1561	Rmet_R0063		*0*	Op0930r_1	NA
1313	TSS_3638072+2	238	49	0,1	-16	1	-41	TTGTGCTGGC	-14	CGTACTCTGT	-142	1,1	6.71	0.40	*(w)-sba*	7725	Rmet_3369	*paaA*		Op0937f_1	NA
1314	TSS_2325472-3	237	38	0,1	-17	1	-40	TGGACTCCAT	-15	GGGACAATCG	-141	1,1	7.60	4.90	*(m)-sba*	45	Rmet_5571		*0*	Op1657r_2	NA
1315	TSS_1857573-2	237	21	0,1	-16	1	-35	TTGCATTTCA	-14	CCTATCATCC	-138	1,1	8.91	11.60	s	1067	Rmet_1714		*0*	Op0484r_2	NA
1316	TSS_1697044+2	236	33	0,1	-15	1	-46	TTGCGGCACA	-13	TCTACAATGT	-139	1,1	10.01	-8.60	*no*	59	Rmet_1577	*betB*		Op0435f_1	MET-AA-GlySerThr
1317	TSS_316133-2	236	41	0,4	-7	0	-35	GTCAACGCGC	-4	GCTTCAATGG	-145	4,1	4.95	-2.30	*no*	67	Rmet_0298		*0*	Op0086r_3	NA
1318	TSS_630761-2	235	28	0,1	-17	1	-39	TTGATGGGAA	-15	GGGAGTCTCT	-146	1,1	4.68	5.40	m	415	Rmet_0587		*0*	Op0166r_3	NA
1319	TSS_2295016-2	235	29	0,1	-15	1	-46	CCTGCCCCCT	-13	ACTACAATCG	-138	5,1	10.77	-13.60	*no*	2555	Rmet_2110	*ndk*		Op0608r_1	MET-NUC-Pur
1320	TSS_3260122+2	235	69	0,1	-16	1	-38	TTGCGGATCG	-14	GGTACGCTGG	-139	1,1	8.50	6.40	s	471	Rmet_3004	*mmfR*		Op0845f_1	EIP-SIG-2Comp
1321	TSS_2453719+3	235	14	0,1	-16	1	-35	TTGACAGCCG	-14	CAAATACTGC	-139	1,1	6.52	13.60	s	104	Rmet_5695		*0*	Op1702f_1	NA
1322	TSS_3039690+2	234	96	0,1	-14	1	-23	CCGGCCACGT	0	ATCACTTTGA	-144	5,1	5.03	-7.30	*no*	22	Rmet_2793		*0*	Op0785f_2	NA
1323	TSS_2505866+2	234	77	0,1	-18	1	-42	TCGACGTACA	-16	GGTAATATTG	-143	1,1	8.07	1.90	*(w)-sba*	22	Rmet_2284	*yeiE*		Op0653f_1	NA
1324	TSS_3388516+2	234	65	0,1	-15	1	-34	GCACAGGTAT	-13	AGTAAAATAC	-140	4,1	9.82	4.60	m	15033	Rmet_3139		*0*	Op0879f_1	NA
1325	TSS_3149484-2	233	47	0,3	-4	0	-48	GCTGACCGTC	-14	GGTATCTATA	-148	5,1	2.23	-20.60	*no*	83	Rmet_2892	*clpA*		Op0812r_2	GIP-PTL-Fold
1326	TSS_1912670-3	233	45	0,1	-15	1	-33	TTCAGGCGGA	-13	CCTATAATTA	-137	1,1	8.94	9.10	s	104	Rmet_5217		*0*	Op1537r_1	NA
1327	TSS_2910009-2	232	15	0,1	-15	1	-33	TCGATAACGC	-13	AACATAATGC	-142	1,1	7.29	9.10	s	166	Rmet_2675	*tolB*		Op0756r_3	NA
1328	TSS_73396+4	232	22	0,1	-17	1	-46	TCGTGAAGGT	-15	GGTAAATTCT	-141	1,1	7.14	-8.10	*no*	643	Rmet_5984	*czcN*		Op1821f_1	NA
1329	TSS_2979622+2	232	22	0,1	-21	0	-45	TGGATGGGAC	-19	GCTATGTTAG	-143	1,1	5.90	-2.10	*no*	1625	Rmet_2746		*0*	Op0773f_1	NA
1330	TSS_3195309-2	232	17	0,1	-16	1	-37	TTCCCTTGTG	-14	GCGATAATGG	-137	1,1	8.22	9.90	s	63	Rmet_2940	*dpsA*		Op0830r_3	GIP-REP-Recomb
1331	TSS_2399544-2	232	30	0,1	-29	0	-32	GAACTGGCCG	-12	TGGTCAACCC	-145	3,0	4.13	-3.40	*no*	656	Rmet_2189	*rrmJ*		Op0628r_1	DIV-Division
1332	TSS_1235060-3	232	184	0,1	-15	1	-36	TGGCACTGAT	-13	GGTATAGTCG	-139	1,1	8.82	9.90	s	174	Rmet_4608		*0*	Op1327r_1	NA
1333	TSS_105188+2	231	9	0,1	-11	1	-44	GCGGCAGGCG	-9	CATAGCCTGA	-139	5,1	8.10	-13.10	*no*	90	Rmet_0098	*dsbA*		Op0029f_1	GIP-PTL-Fold

1334	TSS_1753926+2	231	43	0,1	-16	1	-44	GCCAGAAAAG	-14	CCTATAATCG	-139	4,1	11.12	-6.60	no	2731	Rmet_1628	0	Op0455f_1	NA
1335	TSS_2232648-3	231	21	0,1	-14	1	-33	TGGCCCTATT	-12	AGCAGAATAG	-140	1,1	6.71	8.10	s	127	Rmet_5488	0	Op1629r_1	GIP-REP-Recomb
1336	TSS_2852971-2	231	38	0,1	-14	1	-34	TTGCAAAGAC	-12	CGTACAATGC	-136	1,1	11.39	12.20	s	33	Rmet_2621	zupT	Op0734r_2	EIP-TRA-Ion
1337	TSS_1237626+2	231	21	0,1	-16	1	-46	TGGCGGGGCG	-14	CGTATTATAT	-142	1,1	6.59	-9.10	no	38	Rmet_1131	rbn	Op0323f_1	NA
1338	TSS_781069+2	229	32	0,1	-5	0	-23	TTGCGTTATG	-3	GGCAAAATTG	-138	1,1	9.10	-2.40	no	75	Rmet_0705	0	Op0199f_1	EIP-TRA-ABC
1339	TSS_1836337+2	229	29	0,1	-14	1	-35	TTGCGGGCAG	-12	TATAAGATCA	-140	1,1	7.40	10.20	s	62	Rmet_1695	0	Op0481f_1	NA
1340	TSS_2418635-3	229	28	0,1	-15	1	-41	GCAGCACCGG	-13	GGCATGATTG	-139	5,1	8.87	-3.60	no	24	Rmet_5659	0	Op1691r_2	NA
1341	TSS_2246795-3	229	50	0,1	-14	1	-36	TCGAGCCGAC	-12	GGTATGATGG	-138	1,1	8.77	9.90	s	4141	Rmet_5498	0	Op1635r_1	NA
1342	TSS_1291213+3	229	34	0,1	-16	1	-44	TTGCCCCCGC	-14	GTTAAACTGA	-140	1,1	9.49	-3.60	no	11	Rmet_4658	tnpA	Op2076f_1	NA
1343	TSS_522002-3	228	5	0,1	-16	1	-42	TCTGCCAGTG	-14	GTTAAGCTTG	-140	5,1	8.59	-3.60	no	65	Rmet_3961	gabD	Op1111r_1	MET-CAH-But
1344	TSS_2509383+3	228	33	0,1	-17	1	-47	TGGCGGCTTC	-15	TGTATCGTAT	-144	1,1	5.03	-11.10	no	36	Rmet_5749	0	Op1720f_1	NA
1345	TSS_2114557+2	228	32	0,1	-17	1	-43	CCTGAAGGCG	-15	GCTACAATCC	-133	5,1	12.11	-5.60	no	40	Rmet_1944	0	Op0569f_1	MET-LIP-Ster
1346	TSS_1774746+3	228	12	0,1	-16	1	-35	TTGCGTATTC	-14	AATACACTCG	-142	1,1	8.30	10.60	s	59	Rmet_5086	0	Op1498f_1	NA
1347	TSS_3923671-2	227	18	0,1	-15	1	-39	TGGCGTTGGT	-13	CCTACACTGC	-138	1,1	8.24	2.90	w	49	Rmet_3611	0	Op1004r_1	NA
1348	TSS_1881167+2	227	62	0,1	-16	1	-43	TTGAGGATTG	-14	CGCAGAATTG	-143	1,1	8.09	-1.60	no	21	Rmet_1736	cybB	Op0493f_1	NA
1349	TSS_1176498-2	227	28	0,1	-15	1	-44	TTGCGACCTG	-13	GTTAGAATGC	-135	1,1	10.47	-3.60	no	829	Rmet_1083	ppiA	Op0312r_2	GIP-PTL-Fold
1350	TSS_2438957-2	227	35	0,1	-15	1	-42	TTGTCAGGAT	-13	GGCATACTAG	-138	1,1	8.19	0.40	(w)-sba	64	Rmet_2224	rfaC	Op0636r_2	MET-GLYC-LPS
1351	TSS_1567702+2	227	54	0,1	-16	1	-35	TCATCTACGC	-14	TATATCATCG	-146	5,1	5.52	9.60	s	586	Rmet_1446	fabZ	Op0403f_3	MET-LIP-FASyn
1352	TSS_20316+2	226	45	0,1	-16	1	-41	TTCCGCCAGC	-14	CATATAATTA	-140	1,1	8.57	1.90	(w)-sba	52	Rmet_0017	0	Op0003f_2	NA
1353	TSS_2531281-3	226	78	0,1	-14	1	-42	TTTCGCTTCC	-12	ACTACAATGG	-136	2,1	13.00	-3.10	no	40	Rmet_5770	panE	Op1733r_2	MET-COF-Pant
1354	TSS_3613063-2	226	83	0,1	-16	1	-46	TTGATAGGCT	-14	GTTAAGCTAA	-142	1,1	7.24	-7.60	no	144	Rmet_R0060	0	Op0930r_1	NA
1355	TSS_1453574+3	226	47	0,1	-16	1	-36	TCGACATGGC	-14	GGTAGTGTCT	-140	1,1	5.96	11.90	s	63	Rmet_4806	0	Op1398f_1	NA
1356	TSS_3807458-2	226	56	0,1	-16	1	-46	CCGCGAGAAC	-14	GTTACGATGT	-144	1,1	3.41	-12.10	no	697	Rmet_3515	0	Op0974r_5	NA
1357	TSS_2339179+3	226	39	0,1	-21	0	-35	TGGCAAGAGA	-14	GCTGTAATGG	-142	1,1	4.42	10.10	s	31	Rmet_5586	0	Op1664f_1	NA
1358	TSS_2352005-3	226	7	0,1	-15	1	-35	TTGCCGCCGG	-13	GGTATGCTGC	-136	1,1	9.19	12.20	s	8	Rmet_5597	0	Op1667r_3	NA
1359	TSS_1701189+2	225	32	0,1	-16	1	-39	TGGAAATCAT	-14	AGTACCATCA	-141	1,1	7.61	5.90	m	23	Rmet_1579	0	Op0435f_2	MET-NUC-Pur
1360	TSS_469157-2	225	34	0,2	-16	1	-46	TTGACGGCCG	-14	AATAGAATGG	-139	1,1	9.20	-5.60	no	32	Rmet_0442	gloA	Op0118r_4	MET-CAH-Pyr
1361	TSS_1829466-2	225	21	0,1	-16	1	-46	TGGTCCTGGT	-14	GGCAAACTCA	-141	1,1	6.34	-10.10	no	1722	Rmet_1687	0	Op0476r_1	NA
1362	TSS_2868992-2	225	27	0,1	-16	1	-43	TTCCGCCCGC	-14	ACTACAATAG	-139	1,1	7.89	-3.10	no	30	Rmet_2632	0	Op0738r_2	NA
1363	TSS_2643155-2	224	15	0,1	-15	1	-42	GCAGAAGCTG	-13	TGTATACTTT	-141	5,1	9.29	-5.60	no	101	Rmet_2433	0	Op0680r_1	NA
1364	TSS_1430699-3	224	30	0,1	-17	1	-46	TTGACCGCGA	-15	CGTATCATTC	-140	1,1	9.36	-4.60	no	31	Rmet_4786	0	Op1393r_1	NA
1365	TSS_569685-2	223	34	0,1	-15	1	-35	TCCTGATCAC	-13	CGTAAAATGC	-140	1,1	7.04	10.20	s	174	Rmet_0534	0	Op0148r_1	NA
1366	TSS_2163622-2	223	46	0,1	-11	1	-49	TCAAGCGCCC	-9	TATACACTTC	-144	4,1	7.95	-21.60	no	508	Rmet_1989	dus	Op0582r_1	GIP-TL-Ass
1367	TSS_2267775+3	223	28	0,1	-17	1	-45	TTGAGGAGAT	-15	GATAGACTCA	-141	1,1	9.01	-4.60	no	3803	Rmet_5525	0	Op1642f_1	NA
1368	TSS_184252+2	223	36	0,1	0	0	-36	GCGGAACTAC	-1	CGTACTATGC	-141	5,1	9.34	-5.10	no	196	Rmet_0177	0	Op0053f_1	MET-EN-OxPhos
1369	TSS_285202+3	223	101	0,5	-17	1	-44	TTGCCGCGCT	-12	GTTATTCAGC	-144	1,1	3.58	-7.60	no	57	Rmet_3743	flgK	Op1042f_3	DIV-MOT-Flagel
1370	TSS_3318514-2	222	68	0,1	-16	1	-44	GCGGACGCAG	-14	GGTACAATCG	-136	5,1	11.69	-8.10	no	8	Rmet_3060	mpl	Op0860r_1	NA
1371	TSS_519139+3	222	30	0,1	-16	1	-35	TTCTCATGGC	-14	ATTACACTTT	-138	1,1	7.51	11.10	s	82	Rmet_3960	0	Op1110f_3	NA
1372	TSS_949566+2	222	20	0,1	-16	1	-48	TGGTGCCGGC	-14	GATAAAATCC	-136	1,1	8.50	-13.10	no	71	Rmet_0869	0	Op0251f_1	NA
1373	TSS_3839639-2	222	25	0,1	-15	1	-44	GCCAAAGTGG	-13	AATACACTGC	-142	4,1	8.74	-9.60	no	2699	Rmet_3539	0	Op0980r_1	NA
1374	TSS_3547675+2	221	17	0,1	0	0	-36	TTGAACTCAG	-10	CTAACGGTGC	-143	1,1	3.84	6.40	s	15	Rmet_6587	0	Op2052f_1	NA
1375	TSS_2628964-2	221	38	0,1	-15	1	-40	TGCACTTACA	-13	TGTAAAGTGC	-142	1,1	6.11	1.40	(w)-sba	201	Rmet_2419	0	Op0680r_4	NA
1376	TSS_2190114+2	221	8	0,1	-16	1	-34	TGGCAAATTT	-14	CGTATCATCA	-144	1,1	8.22	9.10	s	571	Rmet_2026	0	Op0593f_1	NA
1377	TSS_810855-2	221	4	0,1	-15	1	-41	TCGACGAGGC	-13	GCTATACTCG	-137	1,1	9.52	1.90	(w)-sba	12713	Rmet_0720	0	Op0204r_1	NA
1378	TSS_1410487+3	221	36	0,1	0	0	-36	TTGCTCAAGA	-14	GCGACGATAT	-142	1,1	6.27	10.40	s	203	Rmet_4766	0	Op1386f_2	NA
1379	TSS_981224+2	221	29	0,1	-17	1	-38	TTGACCGTTT	-15	CTTACACTAT	-138	1,1	8.61	10.40	s	53	Rmet_0894	0	Op0259f_1	NA
1380	TSS_2091278+2	221	21	0,1	-15	1	-38	TTTGGCTTCC	-13	TGTACCATTA	-142	2,1	9.78	4.90	m	22	Rmet_1924	0	Op0561f_1	NA
1381	TSS_883438+3	221	24	0,1	-17	1	-37	TTCATACCGG	-15	GCGACAATGT	-143	1,1	7.18	10.90	s	46	Rmet_4292	prpR	Op1220f_1	NA
1382	TSS_568332-3	221	18	0,1	-15	1	-40	TGGCAAGGCT	-13	GCTACAATGC	-134	1,1	10.67	2.90	(w)-sba	25	Rmet_3997	0	Op1123r_3	NA
1383	TSS_69254-4	221	53	0,1	-20	0	-34	TGGCACCCGC	-13	ACTACATTGA	-141	1,1	6.44	8.10	s	55	Rmet_5989	0	Op1824r_2	NA
1384	TSS_3761301+2	220	19	0,1	-17	1	-36	TTGACAAAGG	-15	CGTACATTCA	-134	1,1	8.64	13.80	s	332	Rmet_3480	gcvT	Op0969f_1	MET-EN-Nitrog
1385	TSS_1095188+2	220	26	0,1	-6	0	-34	TTGAAAAATC	-4	GCTAGACTTG	-140	1,1	10.34	4.20	(m)-ba	78	Rmet_1004	grpE	Op0287f_1	GIP-PTL-Fold
1386	TSS_1466262+2	220	14	0,1	0	0	-35	TTGAACCCGC	-12	GTCAAACAGC	-142	1,1	2.92	8.20	s	81	Rmet_1357	phaA	Op0383f_2	MET-CAH-Pyr
1387	TSS_513418-3	220	38	0,1	-15	1	-39	TACAGCCTTG	-13	CCTATACTGG	-146	1,1	3.89	3.40	m	26	Rmet_3953	0	Op1109r_1	NA
1388	TSS_981021-2	220	59	0,1	-15	1	-40	GGCCGAATAT	-13	GCTATGTTTC	-140	4,1	7.88	-2.60	no	21	Rmet_0893	xdhA	Op0258r_1	NA
1389	TSS_278265-2	219	20	0,3	-10	1	-27	TTGCGCCGCC	-5	GATACAACCC	-143	1,1	2.19	2.20	(w)-nu	1638	Rmet_0258	gntX	Op0074r_1	NA
1390	TSS_1562999+3	219	48	0,1	-17	1	-37	TCAAGCGCAC	-15	CGTAAAATCG	-140	4,1	9.81	9.40	s	31	Rmet_4899	0	Op1434f_2	NA
1391	TSS_478035+3	219	53	0,1	-17	1	-47	TTGATAGGCT	-15	GTTAAGCTAA	-142	1,1	7.24	-8.60	no	143	Rmet_R0079	0	Op1098f_1	NA
1392	TSS_3070956+2	219	20	0,1	-16	1	-36	GCACCGCAAG	-14	GCTATCCTAC	-138	4,1	6.92	6.40	s	33	Rmet_2823	0	Op0795f_1	NA
1393	TSS_1083168-2	219	83	0,1	-4	0	-22	TGGAGAGCGG	-2	CGGATAATCT	-141	1,1	7.11	-1.90	no	107	Rmet_0993	0	Op0282r_1	NA
1394	TSS_1410540+3	218	22	0,1	-16	1	-41	TCGAATTACG	-14	CGTATTATCT	-143	1,1	7.13	1.90	(w)-sba	150	Rmet_4766	0	Op1386f_2	NA

1395	TSS_3874437+2	218	68	0,1	-16	1	-47	TGGACGGCGC	-14	CCTAATATCG	-145	1,1	7.07	-10.10	no	44	Rmet_3571	copQ1	Op0991f_1	NA
1396	TSS_3427830-2	218	45	0,1	-16	1	-46	TTGATAGGCT	-14	GTTAAGCTAA	-142	1,1	7.24	-7.60	no	144	Rmet_R0050	0	Op0886r_1	NA
1397	TSS_945786+3	218	22	0,1	-7	0	-28	TTGCCAGTCC	-5	GGCAATCTTG	-141	1,1	6.79	3.20	(w)-nu	138	Rmet_6660	0	Op1238f_2	NA
1398	TSS_133050-2	217	45	0,1	-15	1	-42	TTCTCCCATA	-13	AGTACAATAG	-143	1,1	7.46	-1.10	no	178	Rmet_0126	dksA1	Op0032r_3	NA
1399	TSS_319310-2	217	106	0,4	-6	0	-34	TCAAGAAAGC	-13	ACGAAATTGC	-143	4,1	6.87	7.60	s	743	Rmet_0302	rpoX	Op0086r_2	GIP-TL-Ass
1400	TSS_2493546-2	217	155	0,1	-15	1	-48	TTGCCTGAAC	-13	CGTACCTTGC	-142	1,1	6.81	-13.60	no	31	Rmet_2270	acs	Op0648r_2	MET-CAH-FbP
1401	TSS_2190740-3	217	9	0,1	-15	1	-36	GCAGCCGAAT	-13	CGTACCATTT	-143	4,1	8.15	5.40	m	50	Rmet_5440	0	Op1619r_3	NA
1402	TSS_1130025+2	217	40	0,5	-5	0	-36	TCAGCAACTT	-11	CTCCAACTGC	-143	5,1	2.12	4.40	m	196	Rmet_1040	lolE	Op0299f_1	NA
1403	TSS_1122392-2	217	53	0,1	-16	1	-48	TTACAAAAAC	-14	GCCAAACTTA	-146	4,1	8.10	-14.10	no	36	Rmet_1033	0	Op0296r_1	NA
1404	TSS_3720445+2	216	7	0,1	-17	1	-38	GCTGCCGGGG	-15	GCTAGAATCA	-135	5,1	11.30	4.40	m	41	Rmet_3445	0	Op0959f_2	NA
1405	TSS_895054-2	216	44	0,1	-7	0	-41	TGGAAGGTCA	-5	GGCACCATGG	-140	1,1	6.64	-9.10	no	443	Rmet_0821	0	Op0232r_2	NA
1406	TSS_1948242-2	216	26	0,1	-17	1	-45	GTAACGGCCC	-15	TTTAAAATTC	-140	4,1	9.56	-7.10	no	28	Rmet_1795	0	Op0520r_1	NA
1407	TSS_1758481+2	215	52	0,1	-15	1	-34	TTGTCAGTGG	-13	AATATGATGG	-138	1,1	8.54	12.60	s	118	Rmet_1630	0	Op0457f_1	NA
1408	TSS_2840853+2	215	32	0,1	-16	1	-41	GCCCGCGAGC	-14	GCTACCCTGC	-140	4,1	8.31	-5.60	no	95	Rmet_2611	0	Op0731f_1	NA
1409	TSS_114561+4	215	13	0,1	-16	1	-35	TTGCCGATTT	-14	GTTACTATAG	-141	1,1	9.10	11.60	s	21	Rmet_5946	pbrR	Op1807f_1	NA
1410	TSS_3751851-2	214	44	0,1	-4	0	-23	TGGACGGGCT	-2	AGTATCCTAT	-139	1,1	6.30	-0.90	no	55	Rmet_R0066	0	Op2056r_1	NA
1411	TSS_3770945-2	214	9	0,1	-16	1	-48	GGACAACACC	-14	GTTACCATGT	-143	4,1	9.27	-17.60	no	1211	Rmet_3485	ybdK	Op0970r_4	NA
1412	TSS_211694+2	214	10	0,1	-17	1	-35	TGCAGCATAT	-15	GGCATGATGG	-144	1,1	6.41	7.60	s	257	Rmet_0203	0	Op0059f_3	NA
1413	TSS_2315224+2	214	20	0,1	-17	1	-36	TGGAAAGCCT	-15	GTTACAATGC	-139	1,1	10.41	12.30	s	7516	Rmet_2134	trxA	Op0615f_1	GIP-PTL-Fold
1414	TSS_876380-3	214	53	0,1	-15	1	-48	GGACGAATGC	-13	GCTACGCTCA	-142	4,1	8.73	-19.60	no	62	Rmet_4284	encAB	Op1217r_2	NA
1415	TSS_1087434-3	214	43	0,1	-15	1	-44	GCGGCTCTCC	-13	TGTATAATGA	-143	5,1	10.50	-7.10	no	98	Rmet_4487	0	Op1279r_2	NA
1416	TSS_36162+4	213	7	0,1	-17	1	-35	TGGCAAATCT	-15	CGTATGATCC	-139	1,1	8.80	10.10	s	326	Rmet_6023	0	Op1835f_1	NA
1417	TSS_1103287-2	213	31	0,1	-19	0	-33	TTGTCAGATT	-12	CCTATGTTGT	-142	1,1	6.91	10.60	s	26	Rmet_1012	0	Op0290r_1	MET-XEN-Others
1418	TSS_3799086+2	212	22	0,1	-16	1	-31	CCGGCCCTGC	-7	TGCACGCTTC	-144	5,1	6.99	-0.30	no	34	Rmet_3509	0	Op0973f_1	NA
1419	TSS_2457870-2	212	60	0,1	-16	1	-42	TGCAGCCTAG	-14	GATACCCTTT	-144	1,1	4.98	-3.60	no	65	Rmet_2242	0	Op0642r_1	NA
1420	TSS_1454322+3	212	13	0,1	-17	1	-46	TCCCTGCGCC	-15	CGTATGATCA	-142	1,1	5.20	-9.60	no	212	Rmet_4807	0	Op1398f_2	NA
1421	TSS_3848574+2	212	197	0,1	-17	1	-42	TTGACAGGCA	-15	TCTAGAATTC	-140	1,1	10.57	4.40	(m)-sba	100	Rmet_3549	tctC	Op0985f_1	EIP-TRA-Ion
1422	TSS_1586816+3	212	66	0,1	-16	1	-34	TCCTATTTAG	-14	TATAAACTTT	-146	1,1	5.00	5.60	m	95	Rmet_4925	0	Op1444f_1	MET-AA-Tyr
1423	TSS_1836423-2	212	41	0,1	-15	1	-46	GCCCAGGCGC	-13	GCTATGATCC	-139	4,1	10.44	-14.60	no	121	Rmet_1694	0	Op0480r_1	NA
1424	TSS_3500634-2	211	24	0,1	-14	1	-38	TTGTCATTAC	-12	GTGATAATGC	-138	1,1	9.51	8.40	s	120	Rmet_3224	0	Op0912r_1	NA
1425	TSS_2268180+2	211	57	0,1	-16	1	-49	TCCCCATCTC	-14	GTTACAATGG	-138	1,1	8.82	-14.60	no	20	Rmet_2089	talB	Op0605f_1	MET-CAH-PP_KDPG
1426	TSS_2206169-2	211	33	0,1	-15	1	-34	TTGCGATGGA	-13	GGCATTTTAC	-143	1,1	6.05	9.60	s	26	Rmet_2036	0	Op0594r_1	NA
1427	TSS_1903202+2	210	40	0,1	-17	1	-40	GCCAGAAAGC	-15	GGTAGAATTG	-137	4,1	12.38	1.40	(w)-sba	3630	Rmet_1757	0	Op0501f_1	NA
1428	TSS_744510+3	210	38	0,1	-16	1	-36	TCGACGGGCC	-14	GGTAACATAA	-140	1,1	7.12	11.90	s	37	Rmet_4162	0	Op1170f_4	NA
1429	TSS_897475+3	209	3	0,1	-25	0	-36	TGGCGCATTT	-15	CATATAAAAC	-146	1,1	3.55	9.30	s	314	Rmet_4303	0	Op1226f_1	EIP-TRA-Ion
1430	TSS_19152-5	209	60	0,1	-13	1	-33	CCTGCCGGTC	-11	GCAATAATGG	-143	5,1	9.36	5.20	m	2818	Rmet_6326	0	Op1921r_1	NA
1431	TSS_1932676+3	209	109	0,1	-16	1	-47	TTCTCCTCCG	-14	CGCATCATGC	-145	1,1	5.37	-11.10	no	15	Rmet_5235	0	Op1544f_1	NA
1432	TSS_3388557+2	208	62	0,1	-16	1	-39	TTGATAGTCG	-14	GCGACAATCT	-142	1,1	8.29	7.40	s	14992	Rmet_3139	0	Op0879f_1	NA
1433	TSS_3495784+2	208	17	0,1	-8	0	-35	TTGAAGCGCC	-6	CTAACGATCT	-144	1,1	5.19	4.20	(m)-ba	21	Rmet_3220	0	Op0909f_1	NA
1434	TSS_161799+2	208	34	0,1	-17	1	-39	GCACAAGACC	-15	GGTAAAATCC	-137	4,1	12.21	2.40	w	16	Rmet_0157	0	Op0045f_1	NA
1435	TSS_2033270+2	208	49	0,1	-16	1	-41	TCCCTATGTC	-14	GCTACCCTTG	-142	1,1	5.52	-1.60	no	90	Rmet_1873	0	Op0541f_3	GIP-TL-Ass
1436	TSS_3615915-2	208	12	0,1	-18	1	-43	TCAGACCTAT	-16	GACACACTCG	-145	5,1	7.22	-4.60	no	#NV	#NV	#NV	#NV	#NV
1437	TSS_2416298-2	207	33	0,1	-15	1	-49	TTGCGAGGCA	-13	GGTAGATTCT	-142	1,1	8.39	-14.60	no	141	Rmet_2203	prpE	Op0630r_2	MET-CAH-Prop
1438	TSS_3430682-2	207	42	0,1	-18	1	-43	TCAGACCTAT	-16	GACACACTCG	-145	5,1	7.22	-4.60	no	#NV	#NV	#NV	#NV	#NV
1439	TSS_2233707+3	207	27	0,1	-16	1	-32	TTCAGGGAGC	-11	ATAATAAATC	-145	1,1	6.15	8.10	s	250	Rmet_6735	0	Op1630f_2	NA
1440	TSS_2726856+2	207	35	0,1	-16	1	-45	TCGCAAAAGG	-14	GATACAATTG	-137	1,1	9.92	-6.10	no	208	Rmet_2512	0	Op0695f_3	NA
1441	TSS_332260+2	207	42	0,1	-17	1	-39	GCGGAAAGCG	-15	GCTAATATAC	-138	5,1	10.03	2.90	w	132	Rmet_0314	0	Op0091f_1	NA
1442	TSS_2155078-2	207	26	0,1	-15	1	-43	TTGTCACGGG	-13	GTTACAATCG	-135	1,1	10.53	-0.60	no	26	Rmet_1980	ansB	Op0576r_1	MET-EN-Nitrog
1443	TSS_3304157-2	207	9	0,1	-6	0	-45	GCAAAGGTGC	-12	AATACTATTT	-146	4,1	8.69	-13.60	no	2636	Rmet_3045	int	Op0856r_1	NA
1444	TSS_2236002-3	207	31	0,1	-16	1	-42	TTGCGTGCGC	-14	GCTATCATTG	-141	1,1	9.51	0.40	(w)-sba	207	Rmet_5490	pobA	Op1631r_1	MET-XEN-24D
1445	TSS_1527257+2	206	28	0,1	-16	1	-36	TGGATAAAAA	-14	GTTACATTGA	-137	1,1	7.56	11.90	s	28	Rmet_1414	0	Op0397f_2	NA
1446	TSS_89115-2	206	28	0,1	-16	1	-48	CCGGCCATTC	-14	GGTAGACTGT	-138	5,1	10.04	-16.10	no	1389	Rmet_0080	0	Op0022r_1	MET-CAH-Prop
1447	TSS_1039283-3	206	20	0,1	-16	1	-47	CGGCGGTAAC	-14	GCTACACTGC	-142	1,1	4.46	-15.10	no	1055	Rmet_4441	dadA3	Op1263r_2	MET-EN-Nitrog
1448	TSS_110719+4	206	51	0,1	-16	1	-36	TTGCAAATCA	-14	GGCATCATGG	-140	1,1	8.90	12.40	s	3863	Rmet_5946	pbrR	Op1807f_1	NA
1449	TSS_3840743-2	205	33	0,1	-15	1	-36	TTGTAGTCCA	-13	TAAAACCTGA	-146	1,1	2.80	8.40	s	3803	Rmet_3539	0	Op0980r_1	NA
1450	TSS_478690+3	205	6	0,1	-23	0	-36	TTGAACTGAT	-13	GCTCAAATGC	-144	1,1	6.96	10.40	s	484	Rmet_3923	acrA	Op1098f_2	EIP-TRA-Other
1451	TSS_6240-3	205	28	0,1	-16	1	-40	TTCACTATGC	-14	TTTACATTGC	-144	1,1	7.66	3.90	(m)-sba	146	Rmet_5821	xerD	Op1759r_1	NA
1452	TSS_190356+2	204	21	0,1	-18	1	-38	GCAGCGCCGG	-16	AGTAGAATGT	-137	4,1	8.25	5.40	m	48	Rmet_0184	0	Op0055f_1	NA
1453	TSS_1485184+3	204	20	0,1	-16	1	-37	TGGCATGTAT	-14	CCCACAATGA	-140	1,1	6.95	7.90	s	23	Rmet_4831	0	Op1408f_1	EIP-TRA-Ion
1454	TSS_1370674-3	204	54	0,1	-29	0	-31	TGGCGACACA	-4	CGGACGCTCC	-146	1,1	3.91	-0.30	no	2966	Rmet_4724	cls	Op1363r_1	MET-LIP-GlycPLip
1455	TSS_35835+5	204	35	0,1	-17	1	-36	TTGACACTAG	-15	CATAGATTTC	-145	1,1	7.90	13.80	s	23	Rmet_6187	tnpR	Op1894f_4	NA

1456	TSS_922659-3	204	12	0,1	-15	1	-35	TTGAGTTTCC	-13	GTTACGCTAA	-143	1,1	7.21	11.20	s	35	Rmet_4328		0	Op1231r_1	NA	
1457	TSS_1425773-3	204	4	0,1	-16	1	-35	TTGCAAAAAT	-14	TGAACAATCG	-136	1,1	8.32	11.60	s	42	Rmet_4781		0	Op1391r_1	EIP-TRA-ABC	
1458	TSS_53286+5	204	31	0,4	-12	1	-37	TTCAAGGGGG	-12	CGTATAAGAG	-148	1,1	0.93	6.90	s	23	Rmet_6205	*cnrY*		Op1896f_2	NA	
1459	TSS_2048025-3	204	37	0,1	-16	1	-43	TTGAATGCAA	-14	ATTAAGATAG	-141	1,1	5.80	-3.10	*no*	95	Rmet_5335		0	Op1581r_1	NA	
1460	TSS_1790737+2	203	39	0,1	-16	1	-45	GCATACGTAT	-14	AGCACAATGC	-143	5,1	6.58	-12.60	*no*	56	Rmet_1656	*yqjG*		Op0467f_1	GIP-PTL-Fold	
1461	TSS_1489281+2	203	11	0,1	-16	1	-35	TTCCGTCCCG	-14	CGTAAAGTGC	-135	2,1	10.68	9.10	s	96	Rmet_1380		0	Op0385f_3	NA	
1462	TSS_231954+4	203	60	0,1	-17	1	-41	GCTGACGTGG	-15	GGTACCATCA	-138	5,1	9.05	-3.60	*no*	20	Rmet_6066		0	Op1849f_2	NA	
1463	TSS_940876-2	202	20	0,1	-15	1	-43	TTCATGTGAA	-13	CCTATAATGA	-144	1,1	8.98	-2.10	*no*	20	Rmet_0859	*greB*		Op0246r_1	GIP-TK-Assoc	
1464	TSS_185992+3	202	34	0,1	-21	0	-49	GCCCAAATGG	-28	GAGACATTCC	-147	4,1	6.29	-8.20	*no*	111	Rmet_3651	*pilA*		Op1020f_2	GIP-PTL-T2S	
1465	TSS_1305488-3	202	26	0,1	-2	0	-49	TTGCCGACAC	-14	ACTACTATGT	-141	1,1	8.48	-13.60	*no*	31	Rmet_4672		0	Op1351r_1	NA	
1466	TSS_769675-2	202	38	0,1	-22	0	-44	TTGACCCAGC	-13	TTCATACTGG	-141	1,1	8.43	-3.60	*no*	4	Rmet_0697	*pilA*		Op0196r_2	GIP-PTL-T2S	
1467	TSS_1226074-3	202	43	0,1	-16	1	-41	GCAAGAACGC	-14	CCTAAAGTAT	-140	4,1	9.42	-2.60	*no*	4838	Rmet_4599	*tnpR*		Op1323r_1	NA	
1468	TSS_1372983+3	202	32	0,3	-25	0	-45	CAAATTCCTG	-25	CGCCAAACCA	-149	3,0	4.10	-8.20	*no*	230	Rmet_4729		0	Op1366f_3	NA	
1469	TSS_211592-2	201	35	0,1	-16	1	-43	TCAAAAAAAT	-14	GATATGCTGG	-141	4,1	9.76	-3.60	*no*	3194	Rmet_0199		0	Op0058r_1	NA	
1470	TSS_648966+3	201	45	0,1	-17	1	-39	TTGAATAATG	-15	GCCATCATAG	-144	1,1	7.64	7.40	s	784	Rmet_4080		0	Op1146f_1	NA	
1471	TSS_1621992+2	201	19	0,1	-26	0	-35	TTGATTTTAT	-14	ACTATTATGA	-147	1,1	8.38	12.60	s	919	Rmet_6452		0	Op0423f_1	NA	
1472	TSS_2586051+2	201	47	0,1	-16	1	-40	TCTTAACTTT	-14	ACCATCATTC	-147	5,1	4.65	1.40	*(w)-sba*	120	Rmet_2375	*ccoN*		Op0669f_2	MET-EN-OxPhos	
1473	TSS_3625148+2	201	21	0,1	-16	1	-45	TCGCCTAGCG	-14	GGTAGCGTGT	-145	1,1	5.21	-8.10	*no*	3250	Rmet_3352		0	Op0933f_1	NA	
1474	TSS_3760702-2	201	27	0,1	-16	1	-49	GCGACCAGAC	-14	GTTAACATCG	-139	5,1	8.42	-17.10	*no*	74	Rmet_3478	*rep*		Op0968r_1	GIP-REP-Recomb	
1475	TSS_1672031+3	200	27	0,1	-16	1	-41	GCCCGCACGC	-14	GCTATACTCC	-136	4,1	10.69	-3.60	*no*	1754	Rmet_4994		0	Op1466f_1	NA	
1476	TSS_1267011-3	200	33	0,1	-16	1	-36	CCTGACTTGC	-14	GCTACCATGC	-140	5,1	8.53	5.40	m	2950	Rmet_4633	*tauD*		Op1337r_1	MET-OAA-Taur	
1477	TSS_395348+2	200	123	0,1	-16	1	-49	GGCGGGCCGC	-14	AGTAAGCTAT	-142	4,1	5.53	-21.60	*no*	86	Rmet_0370		0	Op0103f_3	NA	
1478	TSS_3649824+2	200	42	0,1	-17	1	-45	TGGCGGGGAC	-15	TATACAATCA	-140	1,1	8.25	-6.10	*no*	870	Rmet_3375		0	Op0937f_2	NA	
1479	TSS_3195574-2	200	121	0,1	-15	1	-39	TCGAATAGAC	-13	AATACACTTC	-146	1,1	7.44	3.90	m	51	Rmet_6566		0	Op0830r_2	NA	
1480	TSS_140021+2	199	72	0,1	-15	1	-35	TGGCGCGGTT	-13	GAGACCCTTG	-145	1,1	4.31	7.70	s	2586	Rmet_0137		0	Op0037f_1	NA	
1481	TSS_788249-2	199	27	0,1	-17	1	-39	GCCCAAAACC	-15	AATATATTTA	-143	4,1	8.57	2.40	w	162	Rmet_0712	*ompA*		Op0202r_2	EIP-TRA-Pores	
1482	TSS_771541+3	199	17	0,4	-19	0	-36	TTGTGTATCT	-15	GATAAAGTGG	-140	1,1	7.67	10.80	s	6563	Rmet_4193		0	Op1186f_1	NA	
1483	TSS_2023756+2	199	31	0,1	-16	1	-45	CCGGACTTCG	-14	GCTAAAATTC	-138	5,1	10.43	-10.10	*no*	120	Rmet_1866	*ispZ*		Op0539f_1	DIV-Division	
1484	TSS_663686-2	199	22	0,1	-15	1	-34	TTGCCCACTA	-13	GTTACATTCG	-139	1,1	8.81	10.60	s	103	Rmet_0609	*accB*		Op0172r_2	MET-AA-ValLeuIle	
1485	TSS_3513712-2	199	33	0,1	-15	1	-38	TTCACGATAT	-13	GGTACACTTT	-140	1,1	9.44	6.90	s	380	Rmet_3237	*tatA*		Op0918r_3	GIP-PTL-Exp	
1486	TSS_153485+3	199	59	0,1	-17	1	-47	TTGATAGGCT	-15	GTTAAGCTAA	-142	1,1	7.24	-8.60	*no*	143	Rmet_R0074		0	Op1006f_1	NA	
1487	TSS_226612+2	199	35	0,1	-11	1	-38	TGACAACCCC	-9	CTTACGATCA	-143	4,1	6.69	0.40	*(w)-ba*	180	Rmet_0214		0	Op0065f_1	MET-LIP-FAMet	
1488	TSS_1682540+2	199	74	0,1	-16	1	-36	TTGACTTCCG	-14	GTAAAAGTTC	-145	1,1	7.53	13.40	s	733	Rmet_1562		0	Op0431f_4	NA	
1489	TSS_3083004-2	198	54	0,1	-15	1	-36	TTGAGCTGCA	-13	GGCAAAATCG	-140	1,1	9.25	11.40	s	5304	Rmet_2828		0	Op0796r_1	NA	
1490	TSS_767410-3	198	36	0,1	-15	1	-46	TGGCAACACC	-13	GCGATACTGG	-135	1,1	7.38	-10.10	*no*	36	Rmet_4182	*estB*		Op1181r_2	NA	
1491	TSS_204501+3	198	26	0,1	-17	1	-43	TTCCGCAAGG	-15	ACTACCATGC	-141	1,1	7.36	-3.10	*no*	159	Rmet_3666		0	Op1020f_4	NA	
1492	TSS_1373173+3	198	70	0,1	-16	1	-42	TTGTGTAGCC	-14	GATACGCTTG	-144	1,1	7.00	-1.60	*no*	40	Rmet_4729		0	Op1366f_3	NA	
1493	TSS_3730234+2	197	33	0,1	-16	1	-46	TCTGAACGGT	-14	GCTATACTCC	-137	5,1	11.09	-9.60	*no*	52	Rmet_3453		0	Op0961f_3	NA	
1494	TSS_2018267-2	197	26	0,1	-14	1	-33	TTCCCGTTCG	-12	TGGACAATGC	-140	1,1	7.42	8.10	s	12	Rmet_1861		0	Op0538r_1	EIP-TRA-ABC	
1495	TSS_508587+3	197	3	0,1	-18	1	-23	TTGCACTGCC	-3	TCCATAATGA	-144	1,1	9.22	-1.40	*no*	44	Rmet_3951		0	Op1108f_1	NA	
1496	TSS_2073085+3	197	22	0,5	-17	1	-45	GACTCCGGCG	-25	CCGCTTGCGT	-148	3,0	2.49	-9.20	*no*	45	Rmet_5352		0	Op1590f_2	NA	
1497	TSS_3026569-2	197	8	0,1	-15	1	-42	CCAGACCTCC	-13	GCGATAATCG	-139	5,1	8.87	-6.60	*no*	0	Rmet_2778	*cobQ*		Op0782r_1	MET-COF-Porph	
1498	TSS_2929505-2	196	31	0,1	-15	1	-46	TACATATATA	-13	GTTATGCTTG	-148	1,1	3.41	-10.60	*no*	372	Rmet_2697		0	Op0760r_1	NA	
1499	TSS_987621+2	196	22	0,1	-16	1	-41	TGGATCGTAC	-14	GGCATGATCT	-141	1,1	6.60	0.90	*(w)-sba*	87	Rmet_0899		0	Op0261f_2	NA	
1500	TSS_1840989+2	196	33	0,1	-16	1	-43	TCGTCCAATA	-14	CGTAGCCTTG	-141	1,1	4.80	-4.10	*no*	50	Rmet_1699		0	Op0481f_1	NA	
1501	TSS_1487300-3	196	29	0,1	-15	1	-35	TGGCGGCGCC	-13	TCTAAACTGG	-140	1,1	7.80	9.70	s	4457	Rmet_4828		0	Op1407r_1	NA	
1502	TSS_150633+3	196	22	0,1	-19	0	-44	TCAGACCTAT	-17	GACACACTCG	-144	5,1	7.22	-5.60	*no*	#NV	#NV	#NV		#NV	#NV	
1503	TSS_1938955+2	195	44	0,1	-14	1	-32	CCGGCAACGC	-12	GCTAGGCTAC	-138	5,1	8.74	3.10	w	42	Rmet_1788	*poxR*		Op0519f_1	NA	
1504	TSS_3304207-2	195	21	0,1	-15	1	-34	TGGTAAAGCC	-13	GGCATATTGT	-140	1,1	6.44	9.10	s	2686	Rmet_3045	*int*		Op0856r_1	NA	
1505	TSS_57598+4	195	24	0,1	-17	1	-36	TGCAACCTCT	-15	GGCATAATCA	-138	1,1	7.09	9.80	s	6212	Rmet_5994	*gntR*		Op1827f_1	NA	
1506	TSS_566402+3	195	20	0,1	-3	0	-37	TTGTGAACTG	-1	CATACACTGG	-142	1,1	8.01	-2.60	*no*	51	Rmet_3996	*mdoG*		Op1122f_1		0
1507	TSS_2357445+3	195	23	0,1	-17	1	-35	TCAGCCCGAT	-15	ATTAGAATCG	-138	5,1	10.06	8.60	s	149	Rmet_5603		0	Op1670f_1	NA	
1508	TSS_38587+4	195	40	0,1	-17	1	-36	TTGACGTATC	-15	TGTAGCATAA	-143	1,1	8.02	12.80	s	17	Rmet_6020		0	Op1835f_1	NA	
1509	TSS_145984-4	195	44	0,1	-15	1	-45	GCGGACAGTG	-13	CGGATAATTG	-141	5,1	8.96	-11.10	*no*	67	Rmet_6151		0	Op1880r_2	NA	
1510	TSS_475684+2	194	21	0,1	-16	1	-37	TATAGGTGCC	-14	TATACAGTTG	-144	2,1	8.50	7.40	s	8533	Rmet_0456	*rhlE1*		Op0119f_1	NA	
1511	TSS_3338036-2	194	38	0,1	-28	0	-36	TTCTGAAACT	-5	TGGAGCATGC	-147	1,1	4.65	0.90	*(w)-ba*	1894	Rmet_3079		0	Op0864r_1	NA	
1512	TSS_3732810-2	194	33	0,1	-15	1	-36	TCGAACCGTT	-13	GCTACGATGG	-136	1,1	8.43	9.90	s	44	Rmet_6597		0	Op0962r_3	NA	
1513	TSS_475183+3	194	13	0,1	-19	0	-44	TCAGACCTAT	-17	GACACACTCG	-144	5,1	7.22	-5.60	*no*	#NV	#NV	#NV		#NV	#NV	
1514	TSS_1401226+3	194	21	0,1	-16	1	-49	GCCGCCGCCT	-14	GGTAAGATGT	-141	4,1	7.68	-19.60	*no*	2011	Rmet_4756		0	Op1382f_1	NA	
1515	TSS_1791983-3	194	44	0,1	-13	1	-33	CCGTAAGGCG	-11	TGTAGAATGT	-141	5,1	9.34	6.70	s	5	Rmet_5099		0	Op1501r_2	NA	
1516	TSS_3712223+2	193	7	0,1	-16	1	-35	TCGATAGCAT	-14	CCTATAGTAG	-142	1,1	6.34	12.10	s	86	Rmet_3435		0	Op0953f_1	NA	

1517	TSS_207381-2	193	33	0,1	-15	1	-28	TCGACCGCCC	-2	CGCATACTTC	-139	1,1	6.40	0.70	*(w)-nu*	14923	Rmet_0185	*0*	Op0056r_1	NA
1518	TSS_2676389-2	193	4	0,3	-23	0	-48	TCGCCGACAT	-8	CCAACGCTTG	-143	1,1	3.19	-21.10	*no*	176	Rmet_2464	*accD*	Op0688r_4	MET-CAH-Pyr
1519	TSS_2640987-2	193	20	0,1	-15	1	-34	TTGAGTGGGC	-13	GGTATGATGC	-135	1,1	10.40	11.60	s	129	Rmet_2430	*fabH*	Op0680r_2	MET-LIP-FASyn
1520	TSS_952835+2	193	76	0,1	-12	1	-41	TTGTGGGCTC	-10	TTGAACATGG	-143	1,1	5.07	-3.60	*no*	107	Rmet_0872	*0*	Op0253f_1	NA
1521	TSS_624353-2	192	34	0,1	-15	1	-34	CCGGACGGCT	-13	GCTATGCTAT	-140	5,1	9.39	5.10	m	35	Rmet_0582	*0*	Op0164r_1	NA
1522	TSS_448799+3	192	29	0,1	-7	0	-34	TTGAACGAAA	-5	GCGATAGTCG	-142	1,1	7.14	4.20	*(m)-ba*	524	Rmet_3904	*yeaG*	Op1092f_1	NA
1523	TSS_102232-3	192	39	0,1	-15	1	-34	TTGCCATTGT	-13	TCGACAATCG	-138	1,1	9.18	11.60	s	48	Rmet_5901	*0*	Op1789r_1	NA
1524	TSS_1586732-3	191	44	0,1	-15	1	-35	GCAGGAAAAC	-13	GATAAGATTT	-142	4,1	10.85	6.20	m	24	Rmet_4924	*0*	Op1443r_1	NA
1525	TSS_27801-4	191	12	0,1	-16	1	-45	TCCCAAAGAC	-14	GGTATATTCG	-141	1,1	7.46	-7.60	*no*	175	Rmet_6037	*0*	Op1838r_1	NA
1526	TSS_3754755-2	190	20	0,1	-16	1	-48	TTGATGCTTA	-14	CATAGGATCG	-139	1,1	7.06	-11.60	*no*	26	Rmet_3474	*hemN*	Op0966r_1	MET-COF-Porph
1527	TSS_185929-4	190	22	0,1	-15	1	-34	TATGGTCGGC	-13	GAGAAAATAG	-141	2,1	8.36	6.60	s	3960	Rmet_6120	*copL*	Op1868r_1	NA
1528	TSS_1224625+3	190	23	0,1	-16	1	-45	TTGCCATTTAT	-19	AATAGAATAG	-142	1,1	9.04	-0.60	*no*	197	Rmet_4603	*0*	Op1324f_2	NA
1529	TSS_3403486-2	189	54	0,1	-27	0	-35	TTGACTTCCT	-13	ATCACTCTAA	-145	1,1	4.29	11.20	s	221	Rmet_3138	*0*	Op0878r_1	MET-COF-CoQ
1530	TSS_1585775+3	189	32	0,1	-17	1	-40	TGCCGCACAC	-15	GGCAAAATGA	-140	1,1	6.42	1.40	*(w)-sba*	80	Rmet_4923	*0*	Op1442f_1	GIP-PTL-Fold
1531	TSS_2043295-3	189	76	0,1	-16	1	-39	TTCAGAGTGC	-14	CGTACCATTG	-140	1,1	7.83	5.90	m	56	Rmet_5330	*zneB*	Op1577r_1	EIP-TRA-Ion
1532	TSS_2216253-3	189	12	0,1	-16	1	-41	TGCTGAACAG	-14	TGTACGATAG	-142	1,1	5.18	-0.60	*no*	84	Rmet_5470	*0*	Op1625r_2	NA
1533	TSS_3409528+2	189	100	0,1	-16	1	-48	TCGACTGACG	-14	CATATACTTG	-143	1,1	7.45	-11.10	*no*	26	Rmet_3146	*0*	Op0881f_1	NA
1534	TSS_2317116-3	189	31	0,1	-15	1	-45	TGGCCGCATC	-13	GTTATAGTCG	-139	1,1	8.06	-7.10	*no*	1550	Rmet_5562	*0*	Op1651r_1	NA
1535	TSS_1162961-3	188	28	0,1	-15	1	-37	TTGCCTCACA	-13	TGTATGCTGT	-143	1,1	8.06	9.40	s	2164	Rmet_4549	*uvrA2*	Op1305r_1	GIP-REP-Recomb
1536	TSS_3164780+2	188	37	0,1	-16	1	-35	TGGTGACGGC	-14	ACGATAATCG	-139	1,1	6.41	11.10	s	34	Rmet_2910	*0*	Op0823f_2	NA
1537	TSS_2722334-2	188	11	0,2	-2	0	-35	TTGAAAAACC	-6	CACACGCATC	-145	1,1	0.86	2.20	*(w)-ba*	68	Rmet_2506	*htpG*	Op0694r_2	GIP-PTL-Fold
1538	TSS_941854+3	188	28	0,1	-15	1	-38	TTCAAGCGGT	-13	TATATATTTG	-140	1,1	7.73	6.90	s	27	Rmet_4345	*0*	Op1236f_2	NA
1539	TSS_393395+2	188	11	0,1	-23	0	-48	TTGCAGTCTG	-21	GTGACGGTGC	-142	1,1	5.09	-7.60	*no*	58	Rmet_0369	*0*	Op0103f_2	EIP-TRA-ABC
1540	TSS_862906-3	188	11	0,1	-15	1	-41	TCGTAAAAAG	-13	GATAAAATGC	-141	1,1	9.09	0.90	*(w)-sba*	0	Rmet_4271	*0*	Op1213r_1	NA
1541	TSS_2347630-3	188	9	0,1	-15	1	-33	TTGACAGATT	-13	GGAATAATTG	-142	1,1	9.55	11.60	s	31	Rmet_5592	*0*	Op1665r_1	EIP-TRA-Other
1542	TSS_2604168+2	187	27	0,1	-16	1	-38	TTTAGTTTCG	-14	CGCATGATCC	-140	2,1	10.30	6.90	s	22	Rmet_2394	*dsbD*	Op0673f_2	GIP-PTL-Fold
1543	TSS_3495584-2	187	19	0,1	-15	1	-34	TTGTTCCAGT	-13	TTTAGATTCG	-144	1,1	6.51	9.60	s	46	Rmet_3219	*0*	Op0908r_2	NA
1544	TSS_97206-4	187	23	0,1	-17	1	-38	TTGCACCATT	-15	TGTATCCTCT	-139	1,1	8.15	8.40	s	90	Rmet_5964	*tnpA*	Op1814r_1	NA
1545	TSS_882330+2	187	24	0,1	-17	1	-47	TTGCCGGCTG	-15	CGTAGCATTG	-138	1,1	7.92	-8.60	*no*	16	Rmet_0806	*0*	Op0229f_1	NA
1546	TSS_3531370+2	187	31	0,1	-16	1	-36	GGAGGACCCC	-14	TTTATAATCG	-140	4,1	10.26	8.40	s	16320	Rmet_6587	*0*	Op2052f_1	NA
1547	TSS_80422+2	186	26	0,1	-16	1	-45	TGGCGATCAG	-14	GCTACAATCT	-137	1,1	9.78	-6.10	*no*	62	Rmet_0073	*birA*	Op0021f_1	NA
1548	TSS_1336616-2	186	27	0,1	-18	1	-43	TTCCACGCCC	-16	GCAACGATCT	-141	1,1	4.61	-3.10	*no*	91	Rmet_R0019	*0*	Op0352r_1	NA
1549	TSS_856632+3	186	32	0,1	-15	1	-43	TGCCGTTATG	-13	GGTAGACTGC	-141	1,1	6.99	-6.60	*no*	12	Rmet_4265	*0*	Op1210f_1	NA
1550	TSS_406157+2	186	42	0,1	-17	1	-47	GCGGCCGGAG	-15	GCTATGCTTT	-136	5,1	10.46	-13.10	*no*	505	Rmet_0381	*0*	Op0103f_5	NA
1551	TSS_1161097+3	186	15	0,1	-16	1	-45	CCGGCAATGC	-14	GCTATACTGT	-138	5,1	11.75	-8.10	*no*	386	Rmet_4550	*0*	Op1306f_2	NA
1552	TSS_2117771-3	186	29	0,5	-18	1	-40	TCGCAAGGTT	-14	CGCATTGTGG	-145	1,1	4.11	1.90	*(w)-sba*	32	Rmet_5391	*0*	Op1605r_1	NA
1553	TSS_3355526-2	185	64	0,1	-15	1	-48	TGGCAAAGTG	-13	CCTAGACTGG	-142	1,1	7.97	-14.10	*no*	25	Rmet_3096	*0*	Op0872r_1	NA
1554	TSS_2891078+2	185	49	0,1	-16	1	-36	TTGAAACGAT	-14	CATATGCTAC	-141	1,1	7.92	13.40	s	80	Rmet_2655	*elaB*	Op0753f_1	NA
1555	TSS_197125-4	185	14	0,1	-16	1	-36	TTCCGACACC	-14	GTTAATATTT	-143	1,1	7.59	10.90	s	2354	Rmet_6107	*copM*	Op1864r_1	NA
1556	TSS_630321+2	185	26	0,1	-16	1	-34	TTTTGCTGCG	-14	TGTAGTATGG	-139	2,1	11.58	7.10	s	9133	Rmet_0592	*0*	Op0167f_1	NA
1557	TSS_2646939-2	185	13	0,1	-15	1	-39	TTGCCTGCCG	-13	GCAACAATTG	-139	1,1	7.52	5.40	m	87	Rmet_2438	*gph*	Op0682r_1	MET-CAH-DiCarb
1558	TSS_1988610+2	184	23	0,1	-16	1	-36	CCTGCCATCC	-14	TGTATGATGA	-141	5,1	10.12	7.40	s	21	Rmet_1834	*0*	Op0533f_1	NA
1559	TSS_199719+4	184	18	0,1	-17	1	-48	TTCATATCAC	-15	GGTATCGTTG	-140	1,1	6.71	-11.10	*no*	2275	Rmet_6386	*0*	Op1859f_1	NA
1560	TSS_2723996+2	184	17	0,1	-16	1	-49	TGCTCAACTG	-14	ACTAACATCA	-145	1,1	4.71	-15.60	*no*	2	Rmet_2508	*0*	Op0695f_1	NA
1561	TSS_30169+2	183	35	0,1	-16	1	-36	TTCAGGGGCG	-14	GCTAAGATGG	-136	1,1	7.99	10.90	s	2094	Rmet_0028	*0*	Op0009f_1	NA
1562	TSS_3371181+2	183	11	0,1	-17	1	-44	GCCGACAACC	-15	GGTATCGTTG	-141	4,1	7.73	-9.60	*no*	26	Rmet_3111	*coaE*	Op0875f_2	MET-COF-Pant
1563	TSS_998667-3	183	13	0,1	-15	1	-46	GGAAAGCACG	-13	GCTACTGTCG	-141	4,1	5.47	-15.60	*no*	24	Rmet_4402	*0*	Op1253r_1	NA
1564	TSS_2804169+2	183	27	0,1	-17	1	-43	TCGATGCTAG	-15	AGTATATTGG	-142	1,1	6.70	-1.10	*no*	1011	Rmet_2581	*gluQ*	Op0717f_1	NA
1565	TSS_2185341-3	183	8	0,1	-16	1	-46	TGGATCAGGC	-14	GCTAAACTGC	-139	1,1	8.91	-9.10	*no*	26	Rmet_5436	*0*	Op1617r_2	NA
1566	TSS_997196-3	182	34	0,1	-16	1	-35	TAGAGATCGC	-14	GCTAAGGTGT	-145	1,1	3.61	10.10	s	20	Rmet_4400	*0*	Op1251r_1	NA
1567	TSS_983238-2	182	33	0,1	-14	1	-40	TTGCATATTT	-12	TCGACAATCG	-141	1,1	8.29	1.40	*(w)-sba*	91	Rmet_0895	*0*	Op0260r_1	EIP-TRA-ABC
1568	TSS_1514048-2	182	18	0,1	-15	1	-35	GGAACAGCAT	-13	CATATGCTCT	-140	4,1	7.86	8.20	s	63	Rmet_1402	*0*	Op0394r_1	NA
1569	TSS_2378128-2	182	33	0,1	-16	1	-38	TCCTGTGCGC	-14	GGTAGAATGC	-139	1,1	6.79	5.40	m	129	Rmet_R0033	*0*	Op0624r_1	NA
1570	TSS_210671+4	182	100	0,1	-25	0	-35	TTGAAAACGC	-15	GGCAATATCA	-141	1,1	7.70	10.60	s	64	Rmet_6090	*0*	Op1855f_2	NA
1571	TSS_227989-4	182	52	0,1	-15	1	-43	TTGCCCCCGC	-13	GTTAAACTGA	-141	1,1	9.49	-2.60	*no*	12	Rmet_6073	*tnpA*	Op1850r_1	NA
1572	TSS_3781416-2	181	57	0,1	-15	1	-33	TCGCTATCGA	-13	AGTAAACTAT	-141	1,1	6.59	7.10	s	192	Rmet_6598	*0*	Op0970r_3	NA
1573	TSS_1541003+3	181	25	0,1	-15	1	-35	TTGACTTGCT	-13	CCGAAGATGT	-144	1,1	6.63	12.20	s	82	Rmet_4879	*catM*	Op1426f_1	NA
1574	TSS_147645-3	181	11	0,1	-15	1	-38	TTGCCAACAA	-13	GGTACTCTCC	-141	1,1	7.09	5.90	m	565	Rmet_5941	*0*	Op1805r_1	NA
1575	TSS_3869920+2	181	17	0,1	-16	1	-35	ATCCGTTCGG	-14	CCTATGATCC	-138	2,1	9.38	7.10	s	161	Rmet_3568	*0*	Op0989f_1	NA
1576	TSS_2064887-3	181	26	0,1	-15	1	-40	TTCATAAGAC	-13	GTTATACTTT	-143	1,1	9.24	3.90	*(m)-sba*	42	Rmet_5345	*xpsR*	Op1587r_1	NA
1577	TSS_309405-2	180	22	0,1	-4	0	-26	TTGCCTGTTT	-2	GGCAGGATTT	-141	1,1	7.37	0.20	*no*	159	Rmet_0292	*0*	Op0084r_1	NA

1578	TSS_3629004-2	180	38	0,1	-15	1	-36	TATAGTCGCG	-13	TGTAGGCTCT	-143	2,1	9.18	7.40	s	809	Rmet_3351	*0*	Op0932r_1	NA
1579	TSS_160299+2	179	19	0,1	-16	1	-33	AAATTCGGCA	-13	CGAGAATAAC	-143	3,0	8.38	-3.40	no	210	Rmet_0156	*metK*	Op0045f_1	MET-AA-Met
1580	TSS_2270701-2	179	37	0,1	-16	1	-40	TGGCGCGTTT	-14	GGTACAATGC	-134	1,1	10.24	2.90	*(w)-sba*	0	Rmet_2091	*rlmB*	Op0606r_1	GIP-TL-Ass
1581	TSS_119733+5	179	32	0,1	-16	1	-34	TCCCAACGGA	-14	ACTACACTTA	-141	1,1	6.05	6.60	s	692	Rmet_6396	*0*	Op1910f_1	NA
1582	TSS_1516966+2	179	21	0,1	-17	1	-41	TTCACTCTCC	-15	TGTAAATTTA	-145	1,1	6.65	2.90	*(w)-sba*	3	Rmet_1405	*0*	Op0397f_1	NA
1583	TSS_1951497+3	179	44	0,1	-5	0	-34	GCAGCCGCTC	-3	GTTACTCTCC	-146	5,1	7.70	-4.80	*no*	269	Rmet_5251	*0*	Op1550f_1	NA
1584	TSS_1054212+2	179	23	0,1	-16	1	-34	TTCAACAGCG	-14	CCAAGAATCG	-142	1,1	5.35	8.10	s	22	Rmet_0970	*ggt*	Op0271f_2	MET-LIP-Others
1585	TSS_1603535-2	179	29	0,1	-14	1	-43	GTCAAAAACC	-12	TGTAACATGC	-141	4,1	9.60	-7.10	*no*	794	Rmet_1481	*ydcN*	Op0418r_1	NA
1586	TSS_670596+3	179	32	0,1	-17	1	-37	TTGACTTTTT	-15	CATATTATTT	-140	1,1	8.87	13.40	s	642	Rmet_4103	*nemA*	Op1154f_5	NA
1587	TSS_1928976+2	178	18	0,1	-16	1	-38	TGGCGTGAAA	-14	CGTACAATGG	-142	1,1	8.77	6.90	s	2351	Rmet_1779	*0*	Op0517f_1	NA
1588	TSS_94331-5	178	29	0,1	-14	1	-41	GCAGAAAAGC	-12	GATAGAATTT	-137	4,1	12.40	-4.60	*no*	674	Rmet_6245	*0*	Op1903r_2	NA
1589	TSS_501733-3	178	35	0,1	-15	1	-43	TTGCCCCCGC	-13	GTTAAACTGA	-138	1,1	9.49	-2.60	*no*	12	Rmet_3943	*tnpA*	Op2061r_1	NA
1590	TSS_1918439-3	178	54	0,1	-15	1	-38	TTGAGACATC	-13	GAGATACTTA	-142	1,1	7.69	8.40	s	1996	Rmet_6715	*0*	Op2087r_1	NA
1591	TSS_2165590+2	177	32	0,1	-17	1	-35	GCTAACGGTC	-15	AATACAATGA	-142	5,1	7.20	5.60	m	121	Rmet_1994	*0*	Op0583f_2	NA
1592	TSS_603827-2	177	18	0,1	-16	1	-35	TCGTAGCAGT	-14	CATATACTTT	-143	1,1	6.97	10.10	s	80	Rmet_0563	*ptb*	Op0158r_1	MET-CAH-Pyr
1593	TSS_1678544-3	177	46	0,1	-16	1	-35	TTGACTTTGA	-14	ACCAAGATGG	-139	1,1	7.15	11.60	s	84	Rmet_4998	*0*	Op1467r_1	NA
1594	TSS_1320750+2	177	108	0,1	-16	1	-47	GGAGGGGAGT	-14	CGGATAATGA	-141	4,1	8.00	-15.60	*no*	988	Rmet_1202	*0*	Op0349f_1	NA
1595	TSS_289994-3	177	48	0,1	-15	1	-45	TCGGACAGTG	-13	GGCACAATAT	-141	5,1	8.88	-9.10	*no*	4	Rmet_3745	*0*	Op1043r_1	NA
1596	TSS_2420040-2	176	39	0,1	-15	1	-38	TGGCGAGGCT	-13	TCTATAATCG	-138	1,1	9.91	7.90	s	21	Rmet_2206	*gloB*	Op0630r_1	MET-CAH-Pyr
1597	TSS_132671+5	176	30	0,1	-14	1	-32	TTGACCGCCA	-12	GGCAAGCTAC	-142	1,1	5.98	6.60	s	21	Rmet_6401	*0*	Op2118f_1	NA
1598	TSS_1730544+2	176	20	0,1	-16	1	-41	TTGGTAGGTGC	-14	ATTAGAATCG	-141	1,1	7.50	0.90	*(w)-sba*	37	Rmet_1604	*0*	Op0447f_1	NA
1599	TSS_1230856+3	176	30	0,1	-16	1	-37	TTCACCTTGG	-14	GGTAACATGC	-141	1,1	8.95	9.90	s	66	Rmet_4606	*metY*	Op1326f_1	MET-AA-Met
1600	TSS_115528+5	176	51	0,1	-5	0	-28	CCGGAAGGAG	-3	TCTATGATGC	-140	5,1	10.99	-1.30	*no*	730	Rmet_6395	*0*	Op1908f_2	NA
1601	TSS_3618534+2	176	19	0,1	-16	1	-41	AAATGCGTCA	-21	GGCATGTGAC	-145	3,0	7.51	-4.20	*no*	49	Rmet_3342	*0*	Op0931f_3	NA
1602	TSS_648942+3	176	20	0,1	-16	1	-39	TACCGTGTGA	-14	TGAATAATGT	-148	1,1	2.12	3.40	m	808	Rmet_4080	*0*	Op1146f_1	NA
1603	TSS_90044-3	176	9	0,1	-16	1	-41	GTCAAAGCGC	-14	TATACAATCG	-142	4,1	10.88	-0.10	*no*	73	Rmet_5891	*feoA*	Op1785r_2	EIP-TRA-Ion
1604	TSS_1738324-3	176	37	0,1	-15	1	-26	TTGCCCGGCA	-1	GCAATTGTCT	-144	1,1	4.12	-0.80	*no*	80	Rmet_5049	*hutC*	Op1485r_2	NA
1605	TSS_3341110+2	175	16	0,1	-16	1	-40	CTGAGCCGAT	-14	CGTAAAATCC	-143	1,1	5.64	2.40	*(w)-sba*	11518	Rmet_3094	*ffh*	Op0871f_1	GIP-PTL-Exp
1606	TSS_500221-3	175	24	0,1	-15	1	-39	TTGTCGGGAT	-13	GGCACAATCC	-142	1,1	9.01	5.40	m	665	Rmet_3940	*0*	Op1101r_2	NA
1607	TSS_2500749+2	175	6	0,1	-15	1	-35	TGGAACTGCT	-13	GGTCGGATGT	-145	1,1	3.47	8.70	s	63	Rmet_2277	*tonB*	Op0651f_2	NA
1608	TSS_3626633-2	174	34	0,1	-15	1	-43	TTGCCCCCGC	-13	GTTAAACTGA	-142	1,1	9.49	-2.60	*no*	12	Rmet_3349	*tnpA*	Op0932r_1	NA
1609	TSS_83219-3	174	92	0,1	-15	1	-47	TTGTGCTATG	-13	GACACACTGG	-144	1,1	7.02	-12.60	*no*	2535	Rmet_5884	*0*	Op1781r_1	NA
1610	TSS_2471817+2	174	35	0,1	-16	1	-35	TTGCCGACGT	-14	TCTACACTGG	-135	1,1	9.96	11.60	s	570	Rmet_2253	*bcsE*	Op0645f_1	NA
1611	TSS_2422655+2	173	59	0,1	-21	0	-49	TTGACGCAGA	-19	GCGATGTTGA	-144	1,1	5.67	-8.60	*no*	193	Rmet_2211	*ctpF*	Op0633f_1	EIP-TRA-Ion
1612	TSS_2895513-2	173	33	0,1	-25	0	-46	GCGTCTCGGG	-23	TCGATCATGG	-145	5,1	5.05	-3.10	*no*	54	Rmet_2660	*0*	Op0754r_4	NA
1613	TSS_2848836+2	172	16	0,1	-9	0	-27	TGGCCGGATC	-7	GACACGGTTT	-142	1,1	3.99	-0.90	*no*	70	Rmet_2618	*0*	Op0733f_1	NA
1614	TSS_132014-2	172	10	0,1	-15	1	-49	TTCAGAATGA	-13	GCTACAGTCG	-143	1,1	8.08	-15.10	*no*	24	Rmet_0125	*cobW3*	Op0032r_3	NA
1615	TSS_2783014-2	172	26	0,1	-15	1	-35	TTGACATGCC	-13	GTTACGATTC	-141	1,1	10.46	14.20	s	26	Rmet_2559	*tnpR*	Op0708r_2	NA
1616	TSS_192323+2	172	17	0,1	-16	1	-36	CCAGATCGGT	-14	GCTAGAATCT	-140	5,1	9.25	6.40	s	149	Rmet_0186	*glmU*	Op0057f_1	MET-CAH-AminSug
1617	TSS_93039-2	172	43	0,1	-14	1	-33	TTGTCAGCAA	-12	CCTACACTGA	-135	1,1	8.16	10.60	s	26	Rmet_0086	*0*	Op0024r_1	NA
1618	TSS_7136+3	171	13	0,1	-17	1	-30	TCGTGCCGTC	-3	GGCAGCTTTT	-145	1,1	2.36	-2.30	*no*	128	Rmet_5822	*0*	Op1760f_1	NA
1619	TSS_2409304+3	171	16	0,1	-16	1	-49	TTGCATGCGG	-14	ATTACACTTT	-141	1,1	8.28	-14.60	*no*	25	Rmet_5648	*0*	Op1686f_1	NA
1620	TSS_558448+2	170	23	0,1	-18	1	-36	TGCCATGCTG	-16	GAAAAAATAC	-143	1,1	3.96	6.80	s	2184	Rmet_0525	*0*	Op0143f_1	NA
1621	TSS_2768836-2	170	11	0,1	-12	1	-34	TTGCCCTGCA	-10	TGAATGATGG	-143	1,1	6.84	9.20	s	146	Rmet_2542	*0*	Op0702r_1	NA
1622	TSS_3569158-2	170	47	0,1	-17	1	-39	TGGCGCCGTG	-15	GATAAAGTGG	-141	1,1	6.95	4.90	m	789	Rmet_3291	*rpoA*	Op0926r_2	GIP-TK-RNAP
1623	TSS_2132447-2	170	18	0,1	-15	1	-49	TTGAGCAAGG	-13	GCCACAATGG	-137	1,1	9.66	-14.60	*no*	36	Rmet_1958	*0*	Op0574r_2	NA
1624	TSS_128220+3	169	17	0,1	-16	1	-36	CCTGCAAGCT	-14	GGCACAATGC	-135	5,1	11.50	7.40	s	33	Rmet_5927	*mocA*	Op1800f_1	NA
1625	TSS_283160+2	169	14	0,1	-12	1	-30	CCTGAAGCTG	-10	TCTATGGTTC	-142	5,1	7.13	-0.40	*no*	505	Rmet_0267	*0*	Op0077f_1	NA
1626	TSS_969412+2	168	26	0,1	-16	1	-35	TTGTCATCAG	-14	CGCATTGTCG	-144	1,1	4.48	11.60	s	116	Rmet_0885	*0*	Op0257f_1	NA
1627	TSS_2881736+2	168	38	0,1	-15	1	-45	TCGCAATGCC	-13	GTTATATTTA	-140	1,1	8.12	-7.10	*no*	27	Rmet_2644	*0*	Op0745f_3	NA
1628	TSS_477119+3	168	32	0,1	-17	1	-28	AAATTCCTTG	-8	TCCGACCTGC	-143	3,0	8.73	-7.40	*no*	1059	Rmet_R0079	*0*	Op1098f_1	NA
1629	TSS_230255+4	168	18	0,1	-16	1	-48	TTCCAACGCA	-14	TGTATATTTA	-143	1,1	7.51	-12.10	*no*	14	Rmet_6069	*0*	Op1849f_1	NA
1630	TSS_2801746+2	168	50	0,1	-16	1	-43	GCTGCACGAG	-14	GGTAAGGTAA	-140	5,1	7.87	-7.60	*no*	24	Rmet_2577	*0*	Op0715f_1	NA
1631	TSS_674624-3	168	37	0,1	-16	1	-35	TTGACTTGCG	-14	GCTACGGTTG	-141	1,1	7.51	11.60	s	22	Rmet_4104	*acxR*	Op1155r_1	NA
1632	TSS_2337664-3	168	29	0,1	-15	1	-44	GGCGAGATAC	-13	AGTATGCTGG	-143	4,1	7.99	-11.60	*no*	28	Rmet_5584	*0*	Op1663r_2	NA
1633	TSS_3133945-2	168	25	0,1	-15	1	-44	TTGAGCCGCT	-13	GGAACAATAT	-139	1,1	7.42	-4.60	*no*	3914	Rmet_2878	*purN*	Op0808r_1	NA
1634	TSS_1188529+3	168	22	0,4	-15	1	-36	TTGACGCCCT	-15	TGTATAAAAG	-142	1,1	5.35	12.80	s	126	Rmet_4574	*0*	Op1316f_1	MET-CAH-Prop
1635	TSS_2202093-3	168	42	0,1	-15	1	-43	TTGCCCCCGC	-13	GTTAAACTGA	-139	1,1	9.49	-2.60	*no*	12	Rmet_5453	*tnpA*	Op1621r_2	NA
1636	TSS_2438305-3	168	42	0,1	-15	1	-43	TTGCCCCCGC	-13	GTTAAACTGA	-140	1,1	9.49	-2.60	*no*	12	Rmet_5680	*tnpA*	Op1697r_1	NA
1637	TSS_2853985-2	167	41	0,1	-16	1	-38	CCGGCCCCAC	-14	CTTAGAATTG	-135	5,1	10.57	4.90	m	48	Rmet_2622	*0*	Op0734r_2	MET-XEN-Hex
1638	TSS_1482939-3	167	17	0,1	-17	1	-45	TCGCATTATT	-15	CTCATAATCT	-144	1,1	7.19	-6.10	*no*	96	Rmet_4828	*0*	Op1407r_1	NA

1639	TSS_767063+2	167	18	0,1	-17	1	-41	TTGCTCCCGG	-15	AGTAAAATGT	-138	1,1	9.32	3.40	(m)-sba	38	Rmet_0695	serS		Op0195f_3	MET-AA-GlySerThr
1640	TSS_2050948+2	167	44	0,1	-16	1	-44	GCAAAAACGA	-14	TGTAGCCTTC	-141	4,1	8.33	-9.60	no	4512	Rmet_1888	yhiR		Op0545f_1	NA
1641	TSS_2780068-2	167	29	0,1	-16	1	-34	GCTAACGGTC	-14	AATACAATGA	-142	5,1	7.20	4.60	m	143	Rmet_2555		0	Op0708r_4	NA
1642	TSS_2963190+2	167	33	0,1	-17	1	-48	TTCTCAAGAT	-15	GGTACAATCC	-141	1,1	10.12	-10.10	no	8487	Rmet_2736	apaH		Op0771f_1	MET-NUC-Pur
1643	TSS_3166492-2	167	21	0,3	-13	1	-46	TTGCGATTTT	-12	GCCAGATTTG	-141	1,1	7.39	-10.60	no	62	Rmet_2911	purM		Op0824r_1	MET-NUC-Pur
1644	TSS_2187137+2	166	35	0,1	-26	0	-49	GGAGGAATCC	-15	TGCACACTGT	-142	4,1	7.67	-19.60	no	2941	Rmet_2025		0	Op0593f_1	NA
1645	TSS_3843330+2	166	28	0,1	-15	1	-46	GCTGACAGGC	-13	ACTATACTGT	-144	5,1	10.13	-14.60	no	30	Rmet_3545	dinB		Op0983f_3	GIP-REP-DNA_Pol
1646	TSS_2689281-2	166	30	0,1	-16	1	-40	CCGACCCGCT	-14	GGCATAATCG	-138	5,1	9.14	1.90	(w)-sba	152	Rmet_2475	leuC		Op0688r_2	MET-AA-ValLeuIle
1647	TSS_3514353-2	166	17	0,1	-16	1	-35	TCGAGAAGGC	-14	AGTAGGATAC	-138	1,1	6.88	11.10	s	46	Rmet_3239		0	Op0918r_3	NA
1648	TSS_11651+5	166	30	0,1	-16	1	-35	TTGCTAGGCG	-14	GAAATAATCT	-140	1,1	7.54	12.60	s	4879	Rmet_6327		0	Op1922f_1	NA
1649	TSS_2792397-2	166	23	0,1	-15	1	-36	TGACGCACGC	-13	CCTATGCTTG	-143	4,1	7.24	7.40	s	122	Rmet_2566		0	Op0710r_1	NA
1650	TSS_1129687-3	166	21	0,1	-15	1	-39	GCGGCTACAC	-13	ATGACAATGC	-142	5,1	7.74	0.90	w	35	Rmet_4524		0	Op1297r_3	NA
1651	TSS_261247+2	165	31	0,1	-16	1	-38	GCGGATTGGG	-14	GCTACAATTC	-135	5,1	9.67	3.90	m	19	Rmet_0244		0	Op0071f_3	EIP-TRA-Pts
1652	TSS_1598665-3	165	64	0,1	-28	0	-46	TCGTGCCGGA	-26	TGAAGCCTCC	-143	1,1	1.45	-3.70	no	38	Rmet_4933		0	Op1447r_1	EIP-SIG-2Comp
1653	TSS_375416-2	165	38	0,1	-15	1	-36	TGGCACTGCC	-13	ATTATCATTG	-139	1,1	7.84	9.90	s	1606	Rmet_0353	queF		Op0100r_1	NA
1654	TSS_2330391-2	165	42	0,1	-15	1	-33	TTGCAAAACG	-13	TTTATAATCG	-137	1,1	11.19	10.60	s	57	Rmet_R0032		0	Op0616r_2	NA
1655	TSS_357449-3	165	22	0,1	-15	1	-38	GCCCGCGAGC	-13	GATACGATGC	-139	4,1	9.26	0.40	w	26	Rmet_3815		0	Op1061r_1	NA
1656	TSS_1600168+2	164	39	0,1	-16	1	-39	AACCGCTGCG	-14	GATACACTGT	-143	2,1	10.13	-0.60	no	1607	Rmet_1480		0	Op0417f_1	NA
1657	TSS_2890410+2	164	23	0,1	-13	1	-41	TTGAAGTTGC	-11	GTAATGATCA	-141	1,1	6.98	-0.60	no	748	Rmet_2655	elaB		Op0753f_1	NA
1658	TSS_351119-2	164	57	0,1	-15	1	-34	TTGCACGGTT	-13	ACTATATTCG	-139	1,1	8.64	10.60	s	0	Rmet_0333	arsR		Op0094r_2	NA
1659	TSS_59634+5	164	16	0,1	-16	1	-49	GGCCAGGCCC	-14	GGTACAGTTC	-138	4,1	8.30	-20.60	no	763	Rmet_6211	cnrT		Op1896f_2	NA
1660	TSS_1775039-2	163	26	0,1	-15	1	-39	TTGCGTCGCC	-13	CGCAACATGG	-142	2,1	9.36	3.40	m	53	Rmet_1641		0	Op0462r_3	NA
1661	TSS_1356807+3	163	27	0,1	-16	1	-35	TGGCATTGCA	-14	CCTAGAATGA	-141	1,1	8.11	10.10	s	3987	Rmet_4718		0	Op1362f_1	NA
1662	TSS_1463583+3	163	14	0,1	-16	1	-48	TCGAAACCCT	-22	GTTAGGCTGC	-144	1,1	6.76	-5.10	no	782	Rmet_4817	narK		Op1404f_1	NA
1663	TSS_145-3	163	41	0,1	-15	1	-48	GTAAGGACGC	-13	GGTAACGTAT	-143	4,1	7.39	-18.10	no	355	Rmet_6771		0	Op2107r_1	NA
1664	TSS_3567230-2	163	22	0,1	-14	1	-37	TGGTTAAACA	-12	ATTATCATGC	-142	1,1	6.81	7.90	s	501	Rmet_3289	cutA		Op0926r_2	NA
1665	TSS_753293-3	163	4	0,1	-15	1	-34	CCTGCAACGC	-13	GCTATACTCC	-134	5,1	11.47	6.60	s	47	Rmet_4166	hpnN		Op1171r_1	NA
1666	TSS_3681029+2	163	24	0,1	-16	1	-43	GCTGACGGCT	-14	CTTAAGATGA	-137	5,1	9.00	-8.60	no	1312	Rmet_6594		0	Op0945f_2	NA
1667	TSS_1561119+2	162	22	0,1	-16	1	-46	GCTGCCGTGC	-14	CGCATACTTC	-139	5,1	8.67	-13.60	no	392	Rmet_1441	dxr		Op0403f_2	MET-LIP-Ster
1668	TSS_225267+3	162	45	0,1	-8	0	-46	GCGGAAAGCC	-15	CTTACACTCT	-144	5,1	10.01	-11.10	no	159	Rmet_3685	flhD2		Op1026f_3	EIP-SIG-2Comp
1669	TSS_1412099+3	162	26	0,1	-17	1	-31	TTGCAGTACA	-1	CATATCCTGC	-143	1,1	7.86	-0.80	no	73	Rmet_4767	ecnAB		Op1386f_3	NA
1670	TSS_2504635+2	162	17	0,1	-16	1	-38	TTGCCGTGCG	-14	GGTAGACTCA	-139	1,1	9.29	8.40	s	1253	Rmet_2284	yeiE		Op0653f_1	NA
1671	TSS_1526452+2	162	5	0,1	-17	1	-49	TTCCCGCAAC	-15	GGGACAATAG	-142	1,1	7.25	-14.10	no	23	Rmet_1413		0	Op0397f_2	NA
1672	TSS_332241-2	162	36	0,1	-15	1	-44	TGGCGCCGGG	-13	GGTATATTAG	-140	1,1	7.66	-6.10	no	123	Rmet_0313	uvrA1		Op0090r_1	GIP-REP-Recomb
1673	TSS_887708-2	162	26	0,1	-15	1	-38	TTCCATCGTG	-13	CGTATGATTC	-137	1,1	7.73	5.90	m	48	Rmet_0813		0	Op0230r_2	NA
1674	TSS_2875780+2	161	37	0,1	-16	1	-38	GGACAAACGC	-14	CCTACAATAG	-138	4,1	11.42	3.40	m	44	Rmet_2640		0	Op0745f_1	NA
1675	TSS_490572-2	161	23	0,1	-15	1	-33	TTGACAGCCT	-13	GGTATAATTG	-137	1,1	12.35	12.60	s	7	Rmet_R0005		0	Op0122r_2	NA
1676	TSS_2689233-2	161	31	0,1	-15	1	-35	TCCCCGGCCT	-13	AGCAAAATAC	-141	1,1	4.93	9.20	s	104	Rmet_2475	leuC		Op0688r_2	MET-AA-ValLeuIle
1677	TSS_3130032-2	161	70	0,1	-15	1	-37	TTGCAAGTGT	-13	GGTAGAATCG	-140	1,1	11.43	10.40	s	1	Rmet_2878	purN		Op0808r_1	NA
1678	TSS_117263+3	161	49	0,1	-16	1	-37	GCAGAAACGG	-14	TATATACTCG	-143	4,1	10.49	5.40	m	36	Rmet_5916		0	Op1796f_1	NA
1679	TSS_370288+3	161	7	0,1	-17	1	-49	GCCGGCGCAC	-15	GCTAAACTTC	-137	4,1	9.77	-19.60	no	786	Rmet_3829		0	Op1064f_2	NA
1680	TSS_108770+2	161	59	0,1	-17	1	-46	TTCATGGGGC	-15	GCTAGACTTG	-142	1,1	7.97	-8.10	no	90	Rmet_0102		0	Op0031f_1	NA
1681	TSS_561056+2	161	32	0,1	-7	0	-30	CCTGACCTCC	-5	CGGACAATCG	-140	5,1	7.91	-2.80	no	103	Rmet_0526	amiC		Op0143f_2	MET-PepGlyc
1682	TSS_1942972+2	161	30	0,1	-16	1	-34	CCTGCCGGCC	-14	GCTATACTGG	-136	5,1	11.99	4.60	m	38	Rmet_1791		0	Op0519f_2	NA
1683	TSS_2315085+3	161	36	0,1	-8	0	-20	AAATGTGGTA	0	GTGACACGGG	-146	3,0	5.84	-14.40	no	615	Rmet_5563		0	Op1652f_1	NA
1684	TSS_1549137-3	161	24	0,1	-16	1	-39	GCGTACACGT	-14	GGTAAAATCC	-142	5,1	9.66	1.90	w	53	Rmet_4886		0	Op1429r_2	NA
1685	TSS_3606681+2	160	45	0,1	-17	1	-36	TTGCGAATGC	-15	GTGAAGCTGT	-142	1,1	6.43	9.80	s	11335	Rmet_6590		0	Op0931f_1	NA
1686	TSS_3215661-2	160	51	0,1	-14	1	-35	GCAGCACAGC	-12	GATAAAATCG	-137	4,1	10.93	7.20	s	151	Rmet_2956	purB		Op0832r_1	MET-NUC-Pur
1687	TSS_423063-3	160	42	0,1	-14	1	-34	TCACGGCCCG	-12	CGTATGATTA	-143	4,1	5.87	8.20	s	27	Rmet_3877		0	Op1081r_2	NA
1688	TSS_323364+2	160	41	0,1	-16	1	-36	TGCTCGATCC	-14	GCTATCATGG	-140	1,1	7.02	10.40	s	98	Rmet_0308		0	Op0087f_1	EIP-TRA-Ion
1689	TSS_3307532+2	160	27	0,1	-16	1	-37	GCGGCAATTT	-16	GTGATATTGT	-142	5,1	7.95	7.30	s	69	Rmet_3051		0	Op0859f_1	NA
1690	TSS_153628+3	160	29	0,1	-10	1	-43	AGATTCGCCG	-23	GGGTCAGCCC	-141	3,0	7.06	-8.20	no	0	Rmet_R0074		0	Op1006f_1	NA
1691	TSS_100168+5	160	39	0,1	-16	1	-35	TTGACGACCT	-9	GATATTGTTG	-141	1,1	7.61	9.20	(s)-ba	1417	Rmet_6254		0	Op1904f_4	NA
1692	TSS_1684200+2	160	30	0,1	-17	1	-39	CCTGCTCGCC	-15	GCTATGCTAG	-141	5,1	8.21	1.40	w	282	Rmet_1563	tnpA		Op0431f_4	GIP-REP-Recomb
1693	TSS_88410-4	160	35	0,1	-15	1	-43	TTGCCCCCGC	-13	GTTAAACTGA	-140	1,1	9.49	-2.60	no	12	Rmet_5972	tnpA		Op1818r_1	NA
1694	TSS_831657+3	159	19	0,1	-16	1	-45	TCGGAAATGC	-14	TGCATAATAG	-140	5,1	9.19	-6.10	no	27	Rmet_4244		0	Op1202f_3	NA
1695	TSS_2697507+2	159	35	0,1	-16	1	-30	TTGTCCGGAT	-6	TCGAAAATAC	-140	1,1	7.13	5.20	(m)-nu	4233	Rmet_2489	mdh		Op0689f_1	MET-CAH-TCA
1696	TSS_3572035-2	159	33	0,1	-16	1	-40	GCGGCCAGAT	-14	AGTAGCATTC	-138	5,1	10.01	-0.10	no	28	Rmet_3297	secY		Op0926r_2	GIP-PTL-Exp
1697	TSS_3627674-2	159	26	0,1	-15	1	-37	TTGTTCCTGG	-13	GCTAAGATTC	-140	1,1	8.17	8.40	s	496	Rmet_3350		0	Op0932r_1	NA
1698	TSS_1063787+3	159	30	0,1	-17	1	-49	TCGACTTTCC	-15	CGCATAATAG	-141	1,1	7.18	-12.10	no	8	Rmet_4467	ubiG		Op1274f_2	NA
1699	TSS_1289569+2	158	20	0,1	-17	1	-37	GCCACCGCAG	-15	GTTACGATTC	-137	4,1	8.26	6.40	s	16	Rmet_1179		0	Op0341f_2	NA

1700	TSS_1691615+2	158	49	0,1	-16	1	-39	TTCTCAGCAA	-14	GCTACAATCA	-139	1,1	8.67	6.90	s	77	Rmet_1571	0	Op0433f_2	NA
1701	TSS_3118536-2	158	22	0,1	-24	0	-41	TTCCCTATTT	-20	CATATTCTGC	-143	1,1	6.55	5.30	m	351	Rmet_2867	livK2	Op0806r_1	EIP-TRA-ABC
1702	TSS_3428746-2	158	33	0,1	-16	1	-27	AAATTCCTTG	-7	TCCGACCTGC	-143	3,0	8.73	-8.40	no	1060	Rmet_R0050	0	Op0886r_1	NA
1703	TSS_674460+2	158	39	0,1	-18	1	-37	TTGAAAACGA	-16	CCTATAATTA	-137	1,1	10.97	13.80	s	108	Rmet_0615	groS	Op0175f_1	GIP-PTL-Fold
1704	TSS_2902760-2	158	31	0,1	-15	1	-35	TTGTAGAAGA	-13	AGTATTCTAT	-143	1,1	6.49	11.20	s	634	Rmet_2667	0	Op0756r_5	NA
1705	TSS_1867691+3	158	22	0,1	-16	1	-45	TTCTACCACT	-14	AGTACACTTG	-139	1,1	7.00	-8.10	no	24	Rmet_5169	0	Op1520f_3	NA
1706	TSS_1869709+3	158	25	0,1	-2	0	-35	TTGACTACGG	0	AGCAGGATCT	-145	1,1	6.18	-0.80	no	318	Rmet_6712	0	Op1522f_1	NA
1707	TSS_1421272-2	158	38	0,1	-15	1	-33	TCATCCCGCT	-13	TATACACTGC	-142	5,1	7.84	5.60	m	290	Rmet_1305	patR	Op0376r_6	NA
1708	TSS_2207519-2	158	42	0,1	-15	1	-33	TTCATTGTTC	-13	GTGATGCTGA	-142	1,1	4.25	6.10	m	3	Rmet_2039	0	Op0596r_2	NA
1709	TSS_955843-2	157	24	0,1	-16	1	-35	TTCACAAAAT	-14	GATATTATTG	-140	1,1	9.26	13.10	s	81	Rmet_R0011	0	Op0254r_3	NA
1710	TSS_2294752-3	157	6	0,1	-16	1	-35	TCAGCCGGCG	-14	GTTATGCTTG	-140	5,1	9.51	8.60	s	58	Rmet_5547	0	Op1647r_2	NA
1711	TSS_41281-3	157	18	0,1	-15	1	-44	TCGAAATGGG	-13	TCCATAATCT	-139	1,1	8.22	-4.10	no	26	Rmet_5847	0	Op1767r_1	NA
1712	TSS_1307768-2	157	36	0,1	-16	1	-34	TTCTGATTCT	-14	TTTATAATCC	-139	1,1	9.22	10.10	s	72	Rmet_1192	folD	Op0344r_1	MET-CAH-DiCarb
1713	TSS_102355+2	156	23	0,1	-15	1	-41	CCATCCGCAT	-13	GATAAAATTC	-141	5,1	9.32	-3.60	no	89	Rmet_0096	argS	Op0029f_1	MET-AA-ArgPro
1714	TSS_1458754+3	156	13	0,1	-14	1	-46	CCTGCCCGGC	-12	ACTATAGTGT	-139	5,1	9.47	-14.60	no	1471	Rmet_4811	0	Op1400f_1	NA
1715	TSS_538368-3	156	34	0,1	-15	1	-34	TGGCAAGTGT	-13	TGGAAAATGC	-142	1,1	7.96	9.10	s	1840	Rmet_3972	0	Op1113r_2	NA
1716	TSS_2062244-3	156	24	0,1	-15	1	-49	GTCAACAAGC	-13	GCTATCATCG	-142	4,1	10.25	-18.10	no	9280	Rmet_6719	0	Op1583r_2	NA
1717	TSS_775497-2	156	9	0,1	-16	1	-38	TTGCCGGAAC	-14	CCGACTCTCA	-140	1,1	4.52	6.40	s	92	Rmet_0702	0	Op0198r_1	NA
1718	TSS_302578+2	156	30	0,2	-17	1	-39	TTCAGGAGAA	-15	AATAGAATAG	-140	1,1	7.49	6.90	s	55	Rmet_0286	fdx	Op0083f_3	NA
1719	TSS_2175856+3	156	50	0,1	-17	1	-37	GCACGCGCCC	-15	TGTAGACTAG	-140	4,1	8.07	4.40	m	336	Rmet_5430	0	Op1616f_1	MET-GLYC-LPS
1720	TSS_447447-3	156	27	0,1	-21	0	-43	CCTGCCCGTT	-14	GATAAATTGG	-142	5,1	9.35	-7.60	no	52	Rmet_3901	0	Op1089r_1	NA
1721	TSS_59758+4	156	22	0,1	-24	0	-48	TTGATATCGA	-22	AAGATAATTG	-139	1,1	8.04	-1.60	no	4052	Rmet_5994	gntR	Op1827f_1	NA
1722	TSS_1570574+3	155	46	0,1	-17	1	-36	TTGCCTATCT	-15	GATAATATTT	-143	1,1	8.92	11.80	s	223	Rmet_4908	mmsQ	Op1438f_1	NA
1723	TSS_1985645+2	155	34	0,1	-16	1	-40	GCAGCGCGGCC	-14	CATATAATAC	-135	5,1	10.91	0.40	(w)-sba	88	Rmet_1830	gcvT	Op0531f_1	NA
1724	TSS_1215587+3	155	52	0,1	-18	1	-39	AATGTTTGCC	-16	CCTATAATCG	-140	2,1	10.35	3.40	m	474	Rmet_4596	czcC2	Op1322f_1	NA
1725	TSS_2315402-2	155	67	0,1	-15	1	-33	TGCCCGATTC	-13	CGCACAATAG	-140	1,1	5.81	5.60	m	342	Rmet_2130	nrdJ	Op0614r_1	MET-NUC-Pur
1726	TSS_2577319-3	154	23	0,3	-23	0	-34	TGGACAAAAG	-11	TGAACTATAG	-145	1,1	4.64	9.70	s	270	Rmet_5814	0	Op1755r_1	NA
1727	TSS_1740532-2	154	35	0,1	-15	1	-43	TTGCCCCCGC	-13	GTTAAACTGA	-138	1,1	9.49	-2.60	no	12	Rmet_1613	tnpA	Op0450r_1	NA
1728	TSS_228227+4	154	17	0,1	-17	1	-45	TCGCCAGAAG	-10	ACTATCCTCC	-145	1,1	6.58	-10.10	no	739	Rmet_6071	0	Op1849f_1	NA
1729	TSS_1332226-2	154	22	0,1	-1	0	-44	GCCAGCGCCC	-14	GGCATTCTGT	-141	4,1	6.31	-10.60	no	21	Rmet_1212	tas	Op0352r_1	NA
1730	TSS_3427687-2	154	23	0,1	-9	0	-42	AGATTCGCCG	-22	GGGTCAGCCC	-142	3,0	7.06	-7.20	no	1	Rmet_R0050	0	Op0886r_1	NA
1731	TSS_94038+3	154	41	0,1	-17	1	-49	TGGCGGAACA	-15	GGTATTCTTC	-140	1,1	7.46	-15.10	no	236	Rmet_5895	0	Op1786f_3	NA
1732	TSS_583610+3	154	6	0,2	-17	1	-48	GGAAAAACTC	-15	AATACAGTCG	-149	4,1	6.91	-15.60	no	34	Rmet_4013	pcaH	Op1126f_1	MET-XEN-24D
1733	TSS_862291+2	153	13	0,1	-16	1	-47	GCCGCGCCAT	-14	GCTATAATCA	-141	4,1	9.62	-13.60	no	105	Rmet_0786	yjgR	Op0223f_1	NA
1734	TSS_1925035+3	153	100	0,1	-28	0	-36	TTGACGTCAC	-15	TTTACGTTAC	-142	1,1	6.65	11.80	s	3490	Rmet_5231	0	Op1544f_1	NA
1735	TSS_2350113+3	153	30	0,1	-17	1	-47	GCTGCCGTAC	-15	GATAGGCTGG	-140	5,1	8.49	-15.60	no	247	Rmet_5596	0	Op1666f_2	NA
1736	TSS_3655373-2	153	27	0,4	-28	0	-29	TTGCGCAAAA	-7	AACATATTGC	-145	1,1	6.59	5.20	(m)-nu	15	Rmet_3379	0	Op0940r_3	NA
1737	TSS_478178+3	153	25	0,1	-10	1	-43	AGATTCGCCG	-23	GGGTCAGCCC	-141	3,0	7.06	-8.20	no	0	Rmet_R0079	0	Op1098f_1	NA
1738	TSS_2400491-3	153	36	0,1	-16	1	-35	TTGTTCTTTT	-14	ATTACCATCT	-143	1,1	7.04	10.60	s	89	Rmet_5640	0	Op1681r_1	NA
1739	TSS_326357+2	153	33	0,1	-17	1	-41	TCGATGTCCG	-15	CGTAAAGTTG	-139	1,1	5.88	1.90	(w)-sba	311	Rmet_0311	apt	Op0089f_1	MET-NUC-Pur
1740	TSS_1888212+2	153	25	0,1	-17	1	-46	CCGACCCGAT	-15	GATATGATTG	-140	5,1	9.04	-9.10	no	219	Rmet_1741	uspA6	Op0495f_1	NA
1741	TSS_3653983-2	153	16	0,1	-15	1	-40	TCGCGGTGGA	-13	GTCACGATGC	-144	1,1	6.40	-0.10	no	170	Rmet_3377	0	Op0938r_1	NA
1742	TSS_2373866+3	153	15	0,1	-15	1	-44	TTGAGGCCAG	-13	GATACAATGC	-138	1,1	10.59	-3.60	no	23	Rmet_5617	0	Op1676f_1	MET-LIP-Ster
1743	TSS_2453445-2	152	19	0,1	-14	1	-37	TCGTGCTCGA	-12	GATAATATTG	-140	1,1	6.25	5.90	m	156	Rmet_2237	0	Op0640r_1	NA
1744	TSS_2268617+3	152	4	0,1	-16	1	-36	CCTGCCTTCG	-14	GTCATACTCT	-144	5,1	7.82	6.40	s	2961	Rmet_5525	0	Op1642f_1	NA
1745	TSS_189026+2	152	20	0,1	-16	1	-41	GCGTCCAGCG	-14	GGTAGACTAG	-139	5,1	8.65	-2.10	no	42	Rmet_0182	0	Op0055f_1	NA
1746	TSS_1278231+2	152	14	0,1	-16	1	-45	TGGAACTACT	-14	GATATACTCT	-141	1,1	9.17	-6.10	no	3	Rmet_R0016	0	Op0333f_3	NA
1747	TSS_418436-2	152	22	0,1	-16	1	-35	TCGTGGAATA	-14	GTTAGAATTC	-135	1,1	8.11	10.10	s	1	Rmet_0390	yciA	Op0106r_1	NA
1748	TSS_969298-2	152	56	0,1	-14	1	-34	TTGACAATGG	-12	CAAATAATTG	-145	1,1	8.21	14.20	s	113	Rmet_0884	0	Op0256r_1	NA
1749	TSS_1263011-2	152	25	0,1	-15	1	-37	CCGGCCACAC	-13	GGTACAATCA	-136	5,1	11.93	5.90	m	79	Rmet_1152	0	Op0330r_1	NA
1750	TSS_768182+3	152	6	0,1	-16	1	-47	GCAACATCGG	-14	ATGACAATGG	-145	5,1	4.82	-13.60	no	187	Rmet_4185	cheD	Op1182f_2	DIV-MOT-Chemotax
1751	TSS_131208-4	152	33	0,1	-11	1	-32	TGGCCGGCGC	-9	TGAAATCTCC	-143	1,1	3.18	4.70	(m)-ba	1274	Rmet_6172	merT	Op1890r_1	EIP-TRA-Other
1752	TSS_3613979-2	151	27	0,1	-16	1	-27	AAATTCCTTG	-7	TCCGACCTGC	-143	3,0	8.73	-8.40	no	1060	Rmet_R0060	0	Op0930r_1	NA
1753	TSS_1068882+2	151	28	0,1	-16	1	-41	TTGCCGATGC	-14	AATAAAATCC	-141	1,1	10.21	3.40	(m)-sba	33	Rmet_0983	0	Op0277f_1	NA
1754	TSS_492521-2	151	85	0,1	-16	1	-38	GTCAGAACTC	-14	CCCACAATCC	-145	4,1	6.58	4.90	m	90	Rmet_0462	0	Op0122r_2	NA
1755	TSS_1264318+3	151	11	0,1	-13	1	-47	TTGATTTTTT	-25	TTGACGCTTG	-144	1,1	5.13	-0.60	no	62	Rmet_4634	0	Op1338f_1	DIV-Apoptosis
1756	TSS_1498445-2	151	20	0,1	-15	1	-34	TTGATCTTTT	-13	ACTAATCTTG	-144	1,1	7.01	9.60	s	1154	Rmet_1387	uspA3	Op0386r_1	NA
1757	TSS_152569+3	151	34	0,1	-17	1	-28	AAATTCCTTG	-8	TCCGACCTGC	-143	3,0	8.73	-7.40	no	1059	Rmet_R0074	0	Op1006f_1	NA
1758	TSS_57939-4	151	29	0,1	-16	1	-35	TTGCCCGGGA	-14	GGTACAGTAA	-140	1,1	8.28	11.60	s	129	Rmet_6000	0	Op1828r_3	NA
1759	TSS_3134000+2	150	23	0,1	-16	1	-35	TTGCGCGTGT	-14	CTCAACCTAC	-141	1,1	4.90	8.60	s	1680	Rmet_2883	0	Op0811f_1	GIP-REP-Recomb
1760	TSS_2117235+2	150	18	0,1	-15	1	-42	TGCCCAGGCC	-13	TGGATAATGG	-139	1,1	6.86	-1.60	no	247	Rmet_1947	ompR	Op0571f_1	EIP-SIG-2Comp

1761	TSS_3612920-2	150	28	0,1	-9	0	-42	AGATTCGCCG	-22	GGGTCAGCCC	-142	3,0	7.06	-7.20	no	1	Rmet_R0060		0	Op0930r_1	NA	
1762	TSS_2082415-3	150	55	0,1	-14	1	-38	TTCCCCGCCG	-12	TGCATACTGC	-141	1,1	6.75	4.90	m	36	Rmet_5360		0	Op1593r_1	NA	
1763	TSS_185567+2	150	16	0,1	-17	1	-36	TGGAACGCGC	-15	GATAGAATGA	-137	1,1	8.90	11.30	s	72	Rmet_0178	gst		Op0053f_2	MET-OAA-GSH	
1764	TSS_567958+2	149	34	0,1	-16	1	-38	TGCTTAAGAC	-14	CCGATAATGA	-144	1,1	4.91	6.40	s	3909	Rmet_6422		0	Op0149f_1	NA	
1765	TSS_947364+2	149	26	0,1	-16	1	-46	GCCCGCAACT	-14	GCTAAAATCC	-137	4,1	10.03	-13.60	no	2273	Rmet_0869		0	Op0251f_1	NA	
1766	TSS_52146-5	149	61	0,1	-15	1	-34	TTGAAGTAGC	-13	GCTAAGCTGT	-142	1,1	8.96	9.60	s	0	Rmet_6203	chrB1		Op1895r_1	NA	
1767	TSS_2326067+2	149	25	0,1	-16	1	-36	TTCATGCGCC	-14	CTTACCATCT	-142	1,1	6.24	10.90	s	1859	Rmet_2139	mdtK		Op0615f_2	EIP-TRA-Ion	
1768	TSS_1178900-2	149	44	0,5	-4	0	-34	TTGATTTTTG	-13	CAGAAATTAA	-147	1,1	3.84	9.60	s	2394	Rmet_1084		0	Op0312r_1	NA	
1769	TSS_1946+2	148	20	0,1	-15	1	-45	TCGACGATTT	-13	GGCATACTAC	-137	1,1	7.47	-7.10	no	273	Rmet_0002	dnaN		Op0001f_2	GIP-REP-DNA_Pol	
1770	TSS_800096+2	148	47	0,1	-23	0	-45	TGGACCAACA	-21	TCGAAGGTTG	-144	1,1	3.36	-1.10	no	737	Rmet_0723	ihfB		Op0205f_2	GIP-REP-Recomb	
1771	TSS_933420-2	148	22	0,1	-15	1	-36	GCAGGCACGC	-13	GTTAGAATCG	-135	4,1	11.26	5.40	m	28	Rmet_0852	rph		Op0244r_2	GIP-TL-Ass	
1772	TSS_2384776-2	148	23	0,1	-16	1	-46	TCCAGCGCGG	-14	GTCATACTGC	-142	1,1	5.56	-10.60	no	425	Rmet_2177	ppx		Op0626r_1	MET-NUC-Pur	
1773	TSS_538313+3	148	29	0,1	-17	1	-36	TGGTAGCCTT	-15	GGTACTGTAA	-142	1,1	4.03	8.30	s	136	Rmet_3974		0	Op1114f_1	NA	
1774	TSS_184844-4	148	32	0,1	-16	1	-37	TGGCAGCGGC	-14	ATTAAAATAT	-141	1,1	8.09	8.90	s	2875	Rmet_6120	copL		Op1868r_1	NA	
1775	TSS_927953+2	147	26	0,3	-3	0	-28	TTACAGGCTC	-3	CGCAAGTTGC	-149	4,1	1.50	-2.30	no	2206	Rmet_0849		0	Op0243f_1	NA	
1776	TSS_2300700+2	147	35	0,1	-17	1	-43	TCGAACAGAA	-15	GCTACACTTG	-138	1,1	8.80	-2.10	no	14524	Rmet_R0026	ffs		Op2026f_1	NA	
1777	TSS_177780+3	147	68	0,1	-17	1	-39	TGACACCTGC	-15	ACTAGACTCC	-142	4,1	6.52	3.40	m	35	Rmet_3644		0	Op1016f_1	NA	
1778	TSS_634110-2	147	24	0,4	-16	1	-35	TGGCGGCTGT	-13	GGTAAAACGT	-144	1,1	1.74	8.70	s	143	Rmet_0589	gltS2		Op0166r_2	NA	
1779	TSS_140522+4	147	5	0,1	-16	1	-39	CTGTACTCTT	-14	GCTAATATTA	-145	1,1	3.48	2.40	w	1429	Rmet_6378		0	Op2114f_1	NA	
1780	TSS_1028600+2	147	5	0,1	-15	1	-43	TCGCAGCATC	-13	GCTAAGCTAC	-140	1,1	6.26	-6.10	no	178	Rmet_0943		0	Op0267f_3	NA	
1781	TSS_811456+3	147	26	0,1	-16	1	-48	GCTGACAGGC	-14	TGTACCATCG	-142	5,1	9.46	-18.60	no	5897	Rmet_4232	bug		Op1200f_1	NA	
1782	TSS_140474-4	147	19	0,1	-16	1	-38	ATTAGCCGCG	-14	AGTACAGTTG	-144	2,1	8.58	3.90	m	529	Rmet_6159		0	Op1882r_2	NA	
1783	TSS_198514+3	146	5	0,1	-15	1	-33	TACCGTTTGG	-13	GTTATGATGC	-136	2,1	11.70	5.60	m	50	Rmet_3663	sbcD		Op1020f_3	GIP-REP-Recomb	
1784	TSS_1243068+3	146	21	0,1	-17	1	-43	TCGTCATGTC	-15	TGTACGCTTG	-141	1,1	6.32	-2.10	no	6345	Rmet_4622		0	Op1332f_1	NA	
1785	TSS_914073+2	146	49	0,4	-5	0	-25	TTTCGGCGCC	0	ATAACTCTGG	-145	2,1	7.17	-5.30	no	21	Rmet_0836		0	Op0239f_1	NA	
1786	TSS_2858403+2	146	17	0,1	-16	1	-36	TTGCGCAGCA	-14	CGTAGATTGC	-140	1,1	7.83	11.40	s	335	Rmet_2625		0	Op0735f_1	NA	
1787	TSS_2627405+2	146	11	0,1	-17	1	-47	TGGAAACCAG	-15	GGTATCATCG	-138	1,1	8.93	-8.10	no	4141	Rmet_6524		0	Op2034f_1	NA	
1788	TSS_3244319-2	146	40	0,1	-15	1	-47	TGGCAAAGGG	-13	ACGACAATGT	-138	1,1	7.24	-12.10	no	3509	Rmet_2980	cbbT2		Op0840r_1	MET-CAH-PP_KDPG	
1789	TSS_146561+2	145	36	0,1	-16	1	-38	TCGGCCCGGC	-14	AGTACAATCG	-139	5,1	11.25	7.90	s	33	Rmet_0142	ttk		Op0039f_1	NA	
1790	TSS_1888292-2	145	19	0,1	-15	1	-40	TTCTTACAGC	-13	TCTAACTTTG	-145	1,1	4.39	0.90	(w)-sba	321	Rmet_1740		0	Op0494r_1	NA	
1791	TSS_236377+3	145	22	0,4	-17	1	-35	TTGCGCCAGC	-14	GCTTCAATGG	-136	1,1	4.92	9.60	s	25	Rmet_3697		0	Op1026f_3	NA	
1792	TSS_600747+2	145	24	0,1	-17	1	-43	TGGCACCTGT	-15	GAAATAATTG	-141	1,1	7.30	-2.10	no	3306	Rmet_0564		0	Op0159f_1	NA	
1793	TSS_2121291+2	145	23	0,1	-17	1	-36	CCGTCAGGCG	-15	GGCATACTCC	-139	5,1	8.63	8.30	s	180	Rmet_1950	ahpC		Op0571f_2	GIP-PTL-Fold	
1794	TSS_868818-2	145	24	0,1	-12	1	-47	GGAGAAAACC	-10	CCCAACCTAC	-142	4,1	5.91	-21.60	no	41	Rmet_0793		0	Op0226r_2	NA	
1795	TSS_2348608+3	145	45	0,1	-17	1	-36	TTGCTTATCT	-15	GATAATATTT	-144	1,1	8.23	10.80	s	223	Rmet_5594		0	Op1666f_1	NA	
1796	TSS_785750-2	145	9	0,2	-29	0	-46	CACCTCGTTA	-26	ATGCAATAAC	-148	3,0	2.68	-11.20	no	734	Rmet_0708		0	Op0200r_1	NA	
1797	TSS_1365540-2	145	13	0,1	-14	1	-47	GCGGAAGCTG	-12	GGCATAGTGG	-141	5,1	8.03	-16.10	no	10017	Rmet_1232	yhgF		Op0356r_1	NA	
1798	TSS_3007535-2	145	14	0,1	-14	1	-46	GGACAAACAC	-12	CCTAGAATAG	-136	4,1	10.95	-14.60	no	72	Rmet_2764		0	Op0776r_1	NA	
1799	TSS_153882+3	145	30	0,1	-18	1	-37	TTGCCACGGC	-16	CGTATGATCT	-136	1,1	9.70	12.80	s	1567	Rmet_3620	degP		Op1008f_1	NA	
1800	TSS_640976+2	144	21	0,1	-16	1	-46	TGAGCGGCGC	-14	ACTATGCTCC	-141	4,1	6.36	-10.60	no	87	Rmet_0594	iorA		Op0167f_2	NA	
1801	TSS_891526+2	144	15	0,1	-17	1	-44	TCGTCTCACG	-15	TGCAGAATCC	-141	1,1	5.48	-4.10	no	88	Rmet_0819		0	Op0231f_1	NA	
1802	TSS_1257866+2	144	25	0,1	-17	1	-43	TTCCCCCTTT	-15	CTTAGAATCT	-140	1,1	8.20	-1.10	no	141	Rmet_1147		0	Op0329f_1	NA	
1803	TSS_12146-2	144	33	0,1	-15	1	-38	TTGTCACCGC	-13	GATAAAGTAG	-140	1,1	7.47	8.40	s	1	Rmet_0007		0	Op0002r_2		0
1804	TSS_1800764-2	144	27	0,1	-15	1	-49	TCCAGCTCAG	-13	GCTACAATTT	-143	1,1	8.00	-16.60	no	1020	Rmet_1664		0	Op0468r_2	NA	
1805	TSS_2508529-2	144	16	0,1	-14	1	-47	TGGCCATGTC	-12	GATACAATCC	-138	1,1	10.36	-11.10	no	10	Rmet_R0038		0	Op0654r_2	NA	
1806	TSS_100199-4	144	31	0,1	-16	1	-43	TTGCTGTGCA	-14	ACTAGAATTT	-139	1,1	9.04	-1.60	no	3083	Rmet_5964	tnpA		Op1814r_1	NA	
1807	TSS_175546+2	144	19	0,1	-17	1	-46	CCAGCCCGGC	-15	CATATCCTCT	-141	5,1	7.90	-12.60	no	60	Rmet_0167		0	Op0049f_1	NA	
1808	TSS_1728487+2	144	12	0,1	-17	1	-41	TAGCGCCGCG	-15	GGTACAGTCG	-141	2,1	10.43	0.90	(w)-sba	70	Rmet_1602		0	Op0445f_1	NA	
1809	TSS_3152070-2	144	40	0,1	-15	1	-26	TCGCTGAACG	-5	CCGATCATTC	-145	1,1	4.70	0.10	no	2346	Rmet_2893	clpS		Op0812r_1	GIP-PTL-Fold	
1810	TSS_118686+5	144	25	0,1	-16	1	-34	TGGTCAGAAG	-14	CGTACGATGC	-142	1,1	7.38	9.10	s	1739	Rmet_6396		0	Op1910f_1	NA	
1811	TSS_3523613-2	143	29	0,1	-20	0	-42	TGCCAACACC	-18	ATTACATTGC	-143	1,1	5.85	1.40	(w)-sba	267	Rmet_3250	murA		Op0918r_3	MET-CAH-AminSug	
1812	TSS_832871+3	143	27	0,1	-25	0	-33	GAACCTCTCG	-13	TGTCCATACG	-145	3,0	4.94	-1.40	no	41	Rmet_4245		0	Op1202f_4	NA	
1813	TSS_1441756-3	143	19	0,1	-16	1	-35	TGCTGGCTGG	-14	CTTAGAATGT	-141	1,1	5.88	8.60	s	2337	Rmet_4794	slyA		Op1395r_1	NA	
1814	TSS_2312311-3	143	24	0,1	-10	1	-27	TTGAGCAACG	-7	GAAAGATTGG	-143	1,1	5.15	1.60	(w)-nu	3586	Rmet_6746		0	Op2099r_1	NA	
1815	TSS_1388522+2	143	25	0,1	-17	1	-47	TCGATGCCGC	-15	TGTATCCTGC	-139	1,1	6.42	-10.10	no	4946	Rmet_1271	ritA1		Op0365f_1	NA	
1816	TSS_1536454+3	143	25	0,1	-13	1	-45	TCGCCCCGGC	-11	GCCAGAATAT	-139	1,1	6.80	-10.10	no	4631	Rmet_4879	catM		Op1426f_1	NA	
1817	TSS_1129709-3	143	18	0,1	-16	1	-48	TCGATCAGCG	-14	GCTACACTCG	-140	1,1	7.88	-13.10	no	57	Rmet_4524		0	Op1297r_3	NA	
1818	TSS_1052698+3	142	16	0,1	-17	1	-35	TTGACGGTAC	-15	GATAAAATCT	-139	1,1	10.94	12.60	s	70	Rmet_4456		0	Op1268f_1	NA	
1819	TSS_1913032-3	142	6	0,1	-15	1	-39	GTCACACTTC	-13	ATTATGCTGC	-149	4,1	4.24	2.90	w	466	Rmet_5217		0	Op1537r_1	NA	
1820	TSS_1066740+2	142	22	0,1	-17	1	-42	TCCCCCGCGG	-15	GTTACCCTTC	-146	1,1	5.23	-2.60	no	2175	Rmet_0983		0	Op0277f_1	NA	
1821	TSS_2033649-3	142	16	0,1	-15	1	-33	TCGATAATGC	-13	GTCAAAATCC	-139	1,1	7.88	8.10	s	449	Rmet_5322	zniS		Op1573r_1	NA	

1822	TSS_2236181-2	142	26	0,1	-15	1	-41	GCGGCACTGG	-13	GTTATTCTAG	-138	5,1	8.93	-2.10	no	4	Rmet_6506	*0*	Op0598r_1	NA
1823	TSS_278109+2	141	28	0,1	-16	1	-44	TTGCCCTGCT	-14	CGGATTATGA	-139	1,1	6.90	-3.60	no	285	Rmet_0261	*coxB*	Op0075f_2	MET-EN-OxPhos
1824	TSS_1007220+2	141	11	0,1	-17	1	-36	TTGCGCCAAG	-15	AGTAAGTTAC	-142	1,1	5.66	9.80	s	241	Rmet_0923	*pnp*	Op0267f_1	MET-NUC-Pur
1825	TSS_2322544+2	141	27	0,1	-17	1	-49	TTGCACTAT	-15	GGCACAATAT	-141	1,1	9.60	-10.60	no	196	Rmet_2134	*trxA*	Op0615f_1	GIP-PTL-Fold
1826	TSS_2075677-2	141	15	0,1	-14	1	-46	GCAGCAAGCG	-12	GCTAGAATGG	-133	5,1	12.07	-13.60	no	4326	Rmet_1901	*0*	Op0554r_1	NA
1827	TSS_1169714-2	141	58	0,1	-14	1	-38	GGCACGAAGC	-12	AGTAAAATGC	-139	4,1	10.30	2.40	w	107	Rmet_1075	*mutS*	Op0310r_2	GIP-REP-Recomb
1828	TSS_2597466-2	141	33	0,1	-15	1	-41	TTGCGTCCTA	-13	CCTAACATGA	-145	1,1	7.26	0.40	(w)-sba	32	Rmet_2384	*armR*	Op0672r_1	NA
1829	TSS_2092785+3	141	28	0,1	-17	1	-48	GCCAGAACGT	-15	TGCATACTCC	-138	4,1	9.25	-15.60	no	1066	Rmet_5370	*bug*	Op1598f_1	NA
1830	TSS_291109+3	141	21	0,1	-16	1	-43	TCGCCACCAG	-14	TCTACAATGG	-140	1,1	9.19	-1.10	no	950	Rmet_6622	*0*	Op1044f_1	NA
1831	TSS_2235876-3	141	15	0,1	-16	1	-36	TTGACTTTTG	-14	GATAGTCTGA	-143	1,1	7.14	12.40	s	81	Rmet_5490	*pobA*	Op1631r_1	MET-XEN-24D
1832	TSS_3806752-2	140	7	0,1	-16	1	-41	TCGGCATGAC	-14	GCGACAATGC	-139	5,1	9.59	1.90	(w)-sba	541	Rmet_3514	*0*	Op0974r_5	NA
1833	TSS_258971-2	140	21	0,1	-15	1	-45	TTGCGCAGGT	-13	AGTATGCTCC	-142	1,1	8.40	-7.60	no	358	Rmet_6407	*0*	Op0072r_1	NA
1834	TSS_940973+2	140	15	0,1	-16	1	-34	TTCACAAAAC	-14	TATATAATCT	-142	1,1	10.06	12.10	s	7	Rmet_R0009	*0*	Op0247f_1	NA
1835	TSS_1420332+2	140	30	0,1	-16	1	-29	TTCTCCAGAG	-3	CCGAAGATCC	-146	1,1	4.32	-0.30	no	5092	Rmet_1309	*tnpA*	Op0375f_1	NA
1836	TSS_510839-2	140	39	0,1	-15	1	-39	TTGAGGCCAT	-13	TTTACCATCT	-142	1,1	8.24	5.40	m	1303	Rmet_0480	*tnpA*	Op0126r_1	GIP-REP-Recomb
1837	TSS_1685950-3	140	35	0,1	-16	1	-37	GGACAACAGT	-14	GCTACCATGC	-137	4,1	10.02	4.40	m	93	Rmet_5005	*0*	Op1469r_2	NA
1838	TSS_3130302+2	139	27	0,1	-18	1	-49	TAGACGCAAT	-16	GCCATAATCC	-147	1,1	6.11	-11.10	no	15	Rmet_2879	*tnpA*	Op0809f_1	NA
1839	TSS_590749-2	139	22	0,1	-4	0	-29	TTGCCCCGTT	-2	CATATGCTCC	-143	1,1	7.86	1.20	(w)-nu	7018	Rmet_0547	*0*	Op0152r_1	NA
1840	TSS_834235+3	139	16	0,1	-17	1	-44	TCGCCGATAT	-15	AGTATCCTTG	-144	1,1	6.97	-4.10	no	862	Rmet_4248	*0*	Op1202f_4	NA
1841	TSS_105249+2	139	35	0,1	-15	1	-38	TGGACAACGC	-13	TGTACCCTTG	-137	1,1	7.39	6.90	s	29	Rmet_0098	*dsbA*	Op0029f_1	GIP-PTL-Fold
1842	TSS_856908-2	139	19	0,1	-15	1	-47	TGGCCCGCTC	-13	AGTACAATCG	-138	1,1	8.81	-11.10	no	26	Rmet_0779	*maf*	Op0222r_2	DIV-Division
1843	TSS_3198998-2	139	31	0,1	-15	1	-49	TTGTGGGCGT	-13	GGTAAAATCG	-138	1,1	10.11	-14.60	no	17	Rmet_2942	*recG*	Op0830r_1	GIP-REP-Recomb
1844	TSS_97054-4	139	4	0,1	-15	1	-45	GCGGCCCCCT	-13	CTTACAATGA	-138	5,1	10.91	-10.10	no	242	Rmet_6370	*0*	Op1814r_1	NA
1845	TSS_1158967+2	138	41	0,1	-17	1	-41	TCGATGCCTC	-15	GATAGAATAG	-140	1,1	7.45	2.90	(w)-sba	705	Rmet_1067	*0*	Op0307f_1	NA
1846	TSS_728454+2	138	36	0,1	-16	1	-41	TTTCGCTGGT	-14	ACGACAATGC	-143	2,1	9.84	-0.10	no	1578	Rmet_0662	*0*	Op0183f_1	NA
1847	TSS_1060982+2	138	23	0,1	-16	1	-40	TTGCCTTTCT	-14	GGTATCCTGC	-135	1,1	9.47	4.40	(m)-sba	317	Rmet_0975	*gst*	Op0273f_1	MET-OAA-GSH
1848	TSS_3205141+2	138	49	0,1	-16	1	-41	TTGTGATTGT	-14	GCAAGAATGT	-145	1,1	7.48	2.40	(w)-sba	63	Rmet_2948	*0*	Op0831f_2	NA
1849	TSS_3562074+2	138	13	0,1	-17	1	-37	GGAGCAACAC	-15	AGTATGATAG	-137	4,1	9.58	7.40	s	108	Rmet_3286	*hemB*	Op0925f_1	MET-COF-Porph
1850	TSS_1799322-2	138	7	0,1	-14	1	-32	TGCAGATGGA	-12	AGTACTATGC	-142	1,1	6.66	5.60	m	1070	Rmet_1663	*papD*	Op0468r_2	NA
1851	TSS_2232664-2	138	43	0,4	-9	0	-32	TTCAAGCTGG	-11	CATGTGATTG	-145	1,1	1.02	6.10	m	254	Rmet_2059	*glnG*	Op0598r_3	EIP-SIG-2Comp
1852	TSS_997405+3	138	8	0,1	-16	1	-43	TGGCTGTACT	-14	TCTACACTCG	-142	1,1	7.79	-4.10	no	4009	Rmet_4405	*0*	Op1254f_1	NA
1853	TSS_224160+4	138	34	0,1	-16	1	-34	TCCCCGGGCT	-14	GCTAGATTGC	-139	1,1	6.11	6.60	s	430	Rmet_6077	*0*	Op1851f_1	NA
1854	TSS_1165229+2	137	10	0,4	-17	1	-49	TTGAAAATCG	-13	TATAAGCTGT	-142	1,1	7.85	-14.60	no	6426	Rmet_1079	*0*	Op0311f_1	GIP-TL-Ass
1855	TSS_1578354+2	137	24	0,1	-17	1	-36	TTGACGCGGC	-15	TGCAGATTCA	-141	1,1	6.11	11.80	s	339	Rmet_6449	*0*	Op0407f_1	NA
1856	TSS_2743935-2	137	26	0,1	-16	1	-39	TATGGGTGCC	-14	TCTACAATCT	-140	2,1	11.27	3.40	m	19	Rmet_2523	*uup*	Op0698r_4	NA
1857	TSS_1144374+3	137	23	0,1	-16	1	-47	TTGCCCACGT	-14	GGTAAGGTGT	-138	1,1	7.88	-10.60	no	1938	Rmet_4539	*0*	Op1300f_1	NA
1858	TSS_3046283+2	137	21	0,1	-24	0	-35	CGGCGCATAC	-15	TTCATAATTA	-146	1,1	2.86	6.10	m	468	Rmet_6559	*0*	Op0787f_1	NA
1859	TSS_991007-2	137	36	0,1	-15	1	-33	GCAGCCCGAG	-13	TGTATGCTTC	-139	5,1	9.36	2.60	w	379	Rmet_0903	*dgkA*	Op0262r_2	MET-LIP-GlycLip
1860	TSS_890004+3	137	53	0,1	-16	1	-44	GGCGCAAACT	-14	GGCATCATCA	-143	4,1	6.74	-8.60	no	1130	Rmet_6658	*0*	Op1224f_1	NA
1861	TSS_2319070-3	137	23	0,1	-15	1	-33	TTCCCCTGCG	-13	CTTAACATGC	-141	1,1	7.35	7.10	s	280	Rmet_5564	*0*	Op1653r_2	NA
1862	TSS_1028676-2	137	32	0,1	-16	1	-38	TTTTTTCGCT	-14	GGCAGGATGC	-145	2,1	8.93	4.90	m	23606	Rmet_0919	*0*	Op0264r_1	NA
1863	TSS_203717+2	136	39	0,1	-17	1	-35	TTGATCTTCC	-15	ATTACCATGA	-141	1,1	7.80	10.60	s	28	Rmet_0195	*bfr*	Op0057f_1	NA
1864	TSS_3485111-2	136	10	0,1	-17	1	-47	ATCCTGTGGG	-15	GGTATGCTTC	-138	2,1	9.66	-14.10	no	122	Rmet_3211	*0*	Op0904r_1	NA
1865	TSS_1475683+3	136	15	0,1	-7	0	-35	TTGACATTTA	-14	TACAAACTGT	-145	1,1	7.85	12.60	s	31	Rmet_4823	*0*	Op1406f_1	NA
1866	TSS_3723161-2	136	26	0,1	-15	1	-33	TTCGGTCGCG	-13	GCCAAAATGA	-136	2,1	11.40	6.10	m	23	Rmet_3447	*0*	Op0960r_1	NA
1867	TSS_2451-3	136	15	0,1	-27	0	-38	GCAACACATT	-13	GCTACAGTCG	-144	4,1	6.50	3.40	m	105	Rmet_5818	*csp*	Op1757r_1	NA
1868	TSS_2310931-3	136	25	0,1	-15	1	-34	TTGCACGAAT	-13	GTTCTAATGT	-141	1,1	6.76	9.60	s	2206	Rmet_6746	*0*	Op2099r_1	NA
1869	TSS_223178+4	136	8	0,1	-17	1	-39	GCAAGGCCAC	-15	GGTACAATCC	-137	4,1	11.77	2.40	w	23	Rmet_6079	*0*	Op1853f_3	NA
1870	TSS_269011+2	136	23	0,1	-16	1	-34	TTGCAAAGGA	-14	CCCATCATCG	-138	1,1	7.81	9.60	s	204	Rmet_0252	*grxC*	Op0073f_1	GIP-PTL-Fold
1871	TSS_402043+2	136	21	0,1	-1	0	-32	TTGTCATTTG	-5	ATCAGTTTTA	-145	1,1	3.08	3.20	(w)-ba	32	Rmet_0376	*0*	Op0103f_5	NA
1872	TSS_864238-2	136	16	0,1	-16	1	-47	TTGTGGGATG	-14	ACTACACTGT	-143	1,1	7.74	-10.60	no	39	Rmet_0787	*0*	Op0224r_1	NA
1873	TSS_1926111+3	136	19	0,1	-15	1	-46	GCTGCCAGGC	-13	GTGATCATGA	-144	5,1	8.48	-14.60	no	2414	Rmet_5231	*0*	Op1544f_1	NA
1874	TSS_1940721+2	135	19	0,1	-18	1	-34	TTCATATAAC	-6	CGTCCCATGC	-147	1,1	2.85	2.70	(w)-ba	758	Rmet_1790	*glcD*	Op0519f_2	NA
1875	TSS_1171391+3	135	32	0,1	-16	1	-40	TCAGAAGCCA	-14	GGTAAATTTA	-147	4,1	6.76	1.40	(w)-sba	40	Rmet_4562	*0*	Op1312f_1	NA
1876	TSS_3507350-2	135	33	0,1	-16	1	-45	TTCCAGATTA	-14	TGCATCATCG	-145	1,1	6.35	-8.10	no	151	Rmet_3230	*petA*	Op0914r_1	MET-EN-OxPhos
1877	TSS_3627229-2	135	46	0,1	-9	0	-34	GTAAAGAAAT	-7	GTAAGAATAT	-148	4,1	7.55	1.70	(w)-ba	51	Rmet_3350	*0*	Op0932r_1	NA
1878	TSS_120149-3	135	27	0,1	-14	1	-45	TTGTCCAGAA	-12	GTTAAGATGC	-138	1,1	9.07	-7.60	no	2997	Rmet_5915	*0*	Op1795r_1	NA
1879	TSS_3821545-2	135	8	0,1	-15	1	-35	CCGGCAATAG	-13	CGTATCATCG	-134	5,1	10.40	9.70	s	34	Rmet_3528	*0*	Op0976r_2	NA
1880	TSS_1237608-2	134	32	0,1	-15	1	-47	TTTCTCCTCG	-13	AATATAATAC	-138	2,1	12.75	-11.10	no	1	Rmet_1130	*wrbA*	Op0322r_1	NA
1881	TSS_968261-3	134	15	0,1	-15	1	-44	GTACAGGACG	-13	GCTATCCTGG	-140	4,1	7.49	-10.10	no	4833	Rmet_4365	*cbbR3*	Op1245r_1	NA
1882	TSS_3305310+2	134	12	0,1	-15	1	-48	TTTCGCCGCG	-13	GTCATGATCC	-137	2,1	12.36	-15.10	no	64	Rmet_3048	*pgpA*	Op0857f_1	MET-LIP-GlycPLip

1883	TSS_3526809-2	134	25	0,4	-17	1	-47	ACATTTCGCG	-27	GCCCAACGCC	-145	3,0	5.04	-12.20	no	47	Rmet_3255	ttg2D		Op0918r_3	EIP-TRA-ABC
1884	TSS_103116+4	134	32	0,1	-17	1	-42	TGCCAATAGT	-15	GGCAAGATTC	-141	1,1	6.40	-2.60	no	63	Rmet_5957	tnpA		Op1813f_3	NA
1885	TSS_1850348+2	133	32	0,1	-16	1	-35	TTGCTTTGCG	-14	CTGACTCTTT	-143	2,1	8.17	8.60	s	349	Rmet_1709		0	Op0483f_1	NA
1886	TSS_3301381+2	133	12	0,1	-17	1	-44	TGAGCATGA	-15	TGTAACCTTG	-138	1,1	6.63	-5.10	no	2918	Rmet_3047	thiL		Op0857f_1	MET-COF-Thia
1887	TSS_3466103-2	133	10	0,1	-15	1	-36	GGCCACAAACC	-13	TTTACCATTC	-140	4,1	9.10	7.40	s	44	Rmet_3190	dsbC		Op0896r_1	GIP-PTL-Fold
1888	TSS_2067748-3	133	31	0,1	-15	1	-43	CCGGCCGTGC	-13	GTAATGATTG	-141	5,1	7.81	-7.10	no	945	Rmet_5347		0	Op1589r_2	NA
1889	TSS_201023-4	133	8	0,1	-16	1	-38	GCACCAAAAT	-14	GCTATAGTCT	-141	4,1	10.22	4.40	m	19	Rmet_6100		0	Op1860r_1	NA
1890	TSS_396525-2	133	9	0,1	-15	1	-46	TTGAAGTATT	-13	TCCATCATGC	-141	1,1	8.38	-8.60	no	8680	Rmet_0365	coxS		Op0102r_1	MET-EN-Methane
1891	TSS_3616141-2	133	10	0,3	-22	0	-45	TTGTAGTCAA	-12	CTGAGTGTTT	-147	1,1	2.21	-10.60	no	225	Rmet_R0061		0	Op0930r_1	NA
1892	TSS_170434-4	133	45	0,1	-15	1	-36	TTGCCGGAAG	-13	GCTAGGATTC	-140	1,1	9.28	11.40	s	16	Rmet_6133	silD		Op1874r_1	NA
1893	TSS_417261-3	132	14	0,1	-16	1	-34	TTGCCGGCAA	-14	GAGACAATCG	-137	1,1	8.19	9.60	s	56	Rmet_3873		0	Op1079r_2	NA
1894	TSS_772384-2	132	25	0,1	-15	1	-32	TTGCCCAATG	-11	CAGAATATGT	-142	1,1	5.74	7.60	s	101	Rmet_0699		0	Op0196r_1	NA
1895	TSS_2782228-2	132	30	0,1	-15	1	-31	TTTGGTTGCG	-8	AATAGGATCA	-142	2,1	11.31	4.70	(m)-ba	394	Rmet_2558		0	Op0708r_2	NA
1896	TSS_105095-3	132	6	0,1	-15	1	-47	TGGCCGCGCA	-13	CCTATAGTGA	-141	1,1	6.89	-11.10	no	46	Rmet_5904		0	Op1791r_1	NA
1897	TSS_1364999-2	132	31	0,1	-15	1	-33	TGCAGAAACG	-13	GGGATAATCA	-139	1,1	6.69	7.60	s	9476	Rmet_1232	yhgF		Op0356r_1	NA
1898	TSS_2957538-2	132	24	0,1	-19	0	-34	TTGACTGCCT	-11	GTCAAATTGG	-143	1,1	7.03	10.20	s	549	Rmet_6553		0	Op2041r_1	NA
1899	TSS_1489286+3	132	28	0,1	-16	1	-40	GCCAAGGCAA	-14	TGTATATTTC	-143	4,1	8.28	-0.60	no	301	Rmet_4835		0	Op1410f_1	NA
1900	TSS_944591-3	132	43	0,1	-14	1	-34	TTGACAGTCC	-12	GTCACGATCA	-139	1,1	7.68	12.20	s	709	Rmet_4346		0	Op1237r_2	NA
1901	TSS_1262923-2	131	10	0,1	-16	1	-43	TGCCAACGCC	-14	GGGATAATGC	-141	1,1	7.47	-3.60	no	5247	Rmet_1146	etfD		Op0328r_1	NA
1902	TSS_160080+4	131	38	0,1	-16	1	-37	TTGTTCGTGC	-14	GCTAGAATTT	-138	1,1	9.33	10.40	s	2224	Rmet_6137	ubiE		Op1875f_1	NA
1903	TSS_150966-4	131	14	0,1	-16	1	-36	GGAGAGATCC	-14	GCTACCCTGC	-140	4,1	8.20	4.40	m	3014	Rmet_6379		0	Op1879r_1	NA
1904	TSS_1711245+2	131	24	0,1	-17	1	-36	TTGAAATGCC	-15	TGCAGAATCC	-140	1,1	9.10	12.80	s	118	Rmet_1588	prpR		Op0439f_1	NA
1905	TSS_952757-2	131	22	0,1	-16	1	-47	TTGAGCCCAC	-14	GGTAAACTAG	-139	1,1	9.17	-9.60	no	4	Rmet_0871		0	Op0252r_1	NA
1906	TSS_1163808-2	131	60	0,1	-16	1	-41	GGAGAGCCAC	-14	GCGATAATTA	-143	4,1	8.95	-3.60	no	86	Rmet_1071		0	Op0308r_2	NA
1907	TSS_1923492-2	131	23	0,3	-4	0	-34	TTCTCCTGCC	-13	GGCAGACTGC	-139	1,1	6.25	8.10	s	36	Rmet_1770	gatA		Op0510r_2	GIP-TL-Ass
1908	TSS_3189850-2	131	20	0,1	-15	1	-37	CCTGAACGTG	-13	GGTATTCTCA	-140	5,1	8.35	3.40	m	33	Rmet_2934	gpo		Op0828r_1	MET-LIP-Others
1909	TSS_3758262-2	131	5	0,1	-15	1	-39	CCGGCATCGC	-13	GGTACATTGG	-142	5,1	9.43	1.90	w	782	Rmet_3476	envZ		Op0968r_1	NA
1910	TSS_174919+3	131	6	0,1	-16	1	-47	GCCCGGGCAC	-14	CGCATAATGT	-140	4,1	9.03	-15.60	no	103	Rmet_3640		0	Op1014f_1	NA
1911	TSS_2495877-3	131	25	0,1	-16	1	-44	TGACCACCGC	-14	GCGAAACTTG	-143	4,1	6.25	-6.60	no	3070	Rmet_5732	kpsM		Op1715r_1	NA
1912	TSS_75144+4	131	22	0,4	-8	0	-32	AACTTCGGCG	-12	CGTTAGTACT	-146	3,0	5.77	-4.40	no	31	Rmet_5983	czcI		Op1820f_1	NA
1913	TSS_61215-4	131	21	0,1	-15	1	-33	TCGTCACGGC	-13	TCTACCATGC	-138	1,1	7.25	8.10	s	856	Rmet_5998	atsH		Op1828r_2	NA
1914	TSS_190993-2	130	36	0,1	-14	1	-47	GCTGCAGGTT	-12	GGGATAATCG	-138	5,1	10.44	-15.60	no	1959	Rmet_0181		0	Op0054r_1	NA
1915	TSS_303664-2	130	36	0,1	-16	1	-49	TTTTTTTTCG	-28	CGTACGCTTT	-139	2,1	10.37	-4.70	no	50	Rmet_0287	pth		Op0084r_4	GIP-TL-Ass
1916	TSS_1083157-2	130	4	0,1	-15	1	-33	TGGAGAGCGG	-13	CGGATAATCT	-140	1,1	7.11	9.10	s	96	Rmet_0993		0	Op0282r_1	NA
1917	TSS_3213988-2	130	29	0,1	-16	1	-42	TTGCCGCCAC	-14	GTTACGATCA	-137	1,1	8.85	0.40	(w)-sba	179	Rmet_2955	yceJ		Op0832r_1	NA
1918	TSS_2506689+3	130	57	0,1	-16	1	-35	TTGTAATGAG	-14	TTTACTATTC	-144	1,1	8.39	11.60	s	366	Rmet_5746	fur		Op1718f_2	NA
1919	TSS_750560-3	130	18	0,1	-15	1	-43	GCTGCCCCCG	-13	GTCACAATCT	-136	5,1	9.56	-8.60	no	85	Rmet_4165		0	Op1171r_1	NA
1920	TSS_228246+4	130	24	0,1	-17	1	-36	TTGTGACACT	-15	CGTATCATCC	-141	1,1	8.49	12.80	s	720	Rmet_6071		0	Op1849f_1	NA
1921	TSS_2004426-2	130	9	0,1	-15	1	-45	TTCCCGCATG	-13	AGCATAATTG	-141	1,1	7.59	-7.10	no	513	Rmet_1849		0	Op0536r_1	NA
1922	TSS_2491258+3	130	30	0,1	-16	1	-42	TGGCAACGCC	-14	TTTATACTTT	-140	1,1	8.65	-0.10	no	1743	Rmet_6764		0	Op1716f_1	NA
1923	TSS_1101326-3	130	19	0,1	-16	1	-34	TTCTTCGTAA	-14	GTTACATTTG	-146	1,1	6.16	7.10	s	211	Rmet_4499	rpoJ		Op1281r_1	GIP-TK-RNAP
1924	TSS_2301475-2	129	61	0,1	-14	1	-33	TTGAGCAGCG	-12	GGTATCTTGG	-137	1,1	8.09	9.60	s	908	Rmet_2118	surE		Op0612r_1	MET-NUC-Pur
1925	TSS_951013-3	129	23	0,1	-15	1	-47	TTGCTATATC	-13	GCTACCATGT	-140	1,1	9.58	-10.60	no	52	Rmet_4355		0	Op1241r_1	NA
1926	TSS_211831+2	129	36	0,1	-10	1	-36	TTCCAAGACC	-8	TTCACAATGA	-146	1,1	7.30	4.90	(m)-ba	120	Rmet_0203		0	Op0059f_3	NA
1927	TSS_1971191+2	128	21	0,1	-17	1	-38	TTGCGCAATC	-15	GGTAAGATGA	-139	1,1	9.40	8.40	s	13	Rmet_1817		0	Op0527f_1	NA
1928	TSS_887255-2	128	21	0,1	-15	1	-37	CCAGCAAGCT	-13	TCTACAATGT	-136	5,1	11.90	5.40	m	174	Rmet_0812	fbp		Op0230r_3	GIP-PTL-Fold
1929	TSS_3110492-2	128	31	0,1	-16	1	-44	TGGCCGCTGG	-14	GCCATAATCG	-140	1,1	8.60	-4.10	no	30	Rmet_2860		0	Op0804r_1	NA
1930	TSS_750170-2	128	8	0,1	-14	1	-32	TTGCACGCCC	-12	GCGATAATGC	-137	1,1	9.34	7.60	s	49	Rmet_0677	puuC		Op0188r_1	MET-CAH-FbP
1931	TSS_3245322-2	128	24	0,1	-15	1	-32	TTCTCGAACT	-10	AAGATGGTGA	-147	1,1	2.19	6.70	s	154	Rmet_6571		0	Op0842r_3	NA
1932	TSS_3566553-2	128	45	0,1	-25	0	-46	GTAGCGGCGT	-23	GGCACCATGC	-138	4,1	7.11	-4.10	no	296	Rmet_3288	dsbD		Op0926r_2	GIP-PTL-Fold
1933	TSS_225276+3	128	35	0,1	-17	1	-28	TTCACTTACA	-6	TGTAACGTTT	-146	1,1	5.27	3.70	(m)-nu	150	Rmet_3685	flhD2		Op1026f_3	EIP-SIG-2Comp
1934	TSS_729810+3	128	33	0,1	-18	1	-38	CTGTTCAGAT	-16	GCCATAATCC	-146	1,1	4.26	7.40	s	15	Rmet_4154	tnpA		Op1170f_1	NA
1935	TSS_1044123+3	128	19	0,1	-17	1	-44	TCGCCGCGCA	-15	GCTAACATAT	-140	1,1	6.90	-4.10	no	29	Rmet_4447		0	Op1264f_2	NA
1936	TSS_202114+4	128	13	0,1	-17	1	-40	TACCGAGCAC	-15	GATATCCTCG	-146	1,1	2.81	2.40	(w)-sba	88	Rmet_6098	npd-a		Op1859f_2	NA
1937	TSS_475669+2	127	54	0,1	-1	0	-35	TTCAGAACCG	-12	GATAACGTTT	-144	1,1	5.38	9.70	s	8548	Rmet_0456	rhlE1		Op0119f_1	NA
1938	TSS_36516-2	127	19	0,1	-15	1	-45	TATCGTCGGT	-13	GGTATCCTGT	-141	2,1	10.09	-10.60	no	343	Rmet_0033	minE		Op0012r_2	DIV-Division
1939	TSS_2579086-3	127	18	0,1	-15	1	-35	TGGATCAACA	-13	GGGATCATCT	-144	1,1	5.88	10.70	s	85	Rmet_5815		0	Op1755r_1	NA
1940	TSS_2996659-2	127	9	0,1	-15	1	-48	CCAGCAGGGT	-13	ATTACAATGC	-136	5,1	11.45	-16.60	no	141	Rmet_2754	ldcC		Op0776r_2	MET-AA-GluGSH
1941	TSS_1784867+3	127	20	0,1	-16	1	-47	GCGAAAGCCC	-14	GCTACGCTGC	-139	5,1	7.12	-14.10	no	21	Rmet_5094		0	Op1498f_1	MET-CAH-Prop
1942	TSS_182263+2	127	5	0,1	-17	1	-46	TTGTCCGGTG	-15	GCAATAATGG	-140	1,1	7.86	-5.60	no	117	Rmet_0176		0	Op0053f_1	NA
1943	TSS_2156734+2	127	37	0,1	-17	1	-32	AAATTTCTCG	-12	AGGGTAACAA	-142	3,0	9.15	-2.40	no	131	Rmet_1984	asdA		Op0579f_1	NA

1944	TSS_324264+3	127	24	0,1	-8	0	-32	TCGAAATGGA	-6	GCTACGATGA	-138	1,1	8.62	4.70	(m)-ba	70	Rmet_3787	0	Op1050f_3	NA
1945	TSS_1951308-3	127	24	0,1	-24	0	-49	GCCAACCCGC	-22	GGGAAATTGC	-141	4,1	7.01	-12.60	no	453	Rmet_5250	0	Op1549r_1	DIV-MOT-Chemotax
1946	TSS_22028+2	126	11	0,1	-17	1	-43	TGCCCGAATG	-15	CGTAGACTTC	-142	1,1	6.47	-3.60	no	37	Rmet_0018	0	Op0003f_3	MET-LIP-Others
1947	TSS_894581-2	126	9	0,1	-15	1	-33	TGGCATGGCG	-13	CGTACACTTG	-139	1,1	7.57	6.10	m	901	Rmet_0820	0	Op0232r_2	NA
1948	TSS_2320295-2	126	44	0,1	-15	1	-48	GCAGCCACGC	-13	GCGATGCTGT	-141	4,1	7.05	-19.60	no	1281	Rmet_2131	addA	Op0614r_1	GIP-REP-Recomb
1949	TSS_2641313-2	126	21	0,1	-15	1	-39	GCCGGCGAGT	-13	GCTAAACTTC	-140	4,1	9.32	-1.60	no	455	Rmet_2430	fabH	Op0680r_2	MET-LIP-FASyn
1950	TSS_19222-5	126	24	0,1	-10	1	-35	TTGACAAGTA	-8	TTTACTATCG	-144	1,1	9.11	9.20	(s)-ba	19	Rmet_6329	0	Op1923r_1	NA
1951	TSS_35084+2	126	24	0,1	-17	1	-42	TGGACGAGGT	-15	TTTATAATGC	-136	1,1	10.75	2.90	(w)-sba	2901	Rmet_0036	corA1	Op0013f_1	EIP-TRA-Ion
1952	TSS_1979331-2	126	59	0,5	-26	0	-42	TCGCCGGGCT	-22	CGCAATCTGC	-143	1,1	4.25	1.30	w	82	Rmet_1822	0	Op0528r_1	NA
1953	TSS_1449975+3	126	5	0,1	-17	1	-35	GCGTCATCCT	-15	GATACCATGA	-143	5,1	7.10	7.10	s	448	Rmet_4802	0	Op1396f_6	NA
1954	TSS_177037-3	126	14	0,1	-16	1	-41	TCGACGCTTT	-14	GGCAGAATCT	-137	1,1	7.50	1.90	(w)-sba	804	Rmet_3641	0	Op1015r_1	NA
1955	TSS_762705-3	126	24	0,1	-15	1	-35	TTGACTGCGA	-13	ATGACAATGC	-140	1,1	7.74	13.20	s	2201	Rmet_4174	0	Op1175r_1	NA
1956	TSS_530858+2	126	20	0,1	-17	1	-46	TTGACGTGAG	-15	AGTACATTAA	-143	1,1	7.68	-5.60	no	138	Rmet_0501	pgk2	Op0135f_1	MET-CAH-FbP
1957	TSS_138160-3	126	22	0,1	-14	1	-33	TGGCGCCACG	-12	TGGAGAATGC	-138	1,1	6.29	7.10	s	33	Rmet_5934	0	Op1803r_1	NA
1958	TSS_1387463-3	126	27	0,1	-14	1	-44	GTACGAAAAC	-12	GCTATGATTC	-140	4,1	11.49	-9.10	no	2503	Rmet_4739	nnrU	Op1373r_1	NA
1959	TSS_514612+2	125	30	0,1	-16	1	-43	TTGCCCGATG	-14	GCTATCATAG	-138	1,1	9.37	-0.60	no	6278	Rmet_0494	0	Op0133f_1	NA
1960	TSS_2097259+2	125	31	0,3	-28	0	-36	TTGACGATCT	-12	TATACGTTCT	-138	1,1	6.81	10.40	s	800	Rmet_1930	0	Op0563f_1	NA
1961	TSS_3482837+2	125	9	0,1	-17	1	-42	TTGTAGCCAC	-15	GGCATAATCC	-141	1,1	9.00	1.40	(w)-sba	36	Rmet_3209	0	Op0903f_1	NA
1962	TSS_1078905-2	125	33	0,1	-16	1	-45	TCGTGGGCCG	-24	ACCAAAGTGG	-144	1,1	3.01	-0.70	no	69	Rmet_0992	nit	Op0282r_1	MET-EN-Nitrog
1963	TSS_72769+3	125	14	0,1	-22	0	-36	TTGCGCAGGC	-6	GCTACTAACC	-144	1,1	4.16	1.40	(w)-ba	57	Rmet_5876	0	Op1778f_2	NA
1964	TSS_96132-4	125	29	0,1	-14	1	-45	TCGAACCCGA	-12	GTTACCATGA	-141	1,1	7.35	-9.10	no	340	Rmet_5966	gtrM1	Op1816r_1	NA
1965	TSS_2960934+2	125	21	0,1	-15	1	-36	TCGAACTCGC	-13	GGTCCAATAG	-144	1,1	4.50	8.90	s	338	Rmet_6554	0	Op2042f_1	NA
1966	TSS_3895364+2	125	10	0,1	-16	1	-48	TTTATCCGGG	-14	TCTATAATAC	-143	2,1	11.49	-11.10	no	248	Rmet_3588	acrA	Op0999f_2	EIP-TRA-Other
1967	TSS_2457302-2	125	25	0,1	-15	1	-46	TCGCGATGCG	-13	GCTAGGATGC	-139	1,1	7.96	-10.10	no	27	Rmet_2241	0	Op0642r_1	NA
1968	TSS_2097348+3	125	16	0,1	-17	1	-43	GCGGATGTCC	-15	TCTATAGTGA	-141	5,1	7.70	-5.10	no	848	Rmet_5373	0	Op1600f_1	EIP-TRA-Pores
1969	TSS_1504404+3	125	9	0,1	-2	0	-44	TTGTGCCAAT	0	GATATGCTGT	-141	1,1	7.69	-18.60	no	130	Rmet_4847	0	Op1412f_2	NA
1970	TSS_122669+5	125	5	0,1	-16	1	-43	GCCCGACCGT	-14	GGCATAATGC	-137	4,1	9.92	-6.60	no	22	Rmet_6267	0	Op1910f_1	NA
1971	TSS_1415478-2	124	7	0,4	-23	0	-41	GCCCCGACGC	-13	CGTACATTGT	-141	4,1	8.20	-4.60	no	56	Rmet_1298	hoxK	Op0370r_1	NA
1972	TSS_2097855-2	124	20	0,1	-15	1	-34	TTGTGCTCAG	-13	ACTAGTATCA	-145	1,1	6.09	9.60	s	759	Rmet_1928	0	Op0562r_1	NA
1973	TSS_3152225-2	124	11	0,1	-16	1	-46	GCGGCCGGTC	-14	GTTACAATGC	-139	5,1	12.24	-11.10	no	71	Rmet_2895	icd	Op0814r_1	MET-CAH-TCA
1974	TSS_2236199+2	124	16	0,1	-16	1	-46	TGGACATTTT	-14	GCTAGAATAA	-138	1,1	9.53	-6.10	no	94	Rmet_2063	0	Op0599f_1	NA
1975	TSS_3361548+2	124	28	0,1	-16	1	-47	GTAACCGAGG	-14	GTTACCATTC	-138	4,1	8.17	-13.10	no	82	Rmet_3102	0	Op0873f_1	NA
1976	TSS_290872-3	124	7	0,1	-16	1	-39	TGGACACCAC	-14	CCTACCATTT	-141	1,1	7.59	6.90	s	21	Rmet_6621	0	Op1043r_1	NA
1977	TSS_782245-3	124	16	0,1	-10	1	-40	TGGACCCACG	-8	CGAACACTGG	-143	1,1	4.94	-3.10	no	73	Rmet_4196	0	Op1189r_2	NA
1978	TSS_2311202-3	124	14	0,1	-16	1	-46	TCGTGAAATA	-14	TCTACAATCG	-142	1,1	8.22	-8.10	no	2477	Rmet_6746	0	Op2099r_1	NA
1979	TSS_930016+2	123	19	0,1	-16	1	-49	CCGGCCGCGC	-14	AGTATCATGA	-140	5,1	9.78	-17.10	no	143	Rmet_0849	0	Op0243f_1	NA
1980	TSS_7562+5	123	11	0,1	-17	1	-35	TGCTAATCGT	-15	GGTAGACTAT	-146	1,1	6.29	7.60	s	30	Rmet_6321	tolA	Op1920f_1	NA
1981	TSS_14603+5	123	20	0,1	-16	1	-41	TGGAATATCA	-14	GACATAATCG	-143	1,1	8.13	1.90	(w)-sba	1927	Rmet_6327	0	Op1922f_1	NA
1982	TSS_1684796+2	123	18	0,1	-17	1	-36	TCGTCTCGGC	-15	AGTATCATTC	-140	1,1	6.82	11.30	s	213	Rmet_1564	tnpB	Op0431f_4	NA
1983	TSS_26922-2	123	19	0,1	-15	1	-34	TGGCAAACTG	-13	GCGAGAATGA	-138	1,1	7.09	9.10	s	20	Rmet_0022	0	Op0004r_1	NA
1984	TSS_2611931-2	123	12	0,1	-15	1	-34	TTGTCTGGGC	-13	ACCACAATGG	-139	1,1	7.72	10.60	s	376	Rmet_2401	0	Op0674r_1	NA
1985	TSS_3413065-2	123	13	0,1	-16	1	-41	TTCCCAATGG	-14	GCGAGAATGG	-137	1,1	7.91	1.90	(w)-sba	847	Rmet_3148	fadD	Op0882r_3	MET-LIP-FAMet
1986	TSS_3235273+2	122	33	0,1	-16	1	-47	GCCAAAACCC	-14	TGTACTCTAG	-142	4,1	8.15	-15.60	no	7	Rmet_2976	fur	Op0837f_1	NA
1987	TSS_2162790+3	122	11	0,1	-16	1	-44	GTCAGAAAAC	-14	CCTAGAATGG	-136	4,1	10.81	-6.10	no	40	Rmet_5417	0	Op1612f_2	NA
1988	TSS_8658+4	122	13	0,1	-17	1	-49	TGGCAACGGC	-15	ATTACAATTC	-138	1,1	9.42	-13.10	no	1360	Rmet_6057	0	Op1845f_1	NA
1989	TSS_3046918+2	122	10	0,1	-17	1	-35	TGGCGGCGGT	-15	GCGAAAATGC	-138	1,1	7.79	8.10	s	94	Rmet_2800	0	Op0787f_2	NA
1990	TSS_631431-2	122	71	0,1	-16	1	-40	TCGGCAAGAT	-14	GGCACACTGT	-138	5,1	10.16	2.90	(w)-sba	1085	Rmet_0587	0	Op0166r_3	NA
1991	TSS_2043398+3	122	52	0,1	-17	1	-45	CCGTATGCGT	-15	GATAGAATCA	-143	5,1	6.85	-9.10	no	18	Rmet_5331	0	Op1578f_1	EIP-SIG-2Comp
1992	TSS_650049-3	122	23	0,1	-4	0	-35	TGGACACTAT	-14	CGGATCATTC	-143	1,1	6.55	12.10	s	30	Rmet_6646	0	Op1147r_3	NA
1993	TSS_1789356-3	122	4	0,1	-15	1	-47	TCAGGACCGC	-13	GGTACTATCC	-143	4,1	8.92	-13.60	no	997	Rmet_5096	aldH	Op1499r_1	NA
1994	TSS_2434662-3	122	28	0,1	-15	1	-48	TGCCGGGTAC	-13	ACTATAATTC	-143	1,1	8.09	-14.60	no	54	Rmet_5676	0	Op1697r_2	NA
1995	TSS_500718+2	122	16	0,1	-22	0	-41	TTGAAACCGA	-20	CGTACGATAA	-142	1,1	8.11	7.80	s	82	Rmet_0471	0	Op0123f_3	NA
1996	TSS_2071569+2	121	33	0,1	-14	1	-35	TTGCCACGAC	-14	GCGATATTGT	-141	1,1	7.82	12.60	s	562	Rmet_1903	0	Op0555f_1	NA
1997	TSS_2872674+2	121	37	0,1	-16	1	-43	TTGCTGAGGT	-14	CTCACAATAA	-142	1,1	7.33	-2.60	no	44	Rmet_2636	0	Op0741f_1	GIP-PTL-Fold
1998	TSS_3568399+2	121	27	0,1	-16	1	-35	TTGCATTAGG	-14	AATACCCTCG	-142	1,1	6.87	9.60	s	811	Rmet_6589	0	Op2054f_1	NA
1999	TSS_1333327-2	121	23	0,1	-15	1	-34	GTAAAAACGG	-13	GCTACAATGC	-134	4,1	12.32	8.10	s	0	Rmet_1213	pyrD	Op0352r_1	MET-NUC-Pyr
2000	TSS_46185+4	121	3	0,1	-16	1	-46	TGCCCCTGGT	-24	CGTATTTTGG	-143	1,1	5.22	-0.60	no	163	Rmet_6011	0	Op1833f_1	NA
2001	TSS_1152443-2	121	17	0,1	-9	0	-29	TCGCGACGGC	-7	CCTACATTGC	-140	1,1	7.08	4.70	(m)-nu	19	Rmet_1059	paaY	Op0302r_1	NA
2002	TSS_2298826-2	121	12	0,1	-14	1	-34	TTGAAGCGGT	-12	CTTAAAATCG	-139	1,1	10.31	12.20	s	17	Rmet_2116	0	Op0612r_2	NA
2003	TSS_2713315-2	121	17	0,1	-16	1	-47	TCGCCGCTGT	-14	GTCACAATCG	-141	1,1	7.90	-11.10	no	40	Rmet_2498	0	Op0692r_2	NA
2004	TSS_1796398+2	121	42	0,1	-16	1	-35	TTGTGCGCCA	-14	GCGACAATCC	-139	1,1	7.29	10.60	s	1002	Rmet_6465	0	Op2012f_1	NA

2005	TSS_17303-2	121	19	0,1	-16	1	-35	TCGACACGGC	-14	CATAGGCTCG	-138	1,1	5.68	11.10	s	0	Rmet_0012	*0*	Op0002r_1		0
2006	TSS_2680195-2	120	25	0,1	-16	1	-42	CCGGACGGGC	-14	AGTATCCTGA	-140	5,1	8.54	-5.10	*no*	66	Rmet_2468	*trpF*	Op0688r_3	MET-AA-PheTyrTrp	
2007	TSS_3922808-2	120	12	0,1	-16	1	-48	TTGCCACCCC	-14	GCGACAATAC	-139	1,1	8.52	-10.60	*no*	0	Rmet_3610	*trmE*	Op1004r_2	NA	
2008	TSS_1297753-3	120	15	0,1	-13	1	-34	TTGTATTGTA	-11	ATCATAATAC	-145	1,1	7.37	10.20	s	111	Rmet_4664	*0*	Op1349r_1	NA	
2009	TSS_1383298-3	120	21	0,1	-16	1	-37	TTGCCGCGAT	-14	CCTACAATTA	-144	1,1	10.17	11.40	s	75	Rmet_4737	*aceA*	Op1371r_1	MET-CAH-DiCarb	
2010	TSS_3578637+2	120	25	0,1	-16	1	-34	TCGTCAGGCC	-14	GGCAGAATGG	-139	1,1	7.15	9.10	s	7036	Rmet_3322	*0*	Op0927f_1	NA	
2011	TSS_235855-2	120	3	0,1	-15	1	-35	CCGCGCGTGC	-13	GGTAGCATAA	-143	1,1	1.77	6.70	s	36	Rmet_0221	*0*	Op0066r_1	NA	
2012	TSS_721789-3	120	16	0,1	-16	1	-35	TTGACAGTCC	-14	CCTATTCTCC	-138	1,1	8.24	13.60	s	59	Rmet_4146	*bugT*	Op1169r_3	NA	
2013	TSS_783048-3	120	4	0,1	-16	1	-44	TTCTCGGGCG	-14	GTCATAATGC	-144	1,1	7.70	-4.10	*no*	876	Rmet_4196	*0*	Op1189r_2	NA	
2014	TSS_1165463-3	120	17	0,1	-16	1	-47	TTGCAGACTC	-14	CATAAACTCT	-142	1,1	8.39	-10.60	*no*	4666	Rmet_4549	*uvrA2*	Op1305r_1	GIP-REP-Recomb	
2015	TSS_1279374-3	120	26	0,1	-15	1	-34	TTGTCCATGC	-13	GCCATAATCC	-139	1,1	9.58	11.60	s	0	Rmet_4648	*0*	Op1343r_2	NA	
2016	TSS_2371179-3	120	21	0,1	-16	1	-41	TCTGACGTCT	-14	CGTAGAATAG	-141	5,1	9.33	-0.60	*no*	1487	Rmet_5612	*tar*	Op1675r_3	DIV-MOT-Chemotax	
2017	TSS_424430-2	119	15	0,1	-17	1	-45	TTCCCAAAAA	-15	ATTACAATTC	-139	1,1	9.48	-4.10	*no*	81	Rmet_0396	*0*	Op0108r_1	NA	
2018	TSS_150407+3	119	17	0,3	-23	0	-46	TTGTAGTCAA	-13	CTGAGTGTTT	-147	1,1	2.21	-11.60	*no*	224	Rmet_R0073	*0*	Op1006f_1	NA	
2019	TSS_1594184+3	119	9	0,1	-16	1	-34	TTCAATGATT	-14	TATATGATTT	-141	1,1	7.76	9.10	s	45	Rmet_4931	*bug*	Op1446f_1	NA	
2020	TSS_462617-3	119	10	0,4	-16	1	-34	TTGACGGTGC	-12	CATAAGGTTC	-145	1,1	6.56	11.20	s	5	Rmet_3914	*0*	Op1095r_1	NA	
2021	TSS_3254699+2	119	17	0,1	-16	1	-36	TCGACAAGCT	-14	TGTATCTTGG	-142	1,1	6.90	12.90	s	2783	Rmet_2999	*mmfA*	Op0845f_1	NA	
2022	TSS_616598-2	119	22	0,1	-15	1	-43	TGGATGATGG	-13	CCTACAATTT	-137	1,1	8.71	-3.10	*no*	5387	Rmet_0571	*modA*	Op0160r_1	EIP-TRA-ABC	
2023	TSS_1284143+2	119	6	0,1	-16	1	-40	TCGTGATAAC	-14	TGCACAATAG	-144	1,1	6.37	2.90	*(w)-sba*	770	Rmet_1174	*0*	Op0337f_1	NA	
2024	TSS_571817-2	119	6	0,1	-15	1	-39	TTCTGATTTC	-13	TGTAGAATTC	-138	1,1	8.56	4.90	m	6	Rmet_0537	*exbB2*	Op0148r_1	NA	
2025	TSS_771272-2	119	23	0,1	-15	1	-34	TTGACAGGCC	-13	ACCAATATCA	-140	1,1	6.36	11.60	s	25	Rmet_0698	*0*	Op0196r_1	NA	
2026	TSS_3430908-2	119	9	0,3	-22	0	-45	TTGTAGTCAA	-12	CTGAGTGTTT	-147	1,1	2.21	-10.60	*no*	225	Rmet_R0051	*0*	Op0886r_1	NA	
2027	TSS_1497344-2	118	43	0,1	-15	1	-46	TTGATATTTC	-13	ACTACAATCA	-139	1,1	9.86	-6.60	*no*	53	Rmet_1387	*uspA3*	Op0386r_1	NA	
2028	TSS_2108627-2	118	24	0,1	-16	1	-40	TGGCAGCCAA	-14	GGCACACTCA	-141	1,1	6.23	0.90	*(w)-sba*	8522	Rmet_1931	*amaB-2*	Op0564r_1	MET-NUC-Pyr	
2029	TSS_1958528-3	118	74	0,1	-15	1	-34	TTTGGCCGGC	-13	GGTACAGTGC	-140	2,1	10.09	8.10	s	80	Rmet_5259	*fliE*	Op1551r_1	DIV-MOT-Flagel	
2030	TSS_3072418-2	118	12	0,1	-16	1	-39	TCGCGGACCA	-14	GGTAATGTTG	-145	1,1	5.10	2.90	w	1528	Rmet_2822	*0*	Op0794r_1	NA	
2031	TSS_307776+3	118	30	0,1	-17	1	-42	TGGACGAGGT	-15	TTTATAATGC	-136	1,1	10.75	2.90	*(w)-sba*	155	Rmet_3772	*0*	Op1048f_2	NA	
2032	TSS_226413-4	118	10	0,1	-15	1	-36	GCAAGGGCGC	-13	TCTACAATGG	-137	4,1	11.69	6.40	s	2367	Rmet_6078	*0*	Op1852r_1	NA	
2033	TSS_3440879+2	117	23	0,1	-17	1	-38	TCGCCACCAG	-15	GGTATCATCC	-139	1,1	8.64	9.90	s	7010	Rmet_3174	*0*	Op0891f_1	NA	
2034	TSS_3574576+2	117	27	0,1	-16	1	-39	TCGCTCATGC	-14	TGTAGCCTGT	-143	1,1	5.54	2.90	w	11097	Rmet_3322	*0*	Op0927f_1	NA	
2035	TSS_337056-2	117	16	0,1	-15	1	-38	TTGCTGGTCG	-13	AGTACGCTCC	-141	1,1	6.72	5.40	m	4938	Rmet_0313	*uvrA1*	Op0090r_1	GIP-REP-Recomb	
2036	TSS_584997+3	117	1	0,1	-16	1	-35	TTCCCCCGTG	-14	ATGACAATCG	-141	1,1	6.12	10.10	s	1445	Rmet_4016	*pcaD*	Op1126f_1	MET-XEN-Benz	
2037	TSS_952323+3	117	19	0,1	-17	1	-41	TTGATGCGCG	-15	GCCAAACTTG	-139	1,1	7.40	2.40	*(w)-sba*	89	Rmet_4357	*0*	Op1242f_2	NA	
2038	TSS_579272-3	117	64	0,1	-14	1	-35	TCAAGGAAGC	-12	ACTACACTTA	-140	4,1	8.84	8.20	s	28	Rmet_4007	*0*	Op1125r_3	NA	
2039	TSS_1265946+2	117	37	0,1	-15	1	-46	GTCAGGATGT	-13	CGTACAATCC	-141	4,1	9.53	-12.10	*no*	121	Rmet_1157	*0*	Op0333f_1	NA	
2040	TSS_390572-3	117	11	0,1	-15	1	-36	TCAGCCAGTT	-13	GCTATCTTGT	-141	5,1	8.07	8.40	s	41	Rmet_3847	*0*	Op1069r_1	NA	
2041	TSS_3352577+2	116	13	0,1	-16	1	-35	TGGATTCGCT	-14	GCCACAATGC	-140	1,1	7.74	10.10	s	51	Rmet_3094	*ffh*	Op0871f_1	GIP-PTL-Exp	
2042	TSS_3152325-2	116	21	0,1	-15	1	-33	TGGCGTGCCC	-13	TGCATAATAT	-141	1,1	6.65	7.10	s	171	Rmet_2895	*icd*	Op0814r_1	MET-CAH-TCA	
2043	TSS_1516833-2	116	5	0,1	-15	1	-37	GCGACAGTCT	-13	GATACAATGC	-139	5,1	10.17	7.90	s	42	Rmet_1404	*yheS*	Op0396r_1	GIP-TL-Ass	
2044	TSS_3708300-2	116	6	0,1	-15	1	-40	TTCCGAGGGC	-13	GTCATAATGC	-134	1,1	9.00	2.90	*(w)-sba*	23	Rmet_3429	*yraO*	Op0950r_1	MET-GLYC-LPS	
2045	TSS_790239+3	116	7	0,3	-1	0	-46	CCGGCACTCT	-25	TTTCGCATCT	-145	5,1	2.34	-3.70	*no*	54	Rmet_4206	*0*	Op1192f_1	NA	
2046	TSS_1292329+3	116	24	0,1	-17	1	-42	TGGACGAGGT	-15	TTTATAATGC	-136	1,1	10.75	2.90	*(w)-sba*	5431	Rmet_4665	*0*	Op1350f_1	NA	
2047	TSS_1795535-3	116	19	0,1	-16	1	-35	TTGTGTCGGT	-14	TGAAGAATAT	-141	1,1	5.31	10.60	s	21	Rmet_5103	*0*	Op1503r_1	NA	
2048	TSS_49022+4	116	26	0,1	-16	1	-36	TCGCCCTGTG	-14	GGCACAATTC	-140	1,1	8.29	10.90	s	18	Rmet_6008	*0*	Op1833f_4	NA	
2049	TSS_418434+2	116	19	0,1	-16	1	-36	CCAGCCGGGT	-14	GCGACAATCG	-138	5,1	9.66	6.40	s	19	Rmet_0391	*atmA*	Op0107f_1	EIP-TRA-ABC	
2050	TSS_2945870-2	116	11	0,1	-16	1	-39	TGCTGAGGGG	-14	GATAGAATCC	-138	1,1	7.03	4.40	m	54	Rmet_2712	*0*	Op0768r_2	NA	
2051	TSS_3874683-2	116	29	0,1	-15	1	-42	TCGAAGCTCT	-13	GTCAAACTTG	-140	1,1	6.56	-3.10	*no*	370	Rmet_3570	*0*	Op0990r_1	NA	
2052	TSS_1049942+2	115	25	0,1	-17	1	-36	TTGACCTGTC	-15	CTTATAGTTC	-141	1,1	9.10	13.80	s	193	Rmet_0966	*0*	Op0269f_1	NA	
2053	TSS_1420437+2	115	23	0,1	-16	1	-44	TTGCCCATCG	-14	TATAGAATAC	-139	1,1	9.31	-2.60	*no*	4987	Rmet_1309	*tnpA*	Op0375f_1	NA	
2054	TSS_1100850-2	115	24	0,1	-16	1	-45	TGGCGCCAGG	-14	CATATGCTGC	-140	1,1	6.37	-8.10	*no*	101	Rmet_1010	*0*	Op0290r_1	NA	
2055	TSS_1914808-2	115	19	0,1	-14	1	-43	GCTGCCGTGC	-12	GCTACAGTGC	-139	5,1	9.84	-9.60	*no*	22	Rmet_1762	*yoaE*	Op0504r_1	NA	
2056	TSS_952205+3	115	30	0,1	-17	1	-37	GCGGACGGTC	-15	GCGACAATCG	-138	5,1	9.09	5.90	m	207	Rmet_4357	*0*	Op1242f_2	NA	
2057	TSS_2940862+2	115	13	0,1	-18	1	-37	TTGACCATAC	-16	CCTACATTAC	-143	1,1	8.27	11.80	s	30	Rmet_2708	*0*	Op0767f_1	NA	
2058	TSS_540389-2	115	31	0,1	-16	1	-44	TTCACGTCAT	-14	GTTAACATGA	-140	1,1	8.15	-4.10	*no*	9710	Rmet_0500	*0*	Op0134r_1	NA	
2059	TSS_2184144-2	115	27	0,1	-22	0	-40	TGGCAAGGCG	-20	GCGAGAATGT	-140	1,1	7.27	5.30	*m*	333	Rmet_2018	*0*	Op0588r_1	NA	
2060	TSS_3080205-2	115	10	0,1	-15	1	-37	GTCGGAACAC	-13	TCTACAATCC	-138	4,1	10.96	5.90	m	2505	Rmet_2828	*0*	Op0796r_1	NA	
2061	TSS_140196+4	115	13	0,1	-16	1	-34	TCGCCGCTGG	-14	GGGATAATCA	-141	1,1	7.46	9.10	s	1755	Rmet_6378	*0*	Op2114f_1	NA	
2062	TSS_902551-3	115	20	0,1	-16	1	-34	TTGAATTTTG	-14	TCTAAAATAC	-142	1,1	10.02	10.60	s	28	Rmet_4307	*0*	Op1227r_1	NA	
2063	TSS_3778605-2	114	8	0,1	-15	1	-45	TTGCACTAGG	-13	AGTAAGATCG	-141	1,1	8.80	-7.60	*no*	85	Rmet_3489	*putA*	Op0970r_3	NA	
2064	TSS_2406509+3	114	48	0,1	-16	1	-49	TTGACGAGTG	-14	CCTAGACTGA	-138	1,1	8.65	-12.60	*no*	55	Rmet_5645	*adh*	Op1684f_1	NA	
2065	TSS_768665-3	114	22	0,1	-16	1	-35	TTGATGCGCG	-14	GTCAGCATGT	-141	1,1	6.28	10.60	s	496	Rmet_4184	*0*	Op1181r_1	NA	

2066	TSS_2182993+2	114	26	0,1	-20	0	-46	TTCCCGCCAT	-18	CTAACAATTC	-145	1,1	5.84	-5.10	no	230	Rmet_6502	0	Op2024f_1	NA
2067	TSS_645369-2	114	12	0,2	-15	1	-34	TGGCGCCGGA	-13	AATAGAATCG	-136	1,1	7.65	9.10	s	72	Rmet_0597	rpoO	Op0168r_1	GIP-TK-RNAP
2068	TSS_20424+4	114	15	0,2	-24	0	-42	CGGCGATATC	-14	GAGAACATGA	-147	1,1	0.89	-5.10	no	334	Rmet_6044	0	Op1843f_1	NA
2069	TSS_1400853+2	114	20	0,1	-18	1	-46	TTGAGGCCAC	-16	TGTACAATCA	-138	1,1	9.83	-4.60	no	14701	Rmet_1299	0	Op0371f_1	NA
2070	TSS_319371-2	114	26	0,1	-27	0	-48	ATCCGCAGCG	-25	TCGATCGTCA	-145	2,1	4.02	-7.10	no	804	Rmet_0302	rpoX	Op0086r_2	GIP-TL-Ass
2071	TSS_3606902-2	114	4	0,1	-16	1	-42	GCTGCCTCAG	-14	GGCACACTAG	-144	5,1	7.37	-6.60	no	220	Rmet_3334	rpoB	Op0930r_4	GIP-TK-RNAP
2072	TSS_2502789+2	113	19	0,5	-18	1	-35	TTGTTTTTTA	-12	TATCTGCTCA	-147	1,1	0.87	8.20	s	122	Rmet_2280	hemP	Op0651f_3	NA
2073	TSS_2740506+2	113	6	0,1	-16	1	-36	CCGGCAAGAT	-14	AGTAAACTCG	-136	5,1	10.95	8.90	s	76	Rmet_2522	0	Op0697f_1	NA
2074	TSS_2457835-2	113	12	0,1	-16	1	-41	TTCTTACATC	-14	GATAGACTTC	-144	1,1	6.87	0.90	(w)-sba	30	Rmet_2242	0	Op0642r_1	NA
2075	TSS_2509816-2	113	7	0,1	-14	1	-33	TGGCCTTGTC	-12	GCAAGAATCG	-140	1,1	6.41	8.10	s	323	Rmet_2287	0	Op0654r_1	NA
2076	TSS_3773080-2	113	9	0,1	-14	1	-36	TTCCCGGGTG	-12	CGCATAATTC	-140	1,1	7.86	9.90	s	30	Rmet_3487	0	Op0970r_4	NA
2077	TSS_411078+3	113	15	0,1	-23	0	-38	TTCAAGACAG	-12	TGGAATCTGC	-143	1,1	4.17	2.90	w	2751	Rmet_3870	0	Op1078f_1	NA
2078	TSS_1422249+3	113	12	0,1	-16	1	-49	CCGGCCTCCC	-14	AGTAAGATGC	-139	5,1	8.77	-18.10	no	3650	Rmet_4782	0	Op1392f_1	NA
2079	TSS_1002110-3	113	13	0,1	-16	1	-44	TCCGGAGAAC	-14	AGTACAGTCC	-139	4,1	7.51	-6.60	no	46	Rmet_4406	0	Op1255r_1	NA
2080	TSS_1761593-3	113	32	0,1	-15	1	-49	TTGAAGGGCT	-22	TCCATATTTG	-143	1,1	7.42	-5.60	no	670	Rmet_5072	0	Op1493r_1	MET-AA-Met
2081	TSS_3507399+2	113	30	0,1	-29	0	-37	TGCAGTCTCT	-14	GTTAATCTGG	-145	1,1	5.12	6.40	s	19	Rmet_3231	mscL	Op0915f_1	EIP-TRA-Pores
2082	TSS_474957+3	113	27	0,3	-23	0	-46	TTGTAGTCAA	-13	CTGAGTGTTT	-147	1,1	2.21	-11.60	no	224	Rmet_R0078	0	Op1098f_1	NA
2083	TSS_1561963+3	113	16	0,1	-16	1	-40	TTCTCTTTGG	-14	CCTATGCTGT	-142	1,1	6.04	2.90	(w)-sba	52	Rmet_4898	0	Op1434f_2	NA
2084	TSS_3608646-2	112	10	0,1	-16	1	-37	TTCAGTTCCG	-14	GGTATCATCC	-138	1,1	8.27	9.90	s	571	Rmet_3336	rplJ	Op0930r_4	GIP-TL-Ribo
2085	TSS_1764870+2	112	15	0,1	-17	1	-49	TGCACGATAG	-15	GGAACAATCG	-143	1,1	5.93	-14.60	no	20	Rmet_1636	0	Op0459f_1	MET-CAH-But
2086	TSS_226701-2	112	21	0,1	-15	1	-45	GCCACGCCGG	-13	GTTATACTGG	-139	4,1	8.74	-10.60	no	63	Rmet_0213	crp1	Op0064r_1	NA
2087	TSS_2091583-2	112	23	0,2	-8	0	-34	TTGAACTGCA	-6	AATACCGTTT	-144	1,1	5.97	4.20	(m)-ba	999	Rmet_1923	0	Op0560r_1	GIP-PTL-Fold
2088	TSS_488413+2	112	30	0,1	-16	1	-38	TGCAGCGCCG	-14	GGCATAATCC	-136	1,1	6.85	6.40	s	54	Rmet_0460	0	Op0121f_2	MET-CAH-But
2089	TSS_3220416+2	111	17	0,1	-18	1	-37	TGGAAGGTTC	-16	CAGAGGCTTC	-141	1,1	3.37	7.30	s	333	Rmet_2963	pntAa	Op0835f_1	MET-COF-NicAm
2090	TSS_2667709-2	111	12	0,1	-16	1	-40	GGCGAGGTCC	-14	GCCATTATGC	-143	4,1	6.75	-2.60	no	125	Rmet_2456	0	Op0686r_1	NA
2091	TSS_2268083+2	111	33	0,1	-17	1	-36	TTGCGCGACC	-15	TTCATCCTGA	-141	1,1	5.62	9.80	s	117	Rmet_2089	talB	Op0605f_1	MET-CAH-PP_KDPG
2092	TSS_2664840+2	111	28	0,1	-16	1	-47	GCACAAACGT	-14	TATAAGATGC	-140	4,1	10.51	-15.60	no	26	Rmet_2453	0	Op0685f_2	NA
2093	TSS_3447866+2	111	11	0,1	-18	1	-36	GTCCACAAAC	-16	CCTACAATCT	-140	4,1	9.98	6.30	m	23	Rmet_3174	0	Op0891f_1	NA
2094	TSS_2713344-2	111	9	0,1	-12	1	-37	TGGCAAAACT	-10	GTCCTACTGG	-145	1,1	3.15	2.90	w	69	Rmet_2498	0	Op0692r_2	NA
2095	TSS_449402-2	111	8	0,1	-16	1	-37	CGGCCATTAA	-14	GCTACACTTG	-139	1,1	4.82	6.90	s	26	Rmet_0424	ruvA	Op0116r_3	MET-LIP-GlycPLip
2096	TSS_851844-2	111	31	0,1	-15	1	-33	TCGTTCTGTC	-13	GCCAGAATGC	-142	1,1	6.42	6.10	m	997	Rmet_0774	phnD	Op0220r_1	EIP-TRA-ABC
2097	TSS_2874075-2	111	41	0,1	-17	1	-36	TTGCCGACTG	-15	CTTATCCTGC	-141	1,1	7.95	11.80	s	0	Rmet_2637	0	Op0742r_1	NA
2098	TSS_3046265-2	111	12	0,1	-17	1	-35	TTGGCGGCGG	-15	TTCATAATTA	-141	2,1	11.40	10.60	s	44	Rmet_2798	matR	Op0786r_2	NA
2099	TSS_3157398+2	110	20	0,3	-1	0	-31	AACCTTCGCG	-11	GTCAAACGGG	-143	3,0	3.96	-3.40	no	815	Rmet_6565	0	Op0821f_1	NA
2100	TSS_3630992+2	110	21	0,1	-2	0	-22	TTCCCTTGTC	0	GACACATTGT	-143	1,1	5.78	-3.30	no	560	Rmet_3355	0	Op0935f_2	NA
2101	TSS_823201-2	110	16	0,1	-14	1	-43	TCTGACCGCC	-12	CGTACAATGT	-136	5,1	10.69	-6.60	no	45	Rmet_0744	0	Op0210r_1	NA
2102	TSS_2182496-3	110	6	0,1	-15	1	-34	ATTCGTTGCC	-13	CGCACCATAA	-140	2,1	8.66	4.10	m	42	Rmet_5435	0	Op1617r_2	NA
2103	TSS_75078+4	110	27	0,1	-16	1	-41	GCGGCACCCC	-14	GCGATAATCG	-139	5,1	10.49	-0.10	no	97	Rmet_5983	czcI	Op1820f_1	NA
2104	TSS_3077672-2	110	6	0,1	-16	1	-35	TTGCTTGAAT	-14	TTCATAATAT	-144	1,1	7.82	11.60	s	6782	Rmet_2822	0	Op0794r_1	NA
2105	TSS_60417+3	110	25	0,1	-17	1	-39	TTCCCCACGT	-15	GGTACGCTTT	-138	1,1	7.58	4.90	m	448	Rmet_5864	cynS	Op1774f_1	MET-EN-Nitrog
2106	TSS_118277+3	110	44	0,5	-27	0	-49	TCGGAAGTCT	-23	CGTATTTTGC	-142	5,1	7.76	-6.10	no	129	Rmet_5917	0	Op1796f_2	NA
2107	TSS_2772519+2	110	18	0,1	-16	1	-38	CCTACCCGGC	-14	GGCAGAATGG	-144	5,1	7.81	3.40	m	907	Rmet_2548	0	Op0707f_2	NA
2108	TSS_3618485+2	110	15	0,1	-17	1	-37	TTCCAAAGCC	-15	GCCAAAATCA	-142	1,1	7.99	9.90	s	98	Rmet_3342	0	Op0931f_3	NA
2109	TSS_3590540-2	110	6	0,2	-24	0	-41	TTTCTGCGCC	-14	ATTAAAGTGG	-143	2,1	10.59	-0.10	no	2180	Rmet_3324	tuf	Op0928r_2	NA
2110	TSS_2297308+3	110	16	0,1	-16	1	-41	CCGGCAAGAC	-14	TAGATAATAA	-142	5,1	9.32	-0.10	no	214	Rmet_5550	0	Op1648f_3	NA
2111	TSS_2636014-2	109	18	0,1	-11	1	-42	TGGCCAGGCG	-9	CACAGAATGC	-140	1,1	6.82	-5.10	no	96	Rmet_2425	rpoE	Op0680r_3	GIP-TK-RNAP
2112	TSS_3245188-2	109	19	0,1	-27	0	-41	GCAAGGATGC	-13	ACTAACCTGT	-144	4,1	8.10	-5.60	no	20	Rmet_6571	0	Op0842r_3	NA
2113	TSS_153056+3	109	15	0,1	-16	1	-42	TGGAAGGGCC	-14	GGTACTCTGG	-142	1,1	7.26	-2.10	no	572	Rmet_R0074	0	Op1006f_1	NA
2114	TSS_6005-5	109	28	0,1	-15	1	-35	TTGCGCCACG	-13	TCGAAAATCG	-139	1,1	7.42	11.20	s	20105	Rmet_6301	trbF	Op1917r_1	NA
2115	TSS_769260-2	109	19	0,1	-15	1	-35	TCAATCGTT	-13	TGTACACTTG	-139	1,1	8.24	10.70	s	511	Rmet_0696	0	Op0196r_2	NA
2116	TSS_3867920-2	109	20	0,1	-15	1	-40	GCCCCAAAGC	-13	CGTATACTGA	-136	4,1	10.16	-0.60	no	369	Rmet_3565	0	Op0988r_1	NA
2117	TSS_2372834+3	109	12	0,1	-10	1	-46	TTGCCAAAGG	-8	GTCATACTGC	-140	1,1	9.57	-12.60	no	1055	Rmet_5617	0	Op1676f_1	MET-LIP-Ster
2118	TSS_2533638-3	109	9	0,1	-16	1	-34	GGCCGAACGC	-14	CATAGAATGC	-140	4,1	10.25	4.60	m	81	Rmet_5773	0	Op1735r_2	NA
2119	TSS_3692+5	109	25	0,1	-17	1	-39	TCGACCAGGC	-15	AGTAAGCTGC	-141	1,1	6.78	5.90	m	458	Rmet_6391	0	Op1918f_4	NA
2120	TSS_401767+2	109	29	0,1	-16	1	-42	TTGCCGATGC	-14	GGTAGGGTTG	-139	1,1	7.62	-0.60	no	308	Rmet_0376	0	Op0103f_5	NA
2121	TSS_554513-2	109	38	0,1	-15	1	-41	GTCACGGAGT	-13	GCTACAATCG	-138	4,1	9.89	-1.10	no	32	Rmet_0517	0	Op0140r_2	NA
2122	TSS_2522332+3	109	4	0,1	-17	1	-40	TTGTGTAAAT	-15	GTTAGAATGC	-142	1,1	9.97	5.40	(m)-sba	21	Rmet_5761	0	Op1728f_1	NA
2123	TSS_1615198-3	109	20	0,1	-15	1	-35	TTGATGAAAC	-13	CATATAATGC	-139	1,1	10.20	14.20	s	24	Rmet_4949	0	Op1453r_1	MET-LIP-FASyn
2124	TSS_251645+2	108	23	0,1	-16	1	-37	TGGTGCGAAC	-14	CGGATAATAC	-142	1,1	5.87	8.90	s	656	Rmet_0236	rhtB	Op0069f_2	EIP-TRA-Ion
2125	TSS_438896+2	108	4	0,1	-15	1	-34	GCAGCCGGAT	-13	GCTACAGTTC	-139	5,1	10.20	4.60	m	223	Rmet_0413	0	Op0113f_2	NA
2126	TSS_1428273+3	108	21	0,1	-16	1	-35	TGGCTCGTGT	-14	CTTATAGTCG	-138	1,1	6.79	10.10	s	61	Rmet_4784	frmA	Op1392f_2	MET-EN-Methane

2127	TSS_92278+2	108	22	0,1	-16	1	-46	GGAGGAAAGT	-14	GCGACAATTA	-142	4,1	9.35	-13.60	no	958	Rmet_0087	bhmT	Op0025f_1	MET-AA-Met
2128	TSS_2312516-2	108	8	0,1	-14	1	-34	TTCACGGATG	-12	GATAGCATCT	-139	1,1	7.09	10.70	s	51	Rmet_2129	dnaX	Op0614r_2	GIP-REP-DNA_Pol
2129	TSS_2839350-2	108	19	0,1	0	0	-35	TTGCATTTCG	-14	ACGACGATTC	-141	1,1	6.22	9.60	s	319	Rmet_2608	0	Op0730r_2	NA
2130	TSS_3835948-2	108	18	0,1	-9	0	-33	TGGTTTACAT	-7	CAGATAGTAG	-148	1,1	2.66	3.70	(m)-ba	349	Rmet_3537	0	Op0978r_1	NA
2131	TSS_1269698+3	108	35	0,1	-15	1	-45	CCTGCCGCAG	-13	GCTATCGTCA	-142	5,1	7.99	-12.60	no	30	Rmet_4639	0	Op1340f_1	NA
2132	TSS_1111789-3	108	13	0,1	-16	1	-38	TCGAACGCTT	-14	GGTAGAGTAA	-143	1,1	6.11	6.90	s	47	Rmet_4508	0	Op1287r_1	NA
2133	TSS_2421323-3	108	20	0,1	-15	1	-47	TTGACCCTAA	-13	GCTACGCTGC	-139	1,1	8.82	-10.60	no	37	Rmet_5660	0	Op1691r_1	NA
2134	TSS_563861+2	107	33	0,1	-16	1	-42	TTCCGCTTGG	-14	TCTAGAATGC	-142	2,1	11.92	-1.10	no	31	Rmet_0528	0	Op0145f_1	NA
2135	TSS_3466382-2	107	20	0,1	-10	1	-33	TGGAACTCGG	-8	CAAAGAGTCG	-142	1,1	2.90	4.70	(m)-ba	323	Rmet_3190	dsbC	Op0896r_1	GIP-PTL-Fold
2136	TSS_60099-3	107	28	0,1	-15	1	-39	GCAACCGCGC	-13	GCTAGAGTGA	-141	4,1	8.62	0.40	w	32	Rmet_6610	0	Op1773r_1	NA
2137	TSS_107355-4	107	15	0,1	-11	1	-36	CCGGAACTTT	-9	CAGATGATGC	-142	5,1	6.75	2.90	(w)-ba	57	Rmet_5952	tnpA	Op1810r_2	NA
2138	TSS_502958-2	107	9	0,1	-16	1	-45	GCAACCAACC	-14	CCTACTATTA	-141	4,1	8.06	-10.60	no	7566	Rmet_0465	0	Op0122r_1	EIP-SIG-2Comp
2139	TSS_2709866-2	107	16	0,1	-16	1	-46	TTGTGCCGGT	-14	AGTACAATCG	-139	1,1	9.36	-7.60	no	8517	Rmet_2488	0	Op0688r_1	NA
2140	TSS_2962897-2	107	19	0,1	-16	1	-34	TTTCTGTGCT	-14	GCTATATTAA	-142	2,1	10.95	8.10	s	611	Rmet_2726	0	Op0770r_1	NA
2141	TSS_449202+3	107	31	0,1	-15	1	-33	TCCACGTTCG	-13	ACTATGATGA	-144	1,1	6.20	7.60	s	121	Rmet_3904	yeaG	Op1092f_1	NA
2142	TSS_1491437+2	107	12	0,1	-17	1	-36	GGCCGAGTGT	-15	TCTAAGATGA	-142	4,1	7.82	5.80	m	28	Rmet_1382	rraA	Op0385f_4	NA
2143	TSS_1600904+2	107	37	0,1	-15	1	-40	TGGTAGCCGG	-13	GGCATAATTA	-142	1,1	7.30	1.90	(w)-sba	871	Rmet_1480	0	Op0417f_1	NA
2144	TSS_3037700+2	107	9	0,5	-14	1	-33	CAACTTCGTG	-13	CGTCTTAACC	-147	3,0	3.60	-1.40	no	79	Rmet_2790	0	Op0785f_1	NA
2145	TSS_3380829+2	107	29	0,1	-16	1	-36	TGGATTGGCG	-14	TTTATCATGT	-139	1,1	7.05	11.90	s	1191	Rmet_3120	0	Op0877f_1	NA
2146	TSS_3920116-2	107	18	0,1	-15	1	-40	TGGCCCGGGT	-13	GTTAAACTGG	-137	1,1	9.10	1.90	(w)-sba	41	Rmet_3607	0	Op1004r_2	NA
2147	TSS_41404+3	107	18	0,1	-15	1	-39	CCTGCACGAT	-13	GGTAGACTCC	-139	5,1	11.19	0.40	w	31	Rmet_5848	0	Op1768f_1	NA
2148	TSS_682053+3	107	23	0,1	-15	1	-35	TTGCCGGGTG	-13	CGTAAAATCG	-139	1,1	10.13	13.20	s	3	Rmet_4110	0	Op1158f_1	NA
2149	TSS_486040-3	107	17	0,1	-16	1	-39	TCGCTGACGT	-14	GCCATGATGG	-139	1,1	6.65	3.90	m	31	Rmet_3926	0	Op1099r_3	MET-AA-ArgPro
2150	TSS_50694-4	107	16	0,1	-25	0	-43	TATAGGTTGT	-23	TGGAAATTGG	-145	2,1	4.15	-0.20	no	203	Rmet_6007	0	Op1832r_1	NA
2151	TSS_1956425+2	106	9	0,1	-17	1	-49	TTGCGGTGGC	-15	TATAGAATCG	-137	1,1	9.79	-12.60	no	218	Rmet_1804	0	Op0523f_2	NA
2152	TSS_729677+3	106	13	0,1	-16	1	-44	GGAAAAATCC	-14	AGCATAATGC	-144	4,1	10.10	-7.60	no	148	Rmet_4154	tnpA	Op1170f_1	NA
2153	TSS_1226181+3	106	16	0,1	-25	0	-36	CCAGCCTCAT	-14	ACTATGCTGG	-142	5,1	7.45	6.40	s	4741	Rmet_4606	metY	Op1326f_1	MET-AA-Met
2154	TSS_63222-4	106	19	0,1	-16	1	-42	TACCGAGCCA	-14	ACTAGACTAC	-145	1,1	2.16	-2.60	no	456	Rmet_5996	0	Op1828r_1	NA
2155	TSS_1894907+2	106	24	0,1	-17	1	-47	CCTGCCAGCC	-15	GCTACGCTAG	-139	5,1	9.22	-15.60	no	5895	Rmet_1751	agrR	Op0499f_1	EIP-SIG-2Comp
2156	TSS_1012294+2	106	41	0,1	-23	0	-49	TTCCAGAGGG	-21	GGCAACATTG	-141	1,1	6.68	-10.10	no	252	Rmet_0927	nuoA	Op0267f_3	MET-EN-OxPhos
2157	TSS_1630951+2	106	63	0,1	-16	1	-34	TTCTTTTATT	-14	ATTAATGTTC	-150	1,1	3.39	6.10	m	29	Rmet_1509	cbbS(del	Op0425f_1	NA
2158	TSS_3771048+2	106	2	0,1	-5	0	-34	TTGCAACTTT	-3	GCCATCTTCG	-146	1,1	6.47	1.20	(w)-ba	21123	Rmet_3502	0	Op0971f_1	NA
2159	TSS_2398645-2	106	20	0,1	-16	1	-34	TTGTTATCTC	-14	CCCAACTTGT	-143	1,1	3.54	7.60	s	396	Rmet_2188	ftsH	Op0628r_2	GIP-PTL-Fold
2160	TSS_3747626-2	106	24	0,1	-4	0	-35	TTGACTTCGC	-14	GCGATCGTCG	-140	1,1	5.59	11.60	s	42	Rmet_3467	0	Op0964r_2	NA
2161	TSS_1448645+3	106	15	0,1	-16	1	-35	TTGATCCTTT	-14	GTCAAACTGC	-141	1,1	7.85	10.60	s	240	Rmet_4799	0	Op1396f_5	NA
2162	TSS_2531589-3	106	14	0,1	-18	1	-44	TTGTGCCGAC	-16	TGCAGTCTAC	-144	1,1	3.77	-4.60	no	70	Rmet_6767	0	Op1733r_1	NA
2163	TSS_1506821+3	105	13	0,1	-16	1	-38	TACAACACGG	-14	CGTATATTGC	-143	1,1	3.71	6.40	s	58	Rmet_4849	0	Op1414f_1	NA
2164	TSS_2533108-3	105	39	0,1	-12	1	-37	TTGTCGAAAG	-10	GATAACATTT	-139	1,1	8.32	6.40	s	38	Rmet_5772	0	Op1735r_2	NA
2165	TSS_2734774-2	105	22	0,1	-15	1	-41	CCGCGTGCGT	-13	CGTACCATGT	-143	1,1	2.01	-4.10	no	47	Rmet_2518	0	Op0696r_1	EIP-TRA-Ion
2166	TSS_491742+3	105	25	0,1	-16	1	-48	TTGACTTGGC	-14	GCTATCATTT	-139	1,1	10.39	-9.60	no	76	Rmet_3934	0	Op1100f_1	EIP-SIG-2Comp
2167	TSS_45072+4	105	16	0,1	-17	1	-39	GCTGCCCGGG	-15	GGTACGCTGT	-140	5,1	9.58	0.40	w	161	Rmet_6013	0	Op1835f_2	NA
2168	TSS_2904414+2	105	27	0,1	-16	1	-46	TTGCAGGTTC	-14	CAGATAATTC	-143	1,1	8.25	-7.60	no	8468	Rmet_2680	glyA	Op0757f_1	MET-EN-Methane
2169	TSS_477606+3	104	11	0,1	-16	1	-42	TGGAAGGGCC	-14	GGTACTCTGG	-142	1,1	7.26	-2.10	no	572	Rmet_R0079	0	Op1098f_1	NA
2170	TSS_1922731+3	104	15	0,1	-16	1	-36	TTGTGCGCGT	-14	TGTAGAGTGG	-139	1,1	7.10	11.40	s	2303	Rmet_5229	0	Op1542f_1	NA
2171	TSS_2164415+2	104	34	0,1	-15	1	-38	TTGAATCTCA	-13	CTTACAATAA	-142	1,1	8.78	8.40	s	7	Rmet_1992	tnpA	Op0583f_1	GIP-REP-Recomb
2172	TSS_2836030+2	104	19	0,1	-18	1	-34	TTCTGCTGTT	-14	GATACTCTTG	-141	1,1	5.82	6.10	m	2396	Rmet_6542	0	Op2038f_1	NA
2173	TSS_3010833+2	104	4	0,1	-12	1	-36	TTGAAACCAA	-10	CATAGAGTTC	-142	1,1	7.48	9.40	s	329	Rmet_2769	metG	Op0777f_1	MET-AA-Met
2174	TSS_1006714-2	104	18	0,1	-18	1	-34	TTGTTGATAT	-13	ATGATGATTT	-143	1,1	5.58	9.60	s	1644	Rmet_0919	0	Op0264r_1	NA
2175	TSS_1780392-2	104	11	0,1	-16	1	-38	TTGTGATTTA	-14	GCTAGAATTC	-139	1,1	10.26	9.40	s	27	Rmet_1645	polB	Op0462r_1	GIP-REP-DNA_Pol
2176	TSS_2330109-2	104	30	0,1	-28	0	-29	GGAGCGAAGC	-7	CCGACAAAGG	-147	4,1	2.89	-1.80	no	0	Rmet_R0030	0	Op0616r_2	NA
2177	TSS_65600-4	104	53	0,1	-15	1	-33	TTCTACTGAG	-13	GCGAAAATTG	-141	1,1	6.83	6.10	m	0	Rmet_5992	merP	Op1826r_2	EIP-TRA-Other
2178	TSS_424527+2	104	8	0,1	-17	1	-45	TTGCGCGCCG	-15	GTGATAATCC	-140	1,1	8.49	-4.60	no	1078	Rmet_0398	gdhA	Op0109f_3	MET-EN-Nitrog
2179	TSS_1739416-2	104	10	0,1	-16	1	-41	TGGACGAGGT	-14	TTTATAATGC	-136	1,1	10.75	3.90	(m)-sba	795	Rmet_6458	0	Op2011r_1	NA
2180	TSS_3072330-2	104	19	0,1	-15	1	-33	TCGAGAGCCA	-13	AGTAAACTGA	-141	1,1	6.97	8.10	s	1440	Rmet_2822	0	Op0794r_1	NA
2181	TSS_3431865-2	104	25	0,1	-16	1	-35	TTGTTGGGGA	-14	AGTAACGTAG	-147	1,1	4.32	9.60	s	736	Rmet_R0053	0	Op0886r_1	NA
2182	TSS_1224771+3	104	10	0,1	-17	1	-48	TTTCGTCCGG	-15	GATAGAATGG	-135	2,1	13.22	-12.10	no	51	Rmet_4603	0	Op1324f_2	NA
2183	TSS_1419970+3	104	19	0,1	-16	1	-35	TAGCACAAAG	-14	CCTACACTCC	-139	1,1	5.04	9.10	s	39	Rmet_4775	0	Op1388f_2	EIP-TRA-ABC
2184	TSS_1417635+2	103	24	0,1	-18	1	-46	TTGAGGCCAC	-16	TGTACAATCA	-138	1,1	9.83	-4.60	no	977	Rmet_1304	0	Op0375f_1	NA
2185	TSS_888869+3	103	22	0,1	-17	1	-35	GGCGAAATCC	-15	CCTATATTTC	-143	4,1	8.51	5.60	m	23	Rmet_4296	0	Op1222f_1	NA
2186	TSS_44119+4	103	19	0,1	-16	1	-41	TCGCCAACAT	-14	GCTATGCTTA	-139	1,1	7.73	1.90	(w)-sba	1114	Rmet_6013	0	Op1835f_2	NA
2187	TSS_2245552-2	103	18	0,1	-16	1	-34	TTCTGGCGTT	-14	ATTATAATCC	-136	1,1	8.21	9.10	s	1	Rmet_2069	hrpA	Op0600r_1	GIP-REP-Recomb

2188	TSS_3625517-2	103	33	0,1	-16	1	-41	TGGACGAGGT	-14	TTTATAATGC	-136	1,1	10.75	3.90	(m)-sba	94	Rmet_3347	0	Op0932r_2	NA
2189	TSS_807244+3	103	15	0,1	-12	1	-34	TTGACCGCGT	-10	AGTATGGTCG	-142	1,1	7.24	10.20	s	45	Rmet_4222	0	Op1198f_3	NA
2190	TSS_2175934+3	103	10	0,1	-29	0	-47	GTAAGCATCC	-27	CCTAAGCTTT	-146	4,1	6.62	-5.70	no	258	Rmet_5430	0	Op1616f_1	MET-GLYC-LPS
2191	TSS_146157-4	103	11	0,1	-15	1	-39	TGCAAATGCT	-13	GCTAGAATGT	-139	1,1	9.01	4.40	m	240	Rmet_6151	0	Op1880r_2	NA
2192	TSS_2329877-2	103	40	0,1	-16	1	-37	TCGTGCTGCA	-14	ATTATAATTT	-144	1,1	7.80	9.90	s	180	Rmet_R0028	0	Op0616r_2	NA
2193	TSS_2696792-2	103	18	0,1	-16	1	-46	TTGATGGTTC	-14	TATAAACTGC	-140	1,1	8.72	-7.60	no	66	Rmet_2482	0	Op0688r_2	NA
2194	TSS_3831082-2	103	26	0,1	-15	1	-36	TGGCCATATT	-13	CATATAATCT	-142	1,1	9.83	12.90	s	34	Rmet_3532	0	Op0976r_1	NA
2195	TSS_1259664-3	103	18	0,4	-18	1	-34	TTGCAATCGC	-13	AAGAAGATCG	-138	1,1	6.29	9.60	s	2681	Rmet_4627	0	Op1333r_1	EIP-SIG-2Comp
2196	TSS_1636959-3	103	13	0,1	-15	1	-41	TTCCCTAGGA	-13	TCTATTATCT	-143	1,1	7.74	0.90	(w)-sba	72	Rmet_4965	0	Op1457r_1	EIP-SIG-2Comp
2197	TSS_1889749-3	103	20	0,1	-17	1	-43	TTCCTCTCTC	-15	TGTATACTCG	-140	1,1	8.06	-2.10	no	85	Rmet_5194	0	Op1531r_1	NA
2198	TSS_16319-5	103	6	0,1	-14	1	-33	TCCACACGAT	-12	GCCAAAATTG	-141	1,1	7.18	8.60	s	8952	Rmet_6320	int	Op1919r_1	NA
2199	TSS_2822033+2	102	20	0,1	-17	1	-48	TGGAAAATCA	-15	GGTAGACTGA	-139	1,1	8.70	-11.10	no	64	Rmet_2595	0	Op0727f_2	NA
2200	TSS_3408437+2	102	36	0,1	-16	1	-45	TTCACCCCAT	-14	ACTAGAATGG	-139	1,1	8.64	-5.10	no	48	Rmet_3145	bug	Op0881f_1	NA
2201	TSS_3474396-2	102	17	0,1	-15	1	-45	TTGCAGGAAT	-16	TGCAGTATTG	-142	1,1	6.74	-5.60	no	50	Rmet_3199	0	Op0900r_1	NA
2202	TSS_118318+3	102	11	0,1	-16	1	-35	TTGCGCCACC	-14	CTTACCATGC	-138	1,1	8.26	10.60	s	88	Rmet_5917	0	Op1796f_2	NA
2203	TSS_505613-3	102	22	0,1	-15	1	-43	TCGACTCACG	-13	CTTATACTGT	-145	1,1	7.58	-2.10	no	118	Rmet_3948	0	Op1105r_1	NA
2204	TSS_2179856-3	102	13	0,1	-16	1	-35	TTCCCCGTGG	-14	CGGATAATTC	-139	1,1	7.49	11.10	s	505	Rmet_5432	0	Op1617r_2	MET-CAH-Pyr
2205	TSS_933419+2	102	19	0,1	-16	1	-44	GCGTCCGCTG	-14	GTTAGAATCG	-139	5,1	8.73	-7.10	no	2291	Rmet_0855	0	Op0245f_1	NA
2206	TSS_2781243-2	102	26	0,1	-14	1	-37	TTGAATCTCA	-12	CTTACAATAA	-140	1,1	8.78	9.40	s	8	Rmet_2557	tnpA	Op0708r_3	GIP-REP-Recomb
2207	TSS_1306632+3	102	16	0,1	-16	1	-38	TTGAGATGAC	-14	GCTAGAATGC	-135	1,1	11.60	10.40	s	83	Rmet_4674	0	Op1352f_1	NA
2208	TSS_1767220+3	102	17	0,1	-16	1	-47	TTGCAATGGG	-14	AAGATAATCT	-140	1,1	8.42	-8.60	no	855	Rmet_5080	0	Op1496f_1	NA
2209	TSS_1928363+3	102	7	0,1	-15	1	-36	GTCAGAGAGT	-13	GGCATACTTG	-142	4,1	8.90	7.90	s	162	Rmet_5231	0	Op1544f_1	NA
2210	TSS_2453539-3	102	33	0,1	-15	1	-37	GTAACCCAAT	-13	TCCATGATGG	-145	4,1	6.62	5.90	m	2438	Rmet_5691	0	Op1699r_1	NA
2211	TSS_41026+5	102	21	0,1	-16	1	-36	TTGCACGAAA	-14	GCTAAGCTAT	-139	1,1	7.57	10.40	s	37	Rmet_6191	bph2	Op1894f_6	NA
2212	TSS_2094149+2	102	19	0,1	-1	0	-36	TTGCCCTGTA	-15	TTGATGACTG	-139	1,1	7.15	11.80	s	3910	Rmet_1930	0	Op0563f_1	NA
2213	TSS_2869479-2	102	20	0,2	-15	1	-44	TTCCTGCTCG	-13	AATACCATAC	-139	2,1	9.34	-7.10	no	396	Rmet_6544	0	Op0738r_1	NA
2214	TSS_779958+3	102	50	0,1	-1	0	-28	TTCAGACGAG	-7	GGCGGGCTTG	-150	1,1	-1.52	0.10	no	23	Rmet_4195	cheD	Op1188f_1	DIV-MOT-Chemotax
2215	TSS_1610936-3	102	14	0,1	-14	1	-49	TCGCCGATGC	-12	GGCAACATGG	-139	1,1	6.79	-18.10	no	176	Rmet_4945	0	Op1453r_2	NA
2216	TSS_118235+5	102	4	0,1	-18	1	-37	TTGACTATGT	-16	CTCAGCCTCT	-141	1,1	5.23	9.80	s	2190	Rmet_6396	0	Op1910f_1	NA
2217	TSS_3320723+2	101	23	0,1	-16	1	-34	GTACAGGTAC	-14	GCCAGAATCA	-141	4,1	8.23	4.10	m	139	Rmet_3064	accB	Op0861f_4	MET-CAH-Pyr
2218	TSS_2330329-2	101	22	0,1	-14	1	-44	AAATCTGGTG	-24	CAGTAAATGG	-147	3,0	6.12	-5.20	no	78	Rmet_R0031	0	Op0616r_2	NA
2219	TSS_2701349-2	101	5	0,4	-17	1	-36	TGGACGTGAT	-14	GGTTGAATCC	-149	1,1	4.05	10.90	s	0	Rmet_2488	0	Op0688r_1	NA
2220	TSS_3613492-2	101	32	0,1	-15	1	-41	TGGAAGGGCC	-13	GGTACTCTGG	-142	1,1	7.26	-1.10	no	573	Rmet_R0060	0	Op0930r_1	NA
2221	TSS_74698+3	101	10	0,1	-17	1	-30	GCGGCATCCT	0	GCCACACTGG	-139	5,1	8.68	-5.30	no	94	Rmet_5878	0	Op1778f_3	NA
2222	TSS_1453616-3	101	47	0,1	-16	1	-35	TTGCGACATC	-14	GTTATAGTGC	-137	1,1	9.70	12.60	s	72	Rmet_4805	kbl	Op1397r_1	MET-AA-GlySerThr
2223	TSS_225902+4	101	24	0,1	-16	1	-30	TTGCGGATGT	-3	CTCAACCTAT	-144	1,1	4.92	-0.80	no	3064	Rmet_6071	0	Op1849f_1	NA
2224	TSS_2731746+2	101	14	0,1	-17	1	-37	TCGAGCGGGG	-15	CGTATCTACA	-139	1,1	7.24	10.90	s	31	Rmet_2516	0	Op0695f_3	EIP-TRA-ABC
2225	TSS_484013-2	101	24	0,1	-16	1	-43	GGAGCGATAC	-14	GCTATCATTG	-137	4,1	9.66	-6.60	no	35	Rmet_0455	clcB	Op0118r_1	EIP-TRA-Pores
2226	TSS_1164716-3	101	12	0,1	-15	1	-41	TTCTCCGGCG	-13	GGCAATATAG	-142	1,1	4.63	-1.10	no	3919	Rmet_4549	uvrA2	Op1305r_1	GIP-REP-Recomb
2227	TSS_901028+2	101	12	0,1	-16	1	-34	TGGAGCCGGC	-14	ACTATCATCG	-137	1,1	7.62	9.10	s	44	Rmet_0827	cvrA	Op0235f_1	NA
2228	TSS_3360989+2	101	14	0,1	-16	1	-45	TCGGCATCAT	-14	GGTATTCTGG	-140	5,1	9.01	-6.10	no	641	Rmet_3102	0	Op0873f_1	NA
2229	TSS_2723957-2	101	3	0,1	-15	1	-34	TTGATGTTAG	-13	GGTACAGTAC	-139	1,1	8.43	10.60	s	43	Rmet_2507	0	Op0694r_1	NA
2230	TSS_289615-3	101	15	0,1	-14	1	-47	TTGCACCTGG	-12	CGTAGGATTG	-139	1,1	8.31	-12.60	no	12292	Rmet_3733	flgA	Op1041r_1	DIV-MOT-Flagel
2231	TSS_134942+2	100	2	0,1	-16	1	-41	TCGAGACGGC	-14	CCTATGATCG	-140	1,1	7.90	2.90	(w)-sba	32	Rmet_0129	dehH2	Op0033f_1	MET-XEN-Hex
2232	TSS_1587046+2	100	22	0,1	-16	1	-35	TTGACAACCT	-14	CTCATACTGA	-140	1,1	8.08	13.60	s	4	Rmet_1466	0	Op0411f_1	NA
2233	TSS_1533190+3	100	23	0,1	-16	1	-35	TTGCGATATC	-14	CTCAATATCG	-142	1,1	6.60	10.60	s	99	Rmet_4871	0	Op1422f_1	NA
2234	TSS_460646-3	100	21	0,1	-15	1	-38	TTGCTAGGCT	-13	TCTAGAATCG	-138	1,1	9.80	8.40	s	38	Rmet_3912	0	Op1095r_1	MET-CAH-FruMan
2235	TSS_608999-3	100	8	0,1	-16	1	-45	TTGCAAAGGA	-14	GTCACATTAC	-140	1,1	7.36	-6.60	no	115	Rmet_4042	tniR	Op1139r_1	NA
2236	TSS_58166+2	100	22	0,1	-26	0	-40	TGGCGGCGAA	-19	AGTCTAATCA	-144	1,1	3.64	5.30	(m)-nu	320	Rmet_0051	mreB	Op0017f_1	DIV-Division
2237	TSS_1152532+2	100	19	0,1	-16	1	-45	TTCCCGTGGG	-14	TCTACAATGA	-138	1,1	9.35	-6.10	no	17	Rmet_1060	0	Op0303f_1	NA
2238	TSS_1876494+2	100	11	0,1	-17	1	-45	TTGAAATATT	-15	TTTACATTTC	-142	1,1	9.40	-3.60	no	15	Rmet_1732	0	Op0491f_1	NA
2239	TSS_2393552+2	100	12	0,1	-16	1	-28	TTGACAAGGC	0	ATGACAGTTC	-146	1,1	6.66	0.20	no	5492	Rmet_2190	0	Op0629f_1	NA
2240	TSS_3491152+2	100	22	0,1	-17	1	-47	TTGCACTCGC	-15	GCTATGATCC	-141	1,1	10.37	-8.60	no	37	Rmet_3217	0	Op0907f_1	NA
2241	TSS_136598+3	100	6	0,1	-16	1	-36	TCGACAGCGA	-14	CCTAGAATGC	-138	1,1	8.82	13.90	s	38	Rmet_5933	0	Op1802f_1	NA
2242	TSS_1640129+3	100	15	0,1	-15	1	-46	TCGACGAGAT	-13	GCTACAATGC	-135	1,1	10.59	-8.10	no	68	Rmet_4967	0	Op1458f_1	NA
2243	TSS_1849541+3	100	15	0,1	-17	1	-36	TTGCGACGCA	-15	AATAAGCTCC	-140	1,1	6.79	10.80	s	63	Rmet_5151	0	Op1516f_2	NA
2244	TSS_2352933+3	100	51	0,1	-17	1	-31	TTGAACGACC	-10	AGTTGACTGC	-147	1,1	2.65	5.60	m	123	Rmet_5599	katA	Op1668f_1	MET-EN-Methane
2245	TSS_575314-3	100	6	0,1	-15	1	-35	TTCAGCGATT	-13	GATAGACTGC	-139	1,1	8.11	10.70	s	716	Rmet_4002	0	Op1123r_1	NA
2246	TSS_1181102-3	100	14	0,1	-13	1	-32	TTGACAGAGA	-11	AGCATAATCT	-142	1,1	9.39	11.60	s	35	Rmet_R0082	0	Op1315r_2	NA
2247	TSS_125495+5	100	6	0,1	-17	1	-44	TTGAGGACTT	-15	GAGACCGTCG	-141	1,1	4.52	-4.60	no	508	Rmet_6271	0	Op1912f_1	NA
2248	TSS_1573938+2	100	6	0,1	-16	1	-42	GCGGCTACGC	-14	GGTAGAATAA	-141	5,1	9.52	-3.10	no	191	Rmet_1453	pps	Op0405f_1	MET-CAH-Pyr

2249	TSS_551578-2	99	8	0,1	-15	1	-45	CCTGACGGCG	-13	GGCATACTGC	-139	5,1	9.77	-13.60	no	1530	Rmet_0513	mdtB	Op0138r_1	EIP-TRA-Ion
2250	TSS_1154086+3	99	7	0,1	-16	1	-38	TAGATGTCGG	-14	CGTATCCTTG	-146	1,1	3.49	6.90	s	47	Rmet_4548	0	Op1304f_1	NA
2251	TSS_317843-2	99	2	0,1	-15	1	-36	GCACAAGTGC	-13	TCTATAATGG	-133	4,1	12.47	7.40	s	67	Rmet_0300	ptsN	Op0086r_3	EIP-TRA-Pts
2252	TSS_318504-2	99	21	0,1	-15	1	-34	TCGACATCAC	-13	GTGAGTATGT	-143	1,1	5.48	10.10	s	363	Rmet_0301	0	Op0086r_2	NA
2253	TSS_726743-3	99	9	0,1	-17	1	-45	TTGAGGCCAC	-15	TGTACAATCA	-139	1,1	9.83	-3.60	no	119	Rmet_4151	0	Op1169r_2	NA
2254	TSS_122471-5	99	46	0,1	-14	1	-34	TCGCGCAGCG	-12	GCCAGAATTC	-139	1,1	7.18	8.70	s	5191	Rmet_6262	0	Op1909r_1	NA
2255	TSS_1130105+2	99	12	0,1	-17	1	-39	CCGGCACCGG	-15	GGCACAATCA	-136	5,1	10.01	3.90	m	116	Rmet_1040	lolE	Op0299f_1	NA
2256	TSS_1828093+2	99	7	0,1	-16	1	-42	TGGAAGATTG	-14	AGTATCCTGC	-143	1,1	7.18	-1.10	no	11	Rmet_1688	0	Op0477f_1	NA
2257	TSS_3371997+2	99	14	0,3	-20	0	-40	GGCCAGGAAC	-10	CTTTGCATGG	-148	4,1	1.63	-8.60	no	77	Rmet_3112	0	Op0875f_3	NA
2258	TSS_3885351+2	99	8	0,4	-13	1	-47	CCAGACATCC	-25	GCTGTAATGG	-145	5,1	5.03	-5.60	no	116	Rmet_3581	0	Op0995f_2	NA
2259	TSS_3636254-2	98	20	0,1	-25	0	-36	TTGAACTTTC	-15	TTGAGTCTTA	-146	1,1	4.54	9.80	s	5720	Rmet_3353	tnpA	Op0934r_1	NA
2260	TSS_547444+3	98	8	0,1	-16	1	-49	TTGCATAGCG	-14	CGTAGTATTC	-143	1,1	7.94	-14.60	no	166	Rmet_3981	0	Op1116f_1	NA
2261	TSS_1874378-3	98	32	0,1	-4	0	-46	GCGGCCGTCG	-13	GCTAGGCTTG	-139	5,1	8.99	-14.10	no	16	Rmet_5179	0	Op1525r_2	NA
2262	TSS_180828+2	98	25	0,1	-17	1	-46	TGGCCACTTG	-15	GTCATACTCG	-138	1,1	7.48	-7.10	no	1552	Rmet_0176	0	Op0053f_1	NA
2263	TSS_920123-2	98	9	0,1	-9	0	-34	TTCCAATCGA	-7	TTCACAATAC	-144	1,1	7.25	4.70	(m)-ba	30	Rmet_0840	0	Op0240r_1	NA
2264	TSS_103738+5	98	16	0,1	-16	1	-37	GCAGAAACCT	-14	GCTAAACTCA	-140	4,1	10.32	4.40	m	7031	Rmet_6258	rhsC	Op1906f_1	NA
2265	TSS_2709921+2	97	14	0,1	-16	1	-46	TTGTACTGGG	-26	TGTATAGTTA	-141	1,1	8.07	0.80	w	3509	Rmet_2499	0	Op0693f_1	NA
2266	TSS_3642209+2	97	22	0,1	-16	1	-35	TGCACGAAGA	-14	TGCACAATGA	-142	1,1	6.54	9.60	s	3588	Rmet_3369	paaA	Op0937f_1	NA
2267	TSS_3832162+2	97	34	0,1	-16	1	-35	GCATCCCGCC	-14	GAAACAATGC	-139	5,1	6.70	5.60	m	469	Rmet_3535	phhR	Op0977f_3	NA
2268	TSS_1056731-3	97	20	0,1	-15	1	-37	TCGACCGCCA	-13	CCTATACTCT	-140	1,1	7.52	9.90	s	26	Rmet_4459	0	Op1269r_1	MET-AA-ValLeuIle
2269	TSS_844314+2	97	8	0,1	-16	1	-49	TTGCATGATC	-14	TGTACACTAA	-141	1,1	8.33	-14.60	no	118	Rmet_0767	phnF	Op0219f_1	NA
2270	TSS_3782632-2	97	25	0,1	-15	1	-43	TCGCCATAGG	-13	CTTAGAATGT	-138	1,1	8.78	-2.10	no	15	Rmet_3491	hemE	Op0970r_3	MET-COF-Porph
2271	TSS_774037-3	97	37	0,1	-15	1	-35	TTGCGAAGTG	-13	CCTATATTGA	-135	1,1	8.65	13.20	s	46	Rmet_4190	0	Op1185r_1	NA
2272	TSS_1138955+2	97	9	0,1	-16	1	-45	TGGCACCCGC	-14	CGCACAATAG	-137	1,1	6.66	-8.10	no	166	Rmet_1046	gntK	Op0301f_1	MET-CAH-PP_KDPG
2273	TSS_1277674+2	97	6	0,1	-16	1	-35	TTGCCCGCGG	-14	TGTATGCTGC	-137	1,1	8.58	11.60	s	69	Rmet_1167	0	Op0333f_3	NA
2274	TSS_2295016+2	97	12	0,5	-19	0	-37	TTGAGACACC	-15	CGTATTGTTT	-140	1,1	6.83	12.40	s	49	Rmet_2113	0	Op0611f_1	NA
2275	TSS_934440+3	97	18	0,1	-16	1	-40	TTCACCTAGC	-14	GTTACAATCC	-140	1,1	10.40	4.90	(m)-sba	137	Rmet_4340	mqo	Op1234f_2	MET-CAH-Pyr
2276	TSS_765995-3	97	18	0,1	-15	1	-41	TGGCCTGACT	-13	GCTATATTGA	-142	1,1	8.07	0.90	(w)-sba	35	Rmet_4180	0	Op1179r_1	NA
2277	TSS_2622660+2	96	33	0,1	-25	0	-45	TCGTGGCCTT	-23	GGTAAAATAG	-140	1,1	7.84	1.90	w	115	Rmet_2412	efp	Op0679f_2	NA
2278	TSS_332066+3	96	20	0,1	-16	1	-47	TGGCCATTCT	-14	AATACAATTA	-140	1,1	9.01	-9.10	no	39	Rmet_3793	0	Op1052f_1	NA
2279	TSS_474000+3	96	27	0,1	-17	1	-36	TTGTTGGGGA	-15	AGTAACGTAG	-147	1,1	4.32	9.80	s	735	Rmet_R0076	0	Op1098f_1	NA
2280	TSS_972420+3	96	15	0,1	-15	1	-34	TTGACATTGC	-13	ATTACGATTT	-141	1,1	9.41	12.60	s	141	Rmet_4374	hmgA	Op1248f_1	MET-AA-Tyr
2281	TSS_1758341+2	96	27	0,1	-16	1	-36	TGGCATGAGC	-14	TGTAGCCTCC	-140	1,1	5.99	8.90	s	258	Rmet_1630	0	Op0457f_1	NA
2282	TSS_1615297+3	96	13	0,1	-17	1	-47	TTCCCTCCGC	-15	GTTATACTCT	-142	1,1	8.57	-9.10	no	15	Rmet_4950	0	Op1454f_1	NA
2283	TSS_2077973-3	96	12	0,1	-2	0	-36	TTGCGACATG	-14	CTTACGCTTC	-141	1,1	7.41	11.40	s	51	Rmet_5356	bug	Op1591r_1	NA
2284	TSS_2200977-3	96	10	0,1	-16	1	-41	TGGACGAGGT	-14	TTTATAATGC	-136	1,1	10.75	3.90	(m)-sba	2146	Rmet_5448	0	Op1619r_1	NA
2285	TSS_1584193+2	96	6	0,1	-17	1	-39	CCGGCATGGC	-15	GTTACTATGG	-136	5,1	9.87	3.90	m	1400	Rmet_1465	int	Op0411f_1	NA
2286	TSS_2341966-2	96	14	0,1	-15	1	-41	GGAAAAGAGT	-13	CCTATAATTC	-138	4,1	12.20	-1.60	no	1882	Rmet_2146	yejA	Op0618r_1	NA
2287	TSS_3106717-2	96	5	0,1	-16	1	-47	GCTGCTGGGC	-14	GGTACTATTC	-139	5,1	9.42	-15.60	no	206	Rmet_2856	hutC	Op0804r_1	NA
2288	TSS_277391-3	96	42	0,1	-16	1	-42	TTCCGCTGCG	-14	CCTACAATGA	-136	2,1	13.36	-1.10	no	68	Rmet_3733	flgA	Op1041r_1	DIV-MOT-Flagel
2289	TSS_388003+2	95	11	0,3	-22	0	-48	TTGCGAAAGC	-19	AAAAGGCTCT	-146	1,1	3.73	-8.60	no	64	Rmet_0366	0	Op0103f_1	NA
2290	TSS_1810113+2	95	21	0,1	-16	1	-37	TCGCCCCGAC	-14	ACTAAAATCG	-141	1,1	8.48	9.90	s	20	Rmet_1674	0	Op0471f_2	NA
2291	TSS_1224055-2	95	18	0,1	-16	1	-36	TTGACGCCTT	-14	ATGATAATAG	-141	1,1	7.74	14.40	s	40	Rmet_1120	rpoI	Op0320r_1	GIP-TK-RNAP
2292	TSS_1795815-2	95	19	0,1	-22	0	-46	TTGCGACTTG	-20	GCGACCATGG	-140	1,1	7.21	-2.60	no	219	Rmet_1660	tnp	Op0468r_3	NA
2293	TSS_20120+5	95	19	0,1	-16	1	-35	TTGTAATCCC	-14	GGGAGGATGG	-140	1,1	6.61	10.60	s	1492	Rmet_6333	tnpA	Op1926f_1	NA
2294	TSS_768497+2	95	22	0,1	-13	1	-33	TTTCGCCGCT	-11	ACCAAGTTAC	-146	2,1	6.29	5.70	m	277	Rmet_6424	0	Op2002f_1	NA
2295	TSS_1498532+2	95	17	0,1	-17	1	-35	TTGCCGCACT	-15	ACTACAATTC	-140	1,1	10.41	11.60	s	41	Rmet_1389	dehII	Op0387f_1	MET-XEN-Hex
2296	TSS_3041646+2	95	29	0,1	-17	1	-36	TTGCAGCAGC	-15	GTTATCGTCG	-138	1,1	7.67	10.80	s	5105	Rmet_6559	0	Op0787f_1	NA
2297	TSS_737888-2	95	5	0,1	-15	1	-43	TTGATACCCA	-13	GTTATTATGA	-139	1,1	8.83	-0.60	no	2444	Rmet_0667	hemL	Op0186r_1	MET-COF-Porph
2298	TSS_2869189-2	95	14	0,1	-16	1	-43	TGGCAACAAC	-14	TGCACAATGA	-137	1,1	7.77	-3.10	no	106	Rmet_6544	0	Op0738r_1	NA
2299	TSS_3438689-2	95	15	0,1	-14	1	-35	TTCAACAGGT	-12	GGTAATATTC	-141	1,1	8.86	9.70	s	44	Rmet_3166	norR	Op0888r_1	NA
2300	TSS_1204445-3	95	17	0,1	-15	1	-34	TGGACGTCTC	-13	CGCATTATTT	-145	1,1	6.15	10.10	s	118	Rmet_4585	ilvD	Op1319r_2	MET-AA-ValLeuIle
2301	TSS_1193106+2	95	9	0,1	-27	0	-35	TTGAGGACAC	-14	AGCAAACTAC	-143	1,1	6.74	10.60	s	11	Rmet_1096	0	Op0315f_3	NA
2302	TSS_3433751+2	95	13	0,1	-13	1	-38	TGGCCGAACT	-11	ATCACAATGT	-137	1,1	7.38	3.90	m	577	Rmet_3163	paaG	Op0887f_1	MET-CAH-Prop
2303	TSS_1373729-2	95	9	0,1	-17	1	-45	TTGAGGCCAC	-15	TGTACAATCA	-139	1,1	9.83	-3.60	no	971	Rmet_1249	0	Op0360r_1	NA
2304	TSS_476113+3	95	48	0,2	-21	0	-49	TTGCTGATTA	-14	CGAAGAGTGC	-145	1,1	4.36	-15.60	no	2065	Rmet_R0079	0	Op1098f_1	NA
2305	TSS_120236+2	94	41	0,1	-15	1	-35	TTGCCAATCC	-13	TCTACCATGA	-138	1,1	9.22	13.20	s	1448	Rmet_0115	bioF	Op0031f_5	MET-COF-Biotine
2306	TSS_1780484+2	94	6	0,3	-27	0	-37	TTGCCTGAGG	-6	GCGATTGTGA	-145	1,1	4.90	1.40	(w)-ba	19	Rmet_1646	0	Op0463f_1	NA
2307	TSS_2379702-2	94	11	0,1	-15	1	-33	TTGACTTGGT	-13	GACATAATCC	-136	1,1	10.17	10.60	s	36	Rmet_6511	0	Op0624r_1	NA
2308	TSS_563569-3	94	13	0,1	-15	1	-39	TCCAACGCCG	-13	GTTAGGCTGG	-143	1,1	4.68	1.40	w	32	Rmet_3993	0	Op1119r_1	NA
2309	TSS_2442133-3	94	19	0,1	-16	1	-47	TTGCCCGAAG	-14	GTTAAGATCG	-140	1,1	9.26	-9.60	no	100	Rmet_5683	0	Op1697r_1	NA

2310	TSS_984434+2	94	8	0,1	-16	1	-38	TGAGAGGCGC	-14	ATTACACTCT	-138	4,1	7.85	4.40	m	49	Rmet_0897	*0*	Op0261f_1	NA	
2311	TSS_1282302+2	94	12	0,1	-13	1	-45	GGCAGCGAAC	-11	GTTAATCTGA	-143	4,1	7.32	-15.60	*no*	21	Rmet_1171	*tnpA*	Op0335f_1	NA	
2312	TSS_3622851+2	94	27	0,1	-16	1	-34	TTGCCGGACG	-14	CCTATATTGA	-140	1,1	8.28	10.60	s	40	Rmet_3345	*0*	Op0931f_4	NA	
2313	TSS_285268+3	94	27	0,1	-1	0	-36	TTGCCATTCG	-14	ACAAACATGT	-142	1,1	5.75	12.40	s	1942	Rmet_3744	*flgL*	Op1042f_3	DIV-MOT-Flagel	
2314	TSS_597101+3	94	8	0,1	-17	1	-36	TGGCGATCGA	-15	CCTATACTGG	-138	1,1	8.72	11.30	s	3106	Rmet_4030	*0*	Op1136f_1	NA	
2315	TSS_2715093+2	94	19	0,1	-16	1	-35	TCGAGACACG	-14	GCTATATTGA	-139	1,1	7.72	12.10	s	8905	Rmet_2508	*0*	Op0695f_1	NA	
2316	TSS_3244443+2	94	27	0,1	-16	1	-47	GCGGCAAGGG	-26	CCGAAAATGT	-138	5,1	9.58	-2.70	*no*	9233	Rmet_2996	*ptxR*	Op0843f_1	NA	
2317	TSS_1988485-2	94	15	0,1	-15	1	-49	TCGCACAACG	-13	CGTATCATTG	-141	1,1	7.65	-16.10	*no*	31	Rmet_1833	*0*	Op0532r_1	NA	
2318	TSS_2618452-2	94	14	0,1	-14	1	-33	TTGCGCGATA	-12	GGTAAGATGC	-140	1,1	9.51	8.60	s	5	Rmet_R0040	*0*	Op0678r_2	NA	
2319	TSS_3428259-2	94	24	0,1	-15	1	-41	TGGAAGGGCC	-13	GGTACTCTGG	-142	1,1	7.26	-1.10	*no*	573	Rmet_R0050	*0*	Op0886r_1	NA	
2320	TSS_3750746-2	94	30	0,2	-10	1	-30	TTCCAGCTTT	-8	AATAGGGTCT	-146	1,1	4.02	3.70	*(m)-nu*	91	Rmet_3470	*0*	Op0964r_1	MET-CAH-Prop	
2321	TSS_2333555+3	94	19	0,1	-16	1	-35	TCGAGCCGAC	-14	AGTACTATGG	-137	1,1	6.88	10.10	s	2623	Rmet_5582	*0*	Op1662f_1	NA	
2322	TSS_2437189-3	94	22	0,1	-16	1	-41	TGGACGAGGT	-14	TTTATAATGC	-136	1,1	10.75	3.90	*(m)-sba*	94	Rmet_5678	*nimA1*	Op1697r_2	EIP-TRA-Ion	
2323	TSS_135784+4	94	12	0,1	-16	1	-36	TTGACCTCTC	-14	GGCACAATTC	-139	1,1	9.95	13.40	s	677	Rmet_6163	*0*	Op1885f_1	NA	
2324	TSS_63682-4	94	11	0,1	-16	1	-35	TTGCTTTGGT	-14	ACTACATTGT	-145	1,1	7.89	10.60	s	38	Rmet_5995	*mhpD*	Op1828r_1	NA	
2325	TSS_393349+2	93	2	0,1	-16	1	-37	TCGGCACGAC	-14	GGTAAAGTGC	-137	5,1	10.76	9.90	s	104	Rmet_0369	*0*	Op0103f_2	EIP-TRA-ABC	
2326	TSS_2055387+2	93	9	0,1	-16	1	-41	TTCATACGGC	-14	GGTAGCATAG	-139	1,1	7.29	1.90	*(w)-sba*	73	Rmet_1888	*yhiR*	Op0545f_1	NA	
2327	TSS_2851151+2	93	17	0,1	-17	1	-44	GCCGAGGTGC	-15	TGTAGCATTG	-139	4,1	8.25	-9.60	*no*	31	Rmet_2620	*sseA*	Op0733f_2	MET-AA-Cys	
2328	TSS_212107+3	93	19	0,1	-16	1	-37	TCGCGGGATC	-14	TCTACAATGA	-139	1,1	8.45	8.90	s	35	Rmet_3675	*int*	Op1024f_1	NA	
2329	TSS_1025219+3	93	8	0,1	-17	1	-37	TTGACGCCCT	-15	CCCATAATTC	-138	1,1	9.12	13.40	s	180	Rmet_4430	*betB*	Op1260f_2	NA	
2330	TSS_1815568+3	93	7	0,1	-17	1	-36	TTGTCACGAC	-15	CGCAGACTCC	-140	1,1	6.57	11.80	s	30	Rmet_5121	*mopI*	Op1508f_3	NA	
2331	TSS_1066727-2	93	38	0,1	-16	1	-42	TCCACGGGGA	-14	ATTAGAATGA	-143	1,1	6.60	-0.60	*no*	133	Rmet_0980	*0*	Op0276r_2	NA	
2332	TSS_945156+3	93	11	0,1	-17	1	-48	GTCAAAATGC	-15	GGTACGATGC	-138	4,1	10.77	-14.10	*no*	36	Rmet_4349	*0*	Op1238f_1	NA	
2333	TSS_1475230-3	93	8	0,1	-16	1	-49	TGCCGGGCGA	-14	ATTACAATAC	-143	1,1	6.52	-16.60	*no*	59	Rmet_4822	*0*	Op1405r_1	NA	
2334	TSS_1864191+3	93	22	0,1	-16	1	-36	TGGCCCGGTG	-14	CGCATAATGC	-138	1,1	8.05	11.90	s	28	Rmet_5165	*0*	Op1520f_2	NA	
2335	TSS_857859-3	93	14	0,1	-16	1	-41	TTGAGTGGAT	-14	GCCATAATAG	-141	1,1	9.25	3.40	*(m)-sba*	223	Rmet_4266	*0*	Op1211r_1	NA	
2336	TSS_198346+4	93	5	0,1	-21	0	-41	TGGCTTGACT	-19	TCTAATATTT	-142	1,1	6.44	4.90	*(m)-nu*	940	Rmet_6383	*0*	Op1861f_1	NA	
2337	TSS_530723+2	92	11	0,1	-17	1	-43	TTCAGACTAC	-15	CTTATTCTTG	-141	1,1	6.56	-1.10	*no*	273	Rmet_0501	*pgk2*	Op0135f_1	MET-CAH-FbP	
2338	TSS_3718791+2	92	22	0,1	-17	1	-36	TTGCATACAA	-15	ACTATATTGG	-144	1,1	8.48	11.80	s	239	Rmet_3444	*dctA2*	Op0959f_1	NA	
2339	TSS_2043884-2	92	6	0,1	-16	1	-42	GCGGCCCGTT	-14	TGTAGAGTGC	-140	5,1	9.12	-4.10	*no*	590	Rmet_1880	*ybbA*	Op0542r_1	EIP-TRA-ABC	
2340	TSS_2324366-2	92	20	0,1	-16	1	-35	TTGCGCAGGA	-14	TGGAGGATGT	-143	1,1	6.31	9.60	s	2088	Rmet_2133	*0*	Op0614r_1	NA	
2341	TSS_2858812-2	92	12	0,1	-16	1	-26	TCGATGAACT	-5	GTGAACATTT	-141	1,1	5.06	0.10	*no*	306	Rmet_2624	*polA*	Op0734r_1	MET-NUC-Pur	
2342	TSS_256718-3	92	13	0,1	-4	0	-46	TTGACGTGCG	-13	TCTAGACTCG	-141	1,1	9.04	-7.60	*no*	32	Rmet_3715	*0*	Op1031r_1	NA	
2343	TSS_1979529+2	92	54	0,5	-18	1	-35	TTGAGAATCG	-15	CCGTTATTTG	-148	1,1	0.36	9.60	s	29	Rmet_1823	*0*	Op0529f_1	NA	
2344	TSS_2022376-2	92	10	0,1	-14	1	-42	TTGCCGGAGC	-12	GGCAAAATGC	-138	1,1	9.95	-1.60	*no*	1024	Rmet_1863	*0*	Op0538r_1	MET-LIP-FASyn	
2345	TSS_3614985-2	92	44	0,2	-20	0	-48	TTGCTGATTA	-13	CGAAGAGTGC	-145	1,1	4.36	-14.60	*no*	2066	Rmet_R0060	*0*	Op0930r_1	NA	
2346	TSS_311812+3	92	16	0,1	-16	1	-35	TTGACAGCAT	-14	ACTAGATTAT	-142	1,1	7.46	13.60	s	91	Rmet_3776	*0*	Op1050f_1	NA	
2347	TSS_787568-3	92	11	0,1	-18	1	-38	TGGCACATTC	-16	GTGATACTGT	-142	1,1	7.09	7.90	s	35	Rmet_4202	*0*	Op1191r_1	NA	
2348	TSS_2233502-3	92	17	0,1	-15	1	-34	TTGCCGGTGT	-13	GTGAAGATGT	-139	1,1	7.47	9.60	s	981	Rmet_5488	*0*	Op1629r_1	GIP-REP-Recomb	
2349	TSS_3756+4	92	12	0,1	-16	1	-41	TTGCGCGCAA	-14	GGTACCATTG	-139	1,1	9.10	1.40	*(w)-sba*	1986	Rmet_6347	*0*	Op1845f_1	NA	
2350	TSS_3786364+2	91	21	0,1	-17	1	-42	TCGATGTCCA	-15	TGCACAATAT	-142	1,1	5.95	-0.10	*no*	5807	Rmet_3502	*0*	Op0971f_1	NA	
2351	TSS_1059375+3	91	23	0,1	-16	1	-40	TTGATGTCAT	-14	GATACGATTG	-140	1,1	9.14	4.40	*(m)-sba*	162	Rmet_4462	*hns*	Op1272f_1	NA	
2352	TSS_146196-3	91	12	0,1	-15	1	-34	TCGCAAATCC	-13	TGGAAAATTC	-140	1,1	7.35	9.10	s	79	Rmet_5940	*0*	Op1805r_1	EIP-SIG-2Comp	
2353	TSS_497601-3	91	6	0,1	-16	1	-48	CCTGCCACGG	-14	GATATAATCC	-137	5,1	11.96	-15.60	*no*	5926	Rmet_3933	*tctC*	Op1099r_1	EIP-TRA-Ion	
2354	TSS_575996-3	91	28	0,1	-22	0	-48	TACGGTCGGC	-14	ATTAATATTC	-146	2,1	7.40	-15.60	*no*	1398	Rmet_4002	*0*	Op1123r_1	NA	
2355	TSS_666745+2	91	14	0,1	-17	1	-45	GCACGAGCAA	-15	GGTAATCTCC	-141	4,1	7.83	-11.60	*no*	425	Rmet_0613	*0*	Op0173f_1	NA	
2356	TSS_1103345+2	91	24	0,1	-17	1	-44	TTGTCAGAAG	-15	GTCATAATGG	-141	1,1	9.46	-0.60	*no*	2316	Rmet_1016	*0*	Op0293f_1	NA	
2357	TSS_3653953+2	91	17	0,1	-17	1	-40	TAGCTTACGC	-15	ACTACAATAG	-147	1,1	4.79	3.90	*(m)-sba*	73	Rmet_3378	*0*	Op0939f_1	NA	
2358	TSS_2319887-3	91	9	0,1	-15	1	-38	TGGCACCGCC	-13	CCTATACTCG	-137	1,1	8.13	5.90	m	706	Rmet_6748	*0*	Op1653r_1	NA	
2359	TSS_639966+2	91	9	0,1	-17	1	-36	TCGCCACCAT	-15	TGTACCATCG	-139	1,1	7.67	11.30	s	56	Rmet_0593	*0*	Op0167f_1	NA	
2360	TSS_930096+2	91	34	0,1	-1	0	-32	AACCTTGTCA	-12	CCTCCTAAGA	-145	3,0	5.99	-3.40	no	63	Rmet_0849	*0*	Op0243f_1	NA	
2361	TSS_2460937+2	91	8	0,1	-16	1	-48	GGACAAACCC	-14	CGTACACTCG	-137	4,1	10.04	-17.60	*no*	28	Rmet_2247	*pdeA*	Op0643f_2	NA	
2362	TSS_3867151-2	91	23	0,1	-26	0	-35	TCGACGCCGA	-9	TGTATACAGC	-143	1,1	3.35	6.70	*(s)-ba*	186	Rmet_3564	*fmt*	Op0988r_1	MET-AA-Met	
2363	TSS_2287155-3	91	23	0,1	-15	1	-41	TCGGCTATAT	-13	GGCAAACTAC	-142	5,1	7.40	-1.10	*no*	153	Rmet_5540	*0*	Op1647r_4	NA	
2364	TSS_3628137+2	90	29	0,1	-16	1	-38	TCGATGCAAC	-14	CATAAACTGC	-144	1,1	6.86	6.90	s	261	Rmet_3352	*0*	Op0933f_1	NA	
2365	TSS_108934-2	90	8	0,1	-15	1	-44	TCGCGATCAG	-13	CGTAGAATCG	-136	1,1	8.28	-5.10	*no*	22	Rmet_0101	*0*	Op0030r_1		0
2366	TSS_182384-2	90	13	0,1	-16	1	-37	GGCGAAGCGC	-14	GATATACTCC	-137	4,1	10.72	5.40	m	16	Rmet_0175	*0*	Op0052r_1	MET-COF-C1	
2367	TSS_3051060-2	90	11	0,1	-15	1	-33	TTGCGTTTTA	-13	TATATGATTC	-140	1,1	8.94	8.60	s	33	Rmet_2803	*ilvR*	Op0788r_1	NA	
2368	TSS_744310+3	90	8	0,1	-17	1	-35	TTGCGTTATA	-15	CGTAAACTTG	-140	1,1	8.83	9.60	s	237	Rmet_4162	*0*	Op1170f_4	NA	
2369	TSS_2419883+2	90	8	0,1	-17	1	-39	TGGTGATCCC	-15	ATTACAATAG	-139	1,1	7.47	6.90	s	148	Rmet_2207	*0*	Op0631f_1	NA	
2370	TSS_952231+3	90	16	0,1	-15	1	-34	TCGGCATTGT	-13	TATACGATAT	-143	5,1	7.76	10.10	s	181	Rmet_4357	*0*	Op1242f_2	NA	

2371	TSS_1653983-3	90	28	0,1	-14	1	-33	TCGACTTCTC	-12	AGCAAACTGG	-144	1,1	5.84	8.10	s	59	Rmet_4976	0	Op1461r_1	NA
2372	TSS_1107691-2	90	25	0,1	-16	1	-34	TTGCAGGTAC	-14	TTTATGCTGC	-141	1,1	8.53	8.60	s	74	Rmet_1017	phaZ1	Op0294r_1	NA
2373	TSS_1002801+3	90	16	0,1	-18	1	-37	TTGCGCAAGA	-16	TATACAATGC	-138	1,1	10.45	10.80	s	5	Rmet_R0080	0	Op1256f_1	NA
2374	TSS_1483061+3	90	10	0,1	-17	1	-37	TTGGACCTTAC	-15	GCCAATATCT	-144	1,1	6.47	9.90	s	20	Rmet_4829	qseB	Op1408f_1	EIP-SIG-2Comp
2375	TSS_2110525+3	90	5	0,1	-25	0	-48	TGCCCGTTCC	-23	TCCACACAGC	-146	1,1	0.24	-8.60	no	79	Rmet_5384	0	Op1602f_2	NA
2376	TSS_306028-2	89	27	0,1	-15	1	-34	TTGACAAGGA	-13	TCTATAGTTG	-139	1,1	9.79	13.60	s	332	Rmet_R0003	0	Op0084r_2	NA
2377	TSS_947475-2	89	17	0,1	-16	1	-48	TTGCGAGATT	-24	GGCACGCTCG	-139	1,1	6.95	-3.60	no	33	Rmet_0867	0	Op0250r_1	NA
2378	TSS_87294-4	89	26	0,1	-16	1	-41	TGGACGAGGT	-14	TTTATAATGC	-136	1,1	10.75	3.90	(m)-sba	199	Rmet_5974	ompP	Op1818r_2	NA
2379	TSS_12561-5	89	22	0,1	-15	1	-42	TCCAGAGACT	-13	GCTAAGCTCT	-144	1,1	5.49	-3.60	no	5194	Rmet_6320	int	Op1919r_1	NA
2380	TSS_117898-5	89	13	0,1	-16	1	-43	TCACGCACAT	-14	GGGAAACTCG	-144	4,1	6.84	-6.60	no	618	Rmet_6262	0	Op1909r_1	NA
2381	TSS_2159819+2	89	18	0,1	-17	1	-43	TTCCACGGCA	-15	GGTATTATCT	-139	1,1	8.16	-2.10	no	370	Rmet_6495	0	Op0579f_1	NA
2382	TSS_1460589-2	89	4	0,1	-22	0	-41	TGGATGCCGT	-20	GCCATCATCT	-140	1,1	6.53	5.30	m	12307	Rmet_1338	0	Op0378r_1	NA
2383	TSS_2322384-2	89	8	0,1	-16	1	-45	GGCGCCATGC	-14	TCTAGAATGC	-141	4,1	9.00	-10.60	no	106	Rmet_2133	0	Op0614r_1	NA
2384	TSS_3570219-2	89	24	0,1	-17	1	-40	TTGACGAATG	-15	GTTAACATCC	-139	1,1	9.54	6.40	(s)-sba	356	Rmet_3293	rpsK	Op0926r_2	GIP-TL-Ribo
2385	TSS_3628675-2	89	29	0,1	-15	1	-46	TTGCAGTTGA	-13	GTTATCATCA	-141	1,1	9.66	-8.60	no	480	Rmet_3351	0	Op0932r_1	NA
2386	TSS_1043437+2	89	13	0,1	-17	1	-35	TTCTGTGACG	-15	GCTATGATCT	-138	1,1	6.99	9.10	s	33	Rmet_0957	ureD	Op0267f_5	GIP-PTL-Fold
2387	TSS_2056392+2	89	19	0,1	-16	1	-39	TGCAAATCTT	-14	GATAAAATTA	-146	1,1	8.49	5.40	m	219	Rmet_6484	0	Op0545f_1	NA
2388	TSS_2664909+2	89	21	0,1	-16	1	-47	TCGCTGTGAG	-14	GGCAGGATGC	-143	1,1	5.74	-13.10	no	821	Rmet_2454	0	Op0685f_2	NA
2389	TSS_500617-3	89	20	0,1	-16	1	-41	TGGACGAGGT	-14	TTTATAATGC	-136	1,1	10.75	3.90	(m)-sba	308	Rmet_6635	0	Op1101r_1	NA
2390	TSS_2840788-2	88	11	0,1	-15	1	-38	TGGCGCGCTC	-13	GATACAATCC	-136	1,1	9.09	5.90	m	122	Rmet_2610	yhiN	Op0730r_1	NA
2391	TSS_1619360-3	88	8	0,1	-16	1	-35	TTGATGAAAC	-14	GTTAGAGTCG	-143	1,1	8.12	11.60	s	123	Rmet_4953	0	Op1455r_1	NA
2392	TSS_101158+5	88	19	0,1	-17	1	-36	TTCACTAAGC	-15	GGCAAAGTCC	-142	1,1	6.30	10.30	s	427	Rmet_6254	0	Op1904f_4	NA
2393	TSS_1900763+2	88	14	0,1	-17	1	-42	TTGTCATCCA	-15	GTTATCATCG	-141	1,1	9.21	3.40	(m)-sba	39	Rmet_1751	agrR	Op0499f_1	EIP-SIG-2Comp
2394	TSS_2482704+2	88	32	0,1	-3	0	-31	GCAATCGCCG	-11	ATAGATGAGG	-143	3,0	4.47	-5.40	no	355	Rmet_R0036	0	Op0645f_4	NA
2395	TSS_1068827-2	88	22	0,1	-15	1	-35	TTGCACGCAT	-13	AGCAGAATCA	-145	1,1	7.45	11.20	s	60	Rmet_0982	bug	Op0276r_1	NA
2396	TSS_2282797-2	88	6	0,2	-13	1	-42	GAATTTCGTA	-22	CCCGTACGCC	-145	3,0	6.11	-6.20	no	210	Rmet_2101	hfq	Op0608r_2	NA
2397	TSS_2306679+3	88	15	0,1	-20	0	-39	TGGCCCGCAA	-18	CATAGTATGT	-141	1,1	6.04	7.30	s	618	Rmet_5559	0	Op1650f_1	NA
2398	TSS_65539-3	88	5	0,1	-15	1	-33	TCGCGACGAA	-13	GCGATACTGC	-137	1,1	6.90	7.10	s	28	Rmet_5869	bug	Op1777r_1	NA
2399	TSS_159687-3	88	30	0,1	-16	1	-35	TTGCCAAAGG	-14	CATATAGTTT	-141	1,1	8.83	13.60	s	29	Rmet_3623	0	Op1009r_1	NA
2400	TSS_1182487-3	88	15	0,1	-15	1	-36	TGGCAGTTGC	-13	TCTACCATTG	-138	1,1	8.09	8.90	s	32	Rmet_4568	codA	Op1315r_2	MET-NUC-Pyr
2401	TSS_2271445-3	88	4	0,1	-20	0	-34	TTGACGCCGA	-13	AATATCATTG	-140	1,1	8.61	12.60	s	24	Rmet_5524	0	Op1641r_1	MET-LIP-FASyn
2402	TSS_2563332-3	88	21	0,1	-7	0	-30	GAACGTCTCG	-10	TGTCTACGAG	-144	3,0	4.95	-4.40	no	27	Rmet_5803	mscS	Op1749r_2	NA
2403	TSS_163312-5	88	23	0,1	-15	1	-33	GCAACAGCAT	-13	GCTATCATCA	-142	4,1	10.40	5.60	m	5953	Rmet_6301	trbF	Op1917r_1	NA
2404	TSS_3575032+2	88	15	0,1	-17	1	-39	TCGATCTTGT	-15	GGGAAAATGA	-142	1,1	7.20	5.90	m	10641	Rmet_3322	0	Op0927f_1	NA
2405	TSS_1783544-2	88	3	0,1	-15	1	-34	TGCACCGCTG	-13	CCTATAATCC	-138	1,1	8.02	10.60	s	24	Rmet_1648	rpoP	Op0464r_1	GIP-TK-RNAP
2406	TSS_3320696-2	88	26	0,1	-16	1	-38	TTGAGGCGGT	-14	GGCATCGTTG	-142	1,1	6.32	7.40	s	2190	Rmet_3060	mpl	Op0860r_1	NA
2407	TSS_226873-4	88	27	0,1	-16	1	-41	TGGACGAGGT	-14	TTTATAATGC	-136	1,1	10.75	3.90	(m)-sba	126	Rmet_6075	0	Op1850r_2	NA
2408	TSS_3094766+2	87	11	0,1	-16	1	-36	TGGACCTTCT	-14	GCTATCGTTC	-140	1,1	7.27	11.90	s	2568	Rmet_2849	yggG	Op0803f_1	GIP-PTL-Fold
2409	TSS_1610668-2	87	5	0,1	-15	1	-47	TGGCAGTAGC	-13	GCTAGCATTC	-143	1,1	8.35	-13.10	no	7927	Rmet_1481	ydcN	Op0418r_1	NA
2410	TSS_2855591-2	87	31	0,1	-6	0	-34	TTGTCAGGGG	-13	AGCACACTGG	-142	1,1	7.12	10.60	s	122	Rmet_2623	ndh	Op0734r_1	MET-EN-OxPhos
2411	TSS_3295659-2	87	6	0,5	-1	0	-46	TGGAGCAGGG	-5	TCGACCGTTG	-144	1,1	3.21	-20.10	no	1842	Rmet_3039	0	Op0854r_1	NA
2412	TSS_149450+3	87	24	0,1	-17	1	-36	TTGTTGGGGA	-15	AGTAACGTAG	-147	1,1	4.32	9.80	s	735	Rmet_R0071	tRNA-I	Op1006f_1	NA
2413	TSS_1163042+3	87	18	0,1	-16	1	-39	TCGCTATCGT	-14	AGCATCATGA	-145	1,1	5.61	4.90	m	2679	Rmet_4553	0	Op1306f_3	NA
2414	TSS_945899-3	87	15	0,1	-15	1	-35	TTGCTATTCA	-13	CTTAGTCTGT	-144	1,1	6.40	11.20	s	545	Rmet_6659	0	Op1237r_1	NA
2415	TSS_54660+5	87	17	0,1	-2	0	-23	TCGACCTGGT	0	GATAGCCTCC	-142	1,1	6.69	-2.30	no	24	Rmet_6208	cnrC	Op1896f_2	EIP-TRA-Ion
2416	TSS_1109119+2	87	20	0,1	-16	1	-41	TGGTCAGCTG	-14	CGTAGAATCG	-140	1,1	7.77	2.90	(w)-sba	144	Rmet_1019	uvrB	Op0295f_1	GIP-REP-Recomb
2417	TSS_756516+3	87	21	0,1	-15	1	-33	TTGCCAACAG	-13	GCGAGAATGC	-138	1,1	9.02	9.60	s	4097	Rmet_4175	pmi	Op1176f_1	MET-CAH-FruMan
2418	TSS_1758310-3	87	15	0,1	-15	1	-34	TTGCAAAGGT	-13	CGGATGCTTG	-138	1,1	6.86	9.60	s	78	Rmet_5068	0	Op1491r_1	NA
2419	TSS_3737128+2	87	5	0,1	-17	1	-36	TTGCCGTGCG	-15	GGCAAACTAC	-138	1,1	7.94	10.80	s	107	Rmet_3461	0	Op0963f_1	MET-AA-Met
2420	TSS_2276870-3	87	8	0,1	-15	1	-41	TGCAAAAGGA	-13	TGTAGGATAG	-145	1,1	6.27	-0.60	no	5	Rmet_5529	0	Op1643r_2	MET-XEN-Benz
2421	TSS_151563+3	86	33	0,2	-21	0	-49	TTGCTGATTA	-14	CGAAGAGTGC	-145	1,1	4.36	-15.60	no	2065	Rmet_R0074	0	Op1006f_1	NA
2422	TSS_100924+4	86	13	0,1	-16	1	-38	TTGTGCACAG	-14	TATACACTTC	-143	1,1	7.97	7.40	s	429	Rmet_5960	int	Op1813f_2	NA
2423	TSS_116166+5	86	17	0,1	-16	1	-38	TCGACGCAGT	-14	TCTACAATGA	-140	1,1	9.18	8.90	s	92	Rmet_6395	0	Op1908f_2	NA
2424	TSS_3649786+2	86	16	0,1	-16	1	-46	TTGCCGCCAAT	-14	ATTACAATGG	-139	1,1	7.50	-10.60	no	37	Rmet_3374	0	Op0937f_2	NA
2425	TSS_3860340+2	86	14	0,1	-16	1	-45	TTGCCGGTCT	-19	TGTATTCTAC	-140	1,1	7.85	-0.60	no	7474	Rmet_3566	0	Op0989f_1	NA
2426	TSS_449192+3	86	22	0,1	-5	0	-23	TCCACGTTCG	-3	ACTATGATGA	-144	1,1	6.20	-2.40	no	131	Rmet_3904	yeaG	Op1092f_1	NA
2427	TSS_1549801-3	86	17	0,1	-16	1	-41	TCGAGACAGA	-14	CCTATAATGG	-141	1,1	9.57	3.90	(m)-sba	32	Rmet_4887	0	Op1429r_2	NA
2428	TSS_2009927-3	86	33	0,5	-16	1	-28	TCGAGGGGCC	-7	GCCAGCAAAC	-147	1,1	-0.37	0.10	no	68	Rmet_5306	tar	Op1567r_1	DIV-MOT-Chemotax
2429	TSS_2297290-3	86	16	0,1	-15	1	-39	TTGCAACAGG	-13	GTTATTATCT	-139	1,1	9.75	6.40	s	2466	Rmet_6743	0	Op1647r_1	NA
2430	TSS_1727142+3	86	13	0,1	-16	1	-42	GCAGACAAGG	-14	GGTATGATCC	-140	4,1	10.46	-5.60	no	2424	Rmet_5043	0	Op1484f_1	NA
2431	TSS_1163699-3	86	8	0,1	-17	1	-40	TCGACACTAT	-15	TGTATATTTC	-142	1,1	8.44	6.90	(s)-sba	2902	Rmet_4549	uvrA2	Op1305r_1	GIP-REP-Recomb

2432	TSS_1819473-3	86	14	0,1	-1	0	-40	TTCAAGTGCA	0	TGCAATGTTC	-147	1,1	3.67	-13.10	no	126	Rmet_5124	0	Op1509r_1	NA
2433	TSS_3079104+2	85	21	0,1	-16	1	-49	TGCAGGGCAC	-26	GCCAAACTGC	-141	1,1	5.52	-5.60	no	1219	Rmet_2831	0	Op0797f_2	NA
2434	TSS_3882583+2	85	15	0,1	-16	1	-49	TCAACCGCAC	-14	GATACCATTG	-139	4,1	8.36	-15.60	no	106	Rmet_3578	gyaR	Op0995f_1	MET-AA-GlySerThr
2435	TSS_949056-2	85	32	0,1	-15	1	-38	TATCTGTTCC	-13	GCGAAGATGC	-143	2,1	8.63	2.40	w	1614	Rmet_0867	0	Op0250r_1	NA
2436	TSS_3429752-2	85	34	0,2	-20	0	-48	TTGCTGATTA	-13	CGAAGAGTGC	-145	1,1	4.36	-14.60	no	#NV	#NV	#NV	#NV	#NV
2437	TSS_61998+4	85	18	0,1	-27	0	-36	TTGTTGTCTC	-15	TTTAGCTTAG	-146	1,1	4.16	9.80	s	1812	Rmet_5994	gntR	Op1827f_1	NA
2438	TSS_141359+5	85	12	0,1	-16	1	-39	TCGATACAAG	-14	TGTATTCTTG	-142	1,1	6.36	5.90	m	24	Rmet_6286	0	Op1916f_3	NA
2439	TSS_2021638+2	85	5	0,1	-16	1	-36	GCTACAGGGG	-14	AGTACAATAC	-141	5,1	8.89	9.40	s	52	Rmet_1864	0	Op0539f_1	NA
2440	TSS_3789967+2	85	6	0,1	-3	0	-35	TTGCGTTCAG	-1	ATCAAACTAC	-147	1,1	5.95	-1.80	no	2204	Rmet_3502	0	Op0971f_1	NA
2441	TSS_2979681-2	85	16	0,1	-16	1	-40	TCGCGCCGGA	-14	GGTAAAATTC	-136	1,1	9.49	2.90	(w)-sba	125	Rmet_2744	rsuA	Op0772r_1	GIP-TL-Ass
2442	TSS_67668+2	85	11	0,1	-17	1	-35	TTGATAGTGG	-15	ACTAGAATTC	-141	1,1	9.73	12.60	s	104	Rmet_0059	0	Op0019f_1	NA
2443	TSS_214314+2	85	20	0,1	-16	1	-35	TTCTTGAGTG	-14	GGTAGAGTTG	-141	1,1	6.33	9.10	s	71	Rmet_0205	aptA	Op0061f_1	MET-CAH-Prop
2444	TSS_1688961+2	85	17	0,1	-16	1	-46	TGCTCGGTAG	-14	GGTATCCTGG	-142	1,1	5.72	-10.60	no	2040	Rmet_1570	0	Op0433f_1	NA
2445	TSS_1764699+2	85	17	0,1	-17	1	-39	GCGAACGGGT	-15	GGTACGATAT	-143	5,1	7.70	2.90	w	191	Rmet_1636	0	Op0459f_1	MET-CAH-But
2446	TSS_1981608-2	85	18	0,1	-16	1	-43	TGGACGAAGG	-14	GGTATCGTCA	-140	1,1	6.65	-2.10	no	274	Rmet_1824	arp3	Op0530r_2	NA
2447	TSS_2940713-2	85	6	0,1	-15	1	-47	TTGCCCTTTG	-13	GGCACAATTG	-139	1,1	9.78	-10.60	no	61	Rmet_2707	0	Op0766r_1	NA
2448	TSS_3526081-2	84	13	0,1	-15	1	-47	TTTCGTTGCG	-13	TCTACAATGG	-132	2,1	16.30	-12.10	no	66	Rmet_3254	0	Op0918r_3	NA
2449	TSS_433723+3	84	14	0,1	-17	1	-44	TGGATTATGA	-15	GGTAGCCTCC	-148	1,1	6.30	-5.10	no	21	Rmet_3889	0	Op1084f_1	NA
2450	TSS_472543+3	84	26	0,1	-17	1	-45	GCGTCCGCCC	-15	AATACAATAG	-140	5,1	8.26	-8.10	no	601	Rmet_R0075	0	Op1098f_1	NA
2451	TSS_472966+3	84	21	0,1	-25	0	-46	GCAAAGCCGG	-23	TTAACAATCA	-143	4,1	6.48	-4.60	no	178	Rmet_R0075	0	Op1098f_1	NA
2452	TSS_2509422+3	84	2	0,1	-17	1	-38	GCAGGGCCAC	-15	CGTATGGTGC	-144	4,1	7.39	2.40	w	2325	Rmet_5751	0	Op1722f_1	NA
2453	TSS_906612-3	84	8	0,1	-4	0	-35	TTGCGGATCA	-2	ACTACGATTC	-143	1,1	8.51	0.20	no	24	Rmet_4312	0	Op1229r_2	NA
2454	TSS_1307866+2	84	20	0,1	-17	1	-49	TTGATCGGAT	-15	GCTAGCATCC	-140	1,1	8.75	-12.60	no	4256	Rmet_6442	0	Op0347f_1	NA
2455	TSS_2190720+2	84	23	0,1	-5	0	-24	TTCCCGTTGA	-3	GTCACAGTCC	-144	1,1	5.58	-1.90	no	1571	Rmet_2027	emrA	Op0593f_1	EIP-TRA-Other
2456	TSS_3632926+2	84	22	0,2	-21	0	-35	TGGACTTGGG	-14	CGGAAGATTC	-141	1,1	5.59	10.10	s	172	Rmet_3357	0	Op0935f_2	NA
2457	TSS_1306744-2	84	4	0,1	-15	1	-34	TGGATATTTC	-13	CTCATCTTGC	-144	1,1	4.24	9.10	s	39	Rmet_1191	prlC	Op0344r_1	NA
2458	TSS_2395645-2	84	8	0,1	-14	1	-33	TGGCGGGCGG	-12	TCAATGATAT	-143	1,1	3.94	7.10	s	513	Rmet_2186	glmM	Op0628r_3	MET-CAH-AminSug
2459	TSS_3617098-2	84	27	0,1	-16	1	-35	TTGTTGGGGA	-14	AGTAACGTAG	-147	1,1	4.32	9.60	s	736	Rmet_R0063	0	Op0930r_1	NA
2460	TSS_3630556-2	84	10	0,1	-12	1	-44	GGCAGCGAAC	-10	GTTAATCTGA	-144	4,1	7.32	-14.60	no	22	Rmet_3353	tnpA	Op0934r_1	NA
2461	TSS_1406848-3	84	17	0,1	-26	0	-27	TTGCGAAGAA	-7	CTGATCCTTC	-143	1,1	5.88	1.60	(w)-nu	484	Rmet_4760	0	Op1385r_1	NA
2462	TSS_231801+4	84	14	0,1	-16	1	-42	TCGGCTATAT	-14	GGCAAACTAC	-142	5,1	7.40	-2.10	no	173	Rmet_6066	0	Op1849f_2	NA
2463	TSS_1573411-2	84	4	0,1	-15	1	-45	CCTGAAATCC	-13	ACCAGAATGC	-140	5,1	8.52	-12.60	no	558	Rmet_1451	0	Op0404r_1	NA
2464	TSS_666562+3	84	20	0,1	-16	1	-41	TTGCCTTTTT	-14	TGTACCATCC	-141	1,1	9.27	2.40	(w)-sba	18	Rmet_4099	caiB	Op1154f_2	NA
2465	TSS_1380494+3	84	18	0,1	-15	1	-34	TTGCGCGCTT	-13	TGTACGATGA	-140	1,1	8.51	9.60	s	91	Rmet_4736	kdgD	Op1370f_1	MET-CAH-Ascorb
2466	TSS_2126279-3	84	18	0,1	-15	1	-33	TTCCGGTTTC	-13	GCTACAGTCA	-140	1,1	7.18	6.10	m	127	Rmet_5397	0	Op1607r_1	NA
2467	TSS_19137+4	84	6	0,1	-18	1	-36	TTGACAACAT	-16	CGTATTATAC	-140	1,1	9.43	13.80	s	28	Rmet_6350	0	Op1843f_1	NA
2468	TSS_3339051+2	83	30	0,1	-17	1	-49	TGCATCGGTA	-15	GTTAGAATGC	-140	1,1	7.63	-14.60	no	30	Rmet_3084	rdgC	Op0867f_1	GIP-REP-Recomb
2469	TSS_268057-2	83	16	0,1	-14	1	-35	TGGAACGAAT	-12	CATCAAATCC	-142	1,1	3.84	8.70	s	422	Rmet_0250	ctpA	Op0072r_1	NA
2470	TSS_1150735-2	83	4	0,1	-12	1	-35	GCGGAACCAC	-4	ATGACACTCC	-142	5,1	7.13	-2.30	no	34	Rmet_1057	0	Op0302r_1	NA
2471	TSS_1966314-2	83	14	0,1	-15	1	-41	TGCCGACAGA	-13	GGTAGACTCG	-140	1,1	7.06	-1.60	no	46	Rmet_1812	0	Op0526r_2	NA
2472	TSS_729587+3	83	17	0,1	-17	1	-39	TCGTACCTAT	-15	GGCACAATTT	-143	1,1	7.15	4.90	m	238	Rmet_4154	tnpA	Op1170f_1	NA
2473	TSS_1406364-3	83	30	0,5	-16	1	-35	TCGCCCGGGC	-14	GCTGTAATCC	-145	1,1	3.78	10.10	s	0	Rmet_4760	0	Op1385r_1	NA
2474	TSS_158768+4	83	24	0,3	-15	1	-36	TTGCGTACTG	-15	CGCAAGATAT	-143	1,1	5.57	9.80	s	3536	Rmet_6137	ubiE	Op1875f_1	NA
2475	TSS_220388+2	83	8	0,1	-16	1	-47	TCGGAACTGT	-14	TGAATAATGG	-144	5,1	8.53	-10.10	no	55	Rmet_0209	0	Op0063f_1	NA
2476	TSS_3262557+2	83	12	0,1	-15	1	-34	TCGACTTCTC	-13	AGCAAACTGG	-144	1,1	5.84	9.10	s	58	Rmet_3006	0	Op0845f_1	NA
2477	TSS_726869-2	83	25	0,1	-15	1	-35	TAGCGAGATT	-13	ACTATATTCC	-147	1,1	4.66	11.70	s	465	Rmet_0659	0	Op0182r_1	NA
2478	TSS_1998387-2	83	32	0,1	-15	1	-34	TCGCGCAAAA	-13	GCGAGGATAA	-143	1,1	4.15	7.10	s	11	Rmet_1843	0	Op0534r_1	MET-CAH-But
2479	TSS_2961811-2	83	12	0,1	-10	1	-29	TCGAGCTTGA	-8	TTGATAATCG	-143	1,1	7.07	5.10	(m)-nu	185	Rmet_2725	0	Op0770r_1	NA
2480	TSS_943296+3	83	19	0,1	-16	1	-43	TGAAGGGCAT	-14	GAGAAAATAG	-144	4,1	6.73	-4.60	no	1896	Rmet_4349	0	Op1238f_1	NA
2481	TSS_2226872+3	83	6	0,1	-17	1	-43	TGGTAGATAT	-15	CCCAAGCTCC	-144	1,1	3.98	-5.10	no	3065	Rmet_6732	0	Op2096f_1	NA
2482	TSS_754407+2	83	22	0,1	-27	0	-42	TTCATGTGGT	-5	GACATATTTA	-146	1,1	5.91	-10.10	no	20	Rmet_0681	glnB	Op0191f_2	EIP-SIG-2Comp
2483	TSS_772401+2	83	12	0,1	-17	1	-37	TGGCGGGCCC	-13	CATATTCTGG	-142	1,1	5.70	6.90	s	74	Rmet_0700	dadA2	Op0197f_1	MET-EN-Nitrog
2484	TSS_341157-2	83	29	0,1	-15	1	-33	TCGATGGATT	-13	GCTAGGCTCA	-143	1,1	5.54	6.10	m	297	Rmet_0322	tnmB	Op0092r_1	NA
2485	TSS_2363861+3	83	13	0,1	-16	1	-36	TCCCGCAATC	-14	CCTAAACTGC	-144	1,1	6.31	8.40	s	62	Rmet_5608	cyoA	Op1674f_1	MET-EN-OxPhos
2486	TSS_2526642+2	82	17	0,1	-7	0	-36	TGGAGAACCC	-5	GTGACATTGG	-140	1,1	5.73	1.90	(w)-ba	334	Rmet_2305	0	Op0659f_1	NA
2487	TSS_3818027-2	82	13	0,1	-16	1	-30	TTGACCTTCC	-7	TTCACAGTGC	-142	1,1	7.06	6.20	(m)-nu	39	Rmet_3524	cupA	Op0974r_1	MET-EN-P_ATPase
2488	TSS_814441-3	82	10	0,1	-12	1	-44	GGCAGCGAAC	-10	GTTAATCTGA	-142	4,1	7.32	-14.60	no	22	Rmet_4228	tnpA	Op1199r_2	NA
2489	TSS_1179605-3	82	7	0,1	-16	1	-34	TTGCGCGCGA	-14	GATACAATTC	-139	1,1	10.51	9.60	s	6	Rmet_R0081	0	Op1315r_3	NA
2490	TSS_325959-2	82	29	0,1	-15	1	-35	TTGCAGTGCT	-13	CGTAGAGTAG	-137	1,1	7.51	11.20	s	54	Rmet_0309	0	Op0088r_1	NA
2491	TSS_59211+3	82	9	0,1	-11	1	-34	TGCAACCGAT	-9	CTGACAATTA	-148	1,1	5.14	5.20	(m)-ba	1654	Rmet_5864	cynS	Op1774f_1	MET-EN-Nitrog
2492	TSS_2229048-3	82	10	0,1	-15	1	-49	TGGCGCCAGA	-13	GCTATTCTAT	-144	1,1	6.41	-17.10	no	258	Rmet_5486	0	Op1629r_1	NA

2493	TSS_255343-2	82	11	0,1	-16	1	-45	TTGCCGGAGC	-14	GCCATAATCC	-137	1,1	10.18	-4.60	no	245	Rmet_0238	0	Op0070r_1	NA
2494	TSS_1877718-2	82	12	0,1	-15	1	-40	TGGCCACGAC	-13	GCTACATTCT	-136	1,1	8.11	2.90	(w)-sba	1328	Rmet_1731	0	Op0490r_1	NA
2495	TSS_975183+3	82	14	0,1	-17	1	-40	TCGCGTCGGC	-15	GGTAAAATCC	-137	1,1	9.28	3.90	(m)-sba	144	Rmet_4376	0	Op1248f_2	NA
2496	TSS_3271982+2	81	16	0,1	-16	1	-36	TTGTCTTGAG	-14	GGTAAGTTGA	-145	1,1	6.55	11.40	s	74	Rmet_3013	yodB	Op0847f_1	NA
2497	TSS_1535343+3	81	6	0,1	-17	1	-36	TTGACCAGTG	-15	TGGACGATAC	-139	1,1	6.64	11.80	s	23	Rmet_4873	0	Op1424f_1	NA
2498	TSS_2391427+3	81	6	0,1	-16	1	-35	TCGATAAGGC	-14	AGTAGAATCG	-137	1,1	8.46	12.10	s	30	Rmet_5633	yddG	Op1680f_1	NA
2499	TSS_2247944-3	81	19	0,1	-13	1	-36	TCCAGCGAGG	-11	ATTACACTGG	-146	1,1	5.94	6.40	s	5290	Rmet_5498	0	Op1635r_1	NA
2500	TSS_1192358-2	81	9	0,1	-16	1	-34	TGGCCACGGT	-14	GATAAAGTCG	-138	1,1	7.56	9.10	s	5036	Rmet_1091	0	Op0314r_1	NA
2501	TSS_100255+3	81	5	0,1	-16	1	-41	CCGGCAAGGC	-14	CGCATAATTC	-137	5,1	11.02	-0.10	no	64	Rmet_5900	0	Op1788f_1	NA
2502	TSS_26760+4	81	10	0,1	-17	1	-44	TGGCGAGTCG	-15	GGTATAGTCA	-142	1,1	7.75	-3.10	no	3235	Rmet_6033	0	Op1837f_1	NA
2503	TSS_143856+4	81	30	0,1	-16	1	-34	TTGAGGAGGC	-14	GAGAGAATGG	-140	1,1	8.48	9.60	s	346	Rmet_6152	tnpB	Op1881f_1	NA
2504	TSS_212515-4	81	12	0,1	-15	1	-35	TGGCTAGCCC	-13	GGGACAATGG	-136	1,1	7.40	10.70	s	26	Rmet_6089	0	Op1854r_1	NA
2505	TSS_1012316+2	81	5	0,1	-15	1	-33	TTTCTCCCCC	-13	GTGAAATTGG	-144	2,1	7.79	5.10	m	230	Rmet_0927	nuoA	Op0267f_3	MET-EN-OxPhos
2506	TSS_2332070+2	81	11	0,1	-28	0	-34	TGGAATCATT	-9	CTGACATTCG	-143	1,1	4.34	5.70	(m)-ba	65	Rmet_2141	0	Op0617f_1	NA
2507	TSS_2370444+2	81	6	0,1	-17	1	-32	TTGAGCTCGG	0	GCTATGTTGC	-142	1,1	8.24	-0.80	no	390	Rmet_2168	0	Op0623f_1	NA
2508	TSS_2931327+2	81	15	0,1	-13	1	-45	TCGAGGAAGG	-11	GCTATCATCG	-139	1,1	8.44	-9.10	no	63	Rmet_2699	acsA	Op0761f_1	MET-CAH-But
2509	TSS_1630899-2	81	5	0,1	-13	1	-37	TCGCTGTGAT	-11	GATATTCTTT	-147	1,1	6.54	4.90	m	24	Rmet_1508	cbbR2	Op0424r_1	NA
2510	TSS_1756492-2	81	7	0,1	-1	0	-45	TTCCCTAACT	-25	GACCCAATGC	-144	1,1	3.08	-1.70	no	53	Rmet_1627	0	Op0454r_1	EIP-SIG-2Comp
2511	TSS_2062558-2	81	19	0,1	-15	1	-38	TCGATAGTAG	-13	GTAAAAATAC	-145	1,1	5.62	6.90	s	22	Rmet_1894	aaeR	Op0548r_1	NA
2512	TSS_242939+2	80	7	0,1	-16	1	-37	CCTGCCGGCC	-14	GGTATCATCG	-132	5,1	11.32	5.40	m	19	Rmet_0229	dctB	Op0067f_1	NA
2513	TSS_405106+2	80	23	0,1	-22	0	-47	TTGTTACTAC	-20	GGGAGAATGT	-142	1,1	7.49	-3.60	no	56	Rmet_0379	0	Op0103f_5	NA
2514	TSS_1172746+2	80	8	0,1	-15	1	-45	TCGTCAGGGC	-13	CTTACACTCG	-139	1,1	7.06	-7.10	no	107	Rmet_1080	cysE	Op0311f_1	MET-EN-Sulf
2515	TSS_2041020+2	80	21	0,1	-17	1	-42	TTCCGGAATT	-15	GATATACTTG	-140	1,1	9.05	-0.10	no	45	Rmet_1879	pgi	Op0541f_6	MET-CAH-FbP
2516	TSS_992100+3	80	13	0,1	-4	0	-48	TTGCGCTACA	-15	CTTAGAGTGA	-143	1,1	6.94	-11.60	no	49	Rmet_4395	uspA9	Op1250f_1	NA
2517	TSS_2421875+3	80	21	0,1	-17	1	-46	TCGATACGGG	-15	TGTAGAATCC	-139	1,1	8.46	-6.10	no	23	Rmet_5662	0	Op1692f_1	NA
2518	TSS_3557391+2	80	19	0,1	-16	1	-41	CCTGCTCTGC	-14	GATAGAGTTG	-141	5,1	7.24	-3.60	no	3768	Rmet_3285	engB	Op0925f_1	NA
2519	TSS_770314-2	80	12	0,3	-23	0	-35	CTGACCCGCT	-13	ATGAGAATTT	-146	1,1	2.62	10.20	s	643	Rmet_0697	pilA	Op0196r_2	GIP-PTL-T2S
2520	TSS_2269570+3	80	13	0,1	-18	1	-37	TTGTACATCG	-16	TTTAAATTGC	-142	1,1	7.60	9.80	s	2008	Rmet_5525	0	Op1642f_1	NA
2521	TSS_2447424+3	80	2	0,1	-17	1	-36	TGGCGGCTCC	-15	GGTCTACTAC	-141	1,1	3.55	8.30	s	1557	Rmet_5690	0	Op1698f_1	NA
2522	TSS_563598-3	80	4	0,5	-6	0	-39	GTACAAGAAC	-17	GGCCCAGTCC	-145	4,1	3.42	1.90	w	61	Rmet_3993	0	Op1119r_1	NA
2523	TSS_882124-3	80	6	0,1	-15	1	-39	TCGCATGTCC	-13	CCTATACTGG	-139	1,1	7.89	3.90	m	47	Rmet_4290	0	Op1219r_1	NA
2524	TSS_157899+4	80	18	0,1	-1	0	-43	TTGATGGGTA	-14	GGTACAAACG	-144	1,1	5.34	-2.60	no	82	Rmet_6141	0	Op1877f_2	NA
2525	TSS_600814+2	80	18	0,1	-15	1	-40	TTGACCGCAC	-13	AATAATATGA	-144	1,1	7.61	4.40	(m)-sba	3239	Rmet_0564	0	Op0159f_1	NA
2526	TSS_1366129-2	80	24	0,1	-15	1	-41	TTCGGCGAAT	-13	GCGATAATGA	-142	4,1	6.56	-0.10	no	10606	Rmet_1232	yhgF	Op0356r_1	NA
2527	TSS_3631016-2	80	20	0,1	-15	1	-44	TCGAGGGCCA	-13	TGTACAGTAC	-144	1,1	5.84	-6.10	no	482	Rmet_3353	tnpA	Op0934r_1	NA
2528	TSS_2304431-3	80	15	0,1	-15	1	-38	TGCTGCGAGG	-13	TATAGACTTC	-143	1,1	4.96	3.40	m	9607	Rmet_6743	0	Op1647r_1	NA
2529	TSS_52343+5	80	14	0,1	-12	1	-30	TTGGCCAGCCC	-10	ACAAGAATCC	-142	1,1	5.61	5.10	(m)-sba	966	Rmet_6205	cnrY	Op1896f_2	NA
2530	TSS_433419+2	79	18	0,1	-15	1	-46	GCGTCTACGC	-13	CGTACAATGC	-138	5,1	8.24	-12.10	no	398	Rmet_0406	pyrC	Op0111f_1	MET-NUC-Pyr
2531	TSS_2381098+2	79	18	0,1	-16	1	-35	TTGATGGCGG	-14	ACCATACTCA	-140	1,1	6.49	11.60	s	23	Rmet_2175	sixA	Op0625f_3	NA
2532	TSS_3195529-2	79	31	0,1	-1	0	-38	TTCCGGGAAC	-12	TGGACTATGG	-146	1,1	5.11	2.90	w	6	Rmet_6566	0	Op0830r_2	NA
2533	TSS_7108+3	79	10	0,1	-17	1	-47	TCGAAAGAAC	-15	GGGAGAATCT	-140	1,1	7.26	-9.10	no	156	Rmet_5822	0	Op1760f_1	NA
2534	TSS_343697+3	79	10	0,1	-16	1	-38	TTGAACCAGC	-14	CATAGAATCT	-138	1,1	9.39	9.40	s	19	Rmet_3803	0	Op1058f_1	NA
2535	TSS_755824-3	79	10	0,1	-15	1	-34	TTGTCCGTGG	-13	AATATCATTG	-138	1,1	7.89	11.60	s	497	Rmet_4168	hpnH	Op1173r_1	NA
2536	TSS_3429768-2	79	18	0,2	-4	0	-47	TGGTTGGGGG	-13	AGCAAACTCC	-145	1,1	4.54	-14.10	no	#NV	#NV	#NV	#NV	#NV
2537	TSS_273023-3	79	14	0,1	-14	1	-41	TGCCACGAAC	-12	GGTAGACTCG	-139	1,1	7.06	-3.60	no	3842	Rmet_3726	0	Op1039r_1	NA
2538	TSS_2473435-3	79	11	0,1	-16	1	-36	GCGGCAGTGC	-14	GTTATCCTAG	-140	5,1	9.31	8.90	s	482	Rmet_5713	0	Op1709r_2	NA
2539	TSS_1836250-2	79	4	0,1	-15	1	-42	TGGTCCAATA	-13	GGTACGCTTC	-142	1,1	6.84	-3.10	no	1575	Rmet_1693	0	Op0480r_1	NA
2540	TSS_3178509-2	79	45	0,1	-16	1	-42	GCAGGGCTCA	-14	GGTAAGATCA	-146	4,1	6.10	-6.60	no	2103	Rmet_2921	dnaJ	Op0826r_2	GIP-PTL-Fold
2541	TSS_3263843-2	79	6	0,1	-16	1	-45	TTGCAAAGGA	-14	GTCACATTAC	-140	1,1	7.36	-6.60	no	115	Rmet_3007	tniR	Op0846r_1	NA
2542	TSS_1590899+3	79	16	0,1	-17	1	-35	TTCCCGTCCG	-15	CTTATGATGC	-139	1,1	7.89	10.10	s	19	Rmet_4928	lysP2	Op1444f_2	EIP-TRA-Ion
2543	TSS_171056-3	79	15	0,1	-16	1	-48	TCGTGAAAAG	-14	TGTAGACTGT	-144	1,1	6.86	-13.10	no	1101	Rmet_3633	0	Op1011r_1	NA
2544	TSS_1178677+2	78	20	0,1	-15	1	-37	TGGACGGCGG	-13	TTCAATCTGA	-142	1,1	3.89	6.90	s	328	Rmet_1087	accA	Op0313f_2	MET-CAH-Pyr
2545	TSS_271716-2	78	18	0,1	-14	1	-33	TGGCTTAATG	-12	GGAATACTTC	-144	1,1	5.75	7.10	s	3137	Rmet_0251	gpmA	Op0072r_1	MET-CAH-FbP
2546	TSS_1938729-2	78	24	0,3	-21	0	-47	GCCACAACGG	-13	GCTATAGCTC	-147	4,1	1.36	-15.60	no	59	Rmet_1787	0	Op0518r_1	NA
2547	TSS_3050227-2	78	17	0,5	-13	1	-40	TCGCTATGCA	-12	TGTCATATCT	-144	1,1	1.63	-1.10	no	203	Rmet_2802	lgt	Op0788r_2	NA
2548	TSS_3429108-2	78	6	0,1	-15	1	-36	TGGAAGTGGT	-13	TCTAAGCTTC	-141	1,1	7.35	8.90	s	1422	Rmet_R0050	0	Op0886r_1	NA
2549	TSS_3525570-2	78	9	0,1	-3	0	-22	TTGTTCGTCC	-1	CCAAGTGTCC	-145	1,1	1.01	-4.40	no	107	Rmet_3253	yadG	Op0918r_3	NA
2550	TSS_1085914-3	78	6	0,1	-20	0	-33	AAAGTCATTG	-13	GGTCTAATAG	-147	3,0	4.69	1.60	w	528	Rmet_4485	0	Op1279r_2	NA
2551	TSS_2325531-3	78	8	0,1	-15	1	-35	TCGCACCAAG	-13	GTCATACTCC	-139	1,1	6.84	9.70	s	104	Rmet_5571	0	Op1657r_2	NA
2552	TSS_959857+2	78	22	0,1	-17	1	-38	TGGTCGATGG	-15	AGTATAGTCC	-139	1,1	6.87	8.90	s	241	Rmet_0877	0	Op0255f_1	NA
2553	TSS_630610-2	78	13	0,1	-15	1	-39	TGGCTGTGAC	-13	GCCATACTAG	-143	1,1	6.74	2.90	w	264	Rmet_0587	0	Op0166r_3	NA

2554	TSS_476757+3	78	20	0,1	-16	1	-37	TGGAAGTGGT	-14	TCTAAGCTTC	-140	1,1	7.35	7.90	s	1421	Rmet_R0079	*0*	Op1098f_1	NA
2555	TSS_1410518+3	78	16	0,1	-17	1	-36	TGCCTGTGCC	-15	ATTACGATCT	-143	1,1	5.25	7.80	s	172	Rmet_4766	*0*	Op1386f_2	NA
2556	TSS_1665921-3	78	6	0,1	-15	1	-42	TTGTAGATCT	-13	GGTACCCTCG	-141	1,1	7.83	-2.60	*no*	4659	Rmet_4985	*0*	Op1463r_1	EIP-TRA-lon
2557	TSS_47090+4	78	12	0,1	-16	1	-38	GGCAAGGAGT	-14	GCTAGAATTT	-141	4,1	10.40	3.40	m	344	Rmet_6360	*traB*	Op1833f_3	NA
2558	TSS_2371098+2	78	11	0,1	-17	1	-41	GTCAGCGTTG	-15	GATACAATGT	-143	4,1	6.03	-0.10	*no*	8778	Rmet_R0034	*0*	Op0625f_1	NA
2559	TSS_2397958-2	78	16	0,1	-14	1	-38	GCGGCAATCT	-12	GGTATTGTTC	-143	5,1	9.37	2.90	w	1951	Rmet_2187	*folP*	Op0628r_2	MET-COF-Folate
2560	TSS_148416+3	78	27	0,1	-25	0	-46	GCAAAGCCGG	-23	TTAACAATCA	-143	4,1	6.48	-4.60	*no*	178	Rmet_R0070	*0*	Op1006f_1	NA
2561	TSS_151547+3	78	7	0,2	-5	0	-48	TGGTTGGGGG	-14	AGCAAACTCC	-145	1,1	4.54	-15.10	*no*	2081	Rmet_R0074	*0*	Op1006f_1	NA
2562	TSS_180058-3	78	11	0,4	-11	1	-36	GACATTCCCG	-16	GGGTATGTAC	-147	3,0	3.43	0.80	w	109	Rmet_3646	*0*	Op1017r_1	NA
2563	TSS_1753826-2	77	14	0,1	-15	1	-34	GCCCAAAAAC	-13	ATTATAATAA	-140	4,1	11.11	6.60	s	13306	Rmet_1613	*tnpA*	Op0450r_1	NA
2564	TSS_153516+3	77	27	0,1	-15	1	-26	TTGCCCGTAA	-2	ACCAGTGTGT	-147	1,1	3.77	-0.80	*no*	112	Rmet_R0074	*0*	Op1006f_1	NA
2565	TSS_225117+3	77	4	0,1	-16	1	-46	GCAAAACTAG	-14	GTAACAATAT	-146	4,1	7.24	-12.60	*no*	309	Rmet_3685	*flhD2*	Op1026f_3	EIP-SIG-2Comp
2566	TSS_478066+3	77	21	0,1	-15	1	-26	TTGCCCGTAA	-2	ACCAGTGTGT	-147	1,1	3.77	-0.80	*no*	112	Rmet_R0079	*0*	Op1098f_1	NA
2567	TSS_602480-3	77	5	0,1	-15	1	-36	TGGTGCAGTC	-13	GACACAATGG	-143	1,1	7.09	8.90	s	59	Rmet_4033	*0*	Op1137r_1	NA
2568	TSS_1271586+2	77	10	0,1	-16	1	-49	GCTGCCGGAG	-14	AGGATAATTC	-136	5,1	9.91	-18.60	*no*	502	Rmet_1161	*infC*	Op0333f_3	NA
2569	TSS_2782156+2	77	17	0,1	-16	1	-47	TTCCTACTGT	-14	TATATAGTGG	-142	1,1	7.37	-10.10	*no*	51	Rmet_6539	*0*	Op2037f_1	NA
2570	TSS_1061261-2	77	14	0,1	-24	0	-45	TTCAGCATGT	-22	AGTAGTGTGA	-144	1,1	4.54	-0.10	*no*	138	Rmet_0974	*paaH*	Op0272r_1	MET-CAH-But
2571	TSS_1878180-3	77	10	0,1	-15	1	-36	GCTGCAGCCT	-13	CGTAGAATGC	-139	5,1	11.33	7.40	s	26	Rmet_5184	*0*	Op1527r_1	NA
2572	TSS_2772441+2	76	21	0,1	-7	0	-34	CCAGCAACGC	-5	TCGACAATTT	-143	5,1	8.66	-0.80	*no*	985	Rmet_2548	*0*	Op0707f_2	NA
2573	TSS_3529938-2	76	18	0,1	-15	1	-33	TGGCGGACTG	-13	GTGACAATGT	-141	1,1	7.08	6.10	m	842	Rmet_3258	*yrbE*	Op0918r_2	EIP-TRA-ABC
2574	TSS_3860395-2	76	13	0,1	-16	1	-38	TCAGGCAAGT	-14	TGCAAAATGC	-142	4,1	8.20	4.40	m	385	Rmet_3559	*0*	Op0988r_3	NA
2575	TSS_2112040-3	76	30	0,1	-23	0	-47	CCCCCAAGGT	-21	GGAATTCTTC	-147	1,1	-0.75	-8.60	*no*	47	Rmet_5386	*0*	Op1603r_1	NA
2576	TSS_120396+5	76	21	0,4	-13	1	-47	TCGCAGTCAA	-23	TTGATATTGA	-145	1,1	4.63	-3.10	*no*	29	Rmet_6396	*0*	Op1910f_1	NA
2577	TSS_121135-5	76	20	0,1	-15	1	-46	GCAGCCCGGG	-13	GGCATTATCT	-141	5,1	8.50	-14.60	*no*	3855	Rmet_6262	*0*	Op1909r_1	NA
2578	TSS_2861014+2	76	10	0,1	-16	1	-41	TGGAACGTGC	-14	CTTATCATGC	-137	1,1	8.24	1.90	*(w)-sba*	200	Rmet_2628	*0*	Op0735f_1	NA
2579	TSS_2674011-2	76	26	0,1	-16	1	-34	TCGAGCGCGC	-14	GATAGAATCC	-139	1,1	8.55	9.10	s	101	Rmet_2462	*0*	Op0688r_5	NA
2580	TSS_3384278-2	76	15	0,1	-15	1	-49	TCGCCAATGC	-13	CCTATGATGC	-140	1,1	8.78	-14.10	*no*	29	Rmet_3122	*0*	Op0878r_3	GIP-PTL-Fold
2581	TSS_3473132-2	76	17	0,1	-16	1	-48	TGGTGACAGC	-14	GTTACATTGC	-142	1,1	7.36	-13.10	*no*	26	Rmet_3197	*0*	Op0898r_1	NA
2582	TSS_3697779-2	76	13	0,1	-16	1	-35	TTGAAAGGCC	-14	GCTACAATCA	-136	1,1	11.13	13.60	s	3	Rmet_R0065	*0*	Op0950r_2	NA
2583	TSS_152207+3	76	10	0,1	-16	1	-37	TGGAAGTGGT	-14	TCTAAGCTTC	-140	1,1	7.35	7.90	s	1421	Rmet_R0074	*0*	Op1006f_1	NA
2584	TSS_2422711+2	76	31	0,1	-12	1	-36	TTGTCATCGG	-10	GCTAGTATCT	-141	1,1	8.22	9.40	s	137	Rmet_2211	*ctpF*	Op0633f_1	EIP-TRA-lon
2585	TSS_3192134+2	76	12	0,1	-16	1	-26	TTGTACTTCT	-3	CGGATTATGT	-146	1,1	6.22	1.20	*(w)-nu*	2	Rmet_2937	*yggS*	Op0829f_1	NA
2586	TSS_1571833-2	76	17	0,1	-15	1	-34	TTGCCCGCAT	-13	TCCAGAATGA	-139	1,1	8.05	10.60	s	15921	Rmet_1434	*map*	Op0402r_1	GIP-TL-Ass
2587	TSS_1235287+3	76	25	0,2	-3	0	-37	TTGACGCCGT	-15	CCTACCATGT	-141	1,1	8.81	12.40	s	47	Rmet_4609	*bug*	Op1328f_1	NA
2588	TSS_2414401+3	76	10	0,1	-16	1	-45	TCGAAATCAG	-14	CCTAGAATAG	-139	1,1	8.09	-5.10	*no*	31	Rmet_5655	*0*	Op1688f_4	NA
2589	TSS_428994-3	76	26	0,1	-15	1	-47	TCAGGAGCAG	-13	GCTACGCTGG	-140	4,1	7.63	-14.60	*no*	4929	Rmet_3878	*0*	Op1081r_1	NA
2590	TSS_2025358-3	76	8	0,1	-15	1	-33	TTGCATTTCA	-13	CCGATAATCC	-137	1,1	8.29	8.60	s	7380	Rmet_5310	*0*	Op1569r_1	NA
2591	TSS_298215-2	75	11	0,1	-15	1	-33	TGGTTCATGC	-13	GCTAGAATTT	-140	1,1	8.33	7.10	s	274	Rmet_0281	*ftsY*	Op0082r_1	GIP-PTL-Exp
2592	TSS_2206404-2	75	18	0,1	-15	1	-35	TTCAATGTGC	-13	CATAGACTGC	-140	1,1	7.24	10.70	s	261	Rmet_2036	*0*	Op0594r_1	NA
2593	TSS_1430789+3	75	2	0,1	-16	1	-41	TCCCAATACA	-14	TTCATGATGC	-143	1,1	5.31	-0.60	*no*	8660	Rmet_4795	*0*	Op1396f_1	NA
2594	TSS_1686288+3	75	6	0,1	-16	1	-39	TTCTGCTTCC	-14	CCTAACCTTG	-142	1,1	5.08	2.90	w	90	Rmet_5007	*0*	Op1470f_1	NA
2595	TSS_2357579-3	75	7	0,1	-17	1	-46	GCCAGAGCAA	-15	TGTATCTTTC	-141	4,1	6.91	-11.60	*no*	455	Rmet_5602	*0*	Op1669r_1	NA
2596	TSS_104005+5	75	14	0,1	-16	1	-37	TCGATTTGCT	-14	GCTAAGATTG	-141	1,1	7.66	8.90	s	6764	Rmet_6258	*rhsC*	Op1906f_1	NA
2597	TSS_1460840+2	75	19	0,1	-18	1	-37	TGCAAAGCGG	-16	GCTAACCTAC	-142	1,1	5.45	7.80	s	948	Rmet_1353	*rluD*	Op0381f_1	GIP-TL-Ass
2598	TSS_3784225+2	75	18	0,1	-17	1	-47	TTGCGGAGAG	-15	AGTAGCCTGG	-141	1,1	6.99	-10.60	*no*	7946	Rmet_3502	*0*	Op0971f_1	NA
2599	TSS_251627-2	75	9	0,1	-15	1	-33	GGCGCAATCT	-13	GGTATTATCC	-137	4,1	8.36	4.60	m	88	Rmet_0234	*0*	Op0068r_1	NA
2600	TSS_2574440-2	75	23	0,1	-15	1	-34	TCGTCTATCG	-13	TGTACTATGG	-139	1,1	6.38	9.10	s	607	Rmet_2361	*0*	Op0664r_1	NA
2601	TSS_150911+3	75	12	0,1	-16	1	-38	TGGCGAACGA	-14	TGTACTCTTT	-145	1,1	6.42	5.90	m	#NV	#NV	#NV	#NV	#NV
2602	TSS_1277116+3	75	8	0,1	-14	1	-33	TGGAATCGTT	-12	ATTAATTTAC	-146	1,1	4.23	7.10	s	48	Rmet_4647	*0*	Op1342f_1	NA
2603	TSS_51371+4	75	12	0,1	-18	1	-37	TTCCTGCGCC	-16	GACATACTGG	-142	2,1	10.00	8.30	s	3292	Rmet_6003	*0*	Op1829f_1	NA
2604	TSS_228765-4	75	17	0,1	-13	1	-31	TTGATCATTC	-11	CATATTATTC	-142	1,1	8.47	7.60	s	28	Rmet_6072	*0*	Op1850r_1	NA
2605	TSS_3676240+2	75	19	0,1	-16	1	-46	TGGACCCCGC	-14	CGTATGCTGA	-139	1,1	6.78	-8.10	*no*	186	Rmet_3398	*0*	Op0943f_1	NA
2606	TSS_3412327-2	75	28	0,1	-15	1	-38	TTCCCTTGGT	-13	CGCATAATCC	-141	1,1	8.26	6.90	s	109	Rmet_3148	*fadD*	Op0882r_3	MET-LIP-FAMet
2607	TSS_3644166-2	75	11	0,1	-16	1	-39	CTCCGATTTG	-14	GCTATATTCC	-145	1,1	3.83	2.90	w	1259	Rmet_3367	*0*	Op0936r_1	NA
2608	TSS_1166191+3	75	20	0,1	-24	0	-48	TTGCCCAGAA	-22	CCGACGCTGT	-142	1,1	5.48	-5.60	*no*	213	Rmet_4554	*0*	Op1306f_3	NA
2609	TSS_126192+5	75	15	0,1	-17	1	-35	TGGATATCCT	-15	CTTACAATCA	-141	1,1	8.39	11.10	s	4421	Rmet_6274	*0*	Op1914f_1	NA
2610	TSS_2232866+2	74	12	0,1	-16	1	-39	TTGCTCCATA	-14	GGCAAGCTGG	-140	1,1	6.03	3.40	m	3427	Rmet_2063	*0*	Op0599f_1	NA
2611	TSS_3144420+2	74	22	0,1	-16	1	-48	TGGCCGCCTG	-14	GGTATGATTC	-136	1,1	8.83	-12.10	*no*	66	Rmet_2889	*dut*	Op0811f_1	MET-NUC-Pyr
2612	TSS_3215781+2	74	13	0,1	-17	1	-38	TGGCGCATCC	-15	GGTATACTGG	-135	1,1	9.25	7.90	s	112	Rmet_2957	*gst-1*	Op0833f_2	MET-OAA-GSH
2613	TSS_3710590+2	74	12	0,4	-18	1	-46	TTGATTTTGT	-14	CTTAAACTGG	-143	1,1	8.92	-7.60	*no*	192	Rmet_3433	*ycbL*	Op0953f_1	MET-CAH-Pyr
2614	TSS_942916-2	74	7	0,1	-23	0	-41	AAATCTGTCA	-21	TGTAAGCTGT	-143	3,1	11.30	-1.20	*no*	28	Rmet_0863	*0*	Op0248r_2	NA

2615	TSS_2907057-2	74	21	0,1	-15	1	-48	TCAGCCGCGC	-13	GCTAAAATAC	-138	5,1	10.65	-14.60	no	50	Rmet_2672	queC	Op0756r_4	NA
2616	TSS_1291058-3	74	17	0,1	-15	1	-47	TTGAAGATCC	-13	TGTACGGTAT	-143	1,1	6.52	-11.60	no	4249	Rmet_4654	0	Op1345r_1	NA
2617	TSS_1784536+2	74	8	0,1	-17	1	-42	TTGTACCAAT	-15	GTCATAATGC	-139	1,1	9.34	1.40	(w)-sba	6257	Rmet_1656	yqjG	Op0467f_1	GIP-PTL-Fold
2618	TSS_752140-2	74	6	0,1	-17	1	-49	GCTGCCCCGC	-15	GGTAGACTCG	-137	5,1	10.04	-18.60	no	45	Rmet_0679	0	Op0190r_1	NA
2619	TSS_2187011-2	74	13	0,1	-15	1	-37	TGGCAGGCGC	-13	CGTAGAATCG	-138	1,1	8.49	7.90	s	1196	Rmet_2021	0	Op0590r_2	NA
2620	TSS_689199+3	74	6	0,1	-18	1	-49	TGCCGCCCTC	-16	GTTACAATGC	-139	1,1	8.18	-14.60	no	90	Rmet_4119	hmyF	Op1160f_1	NA
2621	TSS_1240994+3	74	13	0,1	-18	1	-40	TGCCGGTATT	-16	GCTAGAATCG	-140	1,1	8.19	3.40	(m)-sba	862	Rmet_4616	0	Op1330f_1	NA
2622	TSS_126556-3	74	15	0,1	-15	1	-34	TGGACATCCC	-13	CCTAATCTTT	-142	1,1	6.31	10.10	s	70	Rmet_5925	0	Op1799r_1	NA
2623	TSS_182181-3	74	8	0,1	-15	1	-40	GCCGCAAAGC	-13	ATTACCATGC	-139	4,1	9.19	-1.60	no	2232	Rmet_3646	0	Op1017r_1	NA
2624	TSS_789433-3	74	11	0,1	-15	1	-41	TTCTCATGCC	-13	GGGAAAATGC	-140	1,1	7.68	0.90	(w)-sba	1900	Rmet_4202	0	Op1191r_1	NA
2625	TSS_1876333-3	74	18	0,1	-16	1	-47	TTGTAAGGAC	-14	CCTATACTCT	-141	1,1	8.99	-8.60	no	425	Rmet_5181	0	Op1525r_1	NA
2626	TSS_117854+5	74	4	0,1	-29	0	-27	CAAATCATCG	-7	TATGTACAGA	-148	3,0	3.89	-6.40	no	2571	Rmet_6396	0	Op1910f_1	NA
2627	TSS_56237-5	74	9	0,1	-15	1	-35	TTGTACGGCG	-13	GATACGATCA	-136	1,1	7.66	11.20	s	4091	Rmet_6203	chrB1	Op1895r_1	NA
2628	TSS_759904+2	74	6	0,1	-16	1	-46	TTCACACGGC	-14	GTTAATATAC	-140	1,1	8.00	-7.10	no	103	Rmet_0686	lolA	Op0193f_1	NA
2629	TSS_3840967+2	74	13	0,1	-16	1	-46	TTCTGATGGG	-14	GCTATAATCG	-139	1,1	9.86	-7.10	no	73	Rmet_3543	0	Op0983f_2	NA
2630	TSS_2384450-2	74	18	0,1	-15	1	-33	TGGCGATGTG	-13	CATAGAATCG	-142	1,1	8.29	8.10	s	99	Rmet_2177	ppx	Op0626r_1	MET-NUC-Pur
2631	TSS_3009887-2	74	22	0,1	-15	1	-38	TTCCCGGCGG	-13	CATATCGTGT	-143	1,1	5.03	5.90	m	59	Rmet_2767	mrp	Op0776r_1	DIV-Division
2632	TSS_1652697+3	74	2	0,1	-17	1	-46	TTGCAAAGGA	-15	GTCACATTAC	-140	1,1	7.36	-7.60	no	114	Rmet_4975	tniR	Op1460f_1	NA
2633	TSS_590801+2	73	15	0,1	-15	1	-34	TTGAACGGGG	-13	CCTATAGTCG	-140	1,1	8.61	11.60	s	91	Rmet_0553	fdsG	Op0155f_1	MET-CAH-DiCarb
2634	TSS_1148401+2	73	19	0,1	-17	1	-44	TCCAGAGAGG	-15	GATATCATCG	-143	1,1	6.87	-3.60	no	1464	Rmet_1056	ftsB	Op0301f_3	DIV-Division
2635	TSS_3338552+2	73	10	0,1	-16	1	-44	GCGTCCTGCG	-14	GATACTATCG	-141	5,1	7.16	-8.10	no	529	Rmet_3084	rdgC	Op0867f_1	GIP-REP-Recomb
2636	TSS_3365265+2	73	27	0,1	-13	1	-40	TTTCTGCGCC	-11	TCTACTTTCA	-144	2,1	8.76	-2.10	no	517	Rmet_3107	ispB	Op0875f_1	NA
2637	TSS_897607-2	73	9	0,1	-16	1	-34	TTCATTATGT	-14	GGAATAATTT	-144	1,1	7.21	9.10	s	48	Rmet_0823	bug	Op0232r_1	NA
2638	TSS_2769757-2	73	14	0,1	-15	1	-45	TTGGACATTTC	-13	GCTATGATGT	-138	1,1	9.82	-5.10	no	45	Rmet_2544	0	Op0704r_1	NA
2639	TSS_2965991-2	73	19	0,1	-17	1	-33	TTGCGGGGGG	-2	GCTATGATGG	-143	1,1	10.07	1.20	(w)-ba	77	Rmet_2729	0	Op0770r_1	NA
2640	TSS_219925+3	73	39	0,5	-16	1	-39	TCGCCCTTCA	-14	GCCGTAATTA	-146	1,1	1.47	3.90	m	34	Rmet_3681	cheW	Op1026f_2	DIV-MOT-Chemotax
2641	TSS_1974398-3	73	5	0,1	-16	1	-34	TTCTGCCTGG	-14	GCTACACTTC	-138	1,1	7.67	7.10	s	4736	Rmet_5270	0	Op1553r_1	NA
2642	TSS_214136+2	73	13	0,1	-17	1	-48	TTGAGCATTC	-15	TGTACAGTGT	-139	1,1	8.61	-10.60	no	249	Rmet_0205	aptA	Op0061f_1	MET-CAH-Prop
2643	TSS_2626751+2	73	23	0,1	-16	1	-38	TTGACCTTGA	-14	TAGACCTTGC	-143	1,1	4.83	7.40	s	4795	Rmet_6524	0	Op2034f_1	NA
2644	TSS_3276932+2	73	20	0,1	-15	1	-40	TTGAATGTGG	-13	CAGATAATTC	-144	1,1	8.15	4.40	(m)-sba	7694	Rmet_3028	0	Op0849f_1	NA
2645	TSS_2286170-2	73	19	0,1	-15	1	-45	TTCAACCTAA	-13	CTTACGATCT	-143	1,1	6.96	-8.10	no	648	Rmet_2103	yfgL	Op0608r_1	NA
2646	TSS_2394713-2	73	11	0,1	-18	1	-47	GCTGCCCGAT	-16	GCGATGGTCG	-143	5,1	6.78	-14.60	no	1228	Rmet_2185	pstS	Op0628r_4	EIP-TRA-ABC
2647	TSS_3384038-2	73	14	0,1	-16	1	-49	TTGCTCCGCG	-14	AGTACGATGC	-143	2,1	11.13	-14.60	no	449	Rmet_3121	lpxC	Op0878r_4	MET-GLYC-LPS
2648	TSS_3614341-2	73	13	0,1	-15	1	-36	TGGAAGTGGT	-13	TCTAAGCTTC	-141	1,1	7.35	8.90	s	1422	Rmet_R0060	0	Op0930r_1	NA
2649	TSS_3774803-2	73	9	0,1	-14	1	-32	TCTGAAAGGC	-12	AGTACAATCG	-135	5,1	10.81	5.60	m	400	Rmet_3488	0	Op0970r_4	EIP-TRA-ABC
2650	TSS_1087446+3	73	12	0,1	-16	1	-35	TTGTGGCTGA	-14	CTTAGAATCA	-145	1,1	7.96	11.60	s	5022	Rmet_6669	0	Op2073f_1	NA
2651	TSS_18350-5	73	12	0,1	-16	1	-35	TGGACCGCGC	-14	GCGAGAATCC	-138	1,1	7.27	11.10	s	2016	Rmet_6326	0	Op1921r_1	NA
2652	TSS_735681+2	73	8	0,1	-16	1	-24	TTGATTTTAA	-3	AAGAGCCTGC	-144	1,1	3.89	-1.40	no	556	Rmet_0669	pilG	Op0187f_1	GIP-PTL-T2S
2653	TSS_475461+3	73	11	0,1	-16	1	-38	TGGCGAACGA	-14	TGTACTCTTT	-145	1,1	6.42	5.90	m	#NV	#NV	#NV	#NV	#NV
2654	TSS_706169-3	73	18	0,1	-15	1	-39	TTGTCCGTCC	-13	CCTATACTGG	-137	1,1	8.94	6.40	s	59	Rmet_4131	0	Op1165r_2	NA
2655	TSS_729092-3	73	9	0,1	-12	1	-44	GGCAGCGAAC	-10	GTTAATCTGA	-145	4,1	7.32	-14.60	no	22	Rmet_4153	tnpA	Op1169r_1	NA
2656	TSS_514594-2	72	17	0,1	-15	1	-43	GCGGAAAGGC	-13	GCTATGATAG	-135	5,1	10.79	-6.10	no	56	Rmet_0486	moaC	Op0130r_1	MET-COF-Folate
2657	TSS_2773662-2	72	19	0,1	-15	1	-43	TCGCTCCTAC	-13	CATATGCTCG	-142	1,1	5.41	-5.10	no	96	Rmet_6533	0	Op2035r_1	NA
2658	TSS_3427799-2	72	27	0,1	-14	1	-25	TTGCCCGTAA	-1	ACCAGTGTGT	-147	1,1	3.77	-1.80	no	113	Rmet_R0050	0	Op0886r_1	NA
2659	TSS_3743069-2	72	8	0,1	-15	1	-37	GCGGCTGGCG	-13	CGTATGATCG	-134	5,1	9.48	5.90	m	26	Rmet_3465	0	Op0964r_3	NA
2660	TSS_1621096+3	72	16	0,2	-5	0	-35	TTGCAAACCC	-14	CATAGTATAC	-142	1,1	7.37	11.60	s	120	Rmet_4955	appC	Op1456f_1	MET-EN-OxPhos
2661	TSS_897460-3	72	13	0,1	-6	0	-32	TTGCAATGAC	-4	TATATGATCT	-140	1,1	9.64	4.20	(m)-ba	61	Rmet_4302	0	Op1225r_1	NA
2662	TSS_124862+2	72	13	0,1	-16	1	-46	TTCCGCAATC	-14	GCCAAAATAC	-139	1,1	7.49	-10.10	no	31	Rmet_0118	ycdW	Op0031f_5	NA
2663	TSS_1094890+2	72	18	0,1	-17	1	-35	TTGAATACGG	-15	GCCATACTCG	-137	1,1	8.56	10.60	s	376	Rmet_1004	grpE	Op0287f_1	GIP-PTL-Fold
2664	TSS_2937778+2	72	18	0,1	-29	0	-47	TGGCCTCTCG	-27	GCTACAATCC	-135	1,1	9.51	-0.70	no	149	Rmet_2704	trmB	Op0765f_2	NA
2665	TSS_2909971-2	72	16	0,1	-14	1	-37	TTCATCGTGG	-12	GCTACCATGA	-142	1,1	7.43	6.90	s	128	Rmet_2675	tolB	Op0756r_3	NA
2666	TSS_1012762+2	72	13	0,1	-16	1	-46	TCGACGTGCG	-14	TTTATCCTGT	-143	1,1	6.73	-8.10	no	150	Rmet_0928	nuoB	Op0267f_3	MET-EN-OxPhos
2667	TSS_1049760+2	72	17	0,1	-17	1	-43	TTGCGCGTGC	-15	GGTACGATCG	-137	1,1	9.58	-1.60	no	375	Rmet_0966	0	Op0269f_1	NA
2668	TSS_1915540+2	72	27	0,1	-16	1	-40	TCGGCAAGAC	-14	TTTATGCTTC	-137	5,1	9.99	3.90	(m)-sba	36	Rmet_1764	0	Op0505f_1	NA
2669	TSS_2414177-2	72	13	0,1	-2	0	-35	TTGCCAGATC	-14	CCTAATATTT	-140	1,1	8.63	12.60	s	58	Rmet_2202	abmA	Op0630r_2	MET-XEN-Others
2670	TSS_607713+3	72	13	0,1	-15	1	-34	TCGACTTCTC	-13	AGCAAACTGG	-144	1,1	5.84	9.10	s	58	Rmet_4041	0	Op2064f_1	NA
2671	TSS_2249288+3	72	20	0,1	-16	1	-36	TTGCATACGT	-14	GCCAGAATGC	-138	1,1	9.26	11.40	s	4815	Rmet_5509	0	Op1640f_1	NA
2672	TSS_2226314-3	72	8	0,1	-15	1	-36	TTGACCGACC	-13	GTTAAAATTG	-135	1,1	11.13	13.40	s	1097	Rmet_5481	tnmB	Op2095r_1	NA
2673	TSS_1272135+2	71	11	0,1	-15	1	-36	TTGATAACGA	-13	GTCAAATTCA	-141	1,1	6.47	11.40	s	586	Rmet_1162	rpmI	Op0333f_3	GIP-TL-Ribo
2674	TSS_2817556+2	71	5	0,1	-15	1	-45	CCTGCCACAC	-13	GGTAAGATCG	-136	5,1	10.35	-12.60	no	33	Rmet_2591	0	Op0723f_2	NA
2675	TSS_2295150+3	71	13	0,1	-17	1	-35	TTTTTTCGCG	-15	CCTAGAATTG	-140	2,1	13.02	9.10	s	30	Rmet_5548	0	Op1648f_2	NA

2676	TSS_66870-3	71	19	0,1	-10	1	-43	GAATTCCCTG	-23	CCGTTTGTTG	-143	3,0	6.81	-6.20	no	476	Rmet_5870	0	Op1777r_1	NA	
2677	TSS_245037-3	71	3	0,1	-15	1	-48	GCCAGACAGC	-13	GGTATGCTTC	-138	4,1	9.74	-17.60	no	30196	Rmet_3677	stpA	Op1025r_1	NA	
2678	TSS_2150156-2	71	9	0,1	-15	1	-41	TGGAAAACGC	-13	TGCATACTGT	-142	1,1	7.69	0.90	(w)-sba	5640	Rmet_1967	aatA	Op0574r_1	NA	
2679	TSS_3654025-2	71	7	0,4	-16	1	-39	TTTTGTTTCC	-8	AATAACATCG	-143	2,1	10.03	-2.10	no	212	Rmet_3377	0	Op0938r_1	NA	
2680	TSS_258772+3	71	12	0,1	-16	1	-48	TTGAAAAACT	-14	AATAAAATTT	-142	1,1	10.42	-9.60	no	32	Rmet_3719	0	Op1034f_1	NA	
2681	TSS_2116638+3	71	20	0,1	-17	1	-42	TTCACCGACA	-15	GCGATGATCG	-139	1,1	6.38	0.90	(w)-sba	1250	Rmet_5392	0	Op1606f_1	EIP-SIG-2Comp	
2682	TSS_142869+5	71	17	0,1	-16	1	-36	TGGCTGTAAC	-14	GCTAACATTA	-140	1,1	7.54	9.90	s	14888	Rmet_6302	trbB	Op1918f_1	NA	
2683	TSS_1175035+2	71	8	0,1	-16	1	-36	TTGCTCATAG	-14	TCGATGATGC	-142	1,1	6.99	11.40	s	1565	Rmet_1085	cysS	Op0313f_1	MET-AA-Cys	
2684	TSS_152543+3	71	16	0,1	-16	1	-30	CCGTAACTAT	-5	GCGAAATTCC	-147	5,1	5.26	-1.30	no	1085	Rmet_R0074	0	Op1006f_1	NA	
2685	TSS_1704998+3	71	4	0,1	-18	1	-44	TTGCTCCTCC	-16	AGTAGAATCG	-135	1,1	8.97	-1.60	no	51	Rmet_5022	0	Op1478f_1	NA	
2686	TSS_3354496+2	70	7	0,4	-1	0	-37	TGGACATGGG	-16	GCCACGCTGC	-139	1,1	6.56	9.30	s	6416	Rmet_3101	nudH	Op0873f_1	NA	
2687	TSS_3605811+2	70	22	0,1	-16	1	-35	TGGCCAGGAC	-14	GGTAGTCTTC	-140	1,1	7.23	10.10	s	12205	Rmet_6590	0	Op0931f_1	NA	
2688	TSS_1010899-2	70	14	0,1	-15	1	-48	TTGTCTCACA	-13	GCTATTGTAG	-144	1,1	5.80	-12.60	no	5829	Rmet_0919	0	Op0264r_1	NA	
2689	TSS_1049863-2	70	8	0,1	-9	0	-37	TTTAGCCTCG	-7	TCCACAATGC	-143	2,1	10.32	1.90	(w)-ba	44793	Rmet_0919	0	Op0264r_1	NA	
2690	TSS_1237621-2	70	17	0,1	-2	0	-34	TTTCTCCTCG	0	AATATAATAC	-138	2,1	12.75	-1.30	no	14	Rmet_1130	wrbA	Op0322r_1	NA	
2691	TSS_299415+3	70	10	0,1	-16	1	-49	TAGAGGTAAC	-14	GGCATCATGT	-146	1,1	4.40	-15.10	no	334	Rmet_3760	0	Op1046f_2	NA	
2692	TSS_425296+3	70	8	0,1	-16	1	-46	TCGTGAAGAA	-23	CTTATCTTGT	-146	1,1	4.71	-0.10	no	312	Rmet_3881	ompR	Op1082f_2	NA	
2693	TSS_857879+3	70	17	0,1	-17	1	-44	TGGCGAGCGT	-15	GCTATTATGG	-139	1,1	8.94	-3.10	no	871	Rmet_4268	citA	Op1212f_1	MET-CAH-TCA	
2694	TSS_2011885-3	70	21	0,1	-16	1	-49	TTGCTGTCAT	-14	CATAGAATGG	-138	1,1	9.25	-13.60	no	38	Rmet_5308	0	Op1569r_1	NA	
2695	TSS_2301681-3	70	1	0,1	-15	1	-33	GCAAAAGATT	-13	GTTACCATCG	-141	4,1	8.28	3.60	m	6857	Rmet_6743	0	Op1647r_1	NA	
2696	TSS_212251+4	70	14	0,1	-16	1	-36	TTCACGTAAT	-14	AGTACGCTGG	-138	1,1	7.14	10.90	s	459	Rmet_6387	0	Op1853f_1	NA	
2697	TSS_1091832+2	70	15	0,1	-14	1	-34	TTGCGGTTTC	-12	CACATCCTGT	-140	1,1	5.77	9.20	s	225	Rmet_1000	0	Op0285f_1	NA	
2698	TSS_3237028+2	70	9	0,1	-16	1	-34	TTCATTGAAT	-14	GCTATAGTGC	-143	1,1	7.99	9.10	s	51	Rmet_2978	0	Op0839f_1	NA	
2699	TSS_37940-2	70	17	0,1	-16	1	-45	TCGGCCACCT	-14	GTTACAGTTC	-137	5,1	9.96	-7.10	no	42	Rmet_0035	minC	Op0012r_1	DIV-Division	
2700	TSS_2491658-2	70	6	0,1	-15	1	-49	TCGGCCCGAT	-13	GCGACAATCT	-138	5,1	9.94	-16.10	no	234	Rmet_2269	0	Op0648r_2	GIP-REP-Recomb	
2701	TSS_3297441-2	70	23	0,1	-2	0	-33	TTGAAGTCTG	0	CTTAGGCTCA	-142	1,1	6.37	-1.80	no	133	Rmet_3041	0	Op0854r_1	NA	
2702	TSS_3328849-2	70	13	0,1	-23	0	-47	CCGTCCGCAG	-21	CGCACTGTGG	-144	5,1	3.59	-9.10	no	332	Rmet_3071	0	Op0862r_3	NA	
2703	TSS_2225875+3	70	17	0,1	-16	1	-47	TTGAACTCGT	-14	CCTATACTGA	-139	1,1	9.86	-8.60	no	78	Rmet_5483	dam	Op1628f_1	GIP-REP-Recomb	
2704	TSS_588580-3	70	4	0,1	-15	1	-34	TGGACAGATC	-13	GTGACAATCC	-141	1,1	8.08	11.10	s	26	Rmet_4017	pobR	Op1127r_1	NA	
2705	TSS_2175560-3	70	2	0,1	-16	1	-34	TGGCATTGTC	-14	GGTATAGTCC	-140	1,1	8.49	8.10	s	51	Rmet_5428	0	Op1615r_2	NA	
2706	TSS_3271933+2	70	15	0,1	-16	1	-47	TTGAGAAAGC	-14	TGTACGATCT	-145	1,1	9.54	-8.60	no	123	Rmet_3013	yodB	Op0847f_1	NA	
2707	TSS_3580748+2	70	16	0,1	-23	0	-41	TTGCCCGGGA	-21	CGGATCCTGT	-139	1,1	5.84	4.80	m	4925	Rmet_3322	0	Op0927f_1	NA	
2708	TSS_403343-2	70	9	0,1	-15	1	-34	TTGCGGATTT	-13	TTGAACATCT	-138	1,1	6.13	8.60	s	15498	Rmet_0365	coxS	Op0102r_1	MET-EN-Methane	
2709	TSS_1503420-2	70	26	0,1	-15	1	-34	TTGATGCAGC	-13	GCGATCATGG	-137	1,1	7.50	10.60	s	1977	Rmet_1391	0	Op0388r_1	NA	
2710	TSS_3430673-2	70	17	0,1	-27	0	-22	ACACTCGTTA	-2	CGAACAAGTG	-147	3,0	5.73	-11.40	no	#NV	#NV	#NV	#NV	#NV	
2711	TSS_1563682+3	70	14	0,1	-16	1	-35	TTGAAGAACA	-14	GCTACTCTTT	-146	1,1	8.19	10.60	s	13	Rmet_4900	0	Op1434f_2	NA	
2712	TSS_2045209+3	70	16	0,1	-16	1	-49	GCACGGATCG	-14	TGGACAATGC	-139	4,1	7.24	-20.60	no	903	Rmet_5334	0	Op1580f_1	EIP-TRA-Other	
2713	TSS_1364901-3	70	11	0,1	-15	1	-20	TGGCCGGCGT	0	CGTATTCTTC	-140	1,1	6.93	-5.90	no	296	Rmet_6681	0	Op1363r_2	NA	
2714	TSS_1872877-3	70	5	0,1	-15	1	-35	TTGCGGAGAC	-13	GCGAGACTGT	-139	1,1	7.30	10.20	s	3570	Rmet_5171	cspA	Op1521r_1	NA	
2715	TSS_2354194+2	69	3	0,1	-16	1	-49	TTGATCTACG	-14	GCGACCATGC	-143	1,1	6.99	-14.60	no	72	Rmet_2155	0	Op0621f_1	NA	
2716	TSS_575863-2	69	11	0,1	-16	1	-38	TTCAGACAGC	-14	GATAGGGTTT	-144	1,1	5.48	6.90	s	4052	Rmet_0537	exbB2	Op0148r_1	NA	
2717	TSS_1830453-2	69	15	0,1	-15	1	-34	TTGACCCAGT	-13	AGGACGCTTG	-144	1,1	5.75	9.60	s	2709	Rmet_1687	0	Op0476r_1	NA	
2718	TSS_3430404-2	69	11	0,1	-15	1	-37	TGGCGAACGA	-13	TGTACTCTTT	-145	1,1	6.42	6.90	s	#NV	#NV	#NV	#NV	#NV	
2719	TSS_3655397-2	69	22	0,4	-4	0	-33	TTGCGCATTT	-12	GCTAAATTTG	-142	1,1	9.18	8.60	s	39	Rmet_3379	0	Op0940r_3	NA	
2720	TSS_551784+2	69	13	0,1	-16	1	-38	TTGCAAAGCC	-14	ACTATCATGT	-140	1,1	9.55	9.40	s	730	Rmet_0516	envZ	Op0139f_1	EIP-SIG-2Comp	
2721	TSS_67614-2	69	18	0,1	-14	1	-35	TTGCCCTGCC	-12	TATATATTCT	-143	1,1	8.67	12.20	s	60	Rmet_0058	gcvA	Op0018r_1		0
2722	TSS_92215-2	69	14	0,1	-16	1	-34	TTGCATCGCA	-14	GGCATAGTTA	-142	1,1	6.97	8.60	s	3067	Rmet_0081	mhpD2	Op0022r_1	NA	
2723	TSS_3428772-2	69	4	0,1	-15	1	-29	CCGTAACTAT	-4	GCGAAATTCC	-147	5,1	5.26	-2.30	no	1086	Rmet_R0050	0	Op0886r_1	NA	
2724	TSS_3615637-2	69	9	0,1	-15	1	-37	TGGCGAACGA	-13	TGTACTCTTT	-145	1,1	6.42	6.90	s	#NV	#NV	#NV	#NV	#NV	
2725	TSS_1268171+3	69	4	0,1	-16	1	-44	TCGAGATGCC	-14	CCTAAACTAC	-142	1,1	7.41	-4.10	no	35	Rmet_4637	0	Op1340f_1	NA	
2726	TSS_195615+4	69	12	0,1	-17	1	-36	TGGACGTGGT	-15	ATTACACTCA	-140	1,1	7.93	11.30	s	22	Rmet_6106	copT	Op1863f_1	NA	
2727	TSS_200246+2	69	18	0,1	-20	0	-35	CCTGAACTAT	-13	CGTACCAACG	-144	5,1	3.62	4.20	m	1836	Rmet_0193	0	Op0057f_1	GIP-PTL-T2S	
2728	TSS_3158536+2	69	10	0,1	-19	0	-38	TCGCGGCAAC	-17	CCGAGCGTCT	-143	1,1	1.42	5.30	m	1	Rmet_2905	mviN	Op0821f_2	EIP-TRA-Ion	
2729	TSS_3792116+2	69	2	0,1	-16	1	-42	TTCAAACCGC	-14	GCTAACATCA	-142	1,1	7.92	-0.10	no	55	Rmet_3502	0	Op0971f_1	NA	
2730	TSS_1856583-2	69	4	0,1	-15	1	-39	CCTGCTACGC	-13	GCTATGGTTC	-141	5,1	7.40	-0.60	no	77	Rmet_1714	0	Op0484r_2	NA	
2731	TSS_383820+3	69	13	0,1	-17	1	-36	TGGCCGGCAG	-15	GGTATGTTCT	-142	1,1	6.47	10.30	s	31	Rmet_3840	0	Op1066f_2	NA	
2732	TSS_620379-3	69	10	0,1	-15	1	-46	TGGCACATGT	-13	CGTAGAATGG	-136	1,1	9.38	-10.10	no	24	Rmet_4053	0	Op1141r_3	NA	
2733	TSS_58092+2	68	28	0,1	-16	1	-36	TCGTAACAAT	-14	ATTAGGCTAC	-145	1,1	4.43	9.90	s	394	Rmet_0051	mreB	Op0017f_1	DIV-Division	
2734	TSS_759885-2	68	12	0,1	-16	1	-42	TTCCGCAGCG	-14	GGTATATTAA	-139	2,1	10.28	-1.10	no	2648	Rmet_0684	trxB	Op0192r_1	MET-NUC-Pyr	
2735	TSS_825719-2	68	10	0,1	-14	1	-34	TTGACCAGGC	-12	GCGACAGTTG	-137	1,1	7.36	11.20	s	367	Rmet_0747	0	Op0212r_1	NA	
2736	TSS_3803788-2	68	28	0,1	-15	1	-45	TTGCTTCATC	-13	TAGACAATGG	-142	1,1	7.14	-7.60	no	1048	Rmet_3511	0	Op0974r_6	EIP-TRA-ABC	

2737	TSS_1161087-3	68	14	0,1	-24	0	-45	TGCCAATAGG	-22	GCTATACTGT	-141	1,1	8.65	0.40	w	290	Rmet_4549	uvrA2	Op1305r_1	GIP-REP-Recomb
2738	TSS_2248156-3	68	14	0,1	-15	1	-46	TGGATACCGG	-13	TGTACGATCG	-142	1,1	7.39	-9.10	no	5502	Rmet_5498	0	Op1635r_1	NA
2739	TSS_4712-5	68	21	0,4	-14	1	-27	TTGCGCTTCG	-6	AATACTGTTT	-147	1,1	5.36	1.60	(w)-nu	18812	Rmet_6301	trbF	Op1917r_1	NA
2740	TSS_2350841+2	68	12	0,1	-15	1	-41	TGACGCCTCG	-13	CTTATAATTC	-143	4,1	6.52	-0.60	no	27	Rmet_2153	0	Op0621f_2	NA
2741	TSS_2361136-2	68	12	0,1	-9	0	-28	TTCAACTCGT	-7	CTTACGATGA	-142	1,1	7.45	3.10	(w)-nu	3714	Rmet_6509	0	Op2029r_1	NA
2742	TSS_2292930+3	68	12	0,1	-16	1	-45	TCAGACGGCG	-14	CGTAAAATTG	-142	5,1	10.36	-8.60	no	802	Rmet_6742	0	Op2098f_1	NA
2743	TSS_2543359+3	68	14	0,1	-17	1	-38	CCGGCCGCTT	-15	CGTACAATGC	-136	5,1	11.16	5.90	m	20	Rmet_5785	0	Op1740f_1	NA
2744	TSS_1061500-3	68	8	0,1	-14	1	-43	TTGATATAGC	-12	AGTACAATGC	-141	1,1	10.95	-1.60	no	51	Rmet_4464	0	Op1273r_1	NA
2745	TSS_2175431-3	68	15	0,1	-15	1	-34	CCGGCCAGGG	-13	CCTATCATGT	-139	5,1	10.52	7.10	s	2813	Rmet_6725	0	Op2091r_1	NA
2746	TSS_364773+2	68	7	0,1	-15	1	-43	TGGCGACGAC	-13	GGTACGCTAG	-139	1,1	6.96	-5.10	no	26	Rmet_0346	0	Op0097f_2	NA
2747	TSS_1577091+2	68	5	0,1	-20	0	-45	GGCGCCGAGC	-18	CGCATTGTCG	-141	4,1	3.69	-8.60	no	87	Rmet_1455	ybbK	Op0405f_1	NA
2748	TSS_3591113+2	68	9	0,1	-17	1	-40	TTCAACGTTA	-15	GGCAGAATGT	-141	1,1	7.69	3.90	(m)-sba	3000	Rmet_3329	0	Op0929f_1	NA
2749	TSS_3620630+2	68	7	0,1	-17	1	-36	TTGCGACGCA	-15	AGTACAGTGA	-141	1,1	7.68	11.80	s	248	Rmet_3344	0	Op0931f_4	NA
2750	TSS_1816110-2	68	8	0,1	-15	1	-35	TTCCCAAGCT	-13	GTCACAATTG	-141	1,1	8.75	11.70	s	6586	Rmet_1672	0	Op0470r_1	NA
2751	TSS_1179126+3	68	21	0,1	-16	1	-44	TGGATTTTTT	-14	TGTACACTTT	-140	1,1	7.92	-5.10	no	9529	Rmet_4574	0	Op1316f_1	MET-CAH-Prop
2752	TSS_573302-3	68	11	0,5	-19	0	-35	TTGACAGCAC	-14	TTAATCATCT	-140	1,1	6.29	13.60	s	156	Rmet_4001	rpoK	Op1123r_1	GIP-TK-RNAP
2753	TSS_48899+2	67	3	0,1	-16	1	-37	TGCGCGACGC	-14	GTCATACTCG	-139	4,1	6.64	7.40	s	33	Rmet_0044	0	Op0015f_3	NA
2754	TSS_1185753+2	67	16	0,1	-16	1	-34	TCGCCAGCGG	-14	GGTATCATTC	-138	1,1	8.94	10.10	s	1980	Rmet_1092	yflS	Op0315f_1	EIP-TRA-Ion
2755	TSS_1711238-2	67	17	0,1	-16	1	-35	TGGAACAGCT	-14	CGTATAGTCG	-139	1,1	7.82	11.10	s	267	Rmet_1587	prpB	Op0438r_1	MET-CAH-Prop
2756	TSS_2581965-2	67	20	0,1	-2	0	-34	TTGTATTTAA	0	AATAAAATAT	-146	1,1	8.15	-0.80	no	108	Rmet_2369	0	Op0668r_1	EIP-TRA-Ion
2757	TSS_567590+3	67	13	0,1	-16	1	-37	TTCCCACGCC	-14	GATAACATTG	-138	1,1	8.07	9.90	s	5566	Rmet_6639	0	Op2062f_1	NA
2758	TSS_2128897+3	67	6	0,1	-16	1	-41	TTCAGGTGCA	-14	GCGATCATGC	-138	1,1	6.56	0.90	(w)-sba	53	Rmet_5400	rpoM	Op1608f_1	GIP-TK-RNAP
2759	TSS_36495-5	67	6	0,1	-15	1	-48	TTGCCGGATG	-13	TGTAGTATCG	-140	1,1	8.10	-12.60	no	4781	Rmet_6344	merR	Op1929r_1	NA
2760	TSS_651493+2	67	3	0,1	-14	1	-35	TTGCGCTGCC	-12	TAGAAGATAG	-140	1,1	5.57	9.20	s	66	Rmet_0602	0	Op0171f_1	NA
2761	TSS_944474+2	67	12	0,1	-16	1	-45	TCGAATGCTT	-14	ATTACGATCT	-141	1,1	6.43	-7.10	no	97	Rmet_0865	0	Op0249f_1	NA
2762	TSS_1655912+2	67	4	0,1	-16	1	-47	GCACGGAAAT	-14	CGTAACCTCA	-139	4,1	7.52	-17.60	no	23	Rmet_1536	hypB2	Op0427f_4	GIP-PTL-Fold
2763	TSS_2904628-2	67	11	0,1	-15	1	-35	TCGATCTGAT	-13	CCCATACTGG	-142	1,1	6.43	10.70	s	1170	Rmet_2669	0	Op0756r_5	NA
2764	TSS_3613032-2	67	15	0,1	-14	1	-25	TTGCCCGTAA	-1	ACCAGTGTGT	-147	1,1	3.77	-1.80	no	113	Rmet_R0060	0	Op0930r_1	NA
2765	TSS_2054151-3	67	10	0,1	-16	1	-37	GCAACAACAA	-14	CCTATACTCC	-139	4,1	9.70	7.40	s	1187	Rmet_6719	0	Op1583r_2	NA
2766	TSS_116278+5	67	21	0,1	-16	1	-39	TTGTGCCCTG	-14	GCAAAAAATTT	-145	1,1	6.30	5.40	m	1315	Rmet_6263	rhsA	Op1910f_1	NA
2767	TSS_1717894+2	67	3	0,2	-5	0	-45	TGCTGCGCCT	-15	CCTAATGTAG	-145	1,1	1.63	-9.60	no	55	Rmet_1594	0	Op0443f_2	NA
2768	TSS_224501-2	67	8	0,1	-15	1	-45	TCGGCACCAC	-13	GGCATGATGT	-139	5,1	9.44	-7.10	no	4218	Rmet_0208	0	Op0062r_1	NA
2769	TSS_2384390-2	67	18	0,1	-17	1	-39	TTGTCATGAT	-15	GACATAATCT	-143	1,1	9.45	9.40	s	39	Rmet_2177	ppx	Op0626r_1	MET-NUC-Pur
2770	TSS_2666395-2	67	11	0,1	-15	1	-39	TTACAGGTAT	-13	AGTAAACTTG	-145	4,1	7.27	2.90	w	3910	Rmet_2450	0	Op0684r_1	NA
2771	TSS_3561403-2	67	14	0,1	-15	1	-39	TTGACCGGCG	-13	GATATGCTGC	-136	1,1	8.86	6.40	s	506	Rmet_3284	0	Op0924r_1	NA
2772	TSS_2071706+3	67	15	0,4	-17	1	-27	GCGGCCTTCC	-3	GGGATCGTGC	-142	5,1	5.73	-3.30	no	34	Rmet_5351	0	Op1590f_1	NA
2773	TSS_276442-3	67	6	0,1	-15	1	-45	TGGCAGCGTC	-13	GCGAAAATGC	-139	1,1	7.74	-9.10	no	35	Rmet_3732	flgM	Op1041r_2	DIV-MOT-Flagel
2774	TSS_1985532-3	67	8	0,1	-16	1	-42	GGTAAGGCGT	-14	TCTATAATCC	-142	4,1	6.46	-3.60	no	3183	Rmet_5282	0	Op1557r_1	NA
2775	TSS_195843+2	66	10	0,1	-15	1	-37	TTGTGCCGCA	-13	GCCATATTCG	-141	1,1	6.32	8.40	s	322	Rmet_0188	0	Op0057f_1	NA
2776	TSS_2073225+2	66	22	0,1	-2	0	-36	TTGAGTCGCC	-15	GATAGGTTGG	-141	1,1	6.36	10.80	s	9	Rmet_6492	0	Op0555f_2	NA
2777	TSS_29673-2	66	7	0,1	-15	1	-43	GCCGGGGCCT	-13	CGCAACATTC	-141	4,1	5.51	-10.60	no	37	Rmet_0024	0	Op0006r_1	NA
2778	TSS_695024-2	66	25	0,1	-15	1	-35	TGCCGGTACC	-13	GATACTCTTC	-142	1,1	5.29	7.20	s	8544	Rmet_0625	0	Op0178r_1	NA
2779	TSS_1295956-2	66	6	0,1	-27	0	-46	CCGACCTGTC	-25	TCTACATTGC	-144	5,1	6.08	-1.70	no	131	Rmet_1182	0	Op0342r_2	NA
2780	TSS_1833839+3	66	13	0,1	-17	1	-43	TGCAATGAGG	-15	ATTACACTGC	-140	1,1	6.44	-3.60	no	13729	Rmet_5149	bugT	Op1516f_1	NA
2781	TSS_2228864+3	66	7	0,1	-16	1	-36	TGGACCTCCT	-14	GGCAGACTGC	-142	1,1	7.21	10.90	s	1073	Rmet_6732	0	Op2096f_1	NA
2782	TSS_1650627-3	66	10	0,1	-15	1	-43	CCGGCAGCAG	-13	GATATTCTTC	-138	5,1	9.74	-6.10	no	1699	Rmet_4971	0	Op1459r_1	NA
2783	TSS_1686086-3	66	10	0,1	-15	1	-33	TCGTTGCTCG	-13	GATACAGTAC	-139	1,1	4.84	6.10	m	229	Rmet_5005	0	Op1469r_2	NA
2784	TSS_1924965-3	66	9	0,1	-15	1	-49	TTGTCTCGTG	-13	GCTACAGTTT	-142	1,1	7.69	-14.60	no	30	Rmet_5228	0	Op1541r_1	NA
2785	TSS_2034321-3	66	35	0,5	-6	0	-40	TGATTCGTCA	-20	GATATCTCCG	-149	3,0	2.71	0.80	w	72	Rmet_5324	0	Op1575r_1	NA
2786	TSS_221885-4	66	14	0,1	-15	1	-33	TGCAAACGGG	-13	GCGAGAATGC	-140	1,1	6.51	6.60	s	9396	Rmet_6089	0	Op1854r_1	NA
2787	TSS_120583-5	66	12	0,1	-15	1	-35	TTGCCCCATC	-13	TATAGATTTG	-142	1,1	7.66	12.20	s	3303	Rmet_6262	0	Op1909r_1	NA
2788	TSS_3617008-2	66	9	0,1	-15	1	-36	TTGACGGGGA	-13	TGGATGATGT	-142	1,1	7.58	12.40	s	646	Rmet_R0063	0	Op0930r_1	NA
2789	TSS_1677574-3	66	13	0,1	-14	1	-39	GTAACGGCGC	-12	GGTATCATGT	-139	4,1	10.08	1.90	w	641	Rmet_4997	0	Op1467r_1	NA
2790	TSS_509981+2	66	11	0,1	-16	1	-45	TTCAAGGAGA	-14	GCTATCATGT	-143	1,1	8.85	-6.10	no	864	Rmet_6420	0	Op0129f_1	NA
2791	TSS_2152021+2	66	7	0,1	-16	1	-35	TTGCGCAGCA	-14	CGCACGATTT	-137	1,1	6.68	9.60	s	3236	Rmet_1981	lexA	Op0577f_1	GIP-TK-Assoc
2792	TSS_220350-2	66	8	0,1	-16	1	-34	TTCCATTATT	-14	ACTACAATCC	-137	1,1	9.04	8.10	s	67	Rmet_0208	0	Op0062r_1	NA
2793	TSS_1371266-2	66	24	0,1	-15	1	-42	GGCGCAGTGC	-13	TCTATATTTT	-138	4,1	8.05	-4.60	no	20	Rmet_1246	0	Op0358r_1	NA
2794	TSS_2667614-2	66	14	0,1	-8	0	-49	TTGCCGGAGT	-6	CTTAATATTT	-143	1,1	8.42	-21.60	no	30	Rmet_2456	0	Op0686r_1	NA
2795	TSS_3550284-2	66	7	0,1	-15	1	-35	TTCCTCGAGC	-13	GCTACCATGA	-136	1,1	7.47	9.70	s	2309	Rmet_3273	0	Op0920r_1	NA
2796	TSS_3859241-2	66	10	0,1	-15	1	-34	TTGACGAACC	-13	GTTATAGTTT	-140	1,1	9.67	12.60	s	5	Rmet_R0069	0	Op0988r_3	NA
2797	TSS_150642+3	66	19	0,1	-28	0	-44	TGGCGGTCGT	-15	TATAGGGTCA	-146	1,1	4.24	-6.10	no	#NV	#NV	#NV	#NV	#NV

2798	TSS_413804+3	66	12	0,1	-17	1	-35	TTCCTGTTCG	-15	GCTAATTTGC	-141	2,1	9.52	7.10	s	25	Rmet_3870	0	Op1078f_1	NA
2799	TSS_1561915+3	66	13	0,1	-17	1	-46	TCGCCCCGTC	-15	CGCATAATTG	-139	1,1	7.61	-7.10	no	100	Rmet_4898	0	Op1434f_2	NA
2800	TSS_1533138-3	66	8	0,1	-15	1	-41	TTGAGCCTCC	-13	GCTACATTTG	-138	1,1	8.89	1.40	(w)-sba	22	Rmet_4870	0	Op1421r_1	NA
2801	TSS_1561620-3	66	6	0,1	-26	0	-46	TGGCCAGCAG	-24	GGCAAGATGT	-142	1,1	6.75	0.90	w	2660	Rmet_4894	0	Op1431r_1	MET-NUC-Pyr
2802	TSS_111913+5	66	10	0,1	-16	1	-36	TGGTCCGATC	-14	AGTACCATTA	-144	1,1	6.22	10.90	s	3136	Rmet_6261	rhsB	Op1908f_1	NA
2803	TSS_429614+2	65	7	0,1	-17	1	-43	TGGTCAAGCG	-15	TGTATGATCG	-144	1,1	7.92	-0.10	no	1877	Rmet_0404	ttuC	Op0111f_1	MET-CAH-But
2804	TSS_2208413+2	65	12	0,1	-15	1	-49	GTAGAACAGC	-13	CGTAAAATGC	-138	4,1	11.06	-18.10	no	19367	Rmet_2056	0	Op0597f_1	NA
2805	TSS_1894418+3	65	17	0,1	-21	0	-35	TTGACATGTA	-14	AATAAAGTAT	-145	1,1	7.54	13.60	s	83	Rmet_5199	0	Op1534f_1	NA
2806	TSS_352238-3	65	8	0,1	-16	1	-49	TTGCAATGTC	-14	TATAGAATCG	-137	1,1	10.26	-12.60	no	1132	Rmet_3809	0	Op1059r_1	NA
2807	TSS_766668-3	65	3	0,1	-16	1	-41	TTGTGTGAGT	-14	GCTAACATGG	-139	1,1	8.53	1.40	(w)-sba	708	Rmet_4180	0	Op1179r_1	NA
2808	TSS_831643-3	65	16	0,1	-11	1	-33	TGCCCCCGAT	-9	ACTATTATGC	-141	1,1	6.74	6.20	(m)-ba	3004	Rmet_4240	acnA3	Op1201r_1	NA
2809	TSS_1290157-3	65	6	0,1	-15	1	-21	GAATTTGTTG	-1	GTGCATCACG	-141	3,0	8.70	-16.40	no	3348	Rmet_4654	0	Op1345r_1	NA
2810	TSS_2278090-3	65	23	0,1	-15	1	-46	TGGCCTCCCA	-13	AGTATAATGG	-142	1,1	9.09	-8.10	no	55	Rmet_5530	0	Op1643r_2	MET-XEN-Benz
2811	TSS_603872+2	65	12	0,1	-17	1	-46	TTGCGGCAAA	-15	GCTACGATGA	-140	1,1	8.99	-7.60	no	181	Rmet_0564	0	Op0159f_1	NA
2812	TSS_2940690+2	65	30	0,1	-10	1	-29	TTTCTGCGGC	-8	AACAGCATAT	-144	2,1	6.92	2.10	(w)-nu	202	Rmet_2708	0	Op0767f_1	NA
2813	TSS_1579493-2	65	16	0,1	-16	1	-36	TTCGGGTCGG	-14	GATACACTTC	-137	2,1	9.36	9.90	s	838	Rmet_1456	smpB	Op0406r_1	GIP-PTL-Fold
2814	TSS_2183265-2	65	13	0,1	-14	1	-39	TGCCGCAGGA	-12	GGCAGAATTG	-137	1,1	6.50	0.40	w	11522	Rmet_2001	tnmB	Op2023r_1	NA
2815	TSS_3750812-2	65	12	0,4	-4	0	-33	TCTGCCAGGT	-11	GTTAAAGTGC	-140	5,1	10.05	7.20	s	157	Rmet_3470	0	Op0964r_1	MET-CAH-Prop
2816	TSS_1868185+3	65	12	0,1	-17	1	-37	TTCTGCAAAG	-15	GGTATGATGC	-138	1,1	8.27	9.90	s	25	Rmet_5170	0	Op1520f_3	NA
2817	TSS_435861+2	65	7	0,1	-16	1	-34	TGGCGGGAAG	-14	GGTAACCTCC	-139	1,1	6.75	6.10	m	748	Rmet_6414	0	Op2001f_1	NA
2818	TSS_2344118+2	65	23	0,4	-6	0	-21	AAATGTGTCG	-1	TAAATGTCAT	-144	3,0	7.85	-13.40	no	28	Rmet_2150	fliC1	Op0621f_1	EIP-SIG-2Comp
2819	TSS_3769068+2	65	9	0,2	-8	0	-43	TCGATCGTGC	-17	ACGAATGTCC	-145	1,1	1.73	-2.10	no	23103	Rmet_3502	0	Op0971f_1	NA
2820	TSS_2968206-2	65	8	0,1	-3	0	-35	TTGTCGGAAT	-1	CTGATTATTA	-147	1,1	5.52	0.20	no	362	Rmet_2731	0	Op0770r_1	NA
2821	TSS_296680+3	65	12	0,1	-16	1	-34	CCTGCTGGCG	-14	CGTATGCTGG	-141	5,1	8.24	3.60	m	7	Rmet_3753	0	Op1044f_2	NA
2822	TSS_474090+3	65	13	0,1	-16	1	-37	TTGACGGGGA	-14	TGGATGATGT	-142	1,1	7.58	11.40	s	645	Rmet_R0076	0	Op1098f_1	NA
2823	TSS_2481212+3	65	11	0,1	-16	1	-34	TCTGCAATTC	-14	ACTAGCATCA	-144	5,1	7.51	7.60	s	66	Rmet_5724	bvgS2	Op1714f_2	NA
2824	TSS_1487613-3	65	32	0,1	-16	1	-46	TCGGCAGCGT	-14	GTCAGAATGT	-142	5,1	9.82	-8.10	no	4770	Rmet_4828	0	Op1407r_1	NA
2825	TSS_140148+4	65	8	0,1	-17	1	-43	GTACACCCGC	-15	GGTAACGTTC	-141	4,1	7.04	-7.10	no	1803	Rmet_6378	0	Op2114f_1	NA
2826	TSS_3916613+2	64	10	0,1	-16	1	-48	TTGCCAAAGC	-14	TCTACCATCG	-139	1,1	9.62	-10.60	no	1018	Rmet_3605	0	Op1003f_1	NA
2827	TSS_2212369-2	64	15	0,1	-15	1	-47	TTGATGAAAG	-13	TCTAAAGTCA	-144	1,1	7.41	-10.60	no	40	Rmet_2044	ccoN	Op0596r_2	MET-EN-OxPhos
2828	TSS_3615906-2	64	5	0,1	-27	0	-22	ACACTCGTTA	-2	CGAACAAGTG	-147	3,0	5.73	-11.40	no	#NV	#NV	#NV	#NV	#NV
2829	TSS_3713032-2	64	10	0,1	-15	1	-40	TTGCCTGTCC	-13	TATATAGTGC	-138	1,1	8.55	4.40	(m)-sba	41	Rmet_3436	yfgD	Op0954r_1	NA
2830	TSS_30578+3	64	15	0,1	-16	1	-35	TGGAGCGGCG	-14	CGTATCCTGG	-138	1,1	6.58	10.10	s	57	Rmet_5841	0	Op1764f_3	NA
2831	TSS_1168585-3	64	38	0,1	-16	1	-42	TGGCCATGAG	-14	CGTATATTAC	-138	1,1	7.81	0.90	(w)-sba	30	Rmet_4557	msrA	Op1307r_1	GIP-PTL-Fold
2832	TSS_64509-5	64	22	0,1	-16	1	-35	TTGCGTCTCG	-14	GCTAATCTCG	-141	2,1	10.60	9.60	s	26	Rmet_6212	tnpA	Op1897r_1	NA
2833	TSS_109367+2	64	9	0,1	-17	1	-35	TTGACGTTTA	-15	AATAAAATAG	-139	1,1	9.52	12.60	s	50	Rmet_0103	ivd1	Op0031f_2	MET-AA-ValLeuIle
2834	TSS_2664637+2	64	22	0,1	-16	1	-36	TTGACGAAAC	-14	GTTATAATTT	-141	1,1	12.04	15.40	s	8	Rmet_R0041	0	Op0685f_2	NA
2835	TSS_3616389-2	64	8	0,1	-9	0	-27	TTGTGTTCAC	-7	TTCACACTTA	-144	1,1	5.85	0.60	(w)-nu	27	Rmet_R0063	0	Op0930r_1	NA
2836	TSS_1205686+3	64	20	0,1	-17	1	-46	GGCCGAAACC	-15	GCTACCATTG	-139	4,1	9.36	-12.60	no	180	Rmet_4587	0	Op1320f_1	NA
2837	TSS_819902+2	64	25	0,1	-16	1	-36	TGGATGTGTG	-14	TCTATACTAT	-142	1,1	7.63	11.90	s	48	Rmet_0741	lrp	Op0209f_1	NA
2838	TSS_2504578-2	64	4	0,1	-15	1	-38	TTCTCCACGG	-13	GGTATTGTGG	-142	1,1	5.84	5.90	m	148	Rmet_2282	0	Op0652r_2	NA
2839	TSS_3078600-2	64	8	0,1	-24	0	-47	TGCAAACTTG	-22	AACACAATGG	-143	1,1	5.85	-3.60	no	900	Rmet_2828	0	Op0796r_1	NA
2840	TSS_3124646-2	64	10	0,1	-15	1	-36	GCCAAGACGC	-13	TGTACGATTC	-136	4,1	10.39	5.40	m	823	Rmet_2873	nadC	Op0808r_2	MET-COF-NicAm
2841	TSS_325079+3	64	11	0,1	-16	1	-36	TTGACATGGT	-14	CTTACATTGC	-140	1,1	9.50	14.40	s	27	Rmet_3788	fadD	Op1050f_3	MET-LIP-FASyn
2842	TSS_556621+3	64	12	0,1	-17	1	-36	TGGCGCTGGT	-15	GACACAGTTG	-141	1,1	5.96	8.30	s	897	Rmet_3988	0	Op1118f_1	NA
2843	TSS_1625699-3	64	9	0,1	-16	1	-43	TCGCCGTACC	-14	GATACCATTC	-139	1,1	7.98	-3.10	no	4662	Rmet_4954	0	Op1455r_1	NA
2844	TSS_3476858+2	63	21	0,1	-17	1	-41	TGGCGACCGC	-15	CCTACAATTT	-136	1,1	8.91	2.90	(w)-sba	304	Rmet_3202	0	Op0901f_2	NA
2845	TSS_2368006-2	63	27	0,1	-15	1	-35	TTCACCGTAT	-14	TGCAATATTG	-141	1,1	6.02	10.10	s	4933	Rmet_2162	0	Op0622r_1	NA
2846	TSS_2399007-2	63	4	0,1	-4	0	-42	TGGCATGAGA	-12	CGTATTATCC	-140	1,1	7.58	-3.10	no	119	Rmet_2189	rrmJ	Op0628r_1	DIV-Division
2847	TSS_3081997-2	63	17	0,1	-15	1	-41	TCCCGCATGT	-13	TGTACCATCG	-142	1,1	6.24	-2.60	no	4297	Rmet_2828	0	Op0796r_1	NA
2848	TSS_3612503-2	63	11	0,1	-27	0	-46	TTGACAGCCA	-25	ACCATAATCA	-139	1,1	8.50	4.80	m	132	Rmet_R0058	0	Op0930r_3	NA
2849	TSS_390679+3	63	11	0,1	-26	0	-47	TGGCATCAAT	-24	CTCATACTGT	-141	1,1	6.36	-2.10	no	57	Rmet_3848	0	Op1070f_1	MET-COF-CoQ
2850	TSS_971523+3	63	5	0,1	-16	1	-34	TTGCCTTGTG	-14	ACGATACTGA	-143	1,1	6.93	9.60	s	211	Rmet_4373	0	Op1248f_1	NA
2851	TSS_1146217-3	63	17	0,1	-14	1	-45	TTGAGGTCAA	-12	GCTACGAGTGG	-139	1,1	9.66	-7.60	no	27	Rmet_4538	soxR	Op1299r_1	NA
2852	TSS_121153+5	63	14	0,1	-16	1	-42	TTCTCAATGC	-14	TAGATAATGC	-142	1,1	7.36	0.90	(w)-sba	959	Rmet_6265	mtp2	Op1910f_1	NA
2853	TSS_269079+2	63	14	0,1	-17	1	-49	TCGAGCAAGG	-15	GGTACAGTGA	-140	1,1	7.40	-14.10	no	136	Rmet_0252	grxC	Op0073f_1	GIP-PTL-Fold
2854	TSS_3511868+2	63	26	0,1	-16	1	-36	ATCCGGCGGC	-14	GCGATAATAA	-142	2,1	7.70	7.90	s	19501	Rmet_3261	0	Op0919f_1	NA
2855	TSS_1832050-2	63	11	0,1	-15	1	-41	TTCCCCTTGG	-13	CGCACAATCC	-139	1,1	7.62	-0.10	no	821	Rmet_6468	0	Op0478r_1	NA
2856	TSS_3125038-2	63	10	0,1	-16	1	-43	GCCCCGCCCC	-14	GCTAAACTTC	-141	4,1	8.13	-7.60	no	64	Rmet_2874	nadA	Op0808r_2	MET-COF-NicAm
2857	TSS_3431156-2	63	1	0,1	-9	0	-27	TTGTGTTCAC	-7	TTCACACTTA	-144	1,1	5.85	0.60	(w)-nu	27	Rmet_R0053	0	Op0886r_1	NA
2858	TSS_2352+3	63	8	0,1	-9	0	-34	TTGAACCACT	-7	ATAAGAATTT	-144	1,1	6.43	6.20	(m)-ba	415	Rmet_5819	parA	Op1758f_1	DIV-Division

2859	TSS_2410179-3	63	8	0,1	-16	1	-41	GGAGCGCCGC	-14	GCTAGACTAC	-140	4,1	8.13	-3.60	no	1086	Rmet_5647	uspA15	Op1685r_1	NA
2860	TSS_1533520+2	63	22	0,1	-16	1	-43	GCAGAACTAC	-14	ACTAAGCTTC	-144	4,1	8.10	-8.60	no	49	Rmet_1418	0	Op0397f_3	NA
2861	TSS_3593130+2	63	12	0,1	-16	1	-43	TTGATGCCGT	-14	GGTATCCTCG	-137	1,1	8.45	-1.60	no	983	Rmet_3329	0	Op0929f_1	NA
2862	TSS_3630835+2	63	15	0,2	-14	1	-35	TTGTGGCAAA	-14	CGAATAGTGT	-146	1,1	4.39	10.60	s	717	Rmet_3355	0	Op0935f_2	NA
2863	TSS_314719-2	63	6	0,1	-16	1	-37	TTGCCAACTA	-14	GGTAATGTGC	-139	1,1	7.72	10.40	s	76	Rmet_0296	0	Op0086r_3	NA
2864	TSS_584053-2	63	13	0,1	-15	1	-36	TCGTCCACGA	-13	GCTAAAATTC	-138	1,1	8.92	10.90	s	322	Rmet_0547	0	Op0152r_1	NA
2865	TSS_2507614+3	63	16	0,1	-16	1	-48	TTACAAACAC	-14	GGTAGAGTGG	-142	4,1	9.13	-13.10	no	35	Rmet_5747	0	Op1718f_2	NA
2866	TSS_143155-4	63	15	0,1	-15	1	-42	TTCACATCGA	-13	GGTAAGCTGC	-141	1,1	8.10	-1.10	no	40	Rmet_6155	0	Op1882r_1	NA
2867	TSS_125951-5	63	1	0,1	-15	1	-44	TCTGCCCTAT	-13	AGTAGCATAC	-145	5,1	8.07	-7.60	no	27	Rmet_6398	0	Op1911r_1	NA
2868	TSS_583828-2	62	12	0,1	-9	0	-38	TTCCATCAAA	-7	GATAACTTAT	-147	1,1	4.54	-2.10	no	97	Rmet_0547	0	Op0152r_1	NA
2869	TSS_1670299-2	62	7	0,2	-13	1	-33	TCGAACACGC	-9	TCGAAGAAGA	-144	1,1	-0.31	3.70	(m)-ba	1711	Rmet_1545	0	Op0430r_1	NA
2870	TSS_2464811-2	62	6	0,1	-16	1	-42	GCAGCCCGCG	-14	TTCACAATCT	-142	5,1	9.06	-5.60	no	15	Rmet_2249	dat	Op0644r_1	MET-AA-Lys_Deg
2871	TSS_149540+3	62	14	0,1	-16	1	-37	TTGACGGGGA	-14	TGGATGATGT	-142	1,1	7.58	11.40	s	645	Rmet_R0071	tRNA-l	Op1006f_1	NA
2872	TSS_556870+3	62	18	0,1	-16	1	-44	TTCACGAGCC	-14	GCTATCCTGC	-139	1,1	8.18	-4.10	no	648	Rmet_3988	0	Op1118f_1	NA
2873	TSS_1111897+3	62	10	0,1	-16	1	-36	TGGACAAGCG	-14	GTTATATTGC	-142	1,1	9.11	13.90	s	145	Rmet_4509	0	Op1288f_1	NA
2874	TSS_2350308+3	62	22	0,5	-15	1	-38	TTGCGTCATC	-10	GTTACACCGG	-146	1,1	1.25	1.40	w	52	Rmet_5596	0	Op1666f_2	NA
2875	TSS_258697-3	62	7	0,1	-14	1	-43	TGAAGCCCAG	-12	GGGATCATTC	-148	4,1	5.44	-6.60	no	26	Rmet_3718	0	Op1033r_1	NA
2876	TSS_3188177+2	62	9	0,1	-17	1	-44	TCCTGACAGT	-15	CGCATGATCC	-141	1,1	4.14	-5.60	no	426	Rmet_2933	0	Op0827f_1	NA
2877	TSS_3788905+2	62	17	0,1	-16	1	-34	TTTCTGAGGG	-14	ATCACAATGC	-142	2,1	9.99	7.10	s	3266	Rmet_3502	0	Op0971f_1	NA
2878	TSS_3685362-2	62	4	0,1	-16	1	-41	GCATCAGGCC	-14	CGTATTATTG	-143	5,1	8.22	-1.60	no	142	Rmet_3406	fieF	Op0946r_1	EIP-TRA-Ion
2879	TSS_508381+3	62	11	0,1	-17	1	-37	TGGACATGAA	-15	GATACATTCA	-138	1,1	7.81	11.90	s	250	Rmet_3951	0	Op1108f_1	NA
2880	TSS_2543238-3	62	27	0,1	-17	1	-43	TTCATTACAA	-18	CGCATTATGC	-140	1,1	5.41	0.90	(w)-sba	28	Rmet_5784	0	Op1739r_1	NA
2881	TSS_1600948-2	62	5	0,1	-25	0	-49	GCCCCAACAC	-23	CGGAAAATGA	-140	4,1	8.38	-9.60	no	38	Rmet_1479	0	Op0416r_1	NA
2882	TSS_1863244-2	62	9	0,1	-15	1	-38	TGGACAACCA	-13	TGCATAATGT	-141	1,1	8.55	8.90	s	5614	Rmet_1715	0	Op0484r_1	NA
2883	TSS_2368051-3	62	16	0,1	-15	1	-44	TTCCGCCGCC	-13	GGCATCATGT	-140	2,1	10.15	-7.10	no	4277	Rmet_5607	0	Op1673r_1	NA
2884	TSS_2092157+2	61	14	0,1	-15	1	-33	TTCATGTGCC	-13	GTTAATATCG	-141	1,1	7.38	7.10	s	5902	Rmet_1930	0	Op0563f_1	NA
2885	TSS_2698756+2	61	20	0,1	-16	1	-44	TCGCGAACAG	-14	TATAGACTTC	-140	1,1	7.10	-5.10	no	2984	Rmet_2489	mdh	Op0689f_1	MET-CAH-TCA
2886	TSS_2060742-2	61	3	0,1	-15	1	-35	TGGAGCCAAG	-15	GTGATATTGT	-140	1,1	5.63	9.10	s	51	Rmet_1892	0	Op0548r_1	NA
2887	TSS_3105681-2	61	5	0,1	-15	1	-46	CCAGCAACAG	-24	CTTATTTTTC	-143	5,1	7.46	-2.60	no	47	Rmet_2855	hutH	Op0804r_1	MET-EN-Nitrog
2888	TSS_2303658-3	61	18	0,2	-15	1	-43	TTGCCGCGTC	-13	CATAGAATGG	-138	1,1	9.61	-1.60	no	8834	Rmet_6743	0	Op1647r_1	NA
2889	TSS_13144-4	61	11	0,1	-15	1	-47	TGGATAAGCT	-13	GGTAATCTAC	-143	1,1	6.72	-12.10	no	8321	Rmet_6060	repA	Op1846r_1	NA
2890	TSS_133326-4	61	16	0,1	-15	1	-35	TCGTCCAGAA	-13	CGTACAGTGC	-141	1,1	6.29	10.70	s	2107	Rmet_6170	0	Op1888r_1	NA
2891	TSS_276527+2	61	14	0,1	-19	0	-48	CCGGCGACGC	-17	CGTATACTCC	-134	5,1	11.46	-11.10	no	124	Rmet_0259	0	Op0075f_1	NA
2892	TSS_302029+2	61	19	0,1	-16	1	-35	TTGTTCAACG	-14	GCAAGAATCA	-142	1,1	5.62	10.60	s	52	Rmet_0285	coaD	Op0083f_3	MET-COF-Pant
2893	TSS_396510+2	61	20	0,1	-16	1	-45	TGCCCCTAGC	-14	ATCAGAATGC	-141	1,1	5.93	-8.60	no	170	Rmet_0372	0	Op0103f_4	NA
2894	TSS_1250627+2	61	19	0,1	-16	1	-34	TCGGCTAGCT	-14	GTTACACTGC	-138	5,1	10.31	8.10	s	5	Rmet_6439	0	Op0327f_1	NA
2895	TSS_2965160+2	61	18	0,1	-17	1	-46	GGCGGAGAAT	-15	GGCAGAATCG	-140	4,1	8.66	-12.60	no	6517	Rmet_2736	apaH	Op0771f_1	MET-NUC-Pur
2896	TSS_1226091+3	61	6	0,1	-16	1	-41	TTGTTTATCC	-21	GCCATGCTGG	-144	1,1	5.85	3.80	m	4831	Rmet_4606	metY	Op1326f_1	MET-AA-Met
2897	TSS_1389294+3	61	21	0,1	-16	1	-35	TGGACTGAGT	-14	CCCATAATGT	-140	1,1	8.06	12.10	s	54	Rmet_4745	0	Op1376f_2	NA
2898	TSS_1257084-3	61	11	0,1	-14	1	-39	TCCATCAAAT	-12	GGTAAAATCG	-138	1,1	8.22	2.40	w	101	Rmet_4627	0	Op1333r_1	EIP-SIG-2Comp
2899	TSS_7465+2	61	10	0,1	-16	1	-40	TGGATCAAGG	-14	TTTAAACTCT	-144	1,1	7.15	2.90	(w)-sba	9930	Rmet_0013	mazG	Op0003f_1	NA
2900	TSS_68633+2	61	13	0,1	-15	1	-43	TCGAGCAGGC	-13	CGTATAATCT	-137	1,1	9.39	-2.10	no	451	Rmet_0061	lipA	Op0019f_2	NA
2901	TSS_2043344+2	61	4	0,1	-17	1	-46	TTCACCCGCT	-15	TGCAAAATCG	-140	1,1	7.56	-7.10	no	47	Rmet_1881	0	Op0543f_1	NA
2902	TSS_50747+5	61	16	0,1	-16	1	-47	TTGCGATGAT	-14	CATAGAATAC	-140	1,1	9.59	-8.60	no	1553	Rmet_6204	chrI	Op1896f_1	NA
2903	TSS_960946+2	60	7	0,1	-17	1	-37	TTCCCGCCTT	-15	CAGAGAATGA	-140	1,1	5.43	9.90	s	8582	Rmet_0885	0	Op0257f_1	NA
2904	TSS_1374669+3	60	1	0,1	-16	1	-37	TGCCGCCGGG	-14	GATACCATAC	-140	1,1	5.78	6.40	s	26	Rmet_4731	0	Op1368f_1	NA
2905	TSS_1198388-3	60	6	0,1	-16	1	-45	GCGGAACCGC	-14	CATATAATCC	-139	5,1	10.89	-8.10	no	10496	Rmet_4573	0	Op1315r_1	NA
2906	TSS_30816-5	60	5	0,1	-20	0	-42	GGAAAACCAT	-18	CGTATGGTAG	-143	4,1	7.28	-0.60	no	191	Rmet_6342	stbD	Op1927r_1	NA
2907	TSS_3078625+2	60	13	0,1	-17	1	-41	TCAACGATGC	-15	GCTACCATTG	-139	4,1	8.79	1.40	(w)-sba	1698	Rmet_2831	0	Op0797f_2	NA
2908	TSS_2137691-2	60	13	0,1	-15	1	-43	GCCAGGCCGT	-13	TATAACATTG	-141	4,1	7.65	-8.60	no	171	Rmet_1960	phaY	Op0574r_1	MET-CAH-But
2909	TSS_319809+3	60	6	0,1	-17	1	-47	TCGCGATCAA	-15	ATTACTATCG	-141	1,1	6.52	-10.10	no	663	Rmet_3784	bugT	Op1050f_2	NA
2910	TSS_377885+3	60	32	0,1	-22	0	-41	TTCCCGGTGC	-20	GACATGATCG	-143	1,1	6.81	5.30	m	71	Rmet_3836	hmvC	Op1066f_2	EIP-TRA-Ion
2911	TSS_574671+3	60	15	0,1	-5	0	-36	TTGAATTCTA	-3	GCCATATTGG	-142	1,1	7.80	1.40	(w)-ba	122	Rmet_4003	0	Op1124f_1	MET-AA-AlaAsp
2912	TSS_1302186+3	60	11	0,1	-16	1	-49	GCAGGAACGC	-27	GACAAACTGG	-140	4,1	8.61	-7.60	no	3768	Rmet_4673	0	Op1352f_1	NA
2913	TSS_1503178+3	60	10	0,1	-16	1	-45	GGAGACGACG	-14	CCTATAATGC	-136	4,1	9.51	-10.60	no	67	Rmet_4846	0	Op1412f_2	NA
2914	TSS_410991-3	60	3	0,1	-17	1	-36	TCGACAGTCA	-15	CCTATACTCG	-138	1,1	8.29	13.30	s	0	Rmet_3867	0	Op1075r_1	NA
2915	TSS_618530-3	60	15	0,1	-15	1	-35	TTGCGGGCTT	-13	GGTACTCTCT	-135	1,1	7.68	10.20	s	272	Rmet_4051	0	Op1141r_3	MET-OAA-GSH
2916	TSS_1462699-3	60	19	0,1	-16	1	-46	TGCCGAACGT	-14	GCTACCATTT	-138	1,1	7.25	-10.60	no	853	Rmet_4813	0	Op1401r_1	NA
2917	TSS_905412+2	60	7	0,1	-17	1	-23	TTGAGCGATT	-3	GCCATACTTC	-139	1,1	8.90	-1.40	no	551	Rmet_0831	alaS	Op0237f_1	MET-AA-AlaAsp
2918	TSS_1600660+2	60	12	0,1	-15	1	-33	TTCAGATAGG	-13	TCGATCATCC	-142	1,1	6.22	8.10	s	1115	Rmet_1480	0	Op0417f_1	NA
2919	TSS_1539301-2	60	8	0,1	-16	1	-37	TTCCCGCTGC	-14	GGCATCATCT	-139	1,1	6.96	8.90	s	177	Rmet_1424	dapC	Op0398r_1	NA

2920	TSS_2618081-2	60	10	0,1	-16	1	-39	TCCACCGACA	-14	GCCAAAATTG	-143	1,1	6.38	4.40	m	94	Rmet_2408	*int*	Op0678r_2	NA
2921	TSS_2637841-2	60	10	0,1	-16	1	-40	TTGTCGAACT	-14	TGGACAATAT	-143	1,1	7.22	4.40	*(m)-sba*	435	Rmet_2426	*fabF*	Op0680r_3	MET-LIP-FASyn
2922	TSS_3624355-2	60	5	0,1	-27	0	-47	TGGCCTCTGT	-25	GACACATTAA	-146	1,1	4.84	-1.10	*no*	694	Rmet_3346	*uspA8*	Op0932r_3	NA
2923	TSS_1675158+3	60	7	0,1	-29	0	-43	TTGCGTTGCG	-14	GGCACAATAC	-138	2,1	13.15	-2.60	*no*	9520	Rmet_5004	*0*	Op1468f_1	NA
2924	TSS_1729716+3	60	10	0,1	-16	1	-38	TTCCCCAGTT	-14	ATTACACTTA	-137	1,1	7.64	6.90	s	12108	Rmet_5052	*0*	Op1486f_1	NA
2925	TSS_1789727+3	60	10	0,1	-16	1	-46	GTAAACCCCT	-14	TATAAAATCT	-143	4,1	8.59	-11.10	*no*	65	Rmet_5098	*0*	Op1500f_1	NA
2926	TSS_107361+4	60	11	0,1	-16	1	-28	TTCCGCTCGT	-6	CGCATCATCT	-143	1,1	5.91	2.70	*(w)-nu*	2106	Rmet_5950	*int*	Op1809f_1	NA
2927	TSS_92535-4	60	3	0,1	-22	0	-36	TTGACCCTGT	-13	TATTTACTAC	-148	1,1	3.27	11.40	s	81	Rmet_5969	*0*	Op1816r_3	NA
2928	TSS_1757673+2	59	7	0,1	-17	1	-36	GTCAGAACGT	-15	GTGACAATAT	-142	4,1	8.41	8.30	s	926	Rmet_1630	*0*	Op0457f_1	NA
2929	TSS_3844603+2	59	10	0,1	-18	1	-48	TCCCCTGTGT	-16	GGTAGAATCG	-139	1,1	8.00	-11.60	*no*	1340	Rmet_3547	*0*	Op0983f_3	NA
2930	TSS_568184-2	59	44	0,1	-15	1	-46	CCTGACCGCG	-13	GGTACCCTCC	-139	5,1	8.93	-16.60	*no*	44	Rmet_0532	*adk*	Op0148r_1	MET-NUC-Pur
2931	TSS_1668665-2	59	8	0,1	-16	1	-34	TCGGCTACGG	-14	CCTAAAATGC	-142	5,1	9.75	9.10	s	77	Rmet_1545	*0*	Op0430r_1	NA
2932	TSS_1083287+3	59	20	0,1	-5	0	-23	TTGAATTATA	-3	TACATAATAT	-143	1,1	8.25	-0.40	*no*	20	Rmet_4483	*0*	Op1278f_1	NA
2933	TSS_2067870-3	59	16	0,1	-15	1	-35	TGCCAATCGG	-13	GCCATCATGG	-139	1,1	6.20	9.20	s	23	Rmet_5348	*0*	Op1589r_1	NA
2934	TSS_2507575-3	59	2	0,1	-15	1	-44	TTGCTACGCC	-13	ATTACCATTC	-142	1,1	8.11	-4.60	*no*	1724	Rmet_5743	*0*	Op1717r_1	MET-CAH-FbP
2935	TSS_98877+5	59	6	0,1	-16	1	-47	TGGAGCTAGT	-14	GCTACATTGC	-139	1,1	8.59	-11.10	*no*	990	Rmet_6253	*0*	Op1904f_4	NA
2936	TSS_2043377+2	59	8	0,1	-20	0	-45	AAAATCGTCA	-25	CGTATCGGCT	-142	3,0	8.85	-6.20	*no*	14	Rmet_1881	*0*	Op0543f_1	NA
2937	TSS_775461-2	59	11	0,1	-15	1	-35	TTGCCGTCCA	-13	GCGACAGTGT	-139	1,1	6.59	11.20	s	56	Rmet_0702	*0*	Op0198r_1	NA
2938	TSS_3120688-2	59	8	0,1	-15	1	-42	TGCCATGAAG	-13	TGGACAATGT	-145	1,1	5.47	-4.60	*no*	318	Rmet_2869	*fkpB*	Op0806r_1	GIP-PTL-Fold
2939	TSS_1286792+3	59	8	0,1	-16	1	-36	TGCCACAACT	-14	CCTAGAATCC	-140	1,1	7.48	9.40	s	34	Rmet_4655	*argH*	Op1346f_1	MET-AA-AlaAsp
2940	TSS_1478111+3	59	5	0,1	-16	1	-41	TTCAGCATCA	-14	GATATCCTGA	-141	1,1	6.78	0.90	*(w)-sba*	4970	Rmet_4829	*qseB*	Op1408f_1	EIP-SIG-2Comp
2941	TSS_274095-3	59	12	0,1	-16	1	-38	TTGCACCTCC	-14	GGCAAAATTC	-141	1,1	9.43	7.40	s	61	Rmet_3729	*icdA*	Op1041r_3	MET-CAH-TCA
2942	TSS_462916+2	59	10	0,1	-16	1	-36	TACCGTCGCC	-14	CAGACAATAC	-141	2,1	8.73	8.40	s	338	Rmet_0437	*surA*	Op0117f_1	MET-COF-B6
2943	TSS_2624606-2	59	12	0,1	0	0	-49	TGGCGGAATG	-23	CTGACCGTTC	-142	1,1	2.58	-9.10	*no*	101	Rmet_2413	*nagZ*	Op0680r_4	MET-CAH-AminSug
2944	TSS_2896469-2	59	8	0,1	-16	1	-48	TCGCCAACAA	-14	GTTAGGATAT	-140	1,1	7.10	-12.10	*no*	513	Rmet_2661	*0*	Op0754r_3	NA
2945	TSS_180173+3	59	13	0,1	-16	1	-49	GGCCAGACGG	-14	CCTATCATGA	-142	4,1	7.98	-19.60	*no*	81	Rmet_3647	*0*	Op1018f_1	NA
2946	TSS_1167979+3	59	9	0,1	-23	0	-41	CTGACCGCGT	-21	ACTATACTGT	-144	1,1	5.13	4.80	*m*	642	Rmet_4558	*0*	Op1308f_1	NA
2947	TSS_479225-3	59	8	0,2	-20	0	-34	TTGTCCTTGC	-13	AGTACTGTTA	-141	1,1	5.53	9.60	s	6144	Rmet_6633	*0*	Op1097r_1	NA
2948	TSS_2524963-3	59	34	0,1	-15	1	-39	TGCCGCTTTG	-13	GCTACGCTGT	-145	1,1	5.74	0.40	w	2726	Rmet_5760	*0*	Op1727r_1	NA
2949	TSS_1496806+2	58	10	0,1	-16	1	-47	TTGAATAACG	-14	ATTAGAATCG	-142	1,1	9.32	-8.60	*no*	1767	Rmet_1389	*dehII*	Op0387f_1	MET-XEN-Hex
2950	TSS_2405837+2	58	29	0,5	-25	0	-44	TTCTACATCC	-23	GCCGTAATTG	-146	1,1	1.32	0.30	*w*	14194	Rmet_2207	*0*	Op0631f_1	NA
2951	TSS_3473252+2	58	14	0,1	-16	1	-35	TTTCGCCCCC	-14	GGTATGCTGG	-139	2,1	10.83	9.10	s	1210	Rmet_3200	*hemA*	Op0901f_1	MET-COF-Porph
2952	TSS_2230968+3	58	21	0,1	-17	1	-39	TCGAAGGGTT	-15	GGTAAAATCT	-139	1,1	9.69	6.90	s	2035	Rmet_6733	*0*	Op2097f_1	NA
2953	TSS_1087393-3	58	11	0,1	-16	1	-35	TTGTGTGTGT	-14	AGTAATGTCC	-147	1,1	5.08	9.60	s	57	Rmet_4487	*0*	Op1279r_2	NA
2954	TSS_302426+2	58	7	0,1	-10	1	-43	TGGCGGGCAT	-8	GAAACGATGT	-141	1,1	4.66	-11.10	*no*	207	Rmet_0286	*fdx*	Op0083f_3	NA
2955	TSS_1095198+2	58	12	0,1	-16	1	-44	TTGAAAAATC	-14	GCTAGACTTG	-139	1,1	10.34	-2.60	*no*	68	Rmet_1004	*grpE*	Op0287f_1	GIP-PTL-Fold
2956	TSS_2523190+2	58	21	0,1	-16	1	-37	TTGACTCTAT	-11	CTAATGATGG	-146	1,1	6.29	8.40	s	2949	Rmet_2304	*pbrC2*	Op0659f_1	GIP-PTL-Exp
2957	TSS_2912433-2	58	5	0,1	-15	1	-35	TCGAACGCGT	-13	GGTCCAATTT	-141	1,1	4.80	9.70	s	124	Rmet_2678	*tolQ*	Op0756r_2	NA
2958	TSS_248884+3	58	9	0,1	-14	1	-35	TTGCCATGTC	-12	TCTAATCTGC	-140	1,1	8.40	11.20	s	37	Rmet_3707	*0*	Op1028f_1	NA
2959	TSS_611069+3	58	6	0,1	-16	1	-44	CCGGCAGCAG	-14	GATATTCTTC	-138	5,1	9.74	-7.10	*no*	1796	Rmet_4046	*ggt*	Op1140f_1	MET-LIP-Others
2960	TSS_2431286+3	58	11	0,1	-16	1	-48	TTCCGGTAAT	-14	CGTACACTTG	-141	1,1	8.20	-14.10	*no*	46	Rmet_5672	*copR2*	Op1696f_1	EIP-SIG-2Comp
2961	TSS_1931525-3	58	7	0,1	-16	1	-39	TCGTAACGCA	-14	CCCAGAATAC	-140	1,1	5.16	4.90	m	3428	Rmet_5230	*iscS2*	Op1543r_1	NA
2962	TSS_67754+4	58	7	0,1	-16	1	-38	TTCCCCCAGC	-14	TTCACAATGT	-139	1,1	7.46	6.90	s	4674	Rmet_6365	*0*	Op2109f_1	NA
2963	TSS_1130192+2	58	9	0,1	-16	1	-47	TGGCCGGCGC	-14	GATAAACTGC	-139	1,1	8.61	-11.10	*no*	29	Rmet_1040	*lolE*	Op0299f_1	NA
2964	TSS_858471-2	58	21	0,1	-15	1	-40	GCAGCAACGG	-13	GATACAATGC	-134	5,1	11.64	-0.60	*no*	188	Rmet_0781	*0*	Op0222r_2	NA
2965	TSS_659730-3	58	14	0,1	-16	1	-36	TTGACGACTG	-14	CTTAAAGTCA	-143	1,1	7.35	13.40	s	27	Rmet_4090	*0*	Op1149r_1	NA
2966	TSS_1365344-3	58	6	0,1	-15	1	-47	GTAAAAAAAA	-13	GGCATCGTGA	-142	4,1	6.18	-15.10	*no*	188	Rmet_4722	*0*	Op1363r_2	NA
2967	TSS_1885046-3	58	11	0,1	-15	1	-34	TTCAAATATG	-13	GAGAAACTGT	-147	1,1	6.54	9.10	s	19	Rmet_5190	*0*	Op1531r_1	NA
2968	TSS_169774+5	58	9	0,1	-16	1	-41	TTCACTCACT	-14	GCAAGAATGG	-143	1,1	6.43	1.90	*(w)-sba*	#NV	#NV	#NV	#NV	#NV
2969	TSS_224379+2	57	8	0,1	-16	1	-35	TTCACGGCGG	-14	GCTACACTCA	-139	1,1	8.17	11.10	s	23	Rmet_0211	*0*	Op0063f_3	EIP-TRA-ABC
2970	TSS_2671095+2	57	9	0,1	-15	1	-39	ATCATGCGCG	-13	GCGACCATAG	-143	2,1	5.43	-0.10	no	30645	Rmet_2489	*mdh*	Op0689f_1	MET-CAH-TCA
2971	TSS_2696392+2	57	3	0,1	-16	1	-48	TCGGACGGCG	-14	TGAATAATAG	-144	5,1	8.25	-13.10	*no*	5348	Rmet_2489	*mdh*	Op0689f_1	MET-CAH-TCA
2972	TSS_2642308-2	57	20	0,1	-15	1	-28	AAAATTCTCG	-8	CATGGCTGTT	-143	3,0	8.25	-7.40	*no*	364	Rmet_2431	*plsX*	Op0680r_2	MET-LIP-FASyn
2973	TSS_452284+3	57	12	0,1	-16	1	-35	TTCCATTCGC	-14	GGTACCGTGG	-140	1,1	6.25	8.10	s	367	Rmet_3906	*0*	Op1092f_1	NA
2974	TSS_1310845+3	57	13	0,1	-13	1	-38	TGGCGAACGT	-11	TCGAAGGTCT	-143	1,1	3.27	1.90	w	1502	Rmet_4681	*0*	Op1354f_1	NA
2975	TSS_1919000+3	57	4	0,1	-17	1	-47	TTGCGCGTAT	-15	GTTAAACTCC	-139	1,1	9.73	-9.60	*no*	6034	Rmet_5229	*0*	Op1542f_1	NA
2976	TSS_1965889+3	57	20	0,1	-16	1	-39	GCCCAAGCAC	-14	GCGACGATTC	-138	4,1	8.51	-0.60	no	26	Rmet_5267	*0*	Op1552f_2	NA
2977	TSS_586671+2	57	13	0,1	-17	1	-49	TGGCAGTCGA	-28	GCCACACTGA	-139	1,1	6.79	-4.70	*no*	42	Rmet_0550	*0*	Op0153f_1	NA
2978	TSS_2914529+2	57	11	0,1	-16	1	-42	CCAGCACGAC	-14	TGCAAAATGC	-140	5,1	10.07	-4.60	*no*	25	Rmet_2681	*nrdR*	Op0757f_1	NA
2979	TSS_3606318+2	57	18	0,1	-16	1	-41	TCGAAGTCAT	-14	TGTAGACTTC	-140	1,1	7.98	0.90	*(w)-sba*	11698	Rmet_6590	*0*	Op0931f_1	NA
2980	TSS_1900579-2	57	8	0,1	-15	1	-37	TTGTGCGGCT	-13	CATATACTTT	-140	1,1	8.12	9.40	s	3149	Rmet_1749	*agrB*	Op0498r_1	NA

2981	TSS_2972658-2	57	12	0,1	-15	1	-35	TTGCAGCGAT	-13	GCTAGGGTGG	-139	1,1	7.26	10.20	s	1183	Rmet_2735	*rfbB*	Op0770r_1	MET-CAH-NucSug
2982	TSS_1796065+2	57	7	0,1	-16	1	-41	TTGCTCTTTT	-14	TCTAAACTCC	-141	1,1	9.10	1.40	*(w)-sba*	159	Rmet_6462	*0*	Op0469f_1	NA
2983	TSS_3201358+2	57	12	0,2	-10	1	-28	TTTCGGCGCG	-4	GCTGGCCTGA	-143	2,1	5.20	-2.30	*no*	161	Rmet_2945	*yajC*	Op0831f_2	GIP-PTL-Exp
2984	TSS_3424995+2	57	17	0,1	-16	1	-36	TGGCACAGGC	-14	GATAGAGTTC	-138	1,1	7.45	9.90	s	362	Rmet_3160	*0*	Op0885f_1	NA
2985	TSS_3485367+2	57	10	0,1	-22	0	-36	TTGCGTGCTG	-11	CTGGTACTGG	-145	1,1	-0.21	6.40	s	1726	Rmet_6585	*0*	Op2051f_1	NA
2986	TSS_1807334-2	57	19	0,1	-16	1	-41	TCGAGTAAGT	-14	GCAACACTCG	-140	1,1	5.44	-0.10	*no*	3415	Rmet_1667	*fimA*	Op0468r_1	NA
2987	TSS_1279488+3	57	18	0,1	-16	1	-49	TTGCTGTCAT	-14	ATCAAAATGT	-144	1,1	7.80	-14.60	*no*	1651	Rmet_4650	*0*	Op1344f_1	NA
2988	TSS_374399-3	57	11	0,1	-16	1	-41	TTGAATTTCT	-14	GCTAGAGTGG	-138	1,1	8.92	2.40	*(w)-sba*	54	Rmet_3832	*bugT*	Op1065r_1	NA
2989	TSS_355817+2	56	8	0,1	-16	1	-41	TTGCCATTGA	-14	CCTAAGATTC	-142	1,1	9.51	3.40	*(m)-sba*	32	Rmet_0338	*0*	Op0095f_1	NA
2990	TSS_1861374+2	56	11	0,1	-16	1	-36	TGGTCGGCGC	-14	GTCATACTGT	-142	1,1	6.55	10.90	s	54	Rmet_1719	*bug*	Op0485f_2	NA
2991	TSS_3265913+2	56	6	0,1	-16	1	-44	CCGGCAGCAG	-14	GATATTCTTC	-138	5,1	9.74	-7.10	*no*	6143	Rmet_3013	*yodB*	Op0847f_1	NA
2992	TSS_3560915+2	56	8	0,1	-8	0	-33	TCGCAATGCG	-6	GTCAGATTTG	-144	1,1	5.52	2.70	*(w)-ba*	244	Rmet_3285	*engB*	Op0925f_1	NA
2993	TSS_3516787-2	56	10	0,1	-15	1	-45	TTCAGAGCCA	-13	GCCAAACTGA	-144	1,1	6.31	-8.10	*no*	73	Rmet_3243	*hisA*	Op0918r_3	MET-AA-His
2994	TSS_375584+3	56	12	0,1	-16	1	-35	TTCACAGGTC	-14	GGTAGATTGG	-139	1,1	8.19	12.10	s	26	Rmet_3834	*0*	Op1066f_1	NA
2995	TSS_2491321+3	56	5	0,1	-10	1	-35	TCGAACCAGT	-8	ATAAGGATTC	-147	1,1	3.77	4.70	*(m)-ba*	1680	Rmet_6764	*0*	Op1716f_1	NA
2996	TSS_125693-3	56	10	0,1	-4	0	-36	TCGGAACGAT	-2	CGTAACTTTG	-142	5,1	7.53	-2.10	*no*	22	Rmet_5923	*0*	Op1799r_1	NA
2997	TSS_211859+2	56	7	0,1	-15	1	-36	TTCACAATGA	-13	TGCAAGCTGT	-147	1,1	5.57	9.90	s	92	Rmet_0203	*0*	Op0059f_3	NA
2998	TSS_666430+2	56	10	0,1	-16	1	-37	TCGCCAAGCC	-14	GCTACACTCA	-135	1,1	8.57	9.90	s	33	Rmet_0612	*0*	Op0173f_1	NA
2999	TSS_1011314+2	56	14	0,1	-1	0	-32	GAATTCGACA	-12	GGTCTGTGTT	-144	3,0	5.74	-3.40	no	413	Rmet_0926	*secG*	Op0267f_2	GIP-PTL-Exp
3000	TSS_1343494+2	56	7	0,1	-17	1	-35	TTGCATAATA	-15	AATAAAATGC	-139	1,1	9.84	10.60	s	108	Rmet_1224	*bzdA*	Op0355f_1	MET-XEN-Benz
3001	TSS_3434233+2	56	10				-35	TTGCGCCCTG	-14	TCTACTATTT	-137	1,1	7.81	10.60	s	95	Rmet_3163	*paaG*	Op0887f_1	MET-CAH-Prop
3002	TSS_565659-2	56	15				-34	TTGTACCGGG	-13	TCTACAGTAC	-142	1,1	6.98	9.60	s	54	Rmet_0529	*0*	Op0146r_1	NA
3003	TSS_823560-2	56	6				-28	TTCCACGCGC	-7	TCTACATTTG	-137	1,1	7.09	2.10	*(w)-nu*	224	Rmet_6427	*0*	Op0210r_1	NA
3004	TSS_1737017-2	56	7				-33	TGGAAAATTA	-13	GGTACCATCG	-140	1,1	8.89	8.10	s	1738	Rmet_1608	*0*	Op0448r_1	NA
3005	TSS_756453+3	56	9				-44	TTCCCGGGAT	-14	GTTACTATAT	-145	1,1	7.46	-5.10	*no*	55	Rmet_4170	*0*	Op1174f_1	Met-SecMetab
3006	TSS_1636977+3	56	9				-43	TTGCCCTTGT	-14	GAGATAATAG	-141	1,1	9.17	-0.60	*no*	3220	Rmet_4967	*0*	Op1458f_1	NA
3007	TSS_2519764+3	56	15				-37	TGGCTGGCCA	-15	CGTAAAGTGC	-139	1,1	6.04	8.90	s	2589	Rmet_5761	*0*	Op1728f_1	NA
3008	TSS_1882221-3	56	12				-42	TCGTCCATCA	-13	TCTATGCTAA	-146	1,1	5.03	-2.10	*no*	25	Rmet_5187	*0*	Op1529r_1	NA
3009	TSS_13519-4	56	4				-34	TTGACCGGGT	-13	AACACAGTCC	-141	1,1	6.32	10.60	s	11	Rmet_6055	*0*	Op1844r_4	NA
3010	TSS_618990+2	56	8				-34	TGAAAGGCGC	-14	GCTATTCTTG	-145	4,1	8.40	6.60	s	20	Rmet_0577	*0*	Op0163f_1	MET-NUC-Pyr
3011	TSS_2291430+2	56	12				-35	GCCGACGCGA	-13	ATTATCATCG	-141	4,1	7.10	6.20	m	1371	Rmet_2111	*0*	Op0609f_1	NA
3012	TSS_2850116+2	56	13				-42	TTGCATTGGT	-15	GCTAGAATCC	-136	1,1	11.09	1.40	*(w)-sba*	93	Rmet_2619	*0*	Op0733f_1	NA
3013	TSS_1004827-2	56	9				-38	TTCTATTCGA	-13	AGTATTCTTA	-143	1,1	5.07	4.90	m	8399	Rmet_0910	*rpoR*	Op0262r_1	GIP-TK-RNAP
3014	TSS_1776092-2	56	22				-34	TGGCGGTACG	-13	CCTACATTGA	-140	1,1	6.44	8.10	s	25	Rmet_1643	*0*	Op0462r_2	NA
3015	TSS_1210790+3	56	14				-37	TTCAGCCAAC	-15	TCTACAATTT	-137	1,1	9.00	10.90	s	273	Rmet_4592	*0*	Op1320f_2	NA
3016	TSS_1927484+3	56	3				-39	TAGAGCGCAC	-14	CATATACTTT	-143	1,1	4.67	5.90	m	1041	Rmet_5231	*0*	Op1544f_1	NA
3017	TSS_688923-3	56	2				-41	TCAGAATGAC	-14	GGCAAGATCG	-143	5,1	6.93	-1.60	*no*	45	Rmet_4117	*pdxR*	Op1159r_2	NA
3018	TSS_833644-3	56	5				-43	TTGCAAACAG	-14	CCTAAAATGT	-141	1,1	10.47	-0.60	*no*	5005	Rmet_4240	*acnA3*	Op1201r_1	NA
3019	TSS_1540951-3	56	15				-34	TGGACGCTTA	-13	TACATACTCC	-144	1,1	6.49	10.10	s	38	Rmet_4878	*catB*	Op1425r_1	MET-XEN-Toluene
3020	TSS_1822283-3	56	13				-37	TTGCGCTCGA	-13	GCTATGCTGT	-137	1,1	8.80	8.40	s	18	Rmet_5126	*0*	Op1511r_1	NA
3021	TSS_2182204+2	55	4				-35	TGCTCTTAAA	-15	GAGACAATTA	-145	1,1	5.22	7.60	s	1019	Rmet_6502	*0*	Op2024f_1	NA
3022	TSS_2967381+2	55	7				-30	AAATTCGCCG	-10	AATACGCAGC	-144	3,0	9.13	-4.40	*no*	4296	Rmet_2736	*apaH*	Op0771f_1	MET-NUC-Pur
3023	TSS_3632753+2	55	1				-42	TCGCACCGTT	-14	GGTATCCTGG	-142	1,1	7.64	-2.10	*no*	345	Rmet_3357	*0*	Op0935f_2	NA
3024	TSS_506811-2	55	13				-34	TCGACCCCCA	-13	CCTATATTGG	-143	1,1	6.95	11.10	s	47	Rmet_0477	*0*	Op0124r_1	NA
3025	TSS_835163-2	55	4				-48	TTGCCAACGG	-14	GCTAGAATGG	-135	1,1	11.34	-9.60	*no*	36	Rmet_0756	*0*	Op0216r_1	NA
3026	TSS_113774+3	55	8				-43	TTCCAGCGGG	-15	CGTAGGATGC	-140	1,1	7.21	-3.10	*no*	1498	Rmet_5913	*ogt3*	Op1794f_1	GIP-REP-Recomb
3027	TSS_224099+3	55	21				-49	TGCCGCCGCC	-23	TCGAAGCTGC	-141	1,1	2.65	-10.60	*no*	176	Rmet_3684	*0*	Op1026f_2	NA
3028	TSS_577223+3	55	15				-37	TGGCGACTGG	-14	GTCATAATGC	-138	1,1	8.84	9.90	s	5025	Rmet_6640	*0*	Op2063f_1	NA
3029	TSS_1169820+3	55	3				-35	TACTGATGCC	-14	CGTAGACTTT	-147	1,1	2.59	8.60	s	20	Rmet_4560	*0*	Op1310f_1	NA
3030	TSS_2112128+3	55	13				-37	TGGCAGCGCC	-15	GTCATGCTGG	-141	1,1	5.82	7.90	s	57	Rmet_5387	*0*	Op1604f_1	NA
3031	TSS_1670332-3	55	7				-45	TTGAAGTAGT	-13	CATACGCTTG	-140	1,1	7.94	-7.60	*no*	150	Rmet_4991	*0*	Op1465r_1	NA
3032	TSS_101961-5	55	8				-44	TTGCCACTGT	-13	ATTAAGATTG	-140	1,1	9.11	-3.60	*no*	7491	Rmet_6246	*0*	Op1903r_1	NA
3033	TSS_1800801+2	55	16				-47	TGGCGGATTA	-15	CCGATAATGG	-143	1,1	7.22	-10.10	*no*	4102	Rmet_1668	*tnp*	Op0469f_1	NA
3034	TSS_762224-2	55	30				-37	TTCCCTACAG	-12	CCGATATTTC	-141	1,1	4.97	6.90	s	105	Rmet_0689	*0*	Op0194r_1	NA
3035	TSS_508599+3	55	11				-35	TTGCACTGCC	-15	TCCATAATGA	-144	1,1	9.22	10.60	s	32	Rmet_3951	*0*	Op1108f_1	NA
3036	TSS_2278797+3	55	2				-35	TTGAGTTCTA	-14	CGCAAGCTAC	-144	1,1	4.85	9.60	s	259	Rmet_5532	*0*	Op1644f_1	NA
3037	TSS_2507542-3	55	14				-38	TCGCGCGTGT	-13	GCTATTCTGT	-142	1,1	7.13	4.90	m	1691	Rmet_5743	*0*	Op1717r_1	MET-CAH-FbP
3038	TSS_129981+4	55	8				-42	TTGTCCTTTC	-15	CGTAACCTTA	-144	1,1	6.57	0.40	*(w)-sba*	24	Rmet_6171	*merR*	Op1889f_1	NA
3039	TSS_120668+5	55	10				-23	TTGATCGGTG	0	GAGACGATTG	-143	1,1	6.36	-1.80	*no*	388	Rmet_6264	*0*	Op1910f_1	NA
3040	TSS_462657+2	55	8				-46	TCGAATCGGA	-14	GGCACGATTG	-140	1,1	6.29	-10.10	*no*	597	Rmet_0437	*surA*	Op0117f_1	MET-COF-B6
3041	TSS_1002437+2	55	11				-33	GTCAACGTCG	-13	GTCACAATGG	-143	4,1	6.89	4.10	m	461	Rmet_0918	*leuA*	Op0263f_4	MET-CAH-Pyr

3042	TSS_1260657+2	55	12	-38	TCGCCACCGC	-13	GGTATGGTTT	-140	1,1	6.74	6.90	s	240	Rmet_1150		*0*	Op0329f_2	NA
3043	TSS_1549535+2	55	15	-35	TCGACAACGC	-14	AGTATGGTGG	-138	1,1	6.41	12.10	s	70	Rmet_1431		*0*	Op0401f_1	NA
3044	TSS_1950924+2	55	24	-36	GTAGCAGTAG	-13	AGTAAATAGC	-151	4,1	1.72	5.90	m	2249	Rmet_1801	*bug*		Op0523f_1	NA
3045	TSS_2913081+2	55	11	-34	TTCCCCGCGC	-14	GCGAAAATCC	-137	1,1	7.45	8.10	s	1473	Rmet_2681	*nrdR*		Op0757f_1	NA
3046	TSS_1506090-2	55	29	-34	TTGCCGGGCG	-13	AGTACGTTGG	-140	1,1	6.65	9.60	s	2139	Rmet_1394		*0*	Op0390r_1	NA
3047	TSS_3275756-2	55	5	-35	TTGCGCGCAT	-14	CGCAGCATAA	-142	1,1	5.13	9.60	s	304	Rmet_3016	*hmzR*		Op0848r_4	NA
3048	TSS_1608499+3	55	9	-36	TCGAGGAATA	-15	AGTATCTTTG	-141	1,1	5.45	10.30	s	79	Rmet_4943		*0*	Op1452f_1	MET-CAH-FbP
3049	TSS_1828653-3	55	20	-39	TTCAGCTTCT	-14	CGCAACATGC	-140	1,1	5.92	3.90	m	359	Rmet_5130		*0*	Op1513r_3	EIP-SIG-2Comp
3050	TSS_2348544-3	55	11	-37	TAGATAAGCA	-13	AGCACAATCA	-149	1,1	3.73	8.90	s	945	Rmet_5592		*0*	Op1665r_1	EIP-TRA-Other
3051	TSS_73647-4	55	23	-37	TTGTGCAGGG	-14	CGGACAATGA	-140	1,1	6.97	9.40	s	482	Rmet_6367		*0*	Op1822r_1	NA
3052	TSS_120724-5	55	3	-33	TTGCGACAAA	-13	ACTATCATGA	-139	1,1	8.71	9.60	s	3444	Rmet_6262		*0*	Op1909r_1	NA
3053	TSS_1321766+2	54	15	-47	TTGCAATCCG	-13	GCTCATATCG	-143	1,1	4.21	-12.60	*no*	532	Rmet_1203		*0*	Op0349f_1	NA
3054	TSS_1723586+2	54	1	-36	TCGACCGATG	-14	AGTACCATCT	-140	1,1	6.79	11.90	s	914	Rmet_1599		*0*	Op0443f_3	NA
3055	TSS_267999-2	54	10	-34	TGGCAACAGC	-13	CGTAAAGTAT	-141	1,1	6.63	9.10	s	364	Rmet_0250	*ctpA*		Op0072r_1	NA
3056	TSS_1331738-2	54	6	-40	TTCAACCCCG	-13	GCGACAATCG	-139	1,1	7.01	1.90	*(w)-sba*	884	Rmet_1211		*0*	Op0352r_2	EIP-TRA-ABC
3057	TSS_1563972-2	54	12	-42	TGGAACAGGC	-12	GCAATAATAC	-142	1,1	7.48	-2.10	*no*	8060	Rmet_1434	*map*		Op0402r_1	GIP-TL-Ass
3058	TSS_1998543-2	54	19	-39	TTGACGATGG	-13	GGTAATATCG	-136	1,1	9.71	6.40	s	167	Rmet_1843		*0*	Op0534r_1	MET-CAH-But
3059	TSS_498020+3	54	1	-45	TTGATCAACA	-15	AGTATCGTGC	-140	1,1	6.72	-4.60	*no*	2221	Rmet_3941		*0*	Op1102f_1	NA
3060	TSS_662003-3	54	17	-34	TTGCAAAGCA	-13	AGTAGGATGA	-142	1,1	8.39	10.60	s	23	Rmet_4092		*0*	Op1151r_1	NA
3061	TSS_1137041-3	54	10	-39	TTGCATTCCT	-13	CTCATACTGC	-145	1,1	7.81	4.40	m	30	Rmet_4529	*sumF1*		Op1297r_3	NA
3062	TSS_1461867-3	54	8	-34	TTGCGAGACG	-13	GCTATCGTTT	-141	1,1	7.56	10.60	s	21	Rmet_4813		*0*	Op1401r_1	NA
3063	TSS_92323+5	54	8	-42	TTCATTCGCG	-14	GGTACGGTGT	-140	2,1	9.02	-2.10	*no*	2145	Rmet_6247		*0*	Op1904f_1	NA
3064	TSS_26972+2	54	4	-28	GCGGCAGTTT	-5	GTCACACTTC	-141	5,1	8.96	-0.30	*no*	55	Rmet_0023	*fsr*		Op0005f_1	NA
3065	TSS_665513+2	54	11	-46	TCGGACAGGC	-16	CCTATAATTC	-137	5,1	11.58	-6.10	*no*	32	Rmet_0611		*0*	Op0173f_1	NA
3066	TSS_1998434+2	54	7	-36	TCGCACAGAC	-15	GCGATGATCG	-143	1,1	6.46	9.30	s	81	Rmet_1844		*0*	Op0535f_1	NA
3067	TSS_3504246+2	54	4	-43	GTCAGAACGC	-19	AATAAGTTAC	-146	4,1	6.41	-1.10	*no*	3172	Rmet_3231	*mscL*		Op0915f_1	EIP-TRA-Pores
3068	TSS_3685308+2	54	9	-48	TGGTAAGCCA	-21	CCGAAGATGC	-147	1,1	4.33	-7.10	*no*	201	Rmet_3407		*0*	Op0947f_1	MET-LIP-Others
3069	TSS_575899-2	54	19	-37	CCGACCGGAT	-14	GCTATTTTTC	-140	5,1	7.24	6.90	s	4088	Rmet_0537	*exbB2*		Op0148r_1	NA
3070	TSS_1801709-2	54	3	-39	TGGACTTGGG	-13	GGAACAATAT	-143	1,1	6.58	4.90	m	1965	Rmet_1664		*0*	Op0468r_2	NA
3071	TSS_2759451-2	54	16	-36	TGGCTGGTGC	-14	ACTATAATGT	-137	1,1	9.04	11.90	s	50	Rmet_2534		*0*	Op0700r_1	NA
3072	TSS_2957448-2	54	13	-40	TTGCCACCAG	-15	GGCATAATCC	-141	1,1	10.13	7.40	*(s)-sba*	459	Rmet_6553		*0*	Op2041r_1	NA
3073	TSS_1786935-3	54	6	-49	TGGCCGGCGT	-12	GGTATGCTGC	-136	1,1	8.32	-17.10	*no*	101	Rmet_5095		*0*	Op1499r_2	NA
3074	TSS_2884814+2	54	7	-40	TCGCAAGGCT	-14	ACTATGATGC	-142	1,1	8.24	3.90	*(m)-sba*	38	Rmet_2648		*0*	Op0749f_1	NA
3075	TSS_3511546+2	54	9	-41	GGCGACGCAT	-13	CGTATTATGT	-144	4,1	8.24	-4.60	*no*	19823	Rmet_3261		*0*	Op0919f_1	NA
3076	TSS_3715421+2	54	9	-39	TGGAAATCCA	-14	GATAAGATGT	-140	1,1	8.26	5.90	m	30	Rmet_3441		*0*	Op0957f_1	NA
3077	TSS_1032274-2	54	8	-46	TCGCCCTGCA	-13	GCGATAGTAC	-143	1,1	5.29	-10.10	*no*	27204	Rmet_0919		*0*	Op0264r_1	NA
3078	TSS_1901919-2	54	3	-40	GGAGGGAAAC	-13	GATACAGTTC	-142	4,1	9.34	-3.60	*no*	1295	Rmet_1750	*agrA*		Op0498r_1	NA
3079	TSS_419151+3	54	8	-38	TGCCGACAAT	-15	TCTACTATCC	-138	1,1	6.35	6.40	s	132	Rmet_3876		*0*	Op1080f_1	NA
3080	TSS_852265-3	54	8	-34	TTGCTGCATT	-13	CTTAGTATTG	-141	1,1	7.26	9.60	s	59	Rmet_4261		*0*	Op1207r_1	NA
3081	TSS_2415486-3	54	16	-48	GGAGAGGCGG	-12	GCTATTGTAT	-142	4,1	6.31	-20.60	*no*	232	Rmet_5656		*0*	Op1689r_1	NA
3082	TSS_181838+2	53	9	-33	GAACTTATTG	-13	CGACACATGG	-142	3,0	5.43	-1.40	no	542	Rmet_0176		*0*	Op0053f_1	NA
3083	TSS_427947+2	53	4	-35	TTGCATGATC	-14	TGCACCGTTC	-143	1,1	4.87	8.60	s	234	Rmet_0400	*gltJ*		Op0109f_4	NA
3084	TSS_1279407+2	53	4	-42	TCGGACATAG	-14	ACTAGCCTTT	-145	5,1	6.84	-3.10	*no*	2916	Rmet_1171	*tnpA*		Op0335f_1	NA
3085	TSS_2128462+2	53	10	-34	TGGCCTGATG	-14	TGTACGATCA	-139	1,1	7.04	8.10	s	3	Rmet_1956	*argE1*		Op0573f_2	MET-AA-Urea
3086	TSS_3293440+2	53	19	-33	TGGAATTCCC	-12	AATATCCTCA	-142	1,1	5.56	8.10	s	3957	Rmet_3042		*0*	Op0855f_1	NA
3087	TSS_3518934-2	53	13	-42	TGGCCCGGAA	-13	AGTAAAATGC	-139	1,1	9.24	-1.10	*no*	98	Rmet_3246	*hisB*		Op0918r_3	MET-AA-His
3088	TSS_2282949-3	53	7	-34	TGGTGTTAAT	-13	TGTATAGTTG	-143	1,1	6.98	9.10	s	157	Rmet_5535		*0*	Op1645r_2	NA
3089	TSS_2779-5	53	5	-35	TCGCTTTTTC	-14	TGTAGGCTCC	-143	1,1	5.32	8.10	s	16879	Rmet_6301	*trbF*		Op1917r_1	NA
3090	TSS_467840+2	53	16	-38	TTCACTACGC	-15	AGCACAATCC	-138	1,1	7.35	8.90	s	1335	Rmet_6417		*0*	Op0117f_2	NA
3091	TSS_3547874+2	53	12	-43	TTGCATAGCG	-15	GCGATCCTTG	-142	1,1	6.34	-2.60	*no*	202	Rmet_3274	*mrcA*		Op0921f_1	NA
3092	TSS_3860324+2	53	17	-37	TTGACGGCTT	-14	GGCAGAATCT	-138	1,1	8.91	11.40	s	7490	Rmet_3566		*0*	Op0989f_1	NA
3093	TSS_3093775-2	53	17	-36	TTCCCCATAT	-15	GGTAAATTCC	-141	1,1	8.37	10.30	s	714	Rmet_6563		*0*	Op2045r_1	NA
3094	TSS_523967+3	53	6	-40	TGAAAGGAGC	-15	TCTACTGTTC	-144	4,1	6.30	1.40	*(w)-sba*	61	Rmet_3963		*0*	Op1112f_2	NA
3095	TSS_808488+3	53	21	-36	TTGTTTGCAT	-15	AACAACATGG	-147	1,1	4.66	9.80	s	19	Rmet_4223	*bug*		Op1198f_3	NA
3096	TSS_1259342+3	53	11	-40	TGCCGATGGG	-15	GCTAGGATTC	-138	1,1	7.11	2.40	*(w)-sba*	1623	Rmet_4631		*0*	Op1336f_1	NA
3097	TSS_879007-3	53	10	-36	CCTTAAGGGG	-13	CCTAGAATAT	-143	5,1	7.76	6.40	s	210	Rmet_4287	*rlpA*		Op1219r_2	NA
3098	TSS_1766605-3	53	15	-38	CCGGCACGCG	-13	GATAAGCTCA	-140	5,1	9.13	2.90	w	554	Rmet_5077		*0*	Op1495r_1	NA
3099	TSS_102994+4	53	14	-46	GAACAAGCGC	-14	TGTACCCTGC	-145	4,1	3.39	-14.60	*no*	69	Rmet_6373		*0*	Op1813f_3	NA
3100	TSS_140004+4	53	6	-44	GTACAAGCAG	-13	CCTAAAATGG	-143	4,1	10.36	-8.10	*no*	48	Rmet_6377		*0*	Op2113f_1	NA
3101	TSS_1967683+2	53	2	-34	TTGTTCTGCT	-13	GCGATTGTTT	-141	1,1	4.10	8.60	s	3521	Rmet_1817		*0*	Op0527f_1	NA
3102	TSS_2419990+2	53	10	-35	TGCCCGTCGT	-15	GATATAGTTG	-138	1,1	7.08	8.60	s	41	Rmet_2207		*0*	Op0631f_1	NA

3103	TSS_2837546+2	53	19	-48	GCAAACCCGA	-14	TGTAGCCTTC	-141	4,1	6.38	-18.60	no	880	Rmet_6542	0	Op2038f_1	NA	
3104	TSS_3507387+2	53	7	-46	TTGCACTCGA	-15	GTCAAACTCA	-143	1,1	7.51	-8.60	no	31	Rmet_3231	mscL	Op0915f_1	EIP-TRA-Pores	
3105	TSS_3696541+2	53	8	-32	TTGCCCTCGG	-6	GATATCTTTT	-142	1,1	7.58	5.20	(m)-ba	103	Rmet_3416	0	Op0949f_1	NA	
3106	TSS_1158015-2	53	17	-49	GCACGGCCGC	-14	CGCATCATGC	-140	4,1	7.65	-20.60	no	733	Rmet_1063	trxB1	Op0306r_1	MET-NUC-Pyr	
3107	TSS_277500+3	53	29	-43	TTCCAGCGAT	-15	CCTAAGATGG	-139	1,1	7.59	-3.10	no	101	Rmet_3734	flgB	Op1042f_2	DIV-MOT-Flagel	
3108	TSS_1819476+3	53	10	-35	TTGACGGGAA	-13	TCGAACATTG	-144	1,1	6.60	12.20	s	333	Rmet_5125	0	Op1510f_1	NA	
3109	TSS_1990394+3	53	7	-36	TGGCAGATTG	-14	ACTATGCTTT	-142	1,1	6.68	9.90	s	264	Rmet_5289	0	Op1560f_3	NA	
3110	TSS_2311423+3	53	8	-33	TTGCGTTACC	-3	GTCAATCTTT	-144	1,1	5.69	-0.80	no	4277	Rmet_5563	0	Op1652f_1	NA	
3111	TSS_649351-3	53	21	-34	TGGCACATCG	-13	GGCATGATGT	-140	1,1	7.24	8.10	s	37	Rmet_4079	0	Op2065r_1	NA	
3112	TSS_1360598-3	53	9	-34	TTCTGTCATG	-13	GGTAAGGTGG	-141	1,1	4.72	7.10	s	634	Rmet_4716	0	Op1361r_1	NA	
3113	TSS_1652775-3	53	5	-34	TTGCACCTGT	-13	TCGATTATGT	-142	1,1	7.11	9.60	s	3847	Rmet_4971	0	Op1459r_1	NA	
3114	TSS_1809779-3	53	8	-33	GGCGAGCAGC	-13	AGTACCATGC	-139	4,1	8.07	1.60	w	2598	Rmet_5113	bug	Op1505r_1	NA	
3115	TSS_110484-5	53	11	-35	CCTGAAACGC	-14	CGTACGATAT	-141	5,1	8.45	5.60	m	383	Rmet_6257	0	Op1905r_1	NA	
3116	TSS_2257+2	52	8	-36	TGCAATTGGT	-15	GAGACAATCT	-143	1,1	6.20	8.80	s	1263	Rmet_0003	gyrB	Op0001f_2	GIP-REP-Complex	
3117	TSS_708507+2	52	16	-36	TGGACCATCG	-14	GGGATGATGG	-139	1,1	7.10	11.90	s	11158	Rmet_0654	0	Op0181f_1	NA	
3118	TSS_2891165+2	52	15	-38	TTGAACCAAC	-15	GAGAAACTAA	-142	1,1	6.41	8.40	s	414	Rmet_2656	0	Op0753f_1	NA	
3119	TSS_3150325+2	52	16	-35	TGGCAAGCGG	-14	GGTTCAATGA	-145	1,1	3.69	9.10	s	2067	Rmet_2896	rluE	Op0815f_1	GIP-TL-Ass	
3120	TSS_169595-2	52	7	-42	TCGGCAGTGT	-14	CGTACCATGA	-138	5,1	9.46	-0.10	no	36	Rmet_0161	aidB	Op0048r_2		0
3121	TSS_2737436-2	52	5	-41	TTGCGGCGGT	-15	TTTAGAATCC	-138	1,1	9.90	3.40	(m)-sba	59	Rmet_2519	parC	Op0696r_1	GIP-REP-Complex	
3122	TSS_3060473-2	52	7	-37	TTCTAAATCG	-12	ACGACAATGT	-143	1,1	6.05	6.90	s	1337	Rmet_2811	cysG	Op0792r_2	MET-COF-Porph	
3123	TSS_3685250-2	52	9	-47	TTGCATCTTC	-15	CTTACCATTT	-142	1,1	8.02	-9.60	no	30	Rmet_3406	fieF	Op0946r_1	EIP-TRA-Ion	
3124	TSS_3784200-2	52	14	-37	TCGAAGGAAC	-14	GCTACTCTTA	-139	1,1	6.39	7.90	s	511	Rmet_3492	0	Op0970r_2	MET-CAH-But	
3125	TSS_1083878+3	52	4	-38	TGGCACGCGT	-15	TTTACCATCT	-140	1,1	7.25	6.90	s	1775	Rmet_6667	0	Op2071f_1	NA	
3126	TSS_1554617+3	52	7	-37	TTTACATGGC	-16	AATAGGCTTT	-150	1,1	0.67	10.30	s	63	Rmet_4891	sorA	Op1430f_1	NA	
3127	TSS_76523-3	52	8	-46	TCGCAGGCTC	-25	CGGATGATTT	-146	1,1	5.10	-0.70	no	29	Rmet_5879	0	Op1779r_1	NA	
3128	TSS_122696-3	52	10	-47	TGGCGCATGG	-21	ACCACACTGC	-142	1,1	6.18	-6.10	no	34	Rmet_5919	0	Op1797r_1	NA	
3129	TSS_309412-3	52	8	-43	TGGCGCCACA	-13	GGTAGATTGA	-143	1,1	6.69	-5.10	no	2915	Rmet_3769	alkB1	Op1047r_1	NA	
3130	TSS_900636-3	52	12	-38	TTCCTGTGCG	-12	AGTAGCATTT	-140	2,1	11.00	3.90	m	344	Rmet_4305	0	Op1227r_1	NA	
3131	TSS_57923+2	52	19	-40	TTCCGAGCGT	-14	GCAACACTGG	-138	1,1	5.95	1.90	(w)-sba	563	Rmet_0051	mreB	Op0017f_1	DIV-Division	
3132	TSS_1562462+2	52	2	-37	TTGAGCGCTT	-15	GCTAGCCTTT	-139	1,1	7.49	10.40	s	269	Rmet_1442	yaeL	Op0403f_2	NA	
3133	TSS_596065+3	52	4	-41	TTAAAGGCGC	-13	GCTACACTTG	-140	4,1	9.60	-0.10	no	25	Rmet_4027	0	Op1134f_1	MET-AA-ArgPro	
3134	TSS_1041816+3	52	5	-38	TTGCGCGGAC	-13	TATACACTCG	-139	1,1	8.73	6.40	s	22	Rmet_4445	0	Op1264f_1	NA	
3135	TSS_2018175+3	52	11	-36	TGGCGTAGCC	-15	CCTACGCTGC	-137	1,1	6.24	8.30	s	117	Rmet_5311	0	Op1570f_1	NA	
3136	TSS_2160897+3	52	9	-35	TTCCGTTGAT	-14	GCGATACTTC	-145	1,1	7.08	9.10	s	38	Rmet_5416	aslA	Op1612f_2	MET-LIP-Others	
3137	TSS_443529+2	52	14	-36	TTCCTTTATC	-14	GCTACGATTC	-141	1,1	7.93	9.90	s	666	Rmet_0418	dtd	Op0115f_1	NA	
3138	TSS_2934319+2	52	7	-49	TTGCAAACGG	-14	TATATATTGC	-139	1,1	9.26	-12.60	no	31	Rmet_2701	phaZ2	Op0763f_1	NA	
3139	TSS_1073685-2	52	11	-39	TCGCCGGGCA	-13	GGCAATATCC	-143	1,1	5.80	2.90	w	1140	Rmet_0986	0	Op0278r_1	NA	
3140	TSS_1727051-2	52	8	-34	TGGCCGGCAC	-14	GGCAGAATGA	-137	1,1	7.47	8.10	s	9845	Rmet_1592	tnpA	Op2009r_1	NA	
3141	TSS_2509487-2	52	8	-49	TGGCTGAACA	-28	ATCAACATCA	-143	1,1	4.04	-4.70	no	968	Rmet_R0038	0	Op0654r_2	NA	
3142	TSS_1483091+3	52	10	-46	TGCCAATATC	-15	TATAAGATGC	-145	1,1	6.85	-9.60	no	694	Rmet_4830	qseC	Op1408f_1	EIP-SIG-2Comp	
3143	TSS_106009+2	51	15	-38	TTCTGTTTTG	-14	GCTATATTGA	-139	1,1	6.84	6.90	s	796	Rmet_0100	0	Op0029f_1	NA	
3144	TSS_269994+2	51	17	-43	TCGAAAGAGA	-14	GCCACTATGG	-139	1,1	6.59	-3.10	no	727	Rmet_0253	0	Op0073f_2	GIP-REP-Recomb	
3145	TSS_2771860+2	51	11	-34	TGGAATGATG	-3	CACAGCATCA	-147	1,1	3.84	-0.30	no	433	Rmet_2547	0	Op0707f_2	NA	
3146	TSS_3010015+2	51	8	-34	TTGTGCACGC	-13	CCTATTGTAG	-144	1,1	5.20	9.60	s	1147	Rmet_2769	metG	Op0777f_1	MET-AA-Met	
3147	TSS_1373673-2	51	11	-33	GTCAGCCCCA	-13	GAAACAATGG	-145	4,1	4.28	4.10	m	915	Rmet_1249	0	Op0360r_1	NA	
3148	TSS_2356710-2	51	8	-32	TTGTGGAGGG	-1	GTTCGAATCC	-145	1,1	4.65	-1.80	no	14718	Rmet_2148	0	Op0620r_1	NA	
3149	TSS_3861442-2	51	14	-35	GCCGCACCGG	-14	TGTACATTGG	-141	4,1	7.24	6.60	s	1432	Rmet_3559	0	Op0988r_3	NA	
3150	TSS_1745959+3	51	8	-35	TGCACGGCTT	-14	GGCATGGTCT	-143	1,1	3.58	8.60	s	1112	Rmet_5056	0	Op1488f_1	NA	
3151	TSS_339969-3	51	21	-45	TTGCGCGAAT	-14	GGGATAATCG	-139	1,1	9.39	-5.60	no	18	Rmet_3799	0	Op1055r_1	NA	
3152	TSS_1621112-3	51	19	-33	TGGCAAATAC	-13	GATAAACTGT	-139	1,1	9.04	7.10	s	75	Rmet_4954	0	Op1455r_1	NA	
3153	TSS_170449-4	51	14	-46	TCCAGGACAA	-13	AGTAAAATCA	-144	1,1	6.57	-10.60	no	31	Rmet_6133	silD	Op1874r_1	NA	
3154	TSS_191954-4	51	12	-48	TTGCCCGCAG	-14	GTTACTATCG	-141	1,1	8.81	-11.60	no	755	Rmet_6112	copA1	Op1866r_1	NA	
3155	TSS_1249542+2	51	23	-49	TGCTCTCGGA	-25	GTCACATTGG	-142	1,1	3.72	-6.60	no	42	Rmet_1142	0	Op0327f_1	NA	
3156	TSS_2896760+2	51	8	-47	TTCAGCCCAG	-26	GCCAAAATGC	-139	1,1	7.69	-0.70	no	2958	Rmet_2665	0	Op0755f_1	NA	
3157	TSS_3619801+2	51	3	-46	TCAGCAAGCT	-14	TTTATCCTTT	-141	5,1	9.03	-9.60	no	1077	Rmet_3344	0	Op0931f_4	NA	
3158	TSS_3517529-2	51	5	-34	TTGCCTGTTT	-14	ACTACCATAG	-143	1,1	7.92	9.60	s	815	Rmet_3243	hisA	Op0918r_3	MET-AA-His	
3159	TSS_3616097-2	51	21	-35	TTGCCAACAG	-5	TTAACAATAT	-145	1,1	6.98	5.20	(m)-ba	181	Rmet_R0061	0	Op0930r_1	NA	
3160	TSS_3882560-2	51	16	-45	TTGCGCAGCA	-13	GGTATCCTTG	-139	1,1	8.79	-7.60	no	309	Rmet_3577	pckG	Op0994r_1	MET-CAH-TCA	
3161	TSS_514392+3	51	17	-36	TTGCCGTGAC	-14	ACTAGTCTTA	-142	1,1	6.70	11.40	s	55	Rmet_3955	0	Op1110f_1	MET-EN-OxPhos	
3162	TSS_592095+3	51	10	-40	GCAGGACCAA	-14	GTTATATTGG	-145	4,1	8.69	-0.60	no	25	Rmet_4022	0	Op1130f_1	NA	
3163	TSS_1563641+3	51	7	-29	TTGACCAAAA	0	CTCACCTTGA	-149	1,1	4.63	-1.80	no	54	Rmet_4900	0	Op1434f_2	NA	

3164	TSS_1883811+3	51	6	-35	TGGCCCCGGG	-15	ATTATCATCC	-136	1,1	7.61	10.10	s	6010	Rmet_5195	*0*	Op1532f_1	NA	
3165	TSS_2212975+3	51	12	-45	CCGGCATGGG	-16	TTTAGAATTA	-142	5,1	10.03	-6.10	*no*	2240	Rmet_6729	*0*	Op2094f_1	NA	
3166	TSS_19150-3	51	2	-38	GTAACGCCCG	-13	CCCATAATGG	-140	4,1	6.58	4.90	m	1157	Rmet_5829	*pstS1*	Op1763r_1	EIP-TRA-ABC	
3167	TSS_877341-3	51	10	-34	TGCGAGGCGT	-14	GGTATTGTGA	-145	4,1	5.61	5.60	m	73	Rmet_4285	*0*	Op1217r_1	MET-CAH-Pyr	
3168	TSS_1445021-3	51	17	-36	TCGACCATCA	-16	GTGATAGTCA	-142	1,1	5.16	10.30	s	5602	Rmet_4794	*slyA*	Op1395r_1	NA	
3169	TSS_2326855-3	51	9	-34	TTGCGCCAAG	-12	TATACGATGG	-139	1,1	8.35	10.20	s	127	Rmet_5572	*0*	Op1657r_2	NA	
3170	TSS_415466+2	51	14	-37	TGGTCAGATC	-15	CGTACTATTT	-141	1,1	6.78	10.90	s	646	Rmet_0388	*0*	Op0105f_2	NA	
3171	TSS_825536+2	51	5	-39	TCGCACGCTC	-14	ATTAACATGG	-144	1,1	6.52	3.90	m	301	Rmet_0749	*rimM*	Op0213f_1	GIP-TL-Ass	
3172	TSS_2031317+2	51	12	-45	TCGACGGCCA	-14	GGCACGATAC	-143	1,1	5.68	-7.10	*no*	70	Rmet_1872	*0*	Op0541f_2	NA	
3173	TSS_3383726+2	51	10	-49	TTGTTTCGGG	-23	GAAATGATAC	-146	1,1	4.52	-5.60	*no*	19823	Rmet_3139	*0*	Op0879f_1	NA	
3174	TSS_3474472+2	51	9	-36	CCTGCAACCT	-15	GGTATAATGA	-137	5,1	12.79	8.80	s	1412	Rmet_3201	*prfA*	Op0901f_1	NA	
3175	TSS_2465579-2	51	9	-37	TCGCAATGAT	-13	GCGATCATCT	-141	1,1	6.74	7.90	s	783	Rmet_2249	*dat*	Op0644r_1	MET-AA-Lys_Deg	
3176	TSS_3236882-2	51	7	-29	TTGCTTTTCG	-4	CTTACACTCG	-144	2,1	11.54	2.20	*(w)-nu*	88	Rmet_2977	*phcA*	Op0838r_2	NA	
3177	TSS_1661092+3	51	9	-37	TGGCCGGCGA	-15	GATAGTATTC	-138	1,1	7.22	9.90	s	529	Rmet_4986	*0*	Op1464f_1	NA	
3178	TSS_85530-3	51	9	-35	TTGACGCCAG	-13	ACTACGCTCC	-138	1,1	7.26	12.20	s	34	Rmet_6611	*0*	Op1783r_1	NA	
3179	TSS_856527-3	51	19	-41	ACAAAACAGT	-13	GATATATTGC	-148	4,1	3.76	-2.60	*no*	63	Rmet_4264	*0*	Op1211r_1	NA	
3180	TSS_943899-3	51	14	-33	CAACTCGTCG	-13	GTAGACTGGT	-143	3,0	6.20	-4.40	no	17	Rmet_4346	*0*	Op1237r_2	NA	
3181	TSS_1483068-3	51	25	-33	TTCAACAATC	-12	CTTATAGTGA	-141	1,1	7.22	9.10	s	225	Rmet_4828	*0*	Op1407r_1	NA	
3182	TSS_1969669-3	51	25	-47	TTGTCATGAA	-25	GATAGATTTC	-144	1,1	8.24	2.40	*w*	7	Rmet_5270	*0*	Op1553r_1	NA	
3183	TSS_1183743+2	50	2	-39	TTCCAAGCCC	-14	GCGATAATGT	-140	1,1	8.14	5.90	m	3990	Rmet_1092	*yflS*	Op0315f_1	EIP-TRA-Ion	
3184	TSS_2145196+2	50	7	-36	TTGAAGTCCG	-12	TCTGCACTCG	-142	1,1	2.58	8.40	s	76	Rmet_1969	*bcp*	Op0575f_2	GIP-PTL-Fold	
3185	TSS_3246454+2	50	13	-45	TACACGCGGC	-15	GATATCCTTC	-144	1,1	3.45	-6.60	*no*	7222	Rmet_2996	*ptxR*	Op0843f_1	NA	
3186	TSS_3507700-2	50	13	-46	TTGAAGGCAC	-13	GCGAGAATCA	-141	1,1	7.84	-8.60	*no*	501	Rmet_3230	*petA*	Op0914r_1	MET-EN-OxPhos	
3187	TSS_1180648-3	50	7	-37	TCTAACTGAC	-14	CCTATGCTCC	-144	5,1	4.95	7.40	s	40	Rmet_4567	*0*	Op1315r_3	NA	
3188	TSS_75311-4	50	12	-34	TGGCTTTTTC	-13	TTTACCTTCC	-143	1,1	4.82	7.10	s	1351	Rmet_5985	*czcM*	Op1822r_1	EIP-TRA-Other	
3189	TSS_6108+5	50	7	-34	GCTGCCGGCT	-14	CCTACGATAT	-138	5,1	9.41	3.60	m	1484	Rmet_6321	*tolA*	Op1920f_1	NA	
3190	TSS_31685-5	50	13	-41	TTGTCCTTTC	-14	CGTAACCTTA	-143	1,1	6.57	1.40	*(w)-sba*	1060	Rmet_6342	*stbD*	Op1927r_1	NA	
3191	TSS_91095+2	50	11	-34	TGGCACTCAG	-14	TCTATCGTAG	-141	1,1	5.29	7.10	s	65	Rmet_0084	*0*	Op0023f_1	MET-XEN-Benz	
3192	TSS_1931291+2	50	15	-49	GCGGCCGCCC	-14	CGTATAATTA	-144	5,1	11.64	-16.10	*no*	36	Rmet_1779	*0*	Op0517f_1	NA	
3193	TSS_2703512+2	50	10	-35	TCGGCAGCCT	-1	CACACAATGT	-139	5,1	9.31	-0.30	*no*	552	Rmet_2491	*0*	Op0689f_2	NA	
3194	TSS_3503169+2	50	8	-48	TTGTTCTCCC	-15	GGGATACTAC	-138	1,1	6.45	-11.60	*no*	4249	Rmet_3231	*mscL*	Op0915f_1	EIP-TRA-Pores	
3195	TSS_3712415+2	50	12	-40	TGGTGCGCAT	-14	ACTACCATTC	-142	1,1	6.23	1.90	*(w)-sba*	718	Rmet_3437	*pat*	Op0955f_1	NA	
3196	TSS_567376-2	50	7	-36	TTGCCGATCG	-14	CGTACAATAG	-138	1,1	9.95	13.40	s	45	Rmet_0531	*0*	Op0148r_1	NA	
3197	TSS_1136995-2	50	11	-26	TGGACGCTCG	-4	TCTACATTAT	-143	1,1	6.42	2.70	*(w)-nu*	233	Rmet_1044	*yebK*	Op0300r_1	NA	
3198	TSS_1300873-2	50	4	-34	TTGACAAAGC	-14	GCTAGAATCT	-139	1,1	11.47	12.60	s	6	Rmet_R0018	*0*	Op2005r_1	NA	
3199	TSS_1509074-2	50	10	-43	TTGCCTGTGT	-12	GATATAAAGC	-142	1,1	6.51	-3.60	*no*	194	Rmet_1399	*0*	Op0392r_1	NA	
3200	TSS_2147270-2	50	3	-37	TGGCGCGCGT	-13	TGTAGAGTTT	-140	1,1	6.43	6.90	s	2754	Rmet_1967	*aatA*	Op0574r_1	NA	
3201	TSS_3276923-2	50	9	-40	TCCAAAGCGT	-14	GCCAAAATTA	-147	4,1	8.69	2.40	*(w)-sba*	113	Rmet_3017	*0*	Op0848r_4	NA	
3202	TSS_3607488-2	50	12	-47	TCGCATTACC	-25	AATCTAGTAG	-148	1,1	0.90	-2.10	*no*	806	Rmet_3334	*rpoB*	Op0930r_4	GIP-TK-RNAP	
3203	TSS_3863510-2	50	14	-35	TGGTGCCCAG	-14	GATACGATTG	-140	1,1	6.57	9.10	s	133	Rmet_3561	*0*	Op0988r_2	NA	
3204	TSS_566416+3	50	12	-44	CTGCGCCGCA	-15	CATACACTGG	-147	1,1	3.65	-6.60	*no*	37	Rmet_3996	*mdoG*	Op1122f_1		0
3205	TSS_946021+3	50	7	-36	TGGACGTTCA	-15	GTGATCATCA	-141	1,1	6.05	11.30	s	876	Rmet_6661	*0*	Op1240f_1	NA	
3206	TSS_1496109+3	50	10	-36	TCGCCCGGTC	-16	CCGAGAATTC	-140	1,1	6.08	9.30	s	137	Rmet_4839	*0*	Op1412f_1	NA	
3207	TSS_20390+5	50	3	-38	TTGCTGCGTG	-14	TGTACCCTGA	-140	1,1	6.74	6.40	s	1222	Rmet_6333	*tnpA*	Op1926f_1	NA	
3208	TSS_83644-2	50	18	-41	TTGAGCACCT	-11	GATACGATGG	-137	1,1	9.38	-0.60	*no*	20	Rmet_0076	*hldC*	Op0022r_3	NA	
3209	TSS_1979530-2	50	16	-47	TTGTCGTAAT	-13	CGCAATATCG	-143	1,1	6.24	-11.60	*no*	281	Rmet_1822	*0*	Op0528r_1	NA	
3210	TSS_83430+3	50	9	-35	TGGCGCCCAC	-14	CGTACGCTGT	-140	1,1	5.95	8.10	s	2338	Rmet_5888	*0*	Op1784f_1	NA	
3211	TSS_1168566+3	50	1	-35	TTGCGGTTTC	-15	GTCATACTCT	-137	1,1	8.38	9.60	s	55	Rmet_4558	*0*	Op1308f_1	NA	
3212	TSS_1325641+3	50	9	-36	TTGCCTAAGT	-14	CAAATAATAG	-141	1,1	7.20	13.40	s	36	Rmet_4690	*0*	Op1356f_2	NA	
3213	TSS_2538868+3	50	15	-35	TGGAGACCAT	-14	GATAAAGTGG	-140	1,1	7.62	11.10	s	486	Rmet_5780	*0*	Op1738f_1	NA	
3214	TSS_1273482-3	50	6	-47	CTCCGTTTGG	-13	GCCATAATCG	-144	1,1	3.58	-15.10	*no*	66	Rmet_4642	*0*	Op1341r_2	NA	
3215	TSS_1296324-3	50	13	-39	GCAGCAGTAC	-14	GGTATTCTGT	-141	5,1	9.46	1.40	w	995	Rmet_4661	*rpoD2*	Op1349r_1	GIP-TK-RNAP	
3216	TSS_1569167-3	50	10	-34	TTGATTGTAA	-13	CGTAATATGA	-144	1,1	7.40	10.60	s	9	Rmet_4905	*0*	Op1437r_1	NA	
3217	TSS_2274874-3	50	9	-34	TGCCCAAAGA	-13	GATACGATGT	-142	1,1	7.44	8.60	s	785	Rmet_5527	*0*	Op1643r_2	NA	
3218	TSS_2107248+2	49	1	-35	TTGATGACGG	-14	ACTAAGCTGG	-139	1,1	7.04	10.60	s	66	Rmet_1938	*0*	Op0565f_2	NA	
3219	TSS_2389700+2	49	7	-38	TCGATCAACC	-14	TGTAGTCTTC	-144	1,1	5.37	5.90	m	9344	Rmet_2190	*0*	Op0629f_1	NA	
3220	TSS_3639226+2	49	13	-39	TGGCAGCATG	-13	TATAGAGTGT	-142	1,1	6.00	2.90	w	6571	Rmet_3369	*paaA*	Op0937f_1	NA	
3221	TSS_208454-2	49	5	-41	TGCACGCAGT	-13	GCTATAATTT	-142	1,1	9.53	1.40	*(w)-sba*	56	Rmet_0199	*0*	Op0058r_1	NA	
3222	TSS_2836443-2	49	10	-47	TTGAGCACGT	-24	GGAAAAATTG	-140	1,1	8.37	0.40	*w*	122	Rmet_2606	*rpoD1*	Op0730r_3	GIP-TK-RNAP	
3223	TSS_150159+3	49	11	-28	TTGTGTTCAC	-8	TTCACACTTA	-144	1,1	5.85	1.60	*(w)-nu*	26	Rmet_R0071	*tRNA-I*	Op1006f_1	NA	
3224	TSS_225159+3	49	7	-47	TTGATGTAAA	-13	TACAAAATTC	-145	1,1	8.17	-10.60	*no*	267	Rmet_3685	*flhD2*	Op1026f_3	EIP-SIG-2Comp	

3225	TSS_1761551+3	49	4	-36	TCTGCAGCTT	-14	TTCATACTGA	-145	5,1	7.90	10.40	s	3327	Rmet_5076		0	Op1494f_1	NA
3226	TSS_69271+4	49	13	-35	TTGCGTGTGC	-8	TGTAGTGTAT	-144	1,1	5.07	5.20	(m)-ba	3157	Rmet_6365		0	Op2109f_1	NA
3227	TSS_214290+2	49	17	-42	TACGGTACGG	-17	CGTATATTCT	-146	2,1	5.96	0.40	(w)-sba	95	Rmet_0205	aptA		Op0061f_1	MET-CAH-Prop
3228	TSS_3152393+2	49	13	-41	GCACGCCAAA	-16	TGCATACTTC	-145	4,1	6.55	-2.60	no	750	Rmet_2897		0	Op0815f_2	NA
3229	TSS_3298519+2	49	12	-37	TCGCGAGCGT	-14	GGTAAAAAAC	-143	1,1	4.08	7.90	s	5780	Rmet_3047	thiL		Op0857f_1	MET-COF-Thia
3230	TSS_3631609+2	49	12	-48	TTGCGACATC	-28	TTCAGGCTGC	-141	1,1	5.66	-3.20	no	376	Rmet_3356		0	Op0935f_2	NA
3231	TSS_3881775+2	49	21	-46	CCAGCACGTC	-14	GTCATGATGC	-138	5,1	8.80	-12.60	no	914	Rmet_3578	gyaR		Op0995f_1	MET-AA-GlySerThr
3232	TSS_1210636-2	49	11	-37	TGCAGCGCGG	-13	GGCATACTGC	-137	1,1	6.19	6.40	s	478	Rmet_1110	lysA		Op0320r_2	MET-AA-Lys_Syn
3233	TSS_2395400-2	49	11	-34	TGGATTTGGC	-14	ATCATAATCT	-141	1,1	7.02	9.10	s	268	Rmet_2186	glmM		Op0628r_3	MET-CAH-AminSug
3234	TSS_3632701-2	49	12	-35	TTGCCGCTAT	-12	GCGAATATGG	-142	1,1	7.29	10.20	s	2167	Rmet_3353	tnpA		Op0934r_1	NA
3235	TSS_219388+3	49	34	-48	TCGCGAATCG	-26	GGTTCCATCG	-144	1,1	1.75	-3.10	no	100	Rmet_3680		0	Op1026f_2	EIP-SIG-2Comp
3236	TSS_554000+3	49	3	-27	TGGCGGGGGC	-5	CAAAAACTGT	-145	1,1	4.20	0.70	(w)-nu	3518	Rmet_3988		0	Op1118f_1	NA
3237	TSS_1883430+3	49	6	-35	TTGAGCCGTA	-14	GGTATCGTCT	-140	1,1	7.36	11.60	s	61	Rmet_5189		0	Op1530f_2	NA
3238	TSS_2062753-3	49	7	-46	TCGAATTTAA	-14	CATATGCTGG	-143	1,1	6.38	-9.10	no	123	Rmet_5341		0	Op1583r_1	NA
3239	TSS_791316+2	49	18	-32	CCGGACGTAT	-12	GTCATAATGC	-140	5,1	10.57	3.10	w	720	Rmet_0715	serC		Op0203f_2	MET-AA-GlySerThr
3240	TSS_905423+2	49	2	-34	TTGAGCGATT	-14	GCCATACTTC	-139	1,1	8.90	9.60	s	540	Rmet_0831	alaS		Op0237f_1	MET-AA-AlaAsp
3241	TSS_3299386+2	49	9	-43	TTCTCCTCGG	-14	GGCATAGTTG	-141	1,1	5.84	-3.10	no	4913	Rmet_3047	thiL		Op0857f_1	MET-COF-Thia
3242	TSS_3639685+2	49	6	-35	TTGAAACGAT	-8	TCCACAATGT	-141	1,1	9.43	8.20	(s)-ba	6112	Rmet_3369	paaA		Op0937f_1	NA
3243	TSS_905772-2	49	4	-40	GCAGGGACAT	-13	GATAGAGTGC	-139	4,1	8.96	-3.60	no	28	Rmet_0830		0	Op0236r_1	NA
3244	TSS_1581416-2	49	8	-42	TTGCTGATGC	-15	GCTAATCTCG	-143	1,1	7.76	-0.60	no	36	Rmet_1460	ytfF		Op0408r_1	NA
3245	TSS_3023678-2	49	4	-41	GCGTCATGGG	-12	GCTAGAATCG	-140	5,1	9.06	-2.10	no	100	Rmet_2775		0	Op0780r_1	NA
3246	TSS_3430864-2	49	6	-35	TTGCCAACAG	-5	TTAACAATAT	-145	1,1	6.98	5.20	(m)-ba	181	Rmet_R0051		0	Op0886r_1	NA
3247	TSS_3557807-2	49	12	-46	CCATCAGCGT	-14	GCTATAGTAC	-143	5,1	7.64	-11.60	no	94	Rmet_3282		0	Op0924r_1	NA
3248	TSS_266968-3	49	12	-40	TTGCGCGTAT	-13	GAGATAGTCC	-140	1,1	6.78	2.40	(w)-sba	1727	Rmet_3723		0	Op1037r_1	DIV-MOT-Chemotax
3249	TSS_445185-3	49	11	-49	TTCCGCAAAT	-13	GGAATAATGC	-140	1,1	7.90	-16.10	no	23	Rmet_3899		0	Op1087r_1	MET-CAH-Prop
3250	TSS_142918+5	49	9	-39	TTGACTCGCG	-14	GCTACCATAT	-141	1,1	8.49	7.40	s	14839	Rmet_6302	trbB		Op1918f_1	NA
3251	TSS_327604+2	48	6	-34	GCACCAACAT	-14	GGTATCCTGC	-139	4,1	10.18	4.60	m	4788	Rmet_0314		0	Op0091f_1	NA
3252	TSS_427229+2	48	4	-28	TGGTCAAGGA	-3	GCCAAGCTGG	-145	1,1	5.36	-0.30	no	952	Rmet_0400	gltJ		Op0109f_4	NA
3253	TSS_1289895+2	48	17	-47	TTGCGGTTGC	-12	GTTAAGACAG	-145	1,1	0.77	-14.60	no	6131	Rmet_6440		0	Op2004f_1	NA
3254	TSS_1471455+2	48	13	-33	TGGCGTAAGC	-13	CATACAATTA	-141	1,1	8.22	7.10	s	73	Rmet_1362	bktB		Op0383f_4	MET-CAH-Pyr
3255	TSS_3004563+2	48	13	-20	AAAATCGGTA	0	AAACCCCGAC	-147	3,0	7.12	-16.40	no	5369	Rmet_2768	ompA		Op0777f_1	NA
3256	TSS_3038613+2	48	7	-34	TGGTATGACT	-14	TGTATAGTGT	-139	1,1	6.76	8.10	s	436	Rmet_2792		0	Op0785f_1	NA
3257	TSS_1343456-2	48	10	-49	TCGCCGACGA	-13	ACTATTATGC	-144	1,1	7.59	-15.10	no	102	Rmet_1223	bzdR		Op0354r_1	MET-AA-PheTyrTrp
3258	TSS_2298814-2	48	8	-46	TTGAAGCGGT	-24	CTTAAAATCG	-139	1,1	10.31	3.40	m	5	Rmet_2116		0	Op0612r_2	NA
3259	TSS_2600094-2	48	12	-33	TTCCTTCACT	-13	GGCAAGATGC	-141	1,1	5.97	5.10	m	2660	Rmet_2384	armR		Op0672r_1	NA
3260	TSS_833805+3	48	13	-44	TCATACCCAG	-13	AATAGAATAG	-146	5,1	6.03	-7.60	no	39	Rmet_4247		0	Op1202f_4	NA
3261	TSS_1530672+3	48	20	-36	TGGCAAGGTC	-15	GATTCACTCC	-147	1,1	2.52	8.30	s	193	Rmet_4869		0	Op1420f_1	NA
3262	TSS_1664443+3	48	12	-42	TTGATCGTTC	-14	CATACAATGG	-136	1,1	9.60	1.40	(w)-sba	61	Rmet_4988		0	Op1464f_2	EIP-TRA-ABC
3263	TSS_2048095+3	48	10	-35	TTGCATTCGA	-15	CCTACCATTG	-137	1,1	8.44	9.60	s	26	Rmet_5336		0	Op1582f_1	NA
3264	TSS_210171-3	48	7	-33	TTGCATTCAT	-7	ATTACGATAC	-145	1,1	8.06	5.20	(m)-ba	128	Rmet_3672		0	Op1021r_1	NA
3265	TSS_1279574-3	48	12	-44	GCTGCACGCG	-13	CGGATAATGC	-138	5,1	10.24	-8.60	no	200	Rmet_4648		0	Op1343r_2	NA
3266	TSS_12554+5	48	14	-36	TTGCTAGATT	-15	GATACTTTGA	-143	1,1	6.53	10.80	s	3976	Rmet_6327		0	Op1922f_1	NA
3267	TSS_2189938+2	48	7	-32	TTGCCATGAT	-5	GCGATATTAC	-140	1,1	7.85	5.20	(m)-ba	140	Rmet_2025		0	Op0593f_1	NA
3268	TSS_2395663+2	48	15	-44	TCAACCGCAC	-15	GATATCATTG	-141	4,1	8.89	-3.60	no	3381	Rmet_2190		0	Op0629f_1	NA
3269	TSS_3550878+2	48	12	-37	TCGCTTTCAC	-14	GCTACACTCC	-137	1,1	7.76	7.90	s	117	Rmet_6588		0	Op0923f_1	NA
3270	TSS_1556261-2	48	1	-47	TGGCCAAAGT	-26	TCCAGCATTT	-141	1,1	6.19	-0.70	no	349	Rmet_1434	map		Op0402r_1	GIP-TL-Ass
3271	TSS_2869127-2	48	10	-47	TCGAGGTCCT	-14	AGCATAATCC	-141	1,1	7.42	-10.10	no	44	Rmet_6544		0	Op0738r_1	NA
3272	TSS_2888684-2	48	17	-35	TTGCGGAACC	-13	GCTAGTATAG	-142	1,1	8.17	11.20	s	83	Rmet_2652		0	Op0750r_1	NA
3273	TSS_2998870-2	48	4	-41	TTGCGGCAGT	-14	CGTACAATCG	-137	1,1	10.30	2.40	(w)-sba	28	Rmet_2757	sodC		Op0776r_2	OTHERS
3274	TSS_1493642+2	48	27	-37	TTGTTGCAAC	-15	TCTACACTCT	-145	1,1	7.71	10.40	s	113	Rmet_1385	aceA		Op0385f_4	MET-CAH-DiCarb
3275	TSS_2664488+2	48	3	-36	TTGCATAAAT	-16	GCTATAATGC	-138	1,1	12.25	11.80	s	157	Rmet_R0041		0	Op0685f_2	NA
3276	TSS_3546317+2	48	11	-33	TTGACCCTGC	-12	TCCATGCTGG	-140	1,1	7.01	9.60	s	1373	Rmet_6587		0	Op2052f_1	NA
3277	TSS_3662318+2	48	9	-35	TTGACGCGAG	-9	GGTATGCTGC	-139	1,1	9.48	9.20	(s)-ba	121	Rmet_3389	gspL		Op0941f_2	GIP-PTL-T2S
3278	TSS_1797369-2	48	15	-43	TCGACACCGC	-13	CGTAGGATGC	-145	1,1	7.48	-2.10	no	317	Rmet_1662		0	Op0468r_3	NA
3279	TSS_3035408-2	48	8	-36	TGGCGTTTCG	-13	GGTATCCTCG	-138	1,1	7.18	8.90	s	209	Rmet_2788	btuC		Op0784r_1	EIP-TRA-ABC
3280	TSS_2352904+3	48	11	-49	TCGAAAAGTC	-15	AATACGATAT	-143	1,1	6.72	-13.10	no	152	Rmet_5599	katA		Op1668f_1	MET-EN-Methane
3281	TSS_212015-3	48	17	-36	TTGCACATGG	-14	GATACCTTGG	-140	1,1	7.43	10.40	s	53	Rmet_3674		0	Op1023r_1	MET-EN-Nitrog
3282	TSS_333685-3	48	2	-47	GCAGGCGTAC	-13	ACTAAGATAC	-140	4,1	7.93	-17.60	no	51	Rmet_3794		0	Op1053r_2	NA
3283	TSS_2310261-3	48	12	-37	GCGGCCGCCG	-13	CTTATGATTG	-138	5,1	10.15	5.90	m	1536	Rmet_6746		0	Op2099r_1	NA
3284	TSS_155279+2	47	15	-42	TCGCTCGGGA	-11	CCTATCGTCA	-142	1,1	4.18	-5.10	no	61	Rmet_0151		0	Op0043f_1	NA
3285	TSS_496737+2	47	6	-42	CCTGACGTCG	-14	CGCATACTTG	-139	5,1	8.04	-6.60	no	792	Rmet_0468		0	Op0123f_2	NA

3286	TSS_651968+2	47	10	-42	TTGCCGTTCA	-15	GTCATACTGA	-140	1,1	8.50	1.40	(w)-sba	9	Rmet_0603	0	Op0171f_1	NA
3287	TSS_1257940+2	47	6	-36	TTGAACGACC	-14	GGTAAGCTGC	-141	1,1	8.81	11.40	s	67	Rmet_1147	0	Op0329f_1	NA
3288	TSS_2504597+2	47	6	-45	TTGAACAGCT	-15	ACCACAATAC	-140	1,1	8.36	-4.60	no	6	Rmet_R0037	0	Op2032f_1	NA
3289	TSS_2641294+2	47	17	-35	TCACAAACGA	-15	CGTACCCTTG	-144	4,1	6.81	6.60	s	1820	Rmet_6526	0	Op0681f_1	NA
3290	TSS_3135074+2	47	19	-42	TCGATAGAGA	-14	AGTAGCCTAG	-144	1,1	4.71	-1.10	no	606	Rmet_2883	0	Op0811f_1	GIP-REP-Recomb
3291	TSS_611244-2	47	8	-36	TTGACGGTGC	-14	CTTATATTGC	-140	1,1	9.21	14.40	s	33	Rmet_0571	modA	Op0160r_1	EIP-TRA-ABC
3292	TSS_2597479-2	47	4	-28	TTGCGTCCTA	0	CCTAACATGA	-144	1,1	7.26	-1.80	no	45	Rmet_2384	armR	Op0672r_1	NA
3293	TSS_3328656-2	47	9	-27	CCGGACGCGG	-5	CACCGAATTC	-143	5,1	2.95	-3.30	no	139	Rmet_3071	0	Op0862r_3	NA
3294	TSS_152401+3	47	6	-36	TTGCAATAAA	-12	TGTTTAATAA	-145	1,1	4.55	10.40	s	1227	Rmet_R0074	0	Op1006f_1	NA
3295	TSS_687485+3	47	20	-46	TGCAACGCAT	-14	GGCAGAATTC	-141	1,1	6.71	-10.60	no	1804	Rmet_4119	hmyF	Op1160f_1	NA
3296	TSS_1869281+3	47	6	-41	TCGAGGTTAC	-18	GCGATCATAC	-144	1,1	5.89	4.90	(m)-sba	746	Rmet_6712	0	Op1522f_1	NA
3297	TSS_2235433-3	47	4	-34	GCGGCTCGAG	-13	GGTAAACTGG	-138	5,1	10.31	6.10	m	2912	Rmet_5488	0	Op1629r_1	GIP-REP-Recomb
3298	TSS_228782+4	47	12	-42	TTCACCTCCA	-13	AATAATATGC	-143	1,1	6.77	-1.10	no	184	Rmet_6071	0	Op1849f_1	NA
3299	TSS_969335+2	47	4	-37	TTGACAATTA	-15	AGCACCATTG	-143	1,1	7.53	12.40	s	193	Rmet_0885	0	Op0257f_1	NA
3300	TSS_1626298+2	47	5	-43	TCAAAACCAA	-14	GGCAAAGTCG	-145	4,1	5.77	-4.60	no	4682	Rmet_1509	cbbS(del	Op0425f_1	NA
3301	TSS_2556369+2	47	13	-37	TTGACCATTC	-15	GATAACTTCC	-139	1,1	7.39	11.40	s	3034	Rmet_2345	0	Op0663f_1	NA
3302	TSS_192967-2	47	7	-37	TGGCGCAGAT	-13	ACGATAATGC	-138	1,1	7.60	7.90	s	509	Rmet_0185	0	Op0056r_1	NA
3303	TSS_238782-2	47	3	-46	TGCCGGAGGC	-14	GATACGATCC	-139	1,1	6.86	-11.60	no	35	Rmet_0224	0	Op0066r_1	NA
3304	TSS_530850-2	47	20	-38	TTGCACGCTC	-14	AGTAATGTAC	-142	1,1	5.40	6.40	s	171	Rmet_0500	0	Op0134r_1	NA
3305	TSS_1764815-2	47	2	-36	TTGACGCGCG	-15	TCTATCGTGC	-141	1,1	7.33	12.80	s	174	Rmet_1635	bug	Op0458r_1	NA
3306	TSS_2336693-2	47	9	-27	TCGCGATTTT	0	CGTACGATTC	-142	1,1	7.94	-2.30	no	786	Rmet_2143	yejF	Op0618r_1	NA
3307	TSS_3448056-2	47	10	-20	GGCGGCGTGT	0	CGAAGTCTGG	-145	4,1	2.13	-13.40	no	256	Rmet_3173	mltA	Op0890r_1	NA
3308	TSS_213261+3	47	7	-35	TCGCAGCCGT	-14	GTTATGATGG	-138	1,1	8.60	10.10	s	50	Rmet_3676	0	Op1024f_2	MET-CAH-Gal
3309	TSS_476149+3	47	10	-43	TGCAATCACG	-16	GCTAACGTCC	-145	1,1	4.18	-3.60	no	2029	Rmet_R0079	0	Op1098f_1	NA
3310	TSS_629552+3	47	7	-33	TTGCGGATGA	-13	GATAAAGTTC	-142	1,1	8.68	7.60	s	1389	Rmet_4066	0	Op1142f_1	NA
3311	TSS_1112096-3	47	27	-35	TTCAAGTAGA	-13	CCGATAATGG	-140	1,1	7.73	11.70	s	354	Rmet_4508	0	Op1287r_1	NA
3312	TSS_117004-4	47	10	-31	TTGTAGACGA	-4	AGCAGAATCA	-140	1,1	6.66	2.20	(w)-ba	2508	Rmet_5947	pbrA	Op1808r_1	MET-EN-P_ATPase
3313	TSS_5917+5	47	8	-36	TGGACAACCG	-14	GGCAGACTAC	-139	1,1	6.52	11.90	s	1675	Rmet_6321	tolA	Op1920f_1	NA
3314	TSS_23665+2	47	13	-43	GCTGAACGGG	-15	GCTATGCTCG	-136	5,1	9.52	-6.60	no	26	Rmet_0020	0	Op0003f_4	MET-AA-Tyr
3315	TSS_302642+2	47	1	-48	TGGCCAGTCG	-14	AATATCATGG	-140	1,1	7.74	-11.10	no	6874	Rmet_0293	mutM	Op0085f_1	NA
3316	TSS_1323250+2	47	12	-49	TTGCCAACCA	-15	ATTAATCTTG	-147	1,1	7.14	-12.60	no	51	Rmet_1204	0	Op0349f_2	NA
3317	TSS_2325428+2	47	1	-47	TTGTAGAACC	-15	CCTACTCTTT	-143	1,1	6.42	-10.60	no	223	Rmet_2137	0	Op0615f_2	GIP-TL-Ribo
3318	TSS_3614147-2	47	8	-35	TTGCAATAAA	-11	TGTTTAATAA	-145	1,1	4.55	10.20	s	1228	Rmet_R0060	0	Op0930r_1	NA
3319	TSS_3671976-2	47	8	-42	GCCACGATGT	-13	CGTATCCTGT	-139	4,1	7.39	-5.60	no	28	Rmet_3395	0	Op0942r_1	NA
3320	TSS_272475+3	47	3	-45	TTCCAGCGTG	-14	GACATGATCT	-140	1,1	6.18	-8.10	no	4904	Rmet_6620	0	Op1042f_1	NA
3321	TSS_474709+3	47	16	-28	TTGTGTTCAC	-8	TTCACACTTA	-144	1,1	5.85	1.60	(w)-nu	26	Rmet_R0076	0	Op1098f_1	NA
3322	TSS_1601005+3	47	17	-42	TTGACCCGTG	-15	TGTAATATTT	-145	1,1	8.58	2.40	(w)-sba	58	Rmet_4935	0	Op1448f_2	NA
3323	TSS_2535025+3	47	8	-49	TCGACACGGC	-14	GGTAAATTCG	-139	1,1	8.17	-13.10	no	35	Rmet_5775	0	Op1736f_2	NA
3324	TSS_2573468+3	47	4	-38	GTCAAGCACC	-14	GCTAGAGTAG	-142	4,1	6.63	3.90	m	920	Rmet_6770	0	Op1754f_1	NA
3325	TSS_1052531-3	47	2	-34	TGGCCTGCCT	-13	ACTAGACTGC	-140	1,1	7.21	9.10	s	114	Rmet_4455	0	Op1267r_1	NA
3326	TSS_2226927-3	47	6	-34	CCTGCACCCT	-14	GCTAAACTGG	-138	5,1	11.07	4.60	m	1710	Rmet_5481	tnmB	Op2095r_1	NA
3327	TSS_223419+4	47	2	-46	TTGCTCGGTG	-14	AGTAAAATGG	-137	1,1	9.60	-7.60	no	357	Rmet_6389	0	Op2116f_1	NA
3328	TSS_176183-4	47	8	-41	TAGCCCCGCT	-13	GTTACCATTG	-139	1,1	5.29	-0.10	no	5765	Rmet_6133	silD	Op1874r_1	NA
3329	TSS_56132+5	47	8	-35	GTAAAGCCCG	-14	GTTAAGATCC	-142	4,1	7.66	7.10	s	989	Rmet_6210	cnrA	Op1896f_2	EIP-TRA-Ion
3330	TSS_1165299+2	46	10	-31	TGGTCTATTT	-5	GGCACGTTCC	-145	1,1	4.04	0.70	(w)-ba	6356	Rmet_1079	0	Op0311f_1	GIP-TL-Ass
3331	TSS_2109582+2	46	1	-35	TCGCGCCCTA	-14	GCTAACATGC	-137	1,1	7.34	9.10	s	46	Rmet_1941	0	Op0567f_2	NA
3332	TSS_2124834+2	46	5	-38	GCCGGCAAAC	-14	TCTATAATAC	-137	4,1	10.78	3.40	m	148	Rmet_1955	mfd	Op0573f_1	GIP-REP-Recomb
3333	TSS_3322551+2	46	9	-44	TGGCACATGC	-15	CGTAGACTCC	-136	1,1	7.90	-5.10	no	297	Rmet_3066	prmA	Op0861f_4	GIP-TL-Ass
3334	TSS_1287281-2	46	13	-34	TTGACCAAGC	-14	GTTATTTTCC	-144	1,1	8.23	10.60	s	33	Rmet_1175	0	Op0338r_1	NA
3335	TSS_2388962-2	46	4	-43	TCGAGCGTAT	-12	TGCAGAATTC	-142	1,1	7.01	-5.10	no	108	Rmet_2180	phoB	Op0628r_4	EIP-SIG-2Comp
3336	TSS_3293646-2	46	3	-49	TGGATACGGG	-13	CGTACTATGG	-143	1,1	7.29	-15.10	no	1276	Rmet_3038	0	Op0854r_1	NA
3337	TSS_3507205-2	46	3	-34	TTGACGCAAG	-11	CATCCCCTGG	-149	1,1	2.19	8.20	s	6	Rmet_3230	petA	Op0914r_1	MET-EN-OxPhos
3338	TSS_3850962-2	46	14	-38	GCCACCACGA	-13	CGTAGAGTTC	-141	4,1	6.26	2.40	w	2699	Rmet_3548	0	Op0984r_1	NA
3339	TSS_2242804+3	46	7	-35	TTCATTATGG	-15	GATATCATGG	-142	1,1	7.78	10.10	s	24	Rmet_5499	0	Op1636f_1	MET-CAH-Pyr
3340	TSS_2579082+3	46	13	-30	CTGCCAACGG	-10	GACAATATGC	-144	1,1	3.06	2.60	(w)-sba	244	Rmet_5816	cspA	Op1756f_1	NA
3341	TSS_41829+5	46	9	-39	TTGCTTTCCT	-14	CGTAAGATAG	-148	1,1	7.48	5.40	m	21	Rmet_6192	bimB1	Op1894f_6	NA
3342	TSS_651891+2	46	5	-45	GCCACAGCGC	-15	TGTACAATCC	-136	4,1	11.29	-7.60	no	86	Rmet_0603	0	Op0171f_1	NA
3343	TSS_1092066+2	46	8	-44	CCTGAACCGC	-14	GGCATCATGG	-142	5,1	8.63	-9.60	no	1148	Rmet_1001	hemH	Op0285f_1	MET-COF-Porph
3344	TSS_1148043+2	46	11	-39	CGGAGATAGC	-15	TATATCATCG	-146	1,1	3.65	4.90	m	327	Rmet_1055	eno	Op0301f_3	MET-CAH-FbP
3345	TSS_1592569-2	46	3	-35	TTGCGGCAGA	-14	TCGAATATGC	-143	1,1	6.18	9.60	s	1186	Rmet_1471	0	Op0412r_1	NA
3346	TSS_2508166-2	46	7	-44	TGGCGCCCGC	-14	CGTACTATCC	-140	1,1	6.87	-6.10	no	113	Rmet_2285	0	Op0654r_2	EIP-TRA-Ion

3347	TSS_3433149-2	46	13	-35	TTGCAAAGCC	-14	CCTAATATTC	-139	1,1	8.97	11.60	s	429	Rmet_R0054	0	Op0886r_1	NA
3348	TSS_1152412+3	46	8	-33	GAACTCGTCG	-13	TGCCATAGAA	-141	3,0	8.07	-3.40	no	1721	Rmet_4548	0	Op1304f_1	NA
3349	TSS_1359281-3	46	2	-34	TATCTTTTCT	-13	CCTAATCTTG	-148	2,1	8.27	5.60	m	373	Rmet_4715	0	Op1361r_1	NA
3350	TSS_462584+2	46	16	-35	TACCGGTAAC	-15	CCGACAATAA	-146	1,1	1.55	6.60	s	670	Rmet_0437	surA	Op0117f_1	MET-COF-B6
3351	TSS_580517+2	46	13	-31	GCGGCCGTGG	-1	CACATGATGC	-140	5,1	8.15	-4.30	no	3448	Rmet_0548	0	Op0153f_1	NA
3352	TSS_941766+2	46	6	-41	TTGCCTTGCC	-18	GCCATCATGA	-140	1,1	8.04	6.40	(s)-sba	265	Rmet_R0010	0	Op0247f_3	NA
3353	TSS_1163933+2	46	11	-36	TATATCCGCG	-13	TGTACAGTCA	-142	2,1	8.54	8.40	s	1219	Rmet_1073	slyD	Op0309f_1	GIP-PTL-Fold
3354	TSS_1334832+2	46	7	-38	GCATACGTGG	-14	TGCACAATCG	-143	5,1	6.28	1.40	w	8770	Rmet_1224	bzdA	Op0355f_1	MET-XEN-Benz
3355	TSS_2158544+2	46	15	-42	TTCTGACTAA	-14	AGTAGGCTAT	-147	1,1	4.17	-2.10	no	1645	Rmet_6495	0	Op0579f_1	NA
3356	TSS_2264600+2	46	8	-49	TTGATCTAGC	-15	GGCACAATTT	-138	1,1	9.42	-12.60	no	68	Rmet_2086	narX	Op0603f_1	EIP-SIG-2Comp
3357	TSS_3365328+2	46	10	-35	TTGCCGCCGG	-13	CGTACATTGT	-142	1,1	8.12	12.20	s	454	Rmet_3107	ispB	Op0875f_1	NA
3358	TSS_371377-2	46	3	-33	TTGAATTAAT	-13	ATTAATATCT	-145	1,1	8.19	8.60	s	21	Rmet_0351	0	Op0098r_1	NA
3359	TSS_1309459-2	46	2	-33	TGGACGGCAT	-13	TTTAAAATCA	-140	1,1	8.74	9.10	s	168	Rmet_1194	0	Op0346r_2	NA
3360	TSS_1312186-2	46	9	-34	TTCAGATTAC	-14	CTTATAATGT	-143	1,1	9.95	11.10	s	145	Rmet_1195	0	Op0346r_1	NA
3361	TSS_1460980-2	46	6	-39	TGGAACGCAA	-9	TCAAATATGG	-143	1,1	4.16	-1.10	no	12698	Rmet_1338	0	Op0378r_1	NA
3362	TSS_2509707-2	46	2	-40	TGGCGCAAAG	-13	GGTATGGTCT	-142	1,1	6.47	0.90	(w)-sba	214	Rmet_2287	0	Op0654r_1	NA
3363	TSS_2771429-2	46	13	-34	TTGAGGATCA	-13	GGTACATTGC	-136	1,1	9.41	10.60	s	28	Rmet_2546	0	Op0706r_1	NA
3364	TSS_3613024-2	46	9	-33	TTGCCCGTAA	-9	ACCAGTGTGT	-147	1,1	3.77	6.20	(m)-ba	105	Rmet_R0060	0	Op0930r_1	NA
3365	TSS_355133+3	46	11	-44	GGACCGATGG	-15	CGTATGATGA	-143	4,1	7.62	-7.60	no	3197	Rmet_3817	acd	Op1062f_1	NA
3366	TSS_418322+3	46	4	-42	TTCCGTATCA	-15	TATAAGCTTC	-146	1,1	5.68	-2.10	no	961	Rmet_3876	0	Op1080f_1	NA
3367	TSS_1028837+3	46	14	-40	TGGACGACTT	-15	GCTAGAATTC	-140	1,1	9.99	5.90	(m)-sba	129	Rmet_4433	bug	Op1262f_1	NA
3368	TSS_1146383+3	46	21	-39	TCCAGTTCAT	-15	TGTATGATCC	-141	1,1	6.64	5.40	m	460	Rmet_4540	0	Op1300f_1	NA
3369	TSS_1832862+3	46	11	-47	TTGCAACGTG	-15	TGTAAGATGA	-140	1,1	8.91	-8.60	no	50	Rmet_5135	0	Op1514f_1	NA
3370	TSS_2321450+3	46	16	-42	TCGGACGGTG	-12	AATAAAATGA	-143	5,1	9.46	-3.10	no	1770	Rmet_5569	0	Op1656f_1	NA
3371	TSS_316732-3	46	9	-44	TCATAAAGCG	-13	GGGAAGATCC	-144	5,1	5.60	-8.60	no	4946	Rmet_3775	0	Op1049r_1	NA
3372	TSS_847016-3	46	18	-42	TTGCGCGCAA	-14	GGTAAACTTG	-138	1,1	9.54	-0.60	no	10813	Rmet_4249	0	Op1203r_1	NA
3373	TSS_22762+2	45	11	-41	GCGGCTGTCG	-14	GCCATAGTTT	-143	5,1	7.25	-2.10	no	90	Rmet_0019	0	Op0003f_3	NA
3374	TSS_579934+2	45	2	-34	TTTGTGCGCT	-14	GATATGCTGA	-139	2,1	8.70	7.10	s	455	Rmet_0545	0	Op0151f_2	MET-EN-Methane
3375	TSS_1322993+2	45	6	-49	TTGACGACGG	-12	GTTTTCTTTC	-146	1,1	1.86	-16.60	no	308	Rmet_1204	0	Op0349f_2	NA
3376	TSS_2369140+2	45	4	-46	TTGCGTGGCC	-20	TGGACAGTCT	-140	1,1	5.34	-3.60	no	796	Rmet_2167	0	Op0623f_1	GIP-PTL-Exp
3377	TSS_3605995+2	45	6	-35	TTGCGCGCCT	-13	GTGAAATTGT	-140	1,1	6.10	10.20	s	12021	Rmet_6590	0	Op0931f_1	NA
3378	TSS_3785796+2	45	10	-47	TTGTCGAGCA	-16	GGTAGAAGTC	-146	1,1	2.01	-8.60	no	6375	Rmet_3502	0	Op0971f_1	NA
3379	TSS_956299-2	45	8	-38	GGACAAGCGC	-13	GGCAAGCTGC	-139	4,1	8.21	-0.60	no	537	Rmet_R0011	0	Op0254r_3	NA
3380	TSS_1167114+3	45	11	-43	TTTCTTTGGG	-17	TGTACTATCC	-138	2,1	13.06	-1.10	no	187	Rmet_4556	int	Op1306f_4	NA
3381	TSS_1809254+3	45	8	-42	TTGCCCGCTG	-14	GGTAGGATTG	-137	1,1	8.99	0.40	(w)-sba	55	Rmet_5115	0	Op1506f_1	EIP-TRA-Ion
3382	TSS_2017233-3	45	7	-35	TACACGTATT	-14	GCTAGAATGC	-139	1,1	6.10	10.60	s	5386	Rmet_5308	0	Op1569r_1	NA
3383	TSS_111684+2	45	5	-42	GCAAGAACCT	-15	TGCATCATCT	-142	4,1	8.37	-3.60	no	861	Rmet_0105	can	Op0031f_3	MET-EN-Nitrog
3384	TSS_3412344+2	45	4	-37	TCCACGAATA	-13	TGGATTATGC	-146	1,1	4.65	7.40	s	4147	Rmet_6577	0	Op0883f_1	NA
3385	TSS_1572879-2	45	14	-34	TATCGGCGCT	-13	GGCACGATGA	-142	2,1	8.81	5.60	m	26	Rmet_1451	0	Op0404r_1	NA
3386	TSS_2521675-2	45	8	-37	TTGACTCTCC	-14	TTCAGCATCC	-141	1,1	6.23	10.40	s	17	Rmet_2299	0	Op0656r_1	NA
3387	TSS_2642165-2	45	3	-37	GCGTCACCAC	-14	GCTACTATCG	-139	5,1	8.35	6.90	s	221	Rmet_2431	plsX	Op0680r_2	MET-LIP-FASyn
3388	TSS_3427791-2	45	10	-33	TTGCCCGTAA	-9	ACCAGTGTGT	-147	1,1	3.77	6.20	(m)-ba	105	Rmet_R0050	0	Op0886r_1	NA
3389	TSS_3790311-2	45	10	-38	TTCTAAGTCT	-13	GTCATCATGC	-147	1,1	6.58	5.90	m	274	Rmet_6599	0	Op0970r_2	NA
3390	TSS_2035339+3	45	9	-41	GCGGCCCGCG	-15	GCTATGATGC	-135	5,1	11.83	-0.10	no	27	Rmet_5326	zneR	Op1576f_2	EIP-SIG-2Comp
3391	TSS_156772-3	45	7	-47	TTGCTCGGCG	-13	GAGAGAATGA	-140	1,1	6.91	-11.60	no	1463	Rmet_6616	0	Op1007r_1	NA
3392	TSS_301158-3	45	10	-47	GCAGCTAGAC	-13	CGTAAGATGG	-141	5,1	8.53	-16.60	no	84	Rmet_3761	0	Op1047r_1	NA
3393	TSS_181983-4	45	3	-41	TGGCGCACAG	-14	GATACACTGC	-140	1,1	8.34	-0.10	no	14	Rmet_6120	copL	Op1868r_1	NA
3394	TSS_140921+5	45	11	-46	TGCAACGCGC	-14	AGTAGACTTC	-143	1,1	6.26	-10.60	no	28	Rmet_6285	0	Op1916f_2	NA
3395	TSS_1226090+2	45	9	-38	TCGACCACAA	-14	ACCAGAATGC	-140	1,1	6.68	7.90	s	1648	Rmet_1122	gltP2	Op0321f_1	NA
3396	TSS_2322724+2	45	2	-44	TCGCGCCGAC	-15	CATAAACTGA	-140	1,1	6.54	-5.10	no	16	Rmet_2134	trxA	Op0615f_1	GIP-PTL-Fold
3397	TSS_3870672+2	45	4	-44	TGCCACAAGG	-13	CTCATCATCG	-145	1,1	4.66	-8.60	no	3809	Rmet_3571	copQ1	Op0991f_1	NA
3398	TSS_513384-2	45	11	-34	TTGAAACGAT	-7	TCCACAATGT	-141	1,1	9.43	7.20	(s)-ba	2527	Rmet_0482	0	Op0128r_1	NA
3399	TSS_2632672-2	45	8	-34	TGGACAGCAG	-14	GGTATCGTAT	-139	1,1	6.27	10.10	s	1117	Rmet_2421	lepA	Op0680r_4	NA
3400	TSS_151599+3	45	10	-43	TGCAATCACG	-16	GCTAACGTCC	-145	1,1	4.18	-3.60	no	2029	Rmet_R0074	0	Op1006f_1	NA
3401	TSS_1166678+3	45	2	-44	TCGTGCGGCG	-15	CTTACTCTTG	-143	1,1	4.14	-6.10	no	219	Rmet_4555	0	Op1306f_3	NA
3402	TSS_1202839+3	45	7	-37	TTGCCCAGGC	-1	TAGAGCATTT	-141	1,1	6.05	-3.60	no	3027	Rmet_4587	0	Op1320f_1	NA
3403	TSS_731330-3	45	2	-34	GCACAAGCCC	-14	GCCACAATGG	-135	4,1	10.50	3.60	m	2260	Rmet_4153	tnpA	Op1169r_1	NA
3404	TSS_903724-3	45	7	-35	TCGGCAATTG	-13	GATATGCTTT	-141	5,1	8.87	11.70	s	85	Rmet_4308	0	Op1227r_1	NA
3405	TSS_1748237-3	45	2	-42	CTCCGAGGGG	-12	GGTACATTGA	-143	1,1	3.09	-6.10	no	18	Rmet_5057	0	Op1489r_1	NA
3406	TSS_176005-4	45	9	-37	TCGTACATCA	-14	GTTACGATCG	-140	1,1	6.96	7.90	s	5587	Rmet_6133	silD	Op1874r_1	NA
3407	TSS_937416+2	44	16	-35	TTGATATGGC	-14	TCGAAGATTG	-143	1,1	6.97	11.60	s	258	Rmet_0858	spoT	Op0245f_2	MET-NUC-Pur

3408	TSS_1562780+2	44	13	-35	TTGCGTTCGT	-15	TGTATCCTGA	-141	1,1	7.96	9.60	s	1419	Rmet_1443	*yaeT*	Op0403f_3	NA
3409	TSS_1176978-2	44	10	-41	GTCGGGACGG	-14	GGCATCCTCG	-143	4,1	5.60	-4.10	*no*	472	Rmet_1084	*0*	Op0312r_1	NA
3410	TSS_2227626-2	44	12	-40	CGGCGTAACA	-12	CATACAGTCG	-149	1,1	1.17	-3.10	*no*	51	Rmet_2055	*ugpB*	Op0596r_1	EIP-TRA-ABC
3411	TSS_3482764-2	44	5	-41	TTGTGGCTAC	-13	CCCATACTTC	-144	1,1	6.87	0.40	*(w)-sba*	39	Rmet_3208	*0*	Op0902r_1	NA
3412	TSS_1030+3	44	3	-38	TGGTAGGGGA	-15	CGTATCATCG	-141	1,1	7.05	7.90	s	1737	Rmet_5819	*parA*	Op1758f_1	DIV-Division
3413	TSS_152384+3	44	5	-45	TGGCCTGCGC	-15	AATAAAATGG	-141	1,1	8.42	-5.10	*no*	1244	Rmet_R0074	*0*	Op1006f_1	NA
3414	TSS_684411+3	44	3	-44	TCGCCGGATC	-15	GATAGCATTT	-141	1,1	7.02	-4.10	*no*	70	Rmet_4113	*0*	Op1158f_1	NA
3415	TSS_140957-3	44	6	-43	TTGCCGCCGC	-12	ACTACCATAG	-138	1,1	7.87	-3.60	*no*	42	Rmet_5936	*0*	Op1805r_3	NA
3416	TSS_61958+4	44	5	-38	TGCCGTCGAA	-14	GGAATACTGA	-145	1,1	4.15	4.40	m	1852	Rmet_5994	*gntR*	Op1827f_1	NA
3417	TSS_955337+2	44	8	-43	TCCCCCCGGC	-15	CCTAGAATTT	-141	1,1	6.94	-2.60	*no*	329	Rmet_0874	*0*	Op0253f_1	NA
3418	TSS_3038760+2	44	17	-37	TTGCACGCAC	-7	TTGACAGTTT	-145	1,1	5.59	1.40	*(w)-ba*	289	Rmet_2792	*0*	Op0785f_1	NA
3419	TSS_3263765+2	44	4	-35	TTGCACCTGT	-14	TCGATTATGT	-142	1,1	7.11	10.60	s	8291	Rmet_3013	*yodB*	Op0847f_1	NA
3420	TSS_345514-2	44	8	-38	TGGCCGCGGT	-13	GCTAGATTGT	-140	1,1	7.73	5.90	m	4654	Rmet_0322	*tnmB*	Op0092r_1	NA
3421	TSS_3466081-2	44	6	-28	TTCACGAACA	0	CGCATCGTTT	-145	1,1	4.00	-2.30	*no*	22	Rmet_3190	*dsbC*	Op0896r_1	GIP-PTL-Fold
3422	TSS_1164540+3	44	8	-40	TTACACAAAG	-14	CCTATACTTT	-142	4,1	7.87	2.90	*(w)-sba*	1181	Rmet_4553	*0*	Op1306f_3	NA
3423	TSS_1388211+3	44	4	-46	TGGCGCAAGG	-14	CTTATAGTAG	-142	1,1	6.61	-9.10	*no*	69	Rmet_4744	*nhoA*	Op1376f_1	NA
3424	TSS_1393948+3	44	12	-40	TGCCCAGATC	-12	CCCATGCTCC	-145	1,1	4.12	-0.60	*no*	18	Rmet_4750	*0*	Op1378f_1	EIP-TRA-Ion
3425	TSS_2361559+3	44	11	-43	GGCGAACAGC	-15	TTTATTATCC	-141	4,1	8.99	-5.60	*no*	2364	Rmet_5608	*cyoA*	Op1674f_1	MET-EN-OxPhos
3426	TSS_61582-3	44	11	-40	TGCCGCTGAT	-14	GGTAGGATGC	-140	1,1	7.21	0.40	*(w)-sba*	72	Rmet_5865	*0*	Op1775r_1	NA
3427	TSS_2423379-3	44	10	-36	CCGAAAGATC	-13	GCTACAATGC	-143	1,1	5.69	8.90	s	22	Rmet_5663	*pstS*	Op1693r_3	EIP-TRA-ABC
3428	TSS_226402-4	44	5	-47	GCAAGGGCGC	-24	TCTACAATGG	-138	4,1	11.69	-4.60	*no*	2356	Rmet_6078	*0*	Op1852r_1	NA
3429	TSS_1295212-2	44	3	-39	TTGTCACCCG	-13	CCTACAGTGT	-138	1,1	7.14	6.40	s	29	Rmet_1181	*gdhB*	Op0342r_2	MET-EN-Nitrog
3430	TSS_3628608-2	44	5	-46	TGACTAACAA	-15	GATAAACTTT	-151	4,1	1.78	-9.60	*no*	413	Rmet_3351	*0*	Op0932r_1	NA
3431	TSS_3783718-2	44	3	-38	TTTGTGTTCG	-14	GTTACGATTC	-142	2,1	11.57	5.90	m	29	Rmet_3492	*0*	Op0970r_2	MET-CAH-But
3432	TSS_478227+3	44	16	-29	TGGAACCACT	-3	AGGACCGTGA	-144	1,1	2.68	-1.30	*no*	419	Rmet_6634	*0*	Op1098f_2	NA
3433	TSS_971559+3	44	10	-26	AAAACAGGCG	-6	GTGTGTGGCG	-150	3,0	0.99	-8.40	*no*	175	Rmet_4373	*0*	Op1248f_1	NA
3434	TSS_2259794+3	44	9	-46	TCGCGCATCT	-14	GCTACGATGG	-140	1,1	8.25	-10.10	*no*	11784	Rmet_5525	*0*	Op1642f_1	NA
3435	TSS_18035-3	44	14	-44	TGACAGATGT	-13	GCTAACCTGA	-146	4,1	6.73	-9.60	*no*	42	Rmet_5829	*pstS1*	Op1763r_1	EIP-TRA-ABC
3436	TSS_528492-3	44	14	-43	TCCATCGGAA	-13	GGTATTGTTC	-145	1,1	4.46	-5.60	*no*	6555	Rmet_3961	*gabD*	Op1111r_1	MET-CAH-But
3437	TSS_1308323-3	44	7	-36	TTGCCCGCCG	-14	GGTAGCATTG	-136	1,1	8.76	12.40	s	25	Rmet_4675	*0*	Op1353r_2	NA
3438	TSS_2352412-3	44	15	-46	TGGCAGGTTT	-13	CCTATCCTTA	-142	1,1	6.43	-11.10	*no*	25	Rmet_5598	*0*	Op1667r_2	NA
3439	TSS_5647+4	44	14	-44	TGGTCCGCAG	-21	GACATGGTGT	-146	1,1	3.44	0.90	*w*	95	Rmet_6347	*0*	Op1845f_1	NA
3440	TSS_204309+4	44	18	-49	TCAAAGCCGC	-15	AGGATAATTC	-143	4,1	8.23	-14.60	*no*	415	Rmet_6095	*0*	Op1857f_1	NA
3441	TSS_210400-4	44	13	-34	TTGACCCGCG	-6	CCAAAGCTTG	-143	1,1	4.26	4.20	*(m)-ba*	22	Rmet_6092	*0*	Op1856r_1	NA
3442	TSS_2226751+2	43	3	-39	TTGCGACACA	-15	GGCATTCTTG	-140	1,1	6.86	6.40	s	1029	Rmet_2056	*0*	Op0597f_1	NA
3443	TSS_1777958-2	43	6	-27	TTGCACGGTT	0	GGTATGCTTG	-141	1,1	9.32	-1.80	*no*	54	Rmet_1644	*0*	Op0462r_2	NA
3444	TSS_2696159-2	43	8	-41	TCGACCGCTT	-13	CCTACATTGA	-142	1,1	6.45	0.90	*(w)-sba*	1769	Rmet_2480	*livK1*	Op0688r_2	EIP-TRA-ABC
3445	TSS_3355732-2	43	9	-34	TGGCAGTTTT	-13	TTGAGAATGA	-144	1,1	6.50	8.10	s	231	Rmet_3096	*0*	Op0872r_1	NA
3446	TSS_3614164-2	43	10	-49	TTGACTGGCC	-14	AATAAAATGG	-140	1,1	9.79	-11.60	*no*	1245	Rmet_R0060	*0*	Op0930r_1	NA
3447	TSS_3631077-2	43	13	-45	GCAGGAACAC	-21	TGTACGCTTC	-141	4,1	9.85	-5.60	*no*	543	Rmet_3353	*tnpA*	Op0934r_1	NA
3448	TSS_328439-3	43	10	-37	TGCTCAAGCT	-14	GATAACATCG	-138	1,1	6.28	8.40	s	16653	Rmet_3775	*0*	Op1049r_1	NA
3449	TSS_744493-3	43	4	-45	CTGATAACTA	-12	GTTATGTTAC	-147	1,1	2.55	-9.60	*no*	15423	Rmet_4153	*tnpA*	Op1169r_1	NA
3450	TSS_1374567-3	43	12	-38	CCGTCCGGTT	-13	CTTATCATCC	-140	5,1	7.87	3.90	m	1637	Rmet_6682	*0*	Op2078r_1	NA
3451	TSS_1951180-3	43	6	-46	TAGAAGCGCG	-14	TTTATATTCG	-146	1,1	4.36	-8.10	*no*	325	Rmet_5250	*0*	Op1549r_1	DIV-MOT-Chemotax
3452	TSS_34552-4	43	6	-38	GGAAAAGAAC	-9	TCGAACTTGC	-147	4,1	6.29	-3.60	*no*	614	Rmet_6026	*0*	Op1836r_1	NA
3453	TSS_229048-4	43	3	-45	TTGTCGAAAT	-13	GGCAAAATGG	-140	1,1	9.31	-6.60	*no*	311	Rmet_6072	*0*	Op1850r_1	NA
3454	TSS_144459+2	43	14	-37	TTGCAATGTG	-14	TGTAAAATCG	-136	1,1	10.98	11.40	s	35	Rmet_0140	*argB*	Op0039f_1	MET-AA-Urea
3455	TSS_1594045+2	43	10	-36	TTGACCTTGT	-15	TTGATACTCC	-139	1,1	8.24	12.80	s	1606	Rmet_1475	*0*	Op0413f_1	NA
3456	TSS_1623041+2	43	13	-46	GCAGGGGACT	-14	CATAACATGC	-143	4,1	7.67	-14.60	*no*	10	Rmet_1503	*tnpA*	Op0423f_1	GIP-REP-Recomb
3457	TSS_2357297+2	43	6	-33	AAATTCCGCG	-13	TGTCGCAATA	-143	3,0	7.72	-2.40	no	422	Rmet_2157	*0*	Op0621f_1	NA
3458	TSS_3619001+2	43	8	-38	TCGCCTGAGT	-14	GCTATGATGC	-137	1,1	9.01	7.90	s	47	Rmet_3343	*0*	Op0931f_3	NA
3459	TSS_2405997-2	43	4	-34	TCCAGCAGGA	-14	GTTACCGTCT	-140	1,1	4.10	5.60	m	1166	Rmet_2194	*yeaS*	Op0630r_3	NA
3460	TSS_2796444-2	43	21	-45	TTGTTACGGG	-5	GCTATGATTC	-141	1,1	9.20	-13.60	*no*	34	Rmet_2570	*pmbA*	Op0712r_1	NA
3461	TSS_2929589-2	43	8	-42	TGGGGAAGGG	-13	CTTATGATTC	-146	1,1	1.69	-1.10	*no*	456	Rmet_2697	*0*	Op0760r_1	NA
3462	TSS_144984-3	43	5	-33	TGCAAGTGCC	-13	GGCAAACTGT	-140	1,1	6.04	4.60	m	70	Rmet_5938	*0*	Op1805r_2	NA
3463	TSS_716302-3	43	6	-45	GCCAACACGC	-13	GGTATGATTG	-138	4,1	11.30	-11.60	*no*	82	Rmet_4141	*0*	Op1167r_1	NA
3464	TSS_805316-3	43	6	-38	TCGACGAGAA	-16	GCGAGTATCA	-143	1,1	4.78	8.90	s	2174	Rmet_4218	*0*	Op1197r_1	NA
3465	TSS_541054+2	43	7	-43	TATCGGCGGG	-15	GGTAGTATCG	-142	2,1	11.06	-4.60	*no*	68	Rmet_0510	*0*	Op0137f_1	NA
3466	TSS_911298+2	43	19	-37	TTGACCGTGT	-15	ATTATACTTC	-140	1,1	10.06	13.40	s	79	Rmet_0834	*gudD*	Op0239f_1	MET-CAH-Ascorb
3467	TSS_2086140+2	43	9	-41	TGCCATGATG	-15	AATAGGATAT	-143	1,1	4.05	-0.60	*no*	5160	Rmet_1924	*0*	Op0561f_1	NA
3468	TSS_507268-2	43	8	-45	TTTGGCTTCT	-25	AGTACGCTGC	-145	2,1	8.10	-1.70	*no*	137	Rmet_0478	*0*	Op0124r_1	NA

3469	TSS_1748249-2	43	6	-46	GCACGAATGC	-14	GATACCATCG	-135	4,1	10.28	-13.60	no	7729	Rmet_1613	*tnpA*		Op0450r_1	NA
3470	TSS_2398995-2	43	11	-37	TAGCGATTTG	-14	GCCAAAATAT	-143	1,1	4.62	8.90	s	107	Rmet_2189	*rrnJ*		Op0628r_1	DIV-Division
3471	TSS_2578591-2	43	11	-34	TCGCGGTCGA	-13	GATACTATTG	-139	1,1	7.21	8.10	s	162	Rmet_2365		*0*	Op0666r_1	NA
3472	TSS_3213890-2	43	20	-34	TTCCCAGCTA	-13	CCAATAATGC	-142	1,1	6.69	11.10	s	81	Rmet_2955	*yceJ*		Op0832r_1	NA
3473	TSS_3428585-2	43	13	-38	TTGCATTGGA	-13	TGTAGGATAG	-142	1,1	8.16	6.40	s	899	Rmet_R0050		*0*	Op0886r_1	NA
3474	TSS_569292+3	43	1	-36	TGGCGAAGGC	-15	TATAGGCTGG	-142	1,1	6.40	9.30	s	3864	Rmet_6639		*0*	Op2062f_1	NA
3475	TSS_922684+3	43	7	-44	TGGTCGTTTA	-16	CGTAACATCA	-146	1,1	6.20	-3.10	no	24	Rmet_4329		*0*	Op1232f_1	NA
3476	TSS_1315289+3	43	5	-35	TTGTCGCCAT	-14	CATATCATTT	-139	1,1	7.79	12.60	s	643	Rmet_4687		*0*	Op1356f_1	NA
3477	TSS_9842-4	43	4	-38	GCAGGCGTAC	-13	GCTAGCATCG	-140	4,1	8.78	0.40	w	5019	Rmet_6060	*repA*		Op1846r_1	NA
3478	TSS_15665+5	43	8	-42	TCTGATATCT	-15	TGTAGCGTGT	-146	5,1	4.94	-3.60	no	865	Rmet_6327		*0*	Op1922f_1	NA
3479	TSS_1000447+2	42	1	-49	TGCCCACGGA	-15	ATTATCCTCA	-145	1,1	5.00	-14.60	no	49	Rmet_0915	*psd*		Op0263f_3	MET-LIP-GlycPLip
3480	TSS_2969505+2	42	2	-23	TTGCGCATGG	0	GAAAGGATCT	-143	1,1	5.38	-2.80	no	2172	Rmet_2736	*apaH*		Op0771f_1	MET-NUC-Pur
3481	TSS_3215803+2	42	10	-30	TCGCGCTCGC	-6	GTTATGAAGT	-142	1,1	3.08	1.70	(w)-nu	90	Rmet_2957	*gst-1*		Op0833f_2	MET-OAA-GSH
3482	TSS_3629185+2	42	14	-42	TTGAATCGCT	-14	GCTACCCTGT	-142	1,1	8.26	-0.60	no	1535	Rmet_3354		*0*	Op0935f_1	NA
3483	TSS_3675952+2	42	8	-37	TGGCGTGTCA	-14	GCTACACTGG	-136	1,1	8.23	7.90	s	21	Rmet_3397	*ypeA*		Op0943f_1	NA
3484	TSS_2117373-2	42	15	-36	TGGCGGCGTG	-13	TTTACACTCC	-144	1,1	7.50	8.90	s	104	Rmet_1946		*0*	Op0570r_1	NA
3485	TSS_3237010-2	42	3	-33	GCAGAATTGG	-13	GGCACTATAG	-144	5,1	5.76	1.60	w	92	Rmet_6570		*0*	Op0838r_1	NA
3486	TSS_3613818-2	42	14	-38	TTGCATTGGA	-13	TGTAGGATAG	-142	1,1	8.16	6.40	s	899	Rmet_R0060		*0*	Op0930r_1	NA
3487	TSS_1166862+3	42	6	-45	TCGCCTGTGA	-13	CGTAAGCTGG	-143	1,1	6.01	-9.10	no	35	Rmet_4555		*0*	Op1306f_3	NA
3488	TSS_1963786+3	42	13	-36	TTGCACGATC	-14	CGCATCATGG	-139	1,1	7.56	11.40	s	459	Rmet_5266	*fliK*		Op1552f_2	DIV-MOT-Flagel
3489	TSS_304321-3	42	7	-46	GCAAGAATCG	-13	CGTATTCTGG	-142	4,1	7.81	-13.60	no	27	Rmet_3765	*ada*		Op1047r_1	GIP-REP-Recomb
3490	TSS_2317533-3	42	10	-43	TTGAACCAAT	-14	GCTAAAGTTG	-142	1,1	9.22	-1.60	no	1967	Rmet_5562		*0*	Op1651r_1	NA
3491	TSS_533462+2	42	9	-38	TCGCGATGGG	-14	CCTATCATCT	-139	1,1	7.64	7.90	s	434	Rmet_0503	*cbbA3*		Op0135f_1	MET-CAH-FbP
3492	TSS_2829084+2	42	13	-35	TTGACAGGTG	-15	GATATACTGG	-139	1,1	10.80	13.60	s	116	Rmet_2601		*0*	Op0729f_1	NA
3493	TSS_3458226+2	42	13	-46	TGGTCACGGC	-17	GCGAGTATCG	-142	1,1	4.87	-6.10	no	199	Rmet_3186		*0*	Op0895f_1	EIP-TRA-ABC
3494	TSS_1252671-2	42	4	-45	TTGCCCTTCG	-13	GTGATACTGC	-139	1,1	8.46	-6.60	no	3328	Rmet_1141		*0*	Op0326r_1	NA
3495	TSS_1728242-2	42	6	-35	TTCCCATTCT	-14	GCCAGACTTC	-145	1,1	7.35	10.10	s	50	Rmet_1601		*0*	Op0444r_2	NA
3496	TSS_3639210-2	42	13	-32	TGGAAGAGCG	-5	TATAGCATGG	-140	1,1	7.02	2.70	(w)-ba	8676	Rmet_3353	*tnpA*		Op0934r_1	NA
3497	TSS_41286+3	42	8	-39	TTGCGTCATC	-16	TATATGATTT	-140	1,1	8.50	8.40	s	149	Rmet_5848		*0*	Op1768f_1	NA
3498	TSS_138258+3	42	7	-42	GGCGGACTAA	-14	CCGATATTCA	-144	4,1	3.20	-5.60	no	25	Rmet_5935		*0*	Op1804f_1	DIV-MOT-Chemotax
3499	TSS_337414+3	42	3	-36	TTGCGCTGGC	-15	CCTAGACTTC	-137	1,1	8.94	10.80	s	67	Rmet_3798		*0*	Op1054f_2	NA
3500	TSS_529475+3	42	20	-48	TTGTGGATTG	-15	AGCATAATAT	-144	1,1	7.30	-10.60	no	8974	Rmet_3974		*0*	Op1114f_1	NA
3501	TSS_1768057+3	42	6	-42	GGCGCCGCGT	-14	TGTAAACTCC	-139	4,1	7.73	-5.60	no	18	Rmet_5080		*0*	Op1496f_1	NA
3502	TSS_650805-3	42	9	-33	TCGCCGTAAC	-12	CTTAGACTCT	-144	1,1	7.06	8.10	s	29	Rmet_4081		*0*	Op1147r_2	NA
3503	TSS_1793751-3	42	10	-35	TTGAACTGTA	-14	CCTATCTTTG	-141	1,1	7.30	11.60	s	1	Rmet_5101		*0*	Op1501r_1	NA
3504	TSS_9276+4	42	11	-39	TTGAAGCTGA	-3	AGTAACGTCA	-143	1,1	5.85	-5.60	no	742	Rmet_6057		*0*	Op1845f_1	NA
3505	TSS_2930131+2	42	11	-40	GCGGCCTGCG	-14	TTTACGCTGG	-138	5,1	8.21	-1.10	no	1259	Rmet_2699	*acsA*		Op0761f_1	MET-CAH-But
3506	TSS_3256491+2	42	4	-37	TTGACTGTGG	-15	CCAATAATTC	-140	1,1	7.82	13.40	s	991	Rmet_2999	*mmfA*		Op0845f_1	NA
3507	TSS_3338169+2	42	5	-49	TGCCTCATGT	-15	GACAAAATCG	-146	1,1	6.20	-16.60	no	912	Rmet_3084	*rdgC*		Op0867f_1	GIP-REP-Recomb
3508	TSS_1257669-2	42	12	-39	TTGCCGCCCA	-12	GAGACAATGG	-139	1,1	8.24	4.40	m	8326	Rmet_1141		*0*	Op0326r_1	NA
3509	TSS_1554724-2	42	7	-48	TCGATGTGCT	-13	GCGAAAATGG	-138	1,1	7.24	-14.10	no	2311	Rmet_1432	*rsuA1*		Op0402r_1	GIP-TL-Ass
3510	TSS_1581888-2	42	15	-35	TCGAAGCGCA	-14	GTGATAATGC	-139	1,1	7.78	11.10	s	508	Rmet_1460	*ytfF*		Op0408r_1	NA
3511	TSS_2232488-2	42	4	-48	TCGAATGCGA	-13	CGTATCCTGT	-143	1,1	6.16	-14.10	no	78	Rmet_2059	*glnG*		Op0598r_3	EIP-SIG-2Comp
3512	TSS_3195515-2	42	5	-40	GTCCAGAAAC	-13	GACATCATGG	-144	4,1	8.13	-2.10	no	269	Rmet_2940	*dpsA*		Op0830r_3	GIP-REP-Recomb
3513	TSS_3428914-2	42	4	-35	TTGCAATAAA	-11	TGTTTAATAA	-145	1,1	4.55	10.20	s	1228	Rmet_R0050		*0*	Op0886r_1	NA
3514	TSS_108249+3	42	3	-35	TTGTCAAATT	-15	CGTACCATTG	-143	1,1	8.76	11.60	s	33	Rmet_5908		*0*	Op1792f_2	MET-XEN-Others
3515	TSS_462734+3	42	16	-37	TTGACATACG	-15	ACCACACTGG	-143	1,1	7.98	12.40	s	9	Rmet_3915		*0*	Op1096f_1	NA
3516	TSS_495515+3	42	10	-39	TTGCTGACCA	-14	GGCACAATGG	-137	1,1	8.89	5.40	m	202	Rmet_3937		*0*	Op1100f_2	NA
3517	TSS_667150+3	42	9	-34	CCGGCATCCT	-14	GGTATCCTGG	-136	5,1	9.42	6.10	m	1553	Rmet_4100		*0*	Op1154f_2	MET-CAH-Prop
3518	TSS_125980-3	42	12	-49	TTGCCGTTCT	-15	CGTACAATGC	-136	1,1	11.12	-11.60	no	14	Rmet_5924		*0*	Op1799r_1	NA
3519	TSS_387560-3	42	13	-40	TTGCGAGCGA	-12	GCTACAATCG	-137	1,1	11.00	3.40	(m)-sba	30	Rmet_3842		*0*	Op1067r_1	NA
3520	TSS_1761314-3	42	6	-37	TTCGGTTCGT	-13	TCTATCCTTG	-146	2,1	6.65	6.90	s	391	Rmet_5072		*0*	Op1493r_1	MET-AA-Met
3521	TSS_3937-4	42	11	-32	TGAGCACCAG	-12	GAGATCATCA	-147	4,1	4.32	5.60	m	4142	Rmet_6390		*0*	Op1848r_1	NA
3522	TSS_727156+2	41	5	-31	AATCTTTTCG	-8	ATAAGACTGA	-150	2,1	6.37	-0.80	no	1297	Rmet_0661		*0*	Op0183f_1	NA
3523	TSS_2373791+2	41	14	-36	TCGCACTGAC	-14	GATATGCTTA	-141	1,1	7.03	9.90	s	6085	Rmet_R0034		*0*	Op0625f_1	NA
3524	TSS_305720-2	41	3	-37	TTTCGCCTGG	-13	TTTAAAGTCA	-142	2,1	9.99	6.90	s	24	Rmet_R0003		*0*	Op0084r_2	NA
3525	TSS_2359035-2	41	3	-37	TATCTCAGCC	-13	TGTAAGGTGG	-145	2,1	7.01	4.40	m	1613	Rmet_6509		*0*	Op2029r_1	NA
3526	TSS_3781244-2	41	15	-34	TTGAATCTTT	-12	TGTAAGGTCG	-145	1,1	6.69	10.20	s	20	Rmet_6598		*0*	Op0970r_3	NA
3527	TSS_3835641-2	41	4	-42	TCGTGCGTAT	-14	GGTATCATCC	-140	1,1	7.67	-1.10	no	42	Rmet_3537		*0*	Op0978r_1	NA
3528	TSS_74793+3	41	8	-37	TCGCTGAAGA	-15	GGTATATTTC	-140	1,1	8.01	9.90	s	1785	Rmet_5880		*0*	Op1780f_1	NA
3529	TSS_2323198+3	41	6	-36	TCACAGGCCC	-14	CCTATACTGC	-144	4,1	8.85	9.40	s	22	Rmet_5569		*0*	Op1656f_1	NA

3530	TSS_137210+5	41	5	-36	TCCCCCCTGA	-14	CTTAAAATGC	-142	1,1	7.39	10.40	s	1339	Rmet_6283	*0*	Op1916f_1	NA
3531	TSS_1289088+2	41	8	-35	TGGCTTCGAC	-15	TGTAACCTGA	-141	1,1	5.41	7.10	s	497	Rmet_1179	*0*	Op0341f_2	NA
3532	TSS_1803069+2	41	16	-49	TGGCCCGCGT	-13	GTTACAATAA	-142	1,1	9.01	-15.10	*no*	1834	Rmet_1668	*tnp*	Op0469f_1	NA
3533	TSS_2903813+2	41	10	-36	TTGACAGCGT	-16	CCCATAAATG	-143	1,1	4.39	11.80	s	9069	Rmet_2680	*glyA*	Op0757f_1	MET-EN-Methane
3534	TSS_1193660-2	41	13	-28	TTGAATATCG	0	TATAGCGTGG	-144	1,1	5.82	-1.80	*no*	6338	Rmet_1091	*0*	Op0314r_1	NA
3535	TSS_2520920-2	41	6	-36	TACGGCCTCG	-14	CGTACCATTG	-141	2,1	8.12	8.40	s	11427	Rmet_2287	*0*	Op0654r_1	NA
3536	TSS_2772225-2	41	11	-35	TTGCGTACCC	-13	AGCAGGATGT	-144	1,1	5.89	10.20	s	824	Rmet_2546	*0*	Op0706r_1	NA
3537	TSS_2971973-2	41	10	-41	TCGACACTAT	-11	TTTAATGTGT	-146	1,1	5.37	-1.10	*no*	498	Rmet_2735	*rfbB*	Op0770r_1	MET-CAH-NucSug
3538	TSS_476934+3	41	10	-45	TGGCCTGCGC	-15	AATAAAATGG	-141	1,1	8.42	-5.10	*no*	1244	Rmet_R0079	*0*	Op1098f_1	NA
3539	TSS_1061540+3	41	9	-41	TTGCAGCATT	-14	TGCAAAATGG	-141	1,1	9.02	1.40	*(w)-sba*	118	Rmet_4465	*czcR2*	Op1274f_1	EIP-SIG-2Comp
3540	TSS_1650798+3	41	6	-42	TCGAGGCGCT	-22	GAAATCCTGT	-142	1,1	3.68	2.30	*w*	735	Rmet_4974	*tniQ*	Op1460f_1	NA
3541	TSS_2537350+3	41	14	-38	TTTCGGGGCG	-15	AGTAAACTAC	-142	2,1	7.28	6.90	s	351	Rmet_5778	*0*	Op1738f_1	NA
3542	TSS_506851-3	41	11	-46	TCAGCCAGCT	-8	GGTACCGTGG	-140	5,1	8.40	-17.60	*no*	1356	Rmet_3948	*0*	Op1105r_1	NA
3543	TSS_527710-3	41	11	-36	GCGGACCGTG	-13	GTTACTATCG	-140	5,1	9.18	5.90	m	5773	Rmet_3961	*gabD*	Op1111r_1	MET-CAH-But
3544	TSS_1396815-3	41	8	-45	TGGCGGCGGG	-12	CCTATATTGC	-140	1,1	7.40	-9.10	*no*	3041	Rmet_6684	*0*	Op1377r_1	NA
3545	TSS_2378690-3	41	10	-35	TTGCCCGCAG	-13	TTCACCATGA	-144	1,1	6.37	11.20	s	258	Rmet_5620	*0*	Op1677r_1	NA
3546	TSS_62612+5	41	10	-35	TTCCGTTCGT	-14	TGCATCATCT	-142	1,1	5.98	9.10	s	12639	Rmet_6223	*0*	Op1900f_1	NA
3547	TSS_410275+2	41	12	-40	ATGCTGTTCG	-14	ACTAGGCTTG	-143	2,1	7.04	-0.60	*no*	175	Rmet_0385	*0*	Op0105f_2	NA
3548	TSS_1544011+2	41	7	-39	GCAGCCGTAT	-14	CTTAAGATGG	-141	5,1	9.09	0.40	w	1322	Rmet_1428	*ligA*	Op0399f_1	GIP-REP-Recomb
3549	TSS_2479599+2	41	13	-47	GCCGAAGCGT	-15	CCTAGACTCT	-140	4,1	8.90	-14.60	*no*	25	Rmet_2259	*0*	Op0645f_1	NA
3550	TSS_3791387+2	41	6	-31	TGGAGCAAGT	-4	GCAAACCTTA	-145	1,1	3.91	-0.30	*no*	784	Rmet_3502	*0*	Op0971f_1	NA
3551	TSS_1420314-2	41	6	-48	TGGACGAGTG	-5	GCCACAATCT	-142	1,1	8.21	-21.10	*no*	1776	Rmet_1303	*tnpA*	Op0374r_1	GIP-REP-Recomb
3552	TSS_1759504-2	41	8	-34	TTGCGAGGAA	-14	GATATCTTGG	-140	1,1	7.77	9.60	s	1177	Rmet_1629	*0*	Op0456r_1	NA
3553	TSS_3383698-2	41	21	-47	TTGACACGGT	-14	CGTATCATTT	-139	1,1	9.98	-6.60	*no*	109	Rmet_3121	*lpxC*	Op0878r_4	MET-GLYC-LPS
3554	TSS_3570958-2	41	6	-35	GTACAACAGC	-13	GATAACCTGA	-144	4,1	8.13	6.70	s	319	Rmet_3296	*infA*	Op0926r_2	NA
3555	TSS_476951+3	41	9	-36	TTGCAATAAA	-12	TGTTTAATAA	-145	1,1	4.55	10.40	s	1227	Rmet_R0079	*0*	Op1098f_1	NA
3556	TSS_608921+3	41	6	-35	TTGCACCTGT	-14	TCGATTATGT	-142	1,1	7.11	10.60	s	3944	Rmet_4046	*ggt*	Op1140f_1	MET-LIP-Others
3557	TSS_186073-3	41	6	-38	TTGACGCATG	-14	GAGAAAATTT	-141	1,1	8.30	9.40	s	2149	Rmet_3649	*0*	Op1019r_1	NA
3558	TSS_228403-3	41	2	-39	TTCCTCCGCC	-18	GGTCTGGTAG	-148	2,1	3.84	4.30	m	13562	Rmet_3677	*stpA*	Op1025r_1	NA
3559	TSS_1712841-3	41	6	-40	TCGCCCACCT	-13	GGCATACTCA	-138	1,1	6.93	1.90	*(w)-sba*	8042	Rmet_5021	*0*	Op1477r_1	NA
3560	TSS_2442145-3	41	10	-35	TTGCCCGAAG	-2	GTTAAGATCG	-141	1,1	9.26	1.20	*(w)-ba*	112	Rmet_5683	*0*	Op1697r_1	NA
3561	TSS_1655168+2	40	11	-37	TTGCCTGCTG	-15	ACTATCATGA	-138	1,1	8.18	12.40	s	94	Rmet_1534	*0*	Op0427f_3	NA
3562	TSS_2110205+2	40	11	-45	TCGATCTGAT	-15	CGGATAATCA	-141	1,1	6.55	-5.10	*no*	4392	Rmet_1944	*0*	Op0569f_1	MET-LIP-Ster
3563	TSS_2704823+2	40	12	-35	TCGATTGGAG	-14	CCCACAATCT	-143	1,1	5.84	10.10	s	2863	Rmet_2493	*0*	Op0689f_2	NA
3564	TSS_3368987+2	40	9	-36	GTAAAACAAA	-12	GGTCAAATCA	-145	4,1	4.94	5.90	m	1280	Rmet_3110	*pilD*	Op0875f_2	GIP-PTL-T2S
3565	TSS_3588393+2	40	5	-39	TTGCCATGTT	-15	AATACAGTCT	-143	1,1	8.26	8.40	s	5720	Rmet_3329	*0*	Op0929f_1	NA
3566	TSS_3863643+2	40	6	-41	CCAGAATGCC	-14	ACCACAATTG	-141	5,1	7.81	-3.60	*no*	4171	Rmet_3566	*0*	Op0989f_1	NA
3567	TSS_910379-2	40	13	-35	TTGCGGCATG	-14	CATATGATGT	-139	1,1	8.38	11.60	s	79	Rmet_0832	*aldH*	Op0238r_1	NA
3568	TSS_2201824-2	40	13	-48	TCCTCGGCGA	-13	CCCATTTTTT	-151	1,1	0.98	-16.60	*no*	160	Rmet_2033	*0*	Op0594r_2	NA
3569	TSS_2224899-2	40	9	-45	TTCATCACGC	-15	CGTATCCTGC	-142	1,1	6.70	-6.10	*no*	461	Rmet_2052	*ugpC*	Op0596r_1	EIP-TRA-ABC
3570	TSS_3612871-2	40	12	-28	TGGAACCACT	-2	AGGACCGTGA	-144	1,1	2.68	-2.30	*no*	364	Rmet_R0059	*0*	Op0930r_2	NA
3571	TSS_3645194-2	40	3	-43	TTGAGGTAAG	-12	GCTATTCTCG	-141	1,1	8.63	-3.60	*no*	122	Rmet_3368	*0*	Op0936r_1	NA
3572	TSS_3754440-2	40	7	-48	CTGTACGTCC	-24	GAGAACATCT	-146	1,1	0.80	-6.60	*no*	2644	Rmet_R0066	*0*	Op2056r_1	NA
3573	TSS_155398+3	40	5	-31	TGGCTTAAGT	-11	ACCATGATTC	-143	1,1	5.96	4.10	m	51	Rmet_3620	*degP*	Op1008f_1	NA
3574	TSS_2564353-3	40	4	-34	TTTCTGCGCG	-13	GCTATAGTCG	-134	2,1	13.24	9.10	s	0	Rmet_5804	*0*	Op1749r_1	NA
3575	TSS_258765+2	40	3	-30	AAATGCGTCA	-10	TATCCAGATC	-143	3,0	7.51	-5.40	*no*	103	Rmet_0242	*gshA*	Op0071f_3	MET-AA-GluGSH
3576	TSS_769560+2	40	5	-37	GCGGAAGCTG	-14	CACATAATCC	-142	5,1	8.64	6.90	s	2915	Rmet_0700	*dadA2*	Op0197f_1	MET-EN-Nitrog
3577	TSS_821613+2	40	10	-39	TTGACTCGTA	-8	TATACAGTAT	-143	1,1	7.08	1.40	*(w)-ba*	1712	Rmet_0745	*0*	Op0211f_1	NA
3578	TSS_2326853+2	40	13	-41	TTCTCATCAT	-13	GGTATATTTC	-138	1,1	8.49	1.90	*(w)-sba*	1073	Rmet_2139	*mdtK*	Op0615f_2	EIP-TRA-Ion
3579	TSS_2363817+2	40	7	-43	TTGCCCGGAA	-15	TGTACTCTCG	-140	1,1	7.54	-1.60	*no*	2126	Rmet_2164	*0*	Op0623f_1	NA
3580	TSS_3320079+2	40	11	-39	TTGTATCCCA	-14	TCTAAGCTAC	-143	1,1	5.65	4.40	m	35	Rmet_3063	*aroQ*	Op0861f_3	MET-AA-PheTyrTrp
3581	TSS_3836022+2	40	2	-31	AGACGCGCCG	-11	TACCTCTGGG	-149	3,0	3.89	-5.40	no	57	Rmet_6601	*0*	Op0979f_1	NA
3582	TSS_1561027-2	40	10	-39	GCAGCACGCC	-13	AGTACCATGA	-138	5,1	9.39	0.40	w	5115	Rmet_1434	*map*	Op0402r_1	GIP-TL-Ass
3583	TSS_1911295-2	40	6	-42	GCCACAGTGG	-13	CGGACAATAG	-144	4,1	6.53	-4.60	*no*	4599	Rmet_1756	*0*	Op0500r_1	NA
3584	TSS_2483446-2	40	9	-36	GGAAGAACCC	-7	AGGAGGGTAC	-145	4,1	4.15	-1.60	*no*	18650	Rmet_2249	*dat*	Op0644r_1	MET-AA-Lys_Deg
3585	TSS_2641186-2	40	17	-48	TCAAGAGCAC	-13	AGTATGATCA	-142	4,1	9.28	-13.60	*no*	328	Rmet_2430	*fabH*	Op0680r_2	MET-LIP-FASyn
3586	TSS_3152409-2	40	3	-33	TGAGAACTGG	-13	GGTAGGGTTT	-144	4,1	4.78	4.60	m	255	Rmet_2895	*icd*	Op0814r_1	MET-CAH-TCA
3587	TSS_445179+3	40	6	-28	TGCAGCATGC	-6	GTCAGTATCG	-144	1,1	4.45	1.20	*(w)-nu*	94	Rmet_3900	*bug*	Op1088f_1	NA
3588	TSS_989086+3	40	3	-37	TTCCTGCTTT	-15	GGTACATTAC	-137	1,1	6.97	8.90	s	30	Rmet_4392	*fabB*	Op1248f_2	MET-LIP-FASyn
3589	TSS_2577334+3	40	4	-37	TTGCGCCCTC	-13	CCTATAGTTC	-140	1,1	8.17	9.40	s	1992	Rmet_5816	*cspA*	Op1756f_1	NA
3590	TSS_2379516-3	40	15	-43	GGCGCAAACC	-14	GGTAGACTCG	-141	4,1	8.93	-6.60	*no*	818	Rmet_5621	*0*	Op1677r_1	NA

3591	TSS_41769-5	40	9	-28	GGAAAGCAAT	-7	CTGAAATTAC	-148	4,1	5.09	-2.40	no	10055	Rmet_6344	*merR*		Op1929r_1	NA
3592	TSS_17400+2	40	3	-49	TGGCGGCGTG	-14	GTGACAATAC	-139	1,1	6.55	-16.10	no	483	Rmet_0014		*0*	Op0003f_1	NA
3593	TSS_585546+2	40	5	-45	GGCCAGGTAG	-13	GCTAACGTCG	-142	4,1	5.31	-14.60	no	1167	Rmet_0550		*0*	Op0153f_1	NA
3594	TSS_1149768+2	40	4	-38	TTGTCAACTG	-1	TGTATCGTCT	-143	1,1	6.50	-3.60	no	97	Rmet_1056	*ftsB*		Op0301f_3	DIV-Division
3595	TSS_1900726+2	40	4	-43	GCAACACCCT	-14	ATTACAGTCT	-144	4,1	7.05	-5.60	no	76	Rmet_1751	*agrR*		Op0499f_1	EIP-SIG-2Comp
3596	TSS_1982305+2	40	6	-38	TTCATAAATT	-15	ACTAATATTT	-143	1,1	7.04	8.90	s	3428	Rmet_1830	*gcvT*		Op0531f_1	NA
3597	TSS_3023246+2	40	3	-41	TCGCCCTTTA	-14	TCGATCATCA	-139	1,1	5.13	0.90	(w)-sba	467	Rmet_2776		*0*	Op0781f_1	NA
3598	TSS_3072674+2	40	10	-38	TTGATGTCGA	-14	GGCACCATTC	-140	1,1	7.58	7.40	s	548	Rmet_2826		*0*	Op0795f_3	NA
3599	TSS_401845-2	40	7	-45	GTCCACAAGC	-13	CATAAAGTTA	-143	4,1	7.14	-12.10	no	14000	Rmet_0365	*coxS*		Op0102r_1	MET-EN-Methane
3600	TSS_1982265-2	40	4	-40	TTGCGCGAAA	-13	TGTAATTTAT	-146	1,1	5.55	1.40	(w)-sba	105	Rmet_1825		*0*	Op0530r_2	GIP-PTL-Exp
3601	TSS_2675307-2	40	2	-37	CCTGACGCGG	-14	AGGACAATGG	-139	5,1	8.10	3.40	m	45	Rmet_2463	*folC*		Op0688r_4	NA
3602	TSS_3629028-2	40	5	-34	TGGCCATGCT	-14	GCTATAGTCG	-141	1,1	9.00	10.10	s	833	Rmet_3351		*0*	Op0932r_1	NA
3603	TSS_474630+3	40	16	-23	TTCATGACTG	-1	AACAAGGTAG	-147	1,1	1.60	-3.30	no	105	Rmet_R0076		*0*	Op1098f_1	NA
3604	TSS_1385017+3	40	9	-38	TGGATGATTG	-14	AGTATGGTTG	-143	1,1	5.47	6.90	s	30	Rmet_4740		*0*	Op1374f_1	NA
3605	TSS_1854990+3	40	6	-38	CTGATCTGCG	-15	GGTAACATTC	-142	1,1	4.55	6.40	s	921	Rmet_5157		*0*	Op1518f_1	NA
3606	TSS_2483418+3	40	9	-41	TGGCTGCGAT	-15	GGCATGATCC	-142	1,1	6.82	0.90	(w)-sba	9583	Rmet_6764		*0*	Op1716f_1	NA
3607	TSS_2251114-3	40	2	-24	TTGCTGTCCA	0	TTCAAACTAG	-145	1,1	5.99	-2.80	no	363	Rmet_5506		*0*	Op1639r_1	NA
3608	TSS_2315557-3	40	10	-37	TTGACAAACC	-14	ACCACAATGG	-141	1,1	9.29	12.40	s	306	Rmet_6747		*0*	Op1651r_1	NA
3609	TSS_218404+4	40	9	-39	TCGCCGATCG	-14	GTTACATTGC	-139	1,1	7.72	4.90	m	26	Rmet_6083		*0*	Op1853f_2	NA
3610	TSS_400427+2	39	2	-38	TGGCCATTCT	-14	GGTATGCTGA	-139	1,1	8.45	7.90	s	1648	Rmet_0376		*0*	Op0103f_5	NA
3611	TSS_1514255+2	39	22	-40	TTGCCGGGGC	-15	ATGAAAATGG	-139	1,1	8.02	5.40	(m)-sba	32	Rmet_1403	*dsbB*		Op0395f_1	GIP-PTL-Fold
3612	TSS_1986782+2	39	9	-49	GCGGCCGGGT	-14	GCGATCATCT	-140	5,1	9.02	-18.10	no	350	Rmet_1832		*0*	Op0531f_1	NA
3613	TSS_2009589+2	39	6	-35	GGAACAGCGG	-6	TAGACCATGT	-142	4,1	5.90	0.20	no	2360	Rmet_1856	*lysP*		Op0537f_1	EIP-TRA-Ion
3614	TSS_3486948+2	39	14	-40	TAGCGAATGG	-14	GCCAAACTGA	-143	1,1	4.02	1.90	(w)-sba	145	Rmet_6585		*0*	Op2051f_1	NA
3615	TSS_3500363+2	39	20	-40	TGGCATCCAG	-15	ACCATACTGT	-143	1,1	5.86	2.90	(w)-sba	511	Rmet_3225		*0*	Op0913f_1	NA
3616	TSS_507539-2	39	11	-35	TGGTGAGCGT	-15	AGTAAAATCT	-142	1,1	8.05	10.10	s	408	Rmet_0478		*0*	Op0124r_1	NA
3617	TSS_763159-2	39	5	-49	TTGAACGCAC	-28	TTCATCTTGG	-141	1,1	5.43	-2.20	no	1040	Rmet_0689		*0*	Op0194r_1	NA
3618	TSS_3315055-2	39	7	-37	TCGAAGCCAG	-13	TGTAATCTGG	-141	1,1	5.82	6.90	s	136	Rmet_3056		*0*	Op0860r_2	GIP-TL-Ass
3619	TSS_799373+3	39	8	-40	TCAGCAACCT	-14	TGCACAATAT	-141	5,1	8.74	2.40	(w)-sba	12	Rmet_4215		*0*	Op1194f_2	NA
3620	TSS_970743+3	39	11	-39	TTGTACGTCA	-14	GCTATTTTTT	-143	1,1	6.55	5.40	m	991	Rmet_4373		*0*	Op1248f_1	NA
3621	TSS_133616-3	39	8	-31	TCGGCAAGCC	-5	GAAACGATCA	-142	5,1	7.29	2.70	(w)-ba	737	Rmet_5929		*0*	Op1801r_2	NA
3622	TSS_2253727-3	39	8	-34	TTGAACCTAT	-13	GCTACGATGG	-141	1,1	10.09	10.60	s	1757	Rmet_5507		*0*	Op1639r_1	MET-CAH-Prop
3623	TSS_117222+4	39	6	-34	GCAAGCGAGC	-14	CCTAAACTGG	-138	4,1	9.99	3.60	m	35	Rmet_5944	*pbrUa*		Op1807f_2	NA
3624	TSS_27171-4	39	10	-34	TGGCAAGGGT	-14	GGTATGTTGG	-139	1,1	7.82	8.10	s	352	Rmet_6038		*0*	Op1838r_1	NA
3625	TSS_142983-5	39	5	-35	GGAAACGTCT	-12	GGTCCAATCC	-144	4,1	5.30	4.20	m	4508	Rmet_6282	*tnpA*		Op1915r_1	NA
3626	TSS_807200+2	39	2	-35	TTGCACGGCC	-15	AGTATCATGC	-136	1,1	9.31	10.60	s	1516	Rmet_0731	*cysM*		Op0205f_4	NA
3627	TSS_1296138+2	39	14	-41	TGACGGATCT	-14	CGGATAATTC	-141	4,1	6.32	-0.60	no	2005	Rmet_1185		*0*	Op0343f_1	NA
3628	TSS_1953135+2	39	5	-42	TTGATTTTGT	-15	GCTAGCTTTC	-145	1,1	6.96	0.40	(w)-sba	38	Rmet_1801	*bug*		Op0523f_1	NA
3629	TSS_2292708+2	39	2	-48	TTGCGTTTTC	-19	GGGATATTGT	-142	1,1	7.27	-7.60	no	93	Rmet_2111		*0*	Op0609f_1	NA
3630	TSS_2766884+2	39	11	-48	GGAGAAAACA	-15	GCGAAGATAC	-142	4,1	5.95	-17.60	no	31	Rmet_2541	*ydcO*		Op0701f_3	EIP-TRA-Ion
3631	TSS_3334261+2	39	9	-36	TTCAACATCT	-14	ATTACGATCG	-142	1,1	7.82	10.90	s	135	Rmet_3078	*ybiX*		Op0863f_3	NA
3632	TSS_3682559+2	39	6	-45	TGCCGCGCCT	-15	CGCAAGATCG	-143	1,1	3.79	-9.60	no	36	Rmet_3404	*tag*		Op0945f_2	GIP-REP-Recomb
3633	TSS_3835890+2	39	9	-32	TTCATCCCTG	-7	TAAACGCTTG	-147	1,1	2.11	2.70	(w)-ba	189	Rmet_6601		*0*	Op0979f_1	NA
3634	TSS_2190039-2	39	5	-45	TTGCCATTGC	-11	TCTATTCTGA	-144	1,1	8.68	-7.60	no	81	Rmet_2024	*bipA*		Op0592r_1	NA
3635	TSS_2264448-2	39	7	-49	TTTGGTCCCG	-25	GTTAACGTGC	-143	2,1	9.40	-6.10	no	30	Rmet_2085		*0*	Op0602r_1	NA
3636	TSS_2389613-2	39	11	-35	TTGCAGGACT	-8	CATACGATTA	-141	1,1	8.03	6.20	(m)-ba	18	Rmet_2181	*phoU*		Op0628r_4	EIP-TRA-ABC
3637	TSS_3464124-2	39	3	-43	TGGCGTCCCG	-14	GCTACAATCG	-135	1,1	8.82	-3.10	no	0	Rmet_3188		*0*	Op0896r_1	NA
3638	TSS_3566771-2	39	14	-49	TCGCTGACGG	-24	ACTACAATCG	-141	1,1	7.89	-5.10	no	42	Rmet_3289	*cutA*		Op0926r_2	NA
3639	TSS_3843328-2	39	8	-32	TGGACGAAGG	0	CGTACAGTAT	-143	1,1	6.72	-1.30	no	2507	Rmet_3542		*0*	Op0982r_1	NA
3640	TSS_3870655-2	39	6	-39	TATCGGCGGC	-13	GCGATGATGA	-141	2,1	8.70	1.40	w	3104	Rmet_3565		*0*	Op0988r_1	NA
3641	TSS_153524+3	39	12	-34	TTGCCCGTAA	-10	ACCAGTGTGT	-147	1,1	3.77	7.20	s	104	Rmet_R0074		*0*	Op1006f_1	NA
3642	TSS_575914+3	39	9	-36	TTGCATTGTT	-14	CTCAAATTGG	-145	1,1	6.48	10.40	s	198	Rmet_4004	*tnpA*		Op1124f_1	NA
3643	TSS_944083+3	39	11	-47	CCTGCAATAT	-14	GTTACACTTC	-139	5,1	11.17	-14.60	no	1109	Rmet_4349		*0*	Op1238f_1	NA
3644	TSS_1758549+3	39	5	-22	TTGCGGCTGA	0	GTCAGAATCG	-141	1,1	8.34	-1.80	no	151	Rmet_5070		*0*	Op1492f_2	NA
3645	TSS_2321495+3	39	4	-43	TTCTAGTTAC	-14	GCGATATTCG	-147	1,1	5.40	-4.10	no	1725	Rmet_5569		*0*	Op1656f_1	NA
3646	TSS_2357565+3	39	17	-28	TCACAAGAAA	0	GCGCCCATTG	-149	4,1	1.02	-6.80	no	29	Rmet_5603		*0*	Op1670f_1	NA
3647	TSS_1754519-3	39	3	-43	TTGCGGGCGT	-13	TCTATGCTTG	-139	1,1	8.20	-3.60	no	5	Rmet_5064		*0*	Op1491r_2	NA
3648	TSS_1498947+2	39	6	-32	GAATTTGCCG	-12	TGTCCAATGG	-139	3,0	8.16	-0.40	no	389	Rmet_1390	*aceB*		Op0387f_2	MET-CAH-Pyr
3649	TSS_2641280+2	39	5	-36	TTGCCAACGA	-1	CGTACCCTTG	-142	1,1	8.07	-0.60	no	1834	Rmet_6526		*0*	Op0681f_1	NA
3650	TSS_3496858+2	39	9	-48	GCAGCAGCCC	-14	GACAACCTGT	-142	5,1	6.72	-18.60	no	1982	Rmet_3222		*0*	Op0911f_1	NA
3651	TSS_551629-2	39	9	-42	GGCGAAACAG	-14	ATTATGCTTC	-142	4,1	7.86	-5.60	no	1581	Rmet_0513	*mdtB*		Op0138r_1	EIP-TRA-Ion

3652	TSS_1903079-2	39	12	-35	TTCACATTGC	-14	TGAATAATTT	-143	1,1	7.73	13.10	s	2455	Rmet_1750	*agrA*	Op0498r_1	NA
3653	TSS_2286208-2	39	11	-46	GCAGCACCCG	-13	GTTATATTCA	-141	5,1	9.45	-12.60	*no*	20	Rmet_2104	*0*	Op0608r_1	NA
3654	TSS_3586977-2	39	12	-36	TGGATACCGC	-13	GCTATCGTCA	-140	1,1	5.83	10.90	s	1623	Rmet_3321	*0*	Op0926r_1	MET-XEN-Hex
3655	TSS_215227+3	39	5	-40	TTGAAAGTAA	-14	GAGATAATTC	-144	1,1	9.63	6.40	*(s)-sba*	165	Rmet_3678	*0*	Op1026f_1	DIV-MOT-Chemotax
3656	TSS_1412241+3	39	10	-37	TCGCCACATT	-14	AATACCATGG	-140	1,1	7.21	9.90	s	3320	Rmet_4771	*0*	Op1388f_1	NA
3657	TSS_2232813+3	39	15	-36	TTGCCCTCCG	-15	GAAAGACTCA	-142	1,1	5.42	10.80	s	190	Rmet_6733	*0*	Op2097f_1	NA
3658	TSS_2525860+3	39	2	-47	TCGCCGCGCG	-16	GGGAACATCC	-143	1,1	5.38	-10.10	*no*	982	Rmet_5768	*0*	Op1732f_1	MET-LIP-FASyn
3659	TSS_2233689-3	39	17	-43	TCGAATATCA	-13	ATTATTATAA	-148	1,1	6.02	-3.10	*no*	1168	Rmet_5488	*0*	Op1629r_1	GIP-REP-Recomb
3660	TSS_2395557-3	39	7	-35	TCGCCGGCAG	-14	GCTATCCTCG	-138	1,1	6.91	10.10	s	22	Rmet_5635	*0*	Op1681r_3	Met-SecMetab
3661	TSS_110503+5	39	6	-45	TTCAGCCAAC	-14	GATATCGTAC	-141	1,1	5.54	-7.10	*no*	266	Rmet_6258	*rhsC*	Op1906f_1	NA
3662	TSS_128752+2	38	13	-46	TGGAAGCGGC	-14	GCTATGATCC	-137	1,1	9.17	-8.10	*no*	6222	Rmet_0129	*dehH2*	Op0033f_1	MET-XEN-Hex
3663	TSS_326568+2	38	7	-47	GCATCAAGAT	-15	TGTATCGTTG	-144	5,1	6.64	-13.60	*no*	100	Rmet_0311	*apt*	Op0089f_1	MET-NUC-Pur
3664	TSS_735584+2	38	13	-42	TCGCGTGCCG	-14	GCTAACATGC	-141	1,1	7.08	-2.10	*no*	81	Rmet_0668	*rubA*	Op0187f_1	NA
3665	TSS_1737055+2	38	5	-42	TGGACCTACC	-17	CGGATAATTT	-143	1,1	7.68	3.90	*(m)-sba*	43	Rmet_1610	*dapE*	Op0449f_1	MET-AA-Urea
3666	TSS_3581327+2	38	3	-35	TCCCCGCTAC	-8	CTTACACTAT	-143	1,1	5.44	4.20	*(m)-ba*	4346	Rmet_3322	*0*	Op0927f_1	NA
3667	TSS_987576-2	38	13	-49	GCAAAAGCGC	-13	GGTACGATCC	-137	4,1	11.92	-19.60	*no*	4429	Rmet_0895	*0*	Op0260r_1	EIP-TRA-ABC
3668	TSS_1667311-2	38	4	-42	TCAAAACCAA	-13	GGCAAAGTCG	-145	4,1	5.77	-3.60	*no*	2384	Rmet_1543	*cbbA (de*	Op0428r_1	NA
3669	TSS_2973885-2	38	10	-33	TTGTAATTCT	-13	CTTATTGTTG	-147	1,1	6.46	8.60	s	89	Rmet_2737	*0*	Op0772r_2	NA
3670	TSS_150451+3	38	8	-36	TTGCCAACAG	-6	TTAACAATAT	-145	1,1	6.98	5.40	*(m)-ba*	180	Rmet_R0073	*0*	Op1006f_1	NA
3671	TSS_1296061-3	38	4	-39	TTCCCGCGAC	-13	TTCATCATGG	-143	1,1	6.35	3.90	m	732	Rmet_4661	*rpoD2*	Op1349r_1	GIP-TK-RNAP
3672	TSS_139333+4	38	7	-38	TCGTCTTCTC	-15	ACTACACTAA	-145	1,1	5.52	7.90	s	719	Rmet_6377	*0*	Op2113f_1	NA
3673	TSS_27539-4	38	11	-40	GTACAGGAGT	-14	GATATGATCG	-140	4,1	9.62	-0.10	*no*	278	Rmet_6358	*0*	Op1838r_1	NA
3674	TSS_345090+2	38	11	-33	TTGCGGTCCC	-13	AGTAGACTAT	-142	1,1	7.65	7.60	s	10759	Rmet_0338	*0*	Op0095f_1	NA
3675	TSS_2369948+2	38	10	-34	TTGCTGAAGC	-14	TCTATGATGG	-140	1,1	9.50	9.60	s	886	Rmet_2168	*0*	Op0623f_1	NA
3676	TSS_2529191+2	38	6	-28	TGGCGGGGAT	-8	ATTATTCTGT	-143	1,1	6.22	1.10	*(w)-nu*	13	Rmet_2309	*0*	Op0659f_2	NA
3677	TSS_3094477+2	38	2	-37	GCTGCAGGCC	-14	GTTACCTTAA	-140	5,1	7.85	4.40	m	137	Rmet_2845	*0*	Op0801f_2	NA
3678	TSS_492377-2	38	12	-34	TGGCAATGCC	-13	CCCACAATGC	-138	1,1	8.06	9.10	s	1812	Rmet_R0005	*0*	Op0122r_2	NA
3679	TSS_1108615-2	38	8	-35	TCGAAGCTGC	-13	GGTCAACTAC	-142	1,1	3.22	8.70	s	998	Rmet_1017	*phaZ1*	Op0294r_1	NA
3680	TSS_2377889-2	38	8	-40	GCGGACATAC	-13	TTTAATATCA	-145	5,1	8.35	-2.10	*no*	118	Rmet_2172	*int*	Op0624r_1	NA
3681	TSS_2428113-2	38	18	-40	TGCCGAGCGG	-13	CCTATACTTG	-138	1,1	6.96	1.40	*(w)-sba*	101	Rmet_2214	*0*	Op0634r_2	NA
3682	TSS_2901529-2	38	9	-41	TTGCAGCTAG	-13	TGCATGATAG	-144	1,1	7.13	0.40	*(w)-sba*	702	Rmet_2666	*0*	Op0756r_5	NA
3683	TSS_3092502-2	38	5	-42	TGCACAGCAC	-13	GATATAATGG	-139	1,1	9.40	0.40	*(w)-sba*	1986	Rmet_2842	*tnpA*	Op2044r_1	NA
3684	TSS_3631163-2	38	8	-34	TTCCATCGCT	-14	GTCAATCTCG	-145	1,1	4.42	5.10	m	629	Rmet_3353	*tnpA*	Op0934r_1	NA
3685	TSS_390670+3	38	9	-38	TGGCATCAAT	-15	CTCATACTGT	-141	1,1	6.36	6.90	s	66	Rmet_3848	*0*	Op1070f_1	MET-COF-CoQ
3686	TSS_2526798+3	38	20	-42	GGACGACAGA	-19	GAGATGATAG	-144	4,1	5.51	-0.60	*no*	44	Rmet_5768	*0*	Op1732f_1	MET-LIP-FASyn
3687	TSS_1208036-3	38	16	-44	CCAGCCGGAT	-13	GCTATGGTGG	-139	5,1	9.14	-10.60	*no*	2517	Rmet_4586	*0*	Op1319r_1	NA
3688	TSS_1291470-3	38	8	-36	TCGCAAAGTA	-13	TTTAAAATAT	-141	1,1	8.49	10.90	s	319	Rmet_4657	*0*	Op1347r_1	NA
3689	TSS_1763182-3	38	9	-34	TTGATGAAAA	-13	CGGACAATAC	-146	1,1	7.35	10.60	s	938	Rmet_5073	*metE*	Op1493r_1	NA
3690	TSS_2514436-3	38	3	-48	TTGAGGCATG	-13	GATACAGTCG	-137	1,1	8.17	-12.60	*no*	163	Rmet_5752	*0*	Op1723r_1	NA
3691	TSS_66551+4	38	8	-49	TCCCCAAGGG	-15	GGTATGCTTA	-141	1,1	6.56	-14.60	*no*	5877	Rmet_6365	*0*	Op2109f_1	NA
3692	TSS_110488-4	38	12	-45	GTCACAGCGC	-14	TGTACGATGT	-138	4,1	9.09	-8.10	*no*	1248	Rmet_5951	*tnpA*	Op1810r_1	NA
3693	TSS_923432+2	38	2	-36	TGGCGCTGGT	-15	GTCAGACTAC	-140	1,1	6.22	8.30	s	732	Rmet_0844	*0*	Op0241f_2	NA
3694	TSS_2714071+2	38	6	-35	TTGAACTTCA	-14	AGTTAAATGA	-147	1,1	3.84	10.60	s	352	Rmet_2501	*gstF*	Op0693f_1	MET-OAA-GSH
3695	TSS_3094600+2	38	14	-49	TTGAACTGGA	-14	TCCATAATGA	-141	1,1	9.45	-12.60	*no*	14	Rmet_2845	*0*	Op0801f_2	NA
3696	TSS_929971-2	38	23	-43	TCCATCATGA	-12	TGTATAATGC	-140	1,1	8.36	-4.60	*no*	51	Rmet_R0008	*0*	Op0242r_1	NA
3697	TSS_3429716-2	38	9	-42	TGCAATCACG	-15	GCTAACGTCC	-145	1,1	4.18	-2.60	*no*	2030	Rmet_R0050	*0*	Op0886r_1	NA
3698	TSS_3624688-2	38	2	-28	TTGACATTGC	-8	CCCACTCTTG	-143	1,1	6.49	3.60	*(m)-nu*	1027	Rmet_3346	*uspA8*	Op0932r_3	NA
3699	TSS_1207954+3	38	9	-36	TTGCGCGTCG	-15	CTGATACTGG	-139	1,1	6.78	10.80	s	28	Rmet_4589	*amB*	Op1320f_2	NA
3700	TSS_2575141+3	38	5	-36	TTGTGCTTGT	-15	TATAGCGTCA	-141	1,1	4.72	9.80	s	4185	Rmet_5816	*cspA*	Op1756f_1	NA
3701	TSS_2553811-3	38	2	-45	GCTGCCGGCC	-13	GGTACGCTTC	-137	5,1	10.04	-13.60	*no*	3367	Rmet_5793	*cyoA*	Op1743r_1	MET-EN-OxPhos
3702	TSS_449856+2	37	5	-47	TCGCCAGTCC	-14	GATAAGCTGG	-140	1,1	6.95	-11.10	*no*	10914	Rmet_0436	*ostA*	Op0117f_1	GIP-PTL-Fold
3703	TSS_1012701+2	37	11	-29	GTACGAGTGC	-6	TCGAGGATGC	-142	4,1	6.58	-0.30	*no*	211	Rmet_0928	*nuoB*	Op0267f_3	MET-EN-OxPhos
3704	TSS_2405754+2	37	3	-44	GCAAGGGTAT	-14	GCCAGAATCG	-142	4,1	8.85	-9.60	*no*	14277	Rmet_2207	*0*	Op0631f_1	NA
3705	TSS_3273628+2	37	9	-49	TTGACGATGG	-8	TCTACATTGA	-142	1,1	8.67	-18.60	*no*	10998	Rmet_3028	*0*	Op0849f_1	NA
3706	TSS_3867012+2	37	10	-45	GCGGCCGGGT	-14	TGTAACCTTA	-142	5,1	8.91	-11.10	*no*	802	Rmet_3566	*0*	Op0989f_1	NA
3707	TSS_1365624-2	37	7	-35	TTGACCTCCA	-12	TAGATGAACC	-146	1,1	1.53	10.20	s	10101	Rmet_1232	*yhgF*	Op0356r_1	NA
3708	TSS_1461072-2	37	18	-36	GCCGGCCAAC	-14	TATATCATGG	-139	4,1	8.29	6.40	s	12790	Rmet_1338	*0*	Op0378r_1	NA
3709	TSS_1696906-2	37	7	-45	TTTGGGAGGG	-19	GGTACATAAC	-148	2,1	3.95	-5.10	*no*	25	Rmet_1576	*0*	Op0434r_1	NA
3710	TSS_3422210-2	37	1	-46	GGAAGCCACG	-15	GGCATCATTC	-143	4,1	6.39	-12.60	*no*	258	Rmet_3157	*dppB*	Op0884r_1	EIP-TRA-ABC
3711	TSS_3568260-2	37	12	-35	TGGAGCCGTT	-9	CATACGCTCG	-141	1,1	5.77	5.70	*(m)-ba*	1023	Rmet_3290	*rplQ*	Op0926r_2	GIP-TL-Ribo
3712	TSS_1049516-3	37	6	-48	TTGCGTGGAT	-13	CGTAGACTTG	-137	1,1	8.53	-13.60	*no*	22	Rmet_6666	*0*	Op1267r_1	NA

3713	TSS_2102509-3	37	13	-34	TTGCGCTCGC	-13	ACGATCATCC	-137	1,1	6.60	9.60	s	5393	Rmet_5371	*katG*	Op1599r_1	MET-EN-Methane
3714	TSS_74141+4	37	9	-46	CGGCAATAAA	-15	CTTAGAATGC	-146	1,1	4.53	-10.10	*no*	749	Rmet_6368	*0*	Op1821f_1	NA
3715	TSS_2359057+2	37	6	-46	TGGAGCCCTG	-13	CTTACACTAG	-143	1,1	6.55	-10.10	*no*	565	Rmet_2160	*0*	Op0621f_3	NA
3716	TSS_3639070+2	37	5	-39	TCGACCGCAG	-16	TGTACAGTTT	-141	1,1	6.66	7.90	s	6727	Rmet_3369	*paaA*	Op0937f_1	NA
3717	TSS_214119-2	37	4	-40	GCTGCAACGC	-13	GCTACACTGT	-140	5,1	10.99	-1.60	*no*	24	Rmet_0204	*0*	Op0060r_1	NA
3718	TSS_1053005-2	37	9	-40	TTCATTTTTC	-14	GCTAGAGTAA	-147	1,1	5.80	2.90	*(w)-sba*	51	Rmet_0968	*0*	Op0270r_1	MET-CAH-Prop
3719	TSS_1600180-2	37	11	-44	TTGTCAGCGA	-5	TCCAGAATAG	-143	1,1	7.09	-11.60	*no*	3740	Rmet_1476	*0*	Op0414r_1	NA
3720	TSS_1762071-2	37	7	-38	TTGCCGGACT	-13	ACGACAATAT	-139	1,1	7.30	7.40	s	353	Rmet_1632	*garR*	Op0458r_1	MET-CAH-DiCarb
3721	TSS_1804890-2	37	5	-43	TCGCTACGCC	-13	CAGATACTTT	-143	1,1	4.89	-4.10	*no*	971	Rmet_1667	*fimA*	Op0468r_1	NA
3722	TSS_1977980-2	37	9	-48	TCGAACGCCA	-14	AGTAGACTTG	-140	1,1	6.74	-13.10	*no*	26	Rmet_1821	*ybdG*	Op0528r_1	NA
3723	TSS_3428931-2	37	6	-49	TTGACTGGCC	-14	AATAAAATGG	-140	1,1	9.79	-11.60	*no*	1245	Rmet_R0050	*0*	Op0886r_1	NA
3724	TSS_3680420-2	37	13	-40	TGGCCTGCGT	-13	GCCAGAGTTG	-143	1,1	6.46	0.90	*(w)-sba*	31	Rmet_3401	*0*	Op0944r_1	NA
3725	TSS_153677+3	37	9	-29	TGGAACCACT	-3	AGGACCGTGA	-144	1,1	2.68	-1.30	*no*	204	Rmet_3617	*0*	Op1006f_2	GIP-REP-Recomb
3726	TSS_1056785+3	37	4	-35	TTGCGGGCAG	-14	GTCAGAATGC	-139	1,1	8.42	10.60	s	2752	Rmet_4462	*hns*	Op1272f_1	NA
3727	TSS_2278203+3	37	15	-44	TGGAGGAAAC	-15	CATACACTCT	-142	1,1	7.44	-4.10	*no*	853	Rmet_5532	*0*	Op1644f_1	NA
3728	TSS_742106-3	37	8	-46	TCGAAGAAGC	-14	GCTAATCTGA	-141	1,1	6.74	-10.10	*no*	13036	Rmet_4153	*tnpA*	Op1169r_1	NA
3729	TSS_1162469-3	37	1	-44	TGCCAAACAG	-13	TGTAAATTCA	-144	1,1	5.68	-7.60	*no*	1672	Rmet_4549	*uvrA2*	Op1305r_1	GIP-REP-Recomb
3730	TSS_1269929-3	37	3	-34	TTGCGATCCG	-13	GCAACCATTT	-139	1,1	6.19	9.60	s	1990	Rmet_4636	*0*	Op1339r_1	NA
3731	TSS_1398502-3	37	10	-36	CGGCGCTTTT	-13	GGCACGATAT	-145	1,1	1.48	4.90	m	914	Rmet_4752	*bcr*	Op1379r_1	NA
3732	TSS_2312378-3	37	4	-33	TGGCTCTTTC	-13	GGTAGAGTTG	-141	1,1	7.12	6.10	m	3653	Rmet_6746	*0*	Op2099r_1	NA
3733	TSS_2343197-3	37	9	-44	TGGAATACGC	-12	ACTAGAATTC	-136	1,1	8.66	-6.10	*no*	74	Rmet_5589	*0*	Op1665r_1	NA
3734	TSS_110804+4	37	4	-36	TTGACATCCT	-15	CGTCCACTCC	-143	1,1	4.90	11.80	s	3778	Rmet_5946	*pbrR*	Op1807f_1	NA
3735	TSS_116048-5	37	11	-34	TTGAGCGCCT	-14	GCTACGCTGC	-139	1,1	8.42	8.60	s	1428	Rmet_6260	*tnpA*	Op1907r_1	NA
3736	TSS_2273689+2	37	6	-46	TGGATGAGTG	-14	GGCATTATCT	-144	1,1	6.29	-9.10	*no*	105	Rmet_2093	*0*	Op0607f_2	NA
3737	TSS_2361057+2	37	9	-38	GCCAAGGACG	-14	AGTAAAGTGC	-144	4,1	7.08	2.40	w	2637	Rmet_2163	*0*	Op0623f_1	NA
3738	TSS_2372192+2	37	2	-35	TGGAACGGAC	-7	GGAACTCTTT	-145	1,1	4.03	2.70	*(w)-ba*	7684	Rmet_R0034	*0*	Op0625f_1	NA
3739	TSS_3910114+2	37	10	-35	TTGACCCACG	-14	CCTACGATGA	-141	1,1	8.34	12.60	s	71	Rmet_3598	*0*	Op1001f_1	NA
3740	TSS_1255583-2	37	3	-36	TCTGCCCGCG	-13	CGTACGATGT	-139	5,1	9.68	8.40	s	6240	Rmet_1141	*0*	Op0326r_1	NA
3741	TSS_1509150-2	37	11	-35	TCGATATTTG	-14	ACGAAAATTC	-140	1,1	6.32	11.10	s	270	Rmet_1399	*0*	Op0392r_1	NA
3742	TSS_1595067-2	37	11	-49	TGGAGATTTT	-23	GTAACGATCG	-142	1,1	5.71	-6.10	*no*	197	Rmet_1474	*0*	Op0412r_1	NA
3743	TSS_2093464-2	37	21	-40	CCAGCCGCCT	-16	ACCACCGTCC	-142	5,1	4.92	-1.60	*no*	0	Rmet_R0022	*0*	Op0562r_2	NA
3744	TSS_2681047-2	37	3	-49	TTGCGACAGA	-13	GGTATGATGC	-135	1,1	10.64	-13.60	*no*	92	Rmet_2469	*truA*	Op0688r_3	GIP-TL-Ass
3745	TSS_2781229-2	37	12	-35	CCGGCACCTC	-14	GGTAGTCTGG	-142	5,1	8.31	7.10	s	392	Rmet_2556	*tnpB*	Op0708r_3	GIP-REP-Recomb
3746	TSS_2940693-2	37	9	-26	TTGCGGCTTT	-1	CTTCATATGC	-145	1,1	3.15	-2.80	*no*	41	Rmet_2707	*0*	Op0766r_1	NA
3747	TSS_3614949-2	37	2	-42	TGCAATCACG	-15	GCTAACGTCC	-145	1,1	4.18	-2.60	*no*	2030	Rmet_R0060	*0*	Op0930r_1	NA
3748	TSS_1727064+3	37	8	-42	TGGAGCATAC	-15	AGTATACTTA	-143	1,1	8.24	0.90	*(w)-sba*	66	Rmet_5040	*0*	Op1482f_1	NA
3749	TSS_554426-3	37	14	-34	TCGTCTATTT	-14	GGCAAGCTAT	-145	1,1	3.81	6.10	m	2590	Rmet_3984	*yebQ*	Op1117r_2	NA
3750	TSS_1169737-3	37	11	-33	GCTGCAGTCG	-13	CATAGAATGC	-141	5,1	10.61	4.60	m	23	Rmet_4559	*0*	Op1309r_1	NA
3751	TSS_1290310-3	37	6	-42	TCCGCCAAGC	-13	GGTATGCTTT	-144	4,1	7.50	-3.60	*no*	3501	Rmet_4654	*0*	Op1345r_1	NA
3752	TSS_1682171-3	37	17	-40	TTGACTTCTC	-14	TGCAGTATTG	-143	1,1	6.58	4.40	*(m)-sba*	81	Rmet_5000	*0*	Op1467r_1	NA
3753	TSS_1789490-3	37	11	-38	TCGTAGCACC	-13	TATACAATAT	-142	1,1	6.91	5.90	m	99	Rmet_5097	*0*	Op1499r_1	MET-AA-Lys_Syn
3754	TSS_2355962-3	37	11	-39	TTGTCGTACA	-14	GGCAGAATCA	-140	1,1	7.81	6.40	s	39	Rmet_5601	*0*	Op1669r_1	NA
3755	TSS_170726-5	37	8	-47	TTCAAGAAAT	-13	CTGATATTCC	-144	1,1	5.49	-12.10	*no*	13367	Rmet_6301	*trbF*	Op1917r_1	NA
3756	TSS_989026+2	36	10	-49	TGGCAATCTC	-15	GACATCATCA	-144	1,1	6.50	-14.10	*no*	523	Rmet_0902	*0*	Op0261f_2	NA
3757	TSS_1230087+2	36	5	-45	TTCTGTACGT	-15	CCTAGTCTCT	-144	1,1	4.11	-8.10	*no*	2343	Rmet_1125	*fdhD*	Op0321f_2	NA
3758	TSS_1262978+2	36	10	-43	TCCCTTATGT	-14	GGCAGTATGC	-146	1,1	4.22	-6.60	*no*	149	Rmet_1153	*atoD*	Op0331f_1	MET-CAH-But
3759	TSS_2227781+2	36	18	-38	TCGCCCTTGC	-16	GATACAATTC	-138	1,1	9.94	9.90	s	924	Rmet_2057	*0*	Op0597f_1	NA
3760	TSS_2420695+2	36	8	-36	TGGCCGATCT	-15	TATATGATCT	-136	1,1	8.05	11.30	s	149	Rmet_2208	*mhA*	Op0631f_1	GIP-REP-Recomb
3761	TSS_2685366+2	36	13	-34	TTCCTCGCCA	-14	CCTACAATCA	-141	1,1	7.35	8.10	s	16374	Rmet_2489	*mdh*	Op0689f_1	MET-CAH-TCA
3762	TSS_2703970+2	36	4	-35	TCGCCCCGTG	-15	TAGAGAATAG	-143	1,1	5.07	9.10	s	94	Rmet_2491	*0*	Op0689f_2	NA
3763	TSS_3037628+2	36	23	-47	TTCAACTTTG	-22	GTCAAGCTGC	-142	1,1	5.56	-5.10	*no*	151	Rmet_2790	*0*	Op0785f_1	NA
3764	TSS_1020108-2	36	11	-39	GCCGCGGCAC	-13	CGCATACTTC	-140	4,1	7.53	-0.60	*no*	15038	Rmet_0919	*0*	Op0264r_1	NA
3765	TSS_2505810-2	36	2	-42	TACCCGTTTT	-14	GATACCCTAC	-150	1,1	2.51	-3.60	*no*	24	Rmet_2283	*0*	Op0652r_1	NA
3766	TSS_2939812-2	36	2	-36	TGGCCGGCAC	-13	GGTACAATCA	-136	1,1	9.52	10.90	s	125	Rmet_2705	*bacA*	Op0766r_2	MET-PepGlyc
3767	TSS_3093790-2	36	5	-21	TTCCCCATAT	0	GGTAAATTCC	-141	1,1	8.37	-3.90	*no*	729	Rmet_6563	*0*	Op2045r_1	NA
3768	TSS_3265742-2	36	7	-41	TCGAGGCGCT	-21	GAAATCCTGT	-142	1,1	3.68	3.30	*m*	736	Rmet_3008	*tniQ*	Op0846r_1	NA
3769	TSS_372+3	36	11	-45	GCAGAAACAG	-13	CGCAGAATCG	-141	4,1	9.07	-12.60	*no*	461	Rmet_6605	*0*	Op1756f_1	NA
3770	TSS_2043372+3	36	4	-33	TTCTTCAGGC	-12	CGTAACATGC	-141	1,1	6.50	7.10	s	44	Rmet_5331	*0*	Op1578f_1	EIP-SIG-2Comp
3771	TSS_311414-3	36	10	-36	TAGACATTAA	-13	TGTATGCTCG	-143	1,1	4.99	11.90	s	4917	Rmet_3769	*alkB1*	Op1047r_1	NA
3772	TSS_610898-3	36	3	-41	TCGAGGCGCT	-21	GAAATCCTGT	-142	1,1	3.68	3.30	*m*	736	Rmet_4043	*tniQ*	Op1139r_1	NA
3773	TSS_1676657-3	36	7	-34	TTGTCTACTA	-13	ACGATTATCC	-144	1,1	5.35	10.60	s	263	Rmet_4996	*0*	Op1467r_2	NA

3774	TSS_17061+4	36	5	-49	TCGCCCGCGC	-14	AGTATCCTGG	-139	1,1	6.53	-15.10	no	1655	Rmet_6047	0	Op1843f_1	NA
3775	TSS_80690+4	36	4	-44	TCGATCGGGA	-15	CTTAACATGA	-141	1,1	6.01	-4.10	no	1003	Rmet_5979	czcD	Op1819f_1	EIP-TRA-Ion
3776	TSS_197027+4	36	3	-48	GTGAGAACAC	-28	GGCATAATCC	-143	4,1	4.40	-2.20	no	426	Rmet_6103	0	Op1863f_1	NA
3777	TSS_15179-4	36	5	-47	TGCTACGCAA	-24	GGCAATCTGT	-147	1,1	2.63	-5.60	no	891	Rmet_6053	0	Op1844r_3	NA
3778	TSS_111006-4	36	10	-32	AAATTCCACG	-12	TACTATTCCT	-143	3,0	6.11	-5.40	no	150	Rmet_5949	pbrD	Op1808r_2	NA
3779	TSS_115291+5	36	7	-36	TCGCCAAACA	-15	CTTACGATGC	-141	1,1	7.70	11.30	s	967	Rmet_6395	0	Op1908f_2	NA
3780	TSS_866833+2	36	12	-36	TGGTGCTTGG	-15	CGTATGGTGT	-139	1,1	5.08	9.30	s	485	Rmet_0791	0	Op0225f_2	MET-LIP-FASyn
3781	TSS_1061158+2	36	6	-42	TTGCTCATCG	-13	GTTAAATTAG	-142	1,1	7.70	-1.60	no	141	Rmet_0975	gst	Op0273f_1	MET-OAA-GSH
3782	TSS_1320726+2	36	6	-33	TGCCGCAGCG	-13	GCTACCATCG	-138	1,1	6.54	4.60	m	15	Rmet_1201	0	Op0349f_1	NA
3783	TSS_2166578+2	36	8	-40	TGGCCGCTCA	-15	GGTAATCTTG	-144	1,1	6.77	2.90	(w)-sba	927	Rmet_1997	0	Op0585f_1	NA
3784	TSS_2981961+2	36	7	-41	CCGGAAACGC	-14	CGTAACCTTG	-142	5,1	8.08	-3.10	no	5015	Rmet_2750	ppc	Op0775f_1	MET-CAH-Pyr
3785	TSS_3518387+2	36	10	-36	TTGATGATGG	-15	GCGAATATTG	-140	1,1	6.71	10.80	s	12982	Rmet_3261	0	Op0919f_1	NA
3786	TSS_27111-2	36	13	-37	TGGATCATGT	-13	GCGAAACTGA	-142	1,1	6.19	6.90	s	209	Rmet_0022	0	Op0004r_1	NA
3787	TSS_399866-2	36	6	-39	TGGTGGGCCA	-15	GGCAACATCT	-140	1,1	4.64	3.90	m	12021	Rmet_0365	coxS	Op0102r_1	MET-EN-Methane
3788	TSS_2122206-2	36	2	-35	TTGCCTTCCA	-14	GCAATCGTGC	-140	1,1	4.64	10.60	s	2477	Rmet_6494	0	Op2020r_1	NA
3789	TSS_2829011-2	36	9	-40	TCAAACACGG	-13	CGTAGACTGT	-144	4,1	8.09	0.40	(w)-sba	44	Rmet_2600	0	Op0728r_1	NA
3790	TSS_2847465-2	36	6	-47	GCGGCCGTGG	-14	GATATTCTCG	-140	5,1	9.04	-14.10	no	251	Rmet_2615	dxs	Op0732r_2	MET-LIP-Ster
3791	TSS_2848017-2	36	5	-40	TTCCATACAC	-13	GCTATGCTGT	-138	1,1	7.54	0.90	(w)-sba	803	Rmet_2615	dxs	Op0732r_2	MET-LIP-Ster
3792	TSS_3094454-2	36	8	-36	TAGAAAAACG	-13	GGTAACCTGT	-143	1,1	4.67	9.90	s	1393	Rmet_6563	0	Op2045r_1	NA
3793	TSS_3408471-2	36	20	-42	GCGACAGCGC	-13	GGCAAAGTCT	-140	5,1	5.81	-4.10	no	1260	Rmet_3143	tnmB	Op0880r_1	NA
3794	TSS_3431235-2	36	14	-22	TTCATGACTG	0	AACAAGGTAG	-147	1,1	1.60	-4.30	no	106	Rmet_R0053	0	Op0886r_1	NA
3795	TSS_3806979-2	36	7	-48	TTGTTTCATA	-27	GTTACATTCC	-143	1,1	7.09	-1.20	no	218	Rmet_3515	0	Op0974r_5	NA
3796	TSS_3923923-2	36	12	-34	TGGACATCTG	-13	ACCAACATCC	-140	1,1	5.76	10.10	s	301	Rmet_3611	0	Op1004r_1	NA
3797	TSS_475001+3	36	8	-36	TTGCCAACAG	-6	TTAACAATAT	-145	1,1	6.98	5.40	(m)-ba	180	Rmet_R0078	0	Op1098f_1	NA
3798	TSS_1181121+3	36	17	-39	TTGCTGCTGC	-17	GAGATTATGC	-142	1,1	6.93	8.40	s	7534	Rmet_4574	0	Op1316f_1	MET-CAH-Prop
3799	TSS_1188028-3	36	4	-35	CCGGCAATTT	-13	CATAAGCTAG	-146	5,1	7.25	7.70	s	136	Rmet_4573	0	Op1315r_1	NA
3800	TSS_17587+5	36	12	-41	TGGCGGTAGG	-14	TGGACAATTG	-143	1,1	7.18	-0.10	no	286	Rmet_6328	0	Op1922f_1	NA
3801	TSS_3336083+2	36	14	-34	TTCCCGATAT	-14	CCTACGATGG	-141	1,1	8.09	8.10	s	57	Rmet_3080	0	Op0865f_1	NA
3802	TSS_91143-2	36	9	-49	TTGCGACGCG	-14	GTCAGTCTCC	-142	1,1	5.48	-15.60	no	1995	Rmet_0081	mhpD2	Op0022r_1	NA
3803	TSS_1061270-2	36	6	-36	TTCAGCATGT	-13	AGTAGTGTGA	-144	1,1	4.54	8.90	s	147	Rmet_0974	paaH	Op0272r_1	MET-CAH-But
3804	TSS_3495554-2	36	2	-36	TCGACCGACG	-13	CGCATACTGC	-143	1,1	6.58	10.90	s	16	Rmet_3219	0	Op0908r_2	NA
3805	TSS_155843+3	36	11	-46	TGGACTAGGC	-12	GGTCTGATCC	-142	1,1	4.43	-11.10	no	1295	Rmet_3621	0	Op1008f_2	NA
3806	TSS_3046-3	36	8	-36	TCAGCAAGGC	-14	TGCAGAATGG	-137	5,1	9.37	10.40	s	700	Rmet_5818	csp	Op1757r_1	NA
3807	TSS_408671-3	36	11	-43	TGGCGATCTA	-14	CGTATCCTGG	-137	1,1	6.98	-3.10	no	931	Rmet_3864	chrF2	Op1075r_2	NA
3808	TSS_832914-3	36	2	-49	TGGCAGCGAA	-14	AGTACATTGG	-143	1,1	7.01	-16.10	no	4275	Rmet_4240	acnA3	Op1201r_1	NA
3809	TSS_1087736-3	36	4	-46	TCGTAGATAC	-12	CCTAGAATAA	-143	1,1	6.69	-11.10	no	400	Rmet_4487	0	Op1279r_2	NA
3810	TSS_1889702-3	36	7	-35	GTAGACGAGT	-13	ATGATTATTT	-149	4,1	5.94	6.70	s	38	Rmet_5194	0	Op1531r_1	NA
3811	TSS_1919171-3	36	9	-38	TGGCACCTCG	-12	TTTATGATAT	-139	1,1	7.08	4.90	m	2728	Rmet_6715	0	Op2087r_1	NA
3812	TSS_1950925-3	36	23	-39	TTGCGCTTAA	0	CGTGCGATGC	-146	1,1	2.43	-10.60	no	70	Rmet_5250	0	Op1549r_1	DIV-MOT-Chemotax
3813	TSS_624371+2	35	4	-49	TGGCGGGCCA	-14	GATAGCATAG	-143	1,1	6.10	-16.10	no	1211	Rmet_0584	0	Op0165f_1	NA
3814	TSS_1032292+2	35	5	-30	GGAGACAAGC	-6	CGCATCGTTG	-144	4,1	6.01	-2.80	no	1058	Rmet_0948	cyoA	Op0267f_4	MET-EN-OxPhos
3815	TSS_1795610+2	35	6	-44	TCGCTACGCC	-14	CAGATACTTT	-144	1,1	4.89	-5.10	no	389	Rmet_1661	0	Op0469f_1	NA
3816	TSS_2384495+2	35	9	-37	TTCTATGAAA	-14	GCCAAAATAA	-147	1,1	5.84	7.90	s	5	Rmet_2178	ppk	Op0627f_1	MET-EN-OxPhos
3817	TSS_3624874+2	35	9	-37	CCGGCAGGCT	-13	TTCAATCTGA	-143	5,1	7.13	3.90	m	3524	Rmet_3352	0	Op0933f_1	NA
3818	TSS_891566-2	35	5	-39	TTCCAGCAGC	-12	GCGACGATTG	-139	1,1	6.13	0.90	w	231	Rmet_0818	glnS	Op0230r_1	MET-AA-GluGSH
3819	TSS_1063210-2	35	4	-41	GCGGCATGGG	-13	GCTAGAATGT	-135	5,1	11.24	-1.10	no	39	Rmet_0976	ybdL	Op0274r_2	NA
3820	TSS_1789392-2	35	5	-39	TTGTCGCTAT	-14	CGTAGACTCC	-143	1,1	8.15	6.40	s	8	Rmet_1653	0	Op0466r_2	NA
3821	TSS_2489005-2	35	9	-35	GTCGGAGATT	-13	CATAACATGC	-146	4,1	5.18	7.70	s	11	Rmet_2268	tnpA	Op0648r_2	NA
3822	TSS_2961847-2	35	6	-35	CGCAGGTAAA	-13	ATTAAACTGG	-147	1,1	1.72	6.20	m	221	Rmet_2725	0	Op0770r_1	NA
3823	TSS_1536491+3	35	9	-35	TTGCGCGCGC	-14	TGCACGCTGT	-138	1,1	5.81	8.60	s	4594	Rmet_4879	catM	Op1426f_1	NA
3824	TSS_646675-3	35	6	-45	TCCAAAAATT	-14	GCGATACTCG	-145	1,1	6.10	-7.60	no	40	Rmet_4077	0	Op1145r_1	NA
3825	TSS_1268208-3	35	10	-35	TGGCGCTGAA	-14	AAGAAAATGT	-139	1,1	6.09	9.10	s	269	Rmet_4636	0	Op1339r_1	NA
3826	TSS_68365+4	35	9	-37	TTGCCGCTGG	-16	GTCATGGTCT	-141	1,1	5.76	9.80	s	4063	Rmet_6365	0	Op2109f_1	NA
3827	TSS_193249+4	35	12	-35	GTACGGGAGC	-14	GCCATGATCG	-141	4,1	8.20	6.10	m	278	Rmet_6109	copN	Op1865f_1	NA
3828	TSS_261696+2	35	3	-39	GTCCAGAACC	-8	AATATGCTGC	-141	4,1	7.16	-5.10	no	314	Rmet_0246	ptsI	Op0071f_3	EIP-TRA-Pts
3829	TSS_332361+2	35	2	-38	GTCAGCAACT	-18	CGGACGATCG	-141	4,1	5.10	3.30	m	31	Rmet_0314	0	Op0091f_1	NA
3830	TSS_415247+2	35	12	-36	TCGCCGTCGA	-14	ACGAGAATAT	-141	1,1	5.21	10.90	s	865	Rmet_0388	0	Op0105f_2	NA
3831	TSS_2004352+2	35	6	-37	TCGATCTCGC	-14	CATAAAATTG	-139	1,1	8.00	9.90	s	7597	Rmet_1856	lysP	Op0537f_1	EIP-TRA-Ion
3832	TSS_2187119+2	35	5	-32	TGGAGGAATC	-6	CGAAAAATAT	-142	1,1	5.37	3.70	(m)-ba	2959	Rmet_2025	0	Op0593f_1	NA
3833	TSS_3638624+2	35	2	-34	TGCCCAAAGC	-14	TGTAGTATCG	-141	1,1	6.49	7.60	s	7173	Rmet_3369	paaA	Op0937f_1	NA
3834	TSS_3819440+2	35	6	-40	TCAGCCCGTC	-14	GGTATTGTGC	-144	5,1	7.85	1.40	(w)-sba	11746	Rmet_3533	phhA	Op0977f_1	MET-AA-PheTyrTrp

3835	TSS_761314-2	35	4	-43	TCGATAGACG	-12	CCTACCATGA	-141	1,1	6.37	-4.10	no	4077	Rmet_0684	*trxB*	Op0192r_1	MET-NUC-Pyr	
3836	TSS_1775049-2	35	8	-29	TTGCGTCGCC	-3	CGCAACATGG	-142	2,1	9.36	0.20	no	63	Rmet_1641	0	Op0462r_3	NA	
3837	TSS_1849525-2	35	3	-42	TCGGATACCG	-13	CGTACGATCG	-141	5,1	7.60	-3.10	no	10552	Rmet_6472	0	Op2015r_1	NA	
3838	TSS_1889609-2	35	9	-36	TCGCGCAAAT	-14	GGGAAAATCG	-138	1,1	7.41	9.90	s	1638	Rmet_1740	0	Op0494r_1	NA	
3839	TSS_2046178-2	35	6	-48	TTCAAGCAGT	-22	GATAGAATCG	-141	1,1	9.29	-4.10	no	27	Rmet_1882	*ppiD*	Op0544r_2	GIP-PTL-Fold	
3840	TSS_3386015-2	35	4	-20	GAATTTGCCG	0	CTTTGACGTG	-144	3,0	8.16	-15.40	no	165	Rmet_3123	*ftsZ*	Op0878r_3	DIV-Division	
3841	TSS_3430761-2	35	16	-37	TTGTGATTGT	-12	TTTAAAGTGA	-145	1,1	7.70	8.40	s	78	Rmet_R0051	0	Op0886r_1	NA	
3842	TSS_3634034-2	35	5	-44	GCTGCCGTTT	-14	GTCAGACTTG	-141	5,1	7.77	-10.60	no	3500	Rmet_3353	*tnpA*	Op0934r_1	NA	
3843	TSS_1164144+3	35	8	-42	TTGAAATTAA	-15	GATACAATCG	-141	1,1	11.47	3.40	(m)-sba	1577	Rmet_4553	0	Op1306f_3	NA	
3844	TSS_2278111+3	35	5	-21	TTCAACCATT	0	AAGAGGATTG	-147	1,1	4.32	-4.90	no	13	Rmet_5531	0	Op1644f_1	NA	
3845	TSS_2431257+3	35	6	-47	GTAACCACCT	-25	ATTACATTCC	-143	4,1	6.87	-2.10	no	75	Rmet_5672	*copR2*	Op1696f_1	EIP-SIG-2Comp	
3846	TSS_2462760+3	35	10	-32	TCGCAGGCCA	-1	CATACCTTGC	-144	1,1	4.30	-3.30	no	420	Rmet_5705	*aqpZ*	Op1706f_2	EIP-TRA-Pores	
3847	TSS_519162-3	35	13	-35	TTGCGCAGCG	-13	TCCATCATGC	-140	1,1	7.54	11.20	s	5770	Rmet_3953	0	Op1109r_1	NA	
3848	TSS_2501511+2	35	9	-47	TTGAACTGAT	-13	CTTACACTGA	-140	1,1	9.34	-10.60	no	14	Rmet_2278	*exbB1*	Op0651f_3	NA	
3849	TSS_34225-2	35	5	-36	TCGCAAAGTA	-13	TTTAAAATAT	-141	1,1	8.49	10.90	s	260	Rmet_0029	0	Op0010r_1		0
3850	TSS_2782152-2	35	6	-47	TGGACAATAG	-13	AGTAGGATCA	-142	1,1	7.23	-10.10	no	318	Rmet_2558	0	Op0708r_2	NA	
3851	TSS_3352511-2	35	9	-33	TCCACCGGCA	-13	GGTAAACTGC	-139	1,1	7.40	6.60	s	0	Rmet_3093	0	Op0870r_1	NA	
3852	TSS_3427638-2	35	11	-28	TGGAACCACT	-2	AGGACCGTGA	-144	1,1	2.68	-2.30	no	328	Rmet_3161	0	Op0886r_2	NA	
3853	TSS_3898518-2	35	6	-34	TTGAACAGCA	-13	GCGATGATCA	-139	1,1	7.47	10.60	s	3397	Rmet_3587	*acrR*	Op0998r_1	NA	
3854	TSS_210199+3	35	8	-46	TCGATTTTTT	-24	GTAATAATTG	-148	1,1	6.68	1.90	w	170	Rmet_3673	0	Op1022f_1	NA	
3855	TSS_767971+3	35	2	-37	TTGCGGTACG	-16	AGTAGATTGT	-140	1,1	7.50	9.80	s	0	Rmet_6651	0	Op1182f_1	NA	
3856	TSS_815417+3	35	11	-44	GGCAAGGAGC	-14	AGTACCATTC	-141	4,1	8.98	-9.60	no	1936	Rmet_4232	*bug*	Op1200f_1	NA	
3857	TSS_1022829+3	35	18	-35	TTGACACTCG	-14	GCGAGTATGT	-142	1,1	6.78	12.60	s	13	Rmet_4428	0	Op1260f_1	NA	
3858	TSS_1270838+3	35	16	-36	TCCACCTTGA	-15	GTAAGCATTA	-147	1,1	2.95	8.80	s	6326	Rmet_4647	0	Op1342f_1	NA	
3859	TSS_1822412+3	35	7	-48	TGGATTATCC	-14	GGTAATGTGT	-141	1,1	5.39	-14.10	no	92	Rmet_5127	0	Op1512f_1	NA	
3860	TSS_2540844+3	35	21	-48	TCTGACCCGT	-14	CGTACCTTGT	-143	5,1	6.34	-16.60	no	95	Rmet_5782	0	Op1738f_1	NA	
3861	TSS_306917-3	35	11	-36	TCGCAAAGTA	-13	TTTAAAATAT	-141	1,1	8.49	10.90	s	420	Rmet_3769	*alkB1*	Op1047r_1	NA	
3862	TSS_639057-3	35	5	-48	TTGATCGGCC	-11	TCTAATATTA	-145	1,1	7.64	-14.60	no	32	Rmet_4070	0	Op1143r_1	NA	
3863	TSS_803212-3	35	5	-42	TTCATCAGAA	-14	AGTATAATCC	-144	1,1	9.23	0.90	(w)-sba	70	Rmet_4218	0	Op1197r_1	NA	
3864	TSS_1102294-3	35	8	-35	TGGCCAAGGC	-14	GTTACGGTTC	-139	1,1	7.08	10.10	s	1179	Rmet_4499	*rpoJ*	Op1281r_1	GIP-TK-RNAP	
3865	TSS_1108122-3	35	8	-39	TCGCAAATAT	-14	AATAGAATCA	-143	1,1	8.04	5.90	m	168	Rmet_4505	0	Op1285r_1	NA	
3866	TSS_1370094-3	35	7	-35	TTGCTCGGCG	-13	ATTACGCTCT	-140	1,1	6.18	10.20	s	2386	Rmet_4724	*cls*	Op1363r_1	MET-LIP-GlycPLip	
3867	TSS_8636-4	35	10	-24	TGGCGTCATG	-2	GAGACCATCG	-143	1,1	4.73	-2.30	no	3813	Rmet_6060	*repA*	Op1846r_1	NA	
3868	TSS_115540+5	35	9	-40	CCGGAAGGAG	-15	TCTATGATGC	-140	5,1	10.99	1.90	(w)-sba	718	Rmet_6395	0	Op1908f_2	NA	
3869	TSS_204587+2	34	14	-41	TCCCTCTGGT	-16	GCGATACTGT	-140	1,1	5.33	0.40	(w)-sba	156	Rmet_0196	0	Op0057f_2	MET-AA-Urea	
3870	TSS_336380+2	34	4	-30	TCAGGACCGC	-3	GGTACTGTTG	-145	4,1	6.76	-1.80	no	569	Rmet_0318	0	Op0091f_2	NA	
3871	TSS_1047304+2	34	3	-48	GCGGCACGCG	-15	TGCACAATGA	-136	5,1	10.30	-14.10	no	115	Rmet_0962	*ureE*	Op0267f_5	GIP-PTL-Fold	
3872	TSS_1112012+2	34	9	-38	TCGTTTCGGC	-14	GGTAGAATCG	-138	1,1	7.61	6.90	s	479	Rmet_1023	0	Op0295f_2	NA	
3873	TSS_1160300+2	34	17	-36	GTCAGCGCGG	-3	GCTAACGTTT	-141	4,1	5.44	-4.10	no	85	Rmet_1068	*dapA*	Op0307f_2	MET-AA-Lys_Syn	
3874	TSS_2814784+2	34	9	-38	TTCCCCTGGC	-14	AGTATCCTGC	-138	1,1	7.43	6.90	s	440	Rmet_2589	0	Op0723f_1	NA	
3875	TSS_2392092-2	34	8	-35	TGGCGCGCTT	-13	GGCACGATCG	-141	1,1	6.25	8.70	s	714	Rmet_2183	*pstA*	Op0628r_4	EIP-TRA-ABC	
3876	TSS_3364623-2	34	13	-40	CTGCGGGGCT	-13	GATAAAATGG	-142	1,1	6.02	0.40	(w)-sba	51	Rmet_3104	*obgE*	Op0874r_1	NA	
3877	TSS_3643809-2	34	9	-44	TTCATAGGTT	-13	CTCACAATTC	-142	1,1	7.20	-5.10	no	902	Rmet_3367	0	Op0936r_1	NA	
3878	TSS_580057+3	34	8	-43	TTGCTGAGTC	-14	TGCACCATGG	-141	1,1	7.00	-3.60	no	2191	Rmet_6640	0	Op2063f_1	NA	
3879	TSS_1449647+3	34	6	-36	TTGCCAAACG	-15	GGCAACGTGC	-140	1,1	6.22	10.80	s	249	Rmet_4801	0	Op1396f_5	NA	
3880	TSS_1915488-3	34	9	-35	TTGACAGTCG	-15	GTGATATTCG	-141	1,1	7.40	12.60	s	450	Rmet_5220	0	Op1539r_1	NA	
3881	TSS_147839-4	34	10	-37	TCGTCAATCC	-7	GGTAGGGTGT	-142	1,1	5.27	1.90	(w)-ba	1922	Rmet_6151	0	Op1880r_2	NA	
3882	TSS_1139744+2	34	9	-34	TTGCGGGTTC	-14	GATATGCTGA	-140	1,1	8.12	8.60	s	118	Rmet_1047	0	Op0301f_1	NA	
3883	TSS_1567044+2	34	13	-37	TCGTCAGATC	-14	AGTATGATCT	-140	1,1	7.04	10.90	s	138	Rmet_1445	*lpxD*	Op0403f_3	MET-GLYC-LPS	
3884	TSS_2303531+2	34	7	-47	TCGGCTCCTT	-24	CGGATACTGA	-141	5,1	5.93	-1.10	no	11693	Rmet_R0026	*ffs*	Op2026f_1	NA	
3885	TSS_2394580+2	34	7	-43	TGGAACACAT	-14	GGCACCATTA	-141	1,1	6.56	-4.10	no	4464	Rmet_2190	0	Op0629f_1	NA	
3886	TSS_133244-2	34	5	-48	CGCCAAGTGG	-20	ACGAAGATGG	-148	1,1	-0.59	-12.60	no	372	Rmet_0126	*dksA1*	Op0032r_3	NA	
3887	TSS_524129-2	34	9	-39	CTGTGGGGGC	-13	GGTATACTAT	-144	1,1	4.38	2.40	w	3237	Rmet_0493	*ilvE*	Op0132r_1	MET-AA-ValLeuIle	
3888	TSS_665515-2	34	9	-46	TTCACGATCT	-14	CGTAACCTGA	-141	1,1	6.37	-9.10	no	215	Rmet_0610	0	Op0172r_1	MET-LIP-FASyn	
3889	TSS_861891-2	34	7	-41	TCCAGCGCGG	-13	CGCAAACTAC	-144	1,1	3.56	-2.60	no	560	Rmet_0784	*purD*	Op0222r_1	MET-NUC-Pur	
3890	TSS_1309692-2	34	8	-35	CCGGCCCTGC	-12	GGTACGGTTG	-137	5,1	8.32	5.70	m	401	Rmet_1194	0	Op0346r_2	NA	
3891	TSS_1952336-2	34	4	-34	TTGCTTAACT	-14	TCTATACTAA	-142	1,1	8.43	9.60	s	0	Rmet_1799	0	Op0522r_1	NA	
3892	TSS_2055982-2	34	2	-34	TTGAGCGTAT	-12	CGTAGTCTTG	-141	1,1	6.97	10.20	s	645	Rmet_1887	*trmC*	Op0544r_1	NA	
3893	TSS_2229103-2	34	5	-35	TTGTCCCACC	-14	GCGACAATGC	-139	1,1	8.24	11.60	s	1528	Rmet_2055	*ugpB*	Op0596r_1	EIP-TRA-ABC	
3894	TSS_3091955-2	34	11	-30	TTGAAATTTT	-4	GCAATAATAC	-146	1,1	9.12	5.20	(m)-nu	1439	Rmet_2842	*tnpA*	Op2044r_1	NA	
3895	TSS_3201617-2	34	13	-35	TGGCGCTTCA	-14	ATCATGATGA	-142	1,1	5.45	9.10	s	2636	Rmet_2942	*recG*	Op0830r_1	GIP-REP-Recomb	

3896	TSS_3566562-2	34	13	-48	TCGAGGCGCG	-14	GGCACCATGC	-138	1,1	6.06	-14.10	no	305	Rmet_3288	dsbD		Op0926r_2	GIP-PTL-Fold
3897	TSS_3612419-2	34	5	-27	TGGTTCGAAT	-5	ACCAGAATGC	-142	1,1	5.65	1.70	(w)-nu	48	Rmet_R0058		0	Op0930r_3	NA
3898	TSS_2560493+3	34	10	-38	TTCCGATGAT	-14	ACTACGCTGA	-140	1,1	6.48	5.90	m	5465	Rmet_5806	fecA1		Op1750f_1	EIP-TRA-Pores
3899	TSS_1393627-3	34	6	-34	TTTCTCCGGG	-13	GCGATATTCG	-141	2,1	10.05	8.10	s	212	Rmet_4749	hupB		Op1377r_2	GIP-REP-Recomb
3900	TSS_2467684-3	34	1	-37	TTCACCGCGA	-14	GCCATACTGC	-139	1,1	7.68	9.90	s	904	Rmet_5709		0	Op1707r_1	NA
3901	TSS_77232-5	34	0	-42	CCAGACCCGC	-13	GTTACGATAC	-141	5,1	8.52	-7.60	no	2429	Rmet_6222		0	Op1899r_1	NA
3902	TSS_126431-5	34	13	-45	TTGCCGATGA	-14	GGCATAATGG	-138	1,1	10.58	-4.60	no	507	Rmet_6398		0	Op1911r_1	NA
3903	TSS_1889058+2	34	9	-47	TTGATAGTGC	-14	CCTATCCTGA	-139	1,1	7.74	-8.60	no	17	Rmet_1742		0	Op0495f_1	NA
3904	TSS_2583039+2	34	2	-39	TCAAGGGAGC	-9	CATACATTCA	-144	4,1	7.07	-1.60	no	943	Rmet_2373		0	Op0669f_1	NA
3905	TSS_2765706+2	34	4	-43	TTGCAGTTGT	-15	GGAACAATCA	-138	1,1	8.32	-1.60	no	144	Rmet_2540		0	Op0701f_3	EIP-TRA-ABC
3906	TSS_2956710+2	34	4	-40	CTGAAGACGC	-15	GGCATTATCG	-143	1,1	3.68	2.40	(w)-sba	220	Rmet_2722		0	Op0769f_2	NA
3907	TSS_590784-2	34	5	-33	TGGCTTTTTT	-13	GCGACTATAG	-145	1,1	4.80	5.10	m	5	Rmet_0552	fdsR		Op0154r_1	NA
3908	TSS_905990-2	34	16	-28	TTGCTGCGAA	-7	TTCATGTTGT	-142	1,1	4.75	2.60	(w)-nu	246	Rmet_0830		0	Op0236r_1	NA
3909	TSS_962243-2	34	10	-49	CGGCCGTGAA	-29	ATGATCATGC	-144	1,1	1.14	-6.70	no	2156	Rmet_6432		0	Op0254r_1	NA
3910	TSS_2155867-2	34	4	-46	TCAGAAGCCT	-13	GATACCCTCC	-140	5,1	7.89	-12.60	no	815	Rmet_1980	ansB		Op0576r_1	MET-EN-Nitrog
3911	TSS_3428986-2	34	6	-45	GAACTCGGCA	-25	GTAACTTCGG	-145	3,0	6.42	-9.20	no	1300	Rmet_R0050		0	Op0886r_1	NA
3912	TSS_476879+3	34	8	-46	GAACTCGGCA	-26	GTAACTTCGG	-146	3,0	6.42	-10.20	no	1299	Rmet_R0079		0	Op1098f_1	NA
3913	TSS_770796+3	34	6	-39	TTGCCCGGCC	-15	AGCATCATGA	-140	1,1	7.07	7.40	s	708	Rmet_4188		0	Op1184f_2	NA
3914	TSS_955413+3	34	2	-45	GTCGGGGTCC	-15	CGTAAAATAT	-143	4,1	7.49	-9.10	no	147	Rmet_4360	oxc		Op1244f_1	MET-CAH-But
3915	TSS_1230216+3	34	7	-43	GGAGAGGCAC	-13	GGCAACATTG	-140	4,1	8.26	-10.60	no	706	Rmet_4606	metY		Op1326f_1	MET-AA-Met
3916	TSS_1304803+3	34	6	-37	CCAGCCATGC	-14	GTGATACTCC	-140	5,1	8.31	4.40	m	1151	Rmet_4673		0	Op1352f_1	NA
3917	TSS_945278-3	34	13	-25	TTGCGTCCGC	-3	GGCAAGCTCC	-141	1,1	6.07	-0.80	no	189	Rmet_4348		0	Op1237r_1	NA
3918	TSS_1933753-3	34	8	-39	GGAACAGAAC	-14	GGTATTGTTC	-142	4,1	8.80	2.40	w	5656	Rmet_5230	iscS2		Op1543r_1	NA
3919	TSS_1981856-3	34	3	-35	TCGTGGGTTT	-14	GGCAGAATGC	-140	1,1	6.74	9.10	s	1879	Rmet_5279		0	Op1555r_1	NA
3920	TSS_789-5	34	4	-34	GCCAAGGTGT	-13	GGTACGATGA	-140	4,1	9.26	4.60	m	14889	Rmet_6301	trbF		Op1917r_1	NA
3921	TSS_2838200+2	33	7	-47	TGGCGGATCC	-14	GATAGAACTG	-146	1,1	0.79	-13.10	no	226	Rmet_6542		0	Op2038f_1	NA
3922	TSS_2971693+2	33	7	-46	GTAACCATCC	-18	CCCATGCTAA	-147	4,1	4.15	-8.10	no	7928	Rmet_2745	phr		Op0773f_1	GIP-REP-Recomb
3923	TSS_3607320+2	33	2	-47	TTGATCACGC	-14	GCCAGAATCA	-140	1,1	8.09	-9.60	no	10696	Rmet_6590		0	Op0931f_1	NA
3924	TSS_1384495-2	33	13	-49	GCATCAACGG	-13	CATAACATGC	-144	5,1	6.81	-19.60	no	11	Rmet_1262	tnpA		Op0364r_1	NA
3925	TSS_2939094-2	33	1	-40	TCGCGCTCCG	-13	GGTATGCTGT	-137	1,1	7.08	0.90	(w)-sba	2035	Rmet_2703		0	Op0764r_1	NA
3926	TSS_4365+3	33	3	-22	CCGGCCGTGT	-2	GTTCAACTCA	-145	5,1	5.19	-8.90	no	2899	Rmet_5822		0	Op1760f_1	NA
3927	TSS_152477+3	33	12	-31	CCTGCCCGGT	-11	TAAATGATGG	-145	5,1	7.28	0.60	w	1151	Rmet_R0074		0	Op1006f_1	NA
3928	TSS_2311946+3	33	10	-49	GGCGAAGTAC	-14	AGTACACTTC	-141	4,1	9.23	-19.60	no	3754	Rmet_5563		0	Op1652f_1	NA
3929	TSS_2400700+3	33	15	-30	TTGACGATGA	-3	GTCAGAGAGG	-147	1,1	1.88	0.20	no	77	Rmet_5641		0	Op1682f_2	DIV-MOT-Chemotax
3930	TSS_2259418-3	33	8	-40	TGGCGCCGTA	-14	GCGATGCTTC	-143	1,1	5.09	0.90	(w)-sba	5403	Rmet_5508		0	Op1639r_1	NA
3931	TSS_18269+4	33	3	-36	TTGTGTATAC	-15	CGGAAAATGT	-143	1,1	6.99	10.80	s	447	Rmet_6047		0	Op1843f_1	NA
3932	TSS_27343-5	33	4	-42	TCAAAACCAA	-13	GGCAAAGTCG	-145	4,1	5.77	-3.60	no	6018	Rmet_6332		0	Op1925r_1	NA
3933	TSS_304866+2	33	4	-36	TTGCACAGCG	-15	TCGATCATGT	-139	1,1	7.04	10.80	s	4650	Rmet_0293	mutM		Op0085f_1	NA
3934	TSS_804387+2	33	12	-47	TCGCAACATG	-25	GTCATTATTG	-143	1,1	6.47	-0.10	no	149	Rmet_0727	rfaE		Op0205f_2	NA
3935	TSS_1578990+2	33	6	-49	TGGAGGTGTT	-29	GATACGCTGC	-141	1,1	7.44	-4.70	no	265	Rmet_1458	yfjF		Op0407f_2	NA
3936	TSS_1669081+2	33	15	-48	TTGAGGTGAC	-15	TTCAAGATGA	-144	1,1	6.91	-11.60	no	519	Rmet_1547		0	Op0431f_1	NA
3937	TSS_1803409+2	33	10	-36	TCGCTATTTA	-14	TGCATACTGG	-141	1,1	6.72	10.90	s	1494	Rmet_1668	tnp		Op0469f_1	NA
3938	TSS_2897786+2	33	10	-35	CTGCCGTCGG	-15	TGTAAAGTGC	-145	1,1	4.07	7.60	s	1932	Rmet_2665		0	Op0755f_1	NA
3939	TSS_2959694+2	33	1	-47	TCGAACGAAG	-14	GAGAAAATAG	-144	1,1	6.29	-11.10	no	1578	Rmet_6554		0	Op2042f_1	NA
3940	TSS_3541380+2	33	9	-35	GCACGAACGG	-14	TGTAGCATGC	-137	4,1	9.41	5.60	m	6310	Rmet_6587		0	Op2052f_1	NA
3941	TSS_3769777+2	33	2	-49	GCGACATCAG	-13	GACATAATGC	-141	5,1	7.61	-16.10	no	22394	Rmet_3502		0	Op0971f_1	NA
3942	TSS_3782063+2	33	11	-35	TTGAGGTACG	-15	GGTAGCATTG	-136	1,1	9.08	10.60	s	10108	Rmet_3502		0	Op0971f_1	NA
3943	TSS_232368-2	33	3	-32	TTGCCGTAGG	-5	TAGACGATCT	-141	1,1	6.41	3.20	(w)-ba	5730	Rmet_0213	crp1		Op0064r_1	NA
3944	TSS_270248-2	33	3	-37	TTCACGGCGT	-13	CTTACATTAC	-143	1,1	6.55	8.90	s	1669	Rmet_0251	gpmA		Op0072r_1	MET-CAH-FbP
3945	TSS_1120482-2	33	11	-38	TCTGACGCGG	-13	GGCATGATCA	-143	5,1	7.69	3.40	m	11416	Rmet_1018	tyrB		Op0294r_1	MET-AA-Met
3946	TSS_3614507-2	33	5	-48	TAGCTGATGG	-12	CGTATGATGC	-143	1,1	5.09	-15.10	no	1588	Rmet_R0060		0	Op0930r_1	NA
3947	TSS_3783867-2	33	4	-40	TTGAAAGCGT	-14	GTCATAATGT	-139	1,1	10.37	6.40	(s)-sba	178	Rmet_3492		0	Op0970r_2	MET-CAH-But
3948	TSS_478074+3	33	3	-34	TTGCCCGTAA	-10	ACCAGTGTGT	-147	1,1	3.77	7.20	s	104	Rmet_R0079		0	Op1098f_1	NA
3949	TSS_2373768+3	33	1	-35	CCGGCCCGAT	-13	GAGAGAATGC	-143	5,1	9.83	7.70	s	121	Rmet_5617		0	Op1676f_1	MET-LIP-Ster
3950	TSS_2264858-3	33	5	-38	TGGATCCGGC	-14	AGTAAGCTAT	-143	1,1	5.17	5.90	m	249	Rmet_5517	vanB		Op1641r_1	MET-XEN-24D
3951	TSS_2284384-3	33	3	-35	TTGCCAAGGT	-14	AGAACAATTT	-139	1,1	8.14	12.60	s	1428	Rmet_6741		0	Op1645r_1	NA
3952	TSS_2405022-3	33	15	-35	TGGCGGTGGT	-13	GGCACAATGT	-135	1,1	8.65	9.70	s	123	Rmet_5643		0	Op1683r_2	NA
3953	TSS_116694-4	33	10	-49	GCAAAAATAC	-18	GGCAGCGTAA	-144	4,1	6.24	-16.60	no	2198	Rmet_5947	pbrA		Op1808r_1	MET-EN-P_ATPase
3954	TSS_129730-5	33	11	-35	TCGCAAGCTG	-14	TGTAGAGTTG	-141	1,1	6.39	10.10	s	1451	Rmet_6272		0	Op1913r_2	NA
3955	TSS_170573-5	33	11	-41	TCGACACCAG	-15	TATAAGCTCC	-145	1,1	5.95	2.90	(w)-sba	13214	Rmet_6301	trbF		Op1917r_1	NA
3956	TSS_48106+2	33	13	-41	TCGACATATA	-13	ACTACACTGA	-146	1,1	7.89	1.90	(w)-sba	38	Rmet_0043		0	Op0015f_3	NA

3957	TSS_2668672+2	33	7	-36	TTGACCGGCC	-14	ATTGAAATGA	-143	1,1	3.19	12.40	s	33068	Rmet_2489	*mdh*		Op0689f_1	MET-CAH-TCA	
3958	TSS_3365150+2	33	8	-40	TTGTCGTGAC	-14	GGTAGCCTTG	-140	1,1	7.63	3.40	*(m)-sba*	632	Rmet_3107	*ispB*		Op0875f_1	NA	
3959	TSS_3683289+2	33	6	-25	TTTACATTGT	-1	TAGATAGTAG	-154	1,1	-0.04	0.70	*(w)-nu*	33	Rmet_6595		*0*	Op0945f_2	NA	
3960	TSS_314593-2	33	11	-40	TTTCTTTCCG	-13	GCTACCCTGG	-136	2,1	12.10	-0.10	*no*	5347	Rmet_0292		*0*	Op0084r_1	NA	
3961	TSS_665392-2	33	14	-34	TTGCCCTTTG	-13	TATACAGTCT	-141	1,1	7.92	10.60	s	92	Rmet_0610		*0*	Op0172r_1	MET-LIP-FASyn	
3962	TSS_863130-2	33	10	-26	TCGCCGGCCT	-4	CGAACAATTC	-142	1,1	5.77	1.70	*(w)-nu*	989	Rmet_0785		*0*	Op0222r_1	NA	
3963	TSS_1061152-2	33	10	-30	GCGGCAAGTG	-2	GCTAATTTAA	-142	5,1	7.33	-3.30	*no*	29	Rmet_0974	*paaH*		Op0272r_1	MET-CAH-But	
3964	TSS_1975234-2	33	11	-42	GGCGCGACAT	-14	CTTATGCTCC	-143	4,1	6.86	-5.60	*no*	4042	Rmet_1816		*0*	Op0526r_1	MET-XEN-Others	
3965	TSS_2729995-2	33	2	-41	TGGACAACGC	-14	CATACACTTG	-138	1,1	8.32	2.90	*(w)-sba*	6081	Rmet_2507		*0*	Op0694r_1	NA	
3966	TSS_3326172-2	33	5	-48	CCGGCAGGAT	-13	GTCATGATGG	-139	5,1	10.35	-16.10	*no*	7666	Rmet_3060	*mpl*		Op0860r_1	NA	
3967	TSS_3538890-2	33	9	-41	GCAACACTAG	-13	GCTATCGTCA	-142	4,1	6.44	-2.60	*no*	447	Rmet_3263	*gltB*		Op0920r_3	MET-EN-Nitrog	
3968	TSS_3577321-2	33	8	-31	TGGCGCAGAT	-7	ATAACAATGC	-139	1,1	6.14	3.70	*(m)-ba*	427	Rmet_3309	*rpmC*		Op0926r_2	GIP-TL-Ribo	
3969	TSS_294546+3	33	3	-41	GGAAGAAAGA	-14	AGTACGATGG	-143	4,1	8.93	-2.60	*no*	910	Rmet_3751		*0*	Op1044f_1	NA	
3970	TSS_2109571+3	33	6	-43	TTGCCCATCG	-14	GGCATACTGC	-136	1,1	9.23	-1.60	*no*	26	Rmet_5383		*0*	Op1602f_1	NA	
3971	TSS_1181696-3	33	6	-38	TCGCATCTGA	-13	TGGACAATTA	-141	1,1	6.13	4.90	m	629	Rmet_R0082		*0*	Op1315r_2	NA	
3972	TSS_1742145-3	33	3	-39	TGGTGGTGCC	-13	GGCATGATCA	-140	1,1	5.84	2.90	w	616	Rmet_5051		*0*	Op1485r_1	NA	
3973	TSS_2237443-3	33	3	-28	TTCATCTCTG	-1	CTGACCATCT	-144	1,1	3.90	-2.30	*no*	1648	Rmet_5490	*pobA*		Op1631r_1	MET-XEN-24D	
3974	TSS_117103-5	33	7	-38	TTGCTGACCA	-14	TTTATCATCG	-140	1,1	8.30	8.40	s	2483	Rmet_6260	*tnpA*		Op1907r_1	NA	
3975	TSS_268754+2	32	15	-37	TGCCAACGTG	-14	ATTACAATAA	-142	1,1	6.63	8.40	s	461	Rmet_0252	*grxC*		Op0073f_1	GIP-PTL-Fold	
3976	TSS_407978+2	32	13	-38	TTCACAATGC	-14	GTCACAATGC	-139	1,1	9.20	8.90	s	23	Rmet_0383		*0*	Op0105f_1	MET-LIP-GlycPLip	
3977	TSS_1314011+2	32	5	-35	TCCACCACGA	-14	CGTATCCTGA	-141	1,1	5.11	9.60	s	1356	Rmet_1197	*aceF*		Op0347f_2	MET-CAH-FbP	
3978	TSS_1701145+2	32	4	-35	TGGCAACTGA	-11	TCAAAGATTG	-143	1,1	4.89	7.70	s	67	Rmet_1579		*0*	Op0435f_2	MET-NUC-Pur	
3979	TSS_3874328+2	32	4	-48	TGGCGAAGAC	-14	GACATAGTCT	-142	1,1	6.30	-13.10	*no*	153	Rmet_3571	*copQ1*		Op0991f_1	NA	
3980	TSS_151515-2	32	6	-38	TTGTTCGGGG	-12	GCAATAATAC	-141	1,1	6.73	6.40	s	15	Rmet_0146	*ampG*		Op0040r_1		0
3981	TSS_240882-2	32	7	-27	TGGACCGGTG	0	GGCCGTCTGG	-145	1,1	-0.13	-5.30	*no*	236	Rmet_0226		*0*	Op0066r_1	NA	
3982	TSS_576415-2	32	14	-25	TTGAACAAGG	-1	GGAAAGCTCG	-142	1,1	5.65	-1.80	*no*	4604	Rmet_0537	*exbB2*		Op0148r_1	NA	
3983	TSS_1903582-2	32	12	-42	TCGCGAGGGT	-13	ACGAAAATAA	-143	1,1	5.61	-2.10	*no*	2958	Rmet_1750	*agrA*		Op0498r_1	NA	
3984	TSS_3403449-2	32	11	-24	TTGACCCTGT	-3	TGTCTGGTTT	-148	1,1	2.74	-0.40	*no*	184	Rmet_3138		*0*	Op0878r_1	MET-COF-CoQ	
3985	TSS_3479881-2	32	6	-44	TTGACGCTGT	-17	TATAACGTTC	-144	1,1	6.78	-0.60	*no*	26	Rmet_3206		*0*	Op0902r_2	NA	
3986	TSS_83905+3	32	9	-37	TTCCGCGGCG	-15	GATACCATGC	-137	1,1	7.54	8.90	s	1863	Rmet_5888		*0*	Op1784f_1	NA	
3987	TSS_487109+3	32	1	-39	TCGACGCGAG	-15	CATATCATCT	-140	1,1	6.95	7.90	s	4709	Rmet_3934		*0*	Op1100f_1	EIP-SIG-2Comp	
3988	TSS_1563716+3	32	7	-40	TTCTATGCAT	-14	AGCATAATGA	-147	1,1	6.35	2.90	*(w)-sba*	293	Rmet_4901		*0*	Op1434f_2	NA	
3989	TSS_1633641+3	32	6	-45	TTGCCCTGAT	-13	GGTAAGATAG	-137	1,1	9.72	-6.60	*no*	2129	Rmet_6699		*0*	Op2081f_1	NA	
3990	TSS_2426368+3	32	8	-29	TTGTCGTCAC	-5	ACGAGGTTGT	-148	1,1	3.06	2.20	*(w)-nu*	118	Rmet_5667		*0*	Op1694f_3	NA	
3991	TSS_2438495+3	32	2	-41	TCCATCACCT	-15	GATATCGTCC	-142	1,1	4.12	0.40	*(w)-sba*	3835	Rmet_5684	*pilE*		Op1698f_1	GIP-PTL-T2S	
3992	TSS_2537251-3	32	6	-49	TTGACGCACG	-13	AATAGACTGA	-141	1,1	7.79	-13.60	*no*	59	Rmet_5777		*0*	Op1737r_1	NA	
3993	TSS_78143+4	32	12	-38	GCAGACGCGG	-14	CGTAACATTG	-140	4,1	8.32	1.40	w	308	Rmet_5980	*czcA*		Op1819f_1	EIP-TRA-Ion	
3994	TSS_145461+4	32	5	-37	TTGACGCGTG	-15	GCCAGAATTG	-138	1,1	8.94	12.40	s	2684	Rmet_6149	*bug*		Op1879f_1	NA	
3995	TSS_58871-4	32	9	-26	TGGCAGATGC	-2	TGAACCATGT	-143	1,1	5.05	-2.30	*no*	1061	Rmet_6000		*0*	Op1828r_3	NA	
3996	TSS_131159-4	32	9	-42	TCGCCGGAGC	-14	GGCATCACTT	-141	1,1	6.76	-1.10	*no*	1225	Rmet_6172	*merT*		Op1890r_1	EIP-TRA-Other	
3997	TSS_201916-4	32	6	-30	GAAATCCGTG	-10	ATAATTGAGC	-145	3,0	5.43	-3.40	*no*	236	Rmet_6099		*0*	Op1860r_1	NA	
3998	TSS_224049-4	32	4	-23	TTCCAAACGG	-2	GGTAGATTGT	-137	1,1	7.81	-1.90	*no*	3	Rmet_6078		*0*	Op1852r_1	NA	
3999	TSS_57815+5	32	9	-41	TTGTGGTGGA	-21	GGGAAGATGG	-140	1,1	6.71	3.80	*m*	2582	Rmet_6211	*cnrT*		Op1896f_2	NA	
4000	TSS_288890+2	32	9	-36	TTGATCGGCA	-14	TTCACTATGA	-140	1,1	5.75	11.40	s	321	Rmet_0273		*0*	Op0079f_1	NA	
4001	TSS_1126410+2	32	10	-39	TCGCGGAGCC	-15	GCGACAATAA	-140	1,1	6.24	4.90	m	45	Rmet_1037	*nlpD*		Op0297f_1	NA	
4002	TSS_1464238+2	32	6	-46	TCGAAACCGA	-13	GCTAGCATAG	-144	1,1	7.08	-9.10	*no*	2105	Rmet_1357	*phaA*		Op0383f_2	MET-CAH-Pyr	
4003	TSS_3642383+2	32	8	-36	TGGATGCCCC	-14	GGTACGTTGG	-142	1,1	5.77	9.90	s	3414	Rmet_3369	*paaA*		Op0937f_1	NA	
4004	TSS_153635-2	32	1	-35	CCTGCACCAG	-13	GCTACAATGC	-135	5,1	12.30	8.20	s	2135	Rmet_0146	*ampG*		Op0040r_1		0
4005	TSS_960960-2	32	11	-35	TTGCGCTTCG	-14	GGCATGTTGC	-139	1,1	6.42	9.60	s	873	Rmet_6432		*0*	Op0254r_1	NA	
4006	TSS_1137735-2	32	12	-32	GAACTGGTTG	-12	TGCCATAGAA	-144	3,0	4.67	-4.40	no	973	Rmet_1044	*yebK*		Op0300r_1	NA	
4007	TSS_2698517-2	32	9	-40	TCACGGAAGG	-14	GGTATCCTGC	-138	4,1	7.97	0.40	*(w)-sba*	1000	Rmet_2483	*sdhB*		Op0688r_2	MET-CAH-TCA	
4008	TSS_3150277-2	32	6	-39	TTGAACCATT	-13	CATAAACTCC	-141	1,1	8.83	5.40	m	553	Rmet_2893	*clpS*		Op0812r_1	GIP-PTL-Fold	
4009	TSS_3606687-2	32	12	-34	TGGCCAGGTC	-13	GCGATGATTC	-138	1,1	7.21	10.10	s	5	Rmet_3334	*rpoB*		Op0930r_4	GIP-TK-RNAP	
4010	TSS_1205811+3	32	10	-48	TTCAAAGCCT	-22	GGTCTTGTCG	-145	1,1	1.61	-6.10	*no*	55	Rmet_4587		*0*	Op1320f_1	NA	
4011	TSS_1638433+3	32	12	-48	GCGGAAGGCC	-15	CGTAAACTTC	-138	5,1	10.55	-15.10	*no*	1764	Rmet_4967		*0*	Op1458f_1	NA	
4012	TSS_2078146+3	32	14	-48	TCACGGCTGC	-14	GCTAACCTCG	-143	4,1	6.36	-16.60	*no*	26	Rmet_5357		*0*	Op1592f_1	MET-CAH-Prop	
4013	TSS_665709-3	32	13	-35	TTCCCGTCCG	-14	ACTACTATAG	-142	1,1	6.28	10.10	s	21	Rmet_4097		*0*	Op1153r_1	NA	
4014	TSS_705367-3	32	4	-40	CGGATAGGAA	-14	TGGATAATCC	-146	1,1	2.79	1.90	*(w)-sba*	6421	Rmet_4127		*0*	Op1163r_1	NA	
4015	TSS_1087959-3	32	7	-36	TTGACGATGA	-15	GAAAAGATGG	-142	1,1	6.71	11.80	s	623	Rmet_4487		*0*	Op1279r_2	NA	
4016	TSS_1245427-3	32	8	-41	TGACGGCCGT	-13	GCTAAGCTTC	-142	4,1	6.35	-3.60	*no*	4554	Rmet_4614		*0*	Op1329r_1	NA	
4017	TSS_1311259-3	32	8	-36	CCTGCAGTAT	-14	GGCATCATGG	-140	5,1	9.60	7.40	s	400	Rmet_4679		*0*	Op1353r_2	NA	

4018	TSS_122168+2	32	6	-35	TGGCGCGTGC	-14	GCTACCCTCA	-138	1,1	6.46	8.10	s	733	Rmet_0116	*bioD*	Op0031f_5	MET-COF-Biotine	
4019	TSS_154584+2	32	9	-27	TTGAGGTTGG	-6	CGTAACATCT	-140	1,1	8.49	3.60	(m)-nu	31	Rmet_0150	*pyrE*	Op0043f_1	MET-NUC-Pyr	
4020	TSS_203666+2	32	2	-35	TCGCATAATC	-13	GATATTTTCG	-145	1,1	5.71	9.70	s	79	Rmet_0195	*bfr*	Op0057f_1	NA	
4021	TSS_436937+2	32	11	-21	CCGGCCCCGT	0	TGTCTCCTCC	-143	5,1	4.15	-8.90	no	349	Rmet_0410	*rplM*	Op0113f_2	GIP-TL-Ribo	
4022	TSS_1026060+2	32	8	-37	TGGTGAACCT	-15	GGCACAGTCC	-141	1,1	5.42	8.90	s	22	Rmet_0940	*nuoN*	Op0267f_3	MET-EN-OxPhos	
4023	TSS_2575290+2	32	6	-30	CCTGCCCGGC	-6	CTCAGCATAG	-144	5,1	6.51	-1.80	no	53	Rmet_2364	*0*	Op0665f_1	NA	
4024	TSS_199465-2	32	8	-36	TTGCCACTCG	-14	CATATCCTGC	-142	1,1	7.96	13.40	s	7007	Rmet_0185	*0*	Op0056r_1	NA	
4025	TSS_506291-2	32	6	-43	TCGGAATGAC	-14	GGCAAATTGC	-144	5,1	7.45	-4.10	no	93	Rmet_0476	*0*	Op0124r_1	NA	
4026	TSS_1466219-2	32	3	-34	TTGACGTAGC	-13	GGTTCAATCG	-139	1,1	5.64	10.60	s	2188	Rmet_1355	*0*	Op0382r_1	NA	
4027	TSS_1580558-2	32	6	-35	TTGCCACTGA	-14	CAGATGATCG	-141	1,1	6.85	12.60	s	232	Rmet_1459	*0*	Op0408r_2	NA	
4028	TSS_3092993-2	32	6	-37	GCTACCGTCG	-13	GATACACTGC	-138	5,1	8.25	4.40	m	2477	Rmet_2842	*tnpA*	Op2044r_1	NA	
4029	TSS_3525558-2	32	6	-34	TTGTTCGTCC	-2	GACACGATTC	-141	1,1	6.08	-0.80	no	95	Rmet_3253	*yadG*	Op0918r_3	NA	
4030	TSS_3848387-2	32	13	-25	TTGACTTGGC	-4	ATCTCACTAC	-148	1,1	0.77	-0.40	no	124	Rmet_3548	*0*	Op0984r_1	NA	
4031	TSS_150554+3	32	15	-38	TTGTGATTGT	-13	TTTAAAGTGA	-145	1,1	7.70	7.40	s	77	Rmet_R0073	*0*	Op1006f_1	NA	
4032	TSS_252992+3	32	4	-34	TTGAATTCCG	-7	GCGAGGCTGT	-142	1,1	5.35	4.20	(m)-ba	757	Rmet_3711	*0*	Op1028f_2	NA	
4033	TSS_475104+3	32	9	-38	TTGTGATTGT	-13	TTTAAAGTGA	-145	1,1	7.70	7.40	s	77	Rmet_R0078	*0*	Op1098f_1	NA	
4034	TSS_2500226+3	32	3	-30	TTCAGAAATT	-5	GGAATATTCC	-147	1,1	5.98	3.70	(m)-nu	201	Rmet_5738	*0*	Op1716f_2	NA	
4035	TSS_1242894-3	32	6	-41	TTGTTTACAG	-13	GTCACAATTT	-147	1,1	7.40	0.40	(w)-sba	2021	Rmet_4614	*0*	Op1329r_1	NA	
4036	TSS_212690+4	32	14	-40	GCCCCCGATC	-14	GTGACAATGA	-144	4,1	5.08	-1.60	no	20	Rmet_6387	*0*	Op1853f_1	NA	
4037	TSS_5628-4	32	8	-46	TTGCACCATG	-14	AATAAACTAC	-142	1,1	7.84	-8.60	no	805	Rmet_6060	*repA*	Op1846r_1	NA	
4038	TSS_11934-4	32	6	-36	GCAGAACAGG	-14	CTTACAATGG	-141	4,1	10.33	7.40	s	7111	Rmet_6060	*repA*	Op1846r_1	NA	
4039	TSS_12520+5	32	7	-24	GGCACGACGC	0	GCTAGATTAG	-144	4,1	7.87	-5.80	no	4010	Rmet_6327	*0*	Op1922f_1	NA	
4040	TSS_607350+2	31	4	-34	TGCATCGAGA	-14	GATATGCTGC	-140	1,1	5.86	6.60	s	296	Rmet_0567	*modE*	Op0159f_1	EIP-TRA-ABC	
4041	TSS_3207448+2	31	4	-47	TGGTCGAGTT	-23	GTCAGAATCC	-139	1,1	7.15	-2.10	no	8242	Rmet_6568	*cobT*	Op0833f_1	NA	
4042	TSS_3346514+2	31	14	-36	TTGAAGACTT	-3	AGCAAAATGC	-144	1,1	8.95	1.40	(w)-ba	6114	Rmet_3094	*ffh*	Op0871f_1	GIP-PTL-Exp	
4043	TSS_96276-2	31	8	-42	GTCGGGCCGA	-13	TTTAGGATGT	-143	4,1	5.24	-6.10	no	3186	Rmet_0086	*0*	Op0024r_1		0
4044	TSS_1254843-2	31	5	-36	TTGCGCCACG	-13	GTTACGATCC	-139	1,1	8.90	10.40	s	5500	Rmet_1141	*0*	Op0326r_1	NA	
4045	TSS_1278805-2	31	9	-36	TGGACGTCAC	-13	CAGAAAATGG	-142	1,1	6.74	10.90	s	12925	Rmet_1156	*bdhA*	Op0332r_1	MET-CAH-But	
4046	TSS_1464350-2	31	7	-34	GCTGCCGCAC	-13	GCCATAATGT	-139	5,1	10.71	5.60	m	319	Rmet_1355	*0*	Op0382r_1	NA	
4047	TSS_1796974-2	31	1	-43	CCGCGACAAT	-13	AATATAGTGA	-147	1,1	1.85	-6.10	no	316	Rmet_6464	*0*	Op0468r_3	NA	
4048	TSS_2872331-2	31	1	-45	GGCGCACCGT	-13	GCTATTTTGG	-143	4,1	6.56	-11.60	no	3248	Rmet_6544	*0*	Op0738r_1	NA	
4049	TSS_3429274-2	31	6	-48	TAGCTGATGG	-12	CGTATGATGC	-143	1,1	5.09	-15.10	no	1588	Rmet_R0050	*0*	Op0886r_1	NA	
4050	TSS_3433506-2	31	8	-48	GCAGACAAAC	-12	GAGACTATGC	-138	4,1	7.79	-21.60	no	261	Rmet_6579	*0*	Op0886r_1	NA	
4051	TSS_3855897-2	31	5	-43	CTGACGGCAC	-14	GCTATGATGC	-144	1,1	5.80	-2.60	no	87	Rmet_3555	*0*	Op0986r_1	NA	
4052	TSS_91279+3	31	6	-35	TAGACACGAA	-14	TGAATAATGG	-148	1,1	4.55	13.10	s	67	Rmet_5893	*0*	Op1786f_1	NA	
4053	TSS_150080+3	31	16	-23	TTCATGACTG	-1	AACAAGGTAG	-147	1,1	1.60	-3.30	no	105	Rmet_R0071	*tRNA-I*	Op1006f_1	NA	
4054	TSS_1294072+3	31	5	-36	TCGAGAACTT	-12	AATCGAATCG	-144	1,1	2.96	8.90	s	3688	Rmet_4665	*0*	Op1350f_1	NA	
4055	TSS_766143-3	31	14	-42	TGGCGGTCAT	-17	GGGAACCTCG	-142	1,1	4.69	-1.10	no	183	Rmet_4180	*0*	Op1179r_1	NA	
4056	TSS_786173-3	31	2	-41	TCGCCGCCAC	-13	GCCATAGTCG	-141	1,1	5.89	-0.10	no	3035	Rmet_4197	*0*	Op1189r_1	NA	
4057	TSS_913864-3	31	5	-34	TCTTCAACGC	-14	CCTAGAATCC	-140	5,1	8.37	8.60	s	1587	Rmet_4317	*0*	Op1229r_1	NA	
4058	TSS_1489400-3	31	11	-34	TGGCAATAAC	-4	TTCATACTGA	-145	1,1	7.18	1.70	(w)-ba	129	Rmet_4834	*ompP*	Op1409r_1	NA	
4059	TSS_2234374-3	31	4	-33	GCCCAACCGC	-13	GCGATACTCT	-138	4,1	8.42	2.60	w	1853	Rmet_5488	*0*	Op1629r_1	GIP-REP-Recomb	
4060	TSS_2288355-3	31	3	-34	TTGCGGAGGC	-14	GAGAGAATGC	-141	1,1	8.45	8.60	s	383	Rmet_5541	*tnpB*	Op1647r_3	NA	
4061	TSS_101020-4	31	5	-48	TTCCAATACG	-20	AGGAGATTTG	-146	1,1	4.50	-8.10	no	154	Rmet_6371	*0*	Op2112r_1	NA	
4062	TSS_81409-5	31	10	-46	TGAGCCGTAC	-13	GCTATCCTGC	-141	4,1	6.87	-11.60	no	402	Rmet_6229	*0*	Op1901r_3	NA	
4063	TSS_133068+2	31	4	-46	GCCAAAACGT	-14	CCTATTGTAC	-142	4,1	7.67	-12.60	no	1906	Rmet_0129	*dehH2*	Op0033f_1	MET-XEN-Hex	
4064	TSS_215620+2	31	3	-41	CCTGAAATGC	-14	GGTACGCTAC	-138	5,1	8.42	-4.60	no	137	Rmet_0206	*mmsA*	Op0061f_2	MET-CAH-Prop	
4065	TSS_958703+2	31	9	-36	TTGCGGAAGC	-15	AGCAGAGTCT	-140	1,1	5.69	9.80	s	1395	Rmet_0877	*0*	Op0255f_1	NA	
4066	TSS_1732589+2	31	3	-43	TTGCGGCCTG	-14	CGAATAATTG	-143	1,1	7.14	-1.60	no	510	Rmet_1607	*0*	Op0447f_1	NA	
4067	TSS_2713403+2	31	11	-26	TTGCCAGCGC	-3	GATTGGCTCT	-146	1,1	1.45	0.20	no	27	Rmet_2499	*0*	Op0693f_1	NA	
4068	TSS_3321460+2	31	7	-36	TCGCAGAGAG	-15	GATAAAATTC	-136	1,1	9.46	10.30	s	1388	Rmet_3066	*prmA*	Op0861f_4	GIP-TL-Ass	
4069	TSS_3483384+2	31	6	-41	CCTGCCGGGC	-14	ACTATCCTCT	-137	5,1	8.68	-3.60	no	19	Rmet_3210	*0*	Op0903f_2	MET-AA-Lys_Syn	
4070	TSS_3572560+2	31	5	-36	TTGACCAGGT	-15	TTGATCATGC	-140	1,1	7.93	12.80	s	13113	Rmet_3322	*0*	Op0927f_1	NA	
4071	TSS_164218-2	31	6	-33	TTGCGATAAC	-12	CTTATGGTGG	-137	1,1	7.51	9.60	s	60	Rmet_0158	*0*	Op0046r_1	NA	
4072	TSS_509547-2	31	7	-47	TGCACAGGGA	-13	CATAACATGC	-144	1,1	6.39	-11.60	no	11	Rmet_0480	*tnpA*	Op0126r_1	GIP-REP-Recomb	
4073	TSS_520591-2	31	9	-33	GCCAACAACC	-6	CGTCCGCTGG	-145	4,1	2.96	-3.80	no	679	Rmet_0492	*0*	Op0132r_1	NA	
4074	TSS_2114414-2	31	2	-36	TTGACGCACT	-15	AGTATATTAC	-140	1,1	8.52	13.80	s	1412	Rmet_1942	*acrB3*	Op0568r_1	NA	
4075	TSS_2506804-2	31	6	-37	TCCGCAACCT	-13	CGCATAATCT	-141	4,1	7.00	8.40	s	1018	Rmet_2283	*0*	Op0652r_1	NA	
4076	TSS_478137+3	31	11	-30	GCAACACAAC	-5	CCCAGATTCG	-145	4,1	6.53	-0.80	no	41	Rmet_R0079	*0*	Op1098f_1	NA	
4077	TSS_491812+3	31	10	-45	TCGCCAAGCT	-24	GCCAGATTGT	-143	1,1	6.00	1.30	w	6	Rmet_3934	*0*	Op1100f_1	EIP-SIG-2Comp	
4078	TSS_1461990+3	31	3	-34	TGGCACCCGA	-13	GGCATGATGC	-141	1,1	7.22	8.10	s	35	Rmet_4814	*rhtA*	Op1402f_1	NA	

4079	TSS_1765926+3	31	3	-41	TCGCGGTCAG	-15	GGCATAGTTC	-142	1,1	6.10	0.90	(w)-sba	453	Rmet_5079	0	Op1496f_1	NA	
4080	TSS_2322059+3	31	10	-34	TCGCCAAGCG	-13	GTAAGAATGT	-144	1,1	6.34	10.10	s	1161	Rmet_5569	0	Op1656f_1	NA	
4081	TSS_1614972-3	31	3	-25	TGGCGATTGC	-2	CGGGCAATCG	-144	1,1	0.68	-2.30	no	1379	Rmet_4948	0	Op1453r_1	NA	
4082	TSS_1762042-3	31	2	-35	TTGATATTGT	-14	AGCAAACTCG	-142	1,1	7.63	11.60	s	1119	Rmet_5072	0	Op1493r_1	MET-AA-Met	
4083	TSS_1875063-3	31	11	-35	TTGACCAGAT	-13	CGAATACTAG	-140	1,1	6.80	13.20	s	289	Rmet_6713	0	Op1525r_2	NA	
4084	TSS_2474000-3	31	10	-35	TCGACAGAGA	-13	TGAATCATGG	-145	1,1	5.57	12.70	s	400	Rmet_5714	bvgA	Op1709r_2	EIP-SIG-2Comp	
4085	TSS_4734+5	31	3	-45	CTGCGCCGAG	-14	TATATACTGA	-145	1,1	4.18	-8.60	no	749	Rmet_6319	0	Op1918f_5	NA	
4086	TSS_393406+2	31	6	-49	GTAAGGAACC	-4	AACAACATGC	-144	4,1	6.44	-29.10	no	47	Rmet_0369	0	Op0103f_2	EIP-TRA-ABC	
4087	TSS_1589916+2	31	12	-46	GCCGACCCGC	-13	GTAATACTTC	-145	4,1	6.93	-15.60	no	5735	Rmet_1475	0	Op0413f_1	NA	
4088	TSS_1684504+2	31	5	-43	TTCAACCTTT	-14	AGTACGATGA	-143	1,1	7.37	-3.10	no	505	Rmet_1564	tnpB	Op0431f_4	NA	
4089	TSS_2048380+2	31	5	-44	TTGCGCCACG	-15	GATATAGTTG	-138	1,1	8.62	-2.60	no	7080	Rmet_1888	yhiR	Op0545f_1	NA	
4090	TSS_2092911+2	31	7	-47	CCGACACGCT	-15	GCGACAATTT	-143	5,1	8.23	-11.10	no	5148	Rmet_1930	0	Op0563f_1	NA	
4091	TSS_3401202+2	31	4	-36	TCGCCGGATT	-15	GCAACAATCG	-139	1,1	6.63	10.30	s	2347	Rmet_3139	0	Op0879f_1	NA	
4092	TSS_3658876+2	31	2	-37	TGGCCGGCTG	-15	GATACATACA	-145	1,1	1.36	7.90	s	230	Rmet_3384	gspG	Op0941f_1	GIP-PTL-T2S	
4093	TSS_3713039+2	31	6	-26	CCGGCAGTCT	-3	CGCACTATAT	-143	5,1	7.39	-2.30	no	94	Rmet_3437	pat	Op0955f_1	NA	
4094	TSS_849649-2	31	8	-34	TGGCCACGCT	-13	GGCAAGGTGC	-140	1,1	5.14	8.10	s	674	Rmet_0772	0	Op0220r_1	NA	
4095	TSS_1694546-2	31	5	-42	TAGCGGTTAC	-13	GATATTCTCG	-143	1,1	4.11	-3.10	no	3916	Rmet_1569	0	Op0432r_1	NA	
4096	TSS_3618132-2	31	6	-45	GCAAAGCCGG	-22	TTAACAATCA	-143	4,1	6.48	-3.60	no	179	Rmet_R0064	0	Op0930r_1	NA	
4097	TSS_3866995-2	31	5	-33	GCGGAAGCGG	-13	GCTAAGGTTA	-142	5,1	7.52	3.10	w	30	Rmet_3564	fmt	Op0988r_1	MET-AA-Met	
4098	TSS_152329+3	31	1	-46	GAACTCGGCA	-26	GTAACTTCGG	-146	3,0	6.42	-10.20	no	1299	Rmet_R0074	0	Op1006f_1	NA	
4099	TSS_857244+3	31	3	-37	TGGTGATTGC	-14	CGTACACTGG	-136	1,1	7.88	8.90	s	625	Rmet_4267	ycaC	Op1212f_1	NA	
4100	TSS_921771+3	31	8	-49	TGCCGTCGGT	-15	GGTACGATTT	-142	1,1	6.87	-16.60	no	937	Rmet_4329	0	Op1232f_1	NA	
4101	TSS_2395662+3	31	12	-36	TGGCTTCCAC	-15	CGTAACATCG	-140	1,1	6.20	9.30	s	4796	Rmet_6755	0	Op1682f_1	NA	
4102	TSS_1874840-3	31	11	-40	TCCACGTACT	-12	GTTAAGGTGC	-141	1,1	5.08	-0.60	no	66	Rmet_6713	0	Op1525r_2	NA	
4103	TSS_36513+5	31	4	-40	TTGCCGGATA	-14	CCGATACTAC	-140	1,1	6.78	4.40	(m)-sba	2934	Rmet_6189	0	Op1894f_4	NA	
4104	TSS_132093+2	30	2	-35	TTCTGAAATC	-15	GGTAGTGTCG	-144	1,1	4.82	8.10	s	2881	Rmet_0129	dehH2	Op0033f_1	MET-XEN-Hex	
4105	TSS_436711+2	30	8	-48	TTCTAATGCG	-21	CGGATTCTTG	-146	1,1	3.82	-7.10	no	131	Rmet_6415	0	Op0113f_1	NA	
4106	TSS_578611+2	30	9	-30	TTGCGGGGGC	-3	AGAATAATTC	-141	1,1	7.80	2.20	(w)-nu	61	Rmet_0543	0	Op0151f_1	NA	
4107	TSS_3634783+2	30	9	-41	TTCCGGCCGA	-14	AGTACTATCT	-139	1,1	6.29	-0.10	no	856	Rmet_3360	0	Op0935f_3	NA	
4108	TSS_3644416+2	30	5	-27	TTCAGAACTA	-7	TACACACTTG	-144	1,1	6.03	1.10	(w)-nu	1381	Rmet_3369	paaA	Op0937f_1	NA	
4109	TSS_1338377-2	30	14	-35	TGGCAACCCA	-10	TCGAACTTCC	-143	1,1	2.90	5.70	m	616	Rmet_1219	0	Op0354r_2	NA	
4110	TSS_1775464-2	30	4	-27	CCGGCCGGAT	-2	CATACAATCC	-142	5,1	11.61	-2.30	no	478	Rmet_1641	0	Op0462r_3	NA	
4111	TSS_3077707-2	30	10	-46	TTTCTTCGGG	-26	GACATATTTT	-140	2,1	11.17	-1.70	no	7	Rmet_2828	0	Op0796r_1	NA	
4112	TSS_3412061-2	30	10	-33	TTCATCTGCA	-13	GATAACCTAC	-142	1,1	5.67	6.10	m	4850	Rmet_3143	tnmB	Op0880r_1	NA	
4113	TSS_3428712-2	30	2	-33	GCACGAATGG	-13	GCCACACTGT	-142	4,1	8.40	1.60	w	1026	Rmet_R0050	0	Op0886r_1	NA	
4114	TSS_152640+3	30	4	-28	TCAGCGAAGT	-8	GTGATGATGC	-144	4,1	6.85	0.60	(w)-nu	988	Rmet_R0074	0	Op1006f_1	NA	
4115	TSS_577782+3	30	6	-40	GCTTACGGAC	-13	ATTACTATGA	-144	5,1	6.51	-3.60	no	4466	Rmet_6640	0	Op2063f_1	NA	
4116	TSS_963495+3	30	11	-38	TCGTCAATGA	-14	TTTATGCTGT	-147	1,1	6.49	7.90	s	149	Rmet_4366	0	Op1246f_1	NA	
4117	TSS_1059448+3	30	6	-41	TGGCGACAAA	-15	TGTAGAATCT	-141	1,1	8.67	2.90	(w)-sba	89	Rmet_4462	hns	Op1272f_1	NA	
4118	TSS_1083261-3	30	5	-34	TCGATATTAT	-13	TATATAATTC	-143	1,1	9.70	12.10	s	29	Rmet_4482	0	Op1277r_1	NA	
4119	TSS_1508813-3	30	7	-39	TCGGCATGAT	-18	CCTATGCTAG	-140	5,1	8.07	8.30	s	43	Rmet_4850	0	Op1415r_1	NA	
4120	TSS_2033229-3	30	6	-38	TCGAACGGCA	-14	GGTAATGTAT	-144	1,1	4.74	5.90	m	29	Rmet_5322	zniS	Op1573r_1	NA	
4121	TSS_2250105-3	30	2	-35	TGGCGCCGTC	-15	TCTAGAATCT	-137	1,1	8.23	9.10	s	1619	Rmet_5504	0	Op1637r_1	NA	
4122	TSS_2258171-3	30	5	-35	TGGACGGCCT	-13	TCCAGAATGC	-139	1,1	7.30	11.70	s	4156	Rmet_5508	0	Op1639r_1	NA	
4123	TSS_2498784-3	30	5	-32	TCGCGATGGC	-12	GGGATGATGC	-141	1,1	7.23	6.10	m	5977	Rmet_5732	kpsM	Op1715r_1	NA	
4124	TSS_198763+4	30	5	-42	TCGAGGCTAC	-14	GGCATATTGT	-141	1,1	6.28	-1.10	no	523	Rmet_6383	0	Op1861f_1	NA	
4125	TSS_99988-5	30	10	-48	GCTGCAAGAT	-13	TTTAAAATCG	-137	5,1	11.93	-16.60	no	5518	Rmet_6246	0	Op1903r_1	NA	
4126	TSS_507716+2	30	6	-40	GCAGCCCTCG	-15	CAGAGAATTA	-145	5,1	6.85	-0.60	no	1871	Rmet_0481	0	Op0127f_1	NA	
4127	TSS_844418+2	30	3	-47	TCGGACACGC	-16	GGCACAATCG	-140	5,1	9.54	-10.10	no	14	Rmet_0767	phnF	Op0219f_1	NA	
4128	TSS_900885+2	30	13	-34	TTCATATGTC	-14	TGCATCCTCA	-144	1,1	4.88	8.10	s	187	Rmet_0827	cvrA	Op0235f_1	NA	
4129	TSS_1600839+2	30	3	-35	TAGCCAAAGC	-14	GCGATGCTCG	-146	1,1	3.25	10.10	s	936	Rmet_1480	0	Op0417f_1	NA	
4130	TSS_2264452+2	30	1	-40	TGGACATGTC	-2	GCAACGATGC	-144	1,1	6.44	-8.10	no	216	Rmet_2086	narX	Op0603f_1	EIP-SIG-2Comp	
4131	TSS_2628669+2	30	13	-36	TTCAACGCCG	-12	CCCACAATGA	-142	1,1	6.50	8.90	s	2877	Rmet_6524	0	Op2034f_1	NA	
4132	TSS_3469057+2	30	7	-38	TCGTAAGCCA	-15	TTTACCATGA	-141	1,1	5.90	7.90	s	4141	Rmet_3198	mtnN	Op0899f_1	MET-AA-Met	
4133	TSS_22003-2	30	2	-42	TTGAGGAGTG	-14	TCTACGCTCT	-140	1,1	7.52	-0.60	no	4700	Rmet_0012	0	Op0002r_1		0
4134	TSS_307550-2	30	14	-35	TGGACAGCCT	-14	AGTACCGTCA	-140	1,1	5.04	11.10	s	297	Rmet_0291	lolB	Op0084r_1	MET-COF-Porph	
4135	TSS_944940-2	30	3	-47	GGACAGACGC	-12	GCTATTTTAG	-141	4,1	8.14	-18.60	no	1751	Rmet_6431	0	Op0248r_1	NA	
4136	TSS_2672980-2	30	7	-44	GCCGCGACGT	-24	GGCAGTCTGC	-141	4,1	5.62	-5.20	no	23	Rmet_2461	cvpA	Op0688r_5	NA	
4137	TSS_255119+3	30	8	-36	TTGACAATAG	-12	TGCACTATCA	-144	1,1	7.22	11.40	s	51	Rmet_3713	0	Op1030f_1	NA	
4138	TSS_287574+3	30	11	-35	TGGCCACGGC	-14	AGTACGATCA	-137	1,1	7.56	11.10	s	4485	Rmet_6622	0	Op1044f_1	NA	
4139	TSS_447540+3	30	8	-36	TGCAGACAGC	-14	GATACGATGA	-142	1,1	6.80	10.40	s	1783	Rmet_3904	yeaG	Op1092f_1	NA	

4140	TSS_452373+3	30	10		-34	TGGAACATCT	-9	CCGATGGTGA	-143	1,1	3.62	5.70	*(m)-ba*	278	Rmet_3906	*0*	Op1092f_1	NA
4141	TSS_1224410+3	30	6		-41	TTGATGCCCA	-13	CTTAACGTGC	-145	1,1	5.36	0.40	*(w)-sba*	412	Rmet_4603	*0*	Op1324f_2	NA
4142	TSS_18693-3	30	8		-36	TTGTACGATG	-14	AATACCATGA	-140	1,1	6.81	11.40	s	700	Rmet_5829	*pstS1*	Op1763r_1	EIP-TRA-ABC
4143	TSS_726261-3	30	11		-35	TTGCGCGTGG	-14	GCGACACTAA	-144	1,1	6.46	9.60	s	605	Rmet_4149	*crtB*	Op1169r_3	MET-LIP-Ster
4144	TSS_1328891-3	30	10		-34	TTGACGCGGG	-13	CCGACACTTC	-139	1,1	7.09	10.60	s	87	Rmet_4692	*0*	Op1357r_1	NA
4145	TSS_1762278-3	30	7		-29	TTCTCGAACA	-8	ATTACGCTCT	-148	1,1	4.96	3.10	*(w)-nu*	34	Rmet_5073	*metE*	Op1493r_1	NA
4146	TSS_2213953-3	30	8		-49	GGAAACAAGC	-13	GTTAACATGG	-140	4,1	10.61	-20.60	*no*	400	Rmet_5468	*0*	Op1625r_2	NA
4147	TSS_98673+5	30	2		-47	GCAGCAGTTT	-25	GGGATCTTAT	-146	5,1	5.10	-4.60	*no*	74	Rmet_6252	*int*	Op1904f_4	NA
4148	TSS_362478+2	30	9		-36	TTGCGCTGCC	-15	CCTATACTCA	-139	1,1	9.05	11.80	s	34	Rmet_0344	*0*	Op0097f_1	NA
4149	TSS_1012530+2	30	7		-44	GCAACAGGAC	-13	GACAACATTC	-142	5,1	6.21	-9.60	*no*	16	Rmet_0927	*nuoA*	Op0267f_3	MET-EN-OxPhos
4150	TSS_1334499+2	30	1		-42	GGCGCGACAC	-14	ATCAGAATGC	-141	4,1	7.37	-5.60	*no*	9103	Rmet_1224	*bzdA*	Op0355f_1	MET-XEN-Benz
4151	TSS_3624276+2	30	9		-36	TCCACAGTAG	-14	GATAGACTTT	-143	1,1	6.64	11.40	s	4122	Rmet_3352	*0*	Op0933f_1	NA
4152	TSS_945007-2	30	1		-33	TGGCACCCAG	-13	ACGATAATCC	-141	1,1	6.71	7.10	s	1818	Rmet_6431	*0*	Op0248r_1	NA
4153	TSS_1000425-2	30	10		-34	TTCACGGGCG	-13	AGGATAATTC	-141	1,1	7.30	11.10	s	3997	Rmet_0910	*rpoR*	Op0262r_1	GIP-TK-RNAP
4154	TSS_2046471-2	30	8		-41	TTCACCATTG	-21	CCACTGATTG	-146	1,1	0.06	3.30	*m*	0	Rmet_R0020	*0*	Op0544r_2	NA
4155	TSS_2791103-2	30	7		-33	AATGGCACCG	-13	GTCATAATGC	-138	2,1	6.85	2.60	w	47	Rmet_2565	*0*	Op0710r_2	NA
4156	TSS_2961274-2	30	4		-40	TGCCACCATA	-14	TGGATAATGG	-145	1,1	6.25	1.40	*(w)-sba*	255	Rmet_2724	*0*	Op0770r_2	NA
4157	TSS_3601641-2	30	11		-37	TCGCCACGTC	-13	TGTATCGTCG	-139	1,1	6.01	8.90	s	3501	Rmet_3332	*0*	Op0930r_4	NA
4158	TSS_214125+3	30	7		-47	GCGGCATGAC	-14	GATATAGTGT	-140	5,1	9.89	-12.10	*no*	1267	Rmet_3678	*0*	Op1026f_1	DIV-MOT-Chemotax
4159	TSS_744296+3	30	1		-21	TTGCGTTATA	-1	CGTAAACTTG	-140	1,1	8.83	-4.40	*no*	251	Rmet_4162	*0*	Op1170f_4	NA
4160	TSS_897406+3	30	7		-41	TCGACCTGTT	-14	CTTATCATCT	-141	1,1	7.74	2.90	*(w)-sba*	383	Rmet_4303	*0*	Op1226f_1	EIP-TRA-Ion
4161	TSS_1165327+3	30	3		-39	TGCAAGGGGA	-15	TCTACGATAC	-141	1,1	6.11	4.40	m	394	Rmet_4553	*0*	Op1306f_3	NA
4162	TSS_1273150+3	30	8		-34	TTGTCCCGCG	-6	GACATATTTC	-142	1,1	6.54	5.20	*(m)-ba*	4014	Rmet_4647	*0*	Op1342f_1	NA
4163	TSS_2335479+3	30	8		-46	CCAGCACCGT	-15	TGTAAGATCA	-138	5,1	8.97	-11.60	*no*	699	Rmet_5582	*0*	Op1662f_1	NA
4164	TSS_2471307+3	30	3		-39	GCGGCATCAT	-19	GCCATGCTAC	-142	5,1	7.04	3.30	*(m)-ba*	705	Rmet_5712	*0*	Op1708f_1	NA
4165	TSS_306259-3	30	8		-39	GGAAAGCCCT	-13	GGTATTCTTT	-140	4,1	7.80	-0.60	no	431	Rmet_3768	*0*	Op1047r_1	NA
4166	TSS_373274-3	30	3		-43	GCACCACAGC	-14	CGTATGATCG	-139	4,1	9.60	-5.60	*no*	5798	Rmet_3825	*0*	Op1063r_1	NA
4167	TSS_725804-3	30	5		-44	TCGACCAGAC	-13	CGTACCATAC	-138	1,1	7.21	-5.10	*no*	148	Rmet_4149	*crtB*	Op1169r_3	MET-LIP-Ster
4168	TSS_1084568-3	30	2		-36	TCGACGCCTG	-13	CGTATCATTG	-139	1,1	7.43	11.90	s	1336	Rmet_4482	*0*	Op1277r_1	NA
4169	TSS_2234743-3	30	5		-46	TGGCGAGCAG	-13	AGTATGCTGC	-142	1,1	6.89	-10.10	*no*	2222	Rmet_5488	*0*	Op1629r_1	GIP-REP-Recomb
4170	TSS_168587+4	30	1		-39	TTGCTGATGA	-13	GGTAATATGC	-137	1,1	9.29	4.40	m	2112	Rmet_6132	*0*	Op1873f_1	NA
4171	TSS_200758-4	30	11		-45	CCGGATCGGT	-13	ATTAAGATCA	-142	5,1	7.30	-12.10	*no*	200	Rmet_6384	*0*	Op1860r_1	NA
4172	TSS_106187+5	30	5		-41	TTGCCATGTC	-13	CGTACCATGT	-137	1,1	9.48	2.40	*(w)-sba*	4582	Rmet_6258	*rhsC*	Op1906f_1	NA
4173	TSS_128691-5	30	10		-43	TGGCTAGGAT	-13	AGTAGAGTTT	-141	1,1	6.26	-4.10	*no*	412	Rmet_6272	*0*	Op1913r_2	NA
4174	TSS_263937+2	29	8		-36	TTACAAGTGC	-13	GTTATCATTT	-147	4,1	9.12	10.90	s	67	Rmet_0247	*bfd*	Op0071f_3	NA
4175	TSS_1176607+2	29	9		-36	CCGGCCGCCG	-14	GGTAAGATAT	-136	5,1	9.97	7.90	s	1465	Rmet_1086	*0*	Op0313f_1	GIP-REP-Recomb
4176	TSS_1578895+2	29	6		-35	TCGGCCACTC	-14	TGTACGATCT	-140	5,1	9.03	10.10	s	360	Rmet_1458	*yfjF*	Op0407f_2	NA
4177	TSS_2009376+2	29	7		-48	TCGATCACCA	-15	GCCATGATTC	-140	1,1	6.22	-12.10	*no*	2573	Rmet_1856	*lysP*	Op0537f_1	EIP-TRA-Ion
4178	TSS_2291498+2	29	3		-35	TTGAGCAGCG	-14	CCCATCATCA	-140	1,1	6.61	11.60	s	1303	Rmet_2111	*0*	Op0609f_1	NA
4179	TSS_2729771+2	29	9		-40	TTAAGGCAGG	-14	CTTACAATCC	-143	4,1	7.58	3.90	*(m)-sba*	220	Rmet_2515	*lldP*	Op0695f_3	EIP-TRA-Ion
4180	TSS_3470029+2	29	6		-36	TTCATCCGCG	-14	CGTACGATGA	-139	1,1	6.48	10.90	s	3169	Rmet_3198	*mtnN*	Op0899f_1	MET-AA-Met
4181	TSS_278090-2	29	7		-36	TTCCGTCGGT	-14	TTCATAATCC	-138	1,1	7.56	10.90	s	1463	Rmet_0258	*gntX*	Op0074r_1	NA
4182	TSS_489264-2	29	8		-49	TCGCCACTGT	-24	TTGAAGATCG	-142	1,1	5.41	-5.10	*no*	1877	Rmet_0458	*uspA1*	Op0120r_1	NA
4183	TSS_769784-2	29	3		-32	TCACCAGTAC	0	GACAAAATTT	-147	4,1	8.01	-2.80	*no*	113	Rmet_0697	*pilA*	Op0196r_2	GIP-PTL-T2S
4184	TSS_976210-2	29	2		-36	CCGGCATCAC	-13	GCTATGATTG	-137	5,1	10.37	8.90	s	129	Rmet_0890	*add*	Op0258r_2	MET-NUC-Pur
4185	TSS_1249382-2	29	6		-35	TTGACGCGGC	-14	TGCAGAATCA	-138	1,1	8.20	12.60	s	39	Rmet_1141	*0*	Op0326r_1	NA
4186	TSS_2190204-2	29	7		-33	TGGCGCGGGC	-13	CCGACACTTT	-141	1,1	5.33	5.10	m	246	Rmet_2024	*bipA*	Op0592r_1	NA
4187	TSS_2302542-2	29	3		-43	TCGAACATGG	-13	GGTATGCTGC	-140	1,1	8.17	-4.10	*no*	1975	Rmet_2118	*surE*	Op0612r_1	MET-NUC-Pur
4188	TSS_2390257-2	29	5		-42	GCGGCAAGTC	-13	AATAAGATGT	-139	5,1	9.37	-4.10	*no*	662	Rmet_2181	*phoU*	Op0628r_4	EIP-TRA-ABC
4189	TSS_3134296-2	29	9		-34	TTGCGCGATT	-13	GGCAGACTTG	-141	1,1	7.78	8.60	s	4265	Rmet_2878	*purN*	Op0808r_1	NA
4190	TSS_3570670-2	29	6		-40	TCGCTGCTGA	-13	GGTAACCTGA	-145	1,1	5.87	-0.10	*no*	31	Rmet_3296	*infA*	Op0926r_2	NA
4191	TSS_3858374-2	29	9		-42	TCGCGGTGTA	-13	GGCATCATGT	-141	1,1	6.65	-3.10	*no*	2564	Rmet_3555	*0*	Op0986r_1	NA
4192	TSS_1096946+3	29	7		-40	TCGACGGCAG	-15	GATACCCTCC	-139	1,1	6.26	3.90	*(m)-sba*	4474	Rmet_4500	*0*	Op1282f_1	NA
4193	TSS_1473554+3	29	7		-45	TTGTGTTATC	-15	GATAAACTTC	-142	1,1	8.67	-5.60	*no*	2160	Rmet_4823	*0*	Op1406f_1	NA
4194	TSS_1856744+3	29	2		-42	TGGAGGCATC	-13	AATAGAATAC	-140	1,1	7.43	-1.10	*no*	23	Rmet_5158	*0*	Op1518f_1	NA
4195	TSS_2214785+3	29	5		-29	TTCCTGAGCC	-5	GAGATTCTCC	-149	1,1	4.23	0.70	*(w)-nu*	430	Rmet_6729	*0*	Op2094f_1	NA
4196	TSS_1359417-3	29	14		-41	TCATAATCAT	-13	TATATAATTA	-149	5,1	7.04	0.40	*(w)-sba*	69	Rmet_6680	*0*	Op1361r_1	NA
4197	TSS_1624982-3	29	11		-35	TTGCAGTCGC	-14	GGCACGATCA	-138	1,1	7.67	9.60	s	3945	Rmet_4954	*0*	Op1455r_1	NA
4198	TSS_1735589-3	29	5		-31	GAACCTGCCG	-11	CCTCCAAGTA	-143	3,0	4.99	-2.40	no	14	Rmet_5047	*hutH*	Op1485r_3	MET-EN-Nitrog
4199	TSS_1912936-3	29	7		-34	ATGCGTTCCG	-13	CTTATGATCG	-143	2,1	9.68	7.60	s	370	Rmet_5217	*0*	Op1537r_1	NA
4200	TSS_41630+4	29	4		-43	TGGCGGCGTC	-14	TCTACACTCG	-140	1,1	7.72	-4.10	*no*	534	Rmet_6015	*0*	Op1835f_1	NA

4201	TSS_49088-4	29	9	-45	GCACGGATGT	-11	CGTAGAATGG	-140	4,1	10.13	-14.60	*no*	10473	Rmet_6359	*0*	Op2108r_1	NA
4202	TSS_140350-5	29	7	-34	TTGCCCCACG	-13	GGCAGCTTGT	-142	1,1	5.00	8.60	s	1875	Rmet_6282	*tnpA*	Op1915r_1	NA
4203	TSS_326244+2	29	2	-36	TTGTTGGCCT	-15	CGCACGATCT	-138	1,1	5.16	9.80	s	424	Rmet_0311	*apt*	Op0089f_1	MET-NUC-Pur
4204	TSS_508141+2	29	5	-36	TCAACGAAAG	-14	GGTACGATGG	-143	4,1	8.67	10.40	s	1446	Rmet_0481	*0*	Op0127f_1	NA
4205	TSS_674997+2	29	6	-47	GCAAAAGACG	-14	GCCAAGATGG	-141	4,1	8.16	-15.60	*no*	11772	Rmet_0626	*0*	Op0179f_1	NA
4206	TSS_900313+2	29	9	-36	TTGATGCCGT	-15	CCCATGCTGA	-141	1,1	5.55	10.80	s	759	Rmet_0827	*cvrA*	Op0235f_1	NA
4207	TSS_1562622+2	29	11	-37	GCAGCCGGCG	-15	GATACGCTCG	-137	5,1	8.88	4.40	m	109	Rmet_1442	*yaeL*	Op0403f_2	NA
4208	TSS_1880843+2	29	3	-47	GCAACCCAAA	-14	GGTAATATCG	-142	4,1	7.39	-14.60	*no*	345	Rmet_1736	*cybB*	Op0493f_1	NA
4209	TSS_2622640+2	29	5	-37	TGGCGGCGAA	-3	GGTAAAATAG	-139	1,1	9.06	-2.10	*no*	135	Rmet_2412	*efp*	Op0679f_2	NA
4210	TSS_3303959+2	29	5	-45	TGGTCGGGGT	-14	GGGAAACTCG	-139	1,1	5.96	-8.10	*no*	340	Rmet_3047	*thiL*	Op0857f_1	MET-COF-Thia
4211	TSS_3424133+2	29	8	-35	TCGCCCCATC	-15	GGCATCATGA	-141	1,1	6.52	9.10	s	1224	Rmet_3160	*0*	Op0885f_1	NA
4212	TSS_3637391+2	29	10	-37	TGGCTCACTT	-15	GTCACCATAG	-141	1,1	5.29	7.90	s	470	Rmet_3362	*0*	Op0935f_3	NA
4213	TSS_785737-2	29	2	-29	TGCTAACGAC	-5	GGCAGCCTAA	-147	1,1	2.16	-0.80	*no*	721	Rmet_0708	*0*	Op0200r_1	NA
4214	TSS_1859822-2	29	4	-34	TTGACCGTGA	-13	CCCATCATCG	-138	1,1	7.39	11.60	s	2192	Rmet_1715	*0*	Op0484r_1	NA
4215	TSS_2294998-2	29	12	-34	TGGACTACAA	-12	AAAACAATAC	-144	1,1	4.94	10.70	s	2537	Rmet_2110	*ndk*	Op0608r_1	MET-NUC-Pur
4216	TSS_2361024-2	29	4	-42	TGGAACTTCC	-13	GGCAAACTAT	-142	1,1	6.64	-3.10	*no*	3602	Rmet_6509	*0*	Op2029r_1	NA
4217	TSS_2896880-2	29	8	-34	TTCAGCTTCC	-13	CGTATCGTGG	-141	1,1	5.91	9.10	s	924	Rmet_2661	*0*	Op0754r_3	NA
4218	TSS_3507400-2	29	3	-40	TTGAACTCGC	-14	GCTATTCTCC	-140	1,1	9.01	4.40	*(m)-sba*	201	Rmet_3230	*petA*	Op0914r_1	MET-EN-OxPhos
4219	TSS_3519971-2	29	14	-47	GCCCACGCGC	-13	GTTAAGCTGG	-140	4,1	8.48	-18.60	*no*	27	Rmet_3247	*hisC*	Op0918r_3	MET-AA-His
4220	TSS_3591335-2	29	12	-45	TCGAAGTACG	-14	CCTAAGCTTC	-140	1,1	6.14	-8.10	*no*	197	Rmet_3326	*rpsG*	Op0928r_2	GIP-TL-Ribo
4221	TSS_3837964-2	29	3	-29	TTCCAGAACG	-8	ATTAAATTCA	-148	1,1	5.96	3.10	*(w)-nu*	1024	Rmet_3539	*0*	Op0980r_1	NA
4222	TSS_383774+3	29	5	-43	TTGGCAACGAG	-15	GATATGATTT	-141	1,1	8.68	-1.10	*no*	77	Rmet_3840	*0*	Op1066f_2	NA
4223	TSS_1361272+3	29	10	-43	TGGCACGCCG	-15	ATCATACTGA	-139	1,1	5.56	-3.10	*no*	414	Rmet_4719	*0*	Op1362f_1	NA
4224	TSS_2051406+3	29	7	-37	TTGACGCCCG	-8	GTTCGGATAC	-145	1,1	2.52	3.40	*(m)-ba*	11359	Rmet_5342	*0*	Op1584f_1	NA
4225	TSS_2186734+3	29	13	-44	TTCCGACGAC	-15	CTGACAATGC	-140	1,1	6.87	-4.10	*no*	36	Rmet_5438	*0*	Op1618f_1	MET-LIP-FAMet
4226	TSS_2251698+3	29	5	-29	TTGTGGATGT	-6	TCCACTATGG	-144	1,1	6.46	3.20	*(w)-nu*	2405	Rmet_5509	*0*	Op1640f_1	NA
4227	TSS_531789-3	29	3	-42	TCGCGAGCGC	-13	ATTACTATCT	-140	1,1	6.33	-2.10	*no*	65	Rmet_3968	*0*	Op1113r_2	NA
4228	TSS_603037-3	29	10	-42	TGCTCCGGTG	-20	GAAAAAATGT	-145	1,1	4.13	3.40	*m*	616	Rmet_4033	*0*	Op1137r_1	NA
4229	TSS_1154154-3	29	9	-35	TTGCGCAGGC	-10	ACGATGATGG	-138	1,1	7.12	8.20	s	313	Rmet_4547	*0*	Op1303r_1	NA
4230	TSS_1291884-3	29	1	-41	TCGTCCAGAC	-13	GCTAAAGTCG	-141	1,1	6.77	-0.10	*no*	733	Rmet_4657	*0*	Op1347r_1	NA
4231	TSS_1455123-3	29	12	-33	TTGCACTGCG	-12	TCCACAATGG	-140	1,1	8.93	8.60	s	1579	Rmet_4805	*kbl*	Op1397r_1	MET-AA-GlySerThr
4232	TSS_2155167-3	29	12	-47	GGAGGACCGA	-25	GCTACAATTT	-140	4,1	9.64	-3.60	*no*	9	Rmet_5412	*0*	Op1611r_1	NA
4233	TSS_154117+4	29	8	-45	CCGGCAGGCC	-14	GCTACGATCG	-137	5,1	10.79	-9.10	*no*	850	Rmet_6144	*nreB*	Op1879f_1	EIP-SIG-2Comp
4234	TSS_223914+4	29	4	-43	TGGCACTTGC	-23	TTTAACGTGG	-141	1,1	5.73	0.30	*w*	676	Rmet_6077	*0*	Op1851f_1	NA
4235	TSS_34492-4	29	7	-48	TTGAAGTCGA	-13	CCCATACTTG	-143	1,1	8.06	-12.60	*no*	554	Rmet_6026	*0*	Op1836r_1	NA
4236	TSS_75063-4	29	5	-36	TCGATCACCA	-13	ATTACGATTA	-144	1,1	5.80	9.90	s	1103	Rmet_5985	*czcM*	Op1822r_1	EIP-TRA-Other
4237	TSS_144585+2	29	14	-36	TTGCCTGTCC	-14	GTCATGATAC	-138	1,1	7.54	12.40	s	1008	Rmet_0141	*0*	Op0039f_1	NA
4238	TSS_1343405+2	29	4	-48	TTCAGGGCGG	-14	GGCATTATAC	-141	1,1	6.01	-13.10	*no*	197	Rmet_1224	*bzdA*	Op0355f_1	MET-XEN-Benz
4239	TSS_2190731+2	29	8	-29	TTGAAGCAAG	-6	CACACAATGA	-142	1,1	7.82	5.20	*(m)-nu*	1560	Rmet_2027	*emrA*	Op0593f_1	EIP-TRA-Other
4240	TSS_2298012+2	29	7	-44	TTGCGGACCT	-15	GTCAGAATCA	-140	1,1	8.04	-3.60	*no*	2622	Rmet_2119	*0*	Op0613f_1	NA
4241	TSS_2557532+2	29	5	-35	TTGCCGGGCA	-14	ATCACCATGT	-141	1,1	6.38	10.60	s	1871	Rmet_2345	*0*	Op0663f_1	NA
4242	TSS_3134362+2	29	10	-47	TTGCAGAAAT	-13	CATATCATGC	-141	1,1	9.45	-10.60	*no*	1318	Rmet_2883	*0*	Op0811f_1	GIP-REP-Recomb
4243	TSS_3528285+2	29	8	-20	TCGAGCGTAG	0	GCGATTCTCT	-143	1,1	4.25	-6.90	*no*	3084	Rmet_3261	*0*	Op0919f_1	NA
4244	TSS_697252-2	29	10	-47	TCGGCCTCGG	-13	GGTAGGCTTT	-140	5,1	7.45	-13.10	*no*	10772	Rmet_0625	*0*	Op0178r_1	NA
4245	TSS_933647-2	29	5	-45	TGCCGATAAC	-23	GCAATGATGT	-142	1,1	4.96	0.40	*w*	255	Rmet_0852	*rph*	Op0244r_2	GIP-TL-Ass
4246	TSS_1155378-2	29	2	-49	TGGAACGCAT	-27	GGAACGAAGC	-145	1,1	0.48	-5.10	*no*	193	Rmet_1061	*0*	Op0304r_1	MET-CAH-FbP
4247	TSS_1266026-2	29	9	-39	TGGACTCCCT	-13	GGCATGATGG	-139	1,1	7.17	4.90	m	146	Rmet_1156	*bdhA*	Op0332r_1	MET-CAH-But
4248	TSS_1418553-2	29	6	-43	TCCCCCGATT	-18	TTTAGAGTCC	-144	1,1	4.98	-0.60	*no*	15	Rmet_1303	*tnpA*	Op0374r_1	GIP-REP-Recomb
4249	TSS_1819053-2	29	9	-39	TCGCGCCTTC	-14	TTTAAAATCT	-140	1,1	8.12	4.90	m	106	Rmet_1681	*aroG*	Op0474r_1	MET-AA-PheTyrTrp
4250	TSS_1891380-2	29	2	-35	TCGCACGGCT	-13	GCCAGAATGC	-140	1,1	7.62	9.70	s	3409	Rmet_1740	*0*	Op0494r_1	NA
4251	TSS_2526632-2	29	2	-48	CCTGCACCGT	-22	CGGATCGTCC	-144	5,1	5.51	-9.60	*no*	3500	Rmet_2302	*pbrR2*	Op0658r_1	NA
4252	TSS_2685352-2	29	6	-33	TTCACATCAC	-12	GTCATGATTG	-138	1,1	7.65	10.10	s	1428	Rmet_2470	*0*	Op0688r_3	NA
4253	TSS_3570981-2	29	2	-42	TTTCTTCTGC	-20	GCTCTGGTGT	-146	2,1	4.77	1.90	*w*	342	Rmet_3296	*infA*	Op0926r_2	NA
4254	TSS_3909910-2	29	6	-34	TGAAAAAAAA	-13	GGCAGAATCG	-141	4,1	8.17	8.60	s	69	Rmet_3597	*0*	Op1000r_1	NA
4255	TSS_1098325+3	29	6	-33	GAACTTCGCG	-13	TATCGAACGA	-144	3,0	5.82	-1.40	no	3095	Rmet_4500	*0*	Op1282f_1	NA
4256	TSS_1899484+3	29	5	-45	TCGTCACCGC	-15	GCTACGATCC	-139	1,1	7.54	-5.10	*no*	741	Rmet_5206	*amnC*	Op1534f_2	NA
4257	TSS_39467-3	29	7	-20	TTCAGGAACT	0	GTTTTTCTCG	-147	1,1	0.97	-7.90	*no*	6970	Rmet_5842	*0*	Op1765r_1	NA
4258	TSS_308332-3	29	7	-34	TGCAGATTTC	-12	GCGACAATAC	-143	1,1	6.39	9.20	s	1835	Rmet_3769	*alkB1*	Op1047r_1	NA
4259	TSS_451173-3	29	4	-41	TCCACCAAAA	-13	TGTATCCTTT	-142	1,1	5.69	-0.60	*no*	2000	Rmet_3903	*0*	Op1091r_1	NA
4260	TSS_1092457-3	29	6	-46	TCGGACAGCG	-25	GAAATCGTTT	-144	5,1	4.96	-1.70	*no*	208	Rmet_4492	*0*	Op1279r_1	NA
4261	TSS_9976+4	29	3	-41	TCGAGAGTCA	-15	GTTAGAATTC	-141	1,1	9.13	3.90	*(m)-sba*	42	Rmet_6057	*0*	Op1845f_1	NA

4262	TSS_20402-4	29	9	-29	TTCTCTTGTT	-7	CACAGTATTC	-145	1,1	3.97	3.70	(m)-nu	4197	Rmet_6050	0	Op1844r_1	NA
4263	TSS_916-5	29	9	-47	GGCGAGGAGC	-13	GAGAACATCG	-143	4,1	6.38	-18.60	no	15016	Rmet_6301	trbF	Op1917r_1	NA
4264	TSS_347086+2	28	8	-35	TGGCGCCGCG	-14	CATAGAGTGC	-139	1,1	5.63	9.10	s	8763	Rmet_0338	0	Op0095f_1	NA
4265	TSS_942929+2	28	10	-36	TTCTGATGTT	-14	GCGAAACTCA	-146	1,1	5.46	9.90	s	119	Rmet_0864	0	Op0249f_1	NA
4266	TSS_966781+2	28	8	-36	TTGACAATCT	0	GTCAGATTGT	-143	1,1	7.58	-0.60	no	2747	Rmet_0885	0	Op0257f_1	NA
4267	TSS_1012441+2	28	3	-27	AGATGCGTCG	-7	CGGCAGTCCG	-144	3,0	5.71	-10.40	no	105	Rmet_0927	nuoA	Op0267f_3	MET-EN-OxPhos
4268	TSS_1193778+2	28	4	-23	TTGTGATCGA	0	GACAACATTT	-142	1,1	6.44	-1.80	no	1161	Rmet_1098	cobW	Op0317f_1	NA
4269	TSS_2836006+2	28	4	-39	TTGCCGCACG	-14	GGTATTCTGC	-136	1,1	8.84	6.40	s	2420	Rmet_6542	0	Op2038f_1	NA
4270	TSS_2910048+2	28	4	-26	TTGCAAAACA	-6	GACACGGTTC	-142	1,1	5.79	-0.40	no	2834	Rmet_2680	glyA	Op0757f_1	MET-EN-Methane
4271	TSS_3624176+2	28	4	-39	TCCGCCAAGT	-14	CGTACAATTT	-141	4,1	8.34	4.40	m	4222	Rmet_3352	0	Op0933f_1	NA
4272	TSS_3901159+2	28	3	-45	TCGACTACGG	-25	TCGAACCTGG	-142	1,1	3.80	-0.70	no	9026	Rmet_3598	0	Op1001f_1	NA
4273	TSS_788072-2	28	3	-22	GGAGAAATAT	-2	GCCAAGCTCG	-146	4,1	7.19	-10.40	no	739	Rmet_0711	ubiG	Op0202r_2	MET-COF-CoQ
4274	TSS_823506-2	28	5	-27	GCTGCCGCCC	0	CACAGCGTGC	-143	5,1	4.68	-8.80	no	170	Rmet_6427	0	Op0210r_1	NA
4275	TSS_875197-2	28	2	-41	TTCACGTCAA	-14	GCTATTATAC	-141	1,1	8.26	2.90	(w)-sba	224	Rmet_0798	0	Op0226r_1	NA
4276	TSS_1013366-2	28	9	-36	TCGCCATATC	-13	GATAGTATTG	-140	1,1	7.65	10.90	s	8296	Rmet_0919	0	Op0264r_1	NA
4277	TSS_1613403-2	28	6	-42	TCAAAACCAA	-13	GGCAAAGTCG	-145	4,1	5.77	-3.60	no	2392	Rmet_1490	0	Op0420r_1	NA
4278	TSS_1928959-2	28	11	-33	TCGACGCAAG	-12	GCCATTGTAC	-145	1,1	3.84	8.10	s	42	Rmet_1776	ampC	Op0514r_1	Met-SecMetab
4279	TSS_2181661-2	28	6	-35	TCGACACCGC	-14	CGTATCGTAG	-143	1,1	5.34	12.10	s	9918	Rmet_2001	tnmB	Op2023r_1	NA
4280	TSS_2611672-2	28	10	-44	TCGCAAGCCT	-13	GGCATCATGC	-140	1,1	7.21	-6.10	no	117	Rmet_2401	0	Op0674r_1	NA
4281	TSS_3924522-2	28	7	-35	TAGTCTTATA	-14	ACTAGAATCC	-146	1,1	4.63	11.10	s	23	Rmet_3612	tam	Op1004r_1	NA
4282	TSS_153222+3	28	8	-28	AAACGTCGTG	-8	GTCCCTATCT	-147	3,0	4.25	-6.40	no	406	Rmet_R0074	0	Op1006f_1	NA
4283	TSS_841672+3	28	6	-45	GGCGGCGACT	-14	GGTACTATCA	-143	4,1	6.92	-12.60	no	309	Rmet_4254	0	Op1204f_2	NA
4284	TSS_903836+3	28	4	-43	TTGCATTGGG	-14	CGCATAATCA	-139	1,1	8.67	-1.60	no	44	Rmet_4309	0	Op1228f_1	NA
4285	TSS_1380061+3	28	6	-39	TCGCGGGCCC	-14	GGCACGATCC	-141	1,1	5.79	2.90	w	524	Rmet_4736	kdgD	Op1370f_1	MET-CAH-Ascorb
4286	TSS_1652794+3	28	9	-46	TTGACGACCA	-25	TGCACACTGT	-144	1,1	7.73	1.80	w	17	Rmet_4975	tniR	Op1460f_1	NA
4287	TSS_1895290+3	28	13	-40	TGGCCGAGCA	-14	CGTATTCTGA	-141	1,1	6.49	2.90	(w)-sba	204	Rmet_5200	0	Op1534f_1	MET-XEN-Toluene
4288	TSS_774975-3	28	9	-47	GCACAAGCCT	-27	GTCATATTTC	-140	4,1	8.33	-6.20	no	24	Rmet_4191	0	Op1185r_1	NA
4289	TSS_777842-3	28	8	-24	TGGTGCATCG	-2	CTGATGATTC	-146	1,1	4.52	-1.30	no	194	Rmet_6652	0	Op1185r_1	NA
4290	TSS_1167088-3	28	10	-40	TGGTTGTAGC	-18	AGGATAGTAC	-145	1,1	3.62	5.90	(m)-sba	992	Rmet_6675	0	Op2074r_1	NA
4291	TSS_2352397-3	28	7	-41	TTCAGGCTGC	-14	GTGAAAATCA	-142	1,1	6.76	0.90	(w)-sba	10	Rmet_5598	0	Op1667r_2	NA
4292	TSS_2377894-3	28	8	-35	TTGTGTTGTC	-14	ATTATGTTGC	-141	1,1	6.12	10.60	s	496	Rmet_5619	0	Op1677r_1	NA
4293	TSS_2402741-3	28	6	-40	TCGCGCGCCG	-14	GATACCATGA	-138	1,1	6.52	1.90	(w)-sba	2339	Rmet_5640	0	Op1681r_1	NA
4294	TSS_218386-4	28	8	-48	TTCATTAGGG	-25	AGTACGGTTA	-145	1,1	4.40	-3.10	no	5897	Rmet_6089	0	Op1854r_1	NA
4295	TSS_11078+5	28	9	-35	TGGAAGTTAC	-14	ACAACAATGA	-144	1,1	6.06	10.10	s	5452	Rmet_6327	0	Op1922f_1	NA
4296	TSS_30865-5	28	1	-41	TGGCGGTCGG	-13	TGTAACCTAC	-140	1,1	5.78	-2.10	no	240	Rmet_6342	stbD	Op1927r_1	NA
4297	TSS_809527+2	28	6	-37	TCGCCGCATG	-15	GGTATCTTTG	-140	1,1	6.37	9.90	s	1475	Rmet_0733	0	Op0207f_1	NA
4298	TSS_1918538+2	28	8	-28	TTCAACGAGT	-7	AACAAGGTCC	-146	1,1	3.20	1.10	(w)-nu	1314	Rmet_1768	argC1	Op0509f_1	MET-AA-Urea
4299	TSS_2384407+2	28	10	-47	TCAAGCGCGA	-22	GCCAAACTCG	-143	4,1	5.89	-5.60	no	93	Rmet_2178	ppk	Op0627f_1	MET-EN-OxPhos
4300	TSS_2700652+2	28	3	-37	TGCCACCGAC	-13	CGTATCATCG	-139	1,1	6.56	6.40	s	1088	Rmet_2489	mdh	Op0689f_1	MET-CAH-TCA
4301	TSS_3014190+2	28	9	-35	TTGACCACGT	-14	GTCAGGCTCT	-140	1,1	6.29	10.60	s	5978	Rmet_6558	0	Op0779f_1	NA
4302	TSS_3355665+2	28	7	-42	CCGGCTATAC	-14	ATTATTATTG	-142	5,1	8.41	-3.10	no	5247	Rmet_3101	nudH	Op0873f_1	NA
4303	TSS_3612364+2	28	7	-37	TCGTAAGCTT	-14	TCTACCATTG	-140	1,1	6.72	8.90	s	5652	Rmet_6590	0	Op0931f_1	NA
4304	TSS_909643-2	28	6	-49	TGGCCGACAG	-14	CTCATAATGG	-139	1,1	7.76	-14.10	no	3899	Rmet_0830	0	Op0236r_1	NA
4305	TSS_1249644-2	28	4	-42	GCTGCCCCAC	-13	AGCAGAATCA	-140	5,1	8.09	-6.60	no	301	Rmet_1141	0	Op0326r_1	NA
4306	TSS_1808688-2	28	5	-46	TGGCCGAGTC	-11	AGTAAGCTGA	-144	1,1	6.32	-13.10	no	4769	Rmet_1667	fimA	Op0468r_1	NA
4307	TSS_2160798-2	28	6	-48	TTGTGCGTGA	-14	TGTATAGTCT	-145	1,1	7.66	-11.60	no	39	Rmet_R0023	0	Op0580r_1	NA
4308	TSS_2215638-2	28	9	-47	GCGGCCCGCG	-10	GTCACAGTTT	-142	5,1	8.33	-19.10	no	383	Rmet_6504	0	Op0596r_2	NA
4309	TSS_3183796-2	28	2	-45	TCGGAATGAT	-25	TCGATGATGC	-142	5,1	7.41	0.30	w	4552	Rmet_2923	cobO	Op0826r_1	MET-COF-Porph
4310	TSS_3613945-2	28	6	-33	GCACGAATGG	-13	GCCACACTGT	-142	4,1	8.40	1.60	w	1026	Rmet_R0060	0	Op0930r_1	NA
4311	TSS_3615994-2	28	11	-37	TTGTGATTGT	-12	TTTAAAGTGA	-145	1,1	7.70	8.40	s	78	Rmet_R0061	0	Op0930r_1	NA
4312	TSS_3836881-2	28	8	-33	TTGATCAGAC	-13	GGCATTATTC	-138	1,1	8.18	8.60	s	1282	Rmet_3537	0	Op0978r_1	NA
4313	TSS_1982595+3	28	7	-36	TTGTTAGGCA	-15	TTCATACTCC	-142	1,1	6.66	11.80	s	20	Rmet_5283	nylB	Op1558f_1	NA
4314	TSS_2234065+3	28	7	-42	TCGGCTGTCC	-14	GATACCCTCA	-144	5,1	6.86	-2.10	no	1478	Rmet_6736	0	Op1632f_1	NA
4315	TSS_2579350+3	28	4	-37	TGGAAAGCAA	-9	CGTAAAGTGG	-145	1,1	7.20	4.90	(m)-ba	1567	Rmet_6605	0	Op1756f_1	NA
4316	TSS_174892-3	28	3	-33	TTGTGCGGAC	-12	GATAGACAGA	-147	1,1	2.59	6.60	s	40	Rmet_3639	0	Op1013r_1	NA
4317	TSS_273876-3	28	10	-42	TCGTGACCAG	-13	CGTATCCTGG	-140	1,1	5.49	-2.10	no	4695	Rmet_3726	0	Op1039r_1	NA
4318	TSS_945940-3	28	3	-36	TCGATCTGGT	-14	GGTAGGCTTT	-141	1,1	6.55	9.90	s	586	Rmet_6659	0	Op1237r_1	NA
4319	TSS_1041734-3	28	4	-40	TTGAATTATC	-13	CCTACACTCC	-139	1,1	9.30	3.40	(m)-sba	36	Rmet_4444	bug	Op1263r_1	NA
4320	TSS_1378922-3	28	1	-36	TTGTGACGCC	-13	TATATCCTGA	-140	1,1	6.71	11.40	s	134	Rmet_4734	garD	Op1369r_2	MET-CAH-Ascorb
4321	TSS_2272306-3	28	14	-36	TTCTAACAGG	-13	CGTAAAATTT	-139	1,1	8.33	10.90	s	885	Rmet_5524	0	Op1641r_1	MET-LIP-FASyn
4322	TSS_2293928-3	28	1	-38	TGAAACGGGC	-13	GATAGGCTCT	-151	4,1	0.23	3.40	m	185	Rmet_5546	vanA	Op1647r_2	MET-XEN-24D

4323	TSS_2299275-3	28	4	-40	GCGGAACGTG	-12	GATATTATTC	-139	5,1	10.02	-1.10	no	4451	Rmet_6743	0	Op1647r_1	NA
4324	TSS_2317419-3	28	4	-33	TCGATACCGG	-13	ACTACAATGC	-140	1,1	8.46	9.10	s	1853	Rmet_5562	0	Op1651r_1	NA
4325	TSS_191346+4	28	9	-47	CCGGACAGGT	-14	GGTAGACTAC	-141	5,1	10.22	-15.10	no	56	Rmet_6111	copR1	Op1865f_1	EIP-SIG-2Comp
4326	TSS_109268-4	28	7	-34	TTGCGTCTCC	-13	GTTAATCTCG	-141	1,1	7.16	8.60	s	28	Rmet_5951	tnpA	Op1810r_1	NA
4327	TSS_38861-5	28	10	-41	TGCCCTGCTT	-15	CGTAGAATTT	-139	1,1	7.00	1.40	(w)-sba	7147	Rmet_6344	merR	Op1929r_1	NA
4328	TSS_461730+2	28	7	-42	GCCAAACCGC	-14	CGTATCCTGA	-139	4,1	8.64	-4.60	no	1524	Rmet_0437	surA	Op0117f_1	MET-COF-B6
4329	TSS_936657+2	28	5	-48	TCGAGCAGAT	-15	AATAAGATCA	-143	1,1	6.36	-12.10	no	728	Rmet_0857	rpoZ	Op0245f_2	GIP-TK-RNAP
4330	TSS_1602777+2	28	5	-36	TGGACAGAAG	-15	AGCACAATTA	-140	1,1	7.38	12.30	s	179	Rmet_1482	0	Op0419f_1	NA
4331	TSS_3089149+2	28	7	-40	TTGCATGACG	-15	ATTAACATAT	-144	1,1	7.02	4.40	(m)-sba	1515	Rmet_6561	0	Op0801f_1	NA
4332	TSS_3366185+2	28	12	-36	TTCCAGATGA	-15	GGCAGCATGC	-139	1,1	6.52	8.30	s	679	Rmet_R0049	0	Op0875f_1	NA
4333	TSS_607246-2	28	4	-47	TGGCGGCCTC	-14	TGTATTGTAG	-141	1,1	4.76	-12.10	no	3499	Rmet_0563	ptb	Op0158r_1	MET-CAH-Pyr
4334	TSS_1019667-2	28	10	-36	TCGTGACCTT	-12	TCGATCATGT	-144	1,1	4.57	8.90	s	14597	Rmet_0919	0	Op0264r_1	NA
4335	TSS_1610756-2	28	7	-41	TTGCTCCCGC	-13	CGTACCCTAA	-142	1,1	5.83	-0.60	no	8015	Rmet_1481	ydcN	Op0418r_1	NA
4336	TSS_2968386-2	28	4	-42	CCTGCACTAT	-14	GGCAAGCTCG	-141	5,1	7.52	-6.60	no	542	Rmet_2731	0	Op0770r_1	NA
4337	TSS_3075446-2	28	13	-27	GCAACACCGC	-5	CCCAGTCTTC	-144	4,1	5.75	-1.80	no	4556	Rmet_2822	0	Op0794r_1	NA
4338	TSS_3076428-2	28	5	-44	GGCGCCGTAC	-13	GGTATGCTGA	-143	4,1	7.16	-10.60	no	5538	Rmet_2822	0	Op0794r_1	NA
4339	TSS_3513820-2	28	9	-36	TGCAACAAGA	-11	AGGACAATTG	-145	1,1	5.84	6.40	s	488	Rmet_3237	tatA	Op0918r_3	GIP-PTL-Exp
4340	TSS_76587+3	28	5	-36	TTGTTAGTGA	-14	CCTAGAATGG	-143	1,1	8.89	13.40	s	1404	Rmet_5881	0	Op1780f_1	NA
4341	TSS_148109+3	28	4	-34	TTGAGTGATT	-13	TTTATCATTG	-142	1,1	9.05	11.60	s	183	Rmet_6615	0	Op1006f_1	NA
4342	TSS_148166+3	28	14	-36	TTGCCAACCC	-15	CGTACTATTC	-142	1,1	9.06	12.80	s	126	Rmet_6615	0	Op1006f_1	NA
4343	TSS_1277539+3	28	12	-34	TCGAGACGCG	-14	GGCATCATCG	-138	1,1	6.59	9.10	s	3600	Rmet_4650	0	Op1344f_1	NA
4344	TSS_1332585+3	28	3	-35	TTGACCGGGT	-14	GTGATGATCG	-139	1,1	8.12	12.60	s	24144	Rmet_4714	0	Op1360f_1	NA
4345	TSS_1383767+3	28	9	-33	TACCGTCGCG	0	GTCAACATTT	-141	2,1	10.02	-5.80	no	23	Rmet_4738	0	Op1372f_1	NA
4346	TSS_1487725+3	28	4	-47	TGGATCAGAT	-14	GTTATCGTGG	-143	1,1	6.59	-11.10	no	43	Rmet_4833	0	Op1408f_2	NA
4347	TSS_1648927+3	28	7	-35	TGCACGAAAC	-15	TCAATACTCG	-144	1,1	4.66	8.60	s	10	Rmet_4972	tniA	Op1460f_1	GIP-REP-Recomb
4348	TSS_1811194+3	28	2	-43	GCGGCAGCGC	-14	ACTAAGATGG	-140	5,1	9.87	-5.10	no	714	Rmet_5118	0	Op1508f_1	NA
4349	TSS_685294-3	28	6	-35	TTGACGGCCG	-8	TGTAGAGTCA	-141	1,1	7.19	8.20	(s)-ba	3347	Rmet_4109	bug	Op1157r_1	NA
4350	TSS_2043482-3	28	11	-43	GCCGCCACGT	-9	CATAACCTTC	-145	4,1	6.29	-13.60	no	243	Rmet_5330	zneB	Op1577r_1	EIP-TRA-Ion
4351	TSS_2530208-3	28	2	-38	TCGATTGGGC	-13	CCTACACTCC	-142	1,1	7.00	5.90	m	60	Rmet_5769	0	Op1733r_2	NA
4352	TSS_141146-4	28	6	-35	TTGATTCAGC	-14	CCCATCTTCC	-144	1,1	4.59	10.60	s	1201	Rmet_6159	0	Op1882r_2	NA
4353	TSS_137231+5	28	7	-45	TGGCACTAAC	-21	GCCCAAGTCG	-150	1,1	1.02	-4.10	no	1318	Rmet_6283	0	Op1916f_1	NA
4354	TSS_90973-5	28	2	-47	TCGACAAAGA	-13	GGTAGAATTC	-143	1,1	10.27	-9.10	no	423	Rmet_6241	parB2	Op1901r_1	NA
4355	TSS_651943+2	27	4	-22	TCGTCTTGCC	0	GTGATAGTCA	-143	1,1	4.17	-2.30	no	34	Rmet_0603	0	Op0171f_1	NA
4356	TSS_762350+2	27	9	-38	TCGAATCGGA	-14	ACTATGTTTC	-142	1,1	5.46	6.90	s	35	Rmet_0690	0	Op0195f_1	EIP-TRA-ABC
4357	TSS_2163174+2	27	5	-40	TTTCGTCCCT	-15	GCCACGATGT	-144	2,1	9.44	1.90	(w)-sba	25	Rmet_1990	0	Op0583f_1	NA
4358	TSS_2848915+2	27	4	-43	TTCACTGCTT	-14	GGGATAATGT	-143	1,1	8.13	-1.10	no	1294	Rmet_2619	0	Op0733f_1	NA
4359	TSS_3508511+2	27	6	-44	TCGATCAGCG	-14	GTGACAATAT	-139	1,1	5.97	-5.10	no	124	Rmet_3233	0	Op0917f_1	NA
4360	TSS_3549383+2	27	2	-44	GCAGGACCCG	-14	GACACAATTG	-139	4,1	7.90	-9.60	no	1612	Rmet_6588	0	Op0923f_1	NA
4361	TSS_3624386+2	27	3	-45	TCGAAACATC	-15	ATTAATGTGT	-147	1,1	4.68	-6.10	no	4012	Rmet_3352	0	Op0933f_1	NA
4362	TSS_3751858+2	27	3	-33	TGCCGCCGTC	-11	AGGATACTCC	-143	1,1	4.44	6.20	m	364	Rmet_3473	0	Op0965f_2	NA
4363	TSS_101094-2	27	15	-34	TTGCGAACCT	-13	GTAAGCATTG	-142	1,1	5.97	9.60	s	469	Rmet_0092	0	Op0026r_1	NA
4364	TSS_769575-2	27	7	-43	TCGAACTGAT	-23	GCGATCATCG	-142	1,1	6.63	2.30	w	826	Rmet_0696	0	Op0196r_2	NA
4365	TSS_1595013-2	27	5	-46	TCGCATCGTC	-14	TGTAATCTGC	-141	1,1	5.91	-11.10	no	143	Rmet_1474	0	Op0412r_1	NA
4366	TSS_2298059-2	27	3	-35	TTGACGGCCT	-14	GACAAGGTGC	-143	1,1	5.46	10.60	s	144	Rmet_2115	rpoS	Op0612r_2	GIP-TK-RNAP
4367	TSS_3311774-2	27	10	-41	TTCACGGCGG	-14	GATATCCTCG	-138	1,1	7.03	1.90	(w)-sba	56	Rmet_3054	aroE	Op0860r_2	MET-AA-PheTyrTrp
4368	TSS_3624997-2	27	5	-47	GCCGGAACGC	-13	GGTATCTTGA	-143	4,1	8.83	-16.60	no	1336	Rmet_3346	uspA8	Op0932r_3	NA
4369	TSS_1061603+3	27	5	-39	TCGCCAATTC	-16	GCTAGTGTCT	-145	1,1	5.47	6.90	s	55	Rmet_4465	czcR2	Op1274f_1	EIP-SIG-2Comp
4370	TSS_1228729+3	27	7	-44	CCAGAAATGT	-16	CCTACGATGT	-137	5,1	8.73	-7.60	no	2193	Rmet_4606	metY	Op1326f_1	MET-AA-Met
4371	TSS_1240929+3	27	6	-44	TTCCGATTGC	-19	TGTAGGATGA	-142	1,1	7.61	-0.10	no	927	Rmet_4616	0	Op1330f_1	NA
4372	TSS_1977225+3	27	2	-40	GCCACGCCGC	-14	GCTAGAATCG	-136	4,1	9.86	0.40	(w)-sba	57	Rmet_5277	0	Op1554f_2	NA
4373	TSS_547386-3	27	6	-33	TTGACGCTGC	-11	GCAACAGTGA	-144	1,1	5.97	10.20	s	84	Rmet_3980	livH4	Op1115r_1	EIP-TRA-ABC
4374	TSS_887276-3	27	5	-43	TGCAGAGCCT	-13	ACTACCATGC	-139	1,1	6.21	-4.60	no	3974	Rmet_4291	prpB	Op1219r_1	NA
4375	TSS_1111879-3	27	8	-41	TGCATGAAAC	-13	GGCAATATAA	-146	1,1	3.85	-2.60	no	137	Rmet_4508	0	Op1287r_1	NA
4376	TSS_1738304-3	27	3	-35	TTGCGATAAT	-13	CTAATCATGT	-143	1,1	6.46	12.20	s	60	Rmet_5049	hutC	Op1485r_2	NA
4377	TSS_1741566-3	27	5	-40	TGGTGATGCG	-14	CGTACGCTCG	-140	1,1	5.63	1.90	(w)-sba	37	Rmet_5051	0	Op1485r_1	NA
4378	TSS_2259623-3	27	9	-31	AACGTTCTCA	-11	GTCTGTCACA	-149	3,0	3.34	-6.40	no	5608	Rmet_5508	0	Op1639r_1	NA
4379	TSS_2572433-3	27	6	-48	GCGGCCGGAT	-14	TCTATTATTC	-138	5,1	11.06	-15.10	no	197	Rmet_5808	prfC	Op1751r_2	NA
4380	TSS_29273+4	27	3	-38	TTGAGGACTT	-8	AAGAACATGT	-144	1,1	5.70	1.40	(w)-ba	722	Rmet_6033	0	Op1837f_1	NA
4381	TSS_138760+2	27	7	-44	TGGAAGGCAC	-15	GGGATGATCA	-140	1,1	6.19	-4.10	no	1178	Rmet_0134	0	Op0035f_1	NA
4382	TSS_396421+2	27	12	-42	TCGAAGGTCT	-14	TCTACGATGT	-141	1,1	7.60	-1.10	no	259	Rmet_0372	0	Op0103f_4	NA
4383	TSS_2276981+2	27	9	-41	TGGCCGCCCT	-15	TTTAGCATGA	-141	1,1	6.54	1.90	(w)-sba	15820	Rmet_2111	0	Op0609f_1	NA

4384	TSS_2992402+2	27	8	-34	TCGAAATACG	-14	GCAATAATGA	-142	1,1	7.31	10.10	s	6285	Rmet_6557		0	Op2043f_1	NA
4385	TSS_3223950+2	27	8	-36	TTCAAGGCGG	-12	GTTAAGCTTC	-140	1,1	6.92	7.90	s	197	Rmet_2966		0	Op0835f_1	NA
4386	TSS_3572701+2	27	3	-34	TTGCACTTTT	-14	GCGACATTTC	-139	1,1	7.35	7.60	s	12972	Rmet_3322		0	Op0927f_1	NA
4387	TSS_3795105+2	27	8	-37	TCGAAGCCCA	-14	GGTATAGTCG	-138	1,1	7.57	9.90	s	4015	Rmet_3509		0	Op0973f_1	NA
4388	TSS_1137-2	27	5	-32	TTCTTGCCGG	-12	AATATCATCG	-143	1,1	5.44	6.10	m	1447	Rmet_3615		0	Op1004r_1	GIP-TL-Ass
4389	TSS_101024-2	27	3	-44	TACCGTCGGC	-14	GTTACGATTG	-142	2,1	10.26	-7.60	*no*	399	Rmet_0092		0	Op0026r_1	NA
4390	TSS_204352-2	27	5	-28	TGGAGGCGGG	-8	CGAACCGTCG	-146	1,1	1.84	0.10	*no*	11894	Rmet_0185		0	Op0056r_1	NA
4391	TSS_1083452-2	27	4	-43	TCGACGTGCG	-14	AGTACAATAC	-137	1,1	8.54	-1.10	*no*	391	Rmet_0993		0	Op0282r_1	NA
4392	TSS_1229907-2	27	8	-36	CTCCGGTCGC	-14	TCGATAATGC	-145	1,1	2.94	7.90	s	2618	Rmet_1121	*acrD*		Op0320r_1	EIP-TRA-Ion
4393	TSS_1832018-2	27	3	-38	TCCACAGACA	-14	GTCATACTTG	-141	1,1	6.08	7.40	s	789	Rmet_6468		0	Op0478r_1	NA
4394	TSS_2382919-2	27	9	-49	TGGCCAGCGC	-13	AGTACGATTG	-140	1,1	7.94	-15.10	*no*	3253	Rmet_6511		0	Op0624r_1	NA
4395	TSS_2823195-2	27	5	-34	TTGCCGCTCT	-13	AGCACGATGC	-138	1,1	7.29	9.60	s	1307	Rmet_2594		0	Op0726r_1	NA
4396	TSS_3225326-2	27	2	-38	TCGCGCCGCA	-14	GGCAATATCG	-142	1,1	5.28	4.90	m	1156	Rmet_6569		0	Op2046r_1	NA
4397	TSS_3616468-2	27	7	-22	TTCATGACTG	0	AACAAGGTAG	-147	1,1	1.60	-4.30	*no*	106	Rmet_R0063		0	Op0930r_1	NA
4398	TSS_793558+3	27	6	-35	TAGCAGGCGC	-15	GATACAGTCC	-141	1,1	4.37	8.10	s	74	Rmet_4210	*tcuA*		Op1194f_1	NA
4399	TSS_1372625+3	27	5	-36	TTGACCGACG	-15	GCGAGCATGA	-140	1,1	6.36	11.80	s	588	Rmet_4729		0	Op1366f_3	NA
4400	TSS_1374115+3	27	1	-29	TTCAACGCGA	-5	TCGACAATCT	-142	1,1	6.54	2.70	*(w)-nu*	580	Rmet_4731		0	Op1368f_1	NA
4401	TSS_2409537+3	27	3	-46	GCGGCCCGAG	-15	GCCATCATGA	-138	5,1	9.37	-11.10	*no*	1064	Rmet_5650		0	Op1688f_1	NA
4402	TSS_2579114+3	27	10	-44	TCGACAATAT	-24	GAGATGATCC	-141	1,1	6.95	3.30	*m*	212	Rmet_5816	*cspA*		Op1756f_1	NA
4403	TSS_143877-3	27	5	-33	TTTCGGTTCC	-13	CCTATACTTG	-135	2,1	12.43	7.10	s	25	Rmet_5937		0	Op1805r_2	NA
4404	TSS_766269-3	27	4	-45	GGAACAACAG	-14	GGTATTGTTT	-141	4,1	7.87	-9.60	*no*	309	Rmet_4180		0	Op1179r_1	NA
4405	TSS_996728-3	27	8	-48	TTGCGCTGCT	-13	TCTATCATGC	-137	1,1	9.72	-12.60	*no*	61	Rmet_4399	*potG*		Op1251r_1	NA
4406	TSS_1916038-3	27	3	-38	TTGATGCGCT	-13	TATAGGCTTC	-143	1,1	6.65	6.40	s	1000	Rmet_5220		0	Op1539r_1	NA
4407	TSS_2065047-3	27	3	-38	CCGGAACCAG	-13	GTGATCATGG	-139	5,1	8.24	2.90	w	202	Rmet_5345	*xpsR*		Op1587r_1	NA
4408	TSS_8117-4	27	5	-35	AAGCGGTGCG	-10	ATGACGCTCT	-143	2,1	4.91	1.70	w	3294	Rmet_6060	*repA*		Op1846r_1	NA
4409	TSS_140953+5	27	5	-34	TTCCCAATCA	-13	AGCAGCATTT	-143	1,1	5.33	9.10	s	430	Rmet_6286		0	Op1916f_3	NA
4410	TSS_12119-5	27	3	-43	TGGACCGAAT	-13	GGTAGCCTGC	-141	1,1	7.41	-4.10	*no*	4752	Rmet_6320	*int*		Op1919r_1	NA
4411	TSS_169300-5	27	3	-25	TGGCACGTGC	0	CTTACGCTGT	-143	1,1	6.31	-4.30	*no*	11941	Rmet_6301	*trbF*		Op1917r_1	NA
4412	TSS_315646+2	27	9	-39	TGGCGGCGTC	-13	AGCAAAATGG	-142	1,1	7.00	2.90	w	7816	Rmet_0308		0	Op0087f_1	EIP-TRA-Ion
4413	TSS_1163324+2	27	7	-35	TTGCAGCGCA	-14	AGAACAATAC	-141	1,1	6.47	10.60	s	1828	Rmet_1073	*slyD*		Op0309f_1	GIP-PTL-Fold
4414	TSS_1269697+2	27	2	-38	TCGAGTCCAA	-16	ACGAACATGC	-144	1,1	3.97	7.90	s	295	Rmet_1160	*thrS*		Op0333f_2	MET-AA-GlySerThr
4415	TSS_1591376+2	27	3	-46	TGGAACAAGT	-14	CGTAAGCTGT	-139	1,1	7.01	-10.10	*no*	4275	Rmet_1475		0	Op0413f_1	NA
4416	TSS_1687089+2	27	9	-26	TTCCAGGCGT	-3	TCCAAAGTCC	-143	1,1	5.16	-1.30	*no*	3912	Rmet_1570		0	Op0433f_1	NA
4417	TSS_2652155+2	27	1	-44	TGCCGCAAGC	-16	GGCAAACTGT	-145	1,1	5.84	-6.60	*no*	43	Rmet_2441	*moaA*		Op0683f_1	MET-COF-Folate
4418	TSS_2841833+2	27	13	-47	TCGAGGTGTT	-14	CCGACAATGG	-141	1,1	6.82	-11.10	*no*	226	Rmet_2613		0	Op0731f_1	NA
4419	TSS_2867237+2	27	2	-46	TCCAGGCGCT	-20	GTCAAGGTGT	-144	1,1	2.33	-6.60	*no*	396	Rmet_2631	*galU*		Op0737f_2	MET-CAH-Pent
4420	TSS_2957179+2	27	3	-43	TGGAAAATCA	-16	GCTACAATCT	-136	1,1	10.24	0.90	*(w)-sba*	4093	Rmet_6554		0	Op2042f_1	NA
4421	TSS_3008800+2	27	11	-26	TTGCTCATGT	-3	GGCAACCTTG	-141	1,1	6.55	-0.80	*no*	1132	Rmet_2768	*ompA*		Op0777f_1	NA
4422	TSS_3155680+2	27	4	-44	TTGCTCCCCT	-14	GGTAAATTCT	-139	1,1	8.06	-4.60	*no*	1610	Rmet_2903		0	Op0819f_1	NA
4423	TSS_3552930+2	27	1	-40	TGGCCGCTTG	-14	TGTAGGCTAC	-142	1,1	5.61	1.90	*(w)-sba*	8229	Rmet_3285	*engB*		Op0925f_1	NA
4424	TSS_3589471+2	27	3	-41	TTTCTTCGCG	-15	TGCAGAATAC	-136	2,1	12.62	0.90	*(w)-sba*	4642	Rmet_3329		0	Op0929f_1	NA
4425	TSS_3708617+2	27	2	-47	CGCATGATCA	-15	GCGATAATTG	-146	1,1	1.73	-13.60	*no*	147	Rmet_3431	*yraL*		Op0951f_1	NA
4426	TSS_910692-2	27	5	-44	TTCATCTGTC	-13	CCGATACTTC	-141	1,1	5.94	-6.10	*no*	392	Rmet_0832	*aldH*		Op0238r_1	NA
4427	TSS_1012750-2	27	7	-27	GGAAGGCAGT	-7	AACAGGATAA	-145	4,1	4.46	-4.40	*no*	7680	Rmet_0919		0	Op0264r_1	NA
4428	TSS_1764762-2	27	7	-41	TCTGCAAGTC	-14	TGTAGATTTG	-140	5,1	8.79	0.40	*(w)-sba*	121	Rmet_1635	*bug*		Op0458r_1	NA
4429	TSS_3038596-2	27	12	-41	TGCATCGGTC	-14	GCTACACTAT	-140	1,1	6.30	-0.60	*no*	1282	Rmet_2789	*btuB*		Op0784r_1	EIP-TRA-Pores
4430	TSS_3064586-2	27	9	-47	TCGCTTCATT	-24	CCTAAAGTTA	-143	1,1	5.25	-2.10	*no*	9	Rmet_2816	*cysI*		Op0792r_2	MET-EN-Sulf
4431	TSS_3428641-2	27	1	-22	GCGGCTAGAC	-1	ATGAACCTTT	-146	5,1	4.91	-7.90	*no*	955	Rmet_R0050		0	Op0886r_1	NA
4432	TSS_3576076-2	27	2	-42	TGGCCGATAA	-7	ATCAAGGTAC	-146	1,1	2.89	-10.10	*no*	307	Rmet_3306	*rplX*		Op0926r_2	GIP-TL-Ribo
4433	TSS_171244+3	27	5	-37	TTGTGTCAGA	-17	AATATAGTAT	-145	1,1	6.05	9.80	s	58	Rmet_3635		0	Op1012f_2	NA
4434	TSS_387843+3	27	9	-41	TCGCAGATCA	-14	ATTAACATGC	-139	1,1	6.89	-0.10	*no*	305	Rmet_3844	*rpoT*		Op1068f_1	GIP-TK-RNAP
4435	TSS_1602357+3	27	3	-48	TTGACACCGG	-17	TTCACGATGA	-139	1,1	7.06	-7.60	*no*	115	Rmet_4937		0	Op1450f_1	NA
4436	TSS_1663990+3	27	5	-37	TGGACTGGCA	-15	GCCAGAATTG	-137	1,1	7.47	10.90	s	514	Rmet_4988		0	Op1464f_2	EIP-TRA-ABC
4437	TSS_1678663+3	27	5	-37	TTCCGGGCCG	-13	GCCATAATGC	-136	1,1	8.10	7.90	s	6015	Rmet_5004		0	Op1468f_1	NA
4438	TSS_1919188+3	27	10	-43	TGAAAGCCGG	-14	GATATCATAA	-145	4,1	6.70	-3.60	*no*	5846	Rmet_5229		0	Op1542f_1	NA
4439	TSS_2229595+3	27	9	-47	TTGCATCAAC	-14	GTTACCATCC	-139	1,1	9.14	-10.60	*no*	342	Rmet_6732		0	Op2096f_1	NA
4440	TSS_2373031+3	27	3	-37	TGGAAAAGAC	-15	GGCAGAATAA	-140	1,1	7.67	10.90	s	858	Rmet_5617		0	Op1676f_1	MET-LIP-Ster
4441	TSS_566375-3	27	3	-37	TTGCCAGTGT	-13	TGTAGACTTG	-140	1,1	9.84	10.40	s	129	Rmet_3995	*bglX*		Op1121r_1	MET-CAH-Starch
4442	TSS_583568-3	27	6	-36	TCGACTGTAT	-10	TGTAAATTGA	-143	1,1	6.86	7.90	s	29	Rmet_4012		0	Op1125r_1	NA
4443	TSS_608902-3	27	6	-45	TTGACGACCA	-24	TGCACACTGT	-144	1,1	7.73	2.80	*w*	18	Rmet_4042	*tniR*		Op1139r_1	NA
4444	TSS_959697-3	27	2	-34	TCACCGCCAC	-14	CGCATAATCC	-140	4,1	7.48	7.60	s	4364	Rmet_4359	*sucC1*		Op1243r_1	MET-CAH-TCA

4445	TSS_1001207-3	27	7	-38	CCTGATACGC	-13	GCCACAATCG	-139	5,1	7.99	0.40	w	504	Rmet_4403	*shc*		Op1253r_1	NA
4446	TSS_1277100-3	27	2	-34	TGGACGCCGA	-13	GGTAAATTAA	-143	1,1	6.88	10.10	s	911	Rmet_4645	*bug*		Op1341r_1	NA
4447	TSS_1920807-3	27	9	-39	GGAGGGGAAC	-19	ACAATGATTT	-144	4,1	6.03	0.80	*(w)-ba*	1199	Rmet_5224		*0*	Op1541r_3	NA
4448	TSS_2273129-3	27	2	-37	TGGTCAAGCC	-13	ATTACCATGT	-144	1,1	6.88	8.90	s	1708	Rmet_5524		*0*	Op1641r_1	MET-LIP-FASyn
4449	TSS_176655+4	27	2	-41	TTGCCGTATT	-14	TATAGAATTT	-143	1,1	10.11	3.40	*(m)-sba*	236	Rmet_6381		*0*	Op1873f_2	NA
4450	TSS_114524-4	27	6	-37	TTGACTCTAT	-14	TGTTAAATCG	-145	1,1	4.35	10.40	s	28	Rmet_5947	*pbrA*		Op1808r_1	MET-EN-P_ATPase
4451	TSS_75226+5	27	1	-42	TCGAAGTCAT	-15	CCGACAATTC	-144	1,1	6.90	-0.10	*no*	25	Rmet_6223		*0*	Op1900f_1	NA
4452	TSS_1027297+2	26	8	-37	TTCCGCCGAC	-14	GCCAAGCTGT	-141	1,1	4.98	5.90	m	282	Rmet_0941		*0*	Op0267f_3	NA
4453	TSS_1254853+2	26	7	-45	TGGCCTACTG	-15	TGCATCATCA	-143	1,1	5.62	-6.10	*no*	3154	Rmet_1147		*0*	Op0329f_1	NA
4454	TSS_1508993+2	26	9	-40	TTGCAGGGAA	-13	GTCAAAGTGG	-137	1,1	7.03	1.40	*(w)-sba*	346	Rmet_1400	*dadX*		Op0393f_1	MET-AA-AlaAsp
4455	TSS_1722142+2	26	2	-45	GGCGACATGC	-14	GCTATCCTCA	-138	4,1	7.86	-12.60	*no*	360	Rmet_1597		*0*	Op0443f_2	NA
4456	TSS_2056208+2	26	3	-44	TCGCGGGCGG	-15	GGTATGTTCA	-142	1,1	5.59	-5.10	*no*	403	Rmet_6484		*0*	Op0545f_1	NA
4457	TSS_2526988+2	26	7	-49	TGCAAGCTTT	-14	GATATGCTGA	-143	1,1	6.02	-16.60	*no*	436	Rmet_2306		*0*	Op0659f_1	NA
4458	TSS_2939026+2	26	4	-42	TCGTCAGCAG	-14	TGTAGAGTTC	-141	1,1	5.82	-0.10	*no*	574	Rmet_6552		*0*	Op2040f_1	NA
4459	TSS_3606371+2	26	4	-41	TTCACCTTGC	-21	GCGATCATTG	-142	1,1	7.07	5.30	*m*	11645	Rmet_6590		*0*	Op0931f_1	NA
4460	TSS_900674-2	26	7	-38	TCGAGGAGAA	-4	AGGACTTTGC	-147	1,1	2.72	-5.10	*no*	451	Rmet_0825		*0*	Op0234r_1	NA
4461	TSS_911512-2	26	4	-28	TTGCCTGCGC	-7	ATCACGATGT	-138	1,1	6.45	3.60	*(m)-nu*	228	Rmet_0833		*0*	Op0238r_1	NA
4462	TSS_1094989-2	26	4	-39	GGAGCAGAGA	-14	TGCATCCTTC	-149	4,1	5.38	0.40	w	3008	Rmet_0999	*nadK*		Op0284r_1	MET-COF-NicAm
4463	TSS_1246548-2	26	9	-37	TGGCGCTCGT	-14	CCTATCATCA	-137	1,1	7.41	8.90	s	4883	Rmet_1134	*metY*		Op0324r_1	MET-AA-Met
4464	TSS_1796874-2	26	4	-37	TTGTGCAGTG	-14	TGCACGATCT	-141	1,1	6.03	8.40	s	216	Rmet_6464		*0*	Op0468r_3	NA
4465	TSS_1918651-2	26	8	-36	TCGAGGAACT	-13	GATATCGTTG	-142	1,1	6.08	9.90	s	1645	Rmet_1765		*0*	Op0506r_1	NA
4466	TSS_2484400-2	26	2	-34	TTGCGAAAAT	-13	AATAGACTCT	-143	1,1	8.47	10.60	s	891	Rmet_6514		*0*	Op2031r_1	NA
4467	TSS_2700967-2	26	8	-38	TCGTGACTAC	-12	GTTAAATTCC	-141	1,1	6.55	4.90	m	846	Rmet_2486	*sdhC*		Op0688r_2	MET-CAH-TCA
4468	TSS_2719559-2	26	13	-39	TTCAGAATCC	-14	ACTATCATAT	-147	1,1	7.37	6.90	s	35	Rmet_2504		*0*	Op0694r_2	MET-CAH-PP_KDPG
4469	TSS_893170+3	26	4	-48	TTCATGATGT	-14	GCGAGAATTT	-143	1,1	7.05	-13.10	*no*	4619	Rmet_4303		*0*	Op1226f_1	EIP-TRA-Ion
4470	TSS_975299+3	26	8	-47	GCTGCATCCG	-14	GTTACTTTTC	-143	5,1	7.04	-15.60	*no*	28	Rmet_4376		*0*	Op1248f_2	NA
4471	TSS_1455549+3	26	4	-37	CCATCAGCAC	-15	GGTATCGTCA	-144	5,1	6.02	6.40	s	913	Rmet_4809	*acpA*		Op1400f_1	MET-COF-Ribo
4472	TSS_1658720+3	26	2	-37	TCGCCTCCGA	-14	GCCATACTGT	-141	1,1	6.76	8.90	s	460	Rmet_4984		*0*	Op1462f_1	NA
4473	TSS_2210089+3	26	7	-37	TGCATTGGTT	-14	ACTACAATCG	-144	1,1	6.96	8.40	s	369	Rmet_5464		*0*	Op1624f_1	NA
4474	TSS_2552483+3	26	4	-38	TGGTCTGATC	-14	GATAAGCTTC	-139	1,1	5.92	5.90	m	1394	Rmet_5796		*0*	Op1746f_1	NA
4475	TSS_367379-3	26	4	-37	TTCCGTGGCC	-13	GCTACACTCG	-137	1,1	8.05	6.90	s	637	Rmet_3824		*0*	Op1063r_1	NA
4476	TSS_903704-3	26	6	-48	TTGCAGAGTG	-17	TTGACTATTC	-143	1,1	6.41	-10.60	*no*	65	Rmet_4308		*0*	Op1227r_1	NA
4477	TSS_1602200-3	26	1	-43	GTCCGAACTT	-14	TCTACATTGA	-142	4,1	5.48	-6.10	*no*	9	Rmet_4936		*0*	Op1449r_1	NA
4478	TSS_1688799-3	26	3	-40	TGGAAGGATC	-12	CTTATAATTC	-136	1,1	9.68	2.90	*(w)-sba*	23	Rmet_5009		*0*	Op1471r_1	NA
4479	TSS_1739141-3	26	7	-34	TGGACGCCAG	-13	GCAAGTATTC	-142	1,1	4.43	9.10	s	897	Rmet_5049	*hutC*		Op1485r_2	NA
4480	TSS_1954898-3	26	6	-34	TCGAACTGTT	-11	GTCACACTGC	-139	1,1	7.31	7.70	s	4043	Rmet_5250		*0*	Op1549r_1	DIV-MOT-Chemotax
4481	TSS_2239436-3	26	4	-40	TTGCTCATAT	-13	GCCAAAATGT	-139	1,1	9.17	2.40	*(w)-sba*	3641	Rmet_5490	*pobA*		Op1631r_1	MET-XEN-24D
4482	TSS_29132+4	26	4	-43	TCGAGATAGG	-14	GCGATGCTGT	-144	1,1	5.61	-3.10	*no*	863	Rmet_6033		*0*	Op1837f_1	NA
4483	TSS_3056+5	26	12	-41	CCTGACACGT	-11	TGTAAATTTA	-144	5,1	8.27	-7.60	*no*	1094	Rmet_6391		*0*	Op1918f_4	NA
4484	TSS_36756+2	26	5	-35	TTGTCGCGCG	-14	GGCAGGATGA	-140	1,1	6.15	10.60	s	1229	Rmet_0036	*corA1*		Op0013f_1	EIP-TRA-Ion
4485	TSS_1272553+2	26	5	-34	TGGTGATGGT	-3	GTGATTCTTG	-144	1,1	4.87	-0.30	*no*	168	Rmet_1162	*rpmI*		Op0333f_3	GIP-TL-Ribo
4486	TSS_2261407+2	26	4	-49	TTGATATTCG	-15	GCCACGCTTG	-142	1,1	6.65	-13.60	*no*	28	Rmet_2083		*0*	Op0601f_4	MET-EN-Nitrog
4487	TSS_2602689+2	26	2	-42	CCGGAAACCT	-15	GGCATCATTC	-139	5,1	9.37	-3.10	*no*	70	Rmet_2392	*cycL*		Op0673f_1	MET-EN-Nitrog
4488	TSS_3504132+2	26	9	-40	TGGTACCCGA	-20	ATCATGGTTG	-146	1,1	2.62	3.30	*m*	3286	Rmet_3231	*mscL*		Op0915f_1	EIP-TRA-Pores
4489	TSS_2091255-2	26	4	-38	TTCCCGATCA	-14	GGTACAAGAA	-148	1,1	0.90	5.90	m	671	Rmet_1923		*0*	Op0560r_1	GIP-PTL-Fold
4490	TSS_2829071-2	26	5	-36	TTGCACAACT	-14	ATTAGTATCC	-141	1,1	7.92	11.40	s	104	Rmet_2600		*0*	Op0728r_1	NA
4491	TSS_319199+3	26	4	-39	GCGACCTGAT	-14	GTCATACTTC	-142	5,1	6.95	2.90	w	239	Rmet_3783		*0*	Op1050f_2	NA
4492	TSS_1415985+3	26	5	-47	TTGCCGCGCG	-14	TCTACGCTTT	-141	1,1	7.49	-10.60	*no*	484	Rmet_4772		*0*	Op1388f_1	NA
4493	TSS_1878282+3	26	9	-37	TTCCTGCAGC	-14	GCCATAATCG	-139	1,1	8.00	8.90	s	576	Rmet_5185		*0*	Op1528f_1	MET-EN-Nitrog
4494	TSS_2263621+3	26	8	-37	TTCTCGTCTC	-14	GATATCTTCA	-147	1,1	5.01	8.90	s	7957	Rmet_5525		*0*	Op1642f_1	NA
4495	TSS_2282972+3	26	5	-36	TTGCGTTGAC	-14	TATACAATGG	-139	1,1	10.24	12.40	s	26	Rmet_5536		*0*	Op1646f_1	NA
4496	TSS_2323624+3	26	4	-34	GAACTTGCTG	-14	CGAACGAATG	-141	3,0	6.88	-0.40	no	294	Rmet_5570		*0*	Op1656f_1	NA
4497	TSS_136190-3	26	4	-36	GCGTCCGCGC	-12	GATAAGGTGC	-143	5,1	6.08	4.90	m	98	Rmet_5931		*0*	Op1801r_1	NA
4498	TSS_388033-3	26	6	-33	TTGATCACGT	-13	CGTAAGCTTC	-138	1,1	7.57	7.60	s	503	Rmet_3842		*0*	Op1067r_1	NA
4499	TSS_832734-3	26	2	-39	TCGCTGTCAT	-13	CCGAAAATAC	-142	1,1	5.47	2.90	w	4095	Rmet_4240	*acnA3*		Op1201r_1	NA
4500	TSS_84829+4	26	5	-43	TTGTAATCGC	-14	CGTACAATTT	-140	1,1	10.06	-0.60	*no*	37	Rmet_5976	*czcE*		Op1819f_1	NA
4501	TSS_139394-4	26	10	-37	TTCAAGGGAC	-13	GTTATCGTCT	-142	1,1	6.30	7.90	s	33	Rmet_6160	*stbD*		Op1882r_2	NA
4502	TSS_343617+2	26	9	-47	GCAAACGAAG	-27	GCCAGCATGA	-142	4,1	7.11	-7.20	*no*	488	Rmet_0325		*0*	Op0093f_1	NA
4503	TSS_1130126+2	26	4	-47	GGACGGAAAA	-15	GTCATCATGG	-141	4,1	7.11	-15.60	*no*	95	Rmet_1040	*lolE*		Op0299f_1	NA
4504	TSS_1594822+2	26	4	-40	GCCAGAGCGC	-15	TGTAATGTGC	-143	4,1	7.76	-0.60	*no*	829	Rmet_1475		*0*	Op0413f_1	NA
4505	TSS_2380611+2	26	11	-48	TCCACACCCC	-5	AACACATTGC	-143	1,1	3.46	-22.60	*no*	126	Rmet_2174	*infA*		Op0625f_3	NA

4506	TSS_3643166+2	26	2	-39	TGGCCCATGG	-19	TTGATGATAA	-144	1,1	5.14	6.30	(m)-ba	2631	Rmet_3369	*paaA*	Op0937f_1	NA	
4507	TSS_149364-2	26	7	-42	GCAGGGGCGC	-13	AGTACGATCG	-141	4,1	9.17	-7.60	no	4835	Rmet_0139	*0*	Op0038r_1		0
4508	TSS_288872-2	26	12	-42	CCGGCAACCC	-13	GTCATAGTGA	-141	5,1	8.67	-4.10	no	5207	Rmet_0266	*0*	Op0076r_1	NA	
4509	TSS_1622788-2	26	7	-35	TGCTTGCCCT	-13	CGTACCATTT	-143	1,1	4.34	8.20	s	921	Rmet_1501	*cbbL*	Op0422r_1	MET-CAH-DiCarb	
4510	TSS_2225416-2	26	9	-42	TCGACGAACA	-14	TCCATGATGG	-142	1,1	6.50	-0.10	no	120	Rmet_2053	*ugpE*	Op0596r_1	EIP-TRA-ABC	
4511	TSS_2456794-2	26	6	-46	TTGTCGAACA	-22	AGCGCCATGC	-149	1,1	-0.31	-2.60	no	3505	Rmet_2237	*0*	Op0640r_1	NA	
4512	TSS_2911651-2	26	8	-38	TGGCCATCCG	-13	GGAAGAATTC	-142	1,1	6.89	6.90	s	50	Rmet_2677	*tolR*	Op0756r_2	NA	
4513	TSS_2920079-2	26	6	-41	TCGGCATGAT	-13	TGTAACCTTC	-139	5,1	8.41	-0.10	no	7369	Rmet_2679	*ybgC*	Op0756r_1	NA	
4514	TSS_3590595-2	26	9	-49	TTGCCGGGCG	-29	TTACACATCT	-148	1,1	0.00	-5.20	no	46	Rmet_3325	*fusA1*	Op0928r_2	NA	
4515	TSS_3819255-2	26	7	-49	TTCCTACTCA	-5	TCTACCGTTG	-147	1,1	4.83	-25.10	no	94	Rmet_3526	*ompW1*	Op0976r_3	NA	
4516	TSS_309180+3	26	1	-46	TCCTCTTATA	-18	TCTATTGTCC	-148	1,1	3.19	-6.60	no	333	Rmet_3774	*0*	Op1048f_2	NA	
4517	TSS_311087+3	26	4	-42	TGGACGGAGC	-14	TGCACTATTA	-142	1,1	5.76	-1.10	no	816	Rmet_3776	*0*	Op1050f_1	NA	
4518	TSS_1390116+3	26	9	-41	GGCCAGGCAC	-14	GTTACCATCT	-139	4,1	8.93	-4.60	no	22	Rmet_4746	*0*	Op1376f_2	MET-LIP-FAMet	
4519	TSS_1454450+3	26	5	-36	CCGGAACCCG	-2	TTGACTGTCG	-146	5,1	4.38	-6.10	no	84	Rmet_4807	*0*	Op1398f_2	NA	
4520	TSS_2239292+3	26	6	-49	GGCGCCGAGC	-14	GAGACAATGA	-144	4,1	7.05	-19.60	no	1970	Rmet_5496	*0*	Op1634f_1	GIP-REP-Recomb	
4521	TSS_2284085+3	26	4	-49	TATCGTCTGC	-14	TGTATCCTGG	-141	2,1	9.63	-17.60	no	9647	Rmet_6742	*0*	Op2098f_1	NA	
4522	TSS_2336089+3	26	11	-35	TGGTCAGACA	-14	GATACGATGG	-137	1,1	7.58	11.10	s	89	Rmet_5582	*0*	Op1662f_1	NA	
4523	TSS_2410199+3	26	2	-42	TTGCGTTGGC	-15	TGTAGTCTAG	-141	1,1	6.55	-0.60	no	402	Rmet_5650	*0*	Op1688f_1	NA	
4524	TSS_1175636-3	26	1	-34	TCGACAGCCG	-13	CGTAACGTTC	-141	1,1	5.03	10.10	s	2959	Rmet_4563	*0*	Op1313r_1	NA	
4525	TSS_1236908-3	26	13	-38	TCGTCCGGCT	-14	TGGATCATCG	-140	1,1	4.78	6.90	s	2022	Rmet_4608	*0*	Op1327r_1	NA	
4526	TSS_1557119-3	26	4	-33	TTGACATACA	-13	CATACACTGT	-139	1,1	9.40	10.60	s	49	Rmet_4893	*0*	Op1431r_1	NA	
4527	TSS_1800794-3	26	6	-35	CCGAACGTCT	-13	CGTACGATCC	-143	5,1	7.00	7.70	s	652	Rmet_5107	*0*	Op1505r_3	MET-CAH-Prop	
4528	TSS_2378876-3	26	4	-37	TTCCCACATT	-13	TCTACATTGG	-139	1,1	7.84	8.90	s	178	Rmet_5621	*0*	Op1677r_1	NA	
4529	TSS_2573408-3	26	2	-46	GCAGGCGTGT	-13	ACTATGATGA	-140	4,1	8.29	-14.60	no	110	Rmet_5809	*0*	Op1751r_1	NA	
4530	TSS_29112+4	26	4	-33	GCGGACGGTG	-8	TTCATGGTTC	-146	5,1	5.75	0.70	(w)-ba	883	Rmet_6033	*0*	Op1837f_1	NA	
4531	TSS_72644+4	26	10	-36	TTGACCTTCC	-12	TCCATATTAA	-139	1,1	6.62	11.40	s	1395	Rmet_5984	*czcN*	Op1821f_1	NA	
4532	TSS_191327-4	26	8	-40	TTGTTAATAC	-14	CGTAGTCTAC	-145	1,1	5.66	3.40	(m)-sba	128	Rmet_6112	*copA1*	Op1866r_1	NA	
4533	TSS_78601+5	26	1	-48	GCCGGCGTAC	-15	CATAAAATTG	-140	4,1	9.03	-16.60	no	12557	Rmet_6242	*0*	Op1902f_1	NA	
4534	TSS_120250+5	26	6	-36	TTGAAGGGCG	-15	AGCAGTATCA	-142	1,1	5.47	10.80	s	175	Rmet_6396	*0*	Op1910f_1	NA	
4535	TSS_145250+5	26	6	-48	GCCACAATAA	-15	TGCAGAATGA	-142	4,1	6.98	-14.60	no	12507	Rmet_6302	*trbB*	Op1918f_1	NA	
4536	TSS_8535-5	26	5	-45	TCTGCTCCCT	-14	TTGATAATCT	-148	5,1	7.47	-7.60	no	1168	Rmet_6320	*int*	Op1919r_1	NA	
4537	TSS_437154+2	25	5	-47	TTGTGCGATC	-25	CGTATAATGG	-137	1,1	9.98	2.40	w	132	Rmet_0410	*rplM*	Op0113f_2	GIP-TL-Ribo	
4538	TSS_465594+2	25	7	-35	TGCCGACTGC	-15	TGTACCATGA	-137	1,1	6.37	7.60	s	238	Rmet_0439	*ksgA*	Op0117f_1	GIP-TL-Ass	
4539	TSS_949208+2	25	7	-43	TTGTGCCTGT	-15	GGTAGCCTTG	-141	1,1	7.14	-2.60	no	429	Rmet_0869	*0*	Op0251f_1	NA	
4540	TSS_1710031+2	25	6	-34	TCGGACATTT	-14	GTTAAGCTTG	-141	5,1	7.89	6.10	m	1332	Rmet_1588	*prpR*	Op0439f_1	NA	
4541	TSS_1798519+2	25	7	-39	TCCCAACCGT	-13	TGTAACGTAC	-144	1,1	3.67	1.40	w	6384	Rmet_1668	*tnp*	Op0469f_1	NA	
4542	TSS_2395422+2	25	5	-46	GCAAGGCTAG	-14	ATTATGATCT	-144	4,1	7.55	-12.60	no	3622	Rmet_2190	*0*	Op0629f_1	NA	
4543	TSS_3128158+2	25	5	-38	TTCTCGTGGG	-14	CGGATGATTG	-143	1,1	5.28	6.90	s	2159	Rmet_2879	*tnpA*	Op0809f_1	NA	
4544	TSS_470447-2	25	4	-27	TTCCTGAAAT	-6	ATTACGATTT	-141	1,1	6.90	1.10	(w)-nu	136	Rmet_0443	*0*	Op0118r_3	NA	
4545	TSS_561771-2	25	8	-34	TGGTCCCGAT	-13	CGCACAATCG	-138	1,1	6.65	9.10	s	1206	Rmet_0524	*0*	Op0142r_1	NA	
4546	TSS_1531536-2	25	7	-47	TCGAGCGGGC	-13	TGGACAATGC	-141	1,1	7.08	-12.10	no	14745	Rmet_1404	*yheS*	Op0396r_1	GIP-TL-Ass	
4547	TSS_1776152-2	25	7	-34	TTGCTGCGGC	-13	GCCACCATCG	-139	1,1	7.11	8.60	s	85	Rmet_1643	*0*	Op0462r_2	NA	
4548	TSS_1796782-2	25	10	-43	TCGAGAACCT	-14	GGGAAAATGC	-141	1,1	7.91	-2.10	no	124	Rmet_6464	*0*	Op0468r_3	NA	
4549	TSS_2673374-2	25	4	-40	CCTGCCGCCG	-13	ATTATGATTG	-138	5,1	9.72	-1.60	no	417	Rmet_2461	*cvpA*	Op0688r_5	NA	
4550	TSS_2707880-2	25	4	-40	GTAGAGAACG	-13	GGTATTCTAC	-144	4,1	7.24	-2.10	no	6531	Rmet_2488	*0*	Op0688r_1	NA	
4551	TSS_3633371-2	25	12	-36	GCGGCTGCTG	-14	CGCATACTTC	-142	5,1	7.11	7.90	s	2837	Rmet_3353	*tnpA*	Op0934r_1	NA	
4552	TSS_292050+3	25	3	-40	TGGACACCAT	-15	AATAGCATCA	-145	1,1	6.35	5.90	(m)-sba	9	Rmet_6622	*0*	Op1044f_1	NA	
4553	TSS_473045+3	25	9	-30	TTCAAAAGTT	-4	TAAAACATGT	-145	1,1	4.85	1.70	(w)-nu	99	Rmet_R0075	*0*	Op1098f_1	NA	
4554	TSS_1302881+3	25	6	-26	GCGGCAGGTC	-3	GGGATGATGG	-138	5,1	9.33	-1.30	no	3073	Rmet_4673	*0*	Op1352f_1	NA	
4555	TSS_2010081+3	25	9	-36	TTGCATTTAA	-15	CATAGAATCG	-142	1,1	9.29	11.80	s	24	Rmet_5307	*0*	Op1568f_1	NA	
4556	TSS_2213409+3	25	4	-47	TGAGCCAAAC	-14	GCTACTCTCG	-145	4,1	6.77	-13.60	no	1806	Rmet_6729	*0*	Op2094f_1	NA	
4557	TSS_2533595+3	25	4	-45	TGGCTCATAT	-14	GTTATTGTGA	-142	1,1	5.85	-8.10	no	502	Rmet_5774	*0*	Op1736f_1	NA	
4558	TSS_255660-3	25	6	-39	TTCCGAAAGA	-14	GCGACAATAG	-140	1,1	7.38	4.90	m	812	Rmet_3712	*nagH*	Op1029r_1	MET-AA-Phe	
4559	TSS_908265-3	25	8	-45	TCGTGAAACC	-13	GGCATCATGC	-140	1,1	6.45	-8.10	no	1677	Rmet_4312	*0*	Op1229r_2	NA	
4560	TSS_1056865-3	25	5	-48	CCTGCCATGT	-12	GCGACAATGA	-138	5,1	9.33	-19.60	no	160	Rmet_4459	*0*	Op1269r_1	MET-AA-ValLeuIle	
4561	TSS_1311279-3	25	7	-41	GCATCAAGAC	-21	ATCATCCTGC	-144	5,1	5.29	-0.20	no	420	Rmet_4679	*0*	Op1353r_2	NA	
4562	TSS_2259277-3	25	6	-33	TCGAAGCGCT	-12	TGTACATTGC	-141	1,1	7.42	8.10	s	5262	Rmet_5508	*0*	Op1639r_1	NA	
4563	TSS_2318770-3	25	2	-37	TTCAGGCAAG	-13	GGCAACATGC	-141	1,1	6.36	6.90	s	3204	Rmet_5562	*0*	Op1651r_1	NA	
4564	TSS_180770+4	25	6	-35	TTGATGCCCA	-14	GCGATGTTCT	-141	1,1	4.50	10.60	s	1441	Rmet_6382	*copO*	Op1867f_1	NA	
4565	TSS_204320+4	25	2	-34	TCCACTGCAG	-14	CCTATTCTGC	-143	1,1	4.73	7.60	s	404	Rmet_6095	*0*	Op1857f_1	NA	
4566	TSS_100887-4	25	4	-34	GCGGAAGTGT	-13	TATAGACTGT	-143	5,1	9.21	6.10	m	21	Rmet_6371	*0*	Op2112r_1	NA	

4567	TSS_210619+2	25	7	-41	TCGGCCGCAT	-14	GATACGATGT	-137	5,1	10.23	0.90	(w)-sba	1332	Rmet_0203	0	Op0059f_3	NA		
4568	TSS_772447+2	25	7	-42	TGGCATTGGG	-15	TCTACGCTCT	-140	1,1	6.38	-2.10	no	28	Rmet_0700	dadA2	Op0197f_1	MET-EN-Nitrog		
4569	TSS_806594+2	25	10	-40	TCTGCAACGC	-14	CACACAATGG	-141	5,1	8.93	2.40	(w)-sba	118	Rmet_0729	0	Op0205f_2	GIP-PTL-T2S		
4570	TSS_1465921+2	25	11	-36	TGGACTTCGG	-14	GCCAACGTAC	-141	1,1	3.71	9.90	s	422	Rmet_1357	phaA	Op0383f_2	MET-CAH-Pyr		
4571	TSS_1619034+2	25	8	-37	GGCGACAAGT	-14	GGCATAATCA	-142	4,1	9.17	4.40	m	2957	Rmet_1502	cbbR1	Op0423f_1	NA		
4572	TSS_2069480+2	25	2	-40	GTCCAAGACC	-14	GATACTCTTG	-145	4,1	7.44	-1.10	no	4	Rmet_1900	0	Op0553f_2	NA		
4573	TSS_2091588+2	25	12	-32	GAATCTCTCG	-12	TCTCCAAACG	-147	3,0	5.87	-1.40	no	6471	Rmet_1930	0	Op0563f_1	NA		
4574	TSS_2194173+2	25	4	-47	TGGCCGTGGA	-14	GGCATCATGA	-141	1,1	7.25	-11.10	no	12082	Rmet_2037	fnrL	Op0595f_1	NA		
4575	TSS_2380584+2	25	3	-35	TGGAACATTT	-15	CCCCACATCG	-144	1,1	0.74	6.10	m	153	Rmet_2174	infA	Op0625f_3	NA		
4576	TSS_2769878+2	25	8	-39	TCGCCCGAAC	-14	ATTACTATTC	-145	1,1	6.84	4.90	m	102	Rmet_2545	0	Op0705f_1	NA		
4577	TSS_3369475+2	25	8	-34	TTGACGCTGG	-14	GGTATTCTCG	-137	1,1	8.86	10.60	s	792	Rmet_3110	pilD	Op0875f_2	GIP-PTL-T2S		
4578	TSS_3836888+2	25	3	-32	TCGCCACTTT	-3	TGAATAATGC	-142	1,1	7.33	2.70	(w)-ba	61	Rmet_3540	0	Op0981f_1	NA		
4579	TSS_198302-2	25	7	-35	TTGAACCGTT	-14	GCCACCATTC	-140	1,1	8.05	10.60	s	5844	Rmet_0185	0	Op0056r_1	NA		
4580	TSS_1571168-2	25	5	-42	TCGACCGGGA	-13	ATTACCATCG	-140	1,1	6.85	-1.10	no	15256	Rmet_1434	map	Op0402r_1	GIP-TL-Ass		
4581	TSS_1758353-2	25	6	-35	TTGAATCCCC	-14	CCCACACTGG	-139	1,1	6.98	10.60	s	26	Rmet_1629	0	Op0456r_1	NA		
4582	TSS_2365974-2	25	4	-47	TTCTGATTAG	-13	TTTAAAGTTG	-143	1,1	6.26	-12.10	no	2901	Rmet_2162	0	Op0622r_1	NA		
4583	TSS_2793805-2	25	11	-37	TGCAGGAACC	-16	TGGATGATCG	-144	1,1	4.67	7.80	s	1530	Rmet_2566	0	Op0710r_1	NA		
4584	TSS_3508758-2	25	1	-41	TTCCACACTG	-13	GCCACAATGG	-137	1,1	8.05	-1.10	no	143	Rmet_3232	0	Op0916r_1	NA		
4585	TSS_3644184-2	25	5	-33	TGCCAACGAC	-12	GCGACACTTG	-141	1,1	5.58	5.60	m	1277	Rmet_3367	0	Op0936r_1	NA		
4586	TSS_1969+3	25	12	-45	TTCCGTAAGG	-14	TCTATCCTGG	-141	1,1	6.77	-8.10	no	798	Rmet_5819	parA	Op1758f_1	DIV-Division		
4587	TSS_477280+3	25	8	-39	TTGCATTGGA	-14	TGTAGGATAG	-142	1,1	8.16	5.40	m	898	Rmet_R0079	0	Op1098f_1	NA		
4588	TSS_566373+3	25	8	-41	TCTGACAGGC	-15	GTTATTTTTG	-141	5,1	7.96	-0.60	no	80	Rmet_3996	mdoG	Op1122f_1		0	
4589	TSS_1024708+3	25	4	-35	TGGCCAATGG	-14	CGCACAATGT	-136	1,1	8.07	11.10	s	691	Rmet_4430	betB	Op1260f_2	NA		
4590	TSS_1063925+3	25	4	-48	TTCTACGGCA	-15	TTTATGATGC	-141	1,1	7.21	-12.10	no	7691	Rmet_4473	tnpR	Op1276f_1	NA		
4591	TSS_1482987+3	25	5	-35	TGAGAGAAAC	-13	TTAATAATGC	-143	4,1	7.82	9.20	s	94	Rmet_4829	qseB	Op1408f_1	EIP-SIG-2Comp		
4592	TSS_1535004+3	25	5	-29	GCGGCACGGG	-3	GGCAACTTCG	-142	5,1	7.05	-3.30	no	362	Rmet_4873	0	Op1424f_1	NA		
4593	TSS_1658659+3	25	5	-43	TGCTCCGGTG	-21	GAAAAAATGT	-145	1,1	4.13	2.40	w	521	Rmet_4984	0	Op1462f_1	NA		
4594	TSS_2157377+3	25	9	-45	TTCAACGGCG	-14	ACTATCATGT	-139	1,1	7.54	-6.10	no	1122	Rmet_5415	0	Op1612f_1	NA		
4595	TSS_852103-3	25	3	-36	CCGGCAGGAC	-13	GCCAAAATCC	-137	5,1	11.15	7.90	s	993	Rmet_4260	0	Op1207r_1	NA		
4596	TSS_1610854-3	25	5	-35	TTGAAAGACG	-13	TCTACGATCA	-138	1,1	8.71	13.20	s	94	Rmet_4945	0	Op1453r_2	NA		
4597	TSS_44462+4	25	2	-49	GCAGACCATC	-15	GCTACGATGT	-141	4,1	7.17	-19.60	no	771	Rmet_6013	0	Op1835f_2	NA		
4598	TSS_230601+4	25	4	-35	TTGCGGAGGC	-15	GAGAGAATGC	-138	1,1	8.45	9.60	s	382	Rmet_6067	tnpB	Op1849f_1	NA		
4599	TSS_105489-4	25	1	-47	CTGCGCGTGG	-14	GGCAGACTCC	-143	1,1	2.88	-14.60	no	4623	Rmet_6371	0	Op2112r_1	NA		
4600	TSS_19125+5	25	3	-41	TCGCTCTGCC	-14	GCGAAACTGT	-144	1,1	5.63	-1.10	no	273	Rmet_6330	0	Op1924f_1	NA		
4601	TSS_258781+2	25	10	-49	TTGAAATGCG	-15	CTTATGCTGG	-140	1,1	8.62	-11.60	no	87	Rmet_0242	gshA	Op0071f_3	MET-AA-GluGSH		
4602	TSS_409850+2	25	6	-34	TGGAACTGTC	-2	CTGACCATCG	-140	1,1	5.15	-1.30	no	600	Rmet_0385	0	Op0105f_2	NA		
4603	TSS_423709+2	25	3	-26	TGGCGTCCTC	-6	CTGAGGATCT	-141	1,1	3.44	-1.90	no	663	Rmet_6413	0	Op0109f_1	NA		
4604	TSS_551652+2	25	4	-38	TCGCCGTCAG	-15	AGCATAATGT	-138	1,1	7.49	8.90	s	131	Rmet_0515	ompR	Op0139f_1	EIP-SIG-2Comp		
4605	TSS_996508+2	25	5	-46	GGCCGAATGT	-14	TCTAAAATGT	-139	4,1	10.09	-12.60	no	298	Rmet_0911	ilvI	Op0263f_2	MET-CAH-But		
4606	TSS_1366031+2	25	6	-36	CCGGCCACAT	-14	CACATAATTC	-141	5,1	9.98	8.90	s	44	Rmet_1242	radC'	Op0357f_3	NA		
4607	TSS_2364288+2	25	12	-40	TTCATCTTTA	-14	GGCATAGTCG	-141	1,1	6.08	2.90	(w)-sba	1655	Rmet_2164	0	Op0623f_1	NA		
4608	TSS_2394982+2	25	2	-38	TTCCAGCATA	-14	CGTATCCTTG	-140	1,1	6.55	5.90	m	4062	Rmet_2190	0	Op0629f_1	NA		
4609	TSS_2611622+2	25	3	-45	TGCCAAAATT	-14	GTTAAACTTC	-145	1,1	8.12	-8.60	no	52	Rmet_2402	traR	Op0675f_1	NA		
4610	TSS_3360344+2	25	7	-37	TCGCGGAACT	-13	TAGAAGTTGA	-145	1,1	2.29	4.90	m	568	Rmet_3101	nudH	Op0873f_1	NA		
4611	TSS_3569768+2	25	10	-35	TTGCCCTGGC	-14	GTGATGATGG	-137	1,1	8.40	11.60	s	15905	Rmet_3322	0	Op0927f_1	NA		
4612	TSS_432062-2	25	3	-30	GTCAGGATGT	-6	TCGACCTTCA	-146	4,1	3.01	-1.30	no	775	Rmet_0403	yafJ	Op0110r_1	NA		
4613	TSS_2055363-2	25	1	-43	TTCAGGGCGG	-14	GCTACCATCC	-138	1,1	7.79	-3.10	no	26	Rmet_1887	trmC	Op0544r_1	NA		
4614	TSS_2161732-2	25	10	-43	TTGCCCGGAT	-13	ACTACATTGA	-142	1,1	8.14	-2.60	no	597	Rmet_1988	0	Op0582r_1	NA		
4615	TSS_2282603-2	25	5	-43	TTGAACCGGG	-23	CCCATGATCC	-144	1,1	7.48	3.80	m	16	Rmet_2101	hfq	Op0608r_2	NA		
4616	TSS_2304691-2	25	4	-40	TGGCAAGCTG	-14	TGTAGCATGG	-138	1,1	7.35	2.90	(w)-sba	125	Rmet_2122	0	Op0614r_2	EIP-TRA-ABC		
4617	TSS_2445663-2	25	3	-35	TCGATGCAGT	-13	TCTACGATGG	-141	1,1	7.34	10.70	s	1542	Rmet_2228	0	Op0638r_2	NA		
4618	TSS_2668442-2	25	11	-38	TCGCGCAGCG	-13	CGTAGACTTC	-139	1,1	7.02	4.90	m	858	Rmet_2456	0	Op0686r_1	NA		
4619	TSS_2804130-2	25	5	-49	TTCTAATGCT	-14	CCTAAACTGC	-143	1,1	7.81	-15.10	no	71	Rmet_2579	0	Op0716r_1	NA		
4620	TSS_2978646-2	25	5	-44	TGGCCGATCC	-14	TCTATCTTTG	-143	1,1	6.32	-5.10	no	447	Rmet_2742	0	Op0772r_2	GIP-REP-Recomb		
4621	TSS_3077485-2	25	3	-43	TGGCTTAAGG	-14	GATATCATCG	-141	1,1	7.73	-3.10	no	6595	Rmet_2822	0	Op0794r_1	NA		
4622	TSS_3576653-2	25	6	-38	TGCGAAGACT	-13	CGTACCCTCG	-143	4,1	5.51	3.40	m	506	Rmet_3307	rplN	Op0926r_2	GIP-TL-Ribo		
4623	TSS_3628033-2	25	10	-44	TTGATGAGCG	-13	ACCATAATTG	-143	1,1	8.48	-3.60	no	855	Rmet_3350	0	Op0932r_1	NA		
4624	TSS_3846780-2	25	7	-37	TTGCCTCCGG	-13	ATTACATTGC	-140	1,1	7.76	9.40	s	5959	Rmet_3542	0	Op0982r_1	NA		
4625	TSS_1227+3	25	3	-29	TGGACGCGCA	-2	TTGATGATCC	-141	1,1	5.72	0.70	(w)-nu	1540	Rmet_5819	parA	Op1758f_1	DIV-Division		
4626	TSS_153587+3	25	2	-30	GCAACACAAC	-5	CCCAGATTCG	-145	4,1	6.53	-0.80	no	41	Rmet_R0074	0	Op1006f_1	NA		
4627	TSS_1160936+3	25	4	-29	TTGACGATTT	-2	ATGAACATAC	-146	1,1	6.04	1.20	(w)-nu	121	Rmet_6672	0	Op1306f_1	NA		

4628	TSS_1296008+3	25	9	-39	TTGCCGAGTT	-15	GTCACCATAC	-141	1,1	7.58	6.40	s	1752	Rmet_4665	*0*	Op1350f_1	NA
4629	TSS_1475254+3	25	2	-45	TCGCTATTTT	-14	TGTAATATGG	-145	1,1	7.47	-7.10	*no*	460	Rmet_4823	*0*	Op1406f_1	NA
4630	TSS_1510107+3	25	1	-40	GCAACGGCCA	-14	GGCATGATGA	-140	4,1	6.29	-0.60	*no*	1058	Rmet_4853	*bugT*	Op1416f_1	NA
4631	TSS_1791601+3	25	5	-35	TTCAGTTCGC	-14	GCCAGAATCG	-139	1,1	7.33	10.10	s	2226	Rmet_5102	*0*	Op1502f_1	MET-CAH-FbP
4632	TSS_2179350+3	25	7	-31	GCGGACGGCG	-3	GACATGGTGT	-139	5,1	6.74	-4.30	*no*	7420	Rmet_5438	*0*	Op1618f_1	MET-LIP-FAMet
4633	TSS_2431185+3	25	6	-35	TTCTCATCGT	-14	AGCAAAATGG	-141	1,1	7.20	11.10	s	147	Rmet_5672	*copR2*	Op1696f_1	EIP-SIG-2Comp
4634	TSS_2488354+3	25	5	-40	TTGCCACCTT	-11	TAGAGAATGG	-143	1,1	7.83	2.40	*(w)-sba*	4647	Rmet_6764	*0*	Op1716f_1	NA
4635	TSS_943840-3	25	3	-33	TTGCTGGCTG	-13	ATGACAATTG	-141	1,1	6.74	7.60	s	3414	Rmet_4343	*0*	Op1235r_1	NA
4636	TSS_2097236-3	25	8	-47	TTGCTTTAAT	-13	TCTAGGATGG	-138	1,1	8.33	-11.60	*no*	120	Rmet_5371	*katG*	Op1599r_1	MET-EN-Methane
4637	TSS_106170+4	25	8	-28	AGACTCGCTG	-8	AACGGTTTAG	-145	3,0	5.77	-7.40	*no*	3297	Rmet_5950	*int*	Op1809f_1	NA
4638	TSS_227318+4	25	7	-42	TCGTCCAGAC	-14	GCTAAAGTCG	-141	1,1	6.77	-1.10	*no*	1648	Rmet_6071	*0*	Op1849f_1	NA
4639	TSS_117403-4	25	2	-43	CCGTCACCAT	-14	TGTATGATCT	-141	5,1	8.63	-4.10	*no*	2907	Rmet_5947	*pbrA*	Op1808r_1	MET-EN-P_ATPase
4640	TSS_203822-4	25	6	-47	TTCCCGACCC	-12	GGTAGATTGG	-141	1,1	7.61	-13.10	*no*	2142	Rmet_6099	*0*	Op1860r_1	NA
4641	TSS_142599-5	25	2	-40	TGGCCAACTG	-13	GGTACACTGA	-138	1,1	8.98	2.90	*(w)-sba*	4124	Rmet_6282	*tnpA*	Op1915r_1	NA
4642	TSS_295411+2	24	8	-42	TTCCACTGTA	-15	TGTATCCTTT	-140	1,1	7.03	-1.10	*no*	788	Rmet_0280	*maiA*	Op0081f_1	MET-AA-Tyr
4643	TSS_461878+2	24	5	-48	AACCGTTGGG	-15	AGCATCATTC	-141	2,1	7.98	-17.60	*no*	1376	Rmet_0437	*surA*	Op0117f_1	MET-COF-B6
4644	TSS_513422+2	24	2	-36	TTGTGGAGAC	-15	GCTATCGTTT	-141	1,1	6.94	10.80	s	1153	Rmet_0487	*0*	Op0131f_1	NA
4645	TSS_1627021+2	24	5	-39	TGGCCAGCAA	-14	GGCAGCCTTG	-142	1,1	5.10	3.90	m	3959	Rmet_1509	*cbbS(del*	Op0425f_1	NA
4646	TSS_2021436+2	24	2	-46	TTGAATGATC	-25	CGAACAATCG	-145	1,1	7.10	1.80	*w*	254	Rmet_1864	*0*	Op0539f_1	NA
4647	TSS_2257859+2	24	5	-48	TTGACAGCGG	-17	TTAATAATGA	-140	1,1	7.92	-5.60	*no*	377	Rmet_2080	*nirF*	Op0601f_3	NA
4648	TSS_3129147+2	24	9	-39	TGCCTCGGTC	-14	GCTATTTTCG	-145	1,1	4.47	2.40	w	1170	Rmet_2879	*tnpA*	Op0809f_1	NA
4649	TSS_3585353+2	24	3	-36	TTGCCGTCCT	-13	GATAGTCTGG	-138	1,1	7.64	10.40	s	320	Rmet_3322	*0*	Op0927f_1	NA
4650	TSS_217352-2	24	3	-42	TTGAACGGCA	-13	GGCATGATAC	-139	1,1	7.94	-0.60	*no*	3257	Rmet_0204	*0*	Op0060r_1	NA
4651	TSS_1070029-2	24	5	-42	GTACAACAAT	-5	CGCATGATCG	-140	4,1	7.57	-13.10	*no*	1262	Rmet_0982	*bug*	Op0276r_1	NA
4652	TSS_1366315-2	24	11	-47	TGGCACGCGC	-13	GATACGGTGA	-139	1,1	5.49	-14.10	*no*	10792	Rmet_1232	*yhgF*	Op0356r_1	NA
4653	TSS_1984734-2	24	7	-33	GGATTTGCTG	-13	CGTACAATAC	-140	3,1	11.58	2.60	w	190	Rmet_1828	*tmk*	Op0530r_2	NA
4654	TSS_2435086-2	24	8	-40	GCGGCCTCGG	-13	GCTACCATTT	-138	5,1	9.11	-1.10	*no*	3383	Rmet_2217	*0*	Op0634r_1	NA
4655	TSS_3257820-2	24	5	-36	TCGCCTCCGA	-13	GCCATACTGT	-141	1,1	6.76	9.90	s	480	Rmet_2998	*0*	Op0844r_1	NA
4656	TSS_3349332-2	24	8	-37	CCGGCATGCT	-14	GATACACTTC	-136	5,1	10.62	6.90	s	86	Rmet_3090	*pehR*	Op0870r_2	GIP-PTL-T2S
4657	TSS_3495720-2	24	3	-45	GCAGCCGGGG	-13	CGTAACATGA	-140	5,1	9.08	-12.60	*no*	12	Rmet_6586	*0*	Op0908r_1	NA
4658	TSS_152041+3	24	7	-49	TAGCTGATGG	-13	CGTATGATGC	-143	1,1	5.09	-16.10	*no*	1587	Rmet_R0074	*0*	Op1006f_1	NA
4659	TSS_472716+3	24	3	-36	TTGCGTTCCG	-15	TCTAATATTC	-142	2,1	11.15	10.80	s	428	Rmet_R0075	*0*	Op1098f_1	NA
4660	TSS_577859+3	24	4	-47	TCGATCCAGT	-15	GATATCCTTG	-143	1,1	6.72	-10.10	*no*	4389	Rmet_6640	*0*	Op2063f_1	NA
4661	TSS_869551+3	24	2	-46	GCAGGAAACG	-15	AATACCATGG	-138	4,1	8.13	-12.60	*no*	4423	Rmet_4282	*0*	Op1216f_1	NA
4662	TSS_1758796+3	24	2	-28	TTCAATCGAT	0	AGCACCTTTC	-144	1,1	3.58	-4.30	*no*	1254	Rmet_5071	*0*	Op1492f_3	NA
4663	TSS_2110476+3	24	9	-36	TCGACACTCC	-15	GGCAAGATCA	-138	1,1	6.20	11.30	s	128	Rmet_5384	*0*	Op1602f_2	NA
4664	TSS_2280434+3	24	5	-36	TTGCGCATTG	-15	CGTACGCTGC	-140	1,1	7.74	9.80	s	2564	Rmet_5536	*0*	Op1646f_1	NA
4665	TSS_551606-3	24	5	-36	TCGTCAACGC	-13	GTTACCATCT	-140	1,1	7.31	10.90	s	4304	Rmet_3980	*livH4*	Op1115r_1	EIP-TRA-ABC
4666	TSS_566286-3	24	8	-40	TGGCGACAGC	-13	CGCATCATTG	-141	1,1	6.43	1.90	*(w)-sba*	40	Rmet_3995	*bglX*	Op1121r_1	MET-CAH-Starch
4667	TSS_1164002-3	24	10	-49	TTGTAGTCCC	-14	GATAGAGTTG	-144	1,1	7.30	-14.60	*no*	3205	Rmet_4549	*uvrA2*	Op1305r_1	GIP-REP-Recomb
4668	TSS_1164025-3	24	2	-34	TGGCGTTTTT	-13	GCGACCATGT	-146	1,1	5.88	7.10	s	3228	Rmet_4549	*uvrA2*	Op1305r_1	GIP-REP-Recomb
4669	TSS_1181067-3	24	9	-30	TCGCATCGCA	-7	GAAACGAAGC	-152	1,1	-0.98	0.70	*(w)-nu*	0	Rmet_R0082	*0*	Op1315r_2	NA
4670	TSS_1387401-3	24	11	-43	TCAGCAACAT	-13	GGCACACTAT	-143	5,1	8.24	-5.60	*no*	2441	Rmet_4739	*nnrU*	Op1373r_1	NA
4671	TSS_1761456-3	24	5	-42	TTGTCTCGCT	-18	CTTATGTTCC	-146	1,1	6.01	4.40	*(m)-sba*	533	Rmet_5072	*0*	Op1493r_1	MET-AA-Met
4672	TSS_89563+4	24	6	-49	TTGAGCTTTG	-25	GGCACACTAG	-141	1,1	7.79	-3.60	*no*	41	Rmet_5970	*czcP*	Op1817f_1	MET-EN-P_ATPase
4673	TSS_211357-4	24	3	-44	TTGATGTCGT	-13	GAGATCCTGG	-140	1,1	6.22	-5.60	*no*	979	Rmet_6092	*0*	Op1856r_1	NA
4674	TSS_14947+5	24	5	-47	TGCCGGCGGC	-14	GCGACACTCT	-143	1,1	4.74	-14.60	*no*	1583	Rmet_6327	*0*	Op1922f_1	NA
4675	TSS_166880-5	24	2	-38	GCTGCTGCGC	-13	TGAATAATTG	-144	5,1	7.44	2.40	w	9521	Rmet_6301	*trbF*	Op1917r_1	NA
4676	TSS_1987+2	24	3	-47	TACTGGGTGC	-14	GGTAAACTGT	-143	1,1	3.62	-13.60	*no*	232	Rmet_0002	*dnaN*	Op0001f_2	GIP-REP-DNA_Pol
4677	TSS_349841+2	24	7	-36	TTCACGAACT	-15	TTCAGAATCT	-140	1,1	7.04	11.30	s	6008	Rmet_0338	*0*	Op0095f_1	NA
4678	TSS_1002918+2	24	4	-37	GCCCAGGAGC	-13	GACAAACTCA	-144	4,1	7.53	1.40	w	2389	Rmet_0920	*0*	Op0265f_1	EIP-TRA-ABC
4679	TSS_1010618+2	24	10	-37	TGGCGACATC	-15	ATTACCATCA	-139	1,1	6.60	9.90	s	321	Rmet_0925	*cbbJ2*	Op0267f_2	MET-CAH-FbP
4680	TSS_1469948+2	24	4	-42	GCAGGCGCGC	-15	GGTAAAATCA	-138	4,1	11.09	-4.60	*no*	110	Rmet_1361	*0*	Op0383f_3	NA
4681	TSS_1624188+2	24	7	-35	TTCCCGAGCG	-14	ACTAAGCTTA	-143	1,1	5.71	9.10	s	6792	Rmet_1509	*cbbS(del*	Op0425f_1	NA
4682	TSS_1703042+2	24	8	-35	TTGTGGTTGG	-14	TCTACGCTTG	-141	1,1	7.03	9.60	s	38	Rmet_1582	*0*	Op0437f_1	NA
4683	TSS_1817411+2	24	5	-34	TTGATGACGT	-14	GTTAAAATCA	-137	1,1	10.31	10.60	s	1826	Rmet_1682	*0*	Op0475f_1	NA
4684	TSS_2201640+2	24	11	-25	TCGTAGCCCA	-1	GTTTCGATCA	-149	1,1	-0.29	-4.30	*no*	4615	Rmet_2037	*fnrL*	Op0595f_1	NA
4685	TSS_2578575+2	24	3	-35	TTGAGCCCAG	-14	AGTAATCTAC	-144	1,1	6.16	10.60	s	190	Rmet_2366	*0*	Op0667f_1	MET-CAH-Prop
4686	TSS_3570223+2	24	3	-40	CCGTAAATGG	-14	GGTATGCTTG	-141	5,1	7.64	-0.10	*no*	15450	Rmet_3322	*0*	Op0927f_1	NA
4687	TSS_2059078-2	24	8	-40	GCGGAACGCC	-10	ATAACAGTCC	-142	5,1	5.82	-5.10	*no*	1134	Rmet_6486	*0*	Op0546r_1	NA
4688	TSS_2322661-2	24	1	-46	TTGAAGGCGG	-14	GCTACCTTAC	-141	1,1	6.84	-8.60	*no*	383	Rmet_2133	*0*	Op0614r_1	NA

4689	TSS_2384439-2	24	6	-44	TGGCGATGTG	-24	CATAGAATCG	-142	1,1	8.29	2.30	w	88	Rmet_2177	ppx		Op0626r_1	MET-NUC-Pur
4690	TSS_2793651-2	24	10	-38	TCGTACACAT	-13	GGTACTGTTT	-143	1,1	4.92	3.90	m	1376	Rmet_2566		0	Op0710r_1	NA
4691	TSS_3257881-2	24	6	-42	TGCTCCGGTG	-20	GAAAAAATGT	-145	1,1	4.13	3.40	m	541	Rmet_2998		0	Op0844r_1	NA
4692	TSS_3315235-2	24	10	-23	TGCCCGGGCT	-2	GAGATCCTGC	-142	1,1	4.00	-4.40	no	316	Rmet_3056		0	Op0860r_2	GIP-TL-Ass
4693	TSS_3428093-2	24	4	-27	AAACGTCGTG	-7	GTCCCTATCT	-147	3,0	4.25	-7.40	no	407	Rmet_R0050		0	Op0886r_1	NA
4694	TSS_3580812-2	24	4	-44	TGCAGGGCGT	-12	GGTACCATCA	-140	1,1	6.49	-8.60	no	278	Rmet_3316	rplD		Op0926r_2	GIP-TL-Ribo
4695	TSS_3644919-2	24	9	-34	TTCCTCACAC	-13	AGTACGCTCG	-144	1,1	5.63	7.10	s	2012	Rmet_3367		0	Op0936r_1	NA
4696	TSS_3882227-2	24	6	-35	TGGAGTCGCA	-14	CCCACAATGC	-140	1,1	6.84	10.10	s	4378	Rmet_3574	cstA		Op0992r_1	NA
4697	TSS_3907364-2	24	9	-42	TTGACGCGAC	-14	GGTAGGCTAC	-140	1,1	7.91	0.40	(w)-sba	1060	Rmet_3594		0	Op1000r_2	NA
4698	TSS_152730+3	24	10	-39	TTGCATTGGA	-14	TGTAGGATAG	-142	1,1	8.16	5.40	m	898	Rmet_R0074		0	Op1006f_1	NA
4699	TSS_482260+3	24	5	-36	TGGCCAGAGT	-14	TTTATTCTGC	-140	1,1	7.65	11.90	s	1247	Rmet_3925		0	Op1098f_2	NA
4700	TSS_484780+3	24	3	-43	TCAGGACTAC	-14	GGTACAGTAC	-142	4,1	7.69	-4.60	no	7038	Rmet_3934		0	Op1100f_1	EIP-SIG-2Comp
4701	TSS_557351+3	24	7	-49	TGGCATTTGG	-25	CTGAGAGTGT	-142	1,1	3.90	-6.10	no	167	Rmet_3988		0	Op1118f_1	NA
4702	TSS_106086-3	24	8	-34	CCGGCCACCC	-13	GGCAAACTGA	-136	5,1	9.08	5.10	m	49	Rmet_5905		0	Op1791r_1	NA
4703	TSS_476795-3	24	11	-47	TTGCCGAGTT	-12	CTTAGAATAC	-141	1,1	9.67	-10.60	no	3714	Rmet_6633		0	Op1097r_1	NA
4704	TSS_516280-3	24	6	-45	GGAGACGAAC	-14	GCGAGAATCG	-141	4,1	8.64	-12.60	no	2888	Rmet_3953		0	Op1109r_1	NA
4705	TSS_1112172-3	24	6	-42	CCGAAAATAC	-14	GCCACAATGG	-143	5,1	7.92	-3.10	no	430	Rmet_4508		0	Op1287r_1	NA
4706	TSS_1370699-3	24	4	-38	TGACACCCCC	-13	GTCATACTGG	-143	4,1	6.50	3.40	m	2991	Rmet_4724	cls		Op1363r_1	MET-LIP-GlycPLip
4707	TSS_1535538-3	24	10	-38	TATCGTAGCG	-13	GGTACGCTTG	-135	2,1	11.20	2.40	w	319	Rmet_4872		0	Op1423r_1	NA
4708	TSS_90192+4	24	2	-41	GGCAGCGTGT	-15	GCTAATCTTC	-142	4,1	6.86	-3.60	no	6101	Rmet_5965	tnpA		Op1815f_1	NA
4709	TSS_43088-4	24	4	-35	TCAGGAACGC	-8	CACATAGTGA	-144	4,1	6.65	4.20	(m)-ba	4473	Rmet_6359		0	Op2108r_1	NA
4710	TSS_220597-4	24	5	-42	TTGATACACC	-12	GAGAGAATTC	-141	1,1	7.94	-0.60	no	8108	Rmet_6089		0	Op1854r_1	NA
4711	TSS_618-5	24	7	-41	TTCCTCTGCC	-14	CATAAACTCC	-140	2,1	10.44	-0.10	no	14718	Rmet_6301	trbF		Op1917r_1	NA
4712	TSS_270194+2	24	3	-44	TTCCTGACAA	-14	GTCATTATCG	-142	1,1	5.79	-6.10	no	527	Rmet_0253		0	Op0073f_2	GIP-REP-Recomb
4713	TSS_1441025+2	24	6	-43	GGAACCCAGG	-14	GTTATAGTTA	-142	4,1	7.27	-5.60	no	3193	Rmet_1332		0	Op0377f_1	NA
4714	TSS_1508425+2	24	10	-38	TCGACGTACG	-14	AGCAGAATGG	-140	1,1	6.82	7.90	s	914	Rmet_1400	dadX		Op0393f_1	MET-AA-AlaAsp
4715	TSS_1790880+2	24	10	-35	TCCACCGGTG	-13	GTAAGAATGC	-144	1,1	4.83	10.20	s	5119	Rmet_1661		0	Op0469f_1	NA
4716	TSS_2185806+2	24	6	-37	TTGCGCAGGC	-14	GGCAACATCG	-138	1,1	7.77	8.40	s	1273	Rmet_2023		0	Op0591f_1	NA
4717	TSS_2878413+2	24	7	-35	TGGCTGATCG	-13	GCAACCATTT	-140	1,1	4.43	8.70	s	1252	Rmet_2642	pdxH		Op0745f_2	MET-COF-B6
4718	TSS_3096015+2	24	6	-36	TCGCACGCGG	-15	GGCACAATTC	-140	1,1	7.84	9.30	s	1319	Rmet_2849	yggG		Op0803f_1	GIP-PTL-Fold
4719	TSS_3170855+2	24	9	-46	TTCAACCCGG	-16	CGTACACTAC	-141	1,1	7.28	-7.10	no	188	Rmet_2917	panB		Op0825f_3	MET-COF-Pant
4720	TSS_3255176+2	24	4	-47	TGCTGCGCCA	-16	GATATGCTCC	-141	1,1	4.46	-11.60	no	2306	Rmet_2999	mmfA		Op0845f_1	NA
4721	TSS_200418-2	24	5	-49	TCGTTGGCCC	-29	GATATTCTGG	-141	1,1	5.12	-4.70	no	7960	Rmet_0185		0	Op0056r_1	NA
4722	TSS_629822-2	24	2	-36	TGGCTGACGC	-13	GGCATCATGG	-139	1,1	6.85	8.90	s	4287	Rmet_0583		0	Op0164r_1	NA
4723	TSS_1687370-2	24	6	-47	TCGACGTGAT	-13	TGTAGGCTCG	-139	1,1	6.50	-12.10	no	18782	Rmet_1545		0	Op0430r_1	NA
4724	TSS_1697126-2	24	10	-44	TCGATGAACC	-12	GCTACCATTG	-142	1,1	7.66	-7.10	no	245	Rmet_1576		0	Op0434r_1	NA
4725	TSS_1780514-2	24	4	-26	TTCACTCCCC	-2	TCCATCATCT	-141	1,1	5.58	0.70	(w)-nu	149	Rmet_1645	polB		Op0462r_1	GIP-REP-DNA_Pol
4726	TSS_2122978-2	24	3	-35	TGGAACGATG	-14	TCCATGATTT	-141	1,1	6.29	10.10	s	3249	Rmet_6494		0	Op2020r_1	NA
4727	TSS_2771657-2	24	3	-41	TTGTAGCGAA	-13	GCTACGCTTC	-143	1,1	7.65	-0.60	no	256	Rmet_2546		0	Op0706r_1	NA
4728	TSS_2929706-2	24	6	-35	TGGCGCGACG	-13	GTGACGATAC	-144	1,1	4.93	8.70	s	573	Rmet_2697		0	Op0760r_1	NA
4729	TSS_3612961-2	24	7	-29	GCAACACAAC	-4	CCCAGATTCG	-145	4,1	6.53	-1.80	no	42	Rmet_R0060		0	Op0930r_1	NA
4730	TSS_3630536-2	24	9	-40	TTGCTGAATC	-14	CATACAATCC	-139	1,1	9.57	4.40	(m)-sba	2	Rmet_3353	tnpA		Op0934r_1	NA
4731	TSS_3639647-2	24	9	-35	TTGTGGAGAC	-14	GCTATCGTTT	-141	1,1	6.94	10.60	s	197	Rmet_3363		0	Op0936r_2	NA
4732	TSS_3656218-2	24	3	-42	TTAAAAGCGC	-11	GTTCTAATCC	-145	4,1	6.80	-3.10	no	2	Rmet_3380		0	Op0940r_3	NA
4733	TSS_434605+3	24	6	-43	TTGTGGCTGG	-15	CCTATAGTCC	-142	1,1	7.41	-0.60	no	42	Rmet_3890		0	Op1084f_1	EIP-TRA-ABC
4734	TSS_476158+3	24	5	-47	TCACGGGAGA	-25	GCTAACGTCC	-145	4,1	4.84	-3.60	no	2020	Rmet_R0079		0	Op1098f_1	NA
4735	TSS_477510+3	24	2	-49	TCGGACATCG	-13	GCTTAACTGC	-148	5,1	4.05	-19.10	no	668	Rmet_R0079		0	Op1098f_1	NA
4736	TSS_873953+3	24	2	-36	TTGACACGCA	-13	CGTATGATGC	-140	1,1	9.95	14.40	s	21	Rmet_4282		0	Op1216f_1	NA
4737	TSS_1271458+3	24	2	-37	TGGCCTTCAC	-15	GGCACAGTGG	-142	1,1	6.24	8.90	s	5706	Rmet_4647		0	Op1342f_1	NA
4738	TSS_1378967+3	24	5	-46	TCGCGCTCAG	-14	GTCACAATGC	-141	1,1	7.60	-10.10	no	1618	Rmet_4736	kdgD		Op1370f_1	MET-CAH-Ascorb
4739	TSS_1516813+3	24	7	-45	TTGAGGCCAT	-14	GCGACAATCA	-139	1,1	8.16	-5.60	no	222	Rmet_4858		0	Op1418f_1	NA
4740	TSS_1541162+3	24	9	-42	TTGCCCAGGA	-10	GAAAAGCTTC	-142	1,1	5.39	-5.60	no	1890	Rmet_4881	catA		Op1428f_1	MET-XEN-Toluene
4741	TSS_1763012+3	24	7	-45	TTCCAATATC	-15	GCTATCATCT	-143	1,1	9.10	-5.10	no	1866	Rmet_5076		0	Op1494f_1	NA
4742	TSS_135117-3	24	8	-38	GGACATACGG	-13	GCTACGCTAG	-146	4,1	1.91	-0.60	no	28	Rmet_5930	fusA2		Op1801r_2	NA
4743	TSS_356502-3	24	8	-34	TTCCCGAGTT	-13	CCTATGATTA	-138	1,1	8.02	10.10	s	698	Rmet_3814		0	Op1061r_1	NA
4744	TSS_546390-3	24	6	-38	GGAGACCACC	-13	GGCAAAATCG	-140	4,1	8.28	0.40	w	992	Rmet_3978	livG4		Op1115r_1	EIP-TRA-ABC
4745	TSS_1365197-3	24	8	-43	TTCCGAAATT	-17	GCTACTGTCT	-145	1,1	6.02	-1.10	no	41	Rmet_4722		0	Op1363r_2	NA
4746	TSS_1380239-3	24	2	-47	GTAAACGCGC	-14	CTTATAATTC	-141	4,1	11.37	-12.10	no	73	Rmet_4735	garP		Op1369r_1	NA
4747	TSS_1388191-3	24	7	-43	GGAACGCTAC	-15	ACTACTATAA	-140	4,1	6.32	-5.60	no	53	Rmet_4743		0	Op1375r_2	NA
4748	TSS_1406879-3	24	6	-44	TGGCCGCTGT	-13	GTTAGTTTCC	-142	1,1	5.66	-7.10	no	515	Rmet_4760		0	Op1385r_1	NA
4749	TSS_1418747-3	24	5	-48	GCGTCAGCGC	-13	GCCATCATCT	-141	5,1	7.09	-16.10	no	3318	Rmet_4770		0	Op1387r_1	MET-AA-GlySerThr

4750	TSS_1592207-3	24	8	-45	GCTGAAGGCC	-14	ACGATCATGC	-141	5,1	7.88	-11.60	no	5499	Rmet_4924	0	Op1443r_1	NA	
4751	TSS_1926616-3	24	3	-42	TTGCGTACTG	-13	GCTACGCTGT	-139	1,1	7.84	-2.60	no	1681	Rmet_5228	0	Op1541r_1	NA	
4752	TSS_1951377-3	24	1	-40	TGGAGCGAGT	-14	GGTATGCTGC	-140	1,1	8.32	2.90	(w)-sba	522	Rmet_5250	0	Op1549r_1	DIV-MOT-Chemotax	
4753	TSS_2242780-3	24	5	-36	TTGAGTCAGT	-13	GATATCATTG	-142	1,1	9.60	12.40	s	126	Rmet_5498	0	Op1635r_1	NA	
4754	TSS_2246178-3	24	3	-35	TTGATTGGTC	-12	TGCAATCTGT	-146	1,1	5.04	9.20	s	3524	Rmet_5498	0	Op1635r_1	NA	
4755	TSS_2250427-3	24	6	-41	TTGTATTACG	-13	GCTATGGTAC	-142	1,1	6.46	0.40	(w)-sba	1941	Rmet_5504	0	Op1637r_1	NA	
4756	TSS_10544+4	24	8	-46	TGGCCCGATA	-15	GTTAAGGTGT	-143	1,1	5.86	-9.10	no	397	Rmet_6348	0	Op1845f_2	NA	
4757	TSS_60495+4	24	2	-38	TTGACTGACA	-14	GATATCCTTT	-143	1,1	8.15	9.40	s	3315	Rmet_5994	*gntR*	Op1827f_1	NA	
4758	TSS_211398+4	24	5	-42	TTCCCCCCAG	-15	GGTAGTATCG	-139	1,1	7.20	-0.10	no	1312	Rmet_6387	0	Op1853f_1	NA	
4759	TSS_1013246+2	23	6	-42	TCGATGGGGT	-14	GCTACTATCA	-139	1,1	7.07	-1.10	no	150	Rmet_0929	*nuoC*	Op0267f_3	MET-EN-OxPhos	
4760	TSS_1052981+2	23	3	-37	TCGAATTGCA	-14	GTTACCTTTT	-143	1,1	5.53	7.90	s	222	Rmet_0969	0	Op0271f_1	NA	
4761	TSS_1072596+2	23	5	-34	TTGCAAGGGG	-5	GAAACAATGG	-139	1,1	8.28	4.20	(m)-ba	391	Rmet_0987	0	Op0279f_1	NA	
4762	TSS_2152925+2	23	9	-30	TTGATGACTT	-3	TGCATCGTGT	-146	1,1	5.19	1.20	(w)-nu	2332	Rmet_1981	*lexA*	Op0577f_1	GIP-TK-Assoc	
4763	TSS_2363582+2	23	1	-32	TTCTCCGACC	-6	TGCATATTTT	-144	1,1	5.02	3.70	(m)-ba	112	Rmet_2163	0	Op0623f_1	NA	
4764	TSS_2376281+2	23	5	-35	TCCACCTGCT	-13	TAGATAATGA	-144	1,1	5.78	11.20	s	3595	Rmet_R0034	0	Op0625f_1	NA	
4765	TSS_2654045+2	23	4	-37	GCACGAAAGC	-14	GTTAGACTTC	-137	4,1	11.32	4.40	m	84	Rmet_2443	*moeA*	Op0683f_1	MET-COF-Folate	
4766	TSS_3081891+2	23	4	-45	TCGAAGGGCG	-20	CCTATGGTGT	-142	1,1	5.32	-1.10	no	297	Rmet_2833	0	Op0797f_2	NA	
4767	TSS_3338157+2	23	5	-38	TTGCCTCATG	-3	GACAAAATCG	-145	1,1	8.45	-2.60	no	924	Rmet_3084	*rdgC*	Op0867f_1	GIP-REP-Recomb	
4768	TSS_80473-2	23	6	-42	GCGGATGGGG	-18	TCTGACATGT	-147	5,1	1.60	-3.10	no	83	Rmet_0072	0	Op0020r_1		0
4769	TSS_162137-2	23	2	-36	GTACCCGCGC	-13	CCCATACTCG	-137	4,1	7.04	6.90	s	1757	Rmet_0155	0	Op0044r_1		0
4770	TSS_195797-2	23	10	-35	TGGCACAAAT	-12	CACAAAATCG	-142	1,1	7.09	8.70	s	3339	Rmet_0185	0	Op0056r_1	NA	
4771	TSS_327861-2	23	4	-35	TTGCTGCCCT	-14	TGCAGGATCA	-140	1,1	5.46	9.60	s	1268	Rmet_0310	0	Op0088r_1	GIP-PTL-Fold	
4772	TSS_443044-2	23	3	-49	GCGGCACGCC	-13	GCAACCATTC	-139	5,1	8.25	-19.10	no	322	Rmet_0416	0	Op0114r_1	MET-AA-PheTyrTrp	
4773	TSS_1126154-2	23	5	-46	TTCCCGGACG	-23	GGTATCTTTG	-142	1,1	6.61	-0.10	no	1143	Rmet_1035	0	Op0296r_1	NA	
4774	TSS_1491065-2	23	2	-41	CCTGCCCCAC	-13	CAGATACTGG	-140	5,1	7.68	-4.60	no	13048	Rmet_1368	*serB*	Op0384r_1	MET-AA-GlySerThr	
4775	TSS_1533576-2	23	5	-45	GCCAGCATAC	-12	ATTAACATCC	-142	4,1	8.26	-13.60	no	16785	Rmet_1404	*yheS*	Op0396r_1	GIP-TL-Ass	
4776	TSS_2148250-2	23	10	-35	TTGAAATTGG	-13	CTTCCAATGT	-141	1,1	5.87	12.20	s	3734	Rmet_1967	*aatA*	Op0574r_1	NA	
4777	TSS_2164458-2	23	6	-46	TGGACTGAAC	-13	GTAAGAATTT	-142	1,1	6.17	-9.10	no	1344	Rmet_1989	*dus*	Op0582r_1	GIP-TL-Ass	
4778	TSS_2274527-2	23	6	-33	TTGATACCGG	-9	TCTGTGATCT	-142	1,1	2.25	8.20	(s)-ba	1152	Rmet_2092	*mr*	Op0606r_1	NA	
4779	TSS_2699107-2	23	1	-37	TGGCAACATG	-14	GGCACTATCA	-140	1,1	6.01	7.90	s	1590	Rmet_2483	*sdhB*	Op0688r_2	MET-CAH-TCA	
4780	TSS_2961048-2	23	9	-47	GGAGCAGAGA	-17	CCTATAATAC	-143	4,1	9.06	-9.60	no	29	Rmet_2724	0	Op0770r_2	NA	
4781	TSS_3614219-2	23	2	-45	GAACTCGGCA	-25	GTAACTTCGG	-145	3,0	6.42	-9.20	no	1300	Rmet_R0060	0	Op0930r_1	NA	
4782	TSS_26671+3	23	9	-37	TTGACCCAGC	-5	GAAACGCTGG	-141	1,1	5.48	0.40	(w)-ba	15	Rmet_5839	0	Op1764f_2	NA	
4783	TSS_118255+3	23	4	-27	TCGGAAGTCT	-1	CGTATTTTGC	-142	5,1	7.76	-1.30	no	151	Rmet_5917	0	Op1796f_2	NA	
4784	TSS_1253319+3	23	3	-43	TCCCCTTAAT	-13	GTCACAATCA	-142	1,1	6.18	-5.60	no	3722	Rmet_4628	*qor*	Op1334f_1	NA	
4785	TSS_1688881+3	23	7	-37	TCGCCGAATG	-15	GATACCGTCG	-140	1,1	5.39	8.90	s	2308	Rmet_5012	0	Op1474f_1	NA	
4786	TSS_1928154+3	23	7	-38	GTGAGAAATGC	-3	TCCAAAATCC	-144	4,1	9.61	-7.10	no	371	Rmet_5231	0	Op1544f_1	NA	
4787	TSS_307331-3	23	4	-41	TCGTCCAGAC	-13	GCTAAAGTCG	-141	1,1	6.77	-0.10	no	834	Rmet_3769	*alkB1*	Op1047r_1	NA	
4788	TSS_324899-3	23	7	-41	TCGACTCCAT	-13	GCCAGAATCG	-138	1,1	7.12	0.90	(w)-sba	13113	Rmet_3775	0	Op1049r_1	NA	
4789	TSS_433620-3	23	1	-34	TTGTAATTAC	-13	CCTATACTGC	-144	1,1	9.79	11.60	s	63	Rmet_3888	0	Op1083r_1	NA	
4790	TSS_562636-3	23	10	-38	TGCCTTTGCC	-14	GGTAGAATGC	-138	1,1	7.93	5.40	m	2066	Rmet_3991	*arsB1*	Op1119r_1	EIP-TRA-Ion	
4791	TSS_891363-3	23	5	-47	TTCCGATACT	-14	ACTACAGTTC	-142	1,1	7.24	-11.10	no	191	Rmet_4297	0	Op1223r_1	NA	
4792	TSS_1171290-3	23	5	-36	TTCAGAATGA	-13	TGTAGATTTG	-146	1,1	7.32	10.90	s	27	Rmet_4561	0	Op1311r_1	NA	
4793	TSS_1197104-3	23	3	-43	TCGTGCGTCC	-13	GTTAAAGTCC	-139	1,1	5.95	-5.10	no	9212	Rmet_4573	0	Op1315r_1	NA	
4794	TSS_2311936-3	23	6	-44	TCGCGGATCG	-23	GAAATACTGG	-142	1,1	5.39	1.30	w	3211	Rmet_6746	0	Op2099r_1	NA	
4795	TSS_1992+4	23	6	-45	TGCACCGAGC	-18	GTTAAGGTAG	-144	1,1	4.42	-4.60	no	824	Rmet_6061	0	Op1847f_1	NA	
4796	TSS_437823+2	23	4	-36	TTCATCAAGT	-15	GATATCATCG	-139	1,1	8.31	11.30	s	517	Rmet_0412	0	Op0113f_2	NA	
4797	TSS_508036+2	23	1	-47	TTCACCTGCA	-24	ACTAGGCTGT	-141	1,1	5.89	-1.10	no	1551	Rmet_0481	0	Op0127f_1	NA	
4798	TSS_800196+2	23	2	-45	TGGGCATGAA	-15	GGGACGATTT	-149	1,1	0.13	-6.10	no	637	Rmet_0723	*ihfB*	Op0205f_2	GIP-REP-Recomb	
4799	TSS_1012113+2	23	3	-34	CCGGCCGTGC	-14	GCAATAATTG	-137	5,1	9.53	6.10	m	180	Rmet_R0013	0	Op0267f_3	NA	
4800	TSS_1269925+2	23	3	-43	TTTTTTTTCG	-13	AGTAAGCTGC	-141	2,1	10.53	-6.10	no	67	Rmet_1160	*thrS*	Op0333f_2	MET-AA-GlySerThr	
4801	TSS_1564322+2	23	5	-35	TCGCTGGGTC	-14	GCCATTATTG	-143	1,1	5.89	9.10	s	2274	Rmet_1444	*hlpA*	Op0403f_3	NA	
4802	TSS_2294696+2	23	4	-37	TGCATCGAGC	-14	GCCAAAATGC	-139	1,1	6.67	7.40	s	369	Rmet_2113	0	Op0611f_1	NA	
4803	TSS_2322512+2	23	3	-45	TGCCAATAGC	-12	ACTATCATCG	-143	1,1	7.13	-9.60	no	228	Rmet_2134	*trxA*	Op0615f_1	GIP-PTL-Fold	
4804	TSS_2397670+2	23	1	-37	TTCCCCCCGC	-16	CGCATCATGT	-140	1,1	6.04	9.30	s	1374	Rmet_2190	0	Op0629f_1	NA	
4805	TSS_2582975+2	23	8	-33	TTGAGTTAAT	-8	TCTGGCATGT	-148	1,1	1.88	5.20	(m)-ba	1007	Rmet_2373	0	Op0669f_1	NA	
4806	TSS_2658818+2	23	1	-43	CCGTACCGGC	-14	GGGATAATCG	-140	5,1	8.06	-6.10	no	3795	Rmet_6527	0	Op0685f_1	NA	
4807	TSS_3232595+2	23	5	-39	GCGGCCACGG	-15	CCCATAATCG	-135	5,1	9.80	3.90	m	2685	Rmet_2976	*fur*	Op0837f_1	NA	
4808	TSS_3499981+2	23	4	-28	GCGGCAGGCC	-7	TATATCCATC	-144	5,1	4.54	-0.90	no	893	Rmet_3225	0	Op0913f_1	NA	
4809	TSS_3624698+2	23	6	-47	TGCCCAAGCT	-24	AGCAAATTGC	-144	1,1	4.77	-2.60	no	3700	Rmet_3352	0	Op0933f_1	NA	
4810	TSS_3818065+2	23	9	-48	GCGACAACTG	-25	ACCACCATTG	-145	5,1	4.99	-4.10	no	13121	Rmet_3533	*phhA*	Op0977f_1	MET-AA-PheTyrTrp	

4811	TSS_418946-2	23	7	-46	TAGCGACATC	-24	GGCAGGATGC	-144	1,1	3.58	-0.10	no	511	Rmet_0390	*yciA*	Op0106r_1	NA
4812	TSS_630304-2	23	5	-34	TGGATCCAGA	-12	GCCATACTAC	-141	1,1	6.54	9.70	s	4769	Rmet_0583	*0*	Op0164r_1	NA
4813	TSS_673848-2	23	5	-35	TGGTCCTCGT	-13	CGTACAGTAC	-143	1,1	6.10	10.70	s	8548	Rmet_0610	*0*	Op0172r_1	MET-LIP-FASyn
4814	TSS_1322619-2	23	5	-34	TTGTCTTCGC	-5	GTAAAGATCC	-144	1,1	5.51	3.20	(w)-ba	2264	Rmet_1200	*phaP*	Op0348r_1	NA
4815	TSS_1668292-2	23	2	-49	GCAAAGCCAG	-13	GATATCATTC	-142	4,1	9.55	-19.60	no	3365	Rmet_1543	*cbbA (de*	Op0428r_1	NA
4816	TSS_2352005-2	23	3	-34	TTGACCGCTA	-13	CCCAAGCTCG	-141	1,1	5.33	9.60	s	10013	Rmet_2148	*0*	Op0620r_1	NA
4817	TSS_2484471-2	23	1	-38	TCGCCAGCAT	-13	TGTATAATCA	-145	1,1	9.56	8.90	s	962	Rmet_6514	*0*	Op2031r_1	NA
4818	TSS_2648793-2	23	8	-38	GCACGCGAGG	-13	GGGAGAATTG	-140	4,1	7.90	0.40	w	866	Rmet_2439	*rluC*	Op0682r_1	GIP-TL-Ass
4819	TSS_3109216-2	23	3	-35	TTGACTTGTA	-14	GCTAAATTGC	-140	1,1	9.42	12.60	s	79	Rmet_2859	*tauA*	Op0804r_1	EIP-TRA-ABC
4820	TSS_3613874-2	23	1	-22	GCGGCTAGAC	-1	ATGAACCTTT	-146	5,1	4.91	-7.90	no	955	Rmet_R0060	*0*	Op0930r_1	NA
4821	TSS_477153+3	23	3	-34	GCACGAATGG	-14	GCCACACTGT	-142	4,1	8.40	2.60	w	1025	Rmet_R0079	*0*	Op1098f_1	NA
4822	TSS_539890+3	23	3	-27	GTAGAACACT	-3	TGTAGGGTGG	-146	4,1	5.83	-3.30	no	73	Rmet_3975	*0*	Op1114f_1	NA
4823	TSS_1569207+3	23	8	-36	TTCATATTAC	-15	ATTACAATCA	-144	1,1	8.46	12.30	s	32	Rmet_4906	*0*	Op1438f_1	NA
4824	TSS_2208719+3	23	15	-38	TTGCTCTGCG	-15	GATAGAATGG	-138	2,1	13.08	9.40	s	123	Rmet_5461	*0*	Op1622f_1	NA
4825	TSS_2425291+3	23	4	-42	TGGCGCACCC	-14	CCTAGAATCG	-141	1,1	8.08	-1.10	no	13	Rmet_5666	*0*	Op1694f_2	NA
4826	TSS_2486846+3	23	4	-34	TTGACGGCGC	-14	ACGAGAATGT	-140	1,1	7.51	10.60	s	6155	Rmet_6764	*0*	Op1716f_1	NA
4827	TSS_2500706+3	23	7	-47	TGGATCAATC	-14	ACTAAGATTG	-144	1,1	7.07	-11.10	no	464	Rmet_5739	*0*	Op1716f_2	NA
4828	TSS_336034-3	23	6	-37	TTGATTGTGC	-10	TTTACTGTTT	-146	1,1	5.68	5.40	m	34	Rmet_3796	*lipA*	Op1053r_1	MET-LIP-GlycLip
4829	TSS_976972-3	23	6	-38	TTGATATCTT	-14	GTAATAATGA	-141	1,1	8.34	10.40	s	5466	Rmet_4372	*0*	Op1247r_1	NA
4830	TSS_1653603-3	23	10	-42	TGGCGATGCA	-14	GATAAACTCA	-140	1,1	7.98	-1.10	no	4675	Rmet_4971	*0*	Op1459r_1	NA
4831	TSS_1690390-3	23	2	-43	TGGATCAGGT	-14	AGCATGATGG	-143	1,1	6.47	-3.10	no	1614	Rmet_5009	*0*	Op1471r_1	NA
4832	TSS_1764871-3	23	6	-35	TTGATGATTT	-11	AACATGATTG	-141	1,1	6.93	10.20	s	954	Rmet_5075	*msuE1*	Op1493r_1	NA
4833	TSS_2188278-3	23	3	-49	TTGCTACCGC	-13	AGTAGACTTC	-137	1,1	8.44	-14.60	no	1705	Rmet_5437	*0*	Op1617r_1	MET-CAH-Prop
4834	TSS_2385840-3	23	5	-35	TCAGATGGCG	-13	GGCATCATCG	-141	5,1	6.92	8.20	s	871	Rmet_5628	*tctB*	Op1679r_1	NA
4835	TSS_202100+4	23	5	-44	CCATCCCGAC	-1	GATATCCTCG	-144	5,1	7.07	-22.60	no	102	Rmet_6098	*npd-a*	Op1859f_2	NA
4836	TSS_221916-4	23	3	-39	TGGACCAGCG	-11	TCTAATCTCT	-140	1,1	6.09	1.90	w	9427	Rmet_6089	*0*	Op1854r_1	NA
4837	TSS_7116+2	23	3	-48	TGGCCTGACA	-21	GGTAAGTTTG	-145	1,1	6.00	-7.10	no	10279	Rmet_0013	*mazG*	Op0003f_1	NA
4838	TSS_804378+2	23	5	-38	TCGCAACATG	-16	GTCATTATTG	-144	1,1	6.47	8.90	s	158	Rmet_0727	*rfaE*	Op0205f_2	NA
4839	TSS_1935567+2	23	7	-47	TCGCCGGTGT	-14	GGCATGATCT	-141	1,1	7.05	-11.10	no	3430	Rmet_1788	*poxR*	Op0519f_1	NA
4840	TSS_2618716+2	23	6	-38	GCGGCCAGGC	-15	TAGAACATCG	-142	5,1	7.43	3.90	m	2778	Rmet_2411	*0*	Op0679f_1	NA
4841	TSS_2679216+2	23	4	-39	GCCAACGAAG	-15	GCGATACTCG	-140	4,1	7.61	1.40	w	22524	Rmet_2489	*mdh*	Op0689f_1	MET-CAH-TCA
4842	TSS_3046928+2	23	8	-45	TGGCGGCGGT	-25	GCGAAAATGC	-138	1,1	7.79	-0.70	no	84	Rmet_2800	*0*	Op0787f_2	NA
4843	TSS_3624711+2	23	3	-47	TCGCAACGTC	-14	GATACCGTAC	-144	1,1	5.20	-12.10	no	3687	Rmet_3352	*0*	Op0933f_1	NA
4844	TSS_87847-2	23	6	-36	TTGCCGCGCC	-13	CCTATAATTC	-136	1,1	10.91	13.40	s	121	Rmet_0080	*0*	Op0022r_1	MET-CAH-Prop
4845	TSS_1171523-2	23	6	-39	CTCACATGGC	-14	TCTACAATTC	-142	1,1	5.74	4.90	m	95	Rmet_1078	*suhB*	Op0310r_1	MET-CAH-Inos
4846	TSS_1241658-2	23	7	-40	TTCCCGGGTT	-12	GCTACGATGC	-137	1,1	8.82	0.90	(w)-sba	1345	Rmet_1133	*0*	Op0324r_1	NA
4847	TSS_1902740-2	23	4	-36	TTGACGATTG	-14	CCGATGATTC	-141	1,1	7.34	13.40	s	2116	Rmet_1750	*agrA*	Op0498r_1	NA
4848	TSS_2526845-2	23	5	-48	TGGAACGAAA	-13	TTCATAATTG	-142	1,1	7.96	-13.10	no	3713	Rmet_2302	*pbrR2*	Op0658r_1	NA
4849	TSS_2650083-2	23	3	-35	TTGCAGCGCG	-13	GCGAGCCTTC	-139	1,1	5.33	9.20	s	2156	Rmet_2439	*rluC*	Op0682r_1	GIP-TL-Ass
4850	TSS_3072238-2	23	6	-38	TTCCCTATCG	-15	GTCAATATTG	-142	1,1	5.81	6.90	s	1348	Rmet_2822	*0*	Op0794r_1	NA
4851	TSS_3429052-2	23	10	-40	GCAAACCGAC	-14	AGTATTCTAA	-147	5,1	4.63	-1.60	no	1366	Rmet_R0050	*0*	Op0886r_1	NA
4852	TSS_3502785-2	23	4	-23	TTGCTTTTTG	-2	TCGATGGTCG	-145	1,1	4.15	-2.40	no	2271	Rmet_3224	*0*	Op0912r_1	NA
4853	TSS_3578454-2	23	4	-38	TGACAAGAAA	-13	GCCATCATGG	-144	4,1	6.76	4.40	m	331	Rmet_3311	*rpsC*	Op0926r_2	GIP-TL-Ribo
4854	TSS_3590504-2	23	5	-39	TCGTAAGACT	-14	CGTAACATCG	-144	1,1	6.48	4.90	m	2144	Rmet_3324	*tuf*	Op0928r_2	NA
4855	TSS_3784502-2	23	8	-45	TAGCGAAGCC	-13	TGCAGAATCA	-143	1,1	3.95	-8.10	no	813	Rmet_3492	*0*	Op0970r_2	MET-CAH-But
4856	TSS_885781+3	23	2	-40	CCAGCCGTAG	-15	TTTAATCTTA	-143	5,1	7.15	-1.60	no	316	Rmet_6657	*0*	Op1220f_1	NA
4857	TSS_1162236+3	23	6	-39	TCTGCCCGAT	-15	GCTATCCTTC	-142	5,1	10.00	4.40	m	353	Rmet_6674	*0*	Op1306f_2	NA
4858	TSS_1577486+3	23	10	-42	TCGTAGACGC	-15	GCGATGATGG	-140	1,1	5.74	-1.10	no	4563	Rmet_6695	*0*	Op1440f_1	NA
4859	TSS_1625964+3	23	6	-43	TGCCAATCGG	-14	TTTACAATTT	-142	1,1	8.14	-3.60	no	9806	Rmet_6699	*0*	Op2081f_1	NA
4860	TSS_2511667+3	23	10	-33	TTCATCATCA	-9	CTTACGTTCG	-145	1,1	4.57	5.70	(m)-ba	80	Rmet_5751	*0*	Op1722f_1	NA
4861	TSS_274107-3	23	4	-26	TTGCACCTCC	-2	GGCAAAATTC	-141	1,1	9.43	0.20	no	73	Rmet_3729	*icdA*	Op1041r_3	MET-CAH-TCA
4862	TSS_975129-3	23	5	-41	TACCTGTTAC	-14	GATACCCTTC	-143	1,1	2.35	-2.60	no	3623	Rmet_4372	*0*	Op1247r_1	NA
4863	TSS_1165614-3	23	1	-35	TTGACTTCAG	-13	GTCACGCTGG	-141	1,1	6.62	11.20	s	4817	Rmet_4549	*uvrA2*	Op1305r_1	GIP-REP-Recomb
4864	TSS_1288889-3	23	7	-35	TCGCTACCGA	-14	CGTACGATTC	-139	1,1	6.90	10.10	s	2080	Rmet_4654	*0*	Op1345r_1	NA
4865	TSS_1380160-3	23	8	-36	TCCAGGAACC	-11	CCTCTCATGA	-148	1,1	0.89	5.40	m	1372	Rmet_4734	*garD*	Op1369r_2	MET-CAH-Ascorb
4866	TSS_1728027-3	23	5	-31	TTGCCGCTGC	-10	TCTATGAAGC	-142	1,1	4.66	6.60	s	914	Rmet_5039	*0*	Op1481r_1	MET-CAH-But
4867	TSS_2210768-3	23	8	-34	TTGAAAACCC	-14	TCTACACTCG	-136	1,1	9.72	10.60	s	325	Rmet_5463	*0*	Op1623r_1	NA
4868	TSS_85937-4	23	4	-45	TTGTGCCTCC	-13	GACAGGATCG	-147	1,1	5.60	-8.60	no	92	Rmet_5975	*czcJ*	Op1818r_2	NA
4869	TSS_132451-4	23	2	-39	TTCCAGGTTG	-13	TGTAGTCTTC	-143	1,1	5.76	1.90	w	1232	Rmet_6170	*0*	Op1888r_1	NA
4870	TSS_31703+5	23	6	-37	TTGACTCCGT	-8	GTTACGCTAT	-144	1,1	7.64	4.40	(m)-ba	28	Rmet_6345	*merT*	Op1894f_1	NA
4871	TSS_102327-5	23	7	-41	GCAGCATGCC	-19	TACATGGTTT	-145	5,1	5.04	1.40	(w)-nu	7857	Rmet_6246	*0*	Op1903r_1	NA

4872	TSS_345049+2	22	4	-35	TGCCCAAGGG	-14	GAGATACTAC	-143	1,1	5.56	9.60	s	10800	Rmet_0338	0	Op0095f_1	NA	
4873	TSS_494823+2	22	4	-26	TTGCGCAGGC	-4	CCTCGATTGC	-144	1,1	3.04	0.20	no	808	Rmet_0466	recA	Op0123f_1	GIP-REP-Recomb	
4874	TSS_769832+2	22	7	-44	TCAAAGGTAA	-16	GACAGAATTT	-147	4,1	6.41	-4.60	no	2643	Rmet_0700	dadA2	Op0197f_1	MET-EN-Nitrog	
4875	TSS_917458+2	22	3	-45	TTCTAACGCA	-14	GCTACCATAC	-140	1,1	6.96	-7.10	no	39	Rmet_0838	piuC	Op0239f_2	NA	
4876	TSS_1001159+2	22	5	-38	TCGACGCAAC	-13	GGTAACGTGA	-140	1,1	5.71	5.90	m	929	Rmet_0917	0	Op0263f_4	NA	
4877	TSS_1012380+2	22	6	-42	TGGCGAAAGC	-1	AACAACTTGA	-146	1,1	3.11	-16.10	no	166	Rmet_0927	nuoA	Op0267f_3	MET-EN-OxPhos	
4878	TSS_1075346+2	22	3	-46	TAGATTTAAG	-14	CCTATAGTGG	-149	1,1	4.06	-8.10	no	30	Rmet_0990	0	Op0281f_1	NA	
4879	TSS_1183857+2	22	6	-33	TTGCAGATAA	-13	AGTAGCCTGT	-143	1,1	7.11	6.60	s	3876	Rmet_1092	yflS	Op0315f_1	EIP-TRA-Ion	
4880	TSS_3276551+2	22	8	-40	TTTAGTACCC	-15	TGTACTGTTG	-141	2,1	5.73	2.90	(w)-sba	8075	Rmet_3028	0	Op0849f_1	NA	
4881	TSS_3448125+2	22	3	-37	TTCCCGCCGT	-15	TGCAGCATTC	-138	1,1	5.58	8.90	s	424	Rmet_3176	cbbE2	Op0893f_1	MET-CAH-PP_KDPG	
4882	TSS_3507499+2	22	3	-42	TGGCAATGTC	-14	GTCATCATCG	-141	1,1	7.38	-1.10	no	1136	Rmet_3233	0	Op0917f_1	NA	
4883	TSS_3683317+2	22	3	-44	TTGTTTACGT	-13	ATAAGACTGA	-150	1,1	3.65	-6.60	no	5	Rmet_6595	0	Op0945f_2	NA	
4884	TSS_3713123+2	22	4	-47	GCCAGGCAAC	-22	CGCATTATGC	-140	4,1	7.23	-7.60	no	10	Rmet_3437	pat	Op0955f_1	NA	
4885	TSS_34639-2	22	1	-41	TCGTCCAGAC	-13	GCTAAAGTCG	-141	1,1	6.77	-0.10	no	674	Rmet_0029	0	Op0010r_1		0
4886	TSS_1320400-2	22	5	-23	TGGCTTTGAC	-3	CTTAATTTCA	-144	1,1	3.82	-4.90	no	45	Rmet_1200	phaP	Op0348r_1	NA	
4887	TSS_2165918-2	22	2	-40	TAGCGGTCGG	-14	GCTAGACTTC	-140	2,1	9.22	1.90	(w)-sba	2804	Rmet_1989	dus	Op0582r_1	GIP-TL-Ass	
4888	TSS_2577430-2	22	5	-36	GCAGCAGGGC	-14	GTTACGCTCT	-137	5,1	9.13	6.40	s	2252	Rmet_2363	0	Op0664r_1	NA	
4889	TSS_3428378-2	22	7	-33	TGGTACGGTC	-13	CCTAAAGTGC	-143	1,1	6.12	6.10	m	692	Rmet_R0050	0	Op0886r_1	NA	
4890	TSS_3570513-2	22	4	-49	TTGAAAACGG	-14	GGCAAGATGC	-141	1,1	8.64	-13.60	no	124	Rmet_3295	rpmJ	Op0926r_2	GIP-TL-Ribo	
4891	TSS_3800033-2	22	5	-41	TTGAGCATAT	-13	CGTATAATGG	-144	1,1	11.44	3.40	(m)-sba	1100	Rmet_3508	bug	Op0972r_1	NA	
4892	TSS_153206+3	22	9	-45	TCGCCATTTA	-3	GAGACAGTTT	-146	1,1	5.21	-18.10	no	422	Rmet_R0074	0	Op1006f_1	NA	
4893	TSS_1399269+3	22	3	-36	TTCCACGAGA	-14	TATAGGCTGC	-138	1,1	5.88	8.90	s	3968	Rmet_4756	0	Op1382f_1	NA	
4894	TSS_2097251+3	22	10	-48	TCGGATACCG	-14	CCTACCATCC	-140	5,1	7.42	-14.10	no	87	Rmet_5372	0	Op1600f_1	NA	
4895	TSS_2303982+3	22	6	-46	TAGAGCAATG	-26	AGCACGGTCG	-148	1,1	0.35	-2.70	no	1108	Rmet_5557	0	Op1648f_3	MET-CAH-Prop	
4896	TSS_2365564+3	22	9	-41	TGGATGACCA	-14	GTCATGATGT	-140	1,1	6.29	0.90	(w)-sba	1486	Rmet_5610	cyoC	Op1674f_1	MET-EN-OxPhos	
4897	TSS_729072-3	22	5	-40	TTGCTGAATC	-14	CATACAATCC	-139	1,1	9.57	4.40	(m)-sba	2	Rmet_4153	tnpA	Op1169r_1	NA	
4898	TSS_1392357-3	22	5	-42	TTCCACCAAT	-14	GATAAAGTTG	-142	1,1	7.39	-2.10	no	4035	Rmet_6683	0	Op1375r_1	NA	
4899	TSS_1819431-3	22	4	-44	TCTGCAATGT	-24	TCTACATTCC	-145	5,1	9.20	0.80	w	84	Rmet_5124	0	Op1509r_1	NA	
4900	TSS_2086596-3	22	4	-49	TCGTTTATAC	-13	GGCAAACTTT	-149	1,1	5.23	-18.10	no	35	Rmet_5364	dkgB	Op1595r_1	NA	
4901	TSS_5546-4	22	9	-40	TGCCCAGTCT	-20	AATACGTTGC	-144	1,1	4.35	3.80	m	723	Rmet_6060	repA	Op1846r_1	NA	
4902	TSS_63155-4	22	6	-49	TCGGCAAGGC	-10	ACAAGCATGT	-144	5,1	6.37	-19.10	no	389	Rmet_5996	0	Op1828r_1	NA	
4903	TSS_89441-4	22	5	-37	TGGTAATGTG	-14	CGTACTATCT	-142	1,1	6.67	8.90	s	40	Rmet_6369	0	Op2111r_1	NA	
4904	TSS_110827+5	22	4	-38	TTGTACTTTT	-13	ATTAATATTC	-145	1,1	7.74	6.40	s	4222	Rmet_6261	rhsB	Op1908f_1	NA	
4905	TSS_83831+2	22	2	-35	TCGAACAGCA	-14	TGCAGAATCC	-141	1,1	6.93	10.10	s	5360	Rmet_0082	0	Op0023f_1	NA	
4906	TSS_438299+2	22	6	-40	TTCATTCGCG	-15	GATACAATCA	-139	2,1	11.77	4.90	(m)-sba	41	Rmet_0412	0	Op0113f_2	NA	
4907	TSS_442842+2	22	7	-42	GTGCGGTGCG	-14	CCTAGAATGA	-142	2,1	6.06	-2.60	no	98	Rmet_0417	tyrS	Op0115f_1	GIP-TL-Ass	
4908	TSS_1012631+2	22	4	-23	TGGCACTGAT	-3	CGAATTCTCG	-144	1,1	3.91	-4.90	no	281	Rmet_0928	nuoB	Op0267f_3	MET-EN-OxPhos	
4909	TSS_1126294+2	22	5	-41	TTGTAGTGTT	-14	GGAATTATAG	-144	1,1	5.91	1.40	(w)-sba	161	Rmet_1037	nlpD	Op0297f_1	NA	
4910	TSS_1193076+2	22	3	-43	TGGTCCAATA	-11	TCTATGTTGA	-145	1,1	5.24	-6.10	no	41	Rmet_1096	0	Op0315f_3	NA	
4911	TSS_1242345+2	22	2	-38	CCGGCAGTCC	-14	GGTATGATGG	-134	5,1	11.74	5.90	m	46	Rmet_1136	0	Op0325f_1	NA	
4912	TSS_1792629+2	22	4	-35	TTTACATCGT	-14	CCTACGCTCC	-148	1,1	1.63	11.10	s	3370	Rmet_1661	0	Op0469f_1	NA	
4913	TSS_1819233+2	22	10	-29	TTCACCACGG	-3	ACCACGATAT	-145	1,1	4.98	0.70	(w)-nu	4	Rmet_1682	0	Op0475f_1	NA	
4914	TSS_2379829+2	22	7	-33	GCAACCGACG	-10	AATATCTTTC	-145	4,1	5.20	4.20	m	47	Rmet_R0034	0	Op0625f_1	NA	
4915	TSS_3330140+2	22	3	-35	TTGCGGCGCG	-14	AGTAGATTGG	-138	2,1	10.53	10.60	s	74	Rmet_3075	0	Op0863f_2	NA	
4916	TSS_3643385+2	22	8	-37	TTCAGCGAAT	-15	GGCACAATAA	-140	1,1	7.32	9.90	s	2412	Rmet_3369	paaA	Op0937f_1	NA	
4917	TSS_3878129+2	22	4	-41	GGCGGAGCCC	-16	TGTAGTATTG	-139	4,1	7.65	-1.60	no	66	Rmet_3575	0	Op0993f_2	EIP-SIG-2Comp	
4918	TSS_313896-2	22	5	-34	TTGCGCACCG	-13	CCCAGCATAT	-141	1,1	5.02	8.60	s	4650	Rmet_0292	0	Op0084r_1	NA	
4919	TSS_789219-2	22	6	-37	TGACCACACG	-13	GGTAGCCTTC	-140	4,1	4.86	6.40	s	677	Rmet_6426	0	Op0202r_1	NA	
4920	TSS_1600146-2	22	5	-39	TCCAGAATAG	-19	TCTACAGTGT	-144	1,1	5.83	5.80	(m)-ba	3706	Rmet_1476	0	Op0414r_1	NA	
4921	TSS_2056322-2	22	13	-34	TTGCATGAAG	-9	GTCAAAGTGC	-147	1,1	6.87	6.20	(m)-ba	985	Rmet_1887	trmC	Op0544r_1	NA	
4922	TSS_2366818-2	22	6	-45	TCGATGGGCA	-13	CGTATGCTTT	-144	1,1	5.82	-8.10	no	3745	Rmet_2162	0	Op0622r_1	NA	
4923	TSS_2374607-2	22	9	-33	TTCACCAGAA	-12	TCCATACTGG	-140	1,1	7.32	9.10	s	1614	Rmet_2170	0	Op0624r_2	NA	
4924	TSS_2666415-2	22	1	-46	TTGCGCAAGA	-14	GGTATACCGG	-149	1,1	2.60	-9.60	no	3930	Rmet_2450	0	Op0684r_1	NA	
4925	TSS_2801494-2	22	5	-42	TGGTGCAGAC	-7	GATCAGATAG	-146	1,1	1.54	-11.10	no	2129	Rmet_2574	orn	Op0714r_1	NA	
4926	TSS_3276729-2	22	3	-38	TTGAGCGCTT	-14	GATACGCTCT	-144	1,1	7.62	7.40	s	1277	Rmet_3016	hmzR	Op0848r_4	NA	
4927	TSS_476591+3	22	11	-49	TAGCTGATGG	-13	CGTATGATGC	-143	1,1	5.09	-16.10	no	1587	Rmet_R0079	0	Op1098f_1	NA	
4928	TSS_650853+3	22	3	-49	TTCAGAGAGT	-14	AGTAAACTTG	-143	1,1	8.31	-14.10	no	876	Rmet_4084	phoA1	Op1148f_1	MET-COF-Folate	
4929	TSS_992113+3	22	5	-46	GCAACAACAA	-15	GTGAGAATCT	-143	4,1	8.05	-10.60	no	36	Rmet_4395	uspA9	Op1250f_1	NA	
4930	TSS_1287536+3	22	5	-38	ATTCTCCGCG	-15	CTTATGATGC	-137	2,1	11.32	4.90	m	873	Rmet_4656	0	Op1346f_1	NA	
4931	TSS_1304897+3	22	11	-34	TTCCGGCCGC	-14	ATTACCATCA	-138	1,1	6.07	7.10	s	1057	Rmet_4673	0	Op1352f_1	NA	
4932	TSS_1402787+3	22	7	-42	GTCGGAATCT	-15	GGTGTAATGG	-143	4,1	4.05	-3.10	no	450	Rmet_4756	0	Op1382f_1	NA	

4933	TSS_1676674+3	22	12	-36	CCGGCAATCG	-13	CGGATAATCG	-141	5,1	9.77	8.90	s	8004	Rmet_5004	0	Op1468f_1	NA
4934	TSS_1855759+3	22	7	-41	TGGTAGTAGG	-14	CTCAAAATAT	-145	1,1	5.69	-0.10	no	152	Rmet_5157	0	Op1518f_1	NA
4935	TSS_2479918+3	22	6	-39	TTCCCGGAAT	-14	GTTATGCTGG	-144	1,1	7.99	4.90	m	323	Rmet_5722	0	Op1714f_1	NA
4936	TSS_527885-3	22	6	-43	TTCAGAACAT	-13	TTTACCATGC	-140	1,1	8.10	-3.10	no	5948	Rmet_3961	gabD	Op1111r_1	MET-CAH-But
4937	TSS_808419-3	22	9	-39	GCAAACAAGC	-13	TTTATCATCA	-144	4,1	10.38	0.40	w	5277	Rmet_4218	0	Op1197r_1	NA
4938	TSS_1273624-3	22	4	-36	TCGACACACT	-15	CACACAATCC	-138	1,1	7.10	12.30	s	208	Rmet_4642	0	Op1341r_2	NA
4939	TSS_1665023-3	22	6	-38	TTGCGCTGCT	-14	CTTAAGCTTG	-141	1,1	7.41	6.40	s	3761	Rmet_4985	0	Op1463r_1	EIP-TRA-Ion
4940	TSS_1761760-3	22	2	-38	GCGAAAAGCT	-15	GCTACAATTC	-142	5,1	10.43	6.90	s	837	Rmet_5072	0	Op1493r_1	MET-AA-Met
4941	TSS_2033447-3	22	3	-40	TCGCACAGGA	-13	CGCAGAATGG	-139	1,1	6.93	0.90	(w)-sba	247	Rmet_5322	zniS	Op1573r_1	NA
4942	TSS_2217693-3	22	7	-42	TGGCAAGAAC	-13	AATATCATGA	-142	1,1	7.57	-1.10	no	848	Rmet_5471	fumB	Op1625r_2	MET-CAH-TCA
4943	TSS_32833+4	22	1	-41	TTCTAGCGCG	-15	GGCAGAATTC	-140	1,1	6.70	0.90	(w)-sba	1385	Rmet_6025	0	Op1835f_1	NA
4944	TSS_101604-4	22	4	-38	CCAGCACTCG	-12	GCAAAAATGT	-138	5,1	8.40	1.40	w	738	Rmet_6371	0	Op2112r_1	NA
4945	TSS_153501-4	22	7	-35	TGGAATGCGA	-14	GTTAAGGTTC	-142	1,1	5.76	9.10	s	5549	Rmet_6379	0	Op1879r_1	NA
4946	TSS_218157-4	22	6	-48	TCGGAAATAG	-13	AGTATCCTGA	-144	5,1	8.14	-14.10	no	5668	Rmet_6089	0	Op1854r_1	NA
4947	TSS_18778+5	22	7	-35	TCGGCAACGG	-15	GGCAAAATCG	-139	5,1	10.26	10.10	s	620	Rmet_6330	0	Op1924f_1	NA
4948	TSS_91276-5	22	12	-47	TCACGACTAT	-13	GATATAGTCA	-146	4,1	7.23	-12.60	no	726	Rmet_6241	parB2	Op1901r_1	NA
4949	TSS_170852-5	22	7	-38	TTGATGGCAC	-9	CACATACTTG	-141	1,1	6.88	3.40	(m)-ba	13493	Rmet_6301	trbF	Op1917r_1	NA
4950	TSS_474786+2	22	7	-49	TCGCCCATCA	-14	GGCAAAATGG	-139	1,1	8.09	-15.10	no	9431	Rmet_0456	rhlE1	Op0119f_1	NA
4951	TSS_524147+2	22	3	-36	TTGTGGTCTG	-16	AATATAGTAT	-144	1,1	6.20	10.80	s	45	Rmet_0495	0	Op0133f_1	MET-AA-Phe
4952	TSS_1363794+2	22	4	-30	TTCCACGCTT	-3	GGTACACAAG	-146	1,1	2.88	-2.30	no	44	Rmet_1240	ritB2	Op0357f_2	NA
4953	TSS_1627296+2	22	3	-37	TGGCAGCTTT	-14	GATATAAAGC	-145	1,1	5.17	7.90	s	3684	Rmet_1509	cbbS(del	Op0425f_1	NA
4954	TSS_2028561+2	22	6	-36	TCGCACATCG	-15	GGCAGACTGT	-139	1,1	6.26	8.30	s	1783	Rmet_6481	0	Op2018f_1	NA
4955	TSS_2145019+2	22	8	-38	TGGACTTGCA	-14	CCTACAATAT	-136	1,1	8.39	8.90	s	253	Rmet_1969	bcp	Op0575f_2	GIP-PTL-Fold
4956	TSS_2981428+2	22	4	-45	TTGCGAGGAT	-14	TGCATCATTG	-140	1,1	8.08	-5.60	no	5548	Rmet_2750	ppc	Op0775f_1	MET-CAH-Pyr
4957	TSS_3638420+2	22	6	-46	TTGACGCATG	-15	GCTAGAATTT	-139	1,1	10.60	-4.60	no	7377	Rmet_3369	paaA	Op0937f_1	NA
4958	TSS_794116-2	22	6	-40	TTGTGCGGAT	-13	CGGATAATGT	-140	1,1	7.74	3.40	(m)-sba	5574	Rmet_6426	0	Op0202r_1	NA
4959	TSS_829005-2	22	2	-36	TGGCACCTTC	-13	AGTACGGTAT	-142	1,1	4.52	7.90	s	3653	Rmet_0747	0	Op0212r_1	NA
4960	TSS_1061440-2	22	4	-34	TCGCACAACG	-13	GTGATGATGT	-139	1,1	6.09	8.10	s	317	Rmet_0974	paaH	Op0272r_1	MET-CAH-But
4961	TSS_2487858-2	22	7	-34	TTCCCGAGCG	-13	ACTAAGCTTA	-143	1,1	5.71	8.10	s	419	Rmet_2267	fliD1	Op0648r_3	DIV-MOT-Flagel
4962	TSS_2772714-2	22	4	-36	TCGAGGTTTG	-13	GTTAAAGTCG	-142	1,1	7.03	9.90	s	1313	Rmet_2546	0	Op0706r_1	NA
4963	TSS_2908892-2	22	2	-46	TCGATACGGA	-14	CGTAGCATTT	-140	1,1	6.38	-8.10	no	409	Rmet_2674	pal	Op0756r_4	NA
4964	TSS_2945037-2	22	4	-38	GTCGGAGATC	-13	GGTAACATCG	-143	4,1	6.69	2.90	w	4385	Rmet_2707	0	Op0766r_1	NA
4965	TSS_3090997-2	22	4	-48	CCGGCACCCC	-12	CGTAGAATCT	-136	5,1	11.22	-16.10	no	481	Rmet_2842	tnpA	Op2044r_1	NA
4966	TSS_3211455-2	22	7	-40	TCCAGCGCAA	-13	TCTACAATCC	-142	1,1	7.18	1.40	(w)-sba	1240	Rmet_2951	phcS	Op0832r_3	NA
4967	TSS_3263746-2	22	3	-45	TTGACGACCA	-24	TGCACACTGT	-144	1,1	7.73	2.80	w	18	Rmet_3007	tniR	Op0846r_1	NA
4968	TSS_3328900-2	22	7	-37	TTGCCCAGTG	-6	CGAATGATTG	-142	1,1	6.46	2.40	(w)-ba	383	Rmet_3071	0	Op0862r_3	NA
4969	TSS_3366147-2	22	5	-41	TCCATGATGC	-14	ACCATCATCT	-143	1,1	3.95	-0.60	no	826	Rmet_3106	rplU	Op0874r_1	GIP-TL-Ribo
4970	TSS_3537771-2	22	3	-48	TGGCCGCGAC	-14	CGTAAGCTCT	-141	1,1	6.18	-14.10	no	4335	Rmet_3262	gltD	Op0920r_3	MET-EN-Nitrog
4971	TSS_3614285-2	22	8	-40	GCAAACCGAC	-14	AGTATTCTAA	-147	5,1	4.63	-1.60	no	1366	Rmet_R0060	0	Op0930r_1	NA
4972	TSS_3820445-2	22	5	-38	TTCCGGCGAC	-13	GCGACAATGG	-138	1,1	7.48	4.90	m	27	Rmet_3527	0	Op0976r_2	MET-AA-ArgPro
4973	TSS_151481+3	22	3	-39	TCCGAAAACT	-9	CTCACCTTCG	-149	4,1	3.39	-3.60	no	#NV	#NV	#NV	#NV	#NV
4974	TSS_204403+3	22	3	-40	GGACGAGCGC	-14	TGTACGATGG	-138	4,1	10.14	-1.60	no	257	Rmet_3666	0	Op1020f_4	NA
4975	TSS_527928+3	22	3	-35	TGGTAAATCC	-14	CATACCATGT	-141	1,1	6.66	10.10	s	10521	Rmet_3974	0	Op1114f_1	NA
4976	TSS_1908779+3	22	7	-48	TCGATCAGTA	-14	GGTACAGTTA	-141	1,1	6.82	-13.10	no	3931	Rmet_5218	0	Op1538f_1	NA
4977	TSS_1915115+3	22	7	-35	TTGCTTGCGG	-14	CTTAACCTTC	-146	1,1	6.21	9.60	s	1004	Rmet_5222	hipO	Op1540f_1	MET-AA-Phe
4978	TSS_2086650+3	22	2	-44	TTGCCGTCCT	-14	TGTATAAACG	-148	1,1	6.01	-3.60	no	20	Rmet_5365	0	Op1596f_1	NA
4979	TSS_2201422+3	22	6	-42	TCGTCCAGAC	-14	GCTAAAGTCG	-141	1,1	6.77	-1.10	no	6365	Rmet_6726	0	Op2092f_1	NA
4980	TSS_2456081+3	22	4	-34	TCGAGAGAGA	-13	GGGAGAATCT	-140	1,1	6.93	10.10	s	645	Rmet_5698	0	Op1704f_1	NA
4981	TSS_118249-3	22	1	-40	TGGCGCAAGC	-14	GGCAAAATAC	-139	1,1	7.88	1.90	(w)-sba	1097	Rmet_5915	0	Op1795r_1	NA
4982	TSS_1022670-3	22	1	-35	TTGCAAAAGT	-13	ATTACTATGT	-142	1,1	9.08	12.20	s	38	Rmet_4427	0	Op1259r_1	MET-CAH-But
4983	TSS_1869107-3	22	3	-28	TTCTACATGG	-8	TGTAAAGTGG	-145	1,1	6.59	1.10	(w)-nu	5207	Rmet_6711	0	Op2086r_1	NA
4984	TSS_1922349-3	22	4	-33	TGGAGCATGA	-13	TGTAATGTCT	-143	1,1	5.11	6.10	m	6	Rmet_5226	0	Op1541r_2	NA
4985	TSS_74891+4	22	5	-37	TTGCCTGATG	-17	CGCATACACG	-144	1,1	2.23	7.80	s	284	Rmet_5983	czcI	Op1820f_1	NA
4986	TSS_7452-5	22	8	-35	TGACAAATGG	-13	GCTATACTGC	-145	4,1	9.70	10.20	s	85	Rmet_6320	int	Op1919r_1	NA
4987	TSS_12515-5	22	3	-24	TCGCAGGTGA	-2	ACCACACTGT	-145	1,1	5.62	-2.30	no	5148	Rmet_6320	int	Op1919r_1	NA
4988	TSS_62559-5	22	2	-41	TGCAACGTAT	-13	GCGAAAATCC	-141	1,1	6.53	-1.60	no	10413	Rmet_6203	chrB1	Op1895r_1	NA
4989	TSS_319408+2	21	3	-47	GCACGGCCAA	-14	TCGATAATGC	-143	4,1	7.32	-15.60	no	4054	Rmet_0308	0	Op0087f_1	EIP-TRA-Ion
4990	TSS_797763+2	21	6	-43	TGGCAGACCC	-15	CCTATGATGA	-139	1,1	7.66	-2.10	no	539	Rmet_0721	cmk	Op0205f_1	MET-NUC-Pyr
4991	TSS_1105584+2	21	3	-36	TTTCGTCGCG	-15	GCGATGATGC	-136	2,1	13.23	9.30	s	77	Rmet_1016	0	Op0293f_1	NA
4992	TSS_1775546+2	21	7	-38	TTCTGTATGC	-15	TTTAATATCG	-145	1,1	6.05	6.90	s	4957	Rmet_1646	0	Op0463f_1	NA
4993	TSS_1844306+2	21	5	-40	TGGCCATGTC	-14	GTCAATATGC	-140	1,1	7.02	2.90	(w)-sba	1994	Rmet_1703	acrC	Op0481f_2	NA

4994	TSS_1889760+2	21	4	-44	ATTCGTTTCG	-14	CGGAGAATCC	-141	2,1	10.56	-9.10	no	154	Rmet_1743	0	Op0495f_1	NA	
4995	TSS_2342003+2	21	2	-34	TTGAATTATA	-13	CATCCAATGA	-147	1,1	4.64	9.60	s	30	Rmet_2149	0	Op0621f_1	NA	
4996	TSS_2744260+2	21	4	-36	TTGTCACCTG	-14	AATATTCTTG	-143	1,1	6.46	13.40	s	8945	Rmet_2531	ivd1	Op0699f_1	NA	
4997	TSS_3135011+2	21	6	-37	TTCCGGAGCG	-14	GTTATGGTTC	-142	2,1	9.51	7.90	s	669	Rmet_2883	0	Op0811f_1	GIP-REP-Recomb	
4998	TSS_3634697+2	21	7	-47	CCAGAAGCCC	-14	GGGAGAATTT	-141	5,1	8.46	-15.60	no	942	Rmet_3360	0	Op0935f_3	NA	
4999	TSS_3710427+2	21	5	-40	TCGCGCACGC	-14	GATACGATGA	-137	1,1	7.34	1.90	(w)-sba	355	Rmet_3433	ycbL	Op0953f_1	MET-CAH-Pyr	
5000	TSS_3814044+2	21	3	-34	TCGAACGTCT	-14	GCGATCCTAT	-142	1,1	4.41	7.10	s	3925	Rmet_3525	cupC	Op0975f_1	NA	
5001	TSS_35336-2	21	7	-38	GCCAACAACC	-13	TGGACAATGG	-140	4,1	8.71	1.40	w	1371	Rmet_0029	0	Op0010r_1		0
5002	TSS_303678-2	21	2	-35	TTTTTTTTCG	-14	CGTACGCTTT	-140	2,1	10.37	8.10	s	64	Rmet_0287	pth	Op0084r_4	GIP-TL-Ass	
5003	TSS_506221-2	21	7	-43	TGGCCTCGGT	-21	GACAAGATGC	-141	1,1	6.50	2.90	w	23	Rmet_0476	0	Op0124r_1	NA	
5004	TSS_949107-2	21	5	-45	CGCAACCAGA	-13	GTTACGATAC	-146	1,1	1.84	-12.60	no	1665	Rmet_0867	0	Op0250r_1	NA	
5005	TSS_2237809-2	21	6	-34	TTGCTGTGGG	-13	GTCATCACTT	-138	1,1	7.51	9.60	s	222	Rmet_2064	0	Op0600r_2	NA	
5006	TSS_2964956-2	21	2	-45	TCTGACATGC	-19	TTTATGCTAA	-145	5,1	6.97	-4.60	no	174	Rmet_2728	0	Op0770r_1	NA	
5007	TSS_2971553-2	21	5	-40	TAGAATACCG	-14	TTAAAAATAA	-148	1,1	1.69	2.90	(w)-sba	78	Rmet_2735	rfbB	Op0770r_1	MET-CAH-NucSug	
5008	TSS_3042116-2	21	5	-37	TGGAGAGTAA	-12	GCTATCATTA	-136	1,1	8.58	8.90	s	87	Rmet_2794	matB	Op0786r_3	NA	
5009	TSS_3149598-2	21	9	-38	TACAAGGTGG	-13	ACTACACTCC	-143	1,1	3.60	4.40	m	197	Rmet_2892	clpA	Op0812r_2	GIP-PTL-Fold	
5010	TSS_3192163-2	21	9	-35	TTGTTGTGCA	-14	GTCATTCTTC	-140	1,1	5.48	9.60	s	90	Rmet_2936	pilT	Op0828r_1	GIP-PTL-T2S	
5011	TSS_3615067-2	21	8	-38	TCCGAAAACT	-8	CTCACCTTCG	-149	4,1	3.39	-2.60	no	2148	Rmet_R0060	0	Op0930r_1	NA	
5012	TSS_475221+3	21	4	-23	GCATGTGGTG	-3	CGATTACAGG	-149	3,0	2.35	-12.40	no	#NV	#NV	#NV	#NV	#NV	
5013	TSS_478202+3	21	8	-39	CCGGCAACCC	-19	GTGACCATAG	-141	5,1	7.82	3.30	(m)-ba	444	Rmet_6634	0	Op1098f_2	NA	
5014	TSS_1715042+3	21	2	-49	TTGCCGTCGT	-14	GGCATACTGC	-137	1,1	9.61	-13.60	no	220	Rmet_6705	0	Op1480f_1	NA	
5015	TSS_1723222+3	21	6	-37	TTGACTTCGA	-15	TGCACGCTGG	-140	1,1	6.37	10.40	s	3908	Rmet_5040	0	Op1482f_1	NA	
5016	TSS_322751-3	21	8	-39	TTCACCACTA	-15	GTGATACATC	-148	1,1	1.67	4.90	m	10965	Rmet_3775	0	Op1049r_1	NA	
5017	TSS_1188645-3	21	1	-34	GCCGAAATGT	-14	TCCACAATTG	-139	4,1	9.27	3.60	m	753	Rmet_4573	0	Op1315r_1	NA	
5018	TSS_1594166-3	21	9	-42	TGGCCGGATG	-13	TAAATCATAT	-146	1,1	3.85	-2.10	no	56	Rmet_4930	0	Op1445r_1	NA	
5019	TSS_1952223-3	21	6	-33	TGGCTGCCTG	-13	CGTAGAGTGC	-142	1,1	5.65	6.10	m	1368	Rmet_5250	0	Op1549r_1	DIV-MOT-Chemotax	
5020	TSS_2473031-3	21	3	-49	TTCATCAGTA	-13	CGCATCATGG	-145	1,1	5.88	-16.10	no	78	Rmet_5713	0	Op1709r_2	NA	
5021	TSS_219167+4	21	2	-48	GCCAAGACAT	-15	ATTACCATCC	-141	4,1	9.02	-16.60	no	178	Rmet_6082	0	Op1853f_2	NA	
5022	TSS_4399+5	21	6	-36	TCGGCAATAG	-14	GGTACGCTGG	-140	5,1	9.81	10.90	s	266	Rmet_6318	0	Op1918f_5	NA	
5023	TSS_132548-5	21	5	-44	TCGATGCCGG	-15	GCTATCATTC	-139	1,1	7.81	-3.10	no	13	Rmet_6400	0	Op2117r_1	NA	
5024	TSS_315793+2	21	3	-40	TTGCCCTTGT	-13	GACATCTTTT	-143	1,1	6.32	2.40	(w)-sba	7669	Rmet_0308	0	Op0087f_1	EIP-TRA-Ion	
5025	TSS_761995+2	21	5	-36	TTCTCGTGAT	-15	GCGATGATGC	-139	1,1	6.53	10.30	s	390	Rmet_0690	0	Op0195f_1	EIP-TRA-ABC	
5026	TSS_955782+2	21	8	-47	GCTGCTCTAC	-14	CTTAAACTTG	-144	5,1	8.12	-15.60	no	4316	Rmet_0877	0	Op0255f_1	NA	
5027	TSS_1282322+2	21	4	-41	TTGCTGAATC	-15	CATACAATCC	-139	1,1	9.57	3.40	(m)-sba	1	Rmet_1171	tnpA	Op0335f_1	NA	
5028	TSS_1315309+2	21	4	-44	GCGGCAGCGT	-15	AGTAACCTCG	-138	5,1	8.55	-7.10	no	58	Rmet_1197	aceF	Op0347f_2	MET-CAH-FbP	
5029	TSS_1695875+2	21	9	-47	CCGTCAAGGT	-15	CGCATGATCT	-142	5,1	7.10	-12.10	no	1228	Rmet_1577	betB	Op0435f_1	MET-AA-GlySerThr	
5030	TSS_1793615+2	21	2	-36	TCGGCCACGC	-13	GTCACGCTTA	-144	5,1	6.84	7.90	s	2384	Rmet_1661	0	Op0469f_1	NA	
5031	TSS_2358384+2	21	2	-49	TTGCCCTCGG	-14	GCTCTAATAC	-143	1,1	6.36	-13.60	no	318	Rmet_6510	0	Op0621f_2	NA	
5032	TSS_2782247+2	21	2	-44	TCCTAGTGTG	-15	CCTATTATGG	-143	1,1	5.41	-5.60	no	3984	Rmet_2562	0	Op0709f_1	NA	
5033	TSS_2848421+2	21	6	-33	GCGGCACGCT	-8	TTCAGGCTGG	-138	5,1	7.35	1.70	(w)-ba	485	Rmet_2618	0	Op0733f_1	NA	
5034	TSS_2872348+2	21	4	-34	TTCACGGAAC	-13	GCCAAAATAG	-138	1,1	7.71	10.10	s	370	Rmet_2636	0	Op0741f_1	GIP-PTL-Fold	
5035	TSS_2896489+2	21	6	-37	TTCCCGTTGC	-15	CATATCCTAA	-139	1,1	5.80	9.90	s	3229	Rmet_2665	0	Op0755f_1	NA	
5036	TSS_3615326+2	21	7	-36	TCGCTACGCC	-15	GTTAAGCTTG	-142	1,1	6.32	9.30	s	2690	Rmet_6590	0	Op0931f_1	NA	
5037	TSS_138411-2	21	6	-45	TCTGCTCGGT	-13	CCCATGATGT	-141	5,1	6.94	-9.60	no	3569	Rmet_0128	zur	Op0032r_1	NA	
5038	TSS_689426-2	21	6	-33	GTACGGTAAT	-13	GTCATGCTTC	-150	4,1	0.62	2.10	w	2946	Rmet_0625	0	Op0178r_1	NA	
5039	TSS_1579046-2	21	7	-44	TTGAAGACCT	-20	GCGAACGTCA	-142	1,1	4.53	0.40	w	391	Rmet_1456	smpB	Op0406r_1	GIP-PTL-Fold	
5040	TSS_1871776-2	21	3	-36	CCAGCACGCC	-13	GGCATGATGT	-140	5,1	9.52	6.40	s	8406	Rmet_1720	0	Op0486r_1	NA	
5041	TSS_2159446-2	21	7	-22	TTGTTGGTGA	-2	CACACCCTCC	-144	1,1	3.78	-5.40	no	2849	Rmet_1983	0	Op0578r_1	NA	
5042	TSS_2405721-2	21	4	-30	TGCCGATTCT	-3	AGGAACATAC	-145	1,1	3.59	-1.80	no	890	Rmet_2194	yeaS	Op0630r_3	NA	
5043	TSS_2891776-2	21	5	-37	CCTGCCAGCG	-14	AATACGATGG	-138	5,1	9.66	4.40	m	838	Rmet_6546	0	Op0752r_1	NA	
5044	TSS_3320453-2	21	6	-34	TGACGGAACG	-13	TGCACATTGG	-144	4,1	4.40	5.60	m	1947	Rmet_3060	mpl	Op0860r_1	NA	
5045	TSS_3414741-2	21	6	-49	TTCCCGTCGG	-13	GAGACAATGG	-139	1,1	7.48	-16.10	no	453	Rmet_3150	0	Op0882r_2	NA	
5046	TSS_3506200-2	21	1	-35	TGCACATGTT	-14	TGTACGGTTC	-140	1,1	5.52	9.60	s	1062	Rmet_3228	petC	Op0914r_1	MET-EN-OxPhos	
5047	TSS_3556315-2	21	6	-49	TCGATTTACG	-17	TTTACTATCT	-147	1,1	6.01	-12.10	no	119	Rmet_3281	0	Op0924r_2	NA	
5048	TSS_3571720-2	21	6	-42	TCGGCATCAT	-13	ATCATCATGC	-142	5,1	7.33	-1.10	no	1081	Rmet_3296	infA	Op0926r_2	NA	
5049	TSS_3591069-2	21	4	-34	TTCTGCCTGA	-14	GGTAACGTTG	-145	1,1	4.87	6.10	m	520	Rmet_3325	fusA1	Op0928r_2	NA	
5050	TSS_3615319-2	21	5	-33	TTGTATAATG	-2	GGCAAGCTTA	-145	1,1	5.32	-1.80	no	#NV	#NV	#NV	#NV	#NV	
5051	TSS_3616802-2	21	4	-34	TTGCTACGCA	-13	GAGAGACTGC	-141	1,1	6.61	9.60	s	440	Rmet_R0063	0	Op0930r_1	NA	
5052	TSS_3618053-2	21	5	-29	TTCAAAAGTT	-3	TAAAACATGT	-145	1,1	4.85	0.70	(w)-nu	100	Rmet_R0064	0	Op0930r_1	NA	
5053	TSS_3718710-2	21	4	-48	TGCAATTTTT	-27	GCTAACATGA	-145	1,1	6.89	-3.20	no	86	Rmet_3443	0	Op0958r_1	MET-NUC-Pur	
5054	TSS_3883810-2	21	3	-34	TCCAAACATC	-14	GTCAGAATGT	-142	1,1	6.28	7.60	s	1559	Rmet_3577	pckG	Op0994r_1	MET-CAH-TCA	

5055	TSS_147216+3	21	5	-42	TCGCGCGCCG	-15	GCAATACTCT	-142	1,1	4.84	-1.10	no	1076	Rmet_6615	0	Op1006f_1	NA	
5056	TSS_152603+3	21	7	-34	GCACGAATGG	-14	GCCACACTGT	-142	4,1	8.40	2.60	w	1025	Rmet_R0074	0	Op1006f_1	NA	
5057	TSS_631141+3	21	4	-39	CTGAAGATGG	-14	GGTACCTTCG	-145	1,1	3.12	2.40	w	7996	Rmet_4071	0	Op1144f_1	NA	
5058	TSS_803234+3	21	2	-42	CGGACCACGG	-14	ATTATACTGA	-147	1,1	3.40	-2.10	no	21	Rmet_4219	0	Op1198f_1	NA	
5059	TSS_2161623+3	21	5	-43	CCTGAACGCC	-14	CGTATTATCC	-139	5,1	9.82	-6.60	no	1207	Rmet_5417	0	Op1612f_2	NA	
5060	TSS_2269237+3	21	4	-48	TTGCAGCAGG	-14	CTCATACTTG	-141	1,1	7.64	-12.60	no	2341	Rmet_5525	0	Op1642f_1	NA	
5061	TSS_145837-3	21	5	-35	TCCACCCCGC	-13	GGTACTTTCC	-140	1,1	4.52	9.20	s	379	Rmet_5939	0	Op1805r_1	NA	
5062	TSS_176283-3	21	7	-48	TCGAGGATTC	-13	CCTACATTAC	-145	1,1	6.26	-14.10	no	50	Rmet_3641	0	Op1015r_1	NA	
5063	TSS_1597341-3	21	10	-29	TTCAAGCGTC	-9	CCTTCAGTTC	-145	1,1	0.67	1.10	(w)-nu	3231	Rmet_4930	0	Op1445r_1	NA	
5064	TSS_1761808-3	21	7	-46	CCTGCCGGGT	-14	TCTATGATAA	-142	5,1	9.79	-12.60	no	885	Rmet_5072	0	Op1493r_1	MET-AA-Met	
5065	TSS_2234041-3	21	8	-34	AAATTCGATG	-14	GGTATCCTTC	-144	3,1	11.71	2.60	w	1520	Rmet_5488	0	Op1629r_1	GIP-REP-Recomb	
5066	TSS_2484324-3	21	5	-33	CCGGAACTGC	-7	TAGATCCTGA	-145	5,1	5.63	0.70	(w)-ba	4654	Rmet_5721	0	Op1713r_1	NA	
5067	TSS_12048+4	21	3	-42	TTGCACTTTC	-14	TTGACAATGC	-141	1,1	8.62	-0.60	no	4372	Rmet_6049	0	Op1843f_1	NA	
5068	TSS_111789+4	21	2	-38	TAGCGCTCCT	-15	CACATGATCC	-142	1,1	2.69	6.90	s	2793	Rmet_5946	*pbrR*	Op1807f_1	NA	
5069	TSS_182294+4	21	6	-36	TTGCATCGAT	-14	GCTAGTGTTG	-140	1,1	6.59	10.40	s	101	Rmet_6119	*copF*	Op1867f_1	MET-EN-P_ATPase	
5070	TSS_224367+4	21	5	-40	TGGCGGCGTG	-16	GCCAAAGTCC	-142	1,1	5.34	2.90	(w)-sba	223	Rmet_6077	0	Op1851f_1	NA	
5071	TSS_96216-4	21	4	-35	TGACAACTGC	-14	TTTACTATGC	-140	4,1	7.71	8.60	s	424	Rmet_5966	*gtrM1*	Op1816r_1	NA	
5072	TSS_225698-4	21	8	-33	TTCCCGGCAG	-13	ATGAAAATGA	-142	1,1	5.76	7.10	s	1652	Rmet_6078	0	Op1852r_1	NA	
5073	TSS_13908+5	21	8	-36	TCCCGCAAGT	-15	TGTATCTTGG	-145	1,1	4.93	7.80	s	2622	Rmet_6327	0	Op1922f_1	NA	
5074	TSS_45075-5	21	6	-37	TCGTAGCCCG	-13	AGTATCCTGA	-143	1,1	4.71	6.90	s	13361	Rmet_6344	*merR*	Op1929r_1	NA	
5075	TSS_302118+2	21	4	-35	TGGTCGTCGC	-15	GGTACATTCG	-138	1,1	7.37	9.10	s	515	Rmet_0286	*fdx*	Op0083f_3	NA	
5076	TSS_630630+2	21	2	-37	TCGCATCTCT	-16	ACTAGTATGG	-143	1,1	6.12	8.30	s	8824	Rmet_0592	0	Op0167f_1	NA	
5077	TSS_1074794+2	21	4	-36	ATCAGGCGGT	-15	TGCAGAATGC	-143	2,1	5.02	7.30	s	582	Rmet_0990	0	Op0281f_1	NA	
5078	TSS_1324603+2	21	5	-44	TTGCCTCGGC	-15	TGTAGGCTCG	-139	1,1	7.33	-3.60	no	1004	Rmet_1206	*ykgE*	Op0351f_1	NA	
5079	TSS_1399665+2	21	6	-41	GTAACGCTTT	-14	CGTAGGATGC	-146	4,1	4.28	-1.10	no	336	Rmet_1280	*tnpA*	Op0369f_1	NA	
5080	TSS_2155225+2	21	5	-45	TTGCACTTCA	-25	TATAAAATCA	-141	1,1	9.61	1.80	w	32	Rmet_1981	*lexA*	Op0577f_1	GIP-TK-Assoc	
5081	TSS_2161773+2	21	7	-49	CCTGAAGGCG	-14	CGCACACTAT	-143	5,1	7.62	-20.60	no	1426	Rmet_1990	0	Op0583f_1	NA	
5082	TSS_2365977+2	21	6	-36	TTGATGTGGC	-15	ACTACGGTGG	-143	1,1	6.56	10.80	s	1834	Rmet_2165	0	Op0623f_1	NA	
5083	TSS_2384457+2	21	2	-38	GCATCATGAC	-14	ATTAAACTGA	-148	5,1	6.46	3.40	m	43	Rmet_2178	*ppk*	Op0627f_1	MET-EN-OxPhos	
5084	TSS_2558480+2	21	4	-42	GCCAGGACGT	-15	GGTAATGTCA	-141	4,1	6.88	-5.60	no	923	Rmet_2345	0	Op0663f_1	NA	
5085	TSS_2819213+2	21	4	-41	GCAGACAAGG	-14	GCCATAATCG	-139	4,1	9.97	-3.60	no	29	Rmet_2593	0	Op0725f_1	NA	
5086	TSS_2890501+2	21	5	-41	TTTCGTTGGC	-3	GACACCTTGC	-143	2,1	9.09	-13.10	no	657	Rmet_2655	*elaB*	Op0753f_1	NA	
5087	TSS_3456508+2	21	6	-25	TGGCCCGCTT	-3	ATCAGCATAC	-144	1,1	4.28	-0.30	no	161	Rmet_3185	0	Op0895f_1	EIP-TRA-ABC	
5088	TSS_3625962+2	21	2	-42	TCGTCCAGAC	-14	GCTAAAGTCG	-141	1,1	6.77	-1.10	no	2436	Rmet_3352	0	Op0933f_1	NA	
5089	TSS_3637881+2	21	3	-43	TTGAGGTGAC	-14	CCGACAATAC	-141	1,1	7.74	-1.60	no	7916	Rmet_3369	*paaA*	Op0937f_1	NA	
5090	TSS_5080-2	21	3	-44	GCGGATGTGC	-13	GTCATGATGA	-143	5,1	6.90	-10.10	no	5390	Rmet_3615	0	Op1004r_1	GIP-TL-Ass	
5091	TSS_120225-2	21	9	-25	GCGGCCGGAT	-2	GGTAGAAAGG	-141	5,1	7.43	-4.30	no	11313	Rmet_0101	0	Op0030r_1		0
5092	TSS_1327731-2	21	5	-42	TGGCGCTCAT	-13	TGTAAAGTTC	-140	1,1	7.27	-3.10	no	2364	Rmet_1205	0	Op0350r_1	NA	
5093	TSS_2072602-2	21	2	-32	TGGCGACCTG	-9	AGAAGAATTC	-144	1,1	5.24	6.70	(s)-ba	1251	Rmet_1901	0	Op0554r_1	NA	
5094	TSS_2629062-2	21	8	-35	TTGCCGGGCG	-14	GGCATCCTGA	-141	1,1	6.76	10.60	s	299	Rmet_2419	0	Op0680r_4	NA	
5095	TSS_2959552-2	21	6	-47	TTGCTAGCAC	-13	CGTATTGTTC	-143	1,1	6.60	-10.60	no	2563	Rmet_6553	0	Op2041r_1	NA	
5096	TSS_3046850-2	21	7	-42	TACCGCCGCC	-13	GGCATATTGG	-141	2,1	8.76	-4.60	no	212	Rmet_2799	0	Op0786r_1	NA	
5097	TSS_3073199-2	21	6	-37	TTCTACTTCT	-15	ACGAAAATCC	-144	1,1	5.89	8.90	s	2309	Rmet_2822	0	Op0794r_1	NA	
5098	TSS_3253534-2	21	7	-37	TTCATATTGA	-13	AACAAACTGC	-146	1,1	5.87	7.90	s	92	Rmet_2995	*ptxA*	Op0842r_1	EIP-TRA-ABC	
5099	TSS_3509001-2	21	6	-47	GGCGCCATCC	-22	GTTAGAATGT	-141	4,1	8.47	-6.60	no	386	Rmet_3232	0	Op0916r_1	NA	
5100	TSS_3612126-2	21	8	-40	TTGCTACCGA	-7	GGAAAAGTTC	-143	1,1	5.57	-3.60	no	1259	Rmet_R0056	0	Op0930r_4	NA	
5101	TSS_3613611-2	21	2	-33	TGGTACGGTC	-13	CCTAAAGTGC	-143	1,1	6.12	6.10	m	692	Rmet_R0060	0	Op0930r_1	NA	
5102	TSS_3731766-2	21	6	-35	TTTCTCCTGG	-14	ACTAACCTTC	-141	2,1	9.31	8.10	s	86	Rmet_3454	0	Op0962r_3	NA	
5103	TSS_225285+3	21	9	-37	TTCACTTACA	-15	TGTAACGTTT	-147	1,1	5.27	9.90	s	141	Rmet_3685	*flhD2*	Op1026f_3	EIP-SIG-2Comp	
5104	TSS_473083+3	21	3	-32	TGGTTGGGTT	-6	GTCAGTTTTC	-148	1,1	2.37	0.70	(w)-ba	61	Rmet_R0075	0	Op1098f_1	NA	
5105	TSS_947762+3	21	2	-40	TGGCATCCCC	-15	GCTACCATGT	-138	1,1	7.66	2.90	(w)-sba	3394	Rmet_4356	*panE*	Op1242f_1	MET-COF-Pant	
5106	TSS_1591246+3	21	6	-24	TGGAACTACT	0	GTCACGATCG	-140	1,1	6.80	-3.30	no	1302	Rmet_4929	*gst*	Op1444f_3	MET-OAA-GSH	
5107	TSS_1691487+3	21	6	-42	TACCACAACA	-15	TACATCATCT	-146	1,1	1.41	-2.60	no	331	Rmet_5013	0	Op1474f_1	NA	
5108	TSS_2318848+3	21	3	-48	TTGCCTGAAC	-14	CGCAGAATTC	-140	1,1	8.18	-11.60	no	276	Rmet_5565	0	Op1654f_1	NA	
5109	TSS_683891-3	21	5	-34	TGGCATTCTC	-14	TTCAGAATCG	-140	1,1	6.56	7.10	s	1944	Rmet_4109	*bug*	Op1157r_1	NA	
5110	TSS_1973371-3	21	4	-37	TAGTCCGCGC	-13	GCGATGATCT	-148	1,1	2.55	7.90	s	3709	Rmet_5270	0	Op1553r_1	NA	
5111	TSS_2326736-3	21	6	-46	TTGAAGACTG	-8	ACCATACTCA	-144	1,1	7.17	-13.60	no	8	Rmet_5572	0	Op1657r_2	NA	
5112	TSS_2373712-3	21	4	-48	TTCTCTCAAA	-13	GGTACTAAGG	-150	1,1	1.86	-16.10	no	5	Rmet_5616	*bug*	Op1675r_1	NA	
5113	TSS_2376445-3	21	4	-38	TGGCGTCTTG	-13	AGCACACTGA	-142	1,1	5.11	3.90	m	403	Rmet_5618	0	Op1677r_1	NA	
5114	TSS_112599-4	21	3	-35	TCGCAGTGGC	-14	AGGAGAATAG	-141	1,1	5.80	9.10	s	499	Rmet_5948	*pbrB/C*	Op1808r_1	GIP-PTL-Exp	
5115	TSS_191580-4	21	3	-33	TTCCCTGACG	-12	TGCAGAATAT	-140	1,1	5.95	8.10	s	381	Rmet_6112	*copA1*	Op1866r_1	NA	

5116	TSS_111071-5	21	3	-35	TTGAAAAAGC	-14	GCAAAACTAC	-143	1,1	7.30	11.60	s	970	Rmet_6257	0	Op1905r_1	NA
5117	TSS_223267+2	20	7	-39	GCTGACATCC	-14	GCCAGAATAC	-140	5,1	8.59	-0.60	no	1135	Rmet_0211	0	Op0063f_3	EIP-TRA-ABC
5118	TSS_351879+2	20	6	-35	TTGACTGACG	-14	ATGATTGTGC	-145	1,1	4.11	11.60	s	3970	Rmet_0338	0	Op0095f_1	NA
5119	TSS_478485+2	20	1	-45	GCGGCTAGCG	-14	CATACACTAA	-142	5,1	8.03	-10.10	no	5732	Rmet_0456	rhlE1	Op0119f_1	NA
5120	TSS_1045689+2	20	3	-46	TGGACGGTGA	-14	GGCAGAATCA	-139	1,1	7.51	-8.10	no	8	Rmet_0961	ureC	Op0267f_5	MET-NUC-Pur
5121	TSS_1345981+2	20	10	-45	TCCAGCGATA	-16	CTTAAACTCG	-142	1,1	5.52	-6.60	no	173	Rmet_1226	0	Op0355f_2	EIP-TRA-ABC
5122	TSS_2021663+2	20	5	-43	TGCAAGTACA	-12	CTCAATATGT	-145	1,1	3.92	-7.60	no	27	Rmet_1864	0	Op0539f_1	NA
5123	TSS_2203905+2	20	6	-37	TTGCTCAGCG	-12	TTCACTATCG	-141	1,1	5.99	6.40	s	2350	Rmet_2037	fnrL	Op0595f_1	NA
5124	TSS_2207726+2	20	2	-44	TGGACGTTGG	-13	GTCATACTCG	-139	1,1	7.76	-5.10	no	20054	Rmet_2056	0	Op0597f_1	NA
5125	TSS_2297027+2	20	4	-46	TCGCGGTGGG	-14	GTTATGCTCG	-140	1,1	7.18	-10.10	no	3607	Rmet_2119	0	Op0613f_1	NA
5126	TSS_2356919+2	20	4	-45	TCTGACCTAC	-14	GATATGCTGT	-144	5,1	8.35	-9.60	no	203	Rmet_2156	0	Op0621f_1	NA
5127	TSS_2394599+2	20	8	-35	GCGGCACCAT	-14	ACTACGATCT	-140	5,1	9.68	8.10	s	4445	Rmet_2190	0	Op0629f_1	NA
5128	TSS_2439777+2	20	6	-48	TGGCCAGGTG	-15	GCTAAGCTGT	-141	1,1	7.43	-12.10	no	24	Rmet_2226	0	Op0637f_1	NA
5129	TSS_2746540+2	20	1	-46	TCGCCGACGT	-15	GGTACGATTC	-138	1,1	8.75	-8.10	no	6665	Rmet_2531	ivd1	Op0699f_1	NA
5130	TSS_2781200+2	20	5	-47	TGGACTGAAC	-14	GTAAGAATTT	-142	1,1	6.17	-10.10	no	358	Rmet_6537	0	Op2036f_1	NA
5131	TSS_2888701+2	20	5	-33	TTGCGCCGCG	-13	GCTATACTAG	-137	2,1	12.57	8.60	s	122	Rmet_2653	gcdH	Op0751f_1	MET-LIP-FAMet
5132	TSS_3092039+2	20	7	-36	TTGACCGCGA	-15	CATACAGTCG	-146	1,1	7.59	12.80	s	24	Rmet_6562	0	Op0801f_2	NA
5133	TSS_3098381+2	20	2	-44	TGGACCGCGT	-15	GCCATACTGG	-138	1,1	7.95	-3.10	no	12121	Rmet_2861	0	Op0805f_1	NA
5134	TSS_3255803+2	20	5	-48	CCGAACACAT	-14	TGTAGTGTTC	-144	5,1	4.48	-17.10	no	1679	Rmet_2999	mmfA	Op0845f_1	NA
5135	TSS_3366865+2	20	4	-47	TTGTTCTTTG	-27	CGCAAGTTGC	-145	1,1	3.75	-3.20	no	367	Rmet_3108	pilB	Op0875f_2	GIP-PTL-T2S
5136	TSS_3751783+2	20	8	-39	CCAGCAACCC	-14	AGTACTCTAT	-141	5,1	7.23	0.40	w	439	Rmet_3473	0	Op0965f_2	NA
5137	TSS_3920894+2	20	6	-40	TTGCAGGTGC	-16	CGTATGCTTC	-138	1,1	8.51	5.40	(m)-sba	7298	Rmet_0001	dnaA	Op0001f_1	GIP-REP-Complex
5138	TSS_200434-2	20	3	-44	GCAGCAGACC	-13	GATATTCTGG	-140	4,1	8.56	-9.60	no	7976	Rmet_0185	0	Op0056r_1	NA
5139	TSS_259668-2	20	4	-33	TTCCTTGATG	-13	GGTACTTTTT	-142	1,1	4.93	5.10	m	1055	Rmet_6407	0	Op0072r_1	NA
5140	TSS_1126163-2	20	4	-37	TTCCCGGACG	-14	GGTATCTTTG	-142	1,1	6.61	8.90	s	1152	Rmet_1035	0	Op0296r_1	NA
5141	TSS_1694749-2	20	4	-39	TCGCCGCGCC	-13	CCCATCATCA	-141	1,1	5.11	3.90	m	4119	Rmet_1569	0	Op0432r_1	NA
5142	TSS_1715562-2	20	6	-43	TTGCCTCACT	-13	TCTATCTTTC	-142	1,1	7.28	-2.60	no	31	Rmet_1590	azoR1	Op0440r_1	NA
5143	TSS_1945273-2	20	8	-33	GGCGGCACGA	-13	GTAAAGATCG	-143	4,1	4.09	0.60	w	6603	Rmet_1787	0	Op0518r_1	NA
5144	TSS_2057451-2	20	4	-28	TCGGAAATGC	-5	GGGAAAGTTC	-145	5,1	7.18	1.70	(w)-nu	122	Rmet_1889	0	Op0546r_1	NA
5145	TSS_2059161-2	20	2	-35	TTGCGGCTCG	-13	GGTATAGATC	-141	1,1	3.93	10.20	s	1217	Rmet_6486	0	Op0546r_1	NA
5146	TSS_2156715-2	20	4	-46	TGGCGCTTTC	-13	GTTACCCTAG	-142	1,1	6.40	-12.10	no	118	Rmet_1983	0	Op0578r_1	NA
5147	TSS_2354253-2	20	3	-39	TCGAATGGTG	-17	CTCATTATTG	-143	1,1	5.13	7.90	s	12261	Rmet_2148	0	Op0620r_1	NA
5148	TSS_2532125-2	20	4	-35	TTCCGGGACT	-14	TGTAACGCTA	-142	1,1	4.75	8.10	s	25	Rmet_2312	merR	Op0660r_1	NA
5149	TSS_2696747-2	20	3	-48	TTGTGCGTAC	-15	GACAGTCTGT	-145	1,1	4.44	-13.60	no	21	Rmet_2482	0	Op0688r_2	NA
5150	TSS_3566065-2	20	6	-37	TCGACGTGGC	-13	TGTACCGTGA	-142	1,1	5.61	7.90	s	5168	Rmet_3284	0	Op0924r_1	NA
5151	TSS_476813+3	20	6	-41	GCAAACCGAC	-15	AGTATTCTAA	-147	5,1	4.63	-2.60	no	1365	Rmet_R0079	0	Op1098f_1	NA
5152	TSS_477487+3	20	4	-34	TGGTACGGTC	-14	CCTAAAGTGC	-142	1,1	6.12	7.10	s	691	Rmet_R0079	0	Op1098f_1	NA
5153	TSS_1030533+3	20	2	-37	GCCAGCGCGG	-14	TGCATATTTG	-142	4,1	6.37	4.40	m	11305	Rmet_4445	0	Op1264f_1	NA
5154	TSS_1432095+3	20	6	-35	TCGCCGACAG	-14	CGTACCATCG	-139	1,1	7.01	10.10	s	7354	Rmet_4795	0	Op1396f_1	NA
5155	TSS_1502280+3	20	2	-37	GCCGCGCCAG	-13	GCCAAAGTAG	-145	4,1	4.28	2.40	w	20	Rmet_4845	0	Op1412f_1	NA
5156	TSS_2239851+3	20	5	-30	AACCTTGCCG	-10	CAAACACGTT	-144	3,0	5.37	-4.40	no	1411	Rmet_5496	0	Op1634f_1	GIP-REP-Recomb
5157	TSS_2281333+3	20	3	-49	TCGACGACAC	-14	GATATACTCT	-141	1,1	8.67	-13.10	no	1665	Rmet_5536	0	Op1646f_1	NA
5158	TSS_2460555+3	20	3	-49	GGCCCGACAT	-16	TGTAGGATGC	-141	4,1	7.88	-17.60	no	1971	Rmet_5704	0	Op1706f_1	NA
5159	TSS_1161449-3	20	1	-41	TCGCGCAAGA	-13	CGTAATTTAC	-142	1,1	4.26	-2.10	no	652	Rmet_4549	uvrA2	Op1305r_1	GIP-REP-Recomb
5160	TSS_1828723-3	20	4	-46	TTGTGACATG	-25	GTGAGAATGT	-138	1,1	7.28	1.80	w	61	Rmet_5131	0	Op1513r_3	NA
5161	TSS_2053928-3	20	6	-38	TGGTCTTTAC	-14	GATAAACTCT	-142	1,1	7.46	6.90	s	964	Rmet_6719	0	Op1583r_2	NA
5162	TSS_2406382-3	20	7	-34	TTGACCTCTT	-13	CCGATACTTC	-141	1,1	7.85	11.60	s	30	Rmet_5644	carX	Op1683r_1	NA
5163	TSS_22333+4	20	3	-34	TCAGCATGGC	-14	TGGAAAATGC	-142	5,1	8.14	7.60	s	1073	Rmet_6353	0	Op1841f_1	NA
5164	TSS_146774+4	20	4	-35	TCGCGAAACG	-14	CGCAGAATGC	-137	1,1	6.87	10.10	s	1371	Rmet_6149	bug	Op1879f_1	NA
5165	TSS_219940+4	20	1	-35	TCGCCAACCC	-15	ACCATAATGT	-142	1,1	7.70	11.10	s	655	Rmet_6081	0	Op1853f_2	NA
5166	TSS_1620-5	20	7	-48	TGCATAAGGT	-19	GTCAGCCTCG	-146	1,1	3.66	-10.60	no	15720	Rmet_6301	trbF	Op1917r_1	NA
5167	TSS_91428-5	20	6	-45	TAGCCCCTCC	-12	GGTACTTTCC	-146	1,1	3.10	-10.10	no	878	Rmet_6241	parB2	Op1901r_1	NA
5168	TSS_136931-5	20	1	-45	TCGCGGTGGC	-13	GGTAACATCG	-138	1,1	8.04	-9.10	no	177	Rmet_6281	0	Op1915r_2	NA
5169	TSS_202154+2	20	7	-34	TTGTCGAACT	-14	GCGATAATCG	-140	1,1	8.86	10.60	s	390	Rmet_6406	0	Op0057f_1	NA
5170	TSS_220898+2	20	5	-35	TTCACCCAGT	-14	GCGACGATGC	-137	1,1	6.71	10.10	s	133	Rmet_0210	sbm	Op0063f_2	NA
5171	TSS_465766+2	20	2	-34	TCGAAGCCAT	-14	ATTACCATGG	-137	1,1	6.94	8.10	s	66	Rmet_0439	ksgA	Op0117f_1	GIP-TL-Ass
5172	TSS_478299+2	20	7	-40	TCGACAACGA	-15	GTTACGCTCG	-140	1,1	7.25	4.90	(m)-sba	5918	Rmet_0456	rhlE1	Op0119f_1	NA
5173	TSS_545590+2	20	5	-42	TTCAGCGAGA	-14	CCGAGAATGT	-141	1,1	6.00	-1.10	no	6193	Rmet_0515	ompR	Op0139f_1	EIP-SIG-2Comp
5174	TSS_685629+2	20	5	-39	TCGATCTGCT	-15	TGCATGATCG	-141	1,1	6.03	5.90	m	1140	Rmet_0626	0	Op0179f_1	NA
5175	TSS_1256340+2	20	3	-36	TTGCCGTCCG	-15	ACGATCTTCT	-143	1,1	4.19	10.80	s	1667	Rmet_1147	0	Op0329f_1	NA
5176	TSS_1540255+2	20	7	-36	TTGCCAAACA	-14	TAGAAAATCC	-140	1,1	8.23	13.40	s	230	Rmet_1426	smc	Op0399f_1	DIV-Division

5177	TSS_2090104+2	20	3	-49	GGAAACATCT	-14	TGTATTCTGC	-142	4,1	7.86	-19.60	no	1196	Rmet_1924	0	Op0561f_1	NA
5178	TSS_2771654+2	20	5	-45	GGCAAAGAAT	-14	AGTATTCTTC	-143	4,1	8.50	-10.60	no	61	Rmet_6532	0	Op0707f_1	NA
5179	TSS_3619859+2	20	3	-37	TGGAGCCCTG	-14	TCTACGATGC	-140	1,1	7.39	8.90	s	1019	Rmet_3344	0	Op0931f_4	NA
5180	TSS_84624-2	20	6	-45	CCGGCAATCT	-25	GCTACGATCC	-141	5,1	11.07	-1.70	no	62	Rmet_0077	0	Op0022r_3	NA
5181	TSS_271782-2	20	3	-26	TTGTTAGCAC	-6	CTTAACCTTG	-143	1,1	5.95	0.60	(w)-nu	3203	Rmet_0251	gpmA	Op0072r_1	MET-CAH-FbP
5182	TSS_462276-2	20	3	-48	TCGACGCGCC	-19	TCCATGTTGT	-143	1,1	4.11	-8.10	no	1688	Rmet_0435	0	Op0116r_1	NA
5183	TSS_1125095-2	20	8	-49	CCAGACGCCT	-14	CCGAAGATTG	-144	5,1	6.20	-21.60	no	84	Rmet_1035	0	Op0296r_1	NA
5184	TSS_1398221-2	20	5	-45	TTGCACTACG	-13	GATAAAATGC	-138	1,1	11.02	-6.60	no	0	Rmet_1274	tnpA	Op0366r_2	NA
5185	TSS_1888878-2	20	4	-44	TTCAGTCTGA	-13	GGCACCATGG	-140	1,1	6.26	-7.10	no	907	Rmet_1740	0	Op0494r_1	NA
5186	TSS_2855462-2	20	5	-32	TTCAAATTGA	-12	TAGACCATGA	-143	1,1	5.37	6.10	m	1525	Rmet_2622	0	Op0734r_2	MET-XEN-Hex
5187	TSS_2861633-2	20	1	-42	GTACAGCAGC	-13	AATACAGTTG	-140	4,1	7.42	-6.10	no	3127	Rmet_2624	polA	Op0734r_1	MET-NUC-Pur
5188	TSS_2912506-2	20	7	-38	TTCATCGTCC	-13	AATACAATGC	-136	1,1	8.19	6.90	s	197	Rmet_2678	tolQ	Op0756r_2	NA
5189	TSS_3159700-2	20	6	-35	TGGCGGGCGT	-14	GTCAGGATCT	-141	1,1	5.49	8.10	s	1497	Rmet_2904	rpsT	Op0820r_1	GIP-TL-Ribo
5190	TSS_152937+3	20	1	-34	TGGTACGGTC	-14	CCTAAAGTGC	-142	1,1	6.12	7.10	s	691	Rmet_R0074	0	Op1006f_1	NA
5191	TSS_1004452+3	20	5	-39	TGCGCACTAC	-14	TGGATACTTG	-145	4,1	5.15	4.40	m	7501	Rmet_6663	0	Op1258f_1	NA
5192	TSS_1083743+3	20	6	-35	TTGTGCCGGT	-14	GGTAGCTTGG	-138	1,1	6.35	9.60	s	95	Rmet_4484	0	Op1278f_2	NA
5193	TSS_1129688+3	20	8	-49	GCAGGGACGT	-15	TGTAGGGTTC	-142	4,1	7.08	-20.60	no	14669	Rmet_4537	serA2	Op1298f_1	MET-AA-GlySerThr
5194	TSS_1761828+3	20	3	-39	TAGCTTTTCG	-15	GTTATCATAG	-143	2,1	10.85	5.90	m	3050	Rmet_5076	0	Op1494f_1	NA
5195	TSS_1835268+3	20	2	-49	TGGTCAGCGG	-14	GCCACAATGG	-137	1,1	7.69	-14.10	no	12300	Rmet_5149	bugT	Op1516f_1	NA
5196	TSS_1873017+3	20	4	-47	TCCGCGACGT	-14	ACTATGCTTG	-144	4,1	6.17	-12.60	no	25	Rmet_5178	0	Op1524f_1	NA
5197	TSS_1986128+3	20	4	-48	GTAGCGGCGT	-14	CGTAACCTAT	-142	4,1	5.74	-17.10	no	788	Rmet_5286	0	Op1560f_1	NA
5198	TSS_2208050+3	20	6	-35	TTGCCATTGG	-9	ACCAACATCG	-139	1,1	7.18	8.20	(s)-ba	792	Rmet_5461	0	Op1622f_1	NA
5199	TSS_2234764+3	20	5	-48	TTGATCAAAC	-14	AGCATACTAG	-142	1,1	7.16	-11.60	no	779	Rmet_6736	0	Op1632f_1	NA
5200	TSS_2482047+3	20	4	-38	TCGCAACGCA	-15	GTTACCGTGC	-143	1,1	5.90	6.90	s	10954	Rmet_6764	0	Op1716f_1	NA
5201	TSS_602976-3	20	6	-36	TCGCCTCCGA	-13	GCCATACTGT	-141	1,1	6.76	9.90	s	555	Rmet_4033	0	Op1137r_1	NA
5202	TSS_1260188-3	20	4	-46	TTGCCGCCGA	-13	CCTACGATCC	-139	1,1	8.51	-8.60	no	3205	Rmet_4627	0	Op1333r_1	EIP-SIG-2Comp
5203	TSS_1874792-3	20	4	-39	TGCTCACATG	-14	GATATCCTCA	-142	1,1	4.77	4.40	m	18	Rmet_6713	0	Op1525r_2	NA
5204	TSS_2238893-3	20	2	-35	TTGAATACTT	-14	GGCATCTTCC	-144	1,1	6.32	10.60	s	3098	Rmet_5490	pobA	Op1631r_1	MET-XEN-24D
5205	TSS_2365017-3	20	9	-34	TTGTCACCTG	-13	GCCAAAATTG	-140	1,1	8.48	11.60	s	1243	Rmet_5607	0	Op1673r_1	NA
5206	TSS_195510+4	20	4	-49	TTGAGATCCG	-14	GGTAGGATCT	-139	1,1	9.07	-12.60	no	127	Rmet_6106	copT	Op1863f_1	NA
5207	TSS_35706+2	20	6	-41	TTGCCGCTAC	-15	CTGAAAATGG	-140	1,1	7.96	3.40	(m)-sba	2279	Rmet_0036	corA1	Op0013f_1	EIP-TRA-Ion
5208	TSS_86851+2	20	4	-34	TGGTGAAATC	-14	CCGAACATGG	-142	1,1	4.55	7.10	s	2340	Rmet_0082	0	Op0023f_1	NA
5209	TSS_184268+2	20	3	-41	TGAAGAATGT	-17	CGTACTATGC	-142	4,1	8.30	3.40	(m)-sba	180	Rmet_0177	0	Op0053f_1	MET-EN-OxPhos
5210	TSS_1351384+2	20	6	-35	TCGATGCTGT	-14	GTTATCGTCG	-142	1,1	5.76	10.10	s	4316	Rmet_1233	0	Op0357f_1	NA
5211	TSS_1690933+2	20	3	-49	TTGAAAGGCT	-14	TCTATGCTCT	-136	1,1	8.66	-12.60	no	68	Rmet_1570	0	Op0433f_1	NA
5212	TSS_2244588+2	20	3	-35	TGGCAGGATT	-14	CGGATGATGC	-140	1,1	6.19	9.10	s	986	Rmet_2070	argAB	Op0601f_1	MET-AA-Urea
5213	TSS_2332015+2	20	1	-49	GCACGGATGC	-14	TGCATTATAC	-141	4,1	7.55	-20.60	no	120	Rmet_2141	0	Op0617f_1	NA
5214	TSS_2367173+2	20	6	-37	TCGAAGTTGA	-15	GGCAAAATGG	-140	1,1	8.44	9.90	s	638	Rmet_2165	0	Op0623f_1	NA
5215	TSS_2963746+2	20	7	-42	TCGGCAGTAC	-15	TGTAGTATCT	-143	5,1	9.09	0.90	(w)-sba	7931	Rmet_2736	apaH	Op0771f_1	MET-NUC-Pur
5216	TSS_3002119+2	20	4	-35	TTGCAGCGCA	-14	TCGAACATGT	-141	1,1	6.13	9.60	s	7813	Rmet_2768	ompA	Op0777f_1	NA
5217	TSS_3390029+2	20	4	-38	TCCACGAACG	-15	GGCAAAATCA	-141	1,1	6.20	7.40	s	13520	Rmet_3139	0	Op0879f_1	NA
5218	TSS_3631381+2	20	4	-34	TTGACGCTCC	-13	TGCATACCGC	-147	1,1	0.32	9.60	s	171	Rmet_3355	0	Op0935f_2	NA
5219	TSS_3787441+2	20	7	-38	TCGTCTTCCT	-15	GGTATCTTCG	-142	1,1	5.50	7.90	s	4730	Rmet_3502	0	Op0971f_1	NA
5220	TSS_3927926+2	20	5	-25	TTGTCCACAG	-1	GATAACTTGG	-145	1,1	6.24	-0.80	no	266	Rmet_0001	dnaA	Op0001f_1	GIP-REP-Complex
5221	TSS_1612680-2	20	4	-38	TGGCCAGCAA	-13	GGCAGCCTTG	-141	1,1	5.10	4.90	m	1669	Rmet_1490	0	Op0420r_1	NA
5222	TSS_2065692-2	20	2	-44	TCGAGTGGGA	-24	TCTAGGATTG	-145	1,1	6.61	1.30	w	46	Rmet_1897	ycaN	Op0550r_1	NA
5223	TSS_2092018-2	20	2	-43	TCGCCATTAA	-14	GTCATGATGC	-141	1,1	7.55	-2.10	no	1434	Rmet_1923	0	Op0560r_1	GIP-PTL-Fold
5224	TSS_2185010-2	20	1	-44	TTCACGCCGC	-23	TGCATTTTCC	-144	1,1	4.18	2.30	w	23	Rmet_R0024	0	Op0590r_2	NA
5225	TSS_2330014-2	20	6	-47	TCGCGGGTTC	-21	GCCAGACTGA	-139	1,1	5.61	-6.10	no	132	Rmet_R0029	0	Op0616r_2	NA
5226	TSS_2630646-2	20	4	-47	TTCAAGGAAG	-13	TGTACCCTGT	-140	1,1	6.52	-13.10	no	930	Rmet_2420	lepB	Op0680r_4	GIP-PTL-Exp
5227	TSS_3344942-2	20	2	-37	CCGGACCTGT	-12	TGGAAAATGG	-140	5,1	8.64	2.90	w	1572	Rmet_3087	nrdB	Op0870r_3	MET-NUC-Pur
5228	TSS_3609114-2	20	5	-39	TTCCAAGCGC	-14	GCGAAGATCG	-141	1,1	6.07	3.90	m	1039	Rmet_3336	rplJ	Op0930r_4	GIP-TL-Ribo
5229	TSS_3612264-2	20	6	-45	TTCACCCGCT	-5	GTCAAGTTTC	-145	1,1	4.52	-17.10	no	4	Rmet_R0057	0	Op0930r_3	NA
5230	TSS_3792165-2	20	5	-34	TTGCGGCGCA	-13	GCCATCATGA	-140	1,1	7.41	9.60	s	141	Rmet_3501	atpI	Op0970r_1	MET-EN-OxPhos
5231	TSS_272884+3	20	4	-27	TTGATGATTT	-3	GCGAAAGTCG	-142	1,1	6.39	1.20	(w)-nu	4495	Rmet_6620	0	Op1042f_1	NA
5232	TSS_474287+3	20	4	-26	TTGCTACGCA	-5	GAGAGACTGC	-142	1,1	6.61	1.60	(w)-nu	448	Rmet_R0076	0	Op1098f_1	NA
5233	TSS_856810+3	20	1	-39	GCGGCAATGC	-14	GCTATGATGG	-136	5,1	11.53	3.90	m	1059	Rmet_4267	ycaC	Op1212f_1	NA
5234	TSS_945732+3	20	1	-41	TGGATGAGGT	-14	TGTACGATTT	-141	1,1	7.89	0.90	(w)-sba	192	Rmet_6660	0	Op1238f_2	NA
5235	TSS_990298+3	20	5	-34	TCGACACCGA	-13	GCAACAATTT	-139	1,1	6.72	11.10	s	190	Rmet_4393	acpT	Op1248f_2	NA
5236	TSS_1621388+3	20	5	-35	GGAACACCGT	-14	TCTATCATTT	-141	4,1	9.06	8.60	s	1266	Rmet_4956	appB	Op1456f_1	MET-EN-OxPhos
5237	TSS_2279066+3	20	5	-40	TTGCCGCATG	-15	GACACAATGA	-142	1,1	8.64	5.40	(m)-sba	3932	Rmet_5536	0	Op1646f_1	NA

5238	TSS_2437634+3	20	7	-42	TCGTCCAGAC	-14	GCTAAAGTCG	-141	1,1	6.77	-1.10	no	4696	Rmet_5684	*pilE*	Op1698f_1	GIP-PTL-T2S
5239	TSS_294703-3	20	5	-30	GAACTTCTCG	-10	GTCTTTTAAG	-145	3,0	7.19	-6.40	no	2734	Rmet_3747	0	Op1043r_1	NA
5240	TSS_891205-3	20	5	-36	TTTCTCCTCC	-13	CATATGCTGA	-141	2,1	9.21	8.90	s	33	Rmet_4297	0	Op1223r_1	NA
5241	TSS_929648-3	20	7	-49	TTCCGGAGCG	-24	ACCACGGTGT	-142	2,1	6.15	-8.10	no	1432	Rmet_4334	*asfB*	Op1233r_2	NA
5242	TSS_1890469-3	20	7	-33	GGAACAGCGG	-4	CCGATACTGA	-142	4,1	6.78	-0.80	no	805	Rmet_5194	0	Op1531r_1	NA
5243	TSS_2258231-3	20	2	-46	TCCCACTACT	-14	ATTAAGATCA	-144	1,1	5.24	-11.60	no	4216	Rmet_5508	0	Op1639r_1	NA
5244	TSS_2573449-3	20	5	-36	CCGGCCGGTT	-14	TCTACTCTAG	-141	5,1	7.94	6.90	s	151	Rmet_5809	0	Op1751r_1	NA
5245	TSS_23775+4	20	6	-45	TGCGCAGCGG	-13	AGTACGCTGC	-145	4,1	5.20	-9.60	no	724	Rmet_6354	0	Op1841f_2	NA
5246	TSS_138437+4	20	3	-37	TTGCAAAGAT	-13	CTCATAATTG	-141	1,1	9.74	10.40	s	1615	Rmet_6377	0	Op2113f_1	NA
5247	TSS_220397-4	20	3	-33	TTGCAACGCC	-12	CGCATCCTTG	-139	1,1	6.52	8.60	s	7908	Rmet_6089	0	Op1854r_1	NA
5248	TSS_26620-5	20	2	-38	TGGCCAGCAA	-13	GGCAGCCTTG	-141	1,1	5.10	4.90	m	5295	Rmet_6332	0	Op1925r_1	NA
5249	TSS_81847-5	20	2	-30	GCAGGCATAT	-8	CATACGCTGT	-142	4,1	7.07	-0.80	no	390	Rmet_6230	0	Op1901r_3	NA
5250	TSS_127198-5	20	6	-35	TCGATACCGA	-12	GCCATGATCG	-142	1,1	6.29	10.70	s	1274	Rmet_6398	0	Op1911r_1	NA
5251	TSS_1464044+2	19	1	-37	GCCAGCCAAT	-14	ATCAGACTTT	-146	4,1	5.57	3.40	m	132	Rmet_1356	*phaC1*	Op0383f_1	MET-CAH-But
5252	TSS_1466109+2	19	6	-42	TGGACCAATG	-14	CCCATGATTG	-141	1,1	6.50	-0.10	no	234	Rmet_1357	*phaA*	Op0383f_2	MET-CAH-Pyr
5253	TSS_1476556+2	19	4	-38	TCGCCACCCA	-15	AGCATGATGG	-139	1,1	5.96	8.90	s	1774	Rmet_1369	*cysP*	Op0385f_1	EIP-TRA-ABC
5254	TSS_2226079+2	19	3	-36	TTGATGCCGA	-15	TGCAGCATCG	-142	1,1	5.82	10.80	s	1701	Rmet_2056	0	Op0597f_1	NA
5255	TSS_2489399+2	19	5	-30	CCTGCTACCC	-4	ACCAACCTGT	-147	5,1	4.81	-4.80	no	4412	Rmet_2271	0	Op0649f_1	NA
5256	TSS_2636028+2	19	6	-40	TCCAGGCGAT	-14	CGCATTCTGT	-147	1,1	3.19	0.40	(w)-sba	7086	Rmet_6526	0	Op0681f_1	NA
5257	TSS_2867157+2	19	8	-36	TCGCCGGCCC	-14	TCTATGCTCA	-139	1,1	6.00	10.90	s	476	Rmet_2631	*galU*	Op0737f_2	MET-CAH-Pent
5258	TSS_3275774+2	19	5	-36	TCGACCGGGC	-13	TTTATGCTGC	-141	1,1	7.25	10.90	s	8852	Rmet_3028	0	Op0849f_1	NA
5259	TSS_3578754+2	19	6	-47	TGGCCGTCGC	-9	CATATCTTTA	-144	1,1	5.32	-16.10	no	6919	Rmet_3322	0	Op0927f_1	NA
5260	TSS_92714-2	19	3	-41	TCGCGCGCTG	-16	GCCACTATCT	-139	1,1	4.97	0.90	(w)-sba	3566	Rmet_0081	*mhpD2*	Op0022r_1	NA
5261	TSS_310795-2	19	3	-39	TTGCTGCAGC	-12	GAGAGAATGA	-140	1,1	7.28	3.40	m	1549	Rmet_0292	0	Op0084r_1	NA
5262	TSS_427902-2	19	8	-41	TTGAACGGTG	-20	CGGATCATGC	-141	1,1	7.11	6.80	s	3553	Rmet_0396	0	Op0108r_1	NA
5263	TSS_1461615-2	19	4	-33	TGGCTGGTCA	-13	TATATTCTGA	-141	1,1	5.32	6.10	m	13333	Rmet_1338	0	Op0378r_1	NA
5264	TSS_1499371-2	19	3	-37	GCGTCAGGAT	-14	GGCAGAATCT	-139	5,1	8.72	6.90	s	834	Rmet_1388	*lysR*	Op0386r_1	NA
5265	TSS_1519612-2	19	3	-36	TTCATCTCTG	-13	GATAAGCTGC	-140	1,1	6.34	8.90	s	2821	Rmet_1404	*yheS*	Op0396r_1	GIP-TL-Ass
5266	TSS_2284663-2	19	2	-36	TCGCCAAGCG	-14	ACGATACTTC	-139	1,1	6.22	11.90	s	524	Rmet_2102	*der*	Op0608r_1	NA
5267	TSS_2322320-2	19	5	-34	TTCAGACAGA	-13	AGTAGAATGG	-140	1,1	8.85	11.10	s	42	Rmet_2133	0	Op0614r_1	NA
5268	TSS_2700013-2	19	9	-35	TTGCCCTGGG	-14	TCCATCCTGC	-138	1,1	7.01	10.60	s	314	Rmet_2485	*sdhD*	Op0688r_2	MET-CAH-TCA
5269	TSS_2924396-2	19	3	-49	TCCACAAGGA	-13	GCAATGATGT	-144	1,1	5.02	-15.60	no	253	Rmet_2692	0	Op0758r_1	NA
5270	TSS_3338604-2	19	2	-33	GAACTTGTCG	-13	GGGTCATGAA	-140	3,0	8.13	-3.40	no	526	Rmet_3082	0	Op0866r_1	NA
5271	TSS_3428137-2	19	4	-34	TCCCAAGGGT	-10	TTTAAAGAGG	-147	1,1	0.71	4.20	m	451	Rmet_R0050	0	Op0886r_1	NA
5272	TSS_3554330-2	19	4	-43	TCGCACTGCC	-11	GTCAAACTCC	-141	1,1	6.58	-8.10	no	26	Rmet_3278	*yedY*	Op0924r_2	NA
5273	TSS_3613317-2	19	7	-36	AAACGTCGTG	-16	GTCCCTATCT	-148	3,0	4.25	0.80	w	398	Rmet_R0060	0	Op0930r_1	NA
5274	TSS_3618015-2	19	6	-49	TCGCACAGTA	-18	CGGAGCCTGG	-147	1,1	3.36	-14.10	no	62	Rmet_R0064	0	Op0930r_1	NA
5275	TSS_3628378-2	19	6	-47	TGGAGACAGA	-22	CATATGCTTC	-142	1,1	6.79	-2.10	no	183	Rmet_3351	0	Op0932r_1	NA
5276	TSS_3781122-2	19	1	-43	TGGAAAAGCC	-12	GTCACATTTC	-145	1,1	6.74	-5.10	no	110	Rmet_3490	*priA*	Op0970r_3	GIP-REP-Complex
5277	TSS_3874420-2	19	5	-35	GCTGCAATGC	-12	GCGATATTAG	-144	5,1	7.65	6.20	m	107	Rmet_3570	0	Op0990r_1	NA
5278	TSS_2481+3	19	3	-31	TTGACTCGAC	-5	ACCACAATGT	-142	1,1	8.40	5.20	(m)-ba	286	Rmet_5819	*parA*	Op1758f_1	DIV-Division
5279	TSS_475779+3	19	1	-34	TTGTATAATG	-3	GGCAAGCTTA	-145	1,1	5.32	-0.80	no	#NV	#NV	#NV	#NV	#NV
5280	TSS_477586+3	19	2	-22	TGGAAGGGCC	-1	GATAAAAGGT	-148	1,1	1.31	-3.90	no	592	Rmet_R0079	0	Op1098f_1	NA
5281	TSS_682803+3	19	6	-35	TTGCCGCGCC	-14	CCTATGATTT	-138	1,1	9.08	12.60	s	31	Rmet_4111	0	Op1158f_1	NA
5282	TSS_697275+3	19	3	-36	TGGACATCGA	-15	TCTAACCTCT	-143	1,1	6.72	11.30	s	43	Rmet_4125	0	Op1162f_2	NA
5283	TSS_1307051+3	19	3	-39	TCGCATGAGC	-14	GATACCATCA	-143	1,1	6.96	3.90	m	115	Rmet_6679	0	Op1352f_1	NA
5284	TSS_1351858+3	19	3	-40	TTCAGGACCG	-14	GCTACTCTGC	-141	1,1	6.70	1.90	(w)-sba	4871	Rmet_4714	0	Op1360f_1	NA
5285	TSS_1670588+3	19	6	-35	TTGCGTACGC	-14	GTCATATTGC	-139	1,1	7.64	10.60	s	1294	Rmet_4993	0	Op1466f_1	NA
5286	TSS_1946590+3	19	4	-34	TTACAGGAAA	-14	CGCACAATGC	-147	4,1	6.33	7.10	s	41	Rmet_5248	*bug*	Op1548f_1	NA
5287	TSS_2249610+3	19	3	-49	TTGCGAAGGT	-3	TCTATACTGC	-140	1,1	10.86	-23.60	no	4493	Rmet_5509	0	Op1640f_1	NA
5288	TSS_110610-3	19	5	-47	TGACGACAAT	-26	TATACAGTGA	-147	4,1	6.38	-2.20	no	4	Rmet_5910	0	Op1793r_2	NA
5289	TSS_424131-3	19	5	-42	TTCCCTAATG	-13	GCTAGAGTTG	-140	1,1	7.15	-2.10	no	66	Rmet_3878	0	Op1081r_1	NA
5290	TSS_508168-3	19	4	-38	TTTCTTCGCC	-14	GTTATTCTTC	-140	2,1	12.26	5.90	m	2673	Rmet_3948	0	Op1105r_1	NA
5291	TSS_825224-3	19	2	-48	TGGCGCGCTG	-13	GATACGATTC	-141	1,1	7.49	-15.10	no	1326	Rmet_4238	0	Op1201r_1	NA
5292	TSS_858691-3	19	7	-49	TTTTTTTGCG	-13	CATATACTAG	-142	2,1	12.37	-16.10	no	1055	Rmet_4266	0	Op1211r_1	NA
5293	TSS_940470-3	19	4	-30	TATGGGCGCG	-6	TGTAGGCTCG	-141	2,1	8.95	0.20	no	44	Rmet_4343	0	Op1235r_1	NA
5294	TSS_1882783-3	19	3	-35	TTGCATCAGT	-13	AGGAAAGTAG	-144	1,1	5.14	10.20	s	587	Rmet_5187	0	Op1529r_1	NA
5295	TSS_2040956-3	19	2	-48	TGGCCGTCAC	-13	TTTATCCTGA	-140	1,1	6.76	-14.10	no	1986	Rmet_5328	*zneC*	Op1577r_1	EIP-TRA-Ion
5296	TSS_2184571-3	19	3	-43	TCGCATCGGC	-13	CGCATACTGA	-140	1,1	6.00	-5.10	no	2117	Rmet_5435	0	Op1617r_2	NA
5297	TSS_192980+4	19	2	-42	TTGCAGCGTC	-15	GATATGCTGT	-141	1,1	8.60	0.40	(w)-sba	547	Rmet_6109	*copN*	Op1865f_1	NA
5298	TSS_209755+4	19	2	-44	TTGCCCCAAA	-14	CCCACAATGG	-139	1,1	8.57	-3.60	no	648	Rmet_6091	0	Op1855f_1	NA

5299	TSS_98671-4	19	1	-39	CCAGACCGAC	-5	GCGATAGTCC	-144	5,1	7.35	-9.60	no	1555	Rmet_5964	*tnpA*	Op1814r_1	NA
5300	TSS_19235+5	19	10	-27	TTCTTGACTT	-5	AATAGACTGC	-145	1,1	5.79	1.70	(w)-nu	163	Rmet_6330	0	Op1924f_1	NA
5301	TSS_67492+5	19	3	-46	TTGCGGCCAT	-15	GATAACCTTG	-142	1,1	7.56	-8.60	no	7759	Rmet_6223	0	Op1900f_1	NA
5302	TSS_93092-5	19	6	-48	TTGCGGCTTT	-19	ACCACGAAGT	-146	1,1	1.32	-10.60	no	396	Rmet_6243	0	Op1903r_2	NA
5303	TSS_509911+2	19	5	-43	TTGCACGTTGT	-23	GCCAAAGTCT	-143	1,1	5.07	0.80	w	934	Rmet_6420	0	Op0129f_1	NA
5304	TSS_989076+2	19	3	-37	TCGAGCAGGA	-14	CTCAAAATGC	-142	1,1	7.01	8.90	s	473	Rmet_0902	0	Op0261f_2	NA
5305	TSS_1323045+2	19	9	-47	GCTGCCGGTG	-25	GACGAAATCG	-146	5,1	2.76	-6.60	no	256	Rmet_1204	0	Op0349f_2	NA
5306	TSS_2097957+2	19	5	-49	GTAAACACCA	-13	CGTATCATGG	-144	4,1	7.91	-18.10	no	102	Rmet_1930	0	Op0563f_1	NA
5307	TSS_2183749+2	19	4	-41	TGGCTACCAC	-15	GGCAAAATAA	-141	1,1	6.78	1.90	(w)-sba	530	Rmet_2020	0	Op0589f_1	NA
5308	TSS_2241532+2	19	3	-34	TTCTAACGTT	-14	AGTACGATGA	-144	1,1	6.78	8.10	s	4042	Rmet_2070	*argAB*	Op0601f_1	MET-AA-Urea
5309	TSS_3654394+2	19	7	-27	TTGCCAACCT	-6	CGCAGGGTCG	-140	1,1	4.75	2.60	(w)-nu	4712	Rmet_3384	*gspG*	Op0941f_1	GIP-PTL-T2S
5310	TSS_3860464+2	19	4	-48	TTGTGAATCA	-25	GGTCGGATCG	-145	1,1	3.40	-2.60	no	7350	Rmet_3566	0	Op0989f_1	NA
5311	TSS_3884487+2	19	3	-37	TTGCTCAAAC	-14	GGTACACTCG	-136	1,1	9.80	9.40	s	59	Rmet_3580	0	Op0995f_2	NA
5312	TSS_3903770+2	19	5	-48	GCGGAACGTG	-18	GGCATGATGA	-139	5,1	8.63	-12.10	no	6415	Rmet_3598	0	Op1001f_1	NA
5313	TSS_199800-2	19	5	-45	TTGACTGACC	-14	GGTAATCTCA	-140	1,1	7.57	-5.60	no	7342	Rmet_0185	0	Op0056r_1	NA
5314	TSS_427309-2	19	2	-40	TCGCGCACGC	-14	CGTATCCTTG	-141	1,1	6.41	1.90	(w)-sba	2960	Rmet_0396	0	Op0108r_1	NA
5315	TSS_875016-2	19	3	-49	TTGCCTCCTG	-13	CTTACATTGA	-142	1,1	7.17	-14.60	no	43	Rmet_0798	0	Op0226r_1	NA
5316	TSS_1008193-2	19	3	-41	TTGCTGCAGC	-14	GCTAACCTTG	-139	1,1	7.78	0.40	(w)-sba	3123	Rmet_0919	0	Op0264r_1	NA
5317	TSS_1612405-2	19	3	-36	TGGCAGCTTT	-13	GATATAAAGC	-145	1,1	5.17	8.90	s	1394	Rmet_1490	0	Op0420r_1	NA
5318	TSS_1836370-2	19	2	-34	TCGCACGCTG	-13	GGCACCCCTC	-143	1,1	4.63	6.10	m	68	Rmet_1694	0	Op0480r_1	NA
5319	TSS_2013642-2	19	2	-45	TTGCCCTTGT	-13	ACTATGATTA	-141	1,1	9.44	-5.60	no	10	Rmet_6480	0	Op0538r_2	NA
5320	TSS_2216052-2	19	2	-44	TCGACGAGTT	-12	GCGATGATCC	-142	1,1	6.87	-6.10	no	797	Rmet_6504	0	Op0596r_2	NA
5321	TSS_2253855-2	19	5	-35	TTCACAATCT	-13	GGTACATCAG	-146	1,1	0.03	10.70	s	8304	Rmet_2069	*hrpA*	Op0600r_1	GIP-REP-Recomb
5322	TSS_2280465-2	19	5	-36	TGGCCCGCGC	-13	GTTCTAATGT	-139	1,1	5.31	9.90	s	1037	Rmet_2098	*hflC*	Op0608r_3	GIP-PTL-Fold
5323	TSS_2296295-2	19	1	-37	TAGCCGCGAC	-14	ACTACATTAG	-143	1,1	3.45	8.90	s	1245	Rmet_2112	*rumA*	Op0610r_1	GIP-TL-Ass
5324	TSS_2527075-2	19	1	-43	TGGCGGGGGC	-14	GGCATGATTG	-140	1,1	7.21	-4.10	no	3943	Rmet_2302	*pbrR2*	Op0658r_1	NA
5325	TSS_2884732-2	19	6	-46	TTGCGAAATG	-12	CATAAAATTG	-142	1,1	10.03	-8.60	no	31	Rmet_2647	0	Op0748r_1	NA
5326	TSS_3075563-2	19	2	-25	TTCACCACCG	-4	GTGATGAACG	-146	1,1	0.91	-0.90	no	4673	Rmet_2822	0	Op0794r_1	NA
5327	TSS_3431578-2	19	3	-25	TTGCTACGCA	-4	GAGAGACTGC	-141	1,1	6.61	0.60	(w)-nu	449	Rmet_R0053	0	Op0886r_1	NA
5328	TSS_3789468-2	19	3	-43	GCAGACCTGC	-14	GCTCTAATCA	-144	4,1	5.84	-8.60	no	576	Rmet_3496	*atpA*	Op0970r_2	MET-EN-OxPhos
5329	TSS_186628+3	19	1	-36	TGGCGCCGGC	-15	CAGAAAATAC	-138	1,1	5.62	9.30	s	4	Rmet_3652	0	Op1020f_2	NA
5330	TSS_274233+3	19	4	-48	TGGCTATCAC	-14	TTCAAAATCA	-145	1,1	6.63	-13.10	no	3146	Rmet_6620	0	Op1042f_1	NA
5331	TSS_409500+3	19	9	-35	TTGCGCGTCC	-13	GCTTCATTCG	-143	1,1	2.37	9.20	s	1577	Rmet_3868	0	Op1076f_1	MET-LIP-FAMet
5332	TSS_766000+3	19	2	-39	TTCCAATTGT	-14	GTCATCATGG	-144	1,1	7.86	4.90	m	56	Rmet_4181	*xylH*	Op1180f_2	NA
5333	TSS_1083680+3	19	3	-45	TTCACCAAGG	-14	AGGACAATGC	-140	1,1	7.37	-6.10	no	158	Rmet_4484	0	Op1278f_2	NA
5334	TSS_1289200+3	19	2	-46	AAACTCCTCG	-26	GGCGACCAAA	-142	3,0	8.16	-12.20	no	2024	Rmet_4658	*tnpA*	Op2076f_1	NA
5335	TSS_1583616+3	19	5	-39	TGGCGACCCG	-15	ACTATGATTT	-140	1,1	7.34	6.90	s	217	Rmet_4920	0	Op1440f_3	NA
5336	TSS_1714813+3	19	4	-35	TGGCCGCATA	-14	TGTAGCCTAG	-141	1,1	5.38	9.10	s	449	Rmet_6705	0	Op1480f_1	NA
5337	TSS_1727080+3	19	2	-48	TCGAAAGAAT	-14	GCAAGAATCC	-144	1,1	6.73	-12.10	no	50	Rmet_5040	0	Op1482f_1	NA
5338	TSS_1847432+3	19	3	-38	TCCAATAGCG	-14	GATAAGCTGC	-143	1,1	5.24	4.40	m	136	Rmet_5149	*bugT*	Op1516f_1	NA
5339	TSS_197750-3	19	2	-49	TGCCGCCGGT	-24	GCCACGATGT	-140	1,1	5.25	-8.60	no	13826	Rmet_3649	0	Op1019r_1	NA
5340	TSS_261067-3	19	5	-36	TCGCCAGCCG	-14	TCGATAATGG	-139	1,1	7.29	12.90	s	2396	Rmet_3718	0	Op1033r_1	NA
5341	TSS_406157-3	19	4	-35	TTGTCTTCAT	-14	GGCAAGCTCC	-145	1,1	5.98	9.60	s	0	Rmet_3862	0	Op1073r_1	NA
5342	TSS_1186634-3	19	3	-35	TTGAGGGTTG	-13	CCTAGTATGG	-140	1,1	7.73	12.20	s	49	Rmet_4572	0	Op1315r_1	NA
5343	TSS_1297988-3	19	2	-47	TGGCGCCGTT	-12	TACAAATTGT	-142	1,1	4.76	-15.10	no	346	Rmet_4664	0	Op1349r_1	NA
5344	TSS_1357787-3	19	5	-35	TTGATCGCCT	-14	CGGAAAATGT	-140	1,1	7.34	11.60	s	1164	Rmet_4713	*gspG*	Op1359r_1	GIP-PTL-T2S
5345	TSS_1522347-3	19	7	-33	TTGCGAATGC	-12	TTTATCTTGC	-137	1,1	7.92	9.60	s	133	Rmet_4862	0	Op1419r_4	NA
5346	TSS_78567-4	19	2	-32	TTGACCTGAA	-12	GGTAATGTCA	-141	1,1	7.05	7.60	s	4607	Rmet_5985	*czcM*	Op1822r_1	EIP-TRA-Other
5347	TSS_58867-5	19	4	-34	TTCAACTCAT	-14	CATAAACTCG	-141	1,1	7.59	8.10	s	6721	Rmet_6203	*chrB1*	Op1895r_1	NA
5348	TSS_92495-5	19	2	-46	TTGATCGCCA	-13	GGTACAAAGA	-144	1,1	4.85	-9.60	no	1945	Rmet_6241	*parB2*	Op1901r_1	NA
5349	TSS_318497+2	19	6	-41	TTACGCCAAT	-15	CTGACAATTC	-147	4,1	5.50	0.90	(w)-sba	4965	Rmet_0308	0	Op0087f_1	EIP-TRA-Ion
5350	TSS_332768+2	19	9	-29	TTGCAGCGTT	-3	GGCATCATCA	-137	1,1	7.92	1.20	(w)-nu	1156	Rmet_0315	*ssb1*	Op0091f_2	GIP-REP-Complex
5351	TSS_470905+2	19	3	-43	TCGCACTGCGC	-15	GCGATAGTGG	-141	1,1	5.69	-1.10	no	13312	Rmet_0456	*rhlE1*	Op0119f_1	NA
5352	TSS_1066870+2	19	2	-41	GCACGCGTGC	-14	GGCACCATTG	-140	4,1	7.76	-5.60	no	2045	Rmet_0983	0	Op0277f_1	NA
5353	TSS_1270085+2	19	5	-47	CCTGAAGGCC	-14	TGCAAGATGA	-141	5,1	7.88	-16.60	no	2003	Rmet_1161	*infC*	Op0333f_3	NA
5354	TSS_3262937+2	19	5	-43	TGGCGATGCA	-15	GATAAACTCA	-140	1,1	7.98	-2.10	no	9119	Rmet_3013	*yodB*	Op0847f_1	NA
5355	TSS_3570693+2	19	7	-43	GTCCCAACCT	-14	GTTACCCTTG	-145	4,1	6.97	-6.10	no	14980	Rmet_3322	0	Op0927f_1	NA
5356	TSS_427737-2	19	4	-35	TGGCCATGAT	-15	GATAATGTTC	-141	1,1	6.51	9.10	s	3388	Rmet_0396	0	Op0108r_1	NA
5357	TSS_1630880-2	19	3	-32	TTGATATTCT	-12	TTTACCTTTT	-147	1,1	6.76	7.60	s	5	Rmet_1508	*cbbR2*	Op0424r_1	NA
5358	TSS_1775330-2	19	4	-40	TGCTTACTCT	-14	TATATGCTGG	-145	1,1	4.49	1.40	(w)-sba	344	Rmet_1641	0	Op0462r_3	NA
5359	TSS_1802144-2	19	5	-41	TTGAACGACG	-14	GGCATACTCC	-140	1,1	8.75	2.40	(w)-sba	2400	Rmet_1664	0	Op0468r_2	NA

5360	TSS_2507935-2	19	2	-47	TTGCCAACAA	-14	GCTATCTTTG	-140	1,1	8.38	-8.60	no	2149	Rmet_2283	*0*	Op0652r_1	NA
5361	TSS_2945261-2	19	2	-40	TGGAAGCCTG	-14	GGTACCATGA	-139	1,1	7.80	2.90	(w)-sba	4609	Rmet_2707	*0*	Op0766r_1	NA
5362	TSS_2970208-2	19	7	-42	TCGGCCAGAC	-13	CATACACTGC	-139	5,1	10.38	-2.10	no	719	Rmet_2733	*rfbA*	Op0770r_1	MET-CAH-NucSug
5363	TSS_3069019-2	19	3	-33	TCGCGCCAAG	-13	GCTACGGTGG	-139	1,1	5.57	5.10	m	3560	Rmet_2817	*0*	Op0792r_1	NA
5364	TSS_3613889-2	19	2	-46	TCAGCGAAGT	-26	GTGATGATGC	-143	4,1	6.85	-2.20	no	970	Rmet_R0060	*0*	Op0930r_1	NA
5365	TSS_3618382-2	19	5	-35	TTGCCAACCC	-14	CGTACTATTC	-140	1,1	9.06	12.60	s	127	Rmet_6591	*0*	Op0930r_1	NA
5366	TSS_152263+3	19	9	-41	GCAAACCGAC	-15	AGTATTCTAA	-147	5,1	4.63	-2.60	no	1365	Rmet_R0074	*0*	Op1006f_1	NA
5367	TSS_1164218+3	19	6	-39	TGGCCGTCCG	-14	GGCATAATCG	-139	1,1	8.60	5.90	m	1503	Rmet_4553	*0*	Op1306f_3	NA
5368	TSS_2128755+3	19	4	-38	TTCATGACGA	-14	GCTATTGTCA	-139	1,1	5.35	6.90	s	195	Rmet_5400	*rpoM*	Op1608f_1	GIP-TK-RNAP
5369	TSS_2175876+3	19	3	-36	CTGTAGACTA	-14	CATACCCTTT	-145	1,1	1.24	7.40	s	316	Rmet_5430	*0*	Op1616f_1	MET-GLYC-LPS
5370	TSS_2279676+3	19	2	-45	TTGCCGTACT	-22	AATAGCATTG	-143	1,1	8.08	2.40	w	3322	Rmet_5536	*0*	Op1646f_1	NA
5371	TSS_2281542+3	19	4	-43	GGCGCGACCT	-15	TGTATGATGG	-142	4,1	7.94	-5.60	no	1456	Rmet_5536	*0*	Op1646f_1	NA
5372	TSS_91235-3	19	2	-32	TTCAAACCCC	-5	TCTAAAATTG	-140	1,1	9.29	4.70	(m)-ba	32	Rmet_5892	*0*	Op1785r_1	NA
5373	TSS_234814-3	19	4	-48	GCTGAAGCGC	-5	TCTACTATCT	-142	5,1	8.70	-26.60	no	19973	Rmet_3677	*stpA*	Op1025r_1	NA
5374	TSS_556852-3	19	3	-37	TGCTCTCAAA	-13	GGCAGGATAG	-144	1,1	3.22	5.40	m	726	Rmet_3986	*0*	Op1117r_1	EIP-SIG-2Comp
5375	TSS_710205-3	19	5	-34	TGGCGCGCCC	-13	CCTAAACTTG	-139	1,1	7.20	8.10	s	37	Rmet_4135	*0*	Op1165r_1	NA
5376	TSS_973919-3	19	5	-37	TTGACCCAGC	-13	GTCATTATCT	-140	1,1	7.95	10.40	s	2413	Rmet_4372	*0*	Op1247r_1	NA
5377	TSS_1290214-3	19	1	-43	TGGCCTACTA	-13	GGTAACCTGT	-142	1,1	6.85	-5.10	no	3405	Rmet_4654	*0*	Op1345r_1	NA
5378	TSS_1311288-3	19	5	-41	TGAACGAAGG	-15	GGCATCATCC	-143	4,1	6.57	1.40	(w)-sba	429	Rmet_4679	*0*	Op1353r_2	NA
5379	TSS_1561631-3	19	1	-35	TGGCCAGCAG	-13	GGCAAGATGT	-143	1,1	6.75	10.70	s	2671	Rmet_4894	*0*	Op1431r_1	MET-NUC-Pyr
5380	TSS_1819390-3	19	5	-47	TTGACATACG	-14	CCTAGACTCG	-143	1,1	9.12	-7.60	no	43	Rmet_5124	*0*	Op1509r_1	NA
5381	TSS_1894358-3	19	6	-44	TTCTATGTGT	-16	GTGATACTTT	-148	1,1	5.89	-4.10	no	53	Rmet_5198	*0*	Op1533r_1	MET-AA-GlySerThr
5382	TSS_2311317-3	19	5	-33	TGGCGAAAGT	-12	GATACTCTCG	-139	1,1	7.10	7.10	s	2592	Rmet_6746	*0*	Op2099r_1	NA
5383	TSS_23392+4	19	5	-31	TGGACGTCGC	-3	AAGACCATTC	-143	1,1	5.11	0.70	(w)-ba	14	Rmet_6353	*0*	Op1841f_1	NA
5384	TSS_132255+4	19	5	-47	TCGAAAGCCG	-14	TATCTACTTC	-145	1,1	3.05	-11.10	no	11	Rmet_6168	*traW*	Op1887f_1	NA
5385	TSS_110661+5	19	1	-36	TGGCCGATGT	-6	GTGAGCATCT	-144	1,1	5.67	1.90	(w)-ba	108	Rmet_6258	*rhsC*	Op1906f_1	NA
5386	TSS_37935+2	18	7	-47	TTCTGAGACA	-16	GCCACATTCT	-143	1,1	4.75	-10.10	no	50	Rmet_0036	*corA1*	Op0013f_1	EIP-TRA-Ion
5387	TSS_586657+2	18	2	-35	TGGCAGTCGA	-14	GCCACACTGA	-139	1,1	6.79	8.10	s	56	Rmet_0550	*0*	Op0153f_1	NA
5388	TSS_1334301+2	18	5	-41	TCGCATGGTG	-15	CCTAGCATGT	-140	1,1	6.08	0.90	(w)-sba	9301	Rmet_1224	*bzdA*	Op0355f_1	MET-XEN-Benz
5389	TSS_1800249+2	18	4	-38	TTGTAGCGAT	-14	GGCACTATTG	-142	1,1	7.01	6.40	s	4654	Rmet_1668	*tnp*	Op0469f_1	NA
5390	TSS_2673980+2	18	4	-35	TTCCGGCTCA	-13	GGCACGATAT	-141	1,1	5.38	8.70	s	27760	Rmet_2489	*mdh*	Op0689f_1	MET-CAH-TCA
5391	TSS_3076889+2	18	3	-36	TCGCATGCGC	-13	GCTAATATCG	-138	1,1	7.21	8.90	s	825	Rmet_2829	*0*	Op0797f_1	NA
5392	TSS_3204781+2	18	4	-35	TCGCCCTGGC	-15	GGTATTCTGT	-140	1,1	7.78	9.10	s	423	Rmet_2948	*0*	Op0831f_2	NA
5393	TSS_3456485+2	18	3	-48	GCAACGACGC	-13	GGTACACTAC	-139	4,1	10.72	-17.60	no	184	Rmet_3185	*0*	Op0895f_1	EIP-TRA-ABC
5394	TSS_373629-2	18	6	-35	TGGATATCTG	-13	GGTATCGTGG	-139	1,1	6.66	11.70	s	2273	Rmet_0351	*0*	Op0098r_1	NA
5395	TSS_651332-2	18	3	-40	CGGTCATATC	-13	ACTATGCTTG	-148	1,1	1.96	-0.10	no	1285	Rmet_0600	*mdtB*	Op0170r_1	EIP-TRA-Ion
5396	TSS_690810-2	18	3	-37	TTCCCTCGCT	-13	GTCATACTGT	-139	1,1	7.31	7.90	s	4330	Rmet_0625	*0*	Op0178r_1	NA
5397	TSS_930143-2	18	2	-43	CCTGCCACGG	-13	AGTATTCTCC	-141	5,1	8.36	-8.60	no	223	Rmet_R0008	*0*	Op0242r_1	NA
5398	TSS_1594121-2	18	4	-49	TCGCAAAGTC	-23	GGCAGCATCC	-141	1,1	6.36	-7.10	no	804	Rmet_1472	*0*	Op0412r_1	NA
5399	TSS_2047646-2	18	4	-41	TCGAGGTGAC	-15	GACATCATCC	-141	1,1	6.50	1.90	(w)-sba	1056	Rmet_R0021	*0*	Op0544r_2	NA
5400	TSS_2117464-2	18	3	-37	TTGTGGCTGG	-14	GGTATCTTAG	-142	1,1	6.34	9.40	s	195	Rmet_1946	*0*	Op0570r_1	NA
5401	TSS_2133615-2	18	3	-39	TCGACAAGGT	-13	GGCAAGCTGC	-141	1,1	6.53	3.90	m	1204	Rmet_1958	*0*	Op0574r_2	NA
5402	TSS_2282592-2	18	2	-41	TTTCGTCCCC	-13	TATAAATTTG	-144	2,1	10.53	-1.10	no	5	Rmet_2101	*hfq*	Op0608r_2	NA
5403	TSS_2377196-2	18	6	-36	TTGCAGGTGT	-14	CCCAAAATTT	-140	1,1	8.56	11.40	s	1201	Rmet_2171	*0*	Op0624r_1	NA
5404	TSS_2769863-2	18	6	-24	TAGCTGTTGG	-2	AATAAATTGA	-149	2,1	7.13	-1.30	no	151	Rmet_2544	*0*	Op0704r_1	NA
5405	TSS_2903029-2	18	2	-33	TTTCTCCCCG	-13	GATATGCTGG	-145	2,1	10.99	6.10	m	903	Rmet_2667	*0*	Op0756r_5	NA
5406	TSS_2966182-2	18	2	-49	TTGCGGTATA	-13	TGTAGTATGC	-143	1,1	8.43	-15.60	no	268	Rmet_2729	*0*	Op0770r_1	NA
5407	TSS_3256494-2	18	3	-41	TTGCCACTGG	-13	ACCAATATCT	-140	1,1	6.50	1.40	(w)-sba	3052	Rmet_2995	*ptxA*	Op0842r_1	EIP-TRA-ABC
5408	TSS_3329139-2	18	6	-41	TTGCGCGCAA	-13	CTTAACATCA	-145	1,1	7.20	0.40	(w)-sba	151	Rmet_3072	*0*	Op0862r_2	NA
5409	TSS_3815376-2	18	3	-35	TTGCGCTTCG	-14	CGTAATCTCG	-137	1,1	6.89	9.60	s	364	Rmet_3522	*pldB*	Op0974r_2	MET-LIP-GlycPLip
5410	TSS_79463+3	18	6	-40	TTGCCCCGCC	-14	CCTAGAGTGT	-139	1,1	7.52	4.40	(m)-sba	21	Rmet_5883	*0*	Op1780f_2	NA
5411	TSS_152960+3	18	4	-49	TCGGACATCG	-13	GCTTAACTGC	-148	5,1	4.05	-19.10	no	668	Rmet_R0074	*0*	Op1006f_1	NA
5412	TSS_215182+3	18	6	-40	TCAGGCACGT	-14	TTTATCATTT	-145	4,1	8.25	1.40	(w)-sba	210	Rmet_3678	*0*	Op1026f_1	DIV-MOT-Chemotax
5413	TSS_475790+3	18	1	-45	TTGTATAATG	-14	GGCAAGCTTA	-145	1,1	5.32	-8.60	no	#NV	#NV	#NV		#NV
5414	TSS_608093+3	18	3	-43	TGGCGATGCA	-15	GATAAACTCA	-140	1,1	7.98	-2.10	no	4772	Rmet_4046	*ggt*	Op1140f_1	MET-LIP-Others
5415	TSS_910698+3	18	5	-38	TTGCCACGTG	-13	GGTATAAAGG	-141	1,1	6.97	8.40	s	3347	Rmet_4319	*0*	Op1230f_1	NA
5416	TSS_1146822+3	18	3	-44	TCGTACCCCG	-14	CCTACAGTAG	-142	1,1	4.50	-6.10	no	21	Rmet_4540	*0*	Op1300f_1	NA
5417	TSS_1356560+3	18	3	-44	TGGCGGGCAT	-14	GCGATGATCC	-140	1,1	6.31	-6.10	no	169	Rmet_4714	*0*	Op1360f_1	NA
5418	TSS_1360714+3	18	5	-29	CCGGCACGTG	-5	CGGACAATGC	-138	5,1	9.31	0.70	(w)-nu	80	Rmet_4718	*0*	Op1362f_1	NA
5419	TSS_2318891+3	18	3	-41	GCAAACGATT	-16	GTTAATATCC	-143	4,1	7.17	-1.60	no	233	Rmet_5565	*0*	Op1654f_1	NA
5420	TSS_540426-3	18	2	-35	GCACGAACGC	-13	ATCAATCTCC	-142	4,1	6.79	4.20	m	2009	Rmet_6638	*0*	Op1113r_1	NA

5421	TSS_2250684-3	18	4	-40	TTCCTCGGAA	-13	GATACAGTTC	-143	1,1	6.78	0.90	(w)-sba	2198	Rmet_5504	0	Op1637r_1	NA
5422	TSS_2285382-3	18	5	-35	TTGCAAAACT	-10	ATAACAATGT	-141	1,1	7.61	9.20	s	2426	Rmet_6741	0	Op1645r_1	NA
5423	TSS_2316959-3	18	2	-36	TAGCCGCTGG	-13	CTGACAATGC	-145	1,1	3.89	9.90	s	1393	Rmet_5562	0	Op1651r_1	NA
5424	TSS_2477098-3	18	2	-48	GGAGGGGGCGT	-14	TGTATTGTCG	-141	4,1	6.63	-18.60	no	1293	Rmet_5717	0	Op1711r_1	NA
5425	TSS_2487318-3	18	5	-33	TACTCAACGA	-13	AGTATCCTGA	-145	1,1	1.82	6.60	s	1555	Rmet_5726	cat	Op1715r_3	NA
5426	TSS_24790+4	18	4	-47	GCGGCCCGGT	-15	GGCAGGATCA	-141	5,1	8.37	-14.10	no	155	Rmet_6355	0	Op1841f_2	NA
5427	TSS_66347-4	18	2	-46	TCGCACTGCT	-13	TGCATAGTGG	-141	1,1	6.11	-11.10	no	374	Rmet_5991	merT	Op1826r_1	EIP-TRA-Other
5428	TSS_10190+5	18	4	-46	TGGAAAAGGG	-15	GGCAACATCA	-143	1,1	6.71	-8.10	no	836	Rmet_6325	0	Op1920f_2	NA
5429	TSS_19245+5	18	3	-37	TTCTTGACTT	-15	AATAGACTGC	-145	1,1	5.79	8.90	s	153	Rmet_6330	0	Op1924f_1	NA
5430	TSS_111962-5	18	2	-40	TGGCGCGGCC	-12	GGTAGCATAG	-142	1,1	6.99	-0.10	no	1861	Rmet_6257	0	Op1905r_1	NA
5431	TSS_560395+2	18	3	-48	GGCCGGGCGG	-15	CATATAATCC	-137	4,1	8.73	-15.60	no	237	Rmet_0525	0	Op0143f_1	NA
5432	TSS_711808+2	18	5	-37	TTGTGCTCGT	-15	CGTAAAGTCG	-140	1,1	7.24	10.40	s	7857	Rmet_0654	0	Op0181f_1	NA
5433	TSS_996517+2	18	3	-46	TGCCGCCTAA	-23	TCTAAAATGT	-140	1,1	7.47	-1.60	no	289	Rmet_0911	ilvI	Op0263f_2	MET-CAH-But
5434	TSS_1003546+2	18	2	-42	TTGCCACAAT	-15	GGCAAACTCC	-137	1,1	8.88	1.40	(w)-sba	1761	Rmet_0920	0	Op0265f_1	EIP-TRA-ABC
5435	TSS_1160871+2	18	4	-38	ATCCTGCGCC	-14	GGTATCGTCG	-141	2,1	7.55	2.90	w	456	Rmet_1069	0	Op0307f_2	NA
5436	TSS_1269794+2	18	3	-33	AAACGCATCA	-13	AACTCGAACT	-147	3,0	4.24	-3.40	no	198	Rmet_1160	thrS	Op0333f_2	MET-AA-GlySerThr
5437	TSS_1425404+2	18	4	-41	GCGGCTGTTT	-15	GTTAACCTTG	-145	5,1	7.24	-2.10	no	20	Rmet_1309	tnpA	Op0375f_1	NA
5438	TSS_2117499+2	18	6	-49	TTGAGCGAAA	-15	TGGAAAATAC	-146	1,1	7.59	-12.60	no	732	Rmet_1948	ompB	Op0571f_1	EIP-SIG-2Comp
5439	TSS_2377565+2	18	4	-42	TTCCAGAGTC	-15	GGTAAGCTGC	-140	1,1	7.69	-2.10	no	2311	Rmet_R0034	0	Op0625f_1	NA
5440	TSS_3021299+2	18	3	-42	TCTATGTGCG	-15	CGTAGACTTA	-145	2,1	4.21	-1.60	no	150	Rmet_2774	panC	Op0779f_1	MET-OAA-Other
5441	TSS_3253597+2	18	3	-46	TGGAACGTGT	-14	CGAAAAATAG	-147	1,1	5.83	-9.10	no	79	Rmet_2996	ptxR	Op0843f_1	NA
5442	TSS_3507419+2	18	4	-47	GTCAAACTCA	-14	CGGAGAATAG	-148	4,1	4.23	-13.10	no	1216	Rmet_3233	0	Op0917f_1	NA
5443	TSS_3560084+2	18	5	-38	CCTGAAGTGG	-13	GCGGTAATAC	-146	5,1	2.52	0.40	w	1075	Rmet_3285	engB	Op0925f_1	NA
5444	TSS_229737-2	18	2	-34	TCGAAGAAGA	-13	GCTATCCTCT	-141	1,1	7.40	9.10	s	3099	Rmet_0213	crp1	Op0064r_1	NA
5445	TSS_275755-2	18	2	-46	CTGCGCACGC	-14	GGTAAGCTGC	-140	1,1	3.98	-12.60	no	20	Rmet_0257	yibK	Op0074r_1	GIP-TL-Ass
5446	TSS_832047-2	18	2	-37	TTGTTCAGGC	-16	TAGAGGATCT	-142	1,1	4.52	8.80	s	2571	Rmet_0753	0	Op0214r_1	NA
5447	TSS_2339312-2	18	1	-33	TCGAAGAAGT	-13	GGTATCGTTC	-141	1,1	7.20	7.10	s	1232	Rmet_2145	yejB	Op0618r_1	NA
5448	TSS_3088671-2	18	2	-35	TTGTCTTATT	-13	CCTATATTGC	-142	1,1	8.42	13.20	s	131	Rmet_2840	mobC	Op0798r_1	NA
5449	TSS_3190037-2	18	3	-46	TCAAGGAAGT	-14	GGCATGATCT	-142	4,1	8.01	-10.60	no	220	Rmet_2934	gpo	Op0828r_1	MET-LIP-Others
5450	TSS_3468510-2	18	2	-39	TGGCAATCGT	-14	CAGATAATGG	-142	1,1	7.75	5.90	m	914	Rmet_3191	0	Op0896r_1	NA
5451	TSS_3572483-2	18	5	-34	TGGCCTGATC	-13	AGCAACATGC	-142	1,1	5.56	8.10	s	476	Rmet_3297	secY	Op0926r_2	GIP-PTL-Exp
5452	TSS_3616863-2	18	4	-42	TGGCTGTCGT	-19	GAGATGTTGG	-144	1,1	4.06	1.90	(w)-nu	501	Rmet_R0063	0	Op0930r_1	NA
5453	TSS_148533+3	18	4	-32	TGGTTGGGTT	-6	GTCAGTTTTC	-149	1,1	2.37	0.70	(w)-ba	61	Rmet_R0070	0	Op1006f_1	NA
5454	TSS_152659+3	18	3	-47	TCAGCGAAGT	-27	GTGATGATGC	-143	4,1	6.85	-3.20	no	969	Rmet_R0074	0	Op1006f_1	NA
5455	TSS_832107+3	18	4	-43	TCGAGATTCG	-14	CGTAATATCC	-142	1,1	7.09	-2.10	no	605	Rmet_6655	0	Op1202f_3	NA
5456	TSS_971612+3	18	1	-38	TTGTGCTCTA	-14	GAGAGTCTGG	-144	1,1	3.89	5.40	m	122	Rmet_4373	0	Op1248f_1	NA
5457	TSS_1755033+3	18	4	-34	GCTGCAACAG	-5	CATAGACTCT	-142	5,1	9.02	-0.80	no	3197	Rmet_5069	0	Op1492f_1	NA
5458	TSS_1919637+3	18	3	-41	TTGCACGCTT	-14	TTAAGAGTGC	-142	1,1	4.74	0.40	(w)-sba	5397	Rmet_5229	0	Op1542f_1	NA
5459	TSS_118261-3	18	4	-28	TGGCGCAAGC	-2	GGCAAAATAC	-139	1,1	7.88	-1.30	no	1109	Rmet_5915	0	Op1795r_1	NA
5460	TSS_1406667-3	18	4	-30	TTTCTTCGCG	-10	AACATGTTGT	-143	2,1	9.56	2.10	(w)-sba	303	Rmet_4760	0	Op1385r_1	NA
5461	TSS_1639987-3	18	5	-44	TCGCGTATGA	-13	AGTAGCATAC	-142	1,1	5.83	-7.10	no	70	Rmet_4966	0	Op1457r_1	NA
5462	TSS_1871754-3	18	4	-40	TTGCGGAGCG	-14	GATATGGTTA	-140	1,1	6.96	3.40	(m)-sba	2447	Rmet_5171	cspA	Op1521r_1	NA
5463	TSS_2232060-3	18	6	-33	GGCACGATGT	-13	GTTGCAATGG	-147	4,1	3.46	2.60	w	1328	Rmet_5487	0	Op1629r_1	GIP-REP-Recomb
5464	TSS_2479280-3	18	4	-48	TCGCCAACAG	-13	ACTATGATCC	-141	1,1	7.91	-12.10	no	872	Rmet_5720	0	Op1713r_1	NA
5465	TSS_2505766-3	18	2	-35	TTCTGCTGCG	-14	ACTACGATCG	-139	2,1	10.36	9.10	s	1721	Rmet_5742	0	Op1717r_1	NA
5466	TSS_2550507-3	18	3	-34	TTGTATGGAT	-14	CTTAGCATCC	-141	1,1	7.06	8.60	s	63	Rmet_5793	cyoA	Op1743r_1	MET-EN-OxPhos
5467	TSS_162407+4	18	6	-36	TGGCAGCCGC	-15	GGCATTGTGT	-142	1,1	4.59	8.30	s	828	Rmet_6380	0	Op1875f_1	NA
5468	TSS_225986+4	18	4	-43	GCGTCACCTC	-14	GTCATACTCG	-143	5,1	6.94	-5.10	no	2980	Rmet_6071	0	Op1849f_1	NA
5469	TSS_36842-4	18	2	-34	TTCATTCCGG	-14	TCGAAGATGG	-143	1,1	4.39	8.10	s	2904	Rmet_6026	0	Op1836r_1	NA
5470	TSS_133820-4	18	1	-41	CCGGCATGGG	-12	GTAAGAATGG	-143	5,1	8.11	-3.10	no	2601	Rmet_6170	0	Op1888r_1	NA
5471	TSS_203997+2	18	1	-42	TCTGCAGGAC	-13	GCGAGAATAC	-141	5,1	9.24	-2.60	no	309	Rmet_R0002	0	Op0057f_1	NA
5472	TSS_278388+2	18	5	-45	TTGCGGGGCA	-14	TGTAAATTGG	-141	1,1	8.17	-6.60	no	6	Rmet_0261	coxB	Op0075f_2	MET-EN-OxPhos
5473	TSS_798897+2	18	6	-42	GCCGCACCGC	-14	CTTATCGCTT	-141	4,1	6.03	-4.60	no	208	Rmet_0722	rpsA	Op0205f_2	GIP-TL-Ribo
5474	TSS_999709+2	18	6	-36	GCTGCCCGTG	-14	CGTACGCTAC	-138	5,1	7.63	5.40	m	486	Rmet_0914	0	Op0263f_3	NA
5475	TSS_1163815+2	18	4	-49	TCGTCCTTTA	-15	TGAATAATTA	-148	1,1	5.51	-13.10	no	1337	Rmet_1073	slyD	Op0309f_1	GIP-PTL-Fold
5476	TSS_1220258+2	18	5	-45	TTCCACGCCA	-14	GTCAGGATCA	-142	1,1	4.84	-9.10	no	7480	Rmet_1122	gltP2	Op0321f_1	NA
5477	TSS_1362554+2	18	4	-37	TGGCCACATA	-14	CGCAACATGA	-143	1,1	5.52	8.90	s	28	Rmet_1239	ritA2	Op0357f_2	NA
5478	TSS_2011873+2	18	1	-48	TTGATACACG	-14	GGTAGACTCG	-142	1,1	9.17	-10.60	no	76	Rmet_1856	lysP	Op0537f_1	EIP-TRA-Ion
5479	TSS_2098062+2	18	4	-45	TGCAGCGGGT	-23	GCCAACTTCA	-145	1,1	2.56	-1.60	no	2139	Rmet_1932	dppA	Op0565f_1	NA
5480	TSS_2369881+2	18	3	-35	TTGCGACGCA	-14	TCCACTATCG	-142	1,1	6.57	10.60	s	55	Rmet_2167	0	Op0623f_1	GIP-PTL-Exp
5481	TSS_2453462+2	18	5	-33	TCGCCCAGCG	-12	AATATTATCC	-139	1,1	6.93	9.10	s	1376	Rmet_2239	glpD	Op0641f_1	MET-LIP-GlycPLip

5482	TSS_2620858+2	18	4	-42	GCACGGCCGC	-15	CGTATCCTCG	-140	4,1	7.93	-5.60	no	636	Rmet_2411	0	Op0679f_1	NA
5483	TSS_3001418+2	18	4	-36	TTGATCTCGA	-14	GCTGCAATTC	-142	1,1	4.34	11.40	s	8514	Rmet_2768	*ompA*	Op0777f_1	NA
5484	TSS_3093179+2	18	5	-35	TTGCCCCACT	-14	CCTATCGTGA	-139	1,1	6.70	11.60	s	625	Rmet_2844	0	Op0801f_2	NA
5485	TSS_3292523+2	18	4	-33	TTGACGGCGA	-11	ACGATGAAGG	-144	1,1	1.66	9.20	s	4874	Rmet_3042	0	Op0855f_1	NA
5486	TSS_3336616+2	18	5	-36	TTGTACTCCG	-15	ACTAGTGTGA	-143	1,1	4.25	9.80	s	37	Rmet_3081	0	Op0865f_2	NA
5487	TSS_3392930+2	18	1	-35	TTGACCTGCG	-15	GGCATGATCC	-136	1,1	8.75	11.60	s	10619	Rmet_3139	0	Op0879f_1	NA
5488	TSS_3417287+2	18	6	-32	TCAAGCGCAT	-5	ACGACGCTTA	-144	4,1	3.90	-0.80	no	6836	Rmet_3159	*hipO*	Op0885f_1	MET-AA-Phe
5489	TSS_3570556+2	18	3	-39	TCGCATCTTG	-14	AGTACTATGT	-143	1,1	6.38	3.90	m	15117	Rmet_3322	0	Op0927f_1	NA
5490	TSS_396651-2	18	6	-27	TTCTTCTATC	-3	GCCAACATGC	-144	1,1	5.34	-1.30	no	8806	Rmet_0365	*coxS*	Op0102r_1	MET-EN-Methane
5491	TSS_809027-2	18	2	-41	TGGCGACGCT	-14	GGCATGATCA	-139	1,1	6.85	0.90	*(w)-sba*	10885	Rmet_0720	0	Op0204r_1	NA
5492	TSS_1566595-2	18	3	-44	GCGGCCGCGC	-13	TGTAAATTTT	-138	5,1	9.32	-9.10	no	10683	Rmet_1434	*map*	Op0402r_1	GIP-TL-Ass
5493	TSS_1717956-2	18	5	-27	TTGCCAAGAT	0	TGACTCATTT	-147	1,1	2.18	-1.80	no	750	Rmet_1592	*tnpA*	Op2009r_1	NA
5494	TSS_2024352-2	18	1	-44	TGGATATGGC	-14	GCCACAATGA	-139	1,1	8.28	-4.10	no	3000	Rmet_1863	0	Op0538r_1	MET-LIP-FASyn
5495	TSS_2092684-2	18	1	-42	CCGCAAGAAG	-14	GTCATACTCC	-147	1,1	2.58	-4.10	no	460	Rmet_6493	0	Op0562r_2	NA
5496	TSS_2161967-2	18	3	-34	TTCCATGCGA	-13	TGTATGCTGC	-142	1,1	6.96	8.10	s	832	Rmet_1988	0	Op0582r_1	NA
5497	TSS_2184986-2	18	5	-48	TTGCATTTTC	-14	CGTATAGTTC	-139	1,1	8.94	-11.60	no	715	Rmet_2019	0	Op0588r_1	NA
5498	TSS_2610539-2	18	3	-36	TCGAGCACGG	-13	GATATCTTGA	-142	1,1	5.58	9.90	s	13105	Rmet_2384	*armR*	Op0672r_1	NA
5499	TSS_2814020-2	18	2	-42	GCGGCACGCT	-13	GCTATCGTAT	-140	5,1	8.95	-4.10	no	46	Rmet_2587	*gstN*	Op0722r_1	MET-OAA-GSH
5500	TSS_2950729-2	18	3	-40	TCGCCCGGGT	-13	TATAAACTGG	-139	1,1	7.88	1.90	*(w)-sba*	108	Rmet_2716	*cpsG*	Op0768r_1	MET-CAH-FbP
5501	TSS_3317879-2	18	6	-39	GCGTACGGCG	-13	AGTACGATCA	-143	5,1	7.11	-0.10	no	819	Rmet_3059	0	Op0860r_1	NA
5502	TSS_3574511-2	18	4	-49	GGAGAAACAG	-14	GATATGCTGA	-142	4,1	8.98	-19.60	no	366	Rmet_3302	*rplF*	Op0926r_2	GIP-TL-Ribo
5503	TSS_3615877-2	18	2	-22	GCATGTGGTG	-2	CGATTACAGG	-149	3,0	2.35	-13.40	no	#NV	#NV	#NV	#NV	#NV
5504	TSS_3631210-2	18	3	-40	TAGCGATACA	-13	CGTACGCTTG	-146	1,1	3.69	0.90	*(w)-sba*	676	Rmet_3353	*tnpA*	Op0934r_1	NA
5505	TSS_387101+3	18	2	-37	TCGACGGTCC	-15	GATACAGTAC	-138	1,1	6.74	10.90	s	544	Rmet_3843	0	Op1068f_1	NA
5506	TSS_477224+3	18	3	-23	GCGGCTAGAC	-2	ATGAACCTTT	-146	5,1	4.91	-6.90	no	954	Rmet_R0079	0	Op1098f_1	NA
5507	TSS_623357+3	18	3	-49	CCGGCCAGCC	-26	CGCGTTCTCC	-144	5,1	1.04	-9.10	no	7584	Rmet_4066	0	Op1142f_1	NA
5508	TSS_1001421+3	18	6	-44	GGAAGACCGG	-15	GGTAAGCTCA	-140	4,1	7.56	-8.60	no	837	Rmet_4407	0	Op1256f_1	NA
5509	TSS_1165458+3	18	1	-43	GTACACACCA	-14	CCTCTAATAC	-148	4,1	3.41	-7.10	no	263	Rmet_4553	0	Op1306f_3	NA
5510	TSS_1167972+3	18	5	-47	TCGCTGCGCC	-14	ACTATACTGT	-144	1,1	7.23	-11.10	no	649	Rmet_4558	0	Op1308f_1	NA
5511	TSS_1874513+3	18	4	-43	TCGAGCAAGG	-20	TTGATCATGC	-143	1,1	5.66	2.90	*w*	1539	Rmet_5182	0	Op1526f_1	NA
5512	TSS_2345697+3	18	5	-40	GCAGACGCAC	-15	CGTAAGCTGC	-140	4,1	8.63	-2.60	no	2009	Rmet_5593	0	Op1666f_1	NA
5513	TSS_2371198+3	18	6	-40	TTGTCTCTTG	-14	GCTATTCTAC	-140	1,1	7.01	4.40	*(m)-sba*	2691	Rmet_5617	0	Op1676f_1	MET-LIP-Ster
5514	TSS_2550530+3	18	2	-33	TGGCCTGAGG	-13	GCTAAGGTAA	-144	1,1	4.96	6.10	m	25	Rmet_5794	0	Op1744f_1	NA
5515	TSS_688952-3	18	4	-40	TGGCATACCC	-13	GTCAGAATGA	-141	1,1	6.95	0.90	*(w)-sba*	74	Rmet_4117	*pdxR*	Op1159r_2	NA
5516	TSS_1092619-3	18	3	-37	GCCGAAGAGC	-9	TCCATATATC	-147	4,1	3.02	-2.60	no	370	Rmet_4492	0	Op1279r_1	NA
5517	TSS_1463520-3	18	1	-34	TTGCAAAAGT	-14	ACTATAGTCC	-141	1,1	9.42	10.60	s	26	Rmet_4815	0	Op1403r_1	NA
5518	TSS_1536735-3	18	3	-39	TCACCGACAC	-13	ATTACCGTCA	-143	4,1	4.96	1.40	w	1516	Rmet_4872	0	Op1423r_1	NA
5519	TSS_2172332-3	18	3	-45	TTGCCCGAGT	-13	GATATAGTGG	-137	1,1	9.71	-5.60	no	1174	Rmet_5423	*atsA*	Op1613r_1	MET-LIP-Others
5520	TSS_2204586-3	18	4	-44	GGAGACAAAG	-13	TACATACTCC	-145	4,1	7.44	-11.60	no	793	Rmet_5456	*tnpA*	Op1621r_1	GIP-REP-Recomb
5521	TSS_2237622-3	18	3	-35	TTGAGATCGG	-11	AACATCATCC	-142	1,1	7.10	11.20	s	1827	Rmet_5490	*pobA*	Op1631r_1	MET-XEN-24D
5522	TSS_34223+4	18	3	-35	TTGGCAACTCT	-10	TCTCAAATGA	-146	1,1	4.26	6.70	s	639	Rmet_6024	0	Op1835f_1	NA
5523	TSS_152019+2	17	2	-48	GTCCAAGCAG	-14	GGCAAGATGG	-139	4,1	7.35	-17.10	no	2596	Rmet_0150	*pyrE*	Op0043f_1	MET-NUC-Pyr
5524	TSS_444603+2	17	4	-34	TGCAGACCGG	-14	GCGATGATGC	-138	1,1	5.46	7.60	s	96	Rmet_0419	*ybhB*	Op0115f_1	MET-CAH-FbP
5525	TSS_666368+2	17	3	-35	TGCCACATCC	-14	GGCAGGCTGC	-140	1,1	4.13	5.60	m	95	Rmet_0612	0	Op0173f_1	NA
5526	TSS_1227640+2	17	3	-28	TTGCACCGGC	-4	CCTAGAGTGC	-139	1,1	7.93	2.20	*(w)-nu*	98	Rmet_1122	*gltP2*	Op0321f_1	NA
5527	TSS_1558507+2	17	4	-49	CCTGATGGGC	-15	GCCACCATCG	-144	5,1	6.77	-20.60	no	725	Rmet_1438	*frr*	Op0403f_2	NA
5528	TSS_1562722+2	17	5	-41	TGGCCGTTGA	-17	GGCAGATTCT	-140	1,1	5.83	2.90	*(w)-sba*	9	Rmet_1442	*yaeL*	Op0403f_2	NA
5529	TSS_1819197+2	17	4	-34	TTCCTTGACG	-14	TGGAGAATGG	-142	1,1	5.61	7.10	s	40	Rmet_1682	0	Op0475f_1	NA
5530	TSS_2070203+2	17	7	-35	TCACGCGACG	-14	GGTATCTTGT	-143	4,1	5.40	7.60	s	1302	Rmet_1902	0	Op0555f_1	NA
5531	TSS_2378033+2	17	4	-48	CTGAGCTAAG	-16	TTTACAATAC	-144	1,1	5.31	-11.60	no	1843	Rmet_R0034	0	Op0625f_1	NA
5532	TSS_2457883+2	17	4	-36	TATCGGTGCC	-13	TGTAAGAAAG	-150	2,1	5.61	5.40	m	425	Rmet_2244	0	Op0643f_1	NA
5533	TSS_2665731+2	17	4	-38	TTCCAGACGC	-14	GCAACAATGG	-139	1,1	7.11	5.90	m	958	Rmet_2455	*rpsU1*	Op0685f_3	GIP-TL-Ribo
5534	TSS_2700799+2	17	6	-36	TTGAAGTTGG	-3	AGCACCGTGG	-142	1,1	5.15	-0.60	no	941	Rmet_2489	*mdh*	Op0689f_1	MET-CAH-TCA
5535	TSS_3245219+2	17	5	-43	AAAGGTCTTA	-23	GGTTAGTCCA	-151	3,0	3.63	-8.20	no	8457	Rmet_2996	*ptxR*	Op0843f_1	NA
5536	TSS_3529073+2	17	5	-43	TGGCATGACC	-14	CGTACCGTCG	-140	1,1	4.82	-5.10	no	2296	Rmet_3261	0	Op0919f_1	NA
5537	TSS_3427322-2	17	1	-41	TTCACAAATT	-15	GGCAAGCAAT	-148	1,1	0.34	-0.10	no	12	Rmet_3161	0	Op0886r_2	NA
5538	TSS_3428656-2	17	1	-46	TCAGCGAAGT	-26	GTGATGATGC	-143	4,1	6.85	-2.20	no	970	Rmet_R0050	0	Op0886r_1	NA
5539	TSS_3431395-2	17	3	-32	TCGTAGTCTG	-10	GTGAAGCTGG	-145	1,1	3.48	4.70	m	266	Rmet_R0053	0	Op0886r_1	NA
5540	TSS_3432782-2	17	1	-49	TCGCACAGTA	-18	CGGAGCCTGG	-147	1,1	3.36	-14.10	no	62	Rmet_R0054	0	Op0886r_1	NA
5541	TSS_3506441-2	17	2	-47	TCCAAGTACT	-23	TTCAACTTCT	-146	1,1	1.97	-5.60	no	1303	Rmet_3228	*petC*	Op0914r_1	MET-EN-OxPhos
5542	TSS_3642255-2	17	4	-40	TTGCTCCGAA	-13	GTTACTCTGG	-141	1,1	7.43	1.40	*(w)-sba*	45	Rmet_3366	0	Op0936r_1	GIP-REP-Recomb

5543	TSS_667+3	17	1	-24	GCAGAAACGG	-3	GTCAAATTCT	-144	4,1	7.84	-6.40	no	166	Rmet_6605	0	Op1756f_1	NA	
5544	TSS_150671+3	17	1	-23	GCATGTGGTG	-3	CGATTACAGG	-149	3,0	2.35	-12.40	no	#NV	#NV	#NV	#NV	#NV	#NV
5545	TSS_1334126+3	17	2	-47	TTGACGCCAA	-14	GGCATCATCA	-140	1,1	7.68	-8.60	no	22603	Rmet_4714	0	Op1360f_1	NA	
5546	TSS_1367178+3	17	2	-36	TTGCAGTTCG	-15	ATCATCATCA	-141	1,1	6.80	10.80	s	769	Rmet_4725	0	Op1364f_1	NA	
5547	TSS_1765110+3	17	3	-43	AGCAGCGCTG	-23	CGCAACGGCT	-148	3,0	0.67	-6.20	no	1269	Rmet_5079	0	Op1496f_1	NA	
5548	TSS_1910182+3	17	4	-36	TCGCATTGCC	-14	CGGATAATGC	-139	1,1	7.27	10.90	s	2528	Rmet_5218	0	Op1538f_1	NA	
5549	TSS_2460295+3	17	1	-46	GTACCAGCAG	-14	TGCATGATTT	-144	4,1	6.92	-11.10	no	2231	Rmet_5704	0	Op1706f_1	NA	
5550	TSS_156954-3	17	4	-46	TGGCAAGGGT	-18	TTTACGGTAT	-145	1,1	5.37	-6.10	no	1645	Rmet_6616	0	Op1007r_1	NA	
5551	TSS_450294-3	17	3	-47	TTGTCGTAGA	-13	GGCAGTATGG	-141	1,1	6.85	-11.60	no	1121	Rmet_3903	0	Op1091r_1	NA	
5552	TSS_940701-3	17	1	-36	TTGCGCTTGG	-14	GTCAGCATTG	-141	1,1	7.08	10.40	s	275	Rmet_4343	0	Op1235r_1	NA	
5553	TSS_1878344-3	17	1	-39	TTGAATGCCA	-13	GTTAGTCTCT	-142	1,1	6.68	4.40	m	190	Rmet_5184	0	Op1527r_1	NA	
5554	TSS_2056489-3	17	4	-37	GGCGAGACCT	-14	TTTACCATGG	-139	4,1	7.82	3.40	m	3525	Rmet_6719	0	Op1583r_2	NA	
5555	TSS_2136296-3	17	2	-43	TGGAAGCTGT	-23	TTCAGGTTCC	-143	1,1	3.56	0.30	w	2103	Rmet_5403	0	Op1609r_1	NA	
5556	TSS_4870+4	17	3	-47	TGGCAGAGTG	-14	CGTAATATCT	-144	1,1	6.99	-12.10	no	872	Rmet_6347	0	Op1845f_1	NA	
5557	TSS_9287-4	17	3	-31	TTGCTGCTGA	-1	CCGACGATGA	-142	1,1	5.48	-1.80	no	4464	Rmet_6060	repA	Op1846r_1	NA	
5558	TSS_46867-4	17	6	-34	TCGAGCCGTT	-13	AGGACAATAT	-140	1,1	5.65	9.10	s	8252	Rmet_6359	0	Op2108r_1	NA	
5559	TSS_79420-4	17	5	-40	TCGACCAGAC	-13	GTTACGATCT	-140	1,1	8.20	2.90	(w)-sba	5460	Rmet_5985	czcM	Op1822r_1	EIP-TRA-Other	
5560	TSS_85892-4	17	5	-34	TTCAGTGGAG	-13	GCTAGACTGT	-141	1,1	7.78	9.10	s	47	Rmet_5975	czcJ	Op1818r_2	NA	
5561	TSS_143572-4	17	3	-34	TCGACAACCA	-13	CACACAATAC	-138	1,1	6.43	11.10	s	457	Rmet_6155	0	Op1882r_1	NA	
5562	TSS_206957-4	17	2	-38	TTTCTTCGGC	-13	GGCATTCTGC	-140	2,1	9.97	3.90	m	3039	Rmet_6096	0	Op1858r_1	NA	
5563	TSS_115483-5	17	3	-34	TTGTCACGTC	-12	AGTAACCTGA	-143	1,1	6.64	11.20	s	863	Rmet_6260	tnpA	Op1907r_1	NA	
5564	TSS_270053+2	17	0	-38	TTCCCGGAGC	-14	GTTATGGTTC	-141	1,1	6.88	6.90	s	668	Rmet_0253	0	Op0073f_2	GIP-REP-Recomb	
5565	TSS_310607+2	17	1	-36	TCGTGTGCGC	-13	TACAAAATGC	-145	1,1	5.54	8.90	s	60	Rmet_0294	0	Op0085f_2	NA	
5566	TSS_803078+2	17	4	-41	GCAACGCCCC	-17	ATTACTATTC	-142	4,1	7.44	0.40	(w)-sba	5	Rmet_0726	ugd	Op0205f_2	MET-CAH-Pent	
5567	TSS_1792785+2	17	4	-35	GCCCCGATGT	-15	GATAAGCTGT	-141	4,1	6.93	3.60	m	3214	Rmet_1661	0	Op0469f_1	NA	
5568	TSS_2159073+2	17	3	-38	TATCTTCGGT	-13	ATTATCCTGG	-142	2,1	8.48	3.40	m	1116	Rmet_6495	0	Op0579f_1	NA	
5569	TSS_2350931+2	17	2	-40	TGGATGACCA	-14	GGTATCGTCC	-144	1,1	6.02	2.90	(w)-sba	594	Rmet_2154	evgS	Op0621f_2	EIP-SIG-2Comp	
5570	TSS_2368535+2	17	3	-35	TTGAGAAGCT	-15	GCTAAGGTGG	-142	1,1	8.05	10.60	s	145	Rmet_2166	0	Op0623f_1	NA	
5571	TSS_2492294+2	17	2	-40	TCGACGATCG	-14	GATATTCTTG	-140	1,1	6.97	3.90	(m)-sba	1517	Rmet_2271	0	Op0649f_1	NA	
5572	TSS_2595939+2	17	2	-41	TTGTCGGCAG	-14	AGTATCATCA	-138	1,1	7.52	3.40	(m)-sba	1663	Rmet_2385	ccmA	Op0673f_1	MET-EN-Nitrog	
5573	TSS_2769909+2	17	5	-38	TTCAAACACC	-16	GGTAGAGTAA	-145	1,1	6.70	9.90	s	71	Rmet_2545	0	Op0705f_1	NA	
5574	TSS_2779328+2	17	2	-39	CTCACATGGC	-14	GATAGCATTG	-144	1,1	3.71	3.90	m	2230	Rmet_6537	0	Op2036f_1	NA	
5575	TSS_2963564+2	17	1	-37	TAGCCAAATA	-14	CTTAAAATGC	-144	1,1	6.54	10.90	s	8113	Rmet_2736	apaH	Op0771f_1	MET-NUC-Pur	
5576	TSS_3263105+2	17	3	-36	TCAACCAGTC	-15	TCTACAGTAA	-147	5,1	5.02	9.80	s	8951	Rmet_3013	yodB	Op0847f_1	NA	
5577	TSS_3592926+2	17	4	-41	GCGTCCCGCG	-15	AGTACCCTTC	-140	5,1	6.91	-2.10	no	1187	Rmet_3329	0	Op0929f_1	NA	
5578	TSS_3636891+2	17	1	-39	TCGCCGTCAC	-13	GCCACAATGA	-140	1,1	7.77	3.90	m	970	Rmet_3362	0	Op0935f_3	NA	
5579	TSS_547414-2	17	4	-43	TGGTCACGAT	-5	AGCACGATCC	-143	1,1	5.47	-12.10	no	521	Rmet_0512	mdtC	Op0138r_1	EIP-TRA-Ion	
5580	TSS_812677-2	17	5	-35	TTGATCACTT	-14	GTGATCATCA	-139	1,1	6.71	11.60	s	1719	Rmet_0732	mltB	Op0206r_1	NA	
5581	TSS_1600115-2	17	1	-39	TCGTGAGGTG	-14	TTTACATTGA	-145	1,1	5.57	4.90	m	3675	Rmet_1476	0	Op0414r_1	NA	
5582	TSS_1668701-2	17	0	-44	TTGCTCGATA	-14	GGCAAAATGC	-140	1,1	8.94	-4.60	no	113	Rmet_1545	0	Op0430r_1	NA	
5583	TSS_1773273-2	17	4	-38	CCTGACGGCC	-13	CATACACTGC	-138	5,1	9.66	0.40	w	1043	Rmet_1639	0	Op0462r_3	NA	
5584	TSS_2189928-2	17	4	-45	TTCAGGGGAC	-14	CGTAATATCG	-142	1,1	6.93	-7.10	no	2868	Rmet_2022	0	Op0590r_1	MET-CAH-Prop	
5585	TSS_2353394-2	17	2	-37	TCGACGTCCG	-13	AGCAGAATGG	-140	1,1	6.58	8.90	s	11402	Rmet_2148	0	Op0620r_1	NA	
5586	TSS_1648734+3	17	3	-41	TCGTCCTGCG	-15	GCCAATATCC	-139	1,1	5.07	0.90	(w)-sba	203	Rmet_4972	tniA	Op1460f_1	GIP-REP-Recomb	
5587	TSS_1917698+3	17	2	-44	GCGGCCGGAC	-14	AGTATCCTGC	-139	5,1	9.83	-8.10	no	59	Rmet_5223	chrF3	Op1540f_2	NA	
5588	TSS_806405-3	17	5	-43	TTCCCAGGCA	-14	GGTACTTTCC	-141	1,1	6.27	-3.10	no	3263	Rmet_4218	0	Op1197r_1	NA	
5589	TSS_820631-3	17	2	-39	TCGAGACGAA	-13	GCTATCATGT	-136	1,1	8.57	5.90	m	3336	Rmet_4231	0	Op1199r_1	NA	
5590	TSS_1119871-3	17	5	-46	TTGACGCCTT	-7	AATACGATAT	-147	1,1	7.62	-13.60	no	233	Rmet_4514	int	Op1289r_1	NA	
5591	TSS_1762951-3	17	4	-32	TTGATTGAGA	-11	AATATCATCG	-144	1,1	7.85	9.60	s	707	Rmet_5073	metE	Op1493r_1	NA	
5592	TSS_1821762-3	17	2	-35	TTGCAGAGGAC	-13	GAGATCATCG	-142	1,1	4.98	10.20	s	2415	Rmet_5124	0	Op1509r_1	NA	
5593	TSS_2499632-3	17	2	-46	TTGATGCTGG	-14	GGGATAATCA	-141	1,1	8.50	-6.60	no	6825	Rmet_5732	kpsM	Op1715r_1	NA	
5594	TSS_988+4	17	3	-41	TTCCCGGTAA	-14	TGTACCATTC	-139	1,1	7.81	0.90	(w)-sba	133	Rmet_6062	parB	Op1847f_1	NA	
5595	TSS_225582+4	17	3	-48	TGGACACTGC	-15	GGCAGACTTA	-140	1,1	6.96	-11.10	no	112	Rmet_6076	0	Op1851f_1	NA	
5596	TSS_41448-4	17	2	-38	TCGCCGCGAT	-14	CGTACAATTT	-138	1,1	8.98	7.90	s	2833	Rmet_6359	0	Op2108r_1	NA	
5597	TSS_99781-5	17	3	-35	TCGACCAGGT	-13	CGTAGATTTC	-140	1,1	7.15	11.70	s	5311	Rmet_6246	0	Op1903r_1	NA	
5598	TSS_159232+2	17	2	-43	TGGCCCGCAC	-14	GGGATCATGA	-141	1,1	6.25	-3.10	no	1277	Rmet_0156	metK	Op0045f_1	MET-AA-Met	
5599	TSS_508267+2	17	2	-38	TCGTAGAGGG	-14	GTCAGAATTT	-141	1,1	6.46	5.90	m	1320	Rmet_0481	0	Op0127f_1	NA	
5600	TSS_565678+2	17	3	-36	TTGCCCGGGC	-15	CGTACTGTAG	-141	1,1	6.01	10.80	s	51	Rmet_0530	0	Op0147f_1	NA	
5601	TSS_1140608+2	17	2	-36	TCGTCGTGCT	-8	CTCAGCCTAT	-145	1,1	2.23	2.90	(w)-ba	861	Rmet_1048	0	Op0301f_1	NA	
5602	TSS_1258503+2	17	1	-35	TTGCGTCTGT	-13	GGTACTGTCG	-140	1,1	6.91	10.20	s	468	Rmet_1148	0	Op0329f_1	NA	
5603	TSS_1314484+2	17	2	-43	TTCCACGCTG	-15	CGTACGATCC	-141	1,1	7.08	-3.10	no	883	Rmet_1197	aceF	Op0347f_2	MET-CAH-FbP	

5604	TSS_1593557+2	17	4	-25	TTGCGCGTGG	-5	GCCAGGCTTG	-142	1,1	5.92	-2.40	no	2094	Rmet_1475	0	Op0413f_1	NA
5605	TSS_2556863+2	17	1	-39	TGGCAATTCC	-13	GCAATGATGC	-140	1,1	6.65	3.90	m	2540	Rmet_2345	0	Op0663f_1	NA
5606	TSS_3638978+2	17	2	-41	TTGCAATTTC	-15	GCAACAATAG	-141	1,1	8.21	3.40	(m)-sba	6819	Rmet_3369	paaA	Op0937f_1	NA
5607	TSS_208619-2	17	2	-40	GCGAACGCCT	-13	TTCATGATGT	-140	5,1	5.79	-1.10	no	221	Rmet_0199	0	Op0058r_1	NA
5608	TSS_588998-2	17	6	-35	TCGCCGTCAT	-13	GGTATCGTAG	-141	1,1	6.17	10.70	s	5267	Rmet_0547	0	Op0152r_1	NA
5609	TSS_1424685-2	17	2	-40	TTGCACTGGT	-14	GGTATGATGA	-136	1,1	10.65	4.40	(m)-sba	570	Rmet_1307	0	Op0376r_5	EIP-TRA-Pores
5610	TSS_1428731-2	17	2	-34	TCGCTGGATC	-13	GGTAAGCTGG	-142	1,1	6.48	7.10	s	724	Rmet_1311	0	Op0376r_5	NA
5611	TSS_1798816-2	17	3	-45	GTAACCAACG	-25	AGTACGCTTT	-146	4,1	5.79	-2.70	no	564	Rmet_1663	papD	Op0468r_2	NA
5612	TSS_2184217-2	17	4	-39	TTGCCAAGAA	-10	TCCACATTTC	-143	1,1	7.44	2.40	w	406	Rmet_2018	0	Op0588r_1	NA
5613	TSS_2190647-2	17	4	-38	TCCTCTATTC	-13	GCTATCCTGT	-145	1,1	5.21	4.40	m	689	Rmet_2024	bipA	Op0592r_1	NA
5614	TSS_2973642-2	17	2	-26	TTGCCTTGAT	-2	GCCACTATCA	-141	1,1	7.37	0.20	no	2167	Rmet_2735	rfbB	Op0770r_1	MET-CAH-NucSug
5615	TSS_3430086-2	17	2	-33	TTGTATAATG	-2	GGCAAGCTTA	-145	1,1	5.32	-1.80	no	#NV	#NV	#NV	#NV	#NV
5616	TSS_3715420-2	17	3	-37	TTCCCATAAT	-13	ATTAATCTTC	-145	1,1	6.64	7.90	s	28	Rmet_3440	0	Op0956r_1	NA
5617	TSS_301529+3	17	4	-41	GCCACAATGA	-14	GCTAGCATTC	-141	4,1	7.97	-1.60	no	5142	Rmet_3770	tnpA	Op1048f_1	NA
5618	TSS_310827+3	17	4	-39	TTCAGAGCAG	-15	GGCAGAATGG	-139	1,1	7.78	6.90	s	1076	Rmet_3776	0	Op1050f_1	NA
5619	TSS_1380104+3	17	2	-41	TTGACCGTGG	-14	GCGAGAATCC	-139	1,1	8.58	3.40	(m)-sba	481	Rmet_4736	kdgD	Op1370f_1	MET-CAH-Ascorb
5620	TSS_2259228+3	17	4	-36	TTGCGGAGAC	-15	GAGATGATTG	-139	1,1	7.60	10.80	s	12350	Rmet_5525	0	Op1642f_1	NA
5621	TSS_2421448+3	17	3	-36	GTAAAGGAAT	-8	TCGATCTTGG	-145	4,1	5.46	0.90	(w)-ba	450	Rmet_5662	0	Op1692f_1	NA
5622	TSS_2474024+3	17	4	-34	TGCAGTGATA	-2	CCAATTCTCT	-150	1,1	1.28	-2.80	no	373	Rmet_5716	0	Op1710f_1	NA
5623	TSS_2572452+3	17	4	-37	GCACCGAAGA	-14	CGAATAATAG	-145	4,1	6.18	5.40	m	968	Rmet_5810	0	Op1752f_1	NA
5624	TSS_2295307-3	17	4	-21	TCGCCAAGGA	-1	GTAAGGCTGA	-143	1,1	3.60	-5.90	no	483	Rmet_6743	0	Op1647r_1	NA
5625	TSS_40690+4	17	2	-48	TGGCGTAGGT	-14	CGTATCCTCT	-142	1,1	6.64	-14.10	no	81	Rmet_6017	0	Op1835f_1	NA
5626	TSS_73195+4	17	5	-35	TTGCACGTTC	-4	TCTATTTTGC	-144	1,1	7.34	2.20	(w)-ba	844	Rmet_5984	czcN	Op1821f_1	NA
5627	TSS_73205+4	17	4	-45	TTGCACGTTC	-14	TCTATTTTGC	-143	1,1	7.34	-6.60	no	834	Rmet_5984	czcN	Op1821f_1	NA
5628	TSS_100664+4	17	3	-35	TGGACGCGAC	-14	CGTACATTGC	-140	1,1	7.59	11.10	s	689	Rmet_5960	int	Op1813f_2	NA
5629	TSS_177966-4	17	3	-40	TCGGCACGGG	-14	GGTAGAATGA	-136	5,1	11.87	4.90	(m)-sba	7548	Rmet_6133	silD	Op1874r_1	NA
5630	TSS_16704-5	17	4	-40	TTGACCCAAT	-14	CGTATGATTT	-138	1,1	9.64	6.40	(s)-sba	370	Rmet_6326	0	Op1921r_1	NA
5631	TSS_83032+2	16	2	-35	TGCCGAGGGT	-14	TTCAAAATGA	-139	1,1	6.08	8.60	s	6159	Rmet_0082	0	Op0023f_1	NA
5632	TSS_137196+2	16	4	-40	TGGCCTTTTT	-10	GATAGGGATG	-146	1,1	0.35	-4.10	no	111	Rmet_0131	hslV	Op0033f_2	GIP-PTL-Fold
5633	TSS_825029+2	16	3	-47	TTGTCGAGCC	-13	CATACAATGC	-136	1,1	9.60	-9.60	no	420	Rmet_0748	rpsP	Op0213f_1	GIP-TL-Ribo
5634	TSS_1485147+2	16	2	-36	TTCTGTTTAT	-15	TGCAAAATTT	-145	1,1	6.51	9.30	s	173	Rmet_1376	cysU	Op0385f_3	EIP-TRA-ABC
5635	TSS_2361023+2	16	3	-48	GCCGACAAAG	-14	CGTACGATAC	-143	4,1	7.87	-18.60	no	2671	Rmet_2163	0	Op0623f_1	NA
5636	TSS_2500361+2	16	3	-48	TCGTCCATCA	-16	GGTAAAGTTA	-140	1,1	6.69	-11.10	no	174	Rmet_2276	0	Op0651f_1	NA
5637	TSS_2701536+2	16	3	-43	TTGCGGGATG	-14	AGTAGACTTA	-139	1,1	7.83	-2.60	no	204	Rmet_2489	mdh	Op0689f_1	MET-CAH-TCA
5638	TSS_3093223+2	16	3	-37	TTCCCACCGA	-15	GCGAATATCA	-142	1,1	5.21	9.90	s	581	Rmet_2844	0	Op0801f_2	NA
5639	TSS_3685184+2	16	3	-46	GCCAGGGAAT	-26	GGAACTATCG	-141	4,1	6.12	-6.20	no	325	Rmet_3407	0	Op0947f_1	MET-LIP-Others
5640	TSS_3731504+2	16	2	-33	GAACTTGCTG	-13	CGTCGGTATA	-143	3,0	6.88	-3.40	no	226	Rmet_6596	0	Op2055f_1	NA
5641	TSS_827375-2	16	5	-46	TCAGACGCGC	-14	TCGATGATGT	-144	4,1	6.74	-11.60	no	2023	Rmet_0747	0	Op0212r_1	NA
5642	TSS_2090631-2	16	1	-34	TTGATCTATC	-12	CGAATACTCA	-144	1,1	5.95	11.20	s	47	Rmet_1923	0	Op0560r_1	GIP-PTL-Fold
5643	TSS_2141454-2	16	2	-42	GCGACAACAC	-13	CGTACACTGG	-139	5,1	8.44	-3.10	no	1288	Rmet_1964	moeA	Op0574r_1	MET-COF-Folate
5644	TSS_2228631-2	16	4	-38	TTGCGGCGCA	-13	GCGAGAATCG	-142	1,1	7.73	6.40	s	1056	Rmet_2055	ugpB	Op0596r_1	EIP-TRA-ABC
5645	TSS_2419197-2	16	4	-41	TGGAAGGACG	-13	GGGACAATCC	-138	1,1	7.65	-0.10	no	115	Rmet_2205	0	Op0630r_1	NA
5646	TSS_2606215-2	16	2	-41	TGGAAATCAT	-14	CGTACTCTTC	-140	1,1	7.02	0.90	(w)-sba	8781	Rmet_2384	armR	Op0672r_1	NA
5647	TSS_3250523-2	16	2	-40	TTCAGGGTGA	-13	GCTACTCTGG	-139	1,1	6.86	0.90	(w)-sba	1159	Rmet_2991	tnpAB	Op0842r_2	NA
5648	TSS_3710540-2	16	2	-39	TTAAGCACCT	-13	AACAAAATCG	-147	4,1	5.51	3.90	m	36	Rmet_3432	0	Op0952r_1	NA
5649	TSS_3854069-2	16	2	-43	CCAGCAGGTC	-14	GAGAGAATCG	-140	5,1	8.40	-6.60	no	5806	Rmet_3548	0	Op0984r_1	NA
5650	TSS_176363+3	16	2	-34	TCGACAGACT	-13	TGGACAATAA	-143	1,1	6.46	11.10	s	783	Rmet_3643	0	Op1016f_1	NA
5651	TSS_1937964+3	16	4	-35	TTGACAAACA	-14	ACGAACCTTG	-142	1,1	5.51	11.60	s	763	Rmet_5240	0	Op1544f_1	NA
5652	TSS_2033338+3	16	3	-37	TGCCCCTACA	-15	CAGACAATAC	-143	1,1	4.68	8.40	s	1215	Rmet_5325	zneP	Op1576f_1	NA
5653	TSS_104626-3	16	1	-34	TGGAACTCGT	-2	ATGACGCTGA	-145	1,1	3.94	-2.30	no	2442	Rmet_5901	0	Op1789r_1	NA
5654	TSS_290965-3	16	2	-41	AATCGTCGCG	-13	GGCATGATTG	-141	2,1	10.76	-5.60	no	114	Rmet_6621	0	Op1043r_1	NA
5655	TSS_1728729-3	16	4	-41	TTGCCCTGGC	-17	TGCATCATGC	-138	1,1	8.50	5.40	(m)-sba	1616	Rmet_5039	0	Op1481r_1	MET-CAH-But
5656	TSS_204540+4	16	1	-47	TTGATACGAC	-15	GTGAGAATCC	-144	1,1	8.18	-7.60	no	184	Rmet_6095	0	Op1857f_1	NA
5657	TSS_31270+5	16	1	-31	GAACCTGCCA	-11	ACCCTGTAGT	-147	3,0	4.71	-4.40	no	461	Rmet_6345	merT	Op1894f_1	NA
5658	TSS_111601+5	16	4	-38	GCAGCACGGG	-14	GGCAATATGA	-141	5,1	8.14	2.40	w	3448	Rmet_6261	rhsB	Op1908f_1	NA
5659	TSS_4705-5	16	1	-34	TTGCGCTTCG	-13	AATACTGTTT	-146	1,1	5.36	8.60	s	18805	Rmet_6301	trbF	Op1917r_1	NA
5660	TSS_284248+2	16	2	-40	TTCCGCGGAG	-14	GTCAGAATAT	-138	1,1	6.36	1.90	(w)-sba	222	Rmet_0268	0	Op0077f_1	NA
5661	TSS_928073+2	16	3	-41	TCGAGCAATG	-15	GGCAATATCA	-145	1,1	5.35	0.90	(w)-sba	2086	Rmet_0849	0	Op0243f_1	NA
5662	TSS_1355416+2	16	3	-35	TTGCGATAGC	-14	GGCACCGTGG	-139	1,1	6.34	9.60	s	284	Rmet_1233	0	Op0357f_1	NA
5663	TSS_1469444+2	16	1	-42	TTGACGCGTC	-18	GGCATGGTCC	-142	1,1	6.46	4.40	(m)-sba	614	Rmet_1361	0	Op0383f_3	NA
5664	TSS_1739861+2	16	3	-42	TCGTCCAGAC	-14	GCTAAAGTCG	-141	1,1	6.77	-1.10	no	721	Rmet_1614	0	Op0451f_1	EIP-TRA-ABC

5665	TSS_3261345+2	16	3	-46	TGGCCATCCT	-15	CGTATTCTCG	-141	1,1	7.09	-7.10	no	1270	Rmet_3006	0	Op0845f_1	NA
5666	TSS_3272142+2	16	4	-37	TTGCACTGGC	-16	CTCATCATTG	-142	1,1	7.62	9.80	s	507	Rmet_3014	0	Op0847f_1	NA
5667	TSS_3305892+2	16	2	-45	TCAAGGGCCC	-14	CGTAATGTTC	-143	4,1	5.29	-9.60	no	101	Rmet_3049	0	Op0857f_1	NA
5668	TSS_3922157+2	16	1	-46	TTGCCCACAT	-15	CCGACAATCC	-138	1,1	8.25	-6.60	no	6035	Rmet_0001	*dnaA*	Op0001f_1	GIP-REP-Complex
5669	TSS_823290-2	16	2	-39	TTCATGCTGC	-8	GCGAAACACA	-149	1,1	0.09	-4.10	no	134	Rmet_0744	0	Op0210r_1	NA
5670	TSS_2048228-2	16	5	-41	TCGAGAAGGT	-14	AGTACCTTGC	-141	1,1	5.99	0.90	(w)-sba	1638	Rmet_R0021	0	Op0544r_2	NA
5671	TSS_3513310-2	16	2	-34	TCAGGAGCAA	-12	TTTAGCATTT	-143	4,1	6.47	8.20	s	313	Rmet_3236	*tatB*	Op0918r_3	GIP-PTL-Exp
5672	TSS_3517903-2	16	4	-35	TTGCTAATGG	-14	ATGAACATGC	-142	1,1	6.16	10.60	s	356	Rmet_3244	*hisH*	Op0918r_3	MET-AA-His
5673	TSS_3643049-2	16	2	-38	TCGAAGAGAT	-12	CGTACAGTCA	-144	1,1	6.70	4.90	m	142	Rmet_3367	0	Op0936r_1	NA
5674	TSS_3645089-2	16	3	-40	TTGATTTTGT	-11	CGTATATTGG	-140	1,1	8.92	2.40	(w)-sba	17	Rmet_3368	0	Op0936r_1	NA
5675	TSS_151240+3	16	3	-45	TTGTATAATG	-14	GGCAAGCTTA	-145	1,1	5.32	-8.60	no	#NV	#NV	#NV	#NV	#NV
5676	TSS_705832+3	16	4	-37	TGGCCACCTT	-14	CCGATCATCG	-139	1,1	5.71	9.90	s	4172	Rmet_6649	0	Op1166f_1	NA
5677	TSS_823940+3	16	2	-48	GCACCAACGT	-14	TCCAGAATAA	-144	4,1	8.16	-16.60	no	4882	Rmet_4241	0	Op1202f_1	MET-CAH-TCA
5678	TSS_1414169-3	16	3	-34	TGGACAGCCA	-13	TCGACATTGT	-144	1,1	5.02	10.10	s	69	Rmet_4769	*kdgK*	Op1387r_1	MET-CAH-PP_KDPG
5679	TSS_1755192-3	16	1	-35	TGGCGATCTG	-12	TCTACAATAG	-136	1,1	8.75	10.70	s	162	Rmet_5065	0	Op1491r_2	NA
5680	TSS_2062947-3	16	4	-32	TTCCTTTCGC	-12	CGTAGTATTT	-142	1,1	5.98	5.10	m	317	Rmet_5341	0	Op1583r_1	NA
5681	TSS_2246377-3	16	3	-42	GCTGACGGAG	-13	GCTACACTGC	-136	5,1	10.96	-7.60	no	3723	Rmet_5498	0	Op1635r_1	NA
5682	TSS_39961+4	16	2	-35	TTGCCGAAGG	-14	CCGATCATCA	-139	1,1	6.56	11.60	s	810	Rmet_6017	0	Op1835f_1	NA
5683	TSS_228210-4	16	3	-36	TTGCTCCGGA	-13	AGGATAGTGT	-143	1,1	5.68	10.40	s	233	Rmet_6073	*tnpA*	Op1850r_1	NA
5684	TSS_30806+5	16	2	-47	TTCAACGGTC	-14	CGCAAAATTG	-143	1,1	7.34	-11.10	no	57	Rmet_6343	*tnpR*	Op1928f_1	NA
5685	TSS_49695+5	16	1	-42	GTCACCCAGG	-14	TGCAGAATCA	-145	4,1	5.24	-3.10	no	2605	Rmet_6204	*chrI*	Op1896f_1	NA
5686	TSS_1793072+2	16	3	-34	TTGACCGGGT	-13	GATACAAAAC	-141	1,1	5.63	10.60	s	2927	Rmet_1661	0	Op0469f_1	NA
5687	TSS_2368325+2	16	1	-35	TTGAAGTCGC	-3	GGCAGCATAG	-140	1,1	7.12	1.20	(w)-ba	355	Rmet_2166	0	Op0623f_1	NA
5688	TSS_2956830+2	16	5	-32	TGGCAAAACC	-10	TACAGACTGC	-142	1,1	6.01	6.70	s	100	Rmet_2722	0	Op0769f_2	NA
5689	TSS_3070257+2	16	2	-49	TTCAGATCGG	-3	CGTACAAACC	-144	1,1	4.06	-26.10	no	732	Rmet_2823	0	Op0795f_1	NA
5690	TSS_3570508+2	16	3	-35	TCGACAGTCA	-14	GGAAGAATCC	-142	1,1	6.51	12.10	s	15165	Rmet_3322	0	Op0927f_1	NA
5691	TSS_3593161+2	16	2	-38	TCGACCTTCT	-14	TAGACAATGC	-138	1,1	7.09	7.90	s	952	Rmet_3329	0	Op0929f_1	NA
5692	TSS_3869619+2	16	1	-41	TTGCCGAACC	-14	CGTATCATTG	-135	1,1	9.53	3.40	(m)-sba	462	Rmet_3568	0	Op0989f_1	NA
5693	TSS_829229-2	16	3	-42	GCGGACATCG	-14	CAGAAAATCA	-142	5,1	7.37	-5.10	no	3877	Rmet_0747	0	Op0212r_1	NA
5694	TSS_1141455-2	16	1	-47	TCCAGAACAG	-21	CTCAGCATGG	-142	4,1	5.43	-5.60	no	4693	Rmet_1044	*yebK*	Op0300r_1	NA
5695	TSS_1254596-2	16	3	-44	TGGCCCGGTA	-14	GGCATAGTGA	-139	1,1	6.29	-5.10	no	5253	Rmet_1141	0	Op0326r_1	NA
5696	TSS_1583662-2	16	1	-48	TTGCGAGCCG	-14	TGCATGATTG	-141	1,1	7.50	-11.60	no	2282	Rmet_1460	*ytfF*	Op0408r_1	NA
5697	TSS_3158875-2	16	1	-35	GGCGCGCCGA	-13	GCGATACTGC	-141	4,1	5.14	6.20	m	672	Rmet_2904	*rpsT*	Op0820r_1	GIP-TL-Ribo
5698	TSS_3166972-2	16	3	-39	GCACCGCACA	-13	TGTACAGTGA	-140	4,1	5.32	-0.60	no	542	Rmet_2911	*purM*	Op0824r_1	MET-NUC-Pur
5699	TSS_3448872-2	16	2	-37	TGGCGCCGGC	-14	GGGATGATGC	-138	1,1	6.84	7.90	s	392	Rmet_3175	*apaG*	Op0892r_1	NA
5700	TSS_3613512-2	16	1	-21	TGGAAGGGCC	0	GATAAAAGGT	-148	1,1	1.31	-4.90	no	593	Rmet_R0060	0	Op0930r_1	NA
5701	TSS_3747668-2	16	3	-35	GGACAGAAGT	-14	GGCATCCTCG	-141	4,1	7.60	3.60	m	84	Rmet_3467	0	Op0964r_2	NA
5702	TSS_2218877+3	16	1	-43	TCTGCAAGCG	-14	TTGACAATAC	-142	5,1	8.92	-3.60	no	1587	Rmet_5475	0	Op1626f_1	NA
5703	TSS_2399-3	16	1	-29	TTCCGTTCGG	-7	TTCAACATGC	-144	2,1	8.93	2.70	(w)-nu	53	Rmet_5818	*csp*	Op1757r_1	NA
5704	TSS_50705-3	16	2	-35	CCAGCCCGGC	-12	TGTATGTTTC	-140	5,1	8.30	5.20	m	654	Rmet_5854	0	Op1771r_3	NA
5705	TSS_67095-3	16	2	-25	TCGCCGACGC	0	CATACGTTGC	-144	1,1	5.19	-3.30	no	701	Rmet_5870	0	Op1777r_1	NA
5706	TSS_814618-3	16	2	-41	GCAGAACAGC	-13	AGTACACTGA	-141	4,1	10.03	-4.60	no	199	Rmet_4228	*tnpA*	Op1199r_2	NA
5707	TSS_905368-3	16	1	-46	GCGGACCGCC	-13	GACATCATGG	-141	5,1	8.85	-14.10	no	1729	Rmet_4308	0	Op1227r_1	NA
5708	TSS_1473530-3	16	3	-45	TTGCCGGATA	-13	TTTATCCTGT	-143	1,1	7.99	-6.60	no	10036	Rmet_4815	0	Op1403r_1	NA
5709	TSS_1933579-3	16	3	-37	TCGTCATACC	-13	AGTACGCTCC	-140	1,1	5.80	7.90	s	5482	Rmet_5230	*iscS2*	Op1543r_1	NA
5710	TSS_2324731-3	16	1	-35	TGCACGCACA	-13	GGTTCAATGC	-143	1,1	2.67	9.20	s	2438	Rmet_5568	0	Op1655r_1	NA
5711	TSS_2563375-3	16	2	-38	GCTGCACTGC	-14	GGTACGCTTT	-141	5,1	9.20	2.40	w	70	Rmet_5803	*mscS*	Op1749r_2	NA
5712	TSS_988843+2	15	2	-45	TGCTTATAAT	-21	TACAAAGTCG	-144	1,1	2.82	-2.60	no	706	Rmet_0902	0	Op0261f_2	NA
5713	TSS_3380213-2	15	1	-44	TCGCAGATGC	-13	CGTACAACGC	-146	1,1	1.24	-8.10	no	2404	Rmet_3117	*argJ*	Op0876r_2	MET-AA-Urea
5714	TSS_3588344-2	15	2	-34	TTCCATTAGG	-7	GGAAAAGTTC	-145	1,1	4.79	2.70	(w)-ba	1254	Rmet_3323	*rpsJ*	Op0928r_3	GIP-TL-Ribo
5715	TSS_828934+3	15	1	-35	TTCCTGATAC	-14	CATACGATGT	-144	1,1	6.76	9.10	s	794	Rmet_4242	*bug*	Op1202f_2	NA
5716	TSS_726327-3	15	2	-40	TTCTGCTTTC	-14	CAGATAATGT	-142	1,1	5.96	2.90	(w)-sba	671	Rmet_4149	*crtB*	Op1169r_3	MET-LIP-Ster
5717	TSS_743675-3	15	1	-39	GAACTTGTCG	-19	CAAGGTTGTA	-142	3,0	8.13	-5.20	no	14605	Rmet_4153	*tnpA*	Op1169r_1	NA
5718	TSS_940557-3	15	2	-46	TTGTTCTGGC	-13	GACAAAATTC	-142	1,1	8.09	-9.60	no	131	Rmet_4343	0	Op1235r_1	NA
5719	TSS_74100+4	15	2	-41	TGGCCTGGGG	-14	ATTACATTTC	-144	1,1	6.70	0.90	(w)-sba	790	Rmet_6368	0	Op1821f_1	NA
5720	TSS_175581-4	15	1	-46	TCGACATATT	-20	ACCAAGTTCG	-142	1,1	3.92	-3.10	no	5163	Rmet_6133	*silD*	Op1874r_1	NA
5721	TSS_46003+5	15	2	-45	TCCCAACGGA	-13	GCTATCTTGT	-143	1,1	5.35	-9.60	no	6297	Rmet_6204	*chrI*	Op1896f_1	NA
5722	TSS_111134+5	15	3	-42	TGGAACGCTT	-14	TCTAGAGTTT	-146	1,1	6.52	-1.10	no	3915	Rmet_6261	*rhsB*	Op1908f_1	NA
5723	TSS_745868+2	15	2	-45	TTGCGTCAGC	-15	GCGATGCTTG	-140	1,1	6.33	-6.60	no	4417	Rmet_0678	*ppa*	Op0189f_1	MET-EN-OxPhos
5724	TSS_1016700+2	15	0	-48	TGGAAGTCGG	-25	CTGAAGATGG	-141	1,1	5.21	-3.10	no	581	Rmet_0933	*nuoG*	Op0267f_3	MET-EN-OxPhos
5725	TSS_2875790+2	15	1	-48	GGACAAACGC	-24	CCTACAATAG	-138	4,1	11.42	-6.60	no	34	Rmet_2640	0	Op0745f_1	NA

5726	TSS_3038752+2	15	1	-29	TTGCACGCAC	-6	CAGACGTTTG	-146	1,1	3.79	2.20	*(w)-nu*	297	Rmet_2792	*0*	Op0785f_1	NA
5727	TSS_3159012+2	15	0	-36	TTCATGACGC	-14	CGTACATTGG	-139	1,1	6.87	10.90	s	1143	Rmet_2906	*0*	Op0821f_2	NA
5728	TSS_3595682+2	15	3	-37	TGGCTTCACC	-14	GCGACAATGC	-138	1,1	6.94	7.90	s	22334	Rmet_6590	*0*	Op0931f_1	NA
5729	TSS_2667779-2	15	3	-35	TTGTCGACCA	-13	CATAGAGTTT	-139	1,1	6.12	12.20	s	195	Rmet_2456	*0*	Op0686r_1	NA
5730	TSS_3367696-2	15	2	-38	TTGCGCGCGC	-12	GTCATACTCG	-136	1,1	8.16	5.40	m	2375	Rmet_3106	*rplU*	Op0874r_1	GIP-TL-Ribo
5731	TSS_149108+3	15	0	-48	TGGATGACGG	-18	GCTAACTACG	-149	1,1	0.18	-12.10	*no*	1077	Rmet_R0071	*tRNA-l*	Op1006f_1	NA
5732	TSS_477728+3	15	3	-35	TCCCAAGGGT	-11	TTTAAAGAGG	-147	1,1	0.71	5.20	m	450	Rmet_R0079	*0*	Op1098f_1	NA
5733	TSS_906792+3	15	1	-36	TGGCTTGGTC	-14	GGAACAATGG	-138	1,1	6.35	9.90	s	7253	Rmet_4319	*0*	Op1230f_1	NA
5734	TSS_2265057+3	15	0	-38	GCACAGGAGT	-14	GTTACAGTGA	-141	4,1	9.16	1.40	w	6521	Rmet_5525	*0*	Op1642f_1	NA
5735	TSS_608193-3	15	3	-34	TTGAGTCAGT	-13	GTTATCACTT	-150	1,1	1.41	9.60	s	5772	Rmet_4033	*0*	Op1137r_1	NA
5736	TSS_758765-3	15	1	-37	TGGCGCGCTT	-15	CGTAAAATAA	-139	1,1	7.73	9.90	s	0	Rmet_4171	*0*	Op1175r_2	NA
5737	TSS_1189923-3	15	2	-40	TCGCGCAGCA	-13	AATACGATGG	-139	1,1	6.56	0.90	*(w)-sba*	2031	Rmet_4573	*0*	Op1315r_1	NA
5738	TSS_1367933-3	15	2	-47	TTGCAAGCAG	-13	GCGACATTCT	-142	1,1	6.57	-11.60	*no*	225	Rmet_4724	*cls*	Op1363r_1	MET-LIP-GlycPLip
5739	TSS_1625654-3	15	2	-41	GTACGCGCAT	-14	TGTACTATTG	-141	4,1	8.19	-3.10	*no*	4617	Rmet_4954	*0*	Op1455r_1	NA
5740	TSS_88154+4	15	2	-38	TCGCAAAGTA	-15	TTTAAAATAT	-142	1,1	8.49	8.90	s	528	Rmet_5971	*flgB*	Op1817f_1	DIV-MOT-Flagel
5741	TSS_19945+5	15	2	-40	TTGAGCCGCA	-15	CGTATCGTCA	-143	1,1	6.07	5.40	*(m)-sba*	1667	Rmet_6333	*tnpA*	Op1926f_1	NA
5742	TSS_497363+2	15	1	-31	GAAGTCGGCG	-11	ACCACTGATG	-145	3,0	4.99	-4.40	no	166	Rmet_0468	*0*	Op0123f_2	NA
5743	TSS_1136947+2	15	2	-38	TGCTCGGGAT	-14	CATAGAATTG	-141	1,1	6.35	6.40	s	113	Rmet_1045	*edd*	Op0301f_1	MET-CAH-PP_KDPG
5744	TSS_1898809+2	15	2	-43	TTGATGCCGC	-13	TCCACAATGC	-139	1,1	8.42	-2.60	*no*	1993	Rmet_1751	*agrR*	Op0499f_1	EIP-SIG-2Comp
5745	TSS_857000-2	15	2	-36	TGGTCGGTCA	-14	CGTATCATCG	-139	1,1	6.88	11.90	s	118	Rmet_0779	*maf*	Op0222r_2	DIV-Division
5746	TSS_1537245-2	15	1	-37	TCGAGCCGCT	-14	GTCATCATCG	-138	1,1	6.24	8.90	s	296	Rmet_1422	*0*	Op0398r_2	NA
5747	TSS_3612182-2	15	2	-32	TCGGCAGTTC	-9	GGCACCATCA	-142	5,1	7.65	6.70	(s)-ba	40	Rmet_3341	*tuf*	Op0930r_3	NA
5748	TSS_1559202+3	15	2	-36	TGGTGAGAAT	-15	GCTACACTGT	-143	1,1	8.30	10.30	s	85	Rmet_4895	*ygfH*	Op1432f_1	MET-CAH-Pyr
5749	TSS_501843-3	15	2	-42	TCGGACGGGT	-13	GCCATACTTG	-140	5,1	9.51	-3.10	*no*	122	Rmet_3943	*tnpA*	Op2061r_1	NA
5750	TSS_803204-3	15	1	-46	TCAGAAAATC	-22	AGTATAATCC	-146	4,1	9.08	-0.60	*no*	62	Rmet_4218	*0*	Op1197r_1	NA
5751	TSS_1272886-3	15	2	-36	TGGATCCCGT	-13	GTAAACCTTA	-148	1,1	2.89	7.90	s	214	Rmet_4641	*0*	Op1341r_2	NA
5752	TSS_2506610-3	15	1	-22	TTGTGGTCTG	0	GGTATCGAGC	-151	1,1	1.67	-3.80	*no*	759	Rmet_5743	*0*	Op1717r_1	MET-CAH-FbP
5753	TSS_33089+4	15	2	-45	TCGACAGGCT	-15	GGCACAATCC	-138	1,1	8.61	-4.10	*no*	1129	Rmet_6025	*0*	Op1835f_1	NA
5754	TSS_121709-4	15	2	-42	GTACGCGCCT	-14	GCGAGAATAA	-140	4,1	5.94	-5.10	*no*	152	Rmet_6181	*tnmB*	Op1892r_1	NA
5755	TSS_146322-4	15	1	-36	CCAAAACGGG	-13	GGTACTATCG	-141	5,1	7.23	6.40	s	405	Rmet_6151	*0*	Op1880r_2	NA
5756	TSS_68271+5	15	2	-48	TCAGGAGAGC	-15	GATAAAATCC	-140	4,1	10.82	-12.60	*no*	6980	Rmet_6223	*0*	Op1900f_1	NA
5757	TSS_1290011-3	14	2	-33	TTCCCTCTAC	-13	GAGACAATGT	-141	1,1	7.08	7.10	s	3202	Rmet_4654	*0*	Op1345r_1	NA
5758	TSS_1489378-3	14	1	-44	TTGTTACGAC	-13	GGAATAATTG	-145	1,1	7.78	-3.60	*no*	107	Rmet_4834	*ompP*	Op1409r_1	NA
5759	TSS_934843+2	14	1	-40	TAGATGTCGG	-14	CGTATGCTTG	-141	1,1	3.63	2.90	*(w)-sba*	867	Rmet_0855	*0*	Op0245f_1	NA
5760	TSS_159431-2	14	2	-37	TCGACCCGAC	-13	GGTATAACCG	-145	1,1	2.16	8.90	s	98	Rmet_0154	*lpxL*	Op0044r_1	MET-GLYC-LPS
5761	TSS_3445785-2	14	2	-23	TCGACCTGTG	0	GACAACGTCG	-141	1,1	3.63	-3.30	*no*	1015	Rmet_3171	*nirM*	Op0890r_1	NA
5762	TSS_883763-3	14	1	-39	CCGGCCACGC	-14	GATAGCGTTC	-140	5,1	7.51	0.90	w	461	Rmet_4291	*prpB*	Op1219r_1	NA
5763	TSS_2033304-2	14	1	-49	TTGCGTGGGC	-14	AGCAAAATCA	-139	1,1	7.57	-14.60	*no*	2196	Rmet_1871	*0*	Op0540r_1	NA
